# Syntheses and Characterization of Main Group, Transition Metal, Lanthanide, and Actinide Complexes of Bidentate Acylpyrazolone Ligands

**DOI:** 10.1021/acs.inorgchem.3c01506

**Published:** 2023-08-07

**Authors:** Thomas Mies, Andrew J. P. White, Henry S. Rzepa, Luciano Barluzzi, Mohit Devgan, Richard A. Layfield, Anthony G. M. Barrett

**Affiliations:** † Department of Chemistry, Imperial College, Molecular Sciences Research Hub, White City Campus, 82 Wood Lane, London W12 0BZ, England; ‡ Department of Chemistry, University of Sussex, Falmer, Brighton BN1 9QR, England

## Abstract

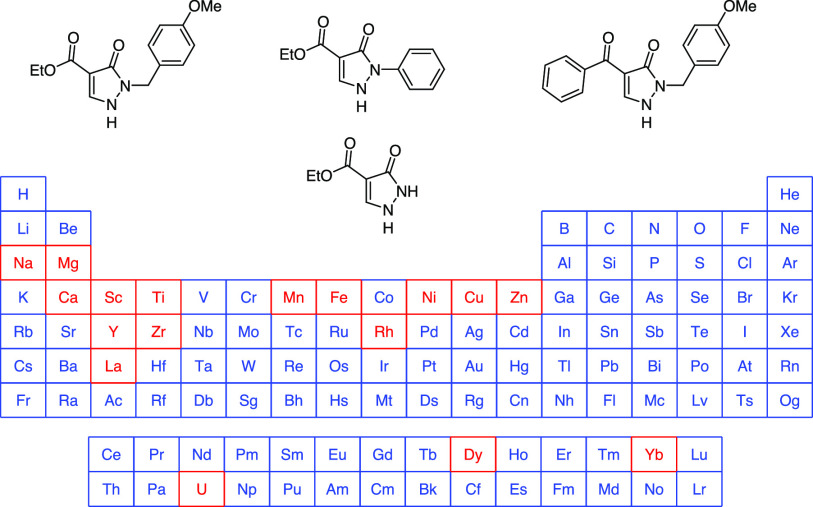

The synthesis of acylpyrazolone salts and their complexes of main group elements, transition metals, lanthanides, and actinides are described and characterized *inter alia* by means of single-crystal X-ray crystallography, NMR, and IR spectroscopies. The complexes consist of two, three, or four acylprazolone ligands bound to the metal atom, resulting in a structurally diverse set of coordination complexes with (distorted) octahedral, pentagonal-bipyramidal, or antiprismatic arrangements. Several complexes proved to be polymeric in the solid state including heterobimetallic sodium/lanthanide coordination polymers. A selection of the polymeric compounds was analyzed via TG/DTA measurements to establish their stability. The ligands, in turn, were readily synthesized in good yields from commercially available hydrazine hydrochloride salts. These findings demonstrate that acylpyrazolone ligands can form complexes with metals of varying ionic radii, highlighted by their utility in other areas such as analytical and metal organic framework chemistry.

## Abstract 2

The syntheses and structural analyses of the representative main group, transition metal, lanthanide, and actinide pyrazolonato complexes highlighted in red are presented.

## Introduction

**Scheme 1:**
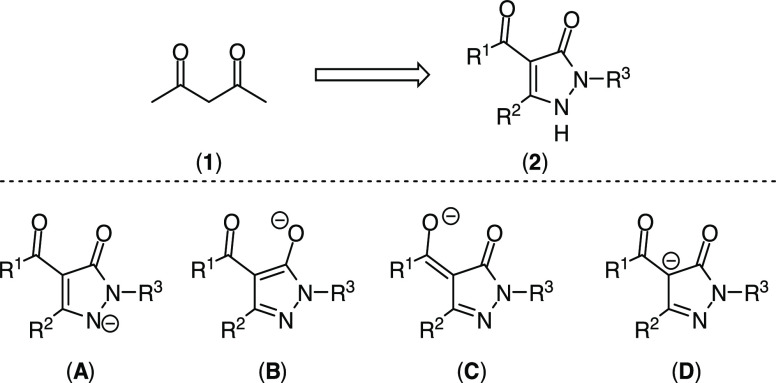
Structures of acac (**1**) and a Generalized Acyl Pyrazolone (**2**) Including the Various Tautomeric Forms **A**–**D** of the Derived Anion

Alternating oligo-carbonyl compounds, such as β-diketones, are well understood bi-, tri-, and higher order-ligands for metal ion complexation due to their *O*,*O*–bidentate chelating coordination properties.^1^ The acetylacetone (acac)H ligand (**1**), Scheme 1, is the archetypical β-diketone, and its longstanding history in metal organic chemistry is mirrored by the multitude of utilization ranging from fundamental research in coordination chemistry to applications, including catalysis, metal extraction, analytical chemistry, water purification, and material science.^1−4^

Fusing the β-dicarbonyl system within a heterocycle, such as pyrazole, gives rise to a subclass of β-diketones, which are known as acyl-pyrazolones (**2**), Scheme 1.^1b,5^ Upon deprotonation, these molecules benefit from an additional potential coordination site on the nitrogen atom (**A**), next to the β-diketone-derived forms of the anion **B**–**D**, and are found to have generally lower p*K*_a_-values than the parent β-diketones. Consequently, ligands derived from acyl-pyrazolones (**2**) were shown to be excellent *O*,*O*-chelators in metal ion extractions via complexes with high-stability constants and low solubility in aqueous media.^5,6^ Furthermore, pyrazolones are commonly used scaffolds in medicinal chemistry, and multiple studies on the biological evaluation of metal-pyrazolone complexes as antitumor agents have been reported.^7,8^

Previously, we reported the synthesis and characterization by single-crystal X-ray and micro-ED structure determinations of a calcium-pyrazolonato complex.^9^ Herein, we wish to report more comprehensive studies on the synthesis and characterization of several acyl-pyrazolones and their coordination chemistry with the representative main group, transition metals, lanthanides and uranium salts.

## Results and Discussion

### Syntheses of Acyl-Pyrazolone Ligands

**Scheme 2:**
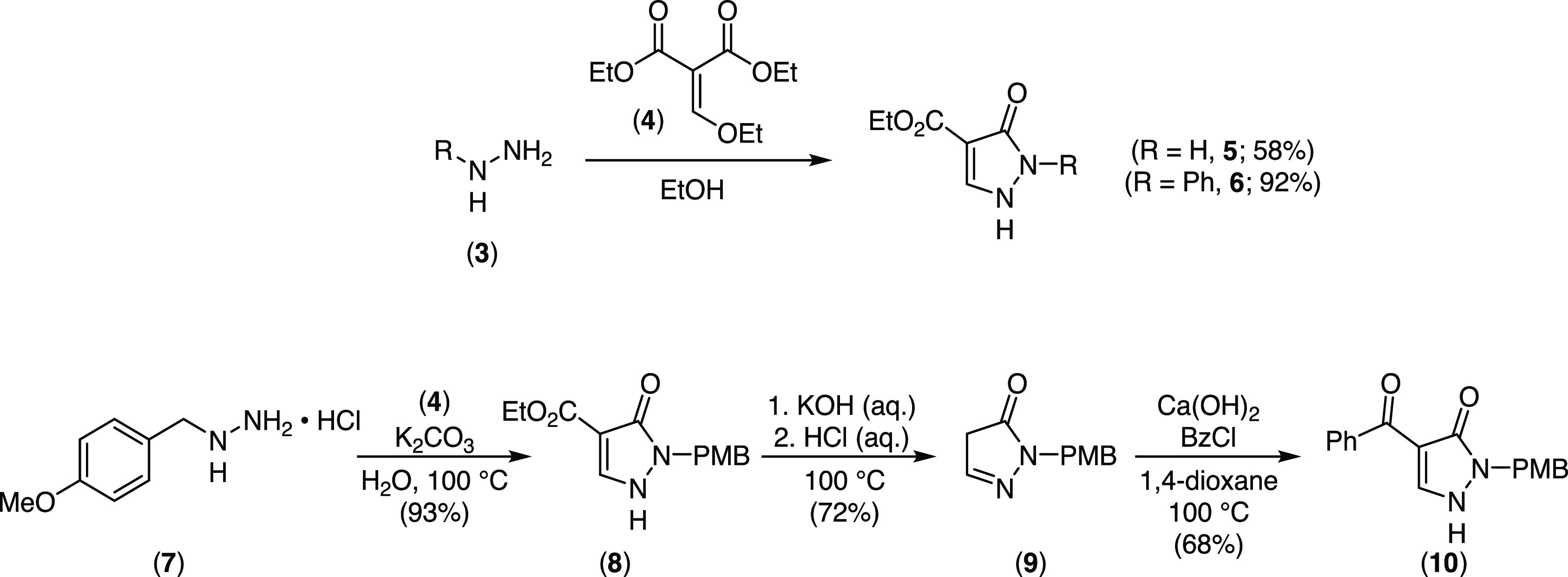
Syntheses of Acyl-Pyrazolone Ligands **5**, **6**, **8**, and **10**

The ligand precursors were prepared from commercially available hydrazine derivatives as described by Holzer and Taylor.^10^ Reaction of hydrazine hydrate or phenylhydrazine with diethyl ethoxymethylenemalonate (**4**) gave the unprotected acylpyrazolone (**5**) and phenyl-protected acylpyrazolone (**6**) in 58 and 92% yields, respectively (Scheme 2). Reaction of 4-methoxybenzyl-(PMB-)hydrazine hydrochloride (**7**) with malonate **4** in water in the presence of potassium carbonate gave acylpyrazolone (**8**) in 93% yield. Saponification with 2 M aqueous potassium hydroxide, followed by acid mediated decarboxylation gave pyrazolone (**9**) (72%). Subsequent reaction with benzoyl chloride and calcium hydroxide in 1,4-dioxane gave benzoyl-pyrazolone (**10**) (68%).

### Syntheses of Acyl-Pyrazolone Metal Complexes

**Scheme 3:**
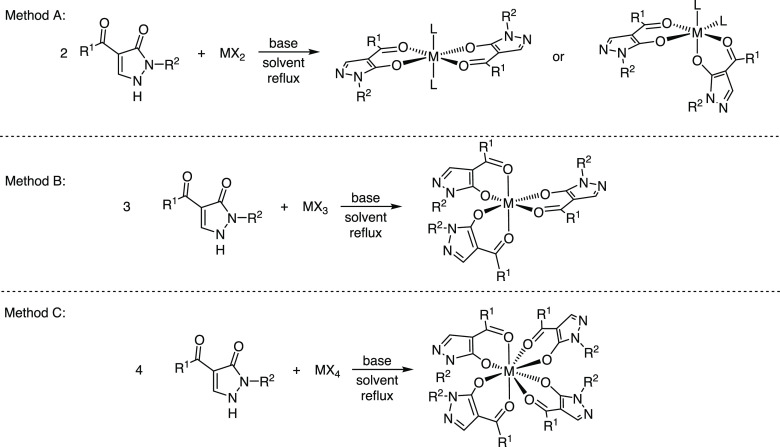
Overview of the General Method to Prepare Acylpyrazolonato Complexes with Di-, Tri-, and Tetravalent Metals Listed in Table 1

Previous syntheses of the acyl-pyrazolone main group and transition metal complexes involved the *in situ* preparation of the alkaline earth metal complexes by reaction with alkali metal hydroxides or methoxides (Li, Na, K), followed by salt metathesis with a second metal salt to provide the target complexes.^5a,5b^ In the context of nucleoside syntheses by *N*-glycosylation reactions, we had occasion to further study these complexation reactions and we initially examined the synthesis of alkaline earth metal complexes (M = Mg, Ca). Since these studies provided us with several interesting metal complexes, we extended our studies to other main group metals, and to transition metals, as well as lanthanide and uranium salts. In the interests of concise presentation, the results are presented with summaries of syntheses and characterizations and with detailed discussions of solid-state structures in the following section. A structurally diverse set of complexes were obtained from the reaction of pyrazolones **5**, **6**, **8**, and **10** with the following metal precursors: sodium methoxide, magnesium chloride, calcium trifluoromethanesulfonate, scandium trifluoromethanesulfonate, yttrium chloride, titanium tetramethoxide, zirconium tetrachloride, zirconocene dichloride, dirhodium tetraacetate, manganous acetate, ferric chloride hexahydrate, zinc acetate dihydrate, cupric acetate hydrate, nickel(II) chloride hexahydrate, lanthanum trifluoromethanesulfonate, dysprosium trifluoromethanesulfonate, ytterbium trifluoromethanesulfonate, and uranyl nitrate hexahydrate (Scheme 3 and Table 1).

**Table 1:**
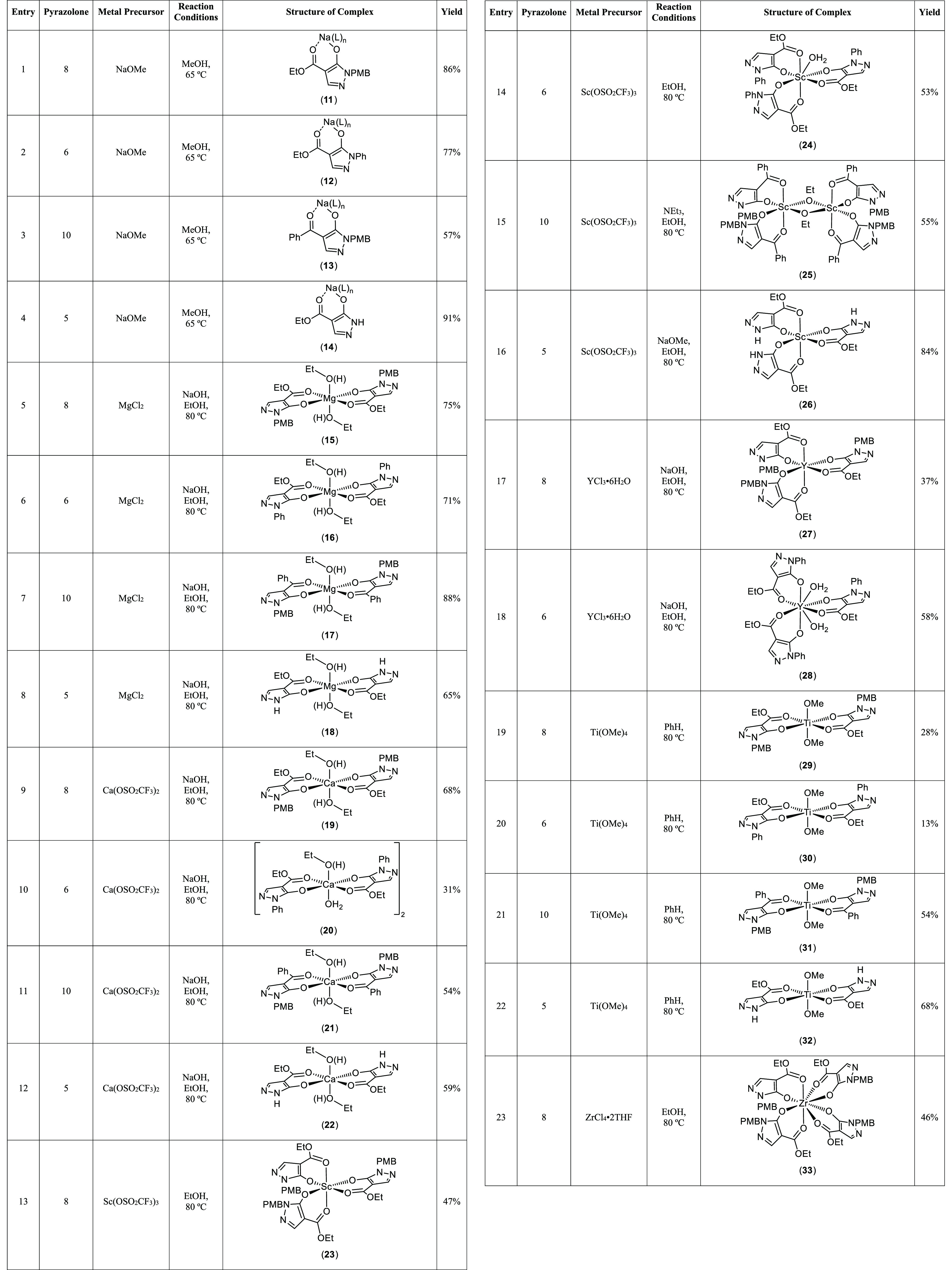

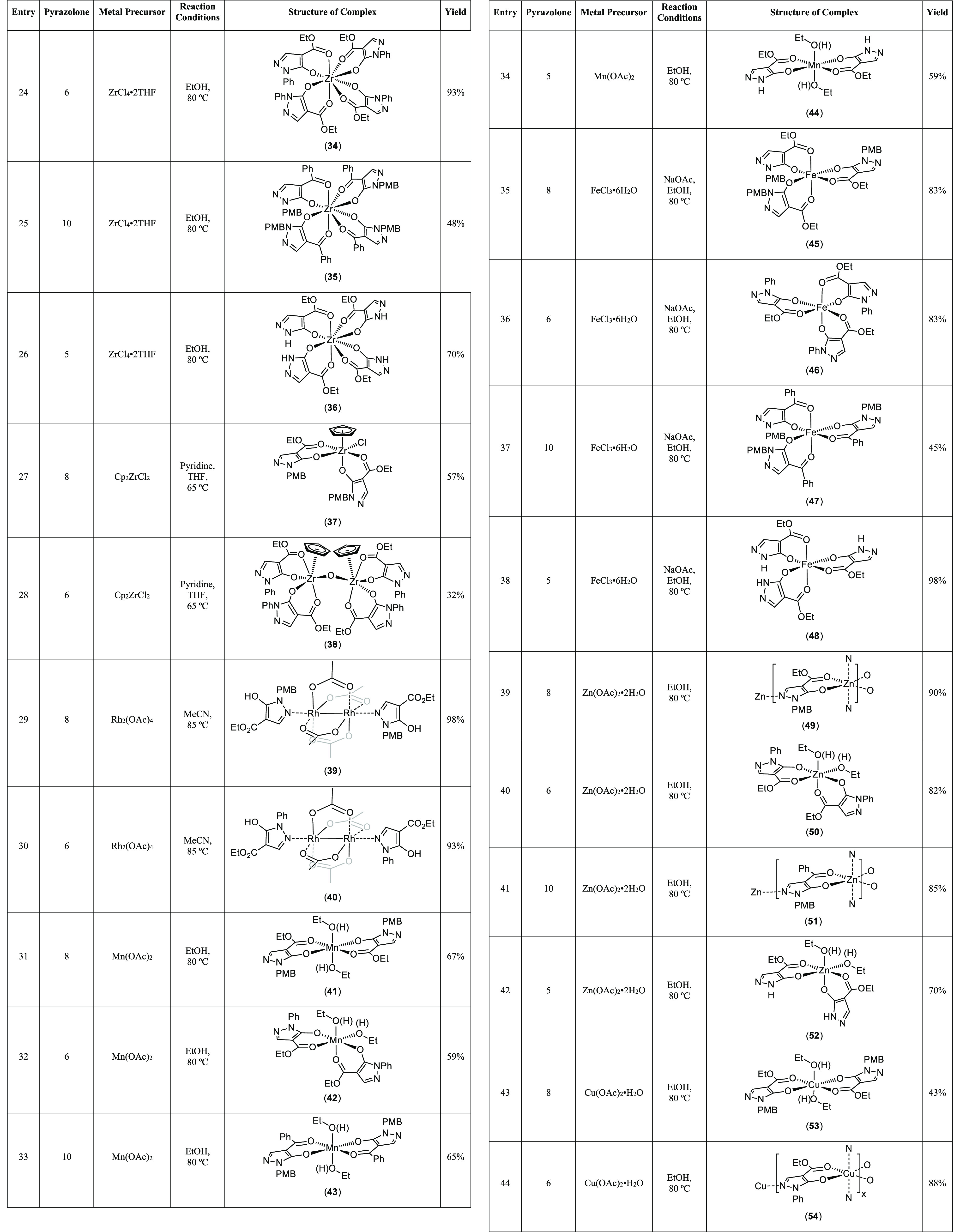
Syntheses of Metal Pyrazolonato Complexes from Pyrazolones **5**, **6**, **8**, and **10**^a^

a M, L, X, R^1^, and R^2^ are defined in the Table. Reaction stoichiometries (pyrazolone:metal precursor) were as follows: 1:1 (entries 1–4); 2:1 (entries 5–12, 19–22, 27, 28, 31–34, 39–50, and 63–66); 3:1 (entries 13–18, 35–38, and 51–62); and 4:1 (entries 23–26, 29, and 30). L = MeOH, EtOH, pyridine, or H_2_O (entries 1–4, 51, 52, 54–56, 58–60, and 62–66).

The ^1^H-NMR spectra of each sodium complex showed coordination-induced shifts for the imine-proton signals. For example, with phenyl-pyrazolone (**6**), the signal shifted from δ = 7.81 to 7.65 ppm in CD_3_OD upon coordination with the sodium cation (**12**). Similar shifts (δ = 7.75 to 7.48 ppm, 7.77 to 7.38 ppm, and 7.83 to 7.46 ppm CD_3_OD) were observed for pyrazolones **11**, **13**, and **14**, respectively, upon formation of the sodium complexes. Related shifts in the ^13^C{^1^H}-NMR signal of the pyrazolone carbonyl group were also observed. DSC measurements of the sodium pyrazolone complexes **11**–**14** revealed phase transitions between 67 and 72 °C, consistent with initial desolvation and possible alteration from an α-phase to a β-phase of the complexes. This phase transition required enthalpies of 3.98 J g^–1^ (complex **11**), 4.62 J g^–1^ (complex **12**), 2.80 J g^–1^ (complex **13**), and 3.53 J g^–1^ (complex **14**).

The reactions of the proligands with alkaline-earth metal salts were conducted according to method A (Scheme 3). Coordination-induced shifts were once more observed in the ^1^H- and ^13^C{^1^H}-NMR spectra, while in the solid state, the compounds are predominantly isostructural. The Mg(II) and Ca(II) ions occupy octahedral coordination sites and reside on a center of symmetry with two pyrazolonato ligands in the *xy*-plane, and with two solvent molecules (EtOH or H_2_O/MeOH) in the axial positions (see the following discussions of X-ray crystal structures).

The scandium and yttrium complexes were prepared according to method B (Scheme 3). Yttrium-complex **27** was found to be isomorphous with the corresponding Ph-protected pyrazolone lanthanum complex **62** and ytterbium-complex **70**, c.f. Table 1, underscoring the structural similarities of these metals. Consequently, the yttrium-complex will be discussed in comparison to the lanthanide complexes.

The syntheses of titanium(IV) complexes were executed according to method A (2 equiv of pyrazolone with titanium tetramethoxide). The ^1^H-NMR spectra indicated characteristic coordination-induced shifts for the imine protons. Furthermore, for titanium complexes **29** and **31**, the benzylic protons are split into a doublet of doublets, indicative of coordination to the metal. The analogous zirconium(IV) complexes were prepared according to method C (Scheme 3). Furthermore, two zirconium half-sandwich complexes were prepared from Cp_2_ZrCl_2_, which was allowed to react with ligands **8** and **6** in the presence of pyridine, giving complexes **37** and **38** in 57% and 32%, respectively.

The reactions of dirhodium tetraacetate with the pyrazalones **8** and **6** at elevated temperatures in acetonitrile resulted in coordination of the pyrazalones ligands at the axial positions giving complexes **39** and **40** in yields of 98% and 93%, respectively. In neither reaction was any substitution of the bridging acetate ligands observed.

Previous studies reported on acylpyrazolone-Fe(III) and -Mn(II) complexes by Okafor revealed high-spin-configured complexes in an octahedral environment as evidenced by magnetic susceptibility studies. Using an Evans/Johnson-Matthey balance, the acylpyrazolone-Fe(III) complexes showed μ_eff_ values of between 5.70 and 5.95 BM,^11a,11b^ while the acylpyrazolone-Mn(II) complexes had μ_eff_ values of between 5.30 and 5.60 BM.^11c^ To further study these observations, reactions of the d^5^ metals manganese(II) and iron(III) were examined with pyrazolones **5**, **6**, **8**, and **10**. The manganese complexes **41**–**44** were synthesized from manganese(II) diacetate according to method A, while the iron complexes **45**–**48** were prepared from ferric chloride hexahydrate according to method B.

The copper(II) and nickel(II) complexes **53**–**56** and **57**–**60**, prepared according to method A, are expected to contain at least one axial solvent molecule. NMR spectroscopic characterization was uninformative since only broad peaks were observed, consistent with the paramagnetic nature of the complexes. Various experiments in common NMR solvents (such as CDCl_3_, CD_3_OD, CD_3_CN, and (CD_3_)_2_CO) failed to provide a spectrum of the complexes and only when dissolved in (CD_3_)_2_SO did the spectrum consist of broad peaks, consistent with the paramagnetic arrangement of the d^8^ electrons. When recrystallized from pyridine, nickel complex **60** was obtained with an octahedral coordination with two pyridine molecules in the axial position replacing ligated ethanol (see the following discussions of X-ray crystal structures). The remaining structures are tentatively assigned as containing two axial ethanol solvent molecules; however, the bulk sample material might be of a polymeric nature,^9^ or without ligated solvent molecules. X-Ray crystal structures of the Cu- or Zn-complexes **49**, **54**, and **55** were found to be polymeric in nature. Ni-pyrazolonato complexes have previously been reported with axial DMF, MeOH, or EtOH ligands.^12^

The reactions of the pyrazolone proligands **5**, **6**, **8**, and **10** with a representative selection of f-block elements were examined, and complexes were prepared according to method B. While complexes **61**, **62**, and **64** gave reasonably well-resolved NMR-spectra, the benzoyl-substituted complex (**63**) showed a broadening of peaks. It is assumed that this could originate from geometrical constraints of the complex, leading to restricted and/or slow bond rotation of the substituents.^13^

DSC measurements of the metal organic framework (MOF)-complex **61** revealed phase transitions at 68 °C, consistent with initial desolvation of the MOF, which required a phase transition enthalpy of 1.43 J g^–1^. Cross validation with TG/DTA measurements confirmed an initial mass-change of 4% (desolvation) followed by negligible changes on the subsequent heating cycles (0.4 and 0.2%). DSC measurements of MOF-complex **65** revealed phase transitions at 68 °C, consistent with initial desolvation of the MOF which required a phase-transition enthalpy of 1.66 J g^–1^. Cross validation with TG/DTA measurements confirmed an initial mass-change of 1% (desolvation) followed by negligible changes on the subsequent heating cycles (0.16 and 0.15%).

### Discussion of Solid-State Structures

#### Alkali-Metal Complexes

**Figure 1:**
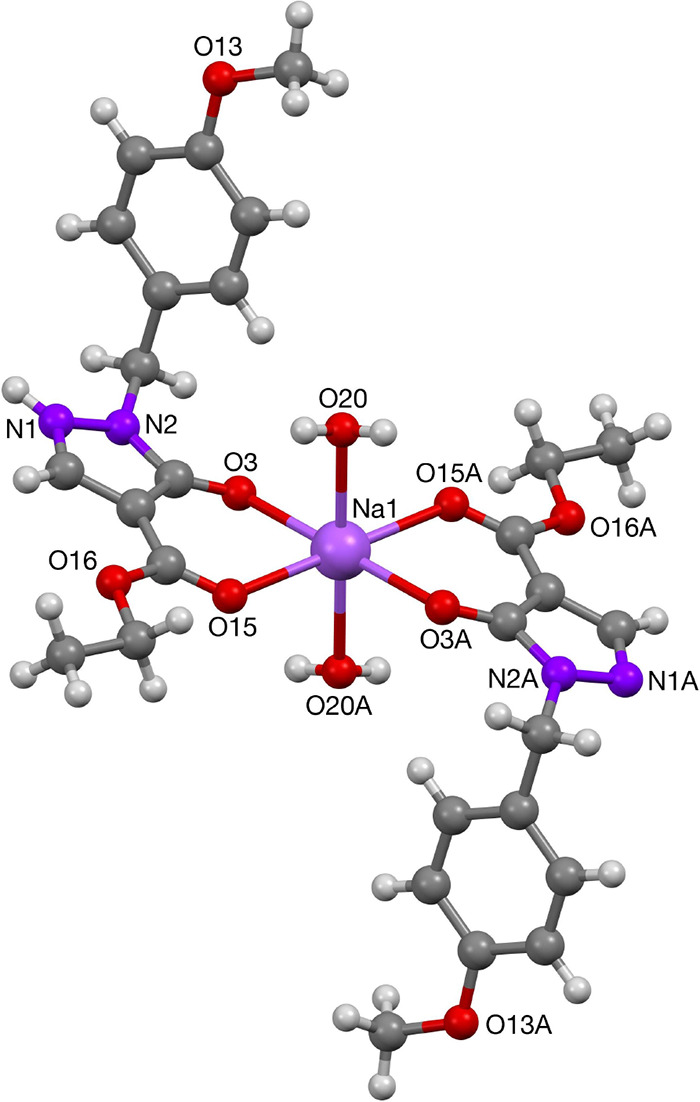
Molecular structure of Na-complex **11**.

**Figure 2:**
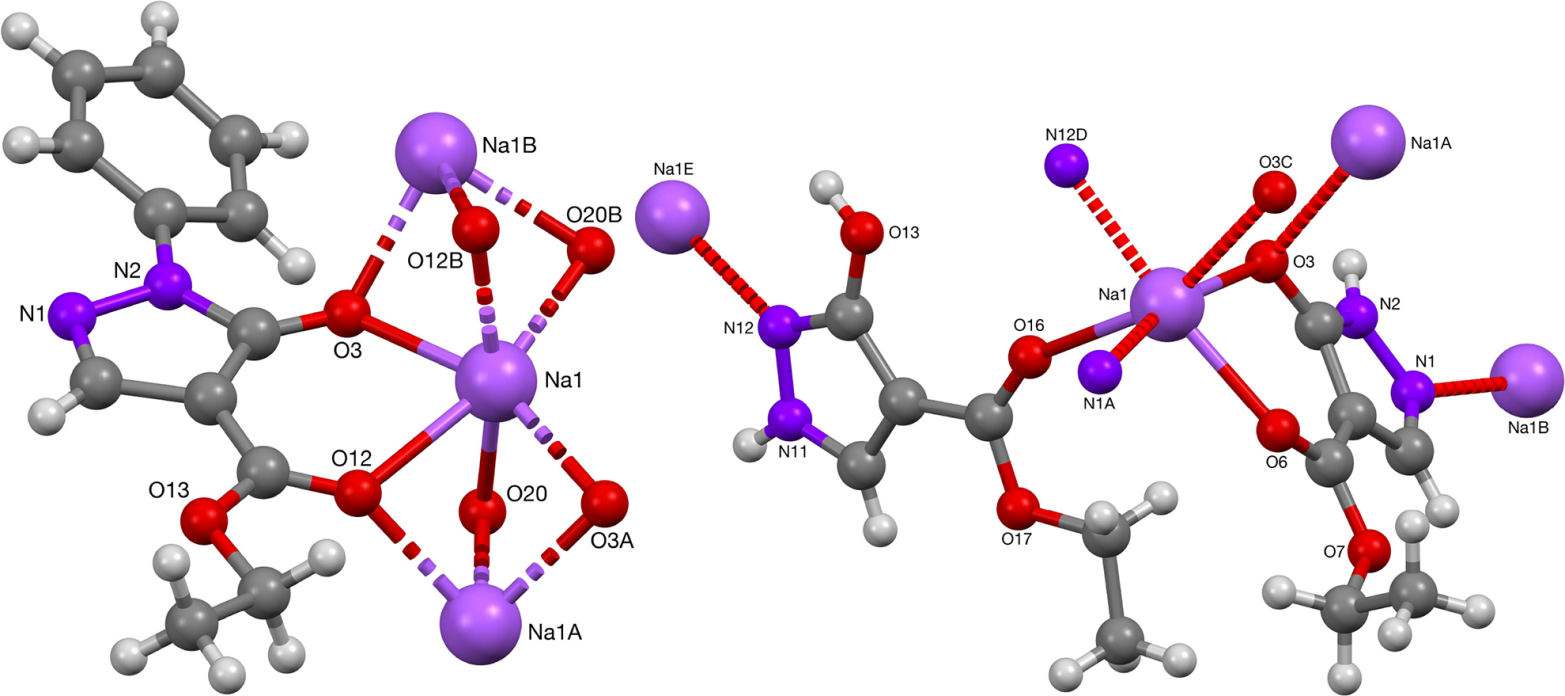
Crystal structures of Na-complexes **12** and **14**.

Selected bond lengths and angles of sodium complexes **11** (Figure 1), and **12** and **14** (Figure 2) are given in Table 2. The C–O bond lengths of the pyrazolonato-ligand were generally comparable to literature values (1.256(2) and 1.245(2) Å, respectively), and thus as reported by Bochkarev, longer than the C=O double bond (1.21 Å).^14^ Similar bond lengths for Na–O bonds (2.335(1) and 2.352(1) Å) were reported for a related dimethoxyethane-coordinated sodium-pyrazolonato complex.^14^ The dihedral angles of the O–Na–O coordination per pyrazolone are slightly larger than the structure reported by Bochkarev and co-workers (77.40(4)°).^14^

**Table 2:** Selected Bond Lengths and Angles for the Alkali-Metal Complexes

selected bond lengths [Å] and angles [°]	**11**	selected bond lengths [Å] and angles [°]	**12**	selected bond lengths [Å] and angles [°]	**14**
M–O^3^/O^3A^	2.3480(9)	M–O^3^/O^3A^	2.353(13)	M–O^3^	2.3133(17)
M–O^15^/O^15A^	2.4555(10)	M–O^12^/O^12A^	2.377(13)/2.444(13)	M–O^6^	2.3542(19)
M–O^20^/O^20A^	2.4496(12)	M–O^20^/O^20A^	2.383(13)/2.443(13)	M–O^16^	2.5258(19)
C–O^3^/O^3A^	1.2522(16)	C–O^3^/O^3A^	1.250(18)	M–N^1^/N^12^	2.375(2)/2.399(2)
C–O^15^/O^15A^	1.2227(16)	C–O^12^/O^12A^	1.24(2)	C–O^3^	1.282(3)
O^3^–M–O^15^	80.33(3)	O^3^–M–O^12^	79.0(4)	C–O^6^/^16^	1.228(3)/1.217(3)
O^3^–M–O^15A^	90.67(3)	O^3^–M–O^12A^	81.9(4)	O^3^–M–O^6^	83.21(6)
O^3^–M–O^20^	85.74(4)	O^3^–M–O^20^	85.3(4)	O^3^–M–O^16^	95.02(7)
O^3^–M–O^20A^	94.26(4)	O^3^–M–O^20A^	114.5(5)	O^3^–M–O^12x^	100.62(7)

The sodium complex **11** has a polymeric structure as a result of intermolecular H-bonding. The repeating unit has an octahedral coordination of the sodium ion with two pyrazolone ligands and two aquo ligands. One of the pyrazolone ligands is charge bearing, while the other one is neutral, and a proton was located in the difference map on the terminal nitrogen. This proton is H-bonding to an adjacent pyrazolone ligand via an N–H–N bond of 2.627(2) Å, giving rise to an H-bonded polymer. Additional X-ray structure determinations of other sodium complexes were measured following crystallization from methanol or ethanol. In the solid state, these complexes also formed polymeric structures, with the sodium center being octahedrally coordinated. The phenyl-protected pyrazolone-sodium complex **12** formed polymers via bridging of the acylpyrazolone ligand with an adjacent repeating unit. The structure resulted in Na···Na separations of 3.110(2) Å. The unprotected-pyrazolone sodium complex **14**, on the other hand, formed polymers by binding to both oxygens of the β-diketone moiety, as well as one of the nitrogens of adjacent pyrazolone rings. This bonding of Na^+^ to the nitrogen atoms was nonspecific, and coordinative bonds via both the N1 or N2 position were found. The Na···Na separation in complex **12** is 3.6155(18) Å, comparable with those of complex **14** (3.110(2) Å) and literature data (3.573(1) Å).^14^

#### Alkaline-Earth Metal Complexes

**Figure 3:**
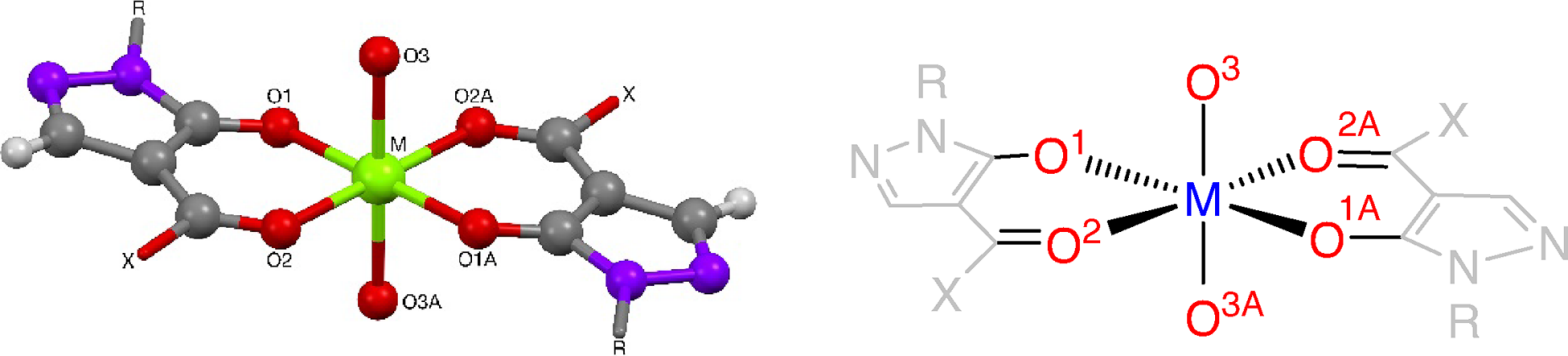
General structure and atom labeling in Mg- and Ca-complexes **15–19**, **21**, and **22** listed in Table 3.

Selected bond lengths and angles for the magnesium and calcium complexes are summarized in Table 3. The metal ion in each complex occupies a slightly distorted octahedral geometry (Figure 3). The bond lengths and angles of the magnesium(II)-complexes compare well to the ones found in [Mg(acac)_2_(H_2_O)_2_] (acac–Mg: 2.040 Å, 2.027 Å; Mg–OH_2_: 2.148 Å)^15^ with the noted distortion along the equatorial Mg–O bond and shorter Mg–O(*H*)Et bond. Notably, in the benzoyl-pyrazolonato magnesium complex **17**, the Mg—O(=C) and the Mg–O(*H*)Et bonds are equidistant (2.107(2) and 2.099(2) Å, respectively).

**Table 3:** Selected Bond Lengths and Angles for the Octahedral Alkaline-Earth Complexes

selected bond lengths [Å] and angles [°]	**15**	**19**	**16**	**17**	**21**	**18**	**22**
M–O^1^/O^1A^	2.0211(10)	2.3116(11)	2.0473(19)	2.050(2)	2.2895(16)	2.0635(9)	2.3048
M–O^2^/O^2A^	2.1295(11)	2.3724(11)	2.075(3)	2.107(2)	2.3370(17)	2.0866(9)	2.3055
M–O^3^/O^3A^	2.0835(11)	2.3296(12)	2.107(3)	2.099(2)	2.3490(18)	2.1063(10)	2.3623
C–O^1^/O^1A^	1.2725(18)	1.2669(19)	1.268(4)	1.268(4)	1.261(3)	1.2816(16)	1.2761
C–O^2^/O^2A^	1.2376(18)	1.2385(19)	1.233(4)	1.255(4)	1.253(3)	1.2386(16)	1.2330
O^1^–M–O^2^	89.77(4)	81.28(4)	89.49(9)	89.34(8)	80.68(6)	88.92(3)	81.06
O^1^–M–O^2A^	90.23(4)	98.72(4)	90.51(9)	90.66(8)	99.32(6)	91.08(3)	98.94
O^1^–M–O^3^	91.32(4)	87.28(4)	89.83(10)	92.40(8)	90.99(6)	90.15(4)	89.87
O^1^–M–O^3A^	88.68(4)	92.72(4)	90.17(10)	87.60(8)	89.01(6)	89.85(4)	90.13

**Figure 4:**
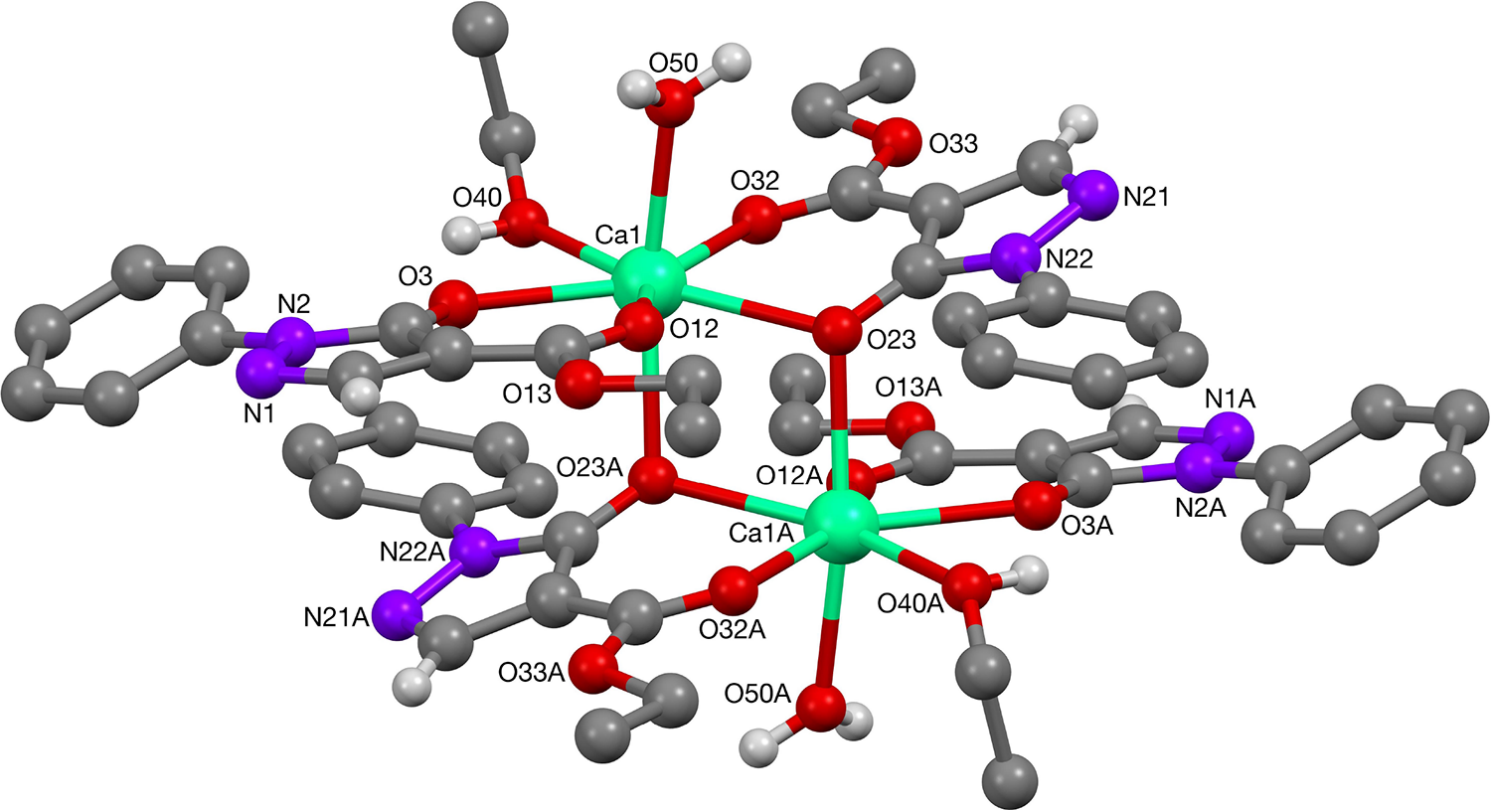
Molecular structure of calcium(II)-complex **20**. Selected bond lengths: Ca^1^–O^3^: 2.4047(11) Å; Ca^1^–O^12^: 2.4314(11) Å; Ca^1^–O^23^: 2.4179(10) Å (part of the Ca_2_O_2_-ring); Ca^1^–O^32^: 2.4397(11) Å; Ca^1^–O^40^: 2.4553(13) Å; Ca^1^–O^50^: 2.3314(11) Å. The Ca_2_O_2_-ring was completed by bond Ca^1^–O^23A^: 2.3829(10) Å, giving a Ca^1^–Ca^1A^ separation of 3.7523(6) Å. Selected bond angles in the Ca_2_O_2_-ring are: O^23A^–Ca^1^–O^23^: 77.19(4)°; Ca^1A^–O^23^–Ca^1^: 102.81(4)°. For clarity, hydrogen atoms bound to carbon are not shown.

The isomorphous calcium(II)-complexes have comparable bond lengths and angles relative to reported literature data of 2.29–2.32 Å, which is typical for Ca–O pyrazolonato bond lengths^8f,16^ and also compares well to reported bond lengths for the Ca–solvent ligand bonds (Ca–O(*H*)Et bond: 2.35–2.38 Å).^8f,16^ The found bond lengths also compare well to the ones found in [Ca(acac)_2_(H_2_O)_2_] (acac–Ca: 2.336(2) Å, 2.320(2) Å; Ca–OH_2_: 2.356(2) Å).^17^ The phenyl-pyrazolone complexes on the other hand gave rise to different structures, while the magnesium complex **16** possessed the expected octahedral coordination sphere (data in Table 2), the corresponding calcium complex **20** had a dimeric, pentagonal-bipyramidal coordination around the calcium center, with two pyrazolonato ligands, a water and ethanol solvent molecule, Figure 4.

To the best of our knowledge, such a structure has not been reported for a calcium pyrazolonato complex, but for the heavier analogs strontium(II) and barium(II) with four bridging ligands, instead of two, giving larger coordination numbers of 8 and higher.^18^ The structure contained a center of symmetry within the Ca_2_O_2_-ring. The bond lengths and angles are comparable to those found in literature for similar coordination environments.^19^

#### Transition Metal Complexes

**Figure 5:**
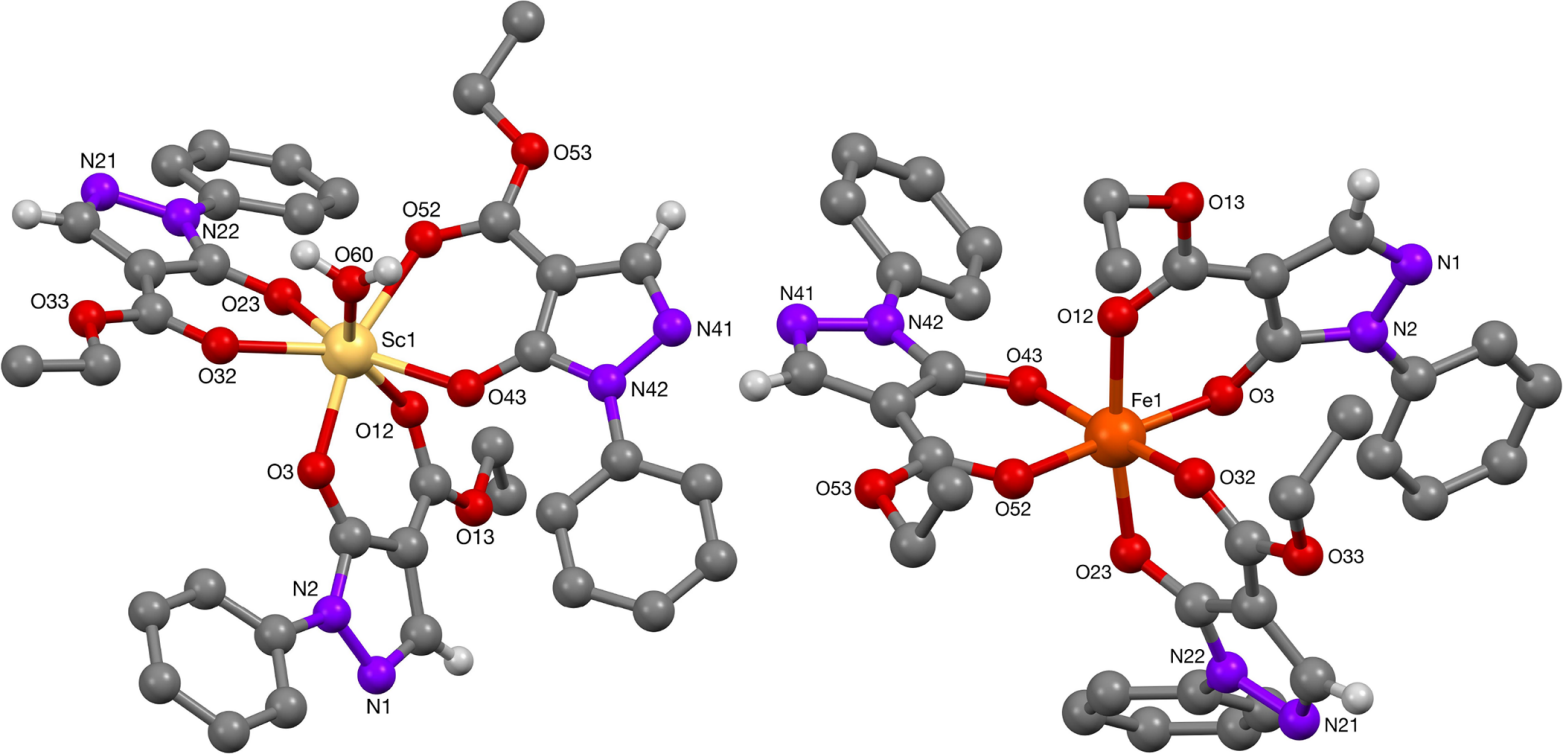
Molecular structures of Sc-complex **24** and Fe-complex **46**.

**Figure 6:**
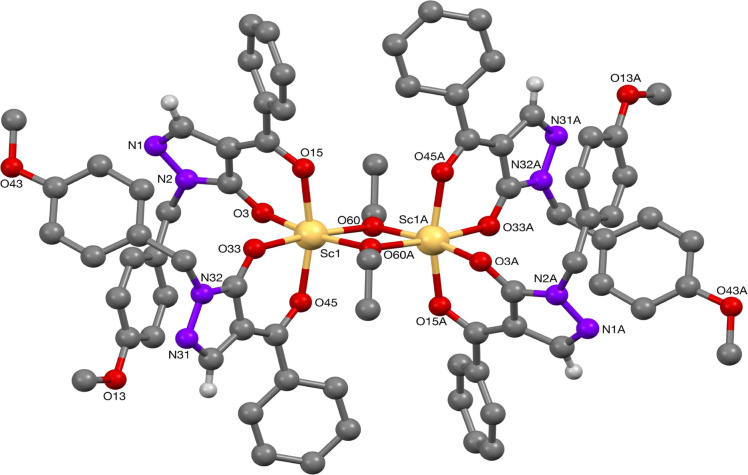
Molecular structure of Sc-complex **25**.

Table 4 consists of a summary of selected bond lengths and angles of the crystal structures for the scandium-complexes **24** (Figure 5) and **25** (Figure 6) as well as the iron complex **46** (Figure 5). In the scandium(III)-complex **24**, the scandium(III)-center is hepta-coordinated with three ligands and an additional water molecule, giving a distorted pentagonal bipyramidal structure. The values of selected bond lengths and angles for complex **24** correspond well to literature reported data on a structurally related *fac*-Sc-complex with an antipyrine-derived phenyl-protected pyrazolonato ligand (which bears a methyl group in the 3-position).^20^ The Sc—O(=C) bond lengths were determined to be 2.082(5), 2.106(5), and 2.104(4) Å, significantly shorter than the one in complex **24** (2.232(6), 2.249(5), and 2.239(5) Å). Presumably, the aquo ligand requires additional space, which shifts the pyrazolonato ligands, resulting in more distorted bond lengths and angles for the pseudo-octahedral complex. This also compares well to the structurally related archetype Sc(acac)_3_, where Sc–O bond lengths of 2.062(6)–2.082(6) Å have been determined, once more with the distinctive difference relative to the Sc—O(=C) bond lengths.^21^ On the other hand, scandium complex **25** formed a dimeric compound, consisting of two octahedral scandium centers bridged by two ethanoate ions to complete charge balance. The structure contains a core Sc_2_O_2_ ring with a C_2_ symmetry axis perpendicular to the ring, similar to the calcium complex **20**. To the best of our knowledge, such a scandium(III)-pyrazolonato dimer has not been reported; however, related compounds include an aqua-malonato scandium(III) (Sc–O bond distances of 2.059(5) and 2.076(5) Å for the Sc_2_O_2_ ring; a Sc···Sc separation of 3.27 Å)^22^ as well as trinuclear scandium disiloxanediolate [{(Ph_2_Si–O)_2_O}_2_Sc_3_(acac)_5_], reported by Edelmann and co-workers (Sc–O(acac) bond distances of 2.095(2) Å, and Sc–O bond distances of 2.130(2) and 1.649(2) Å).^23^

**Table 4:** Selected Bond Lengths and Angles of the Scandium(II)-Pyrazolonato Complexes **24** and **25** as well as Iron(III)-Complex **46**

selected bond lengths [Å] and angles [°]	**24**	**46**	selected bond lengths [Å] and angles [°]	**25**
M–O^3^	2.062(5)	1.9761(15)	M–O^3^/O^3A^	2.0885(15)
M–O^12^	2.232(6)	2.0815(18)	M–O^15^/O^15A^	2.1297(14)
M–O^23^	2.096(5)	1.9484(18)	M–O^33^/O^33A^	2.0960(15)
M–O^32^	2.249(5)	2.0679(17)	M–O^45^/O^45A^	2.1106(14)
M–O^43^	2.144(4)	1.9548(16)	M–O^60^	2.0705(14)
M–O^52^	2.239(5)	2.0500(15)	M–O^60A^	2.0698(14)
M–O^60^	2.183(4)		Sc^1^–Sc^1A^	3.2508(7)
C–O^3^	1.307(9)	1.286(3)	C–O^3^/O^3A^	1.281(3)
C–O^12^	1.274(9)	1.254(3)	C–O^15^/O^15A^	1.265(3)
C–O^23^	1.295(8)	1.300(3)	C–O^33^/O^33A^	1.282(2)
C–O^32^	1.265(8)	1.252(3)	C–O^45^/O^45A^	1.268(2)
C–O^43^	1.275(8)	1.299(3)	C–O^60^	1.422(4)
C–O^52^	1.259(8)	1.254(3)	C–O^60A^	1.433(15)
O^3^–M–O^12^	83.0(2)	88.49(7)	O^3^–M–O^15^	85.06(6)
O^23^–M–O^32^	78.6(2)	91.01(7)	O^33^–M–O^45^	83.18(6)
O^43^–M–O^52^	81.39(18)	89.80(6)	O^60^–M–O^60A^	76.53(6)
O^3^–M–O^60^	106.0(2)		Sc^1^–O60–Sc^1A^	103.47(6)

X-Ray structure determination of complex **46** revealed octahedral coordination of the central iron(III) atom, surrounded by three pyrazolonato ligands. In this arrangement, the complex is assigned as the Λ-isomer. The values reported in Table 4 agree well with those found in literature,^24^ particularly the slight difference in average bond length of the pyrazolonato–*O*–Fe bond of 1.9597(18) Å, being slightly shorter, than the average ester Fe—*O*(=C) bond of 2.066(18) Å. The Fe–O bonds are shorter than the corresponding Sc–O bonds found in complexes **24** and **25** as well as the literature-reported antipyrine-derived scandium(III)-complex.^20a^

**Figure 7:**
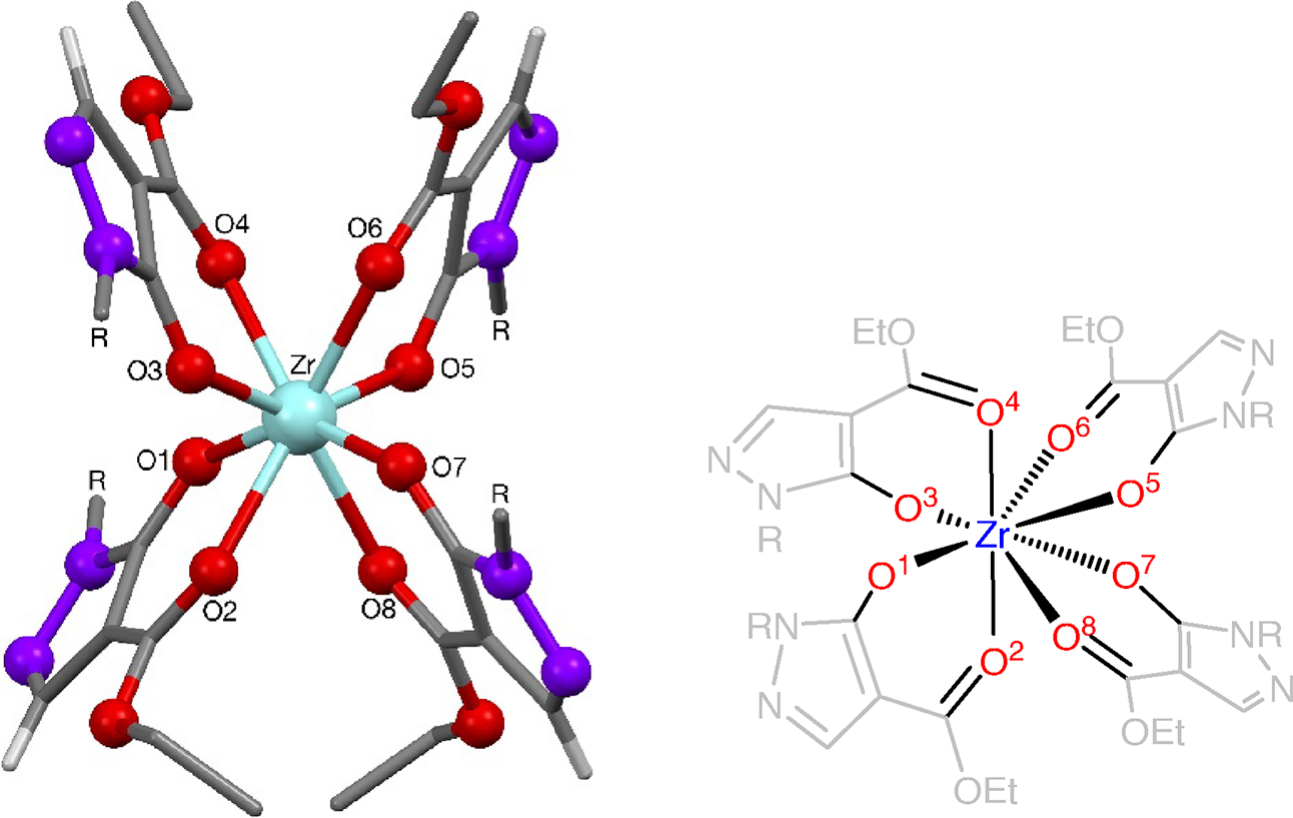
General structure and atom labeling in Zr-complexes **33** and **34** listed in Table 5.

The zirconium complexes **33** and **34** were found to be 8-coordinate, with four ligands around the zirconium(IV)-center, either in an antiprismatic or distorted antiprismatic arrangement (Figure 7). Consequently, those zirconium-pyrazolonato complexes of antiprismatic arrangement contained a center of symmetry. To the best of our knowledge, no structural data have been reported for a zirconium tetrapyrazolonato complex, with spectral data reported for a mixed zirconium pyrazolonato phthalocyaninate complex.^25^ Selected bond lengths and angles can be found in Table 5. The average Zr–O bond length is 2.207(2) Å, with a shortening noted along the Zr–O pyrazolonato bond (2.100(2) Å), and an elongation along the Zr—O(=C) bond (2.313(2) Å). The determined average Zr–O bond lengths of 2.207(2) Å compares well to the one found for Zr(acac)_4_ (2.188(1) Å).^26^

**Table 5:** Selected Bond Lengths and Angles of the Zirconium(IV)-Pyrazolonato Complexes

selected bond lengths [Å] and angles [°]	**33**	**34**
M–O^1^	2.100(2)	2.0878(13)
M–O^2^	2.312(2)	2.2982(13)
M–O^3^	2.107(2)	2.0879(13)
M–O^4^	2.273(2)	2.2983(13)
M–O^5^	2.093(2)	2.0941(13)
M–O^6^	2.305(2)	2.3135(13)
M–O^7^	2.103(2)	2.0941(13)
M–O^8^	2.365(2)	2.3135(13)
C–O^1^	1.303(4)	1.289(2)
C–O^2^	1.243(4)	1.240(2)
C–O^3^	1.288(4)	1.289(2)
C–O^4^	1.239(4)	1.240(2)
C–O^5^	1.293(4)	1.282(2)
C–O^6^	1.239(5)	1.242(2)
C–O^7^	1.296(4)	1.282(2)
C–O^8^	1.235(5)	1.242(2)
O^1^–M–O^2^	79.59(9)	76.90(5)
O^3^–M–O^4^	79.54(9)	76.90(5)
O^1^–M–O^3^	148.78(9)	102.97(5)
O^5^–M–O^6^	77.95(9)	77.52(5)
O^7^–M–O^8^	78.03(9)	77.52(5)

**Figure 8:**
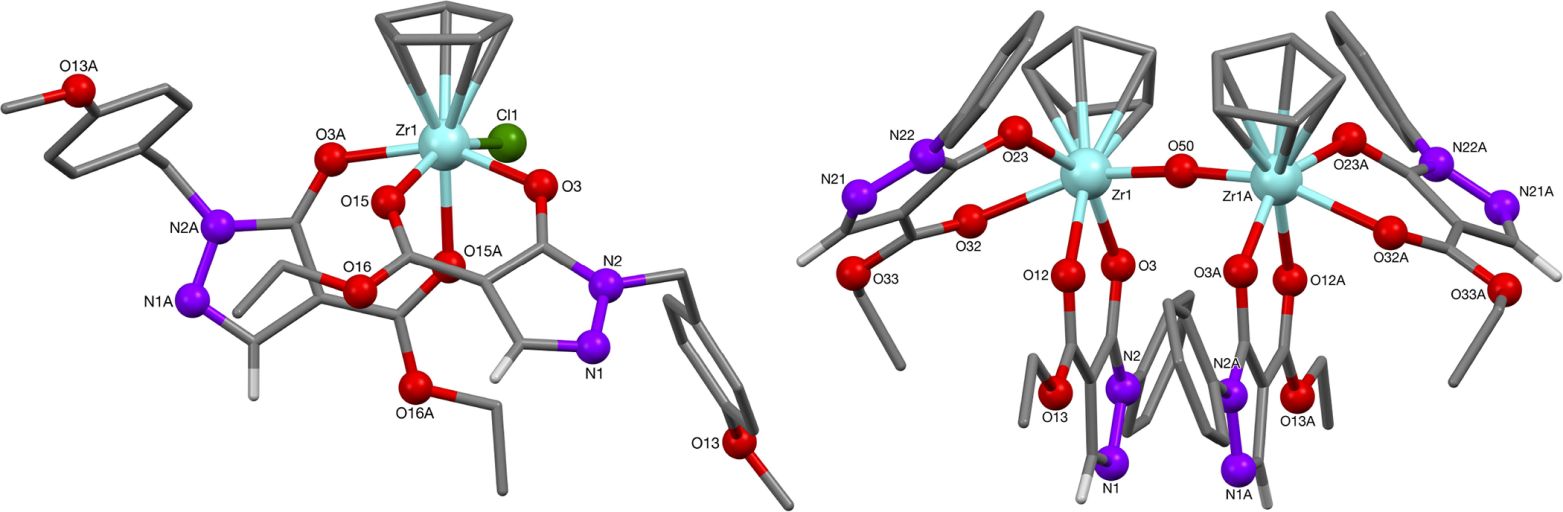
Molecular structures of zirconium half sandwich complexes **37** and **38**.

The zirconium(IV) half-sandwich complexes **37** and **38**, derived from zirconocene dichloride, showed similar Zr–O bond lengths as the complexes **33** and **34**. Selected bond lengths and angles are summarized in Table 6. While complex **37** showed a monomeric structure with two pyrazolonato ligands, one cyclopentadienyl ligand and a chloride, complex **38** is dimeric having lost both chloride ions and being coordinated by four pyrazolonato ligands (Figure 8). The two units were found to be bridged by an oxide ligand, presumably from adventitious water in the reaction medium. The loss of a cyclopentadienyl ligand, presumably as cyclopentadiene, in these reactions most likely resulted by proton transfer from the ligand.^27^

**Table 6:** Selected Bond Lengths and Angles of the Zirconocene-Pyrazolonato Complexes

selected bond lengths [Å] and angles [°]	**37**	selected bond lengths [Å] and angles [°]	**38**
M–O^3^/^3A^	2.1136(12)	M–O^3^/^3A^	2.137(2)
M–O^15^/^15A^	2.2323(12)	M–O^12^/^12A^	2.280(2)
M–CpC^21^	2.469(4)	M–O^23^/^23A^	2.139(2)
M–CpC^22^	2.478(18)	M–O^32^/^32A^	2.293(2)
M–CpC^23^	2.495(4)	M–O^50^	1.9406(3)
M–CpC^24^	2.530(4)	M–CpC^41^/^41A^	2.562(3)
M–CpC^25^	2.497(4)	M–CpC^42^/^42A^	2.552(3)
M–Cl^1^	2.514(4)	M–CpC^43^/^43A^	2.549(3)
C–O^3^	1.296(2)	M–CpC^44^/^44A^	2.526(3)
C–O^15^	1.246(2)	M–CpC^45^/^45A^	2.526(3)
CpC^21^–CpC^22^	1.42(2)	C–O^3^/^3A^	1.277(3)
CpC^22^–CpC^23^	1.408(18)	C–O12/12A	1.240(4)
CpC^23^–CpC^24^	1.387(6)	C–O23/23A	1.291(3)
CpC^24^–CpC^25^	1.407(7)	CpC^41^–CpC^42^	1.387(5)
CpC^25^–CpC^21^	1.401(8)	CpC^42^–CpC^43^	1.415(5)
O^3^–M–O^15^	80.25(4)	CpC^43^–CpC^44^	1.395(5)
O^3^–M–O^15A^	76.65(5)	CpC^44^–CpC^45^	1.400(5)
O^3^–M–Cl^1^	94.53(14)	CpC^45^–CpC^41^	1.399(5)
CpC^21^–CpC^25^–CpC^24^	107.9(4)	O^3^–M–O^12^	79.72(7)
CpC^23^–CpC^22^–CpC^21^	105.6(14)	O^23^–M–O^32^	79.31(8)

**Figure 9:**
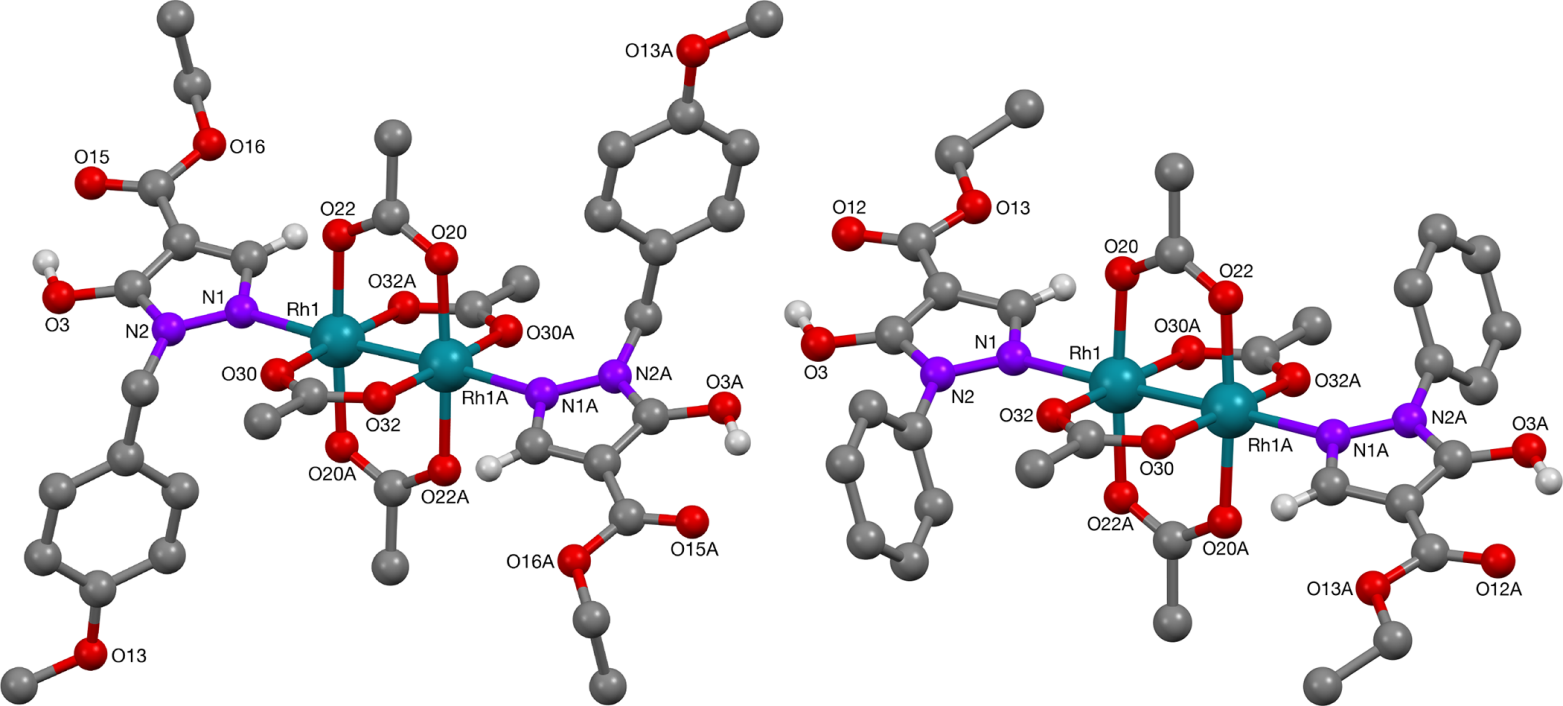
Molecular structures of rhodium-complexes **39** and **40**.

Moving along the 4d transition metal series, the rhodium centers were of slightly distorted octahedral coordination, with four acetate oxygens forming the square-planar arrangement, and the pyrazolone ligand in the axial position bonded via the nitrogen (Figure 9). The Rh–Rh-bond completed the octahedron. Selected bonds lengths and angles of complexes **39** and **40** are summarized in Table 6. The values compare well to those found in the rhodium acetate dimer bis-pyridine adduct (Rh–Rh bond length: 2.3963(2) Å, Rh–N bond length: 2.227(3) Å).^28^

**Table 7:** Selected Bond Lengths and Angles of the Dirhodium(II)-Pyrazolonato Complexes

selected bond lengths [Å] and angles [°]	**39**	selected bond lengths [Å] and angles [°]	**40**
M–O^20^/^20A^	2.031(2)	M–O^20^/^20A^	2.037(2)
M–O^22^/^22A^	2.039(2)	M–O^22^/^22A^	2.039(2)
M–O^30^/^30A^	2.049(2)	M–O^30^/^30A^	2.045(2)
M–O^32^/^32A^	2.034(2)	M–O^32^/^32A^	2.033(2)
M–N^1^	2.278(3)	M–N^1^	2.276(2)
Rh^1^–Rh^1A^	2.3956(5)	Rh^1^–Rh^1A^	2.4015(5)
C–O^3^	1.349(4)	C–O^3^	1.328(4)
C–O^15^	1.224(5)	C–O^12^	1.220(4)
C^21^–O^20^/^22^	1.259(4)	C^21^–O^20^/^22^	1.266(4)/1.262(4)
C^31^–O^30^/^32^	1.263(4)/12.64(4)	C^31^–O^30^/^32^	1.266(4)/1.263(4)
O^20^–M–O^30^	88.69(10)	O^20^–M–O^30^	87.09(9)
O^20^–M–N^1^	93.53(10)	O^20^–M–N^1^	90.90(9)
O^20^–M–O^22A^	176.09(10)	O^20^–M–O^22A^	175.70(9)
O^20^–M–O^32A^	91.35(1)	O^20^–M–O^32A^	91.21(10)
O^22^–M–O^30^	90.61(10)	O^22^–M–O^30^	91.61(10)
O^22^–M–O^32^	89.07(10)	O^22^–M–O^32^	89.79(10)

**Figure 10:**
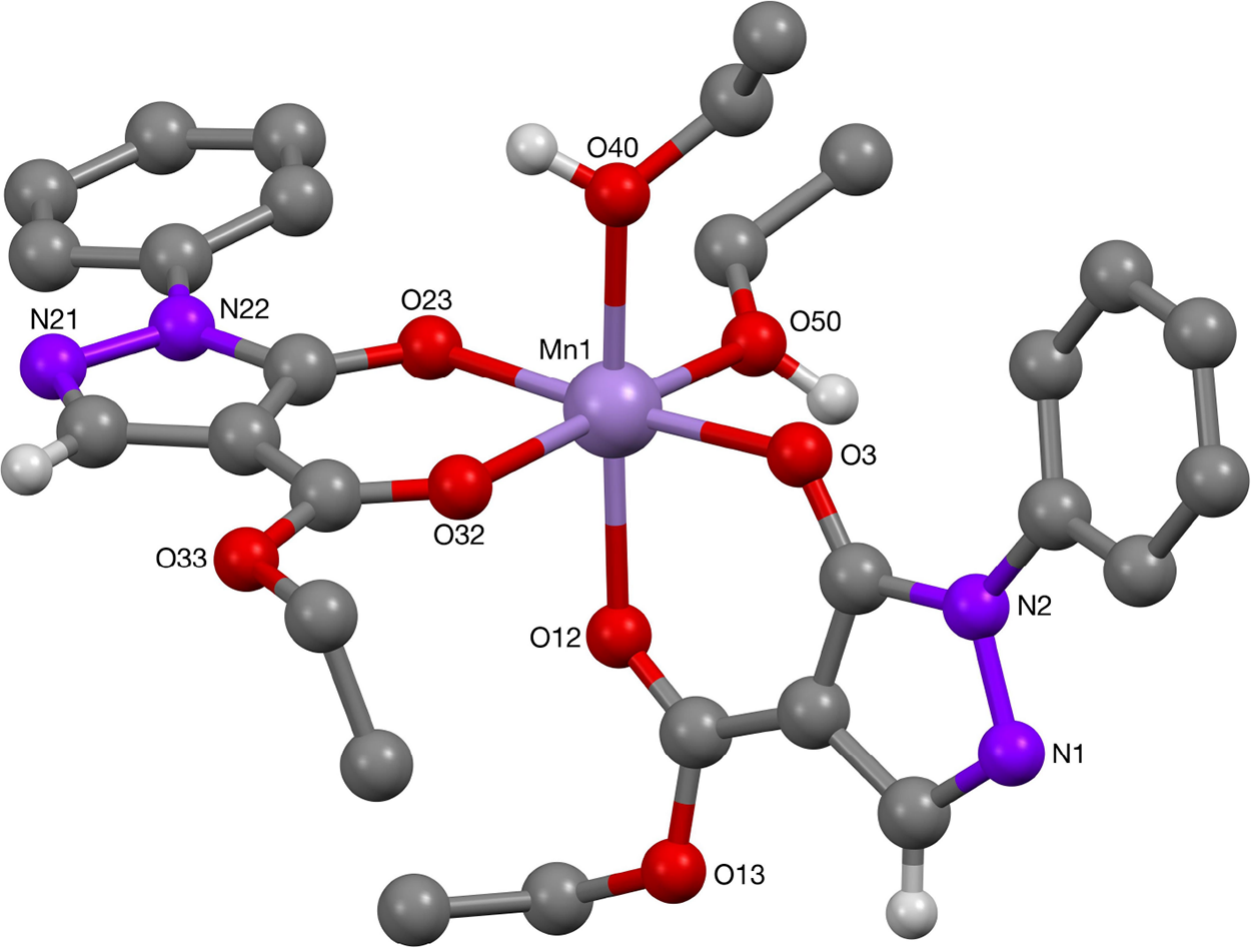
Molecular structure of Mn-complex **42**.

The structure of the phenyl-protected pyrazolonato manganese(II) complex **42** was determined by X-ray crystallography, which showed a slightly distorted octahedral manganese(II)-bis(acylpyrazolonato) complex with a *cis*-configuration of the ligands (Figure 10), contrary to all previously reported pyrazolonato-Mn X-ray structures, with *trans*-stereochemistry of the ligands.^12f,29^ Selected bond lengths and angles are summarized in Table 8, which are in good agreement with those found in literature for the *trans*-configured manganese(II)-pyrazolonato complexes.^12f,29^

**Table 8:** Selected Bond Lengths and Angles of the *cis*-Configured Manganese(II)-, Zinc(II)- and Nickel(II)-Complexes

selected bond lengths [Å] and angles [°]	**42**	**50**	Selected bond lengths [Å] and angles [°]	**58**
M–O^3^	2.1013(19)	1.992(3)	M–O^3^	2.050(2)
M–O^12^	2.210(2)	2.146(3)	M–O^12^	2.080(3)
M–O^23^	2.1029(18)	1.969(3)	M–O^23^	2.025(3)
M–O^32^	2.220(2)	2.169(3)	M–O^32^	2.107(3)
M–O^40^	2.196(2)	2.123(4)	M–N^40^	2.084(3)
M–O^50^	2.201(2)	2.139(3)	M–N^50^	2.071(3)
C–O^3^	1.268(3)	1.277(5)	C–O^3^	1.272(4)
C–O^12^	1.238(3)	1.277(6)	C–O^12^	1.250(5)
C–O^23^	1.265(3)	1.272(6)	C–O^23^	1.285(5)
C–O^32^	1.244(3)	1.246(6)	C–O^32^	1.236(5)
O^3^–M–O^12^	86.94(8)	90.46(14)	O^3^–M–O^12^	92.35(1)
O^23^–M–O^32^	86.58(8)	90.18(13)	O^23^–M–O^32^	90.33(11)
O^40^–M–O^50^	93.25(8)	92.31(13)	N^40^–M–N^50^	91.41(12)
O^3^–M–O^32^	95.23(8)	92.52(13)	O^3^–M–O^32^	87.88(10)

**Figure 11:**
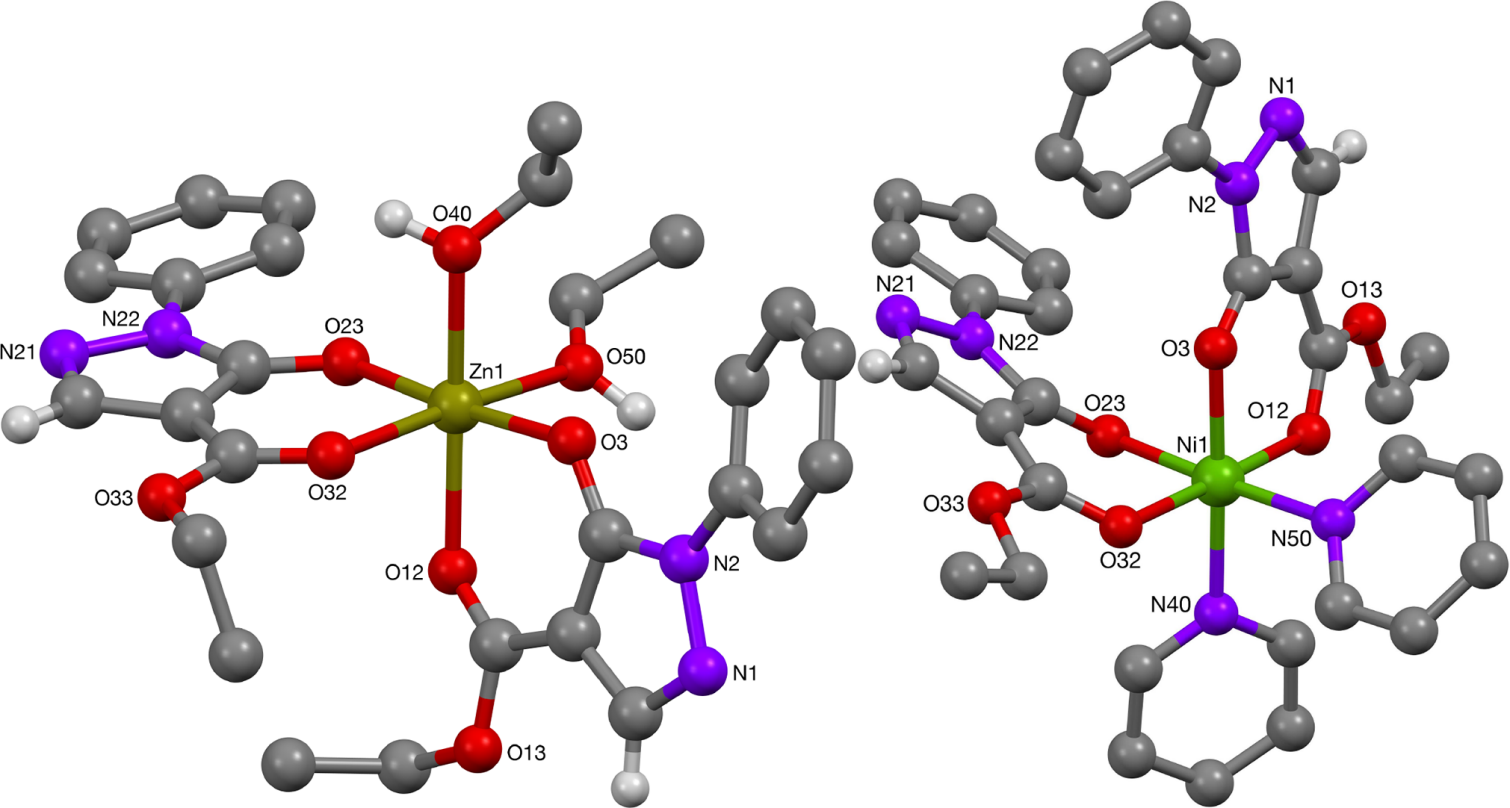
Molecular structures of Zn- and Ni-complexes **50** and **58**, respectively.

The structure of the phenyl-pyrazolone zinc(II) complex **50** showed the complex to have a *cis*-configuration (Figure 11), Table 7. Previous examples of zinc pyrazolonato complexes were reported to have *trans* relationship of the ligands and solvent molecules. In the *cis-*arrangement, the Zn–O bond lengths per pyrazolonato ligand were comparable to the ones of complex **49**, see Table 8. Furthermore, the values compare well to those found in literature of related *trans*-arranged pyrazolonato-zinc complexes of octahedral coordination.^8j,30^ Particularly in a related *cis*-configured zinc(II)-pyrazolonato complex with a *N*,*N*,*N*′-trimethyl-1,2-ethylenediamine ligand (2.046(2) and 2.115(2) Å, and 2.054(2) and 2.115(2) Å, per pyrazolonato ligand; 2.150(2) and 2.192(2) Å for the diamine ligand)^31^ as well as a *cis*-configured zinc(II)-pyrazolonato complex with 1,10-phenanthroline (2.0347(13) and 2.1123(14) Å per pyrazolonato ligand; 2.1908(16) Å for Zn–N(phen)),^32^ the bond lengths were found to be in agreement with those of complex **50**. Zinc(II)-pyrazolonato complexes of square-planar and square-pyramidal geometry were found to exhibit similar Zn–O bond lengths.^8j,32,33^

Crystals of the nickel(II) complex **58** suitable for X-ray crystallographic analysis were obtained after recrystallization from pyridine. Complex **58** is octahedral with the two pyrazolonato ligands in a *cis*-arrangement, Figure 11. The structures of related compounds display Ni–O bond lengths of 1.9966(12)–2.0442(17) Å, while the solvent ligands were reported with bond distances to the Ni(II) center of 2.0651(16)–2.042(12) Å.^12^

**Figure 12:**
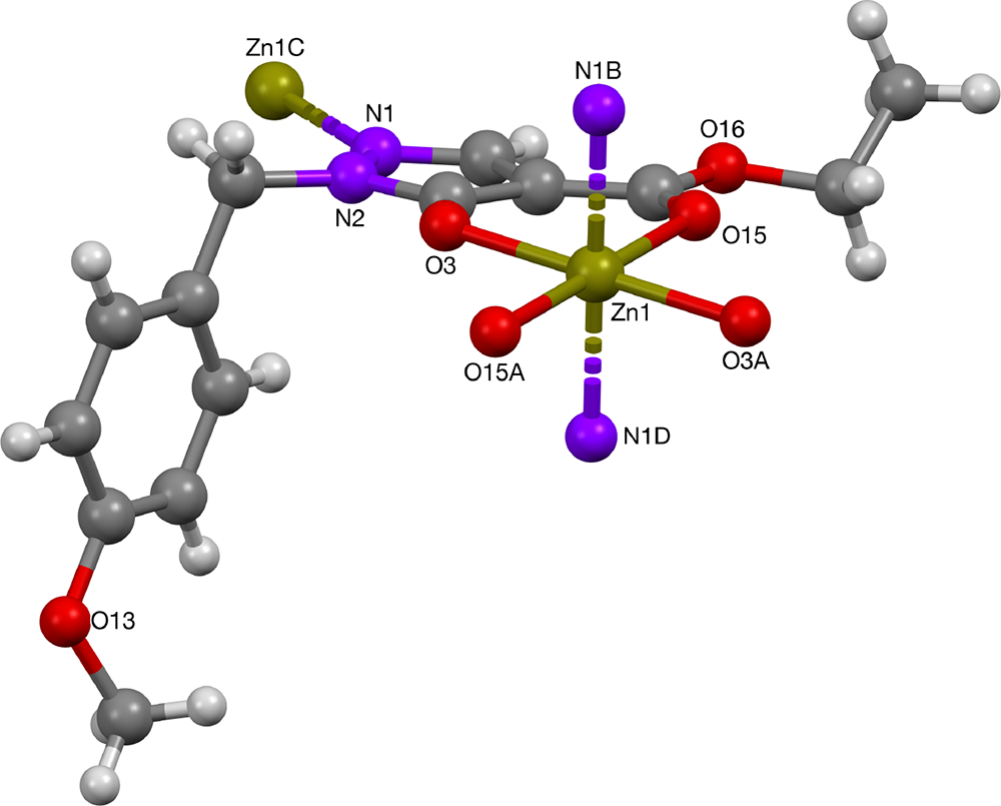
Crystal structure of Zn-complex **49**.

The zinc complex **49** with the PMB-protected ligand has a polymeric structure in the solid state (Figure 12). The zinc(II) center is octahedrally coordinated, with the pyrazolonato ligand in the plane, while the axial positions were occupied by nitrogen atoms of ligands on neighboring molecules. This polymeric structure was evident in the solution as well in that attempts to record ^1^H-NMR spectra on complex **59** in solvents such as CDCl_3_, CD_3_OD, CD_3_CN, and (CD_3_)_2_CO all failed, and only when dissolved in (CD_3_)_2_SO was it possible to obtain NMR data. This only showed the heterocyclic bidentate ligand, with no other ligands present, further confirming the absence of any solvent molecules in the complex. Similar NMR behavior was observed for the benzoyl-substituted pyrazolonato complex **51**, although no X-ray crystal structure determination could be obtained for this complex.

The zinc(II) ion in complex **49** possessed a slightly distorted octahedral coordination, with two sites occupied by the pyrazolonato ligands, and the other two coordination sites were occupied by the pyrazolonato-nitrogen atoms (cf. the sodium complex **14**). Selected bond lengths and angles are summarized in Table 9. The values are in good agreement with the zinc(II) complex **50** and compare well to those reported in the literature.^8j,30−33^

**Table 9:** Selected Bond Lengths and Angles of the Polymeric Zinc(II) and Copper(II)-Complexes

selected bond lengths [Å] and angles [°]	**49**	selected bond lengths [Å] and angles [°]	**54**	selected bond lengths [Å] and angles [°]	**55**
M–O^3^/^3A^	2.0737(13)	M^1A^–O^3^/^3A^	1.927(2)	M^1A^–O^3^/^33^	1.948(4)
M–O^15^/^15A^	2.1381(13)/2.1382(13)	M^1A^–O^12^/^12A^	2.001(2)	M^1A^–O^15^/^45^	1.985(4)/2.000(4)
M–N^1B^/^1D^	2.1719(16)	M^1A^–N^1B^/^1D^	2.519(3)	M^1A^–N^1A^/^31C^	2.458(4)/2.626(4)
C–O^3^	1.272(2)	C–O^3^	1.275(4)	C–O^3^	1.271(6)
C–O^15^	1.235(2)	C–O^12^	1.249(4)	C–O^12^	1.268(6)
O^3^–M–O^15^	92.40(5)	O^3^–M–O^12^	94.59(9)	O^3^–M–O^15^	94.83(15)
O^3A^–M–O^15^	87.60(5)	O^3A^–M–O^12^	85.41(9)	O^33^–M–O^15^	85.58(16)
O^3^–M–N^1B^	91.19(6)	O^3^–M–N^1B^	94.44(10)	O^3^–M–N^1A^	84.12(15)
O^3A^–M–N^1B^	88.81(6)	O^3A^–M–N^1B^	85.56(10)	O^33^–M–N^31C^	89.02(16)
O^15^–M–N^1B^	86.78(6)	O^12^–M–N^1B^	92.37(9)	O^15^–M–N^1A^	82.05(14)
O^15A^–M–O^1B^	93.22(6)	O^15A^–M–N^1B^	87.63(9)	O^15^–M–N^31C^	97.17(15)

**Figure 13:**
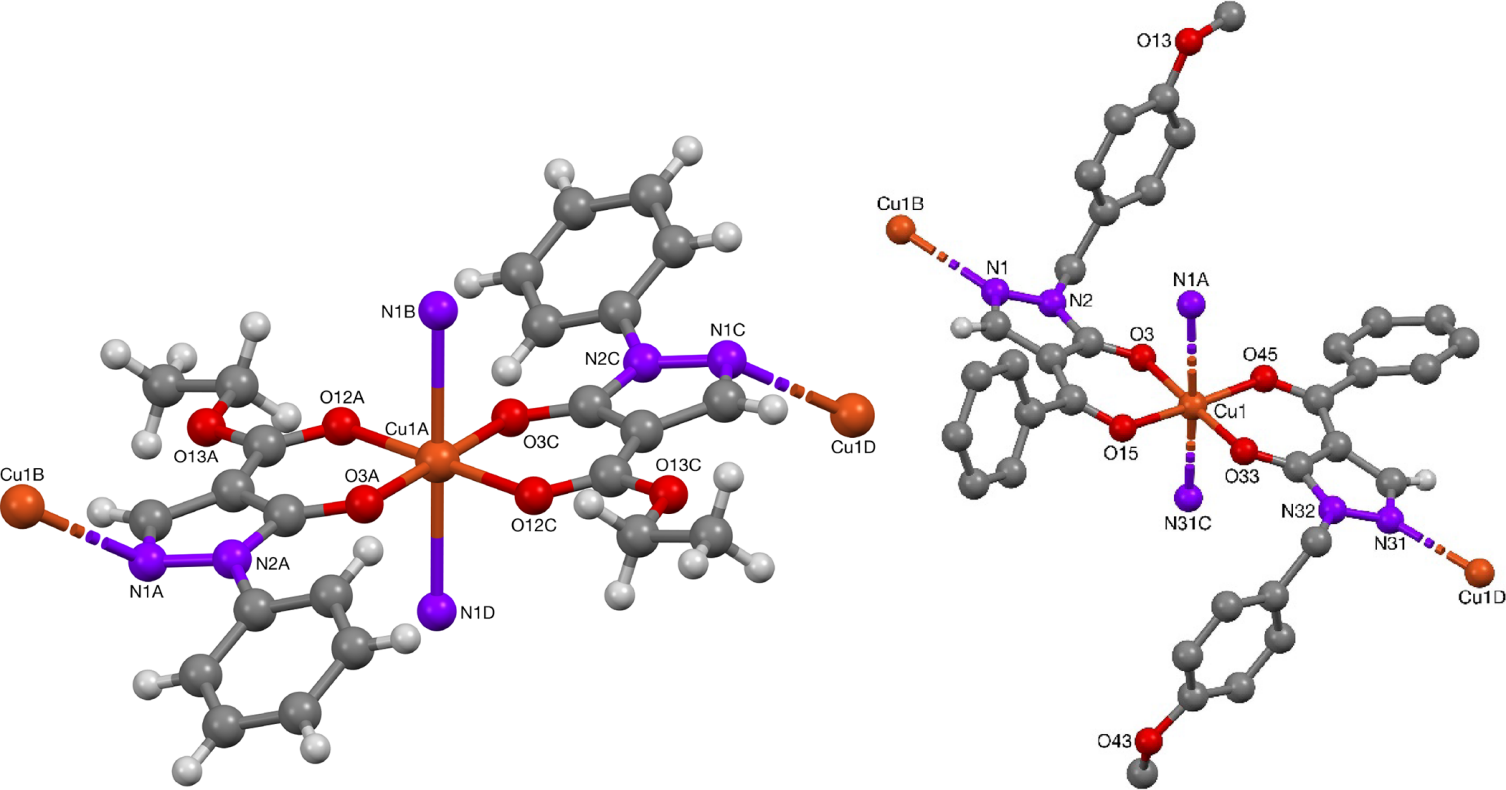
Crystal structures of Cu-complexes **54** and **55**.

The structurally related phenyl-protected pyrazolone copper(II)-complex **54** and benzoyl substituted pyrazolone copper(II) complex **55** were characterized after recrystallization from ethanol and ethanol/pyridine/DMF, respectively, and were found to have a polymeric structure in the solid state (Figure 13). The central copper(II) ions are octahedrally coordinated, with the pyrazolonato ligand in the horizontal plane, while the axial positions are occupied by nitrogen atoms of neighboring ligand molecules, Table 9. Cu–N bond lengths of 2.519(3) Å in complex **54** and 2.458(4)/2.626(4) Å for complex **55** are consistent with Jahn-Teller distortion, with shortened Cu–O bond lengths of 1.927(2) and 2.001(2) Å (complex **54**) and 1.948(4)/1.985(4)/2.000(4) Å (complex **55**). Goetz-Grandmont et al. reported the crystal structure of a square-planar copper-pyrazolonato complex with two centrosymmetric molecules in the asymmetric unit.^34a^ These showed comparable Cu–O bond lengths of 1.912(8)–1.939(8) Å. Furthermore, Jahn-Teller distorted bonding between the two units was observed, displaying a Cu–O bond length of 2.546(8) Å.

**Figure 14:**
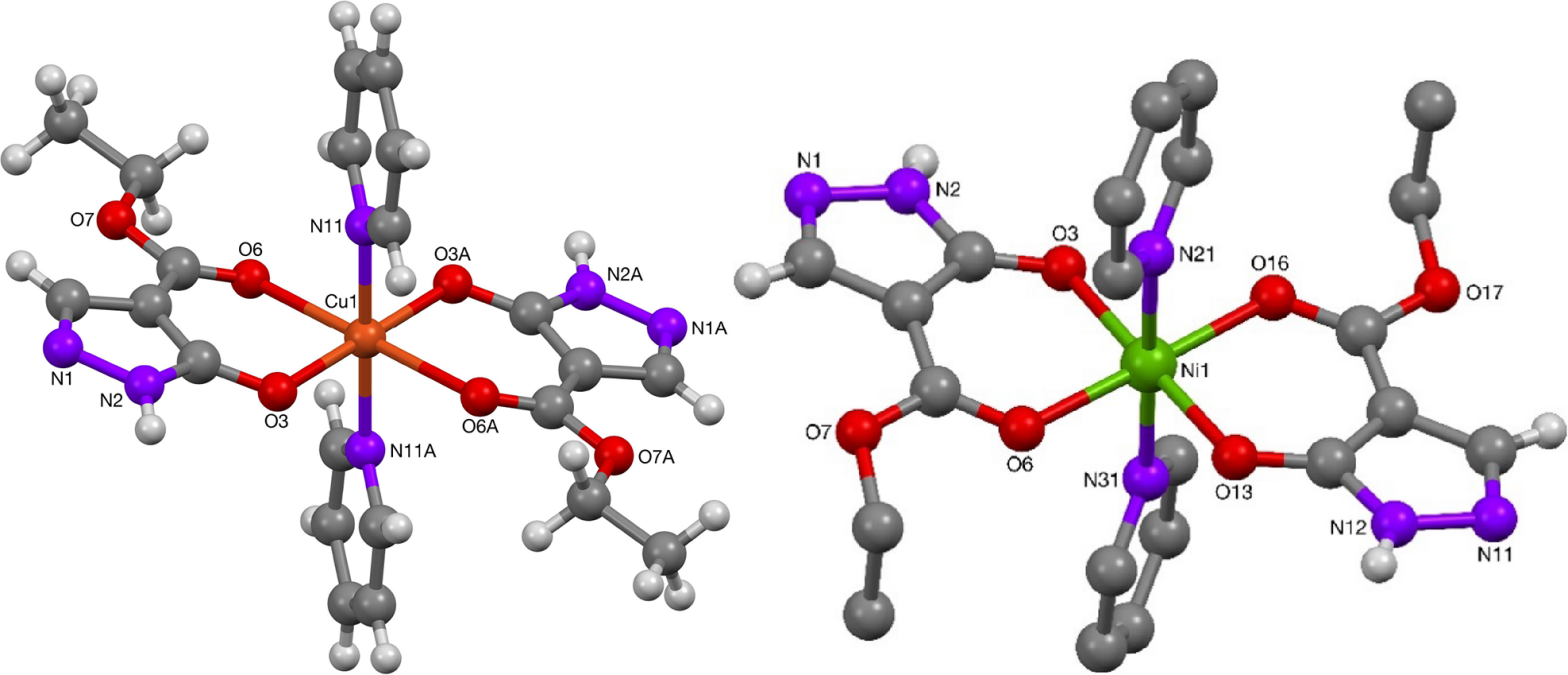
Molecular structures of Cu- and Ni-complexes **56** and **60**.

**Figure 15:**
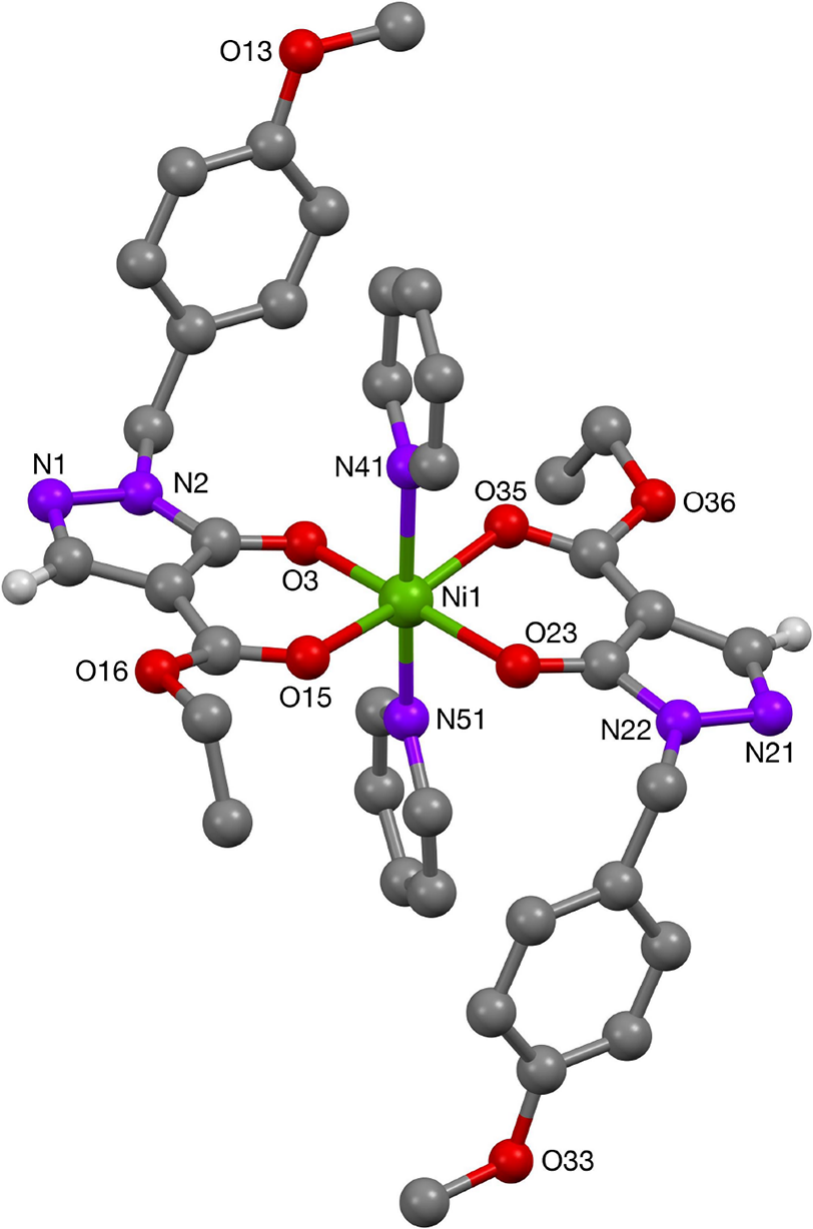
Molecular structure of Ni-complex **57**.

Crystals of the nickel(II) complexes **60** (Figure 14) and **57** (Figure 15) were obtained after recrystallization from pyridine. Crystallographic analysis revealed that the nickel(II) complexes were of slightly distorted octahedral coordination with the two pyrazolonato ligands in a *trans*-arrangement, Table 10. The reported values correspond reasonably well to reported data with structures, which have been shown to display Ni–O bond lengths of 1.9966(12)–2.0442(17) Å, while the solvent ligands were reported with bond distances to the Ni(II) center of 2.0651(16)–2.042(12) Å.^12^

**Table 10:** Selected Bond Lengths and Angles of Pyridine-Coordinated Nickel(II) and Copper(II) Complexes

selected bond lengths [Å] and angles [°]	**56**	selected bond lengths [Å] and angles [°]	**60**	selected bond lengths [Å] and angles [°]	**57**
M–O^3^/^3A^	1.9790(13)	M–O^3^/^13^	2.0613(16)/2.0686(15)	M–O^3^/^23^	1.96(3)/2.11(3)
M–O^6^/^6A^	2.3783(13)	M–O^6^/^16^	2.0726(15)/2.0785(15)	M–O^15^/^35^	1.090(16)/2.052(16)
M–N^11^/^11A^	2.0310(16)/2.0311(16)	M–N^21^/^31^	2.088(2)/2.0895(19)	M–N^41^/^51^	2.16(2)/2.01(2)
C–O^3^	1.295(2)	C–O^3^/^13^	1.284(3)/1.282(3)	C–O^3^/^23^	1.267(11)/1.266(10)
C–O^6^	1.225(2)	C–O^6^/^16^	1.243(3)/1.246(3)	C–O^15^/^35^	1.238(12)/1.249(10)
O^3^–M–O^6^	88.99(5)	O^3^–M–O^6^	93.65(6)	O^3^–M–O^15^	96.0(9)
O^3A^–M–O^6^	91.01(5)	O^13^–M–O^16^	94.11(6)	O^23^–M–O^35^	92.2(9)
O^3^–M–N^11^	92.14(6)	O^13^–M–O^6^	86.97(6)	O^23^–M–O^15^	86.0(8)
O^3A^–M–N^11^	87.86(6)	O^3^–M–N^21^	89.94(7)	O^3^–M–N^41^	90.2(13)
		O^13^–M–N^21^	89.05(7)	O^23^–M–N^21^	92.4(12)

Recrystallization of the copper(II) complexes **53** and **56** (Figure 14) from pyridine gave crystals suitable for X-ray crystal structure determinations. These were consistent with slightly distorted octahedral coordination with two pyridine solvent molecules *trans* to each other in the remaining coordination sites. Complex **56** was found to be Jahn-Teller distorted along the equatorial pyrazolone Cu–O bond with a Cu–O bond length of 2.3783(13) Å. The values reported in Table 10 compare well to a known antipyrine-derived copper complex with two phenyl-protected pyrazolonato ligands and two pyridine ligands.^34c^ Related copper(II) pyrazolonato complexes have been reported with distorted octahedral coordination^33b,34b,34c^ as well as square-planar,^8f,12f,33b,34a^ or square-pyramidal cores.^16b^ In these octahedral complexes, Cu–O bonds lengths were reported to be 1.953(3)–1.979(3) and 2.259(4)–2.383(4) Å with distortion along the C—O(=C) bond and with additional donor ligands (pyridine, bipy, phen) at a distance of 2.004(2)–2.0308(16) Å. Dey et al. reported a copper(II)-pyrazolonato complex with two methanol ligands that showed two shorter bonds of 2.033(3) Å, and four longer Cu–O bonds at 2.108(4)–2.152(4) Å. The square-planar complexes were reported with Cu–O bond lengths of 1.907(8)–1.939(8) Å, while the square-pyramidal complex showed Cu–O bond lengths of 1.911(3)–1.956(3) Å with Jahn-Teller distorted Cu–O bonds of 2.248(4) Å.

This unusual discrepancy of Cu–O bond elongation over Cu–N bond elongation in complex **56**, while the polymeric complexes **54** and **55** showed Cu–N elongation over Cu–O elongation are noteworthy. In addition, literature reports Jahn-Teller distorted Cu–O intermolecular binding instead of C–N binding. Before undertaking DFT calculations (at the M062X/Def2-SVP level)^50^ to provide an insight into the Jahn-Teller distorted bond lengths of the synthesized complexes, we examined their statistical distribution in octahedral copper(II) complexes exhibiting four di-axial Cu–O bonds and two di-axial Cu–N bonds, as found in the Cambridge Structural Database (CSD). This revealed (April, 2023) 1412 error-free structures where the shortened Jahn-Teller distorted O–Cu(II) bonds are ∼1.9 Å and the elongated Jahn-Teller distorted O–Cu bonds are 2.4–2.6 Å, as in complex **56**. The most probable distribution (SI Figure S107) is where one pair of O–Cu bonds is ∼0.6 Å longer than the other, but with a significant, albeit smaller and compact, distribution where both pairs of O–Cu bonds have the same length of ∼1.9 Å and yet another where they both have a length of ∼2.2 Å. A plot of the mean of N–Cu–N lengths vs the mean of O–Cu–O lengths (SI Figure S108) shows a hot spot of both short N–Cu lengths and O–Cu lengths, (that observed for, e.g., complex **56**) with an accompanying but more diffuse distribution with short N–Cu lengths and long O–Cu lengths. Significantly fewer structures were found that displayed shortened O–Cu bonds with longer Cu–N bonds, as in complex **54**, suggesting that the O–Cu elongated case in complex **56** is statistically more commonly found in the literature. Correlations of these bond lengths to bond strengths have been reported.^35^

**Figure 16:**
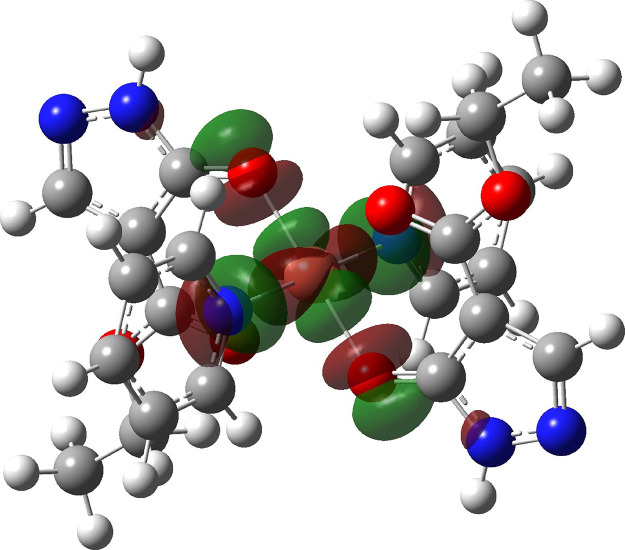
SOMO (iso-surface value is 0.02au) of copper(II) complex (**56**).

The DFT calculations matched this observation, with a starting geometry containing a N–Cu bond-elongated isomer of complex **56** collapsing without activation on optimization to the N–Cu shortened isomer. The form of the SOMO is dominated by the antibonding interaction of the d_*x*_^2^_–*y*_^2^ orbital on copper(II) with the lone pair of the pyridine nitrogen and a lone pair on the pyrazolonato oxygen in a square-planar arrangement (Figure 16).

#### Lanthanide/Rare Earth and Actinide Complexes of Acylpyrazolone Ligands

**Figure 17:**
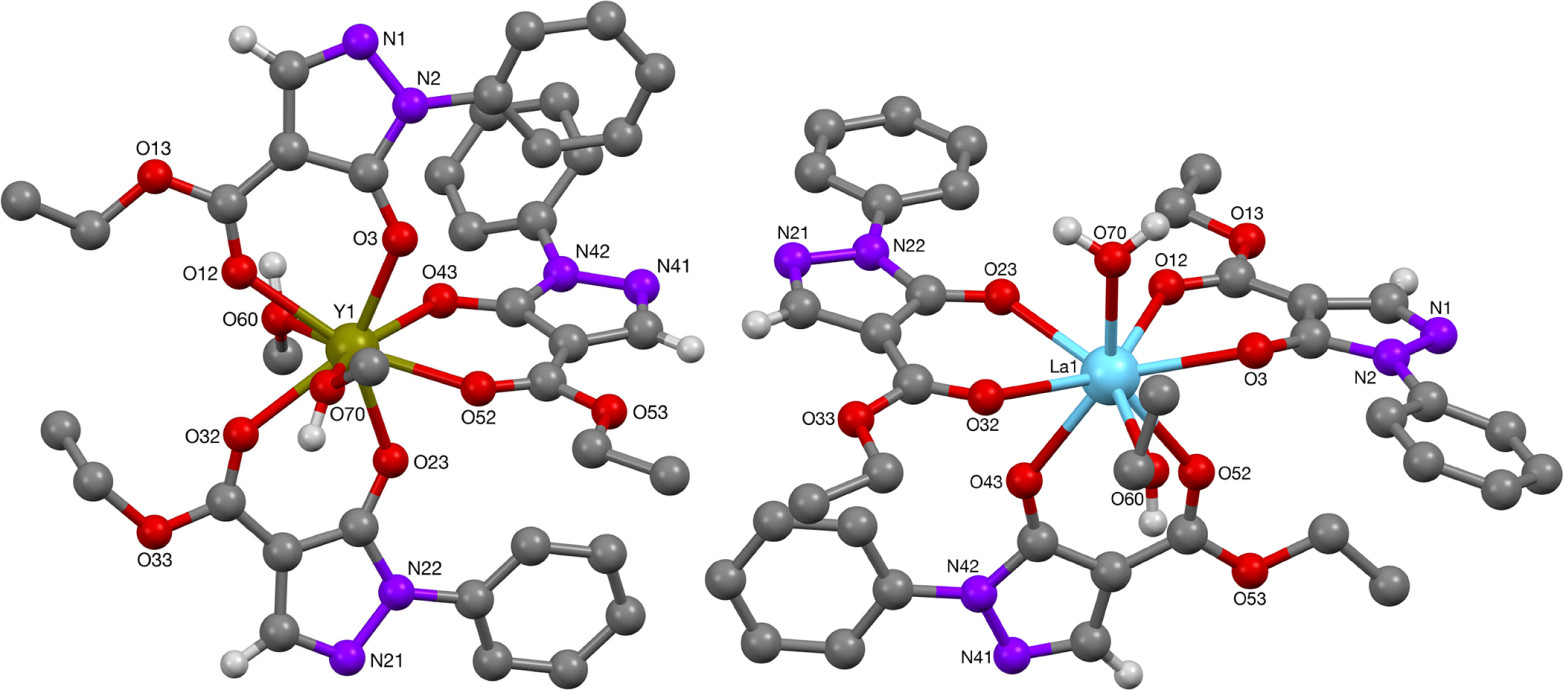
Molecular structures of Y- and La-complexes **28** and **62**, respectively.

The yttrium(III)-ion in complex **28** was found to be eight-coordinate, with three acylpyrazolonato ligands and two aquo ligands (Figure 17). Selected bond lengths and angles of complex **28** are reported in Table 11. These values compare well to literature reported data,^36^ with a slight elongation noted along the Y—O(=C) bond relative to literature data (2.4099(18) Å vs 2.282(2) Å/^36a^ 2.321(6) Å^36b^).

**Table 11:** Selected Bond Lengths and Angles of the Isomorphous Ytterbium(III)-, Lanthanum(III)-, Dysprosium(III)-, and Yttrium(III)-Phenyl-Pyrazolone Complexes

selected bond lengths [Å] and angles [°]	**28**	**62**	**66**	**70**
M–O^3^	2.2899(18)	2.4734(14)	2.253(3)	2.258(2)
M–O^12^	2.4026(18)	2.5234(15)	2.433(3)	2.370(2)
M–O^23^	2.29(52(18)	2.4117(14)	2.374(3)	2.258(2)
M–O^32^	2.3933(18)	2.5803(14)	2.392(3)	2.3601(19)
M–O^43^	2.2992(19)	2.4673(14)	2.331(3)	2.267(2)
M–O^52^	2.4340(19)	2.5437(15)	2.436(3)	2.418(2)
M–O^60^	2.3656(18)	2.5609(16)	2.390(3)	2.345(2)
M–O^70^	2.3761(19)	2.4910(14)	2.358(3)	2.356(2)
C–O^3^	1.268(3)	1.264(3)	1.288(5)	1.274(4)
C–O^12^	1.239(3)	1.238(2)	1.233(5)	1.246(4)
C–O^23^	1.273(3)	1.273(2)	1.259(5)	1.271(4)
C–O^32^	1.237(3)	1.234(2)	1.242(5)	1.242(4)
C–O^43^	1.277(3)	1.266(2)	1.257(5)	1.274(4)
C–O^52^	1.236(3)	1.229(3)	1.238(5)	1.238(4)
O^3^–M–O^12^	74.12(6)	71.73(5)	76.47(10)	74.97(7)
O^23^–M–O^32^	74.62(6)	73.23(5)	75.17(10)	75.56(7)
O^43^–M–O^52^	77.48(7)	69.94(5)	73.23(11)	78.37(8)
O^60^–M–O^70^	137.84(6)	89.93(5)	9785(10)	137.46(7)

The lanthanum(III)-phenyl pyrazolonato complex **62** is isostructural to yttrium complex **28**, while the solvent molecules L were H_2_O and EtOH (Figure 17). Selected bond lengths and angles are reported in Table 11. These values compare reasonably well to known lanthanum(III)-pyrazolonato complex with two additional water molecules of the general formula [La(Q^Ph^)_3_(H_2_O)_2_], in which La–O bond distances for the ligand were reported to be between 2.431 and 2.518 Å, while the aquo ligands were determined to be 2.530 and 2.617 Å.^13^ The values in complex **62** are, furthermore, in good agreement with a known formyl-substituted dimeric lanthanum(III)-pyrazolonato complex, which displayed similar La–O bond lengths between 2.436(3) and 2.650(4) Å and aquo ligands at a La–O distance of 2.560(3)–2.676(3) Å.^37^

**Figure 18:**
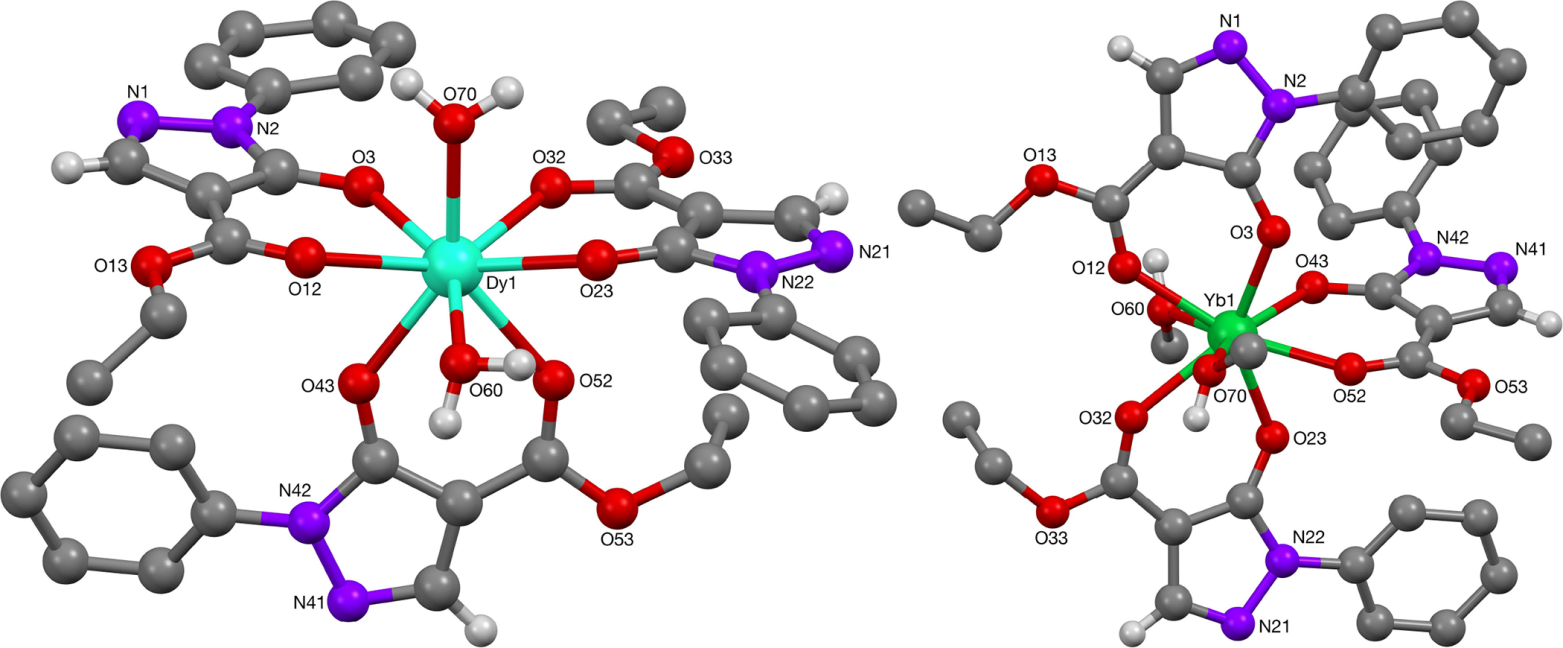
Molecular structures of Dy- and Yb-complexes **66** (L = H_2_O) and **70** (L = H_2_O), respectively.

X-Ray crystal structure determination of the dysprosium(III)-complex **66** revealed it to be isomorphous to the lanthanum(III) complex **62** (Figure 18). Consistent with lanthanide contraction, the Dy–O bond lengths were found to be generally shorter than the La–O bond lengths. In the solid state, the phenyl protected pyrazolonato dysprosium complex **66** gave an octacoordinated dysprosium(III) center, with two positions occupied by aquo ligands. Selected bond lengths and angles are reported in Table 11. Comparable Dy–O bond lengths (2.306(2)–2.380(2) Å) and angles (74.86(8)°–75.49(8)°) have been reported by Jia et al.,^38^ Pettinari et al. (Dy–O bond length between 2.271(3) and 2.410 Å),^39^ Li et al. (2.335(3)–2.422(3) Å),^40^ and Yu et al. (Dy–O bond length between 2.261(3)–2.492(3) Å).^41^

In the solid state, the phenyl-protected pyrazolonato ytterbium(III) complex **70** was isomorphous with complexes **28**, **62**, and **66** (Figure 18). The Yb–O bond lengths were found to be shorter than the La–O and Dy–O bond lengths, consistent with the lanthanide contraction. The ytterbium(III) center is octacoordinated with two additional aquo ligands completing the coordination sphere. Selected bond lengths and angles are reported in Table 11. Comparable Yb–O bond lengths (2.255(4)–2.360(5) Å) and angles (74.6(2)°–75.2(2)°) in ytterbium pyrazolonato complexes with solvent molecules (H_2_O or EtOH) have been reported by Bombierie et al.,^42^ and Yang and co-workers (2.257–2.370 Å),^29^ while Lü and co-workers reported structural data on a mixed ytterbium(III)-pyrazolonato complex with 1,10-phenanthroline (Yb–O bond lengths of: 2.252(7)–2.369(8) Å;O–Yb–O angles per ligand of: 71.9(3)°–73.6(3)°).^43^ Lü et al. further reported Yb–O bond lengths of similar values (2.261(5)–2.399(5) Å) in mixed Zn-salen Yb-tris pyrazolonato complexes with slightly smaller dihedral angles per pyrazolonato ligand (72.26(15)°–73.03(18)°).^43^

**Figure 19:**
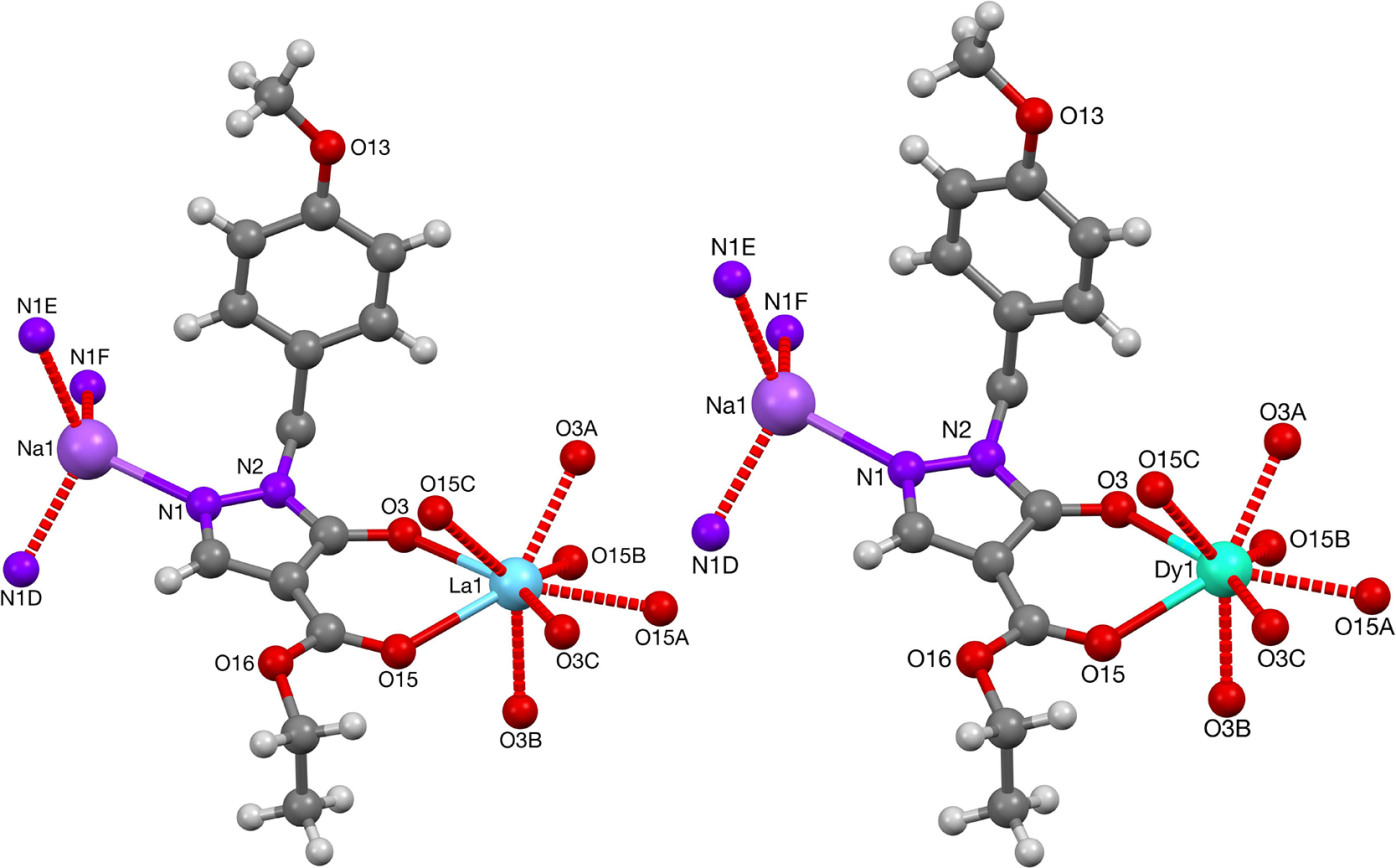
Crystal structures of the heterobimetallic sodium/lanthanide coordination polymers units **61** and **65**.

**Figure 20:**
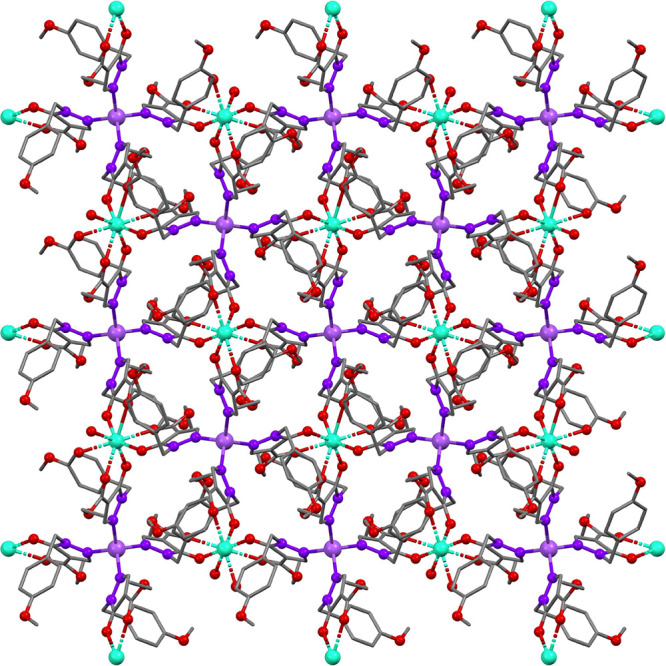
Illustration of the network of MOF-polymer **65** (color scheme: C, gray; O, red; N, dark purple; Dy, turquoise; Na, light purple).

The reactions with the PMB-protected pyrazolone **8** and lanthanum(III) or dysprosium(III) triflate gave crystals of the coordination polymers **61** and **65**, which were isomorphous. The lanthanide centers in both compounds are octa-coordinated, however with four acylpyrazolonato ligands, giving a formal negative charge, balanced with a sodium cation (Figure 19). The pyrazolonato ligands were determined to be symmetry equivalent, and the sodium cation is coordinated to the pyrazolone nitrogens. Selected bond lengths and angles are depicted in Table 12. To the best of our knowledge, a MOF-like complex of lanthanum or dysprosium with four acylpyrazolone ligands and a sodium cation has not been previously reported in literature. In the mixed lanthanum/sodium-polymer, per unit cell, two cavities were found in the MOF, with volumes of 133 Å^3^, which were found to partially host recrystallization solvent molecules. Similarly in the mixed dysprosium(III)/sodium(I)-polymer, per unit cell, two cavities were found in the MOF, with volumes of 184 Å^3^, which were found to partially host recrystallization solvent molecules, Figure 20.

**Table 12:** Selected Bond Lengths and Angles of Heterobimetallic Sodium/Lanthanide Coordination Polymers

selected bond lengths [Å] and angles [°]	**61**	**65**
M–O^3^/^3A^/^3B^/^3C^	2.401(12)/2.402(12)	2.289(7)
M–O^15^/^15A^/^15B^/^15C^	2.546(17)	2.455(6)
Na–N^1^/^1D^/^1E^/^1F^	2.357(19)	2.425(9)
C–O^3^	1.319(18)	1.275(11)
C–O^15^	1.28(3)	1.233(11)
O^3^–M–O^15^	74.4(6)	76.1(2)
N^1^–M–N^1D^	90.9(8)	89.1(5)
N^1^–M–N^1E^	119.5(5)	120.5(2)

Crystal structures of polymeric lanthanide acylpyrazolone complexes have been reported for samarium(III), europium(III), terbium(III), and dysprosium(III) with hydroxonium counter-cations.^44^ In addition, Shul’gin et al. reported the X-ray structure determination of a MOF-like acylpyrazolonato complex with europium(III), and sodium counter-cations.^45^ A related complex with a significantly larger I-*N*-dodecyl-*N*′,*N*′-dimethylamino-stilbazolium counterion has been reported, but this one has been determined to display discrete molecules in the solid state, which are not polymeric.^46^ In the octacoordinated lanthanum(III)-complex anion, La–O bond lengths of 2.454(4)–2.530(3) Å were determined, with O–La–O bond angles per pyrazolonato ligand of 69.16(9)°–70.52(9)°, values which are in good agreement with the ones determined for polymeric complex (**61**). X-Ray structures of related nonpolymeric lanthanide acylpyrazolone complexes with four ligand molecules and ammonium-derived counter-cations have been reported.^39,45,47^ Related ammonium complexes have been reported in studies of dysprosium(III) pyrazolonato complexes as constituents of Langmuir–Blodgett films and for other material applications.^47a,48^

**Figure 21:**
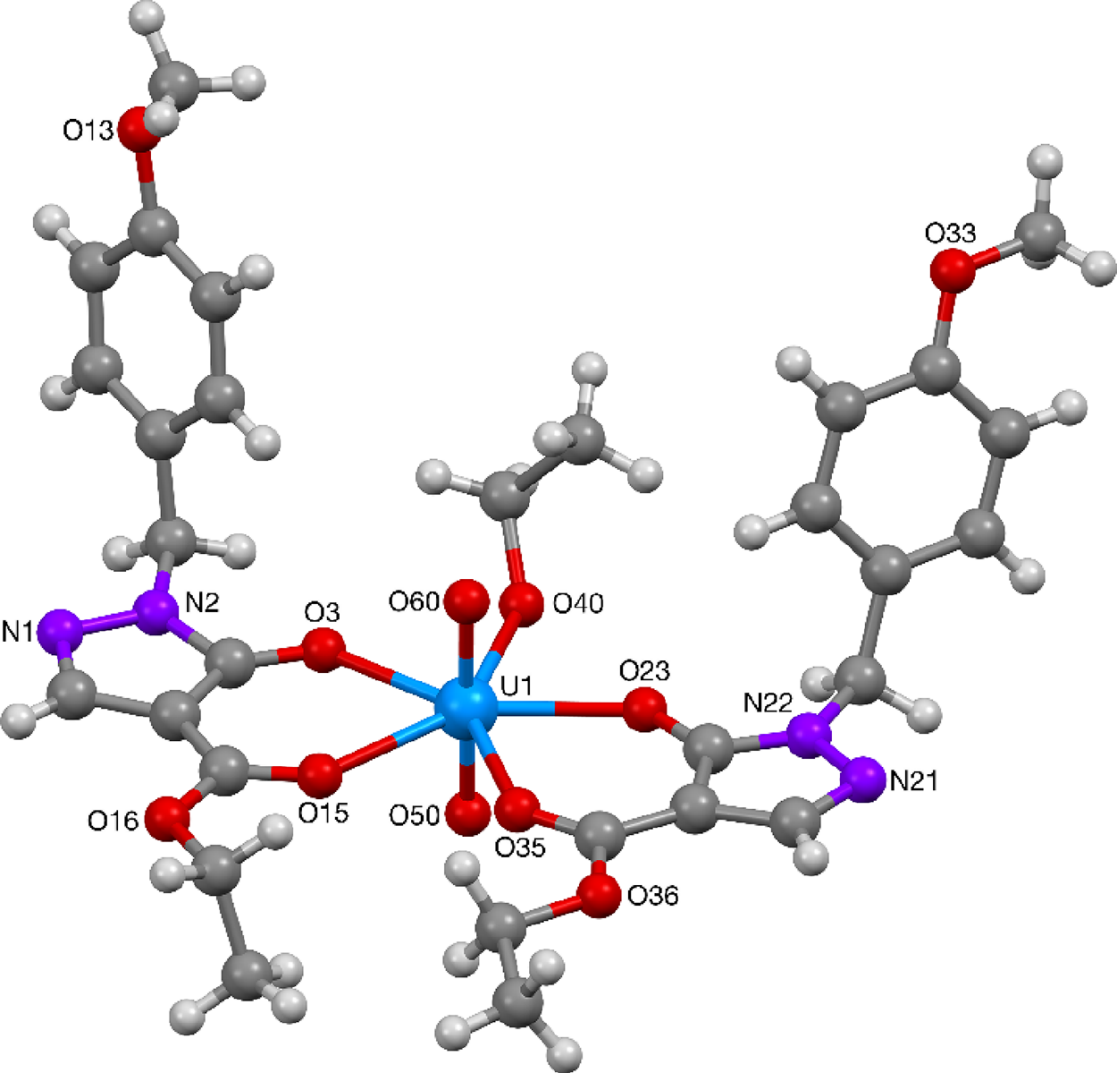
Molecular structure of UO_2_-complex **73**.

**Figure 22:**
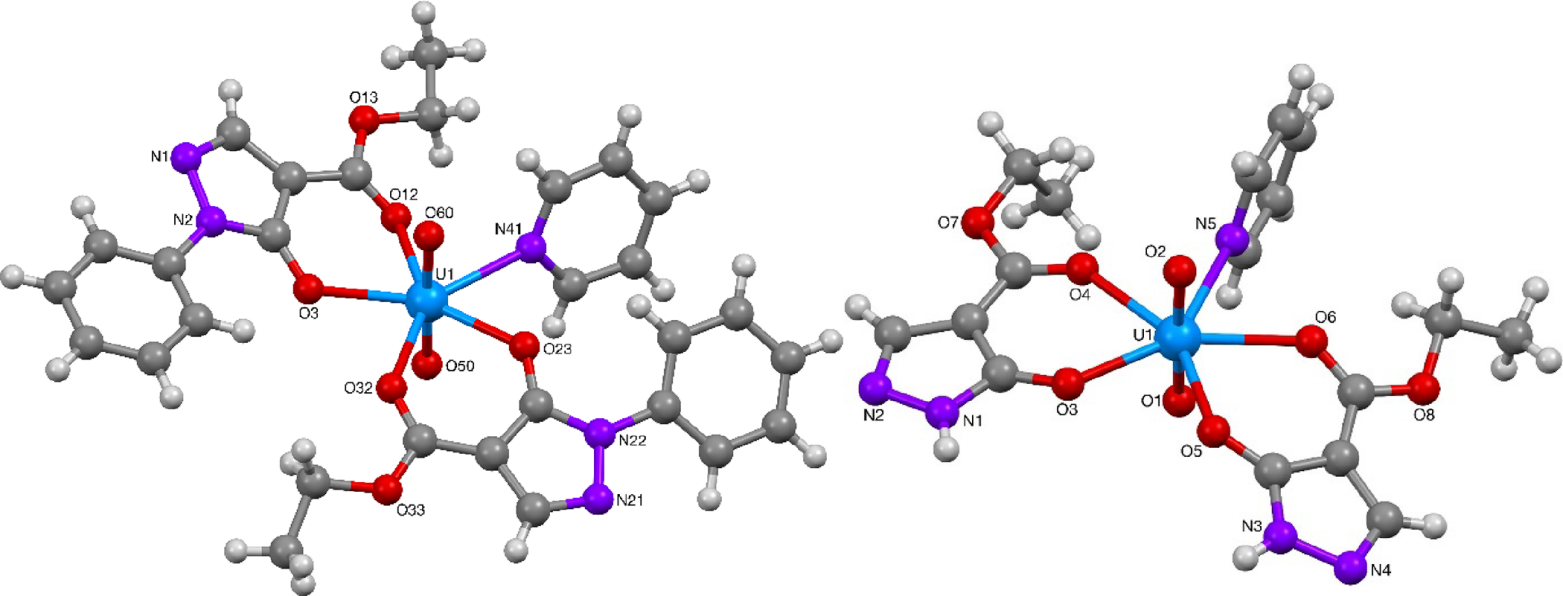
Molecular structures of UO_2_-complexes **74** and **76**.

**Figure 23:**
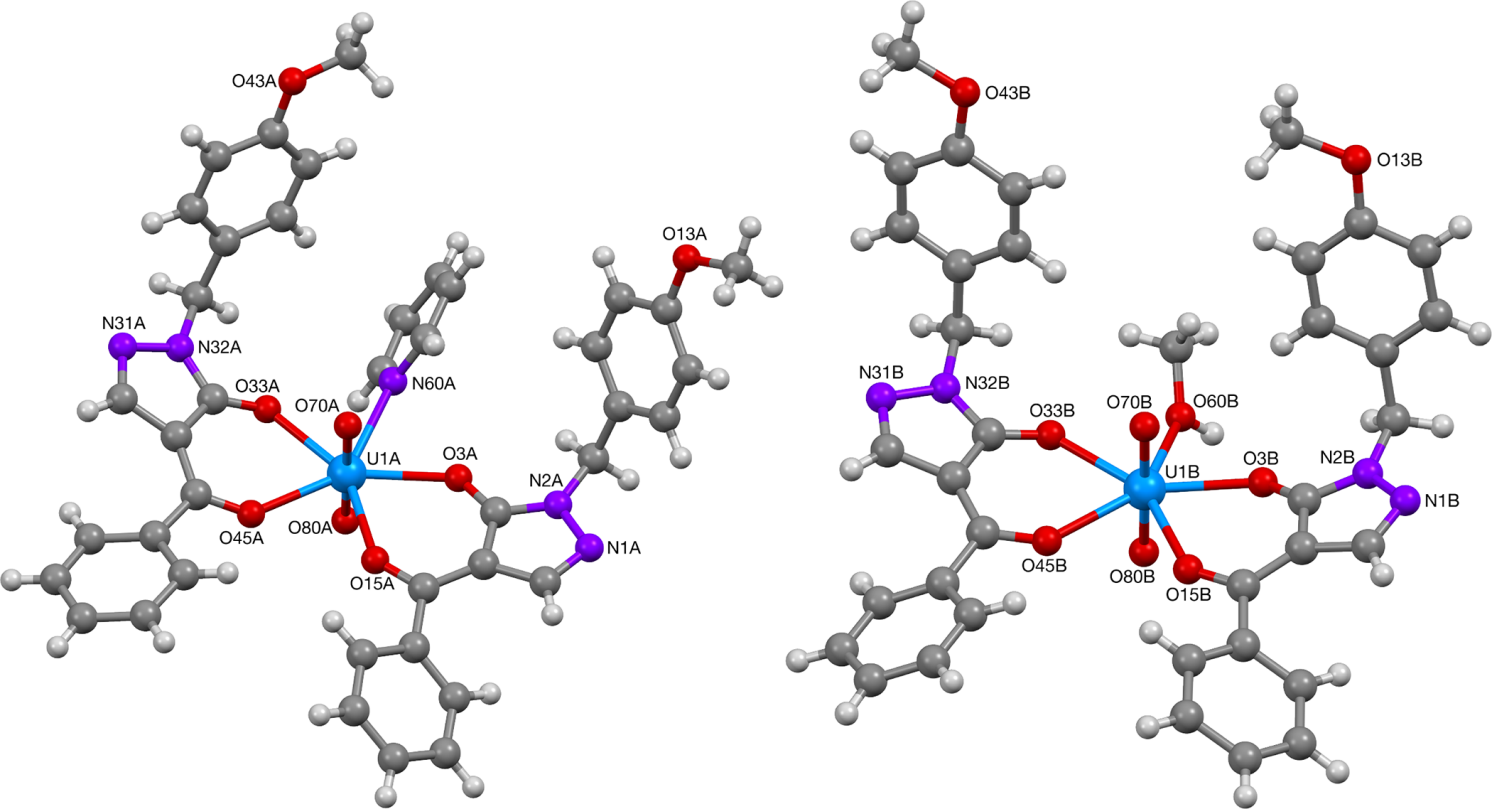
Molecular structures of UO_2_ complex **75** with pyridine (A) and methanol (B) as ligands.

The uranium(VI) ion in complexes **73** (Figure 21), **74** and **76** (Figure 22), and **75** (Figure 23) were found to contain a distorted octahedral core structure with two oxido ligands making up the uranyl(VI) ion, coordinated by two acylpyrazolonato ligands and an additional solvent ligand (methanol, ethanol or pyridine) resulting in the overall pentagonal-bipyramidal structure of the hepta-coordinated complexes. Complexes **73**, **75**, and **76** were found to contain a mirror symmetry element along the UO_2_–solvent ligand plane, while complex **74** was found to lack this symmetry element with the two acylpyrazolonato ligands being in *trans*-relationship to each other. Selected bond lengths and angles for complexes **73**–**76** are summarized in Tables 13 and 14. These values compare well to literature reported data for related uranyl acylpyrazolonato complexes.^49^

**Table 13:** Selected Bond Lengths and Angles of Uranyl Acylpyrazolonato Complexes **73**, **74**, and **76**

selected bond lengths [Å] and angles [°]	**73**	selected bond lengths [Å] and angles [°]	**74**	selected bond lengths [Å] and angles [°]	**76**
M–O^50/60^	1.765(16)/1.785(17)	M–O^50/60^	1.731(10)/1.736(11)	M–O^1/2^	1.773(3)/1.763(4)
M–O^3/23^	2.321(13)/2.361(13)	M–O^3/23^	2.335(7)/2.297(9)	M–O^3/5^	2.297(3)/2.359(3)
M–O^15/35^	2.454(13)/2.458(13)	M–O^12/32^	2.430(10)/2.452(8)	M–O^4/6^	2.411(3)/2.424(3)
M–O^40^	2.368(15)	M-N^41^	2.551(11)	M–N^5^	2.557(4)
C–O^3/23^	1.27(2)/1.29(3)	C–O^3/23^	1.279(13)/1.268(15)	C–O^3/5^	1.290(6)/1.297(6)
C–O^15/35^	1.26(2)/1.27(2)	C–O^12/32^	[1.19(3) + 1.36(3)]/1.244(15)	C–O^4/6^	1.239(6)/1.238(6)
O^3^–M–O^15^/O^23^–M–O^35^	72.9(5)/73.8(5)	O^3^–M–O^12^/O^23^–M–O^32^	72.5(3)/73.2(3)	O^3^–M–O^4^/O^5^–M–O^6^	73.62(12)/74.03(11)
O^50^–M–O^60^	179.2(9)	O^50^–M–O^60^	178.0(4)	O^1^–M–O^2^	176.14(13)
O^50^–M–O^40^/O^60^–M–O^40^	89.5(6)/90.6(7)	O^50^–M–N^41^/O^60^–M–N^41^	89.4(4)/88.6(4)	O^1^–M–N^5^/O^2^–M–N^5^	88.81(14)/87.36(15)

**Table 14:** Selected Bond Lengths and Angles of Uranyl Acylpyrazolonato Complex **75**

selected bond lengths [Å] and angles [°]	**75-A**	selected bond lengths [Å] and angles [°]	**75-B**
M–O^70A/80A^	1.750(8)/1.763(8)	M–O^70B/80B^	1.758(7)/1.768(7)
M–O^3A/33A^	2.388(7)/2.358(6)	M–O^3B/33B^	2.358(8)/2.347(7)
M–O^15A/45A^	2.367(6)/2.369(7)	M–O^15B/45B^	2.393(7)/2.416(9)
M–N^60A^	2.568(8)	M-O^60B^	2.391(5)
C–O^3A/33A^	1.283(11)/1.289(12)	C–O^3B/33B^	1.266(12)/1.289(13)
C–O^15A/45A^	1.269(11)/1.271(11)	C–O^15B/45B^	1.269(12)/1.284(12)
O^3A^–M–O^15A^/O^33A^–M–O^45A^	73.3(2)/73.1(2)	O^3B^–M–O^15B^/O^33B^–M–O^45B^	72.2(3)/72.6(3)
O^70A^–M–O^80A^	179.1(3)	O^70B^–M–O^80B^	179.2(4)
O^70A^–M–N^60A^/O^80A^–M–N^60A^	88.0(3)/91.3(3)	O^70B^–M–O^60B^/O^80B^–M–O^60B^	95.8(3)/84.7(3)

## Conclusions

In conclusion, structurally diverse pyrazolone-metal complexes were synthesized from readily accessible pyrazolone precursors. The complexes contained the main group and transition metals, lanthanides, and actinides. Structural characterization was performed by NMR and IR spectroscopy, X-ray crystal structure determinations, mass-spectrometry, and DSC measurements. Multiple examples were found to be of a polymeric structure in the solid state—a feature which was observed on complexes of all four ligands. Future investigations are directed toward the material properties of the MOF-like polymers and the extension toward other metal ions of interest.

## Supporting Information Available

Spectral data, DSC and TG/DTA measurements, computational calculations, and X-ray crystallography data (PDF)
Detailed experimental procedure, compound characterization, and NMR-spectra of all synthesized compounds as well additional X-Ray crystallography data Raw computational and crystallographic FAIR data are available from the Spiral data repository via ref (50). (PDF)
