# Supplementary material for: Syntheses and Characterization of Main Group, Transition Metal, Lanthanide, and Actinide Complexes of Bidentate Acylpyrazolone Ligands
Source: Inorg Chem. 2023 Aug 7;62(33):13253–76. doi: 10.1021/acs.inorgchem.3c01506 (PMC10445273; doi:10.1021/acs.inorgchem.3c01506)
Supplement: Supplementary file 1 — ic3c01506_si_001.pdf [file ic3c01506_si_001.pdf]

Syntheses and Characterization of Main Group, Transition Metal, Lanthanide and Actinide Complexes of Bidentate Acylpyrazolone Ligands

Supporting Information

Thomas Mies,[1] Andrew J. P. White,[1] Henry S. Rzepa,[1] Luciano Barluzzi,[2] Mohit Devgan,[1] Richard A. Layfield,[2] and Anthony G. M. Barrett[1]*

[1] Department of Chemistry, Imperial College, Molecular Sciences Research Hub, White City Campus, Wood Lane, London W12 0BZ, England.

[2] Department of Chemistry, University of Sussex, Falmer, Brighton, BN1 9QR, England.

Table of Contents

I. **Spectral Data**
II. **DSC and TG/DTA Measurements**
III. **Computational Calculations**
IV. **X-Ray Crystallography Data**

I. Spectral Data

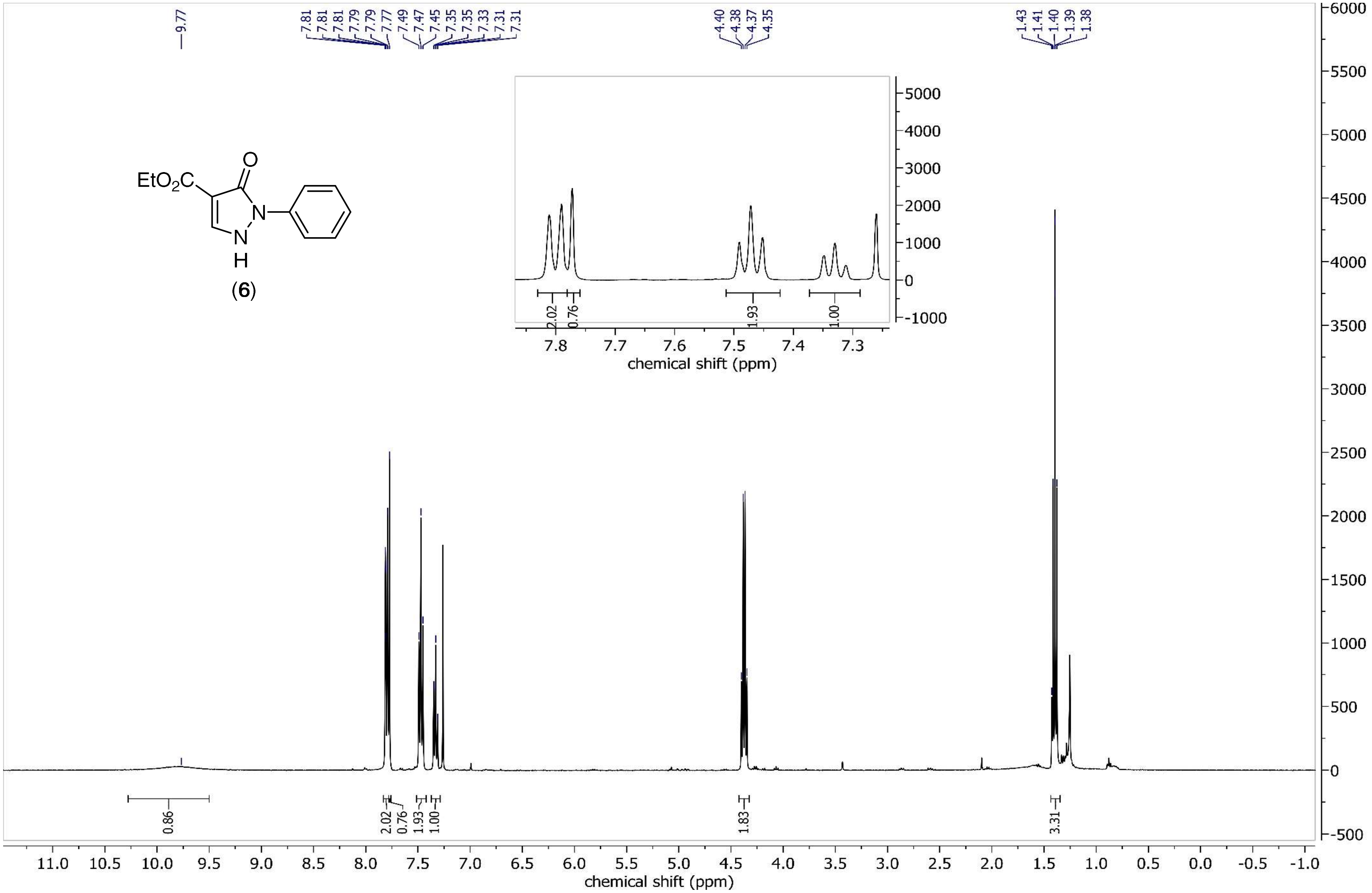

Figure S1: ^{1}H-NMR spectrum of ethyl 3-oxo-2-phenyl-2,3-dihydro-1*H*-pyrazole-4-carboxylate (**6**) in $CDCl_3$.

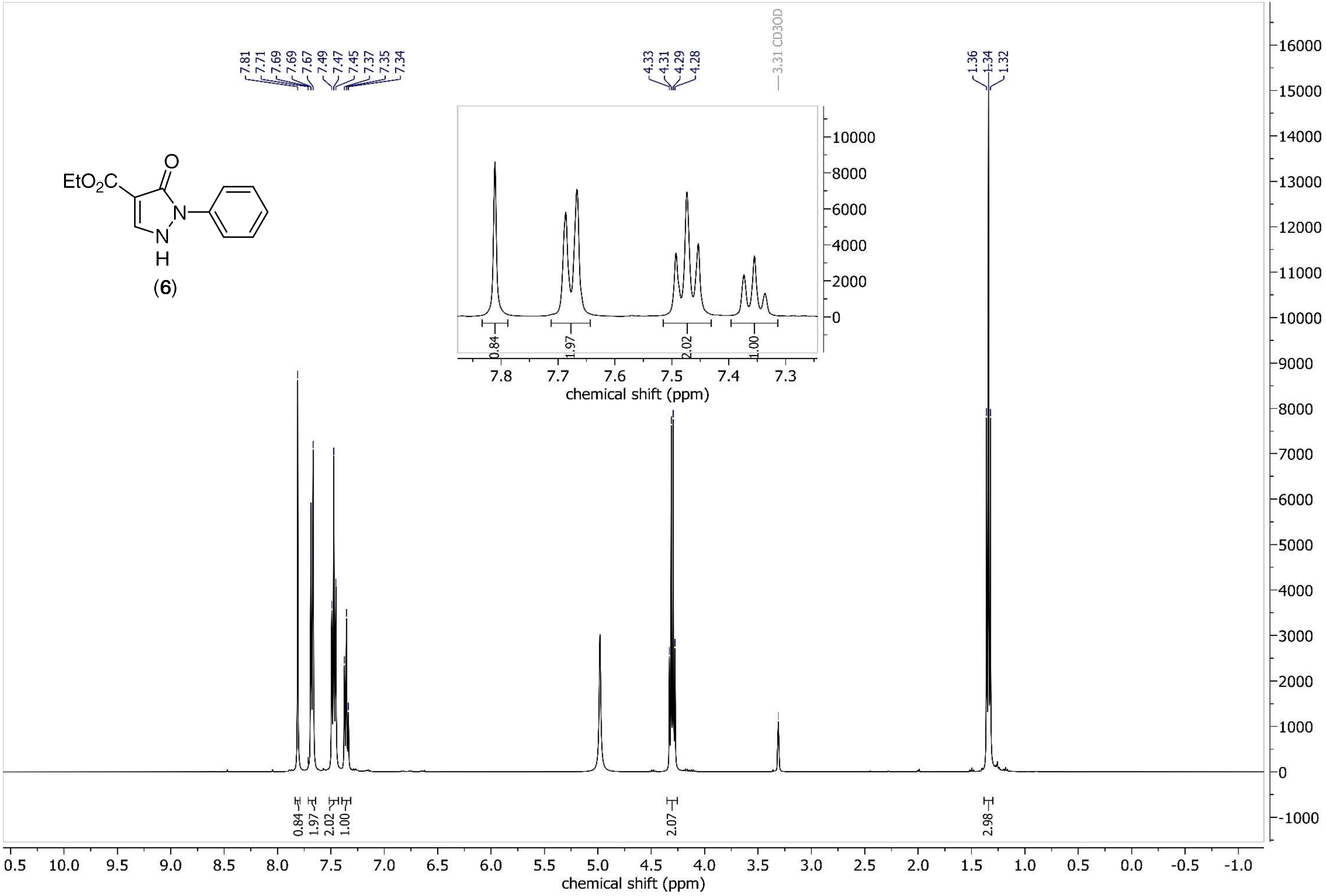

Figure S2: : ^{1}H-NMR spectrum of ethyl 3-oxo-2-phenyl-2,3-dihydro-1*H*-pyrazole-4-carboxylate (**6**) in CD_3OD.

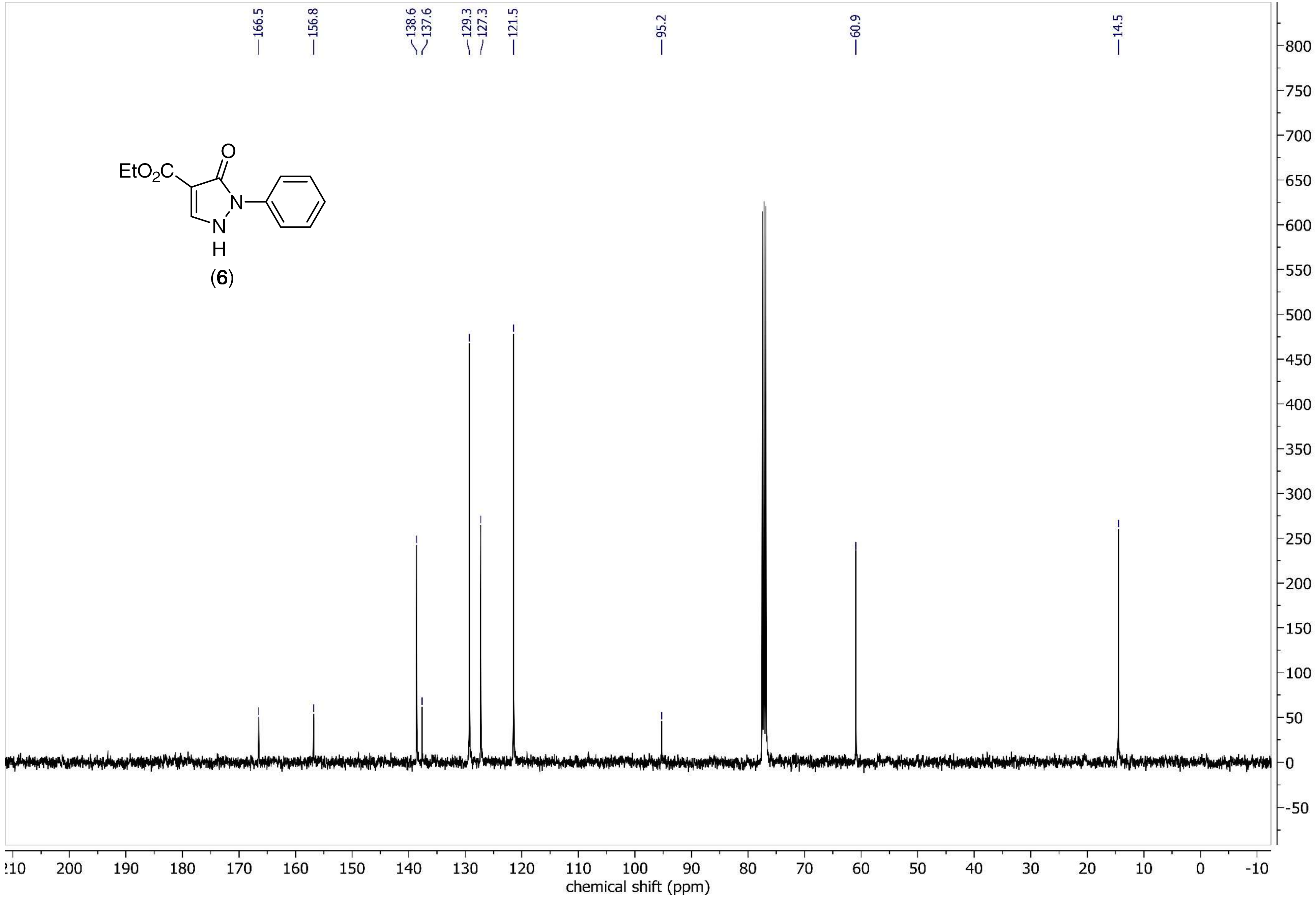

Figure S3: $^{13}C\{^{1}H\}$-NMR spectrum of ethyl 3-oxo-2-phenyl-2,3-dihydro-1*H*-pyrazole-4-carboxylate (**6**) in $CDCl_3$.

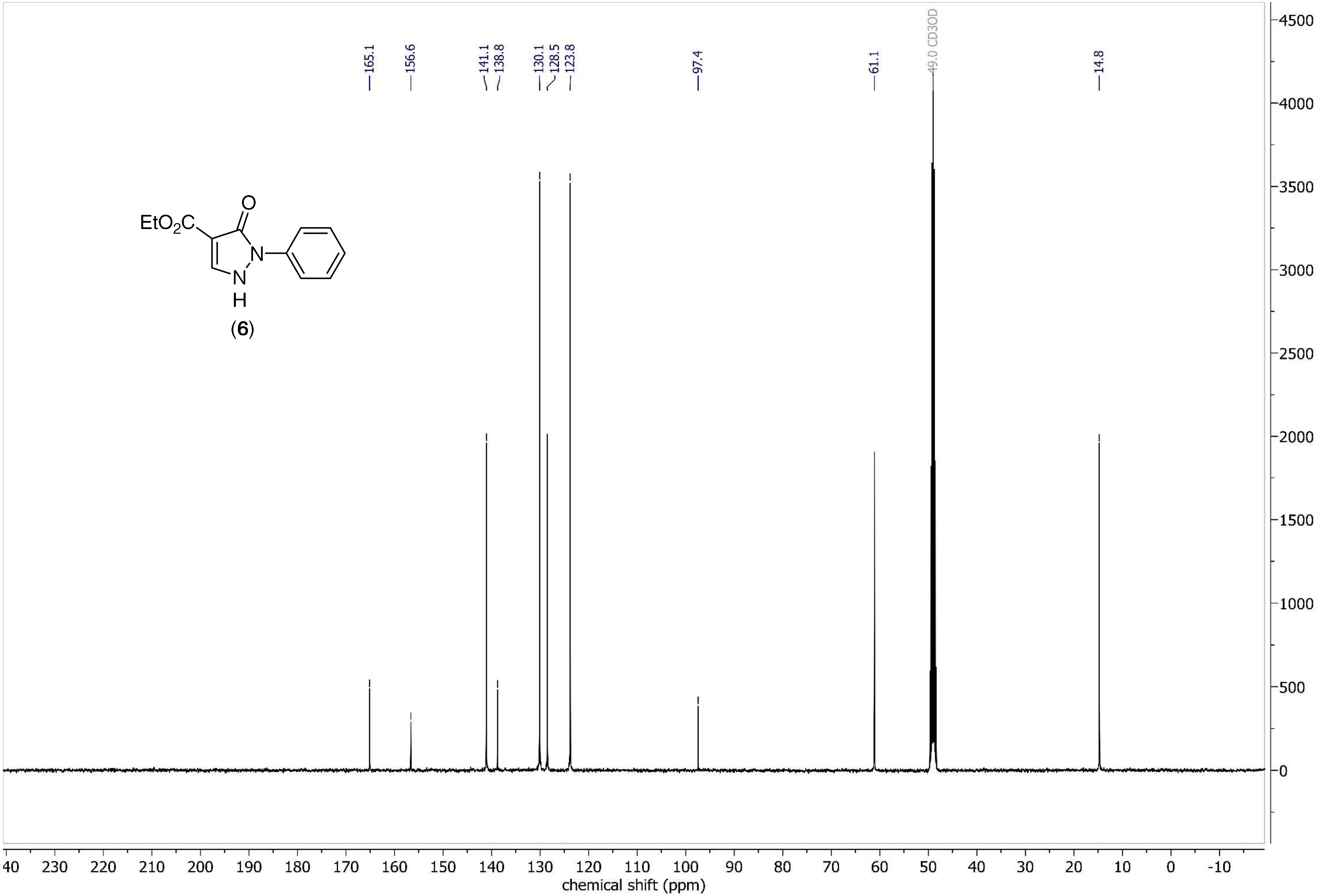

Figure S4: ${}^{13}C\{{}^{1}H\}$-NMR spectrum of ethyl 3-oxo-2-phenyl-2,3-dihydro-1*H*-pyrazole-4-carboxylate (**6**) in CD_3OD.

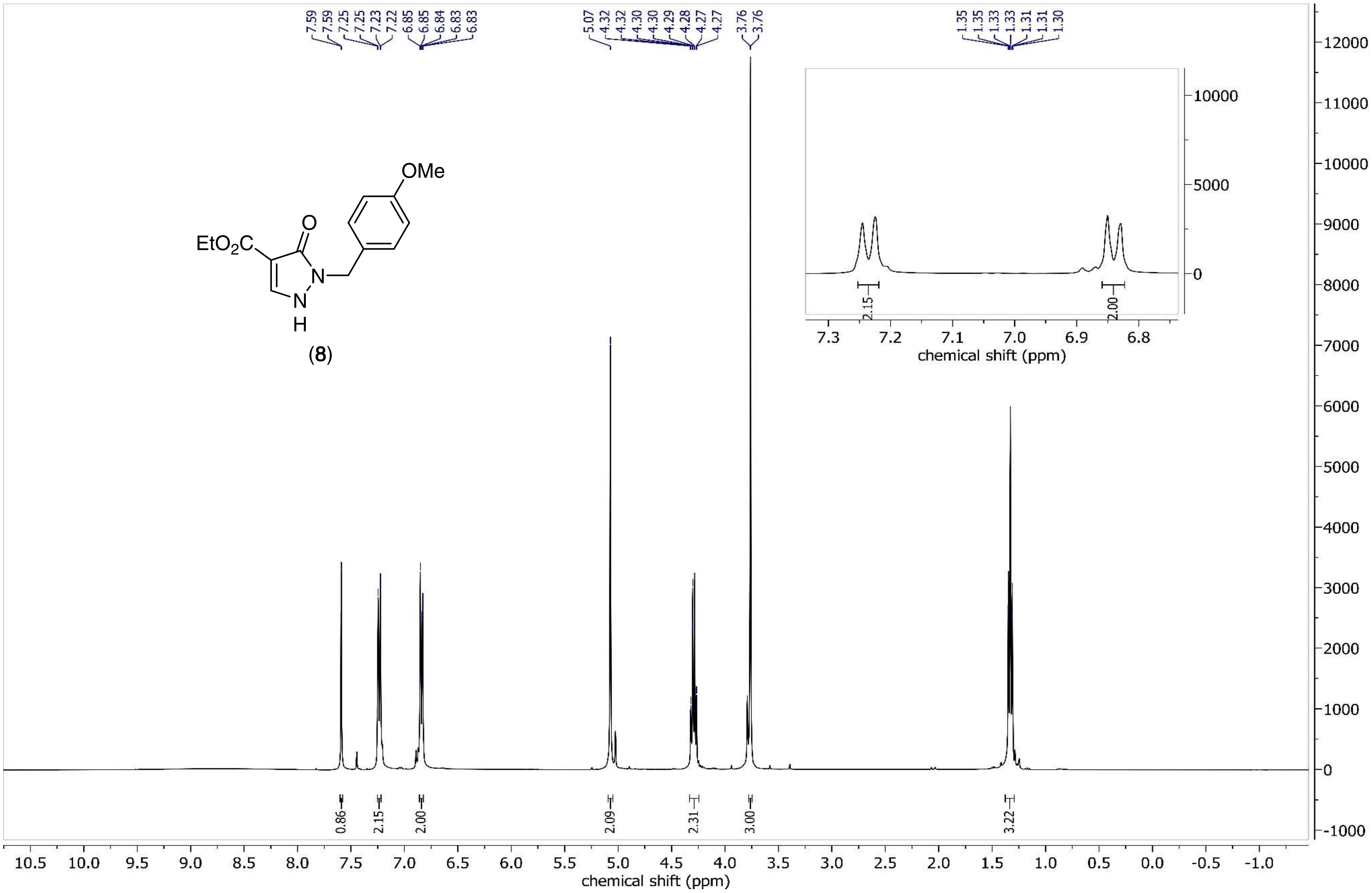

Figure S5: ^{1}H-NMR spectrum of ethyl 2-(4-methoxybenzyl)-3-oxo-2,3-dihydro-1*H*-pyrazole-4-carboxylate (**8**) in $CDCl_3$.

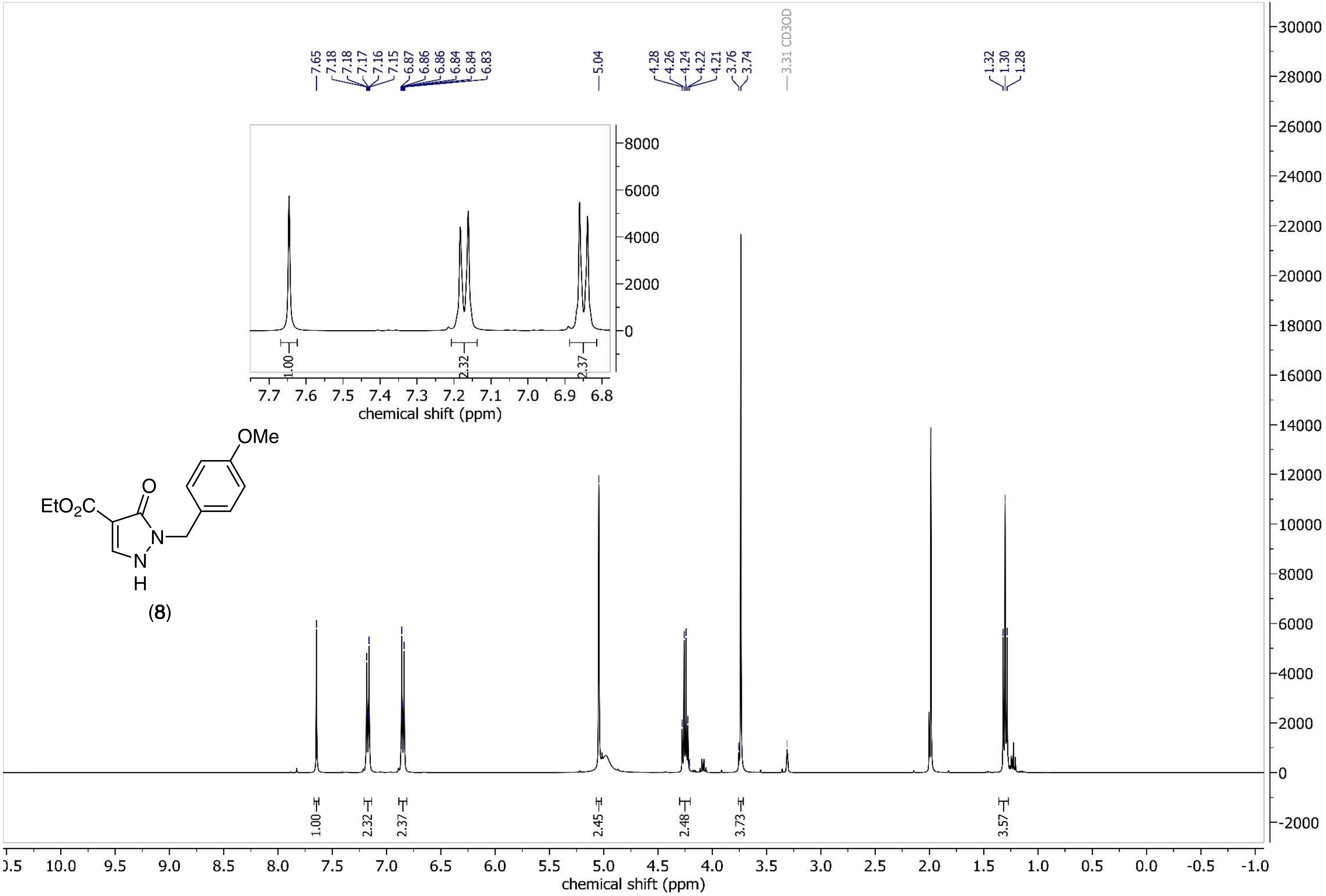

(**8**)

Figure S6: ^{1}H-NMR spectrum of ethyl 2-(4-methoxybenzyl)-3-oxo-2,3-dihydro-1*H*-pyrazole-4-carboxylate (**8**) in CD_3OD.

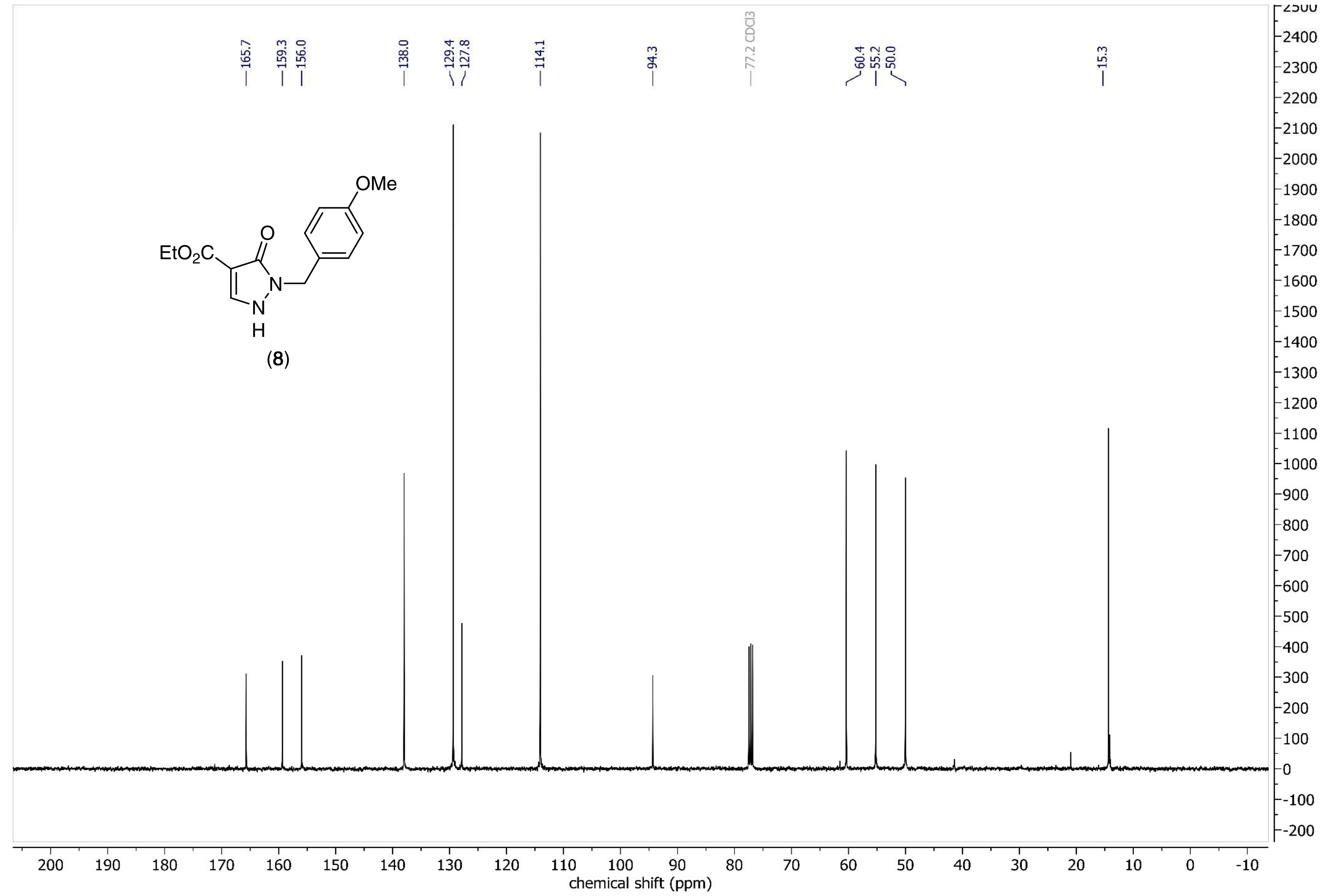

Figure S7: $^{13}C\{^{1}H\}$-NMR spectrum of ethyl 2-(4-methoxybenzyl)-3-oxo-2,3-dihydro-1H-pyrazole-4-carboxylate (**8**) in $CDCl_3$.

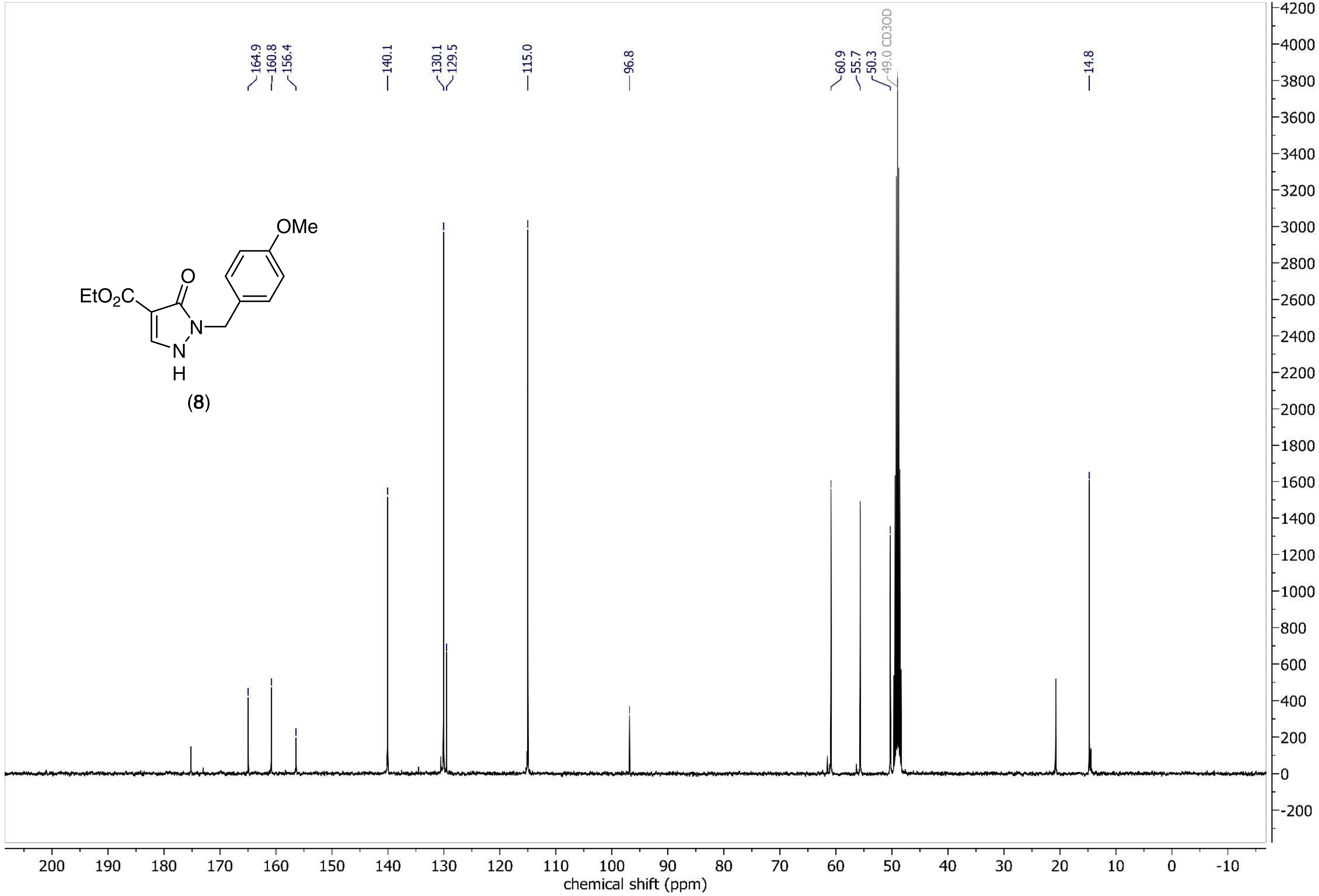

Figure S8: $^{13}C\{^1H\}$-NMR spectrum of ethyl 2-(4-methoxybenzyl)-3-oxo-2,3-dihydro-1H-pyrazole-4-carboxylate (**8**) in CD_3OD.

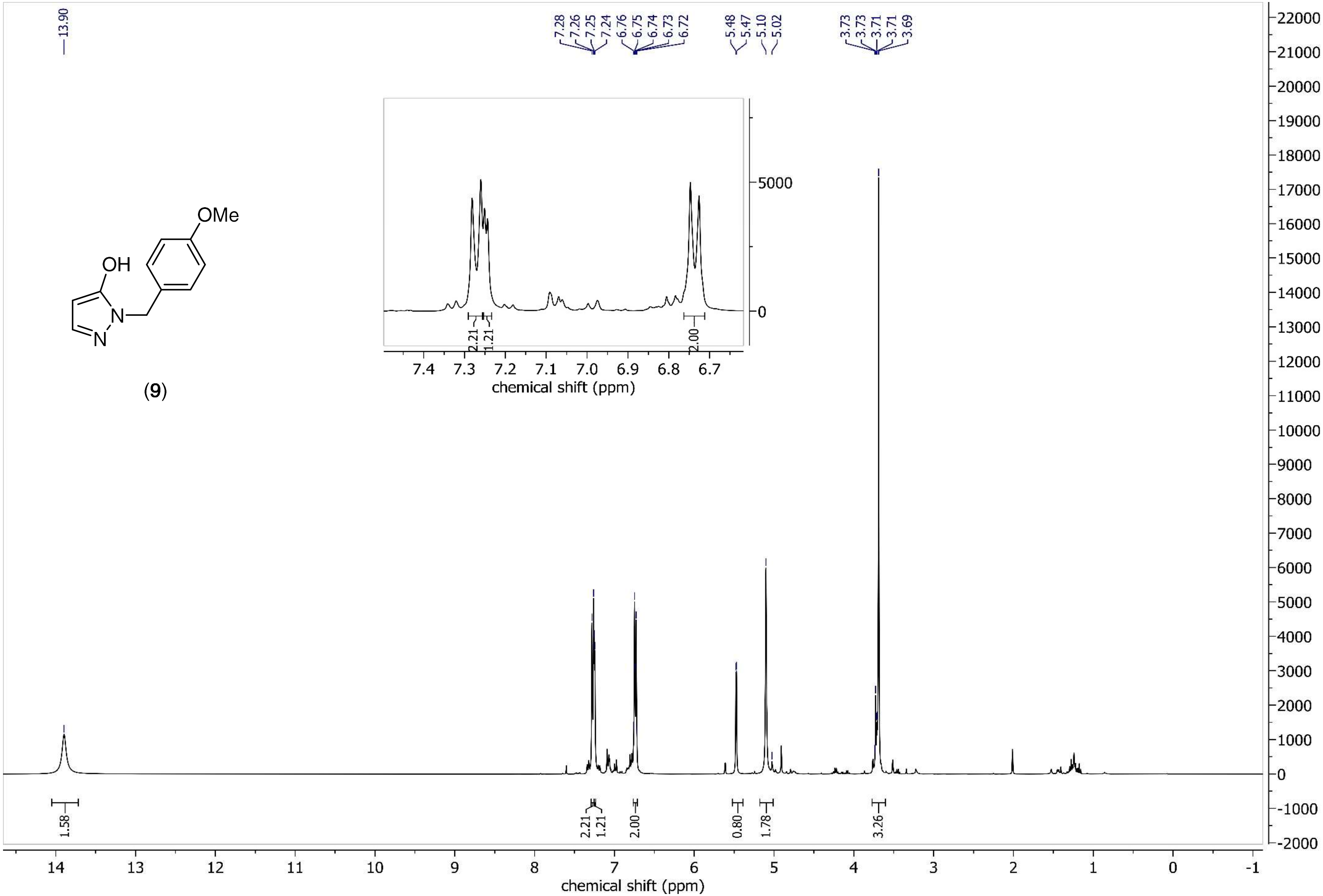

Figure S9: ^{1}H-NMR spectrum of 2-(4-methoxybenzyl)-2,4-dihydro-3*H*-pyrazol-3-one (**9**) majorly as the enol form in a keto-enol mixture.

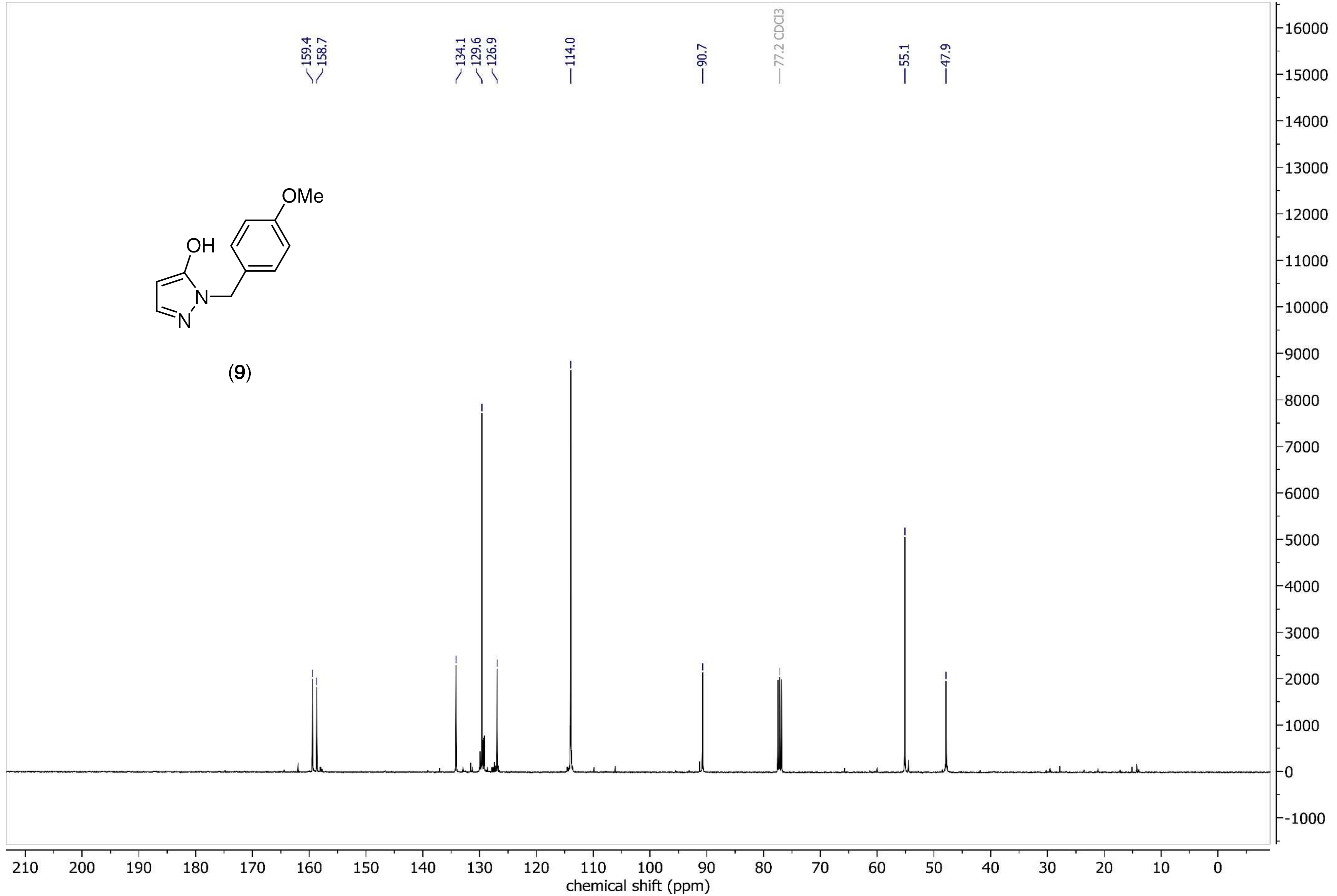

Figure S10: $^{13}C\{^{1}H\}$-NMR spectrum of 2-(4-methoxybenzyl)-2,4-dihydro-3*H*-pyrazol-3-one (**9**) majorly as the enol form in a keto-enol mixture.

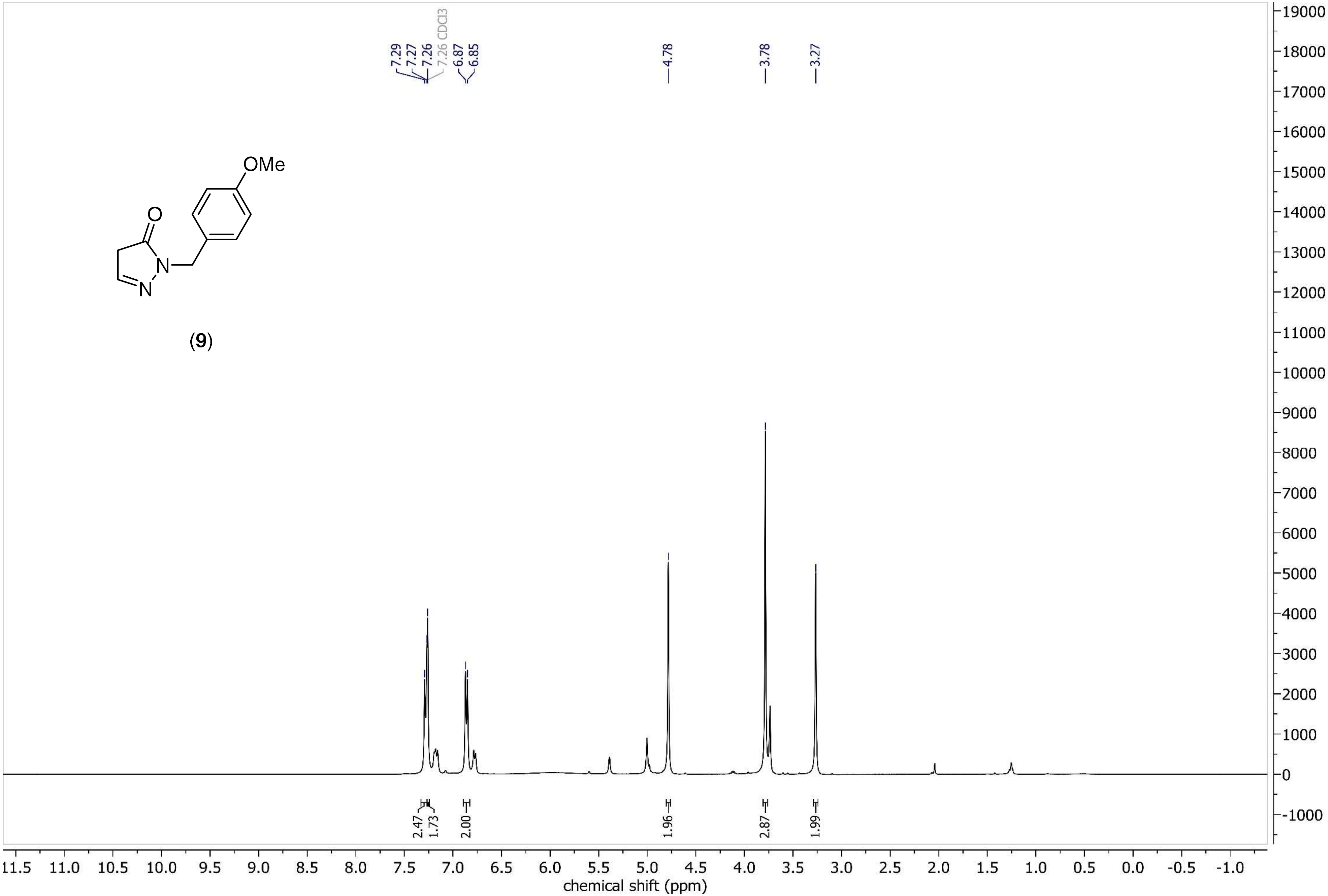

Figure S11: ^{1}H-NMR spectrum of 2-(4-methoxybenzyl)-2,4-dihydro-3*H*-pyrazol-3-one (**9**) majorly as the keto form in a keto-enol mixture.

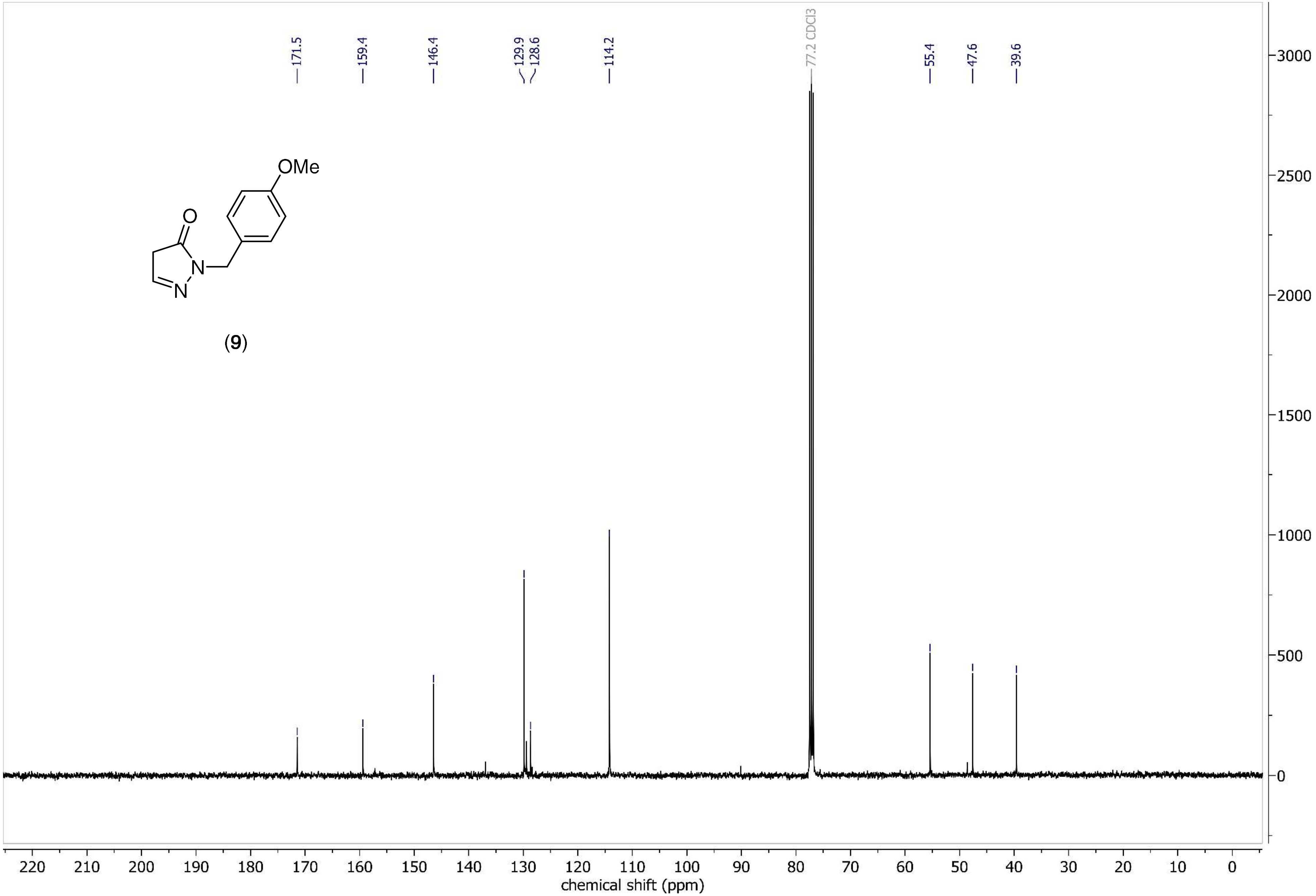

Figure S12: $^{13}C\{^1H\}$-NMR spectrum of 2-(4-methoxybenzyl)-2,4-dihydro-3*H*-pyrazol-3-one (**9**) majorly as the keto form in a keto-enol mixture.

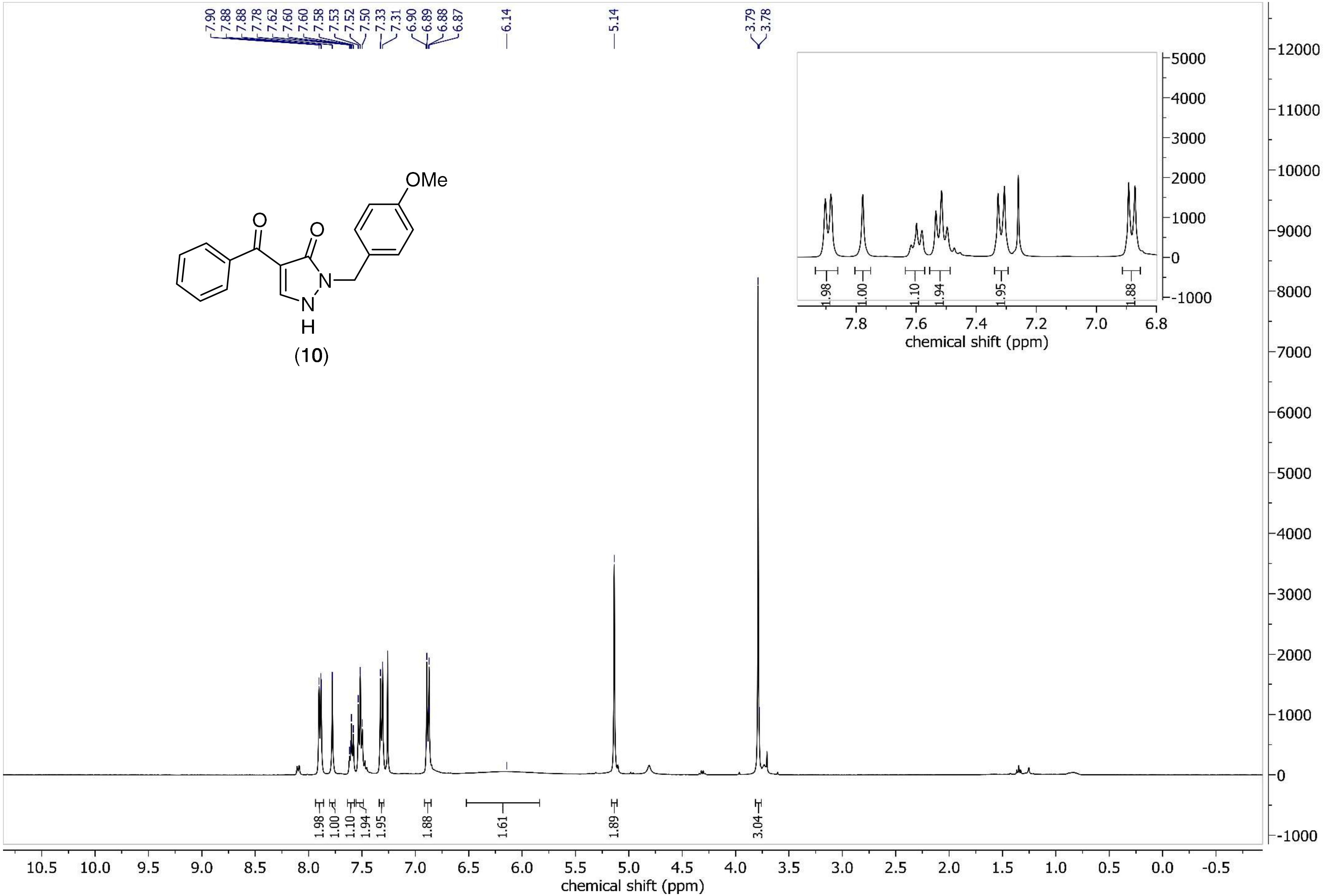

Figure S13: ^{1}H-NMR spectrum of 4-benzoyl-2-(4-methoxybenzyl)-1,2-dihydro-3*H*-pyrazol-3-one (**10**) in $CDCl_3$.

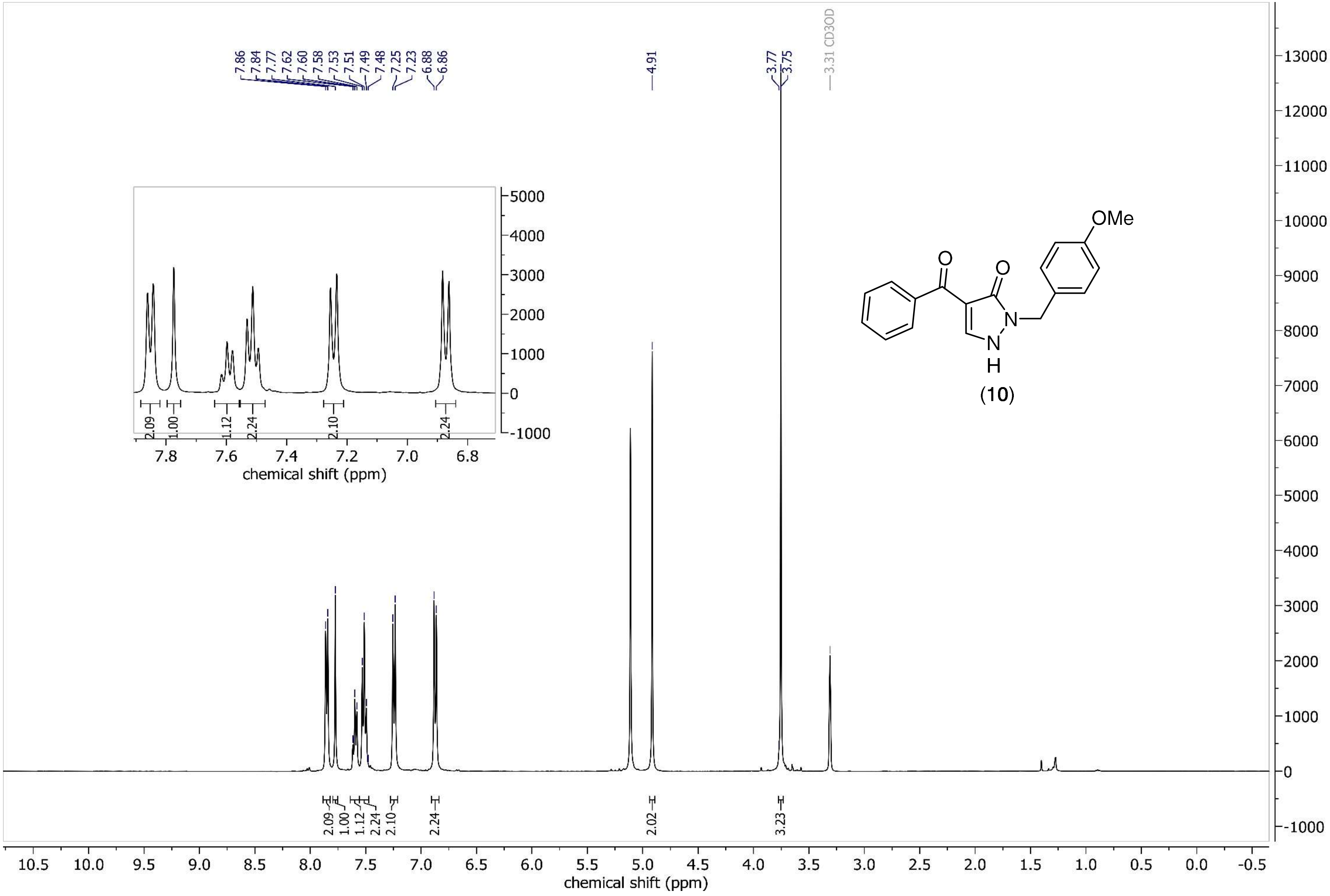

Figure S14: ^{1}H-NMR spectrum of 4-benzoyl-2-(4-methoxybenzyl)-1,2-dihydro-3*H*-pyrazol-3-one (**10**) in CD_3OD.

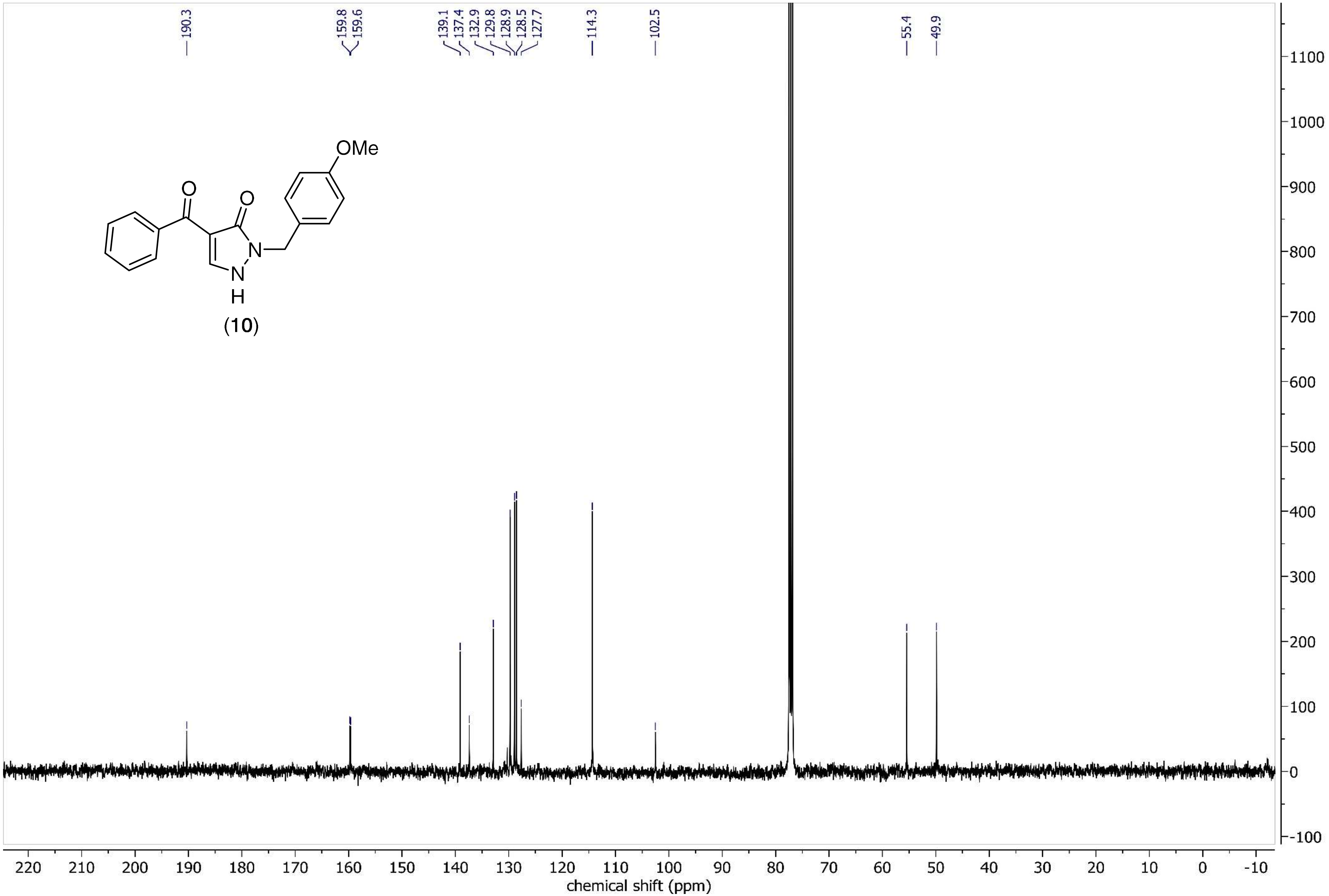

Figure S15: $^{13}C\{^{1}H\}$-NMR spectrum of 4-benzoyl-2-(4-methoxybenzyl)-1,2-dihydro-3*H*-pyrazol-3-one (**10**) in $CDCl_3$.

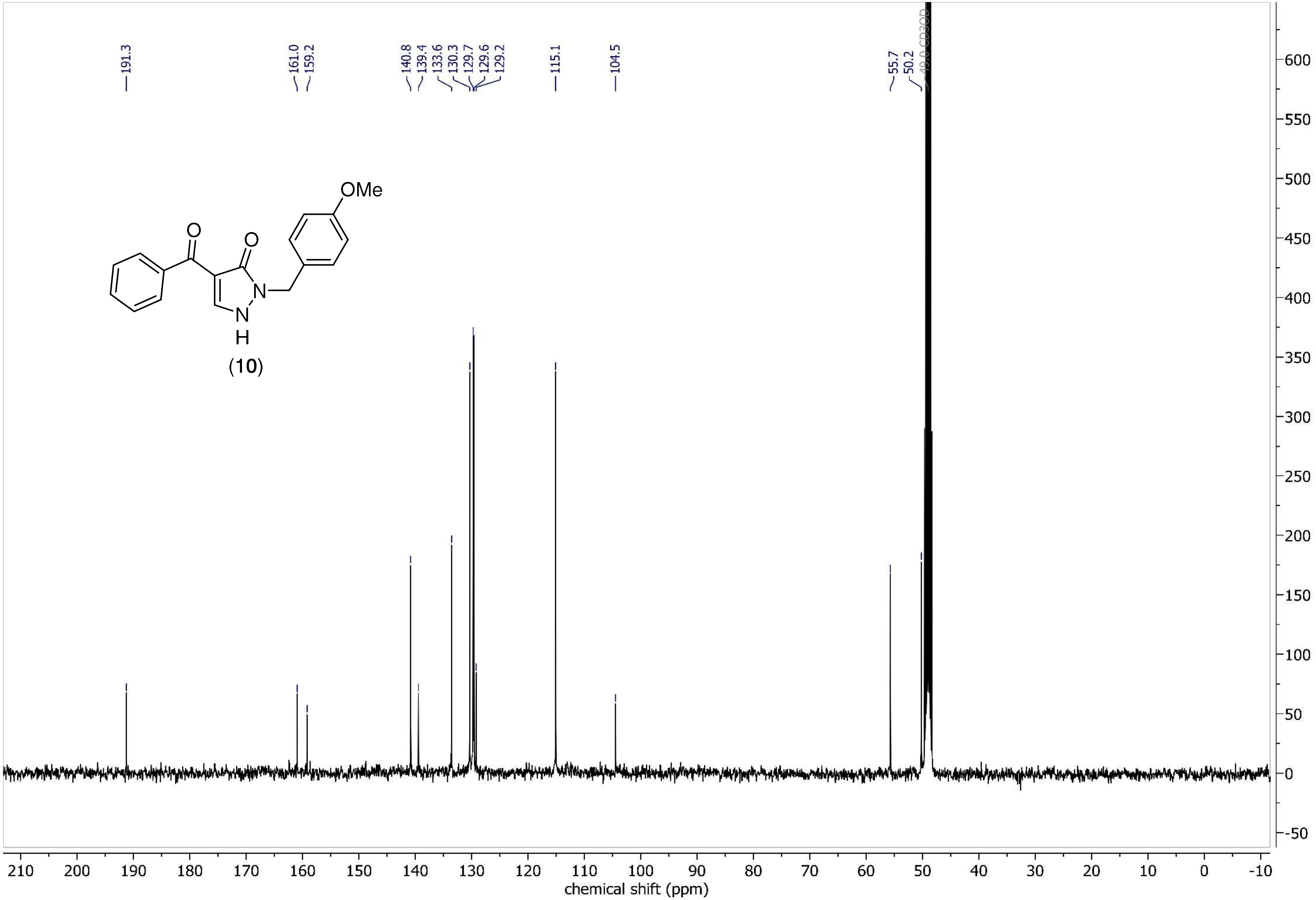

Figure S16: $^{13}C\{^{1}H\}$-NMR spectrum of 4-benzoyl-2-(4-methoxybenzyl)-1,2-dihydro-3*H*-pyrazol-3-one (**10**) in CD_3OD.

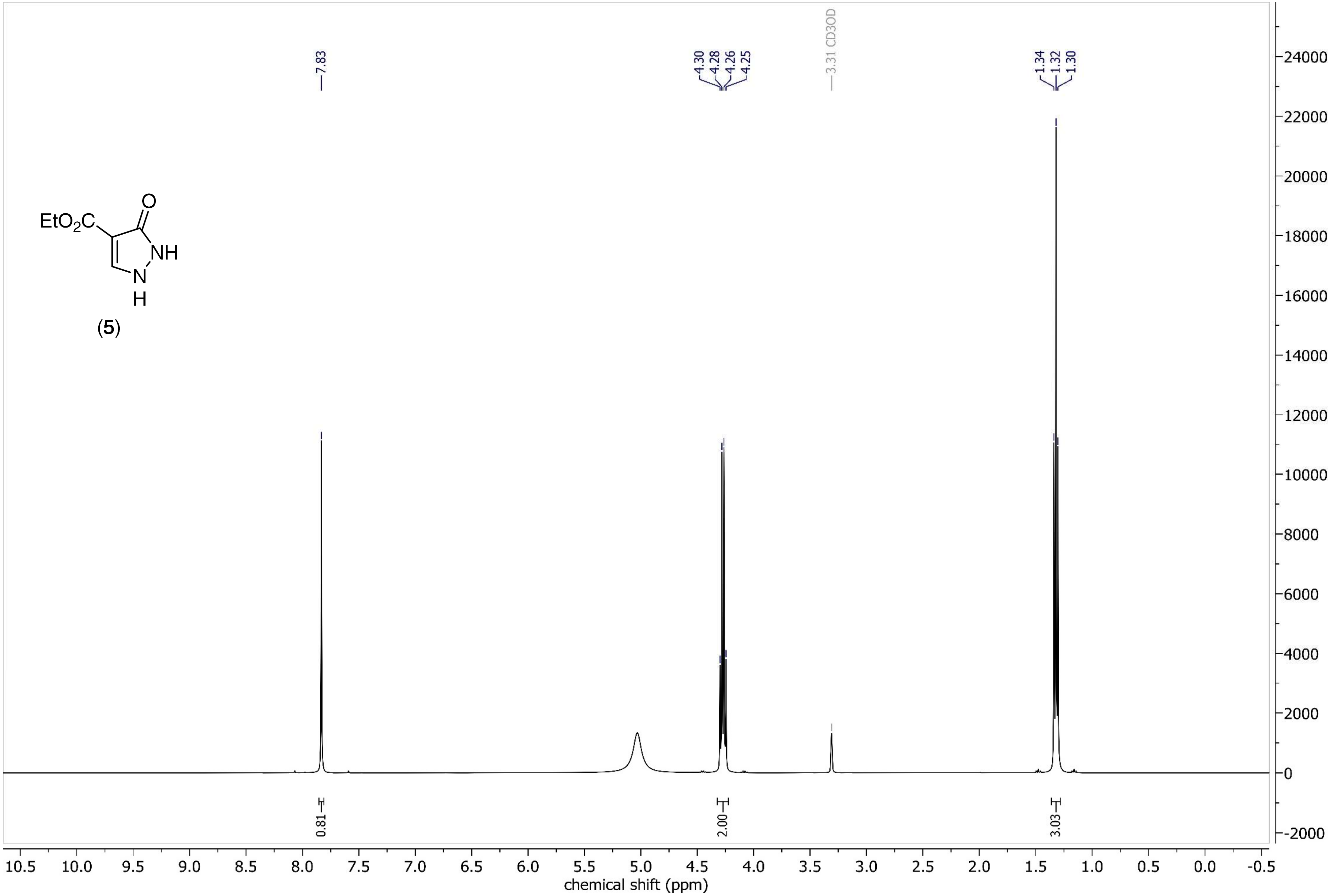

Figure S17: ^{1}H-NMR spectrum of ethyl 3-oxo-2,3-dihydro-1*H*-pyrazole-4-carboxylate (**5**) in CD_3OD.

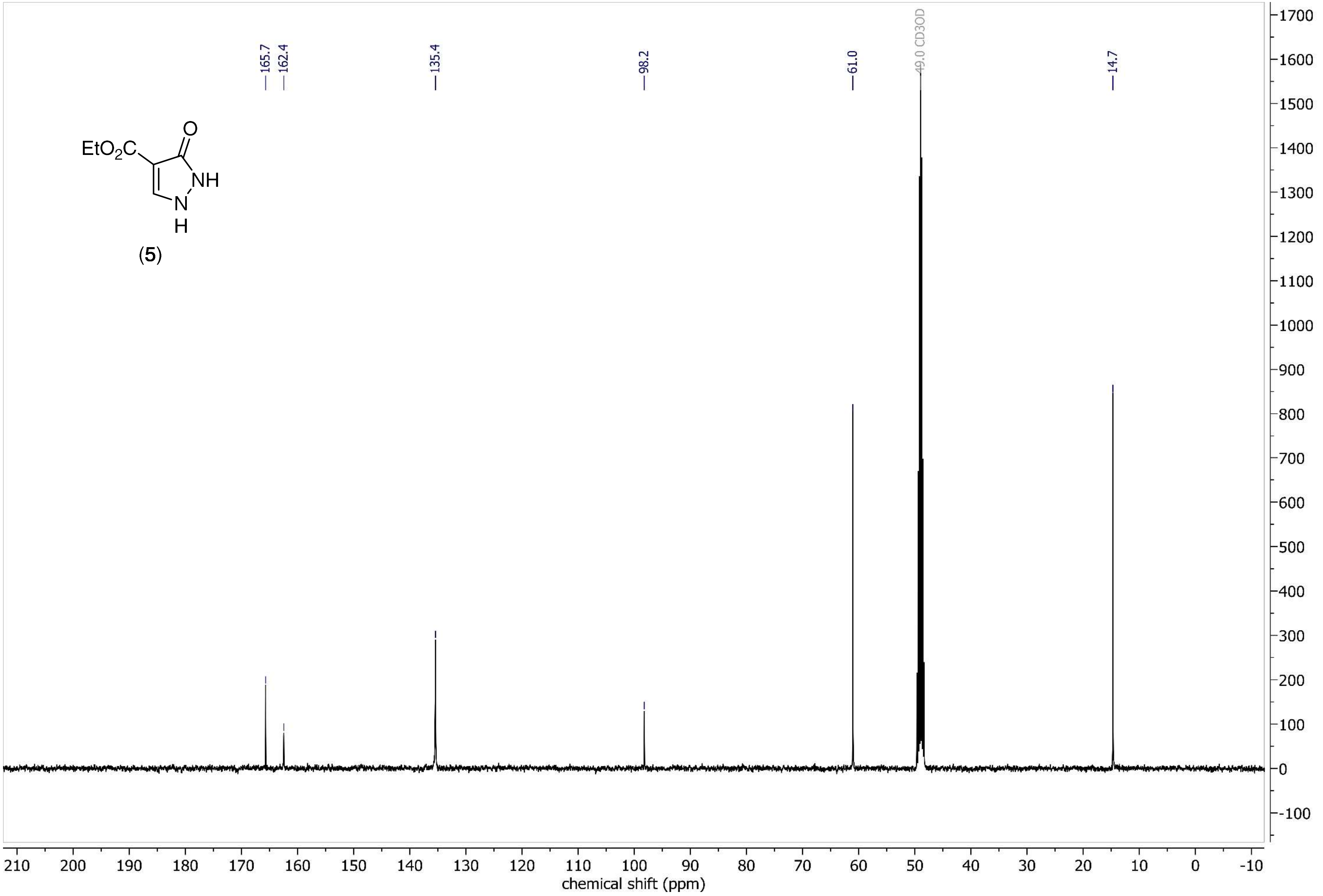

Figure 18: $^{13}C\{^{1}H\}$-NMR spectrum of ethyl 3-oxo-2,3-dihydro-1*H*-pyrazole-4-carboxylate (**5**) in CD_3OD.

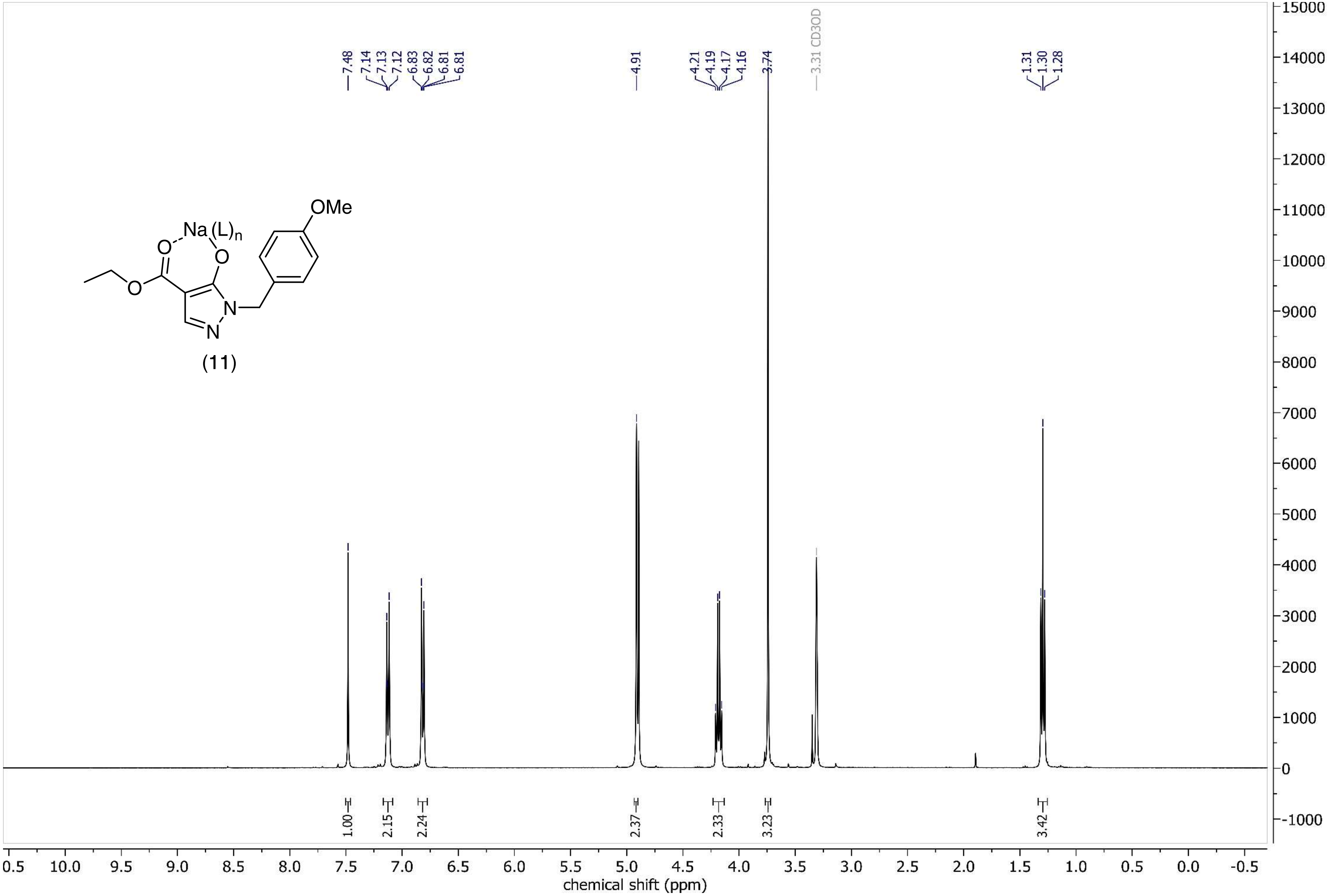

Figure S19: ^{1}H-NMR spectrum of (4-(ethoxycarbonyl)-1-(4-methoxybenzyl)-1*H*-pyrazol-5-olate)sodium (**11**).

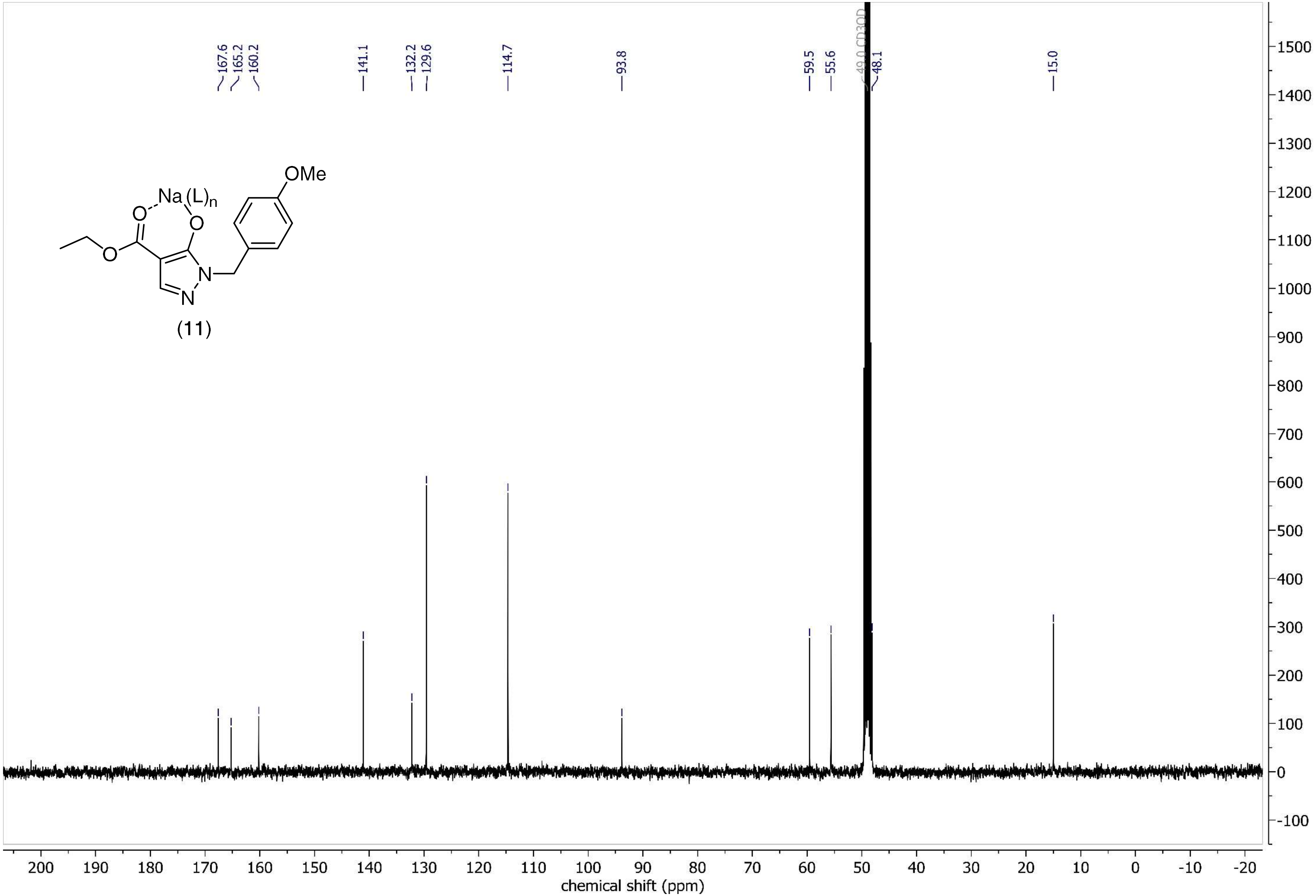

Figure S20: $^{13}C\{^{1}H\}$-NMR spectrum of (4-(ethoxycarbonyl)-1-(4-methoxybenzyl)-1*H*-pyrazol-5-olate)sodium (**11**).

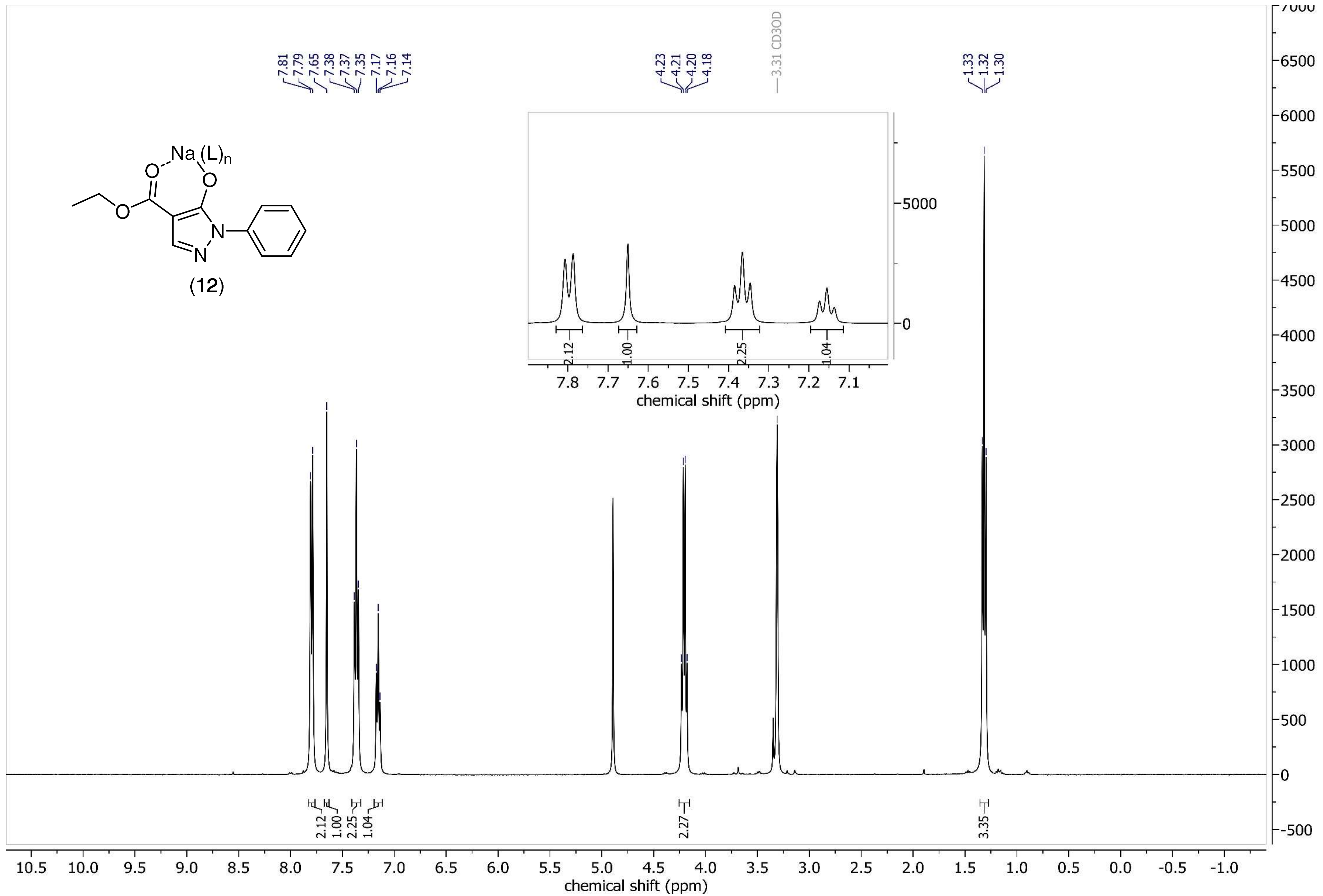

Figure S21: ^{1}H-NMR spectrum of (4-(ethoxycarbonyl)-1-phenyl-1*H*-pyrazol-5-olate)sodium (**12**).

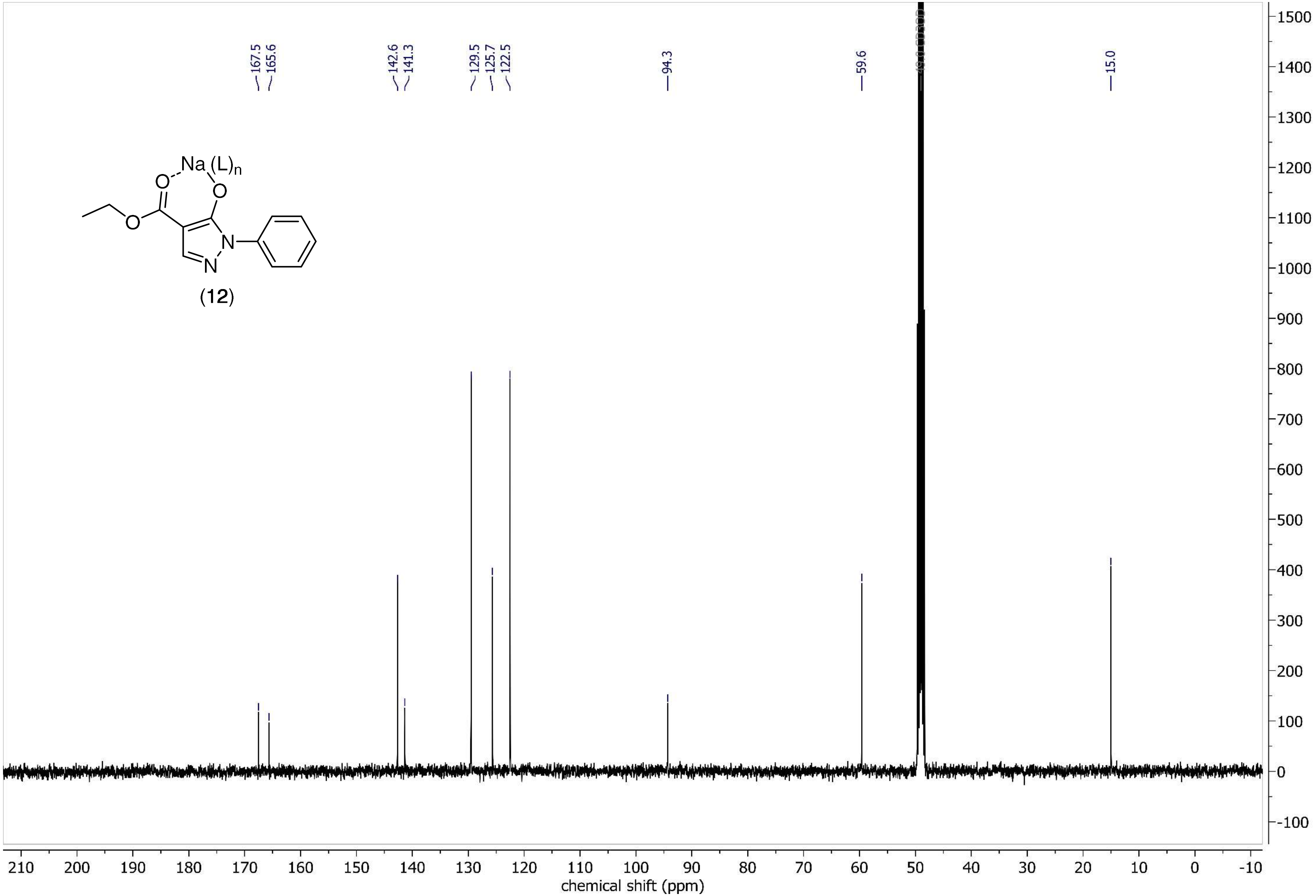

Figure S22: $^{13}C\{^1H\}$-NMR spectrum of (4-(ethoxycarbonyl)-1-phenyl-1*H*-pyrazol-5-olate)sodium (**12**).

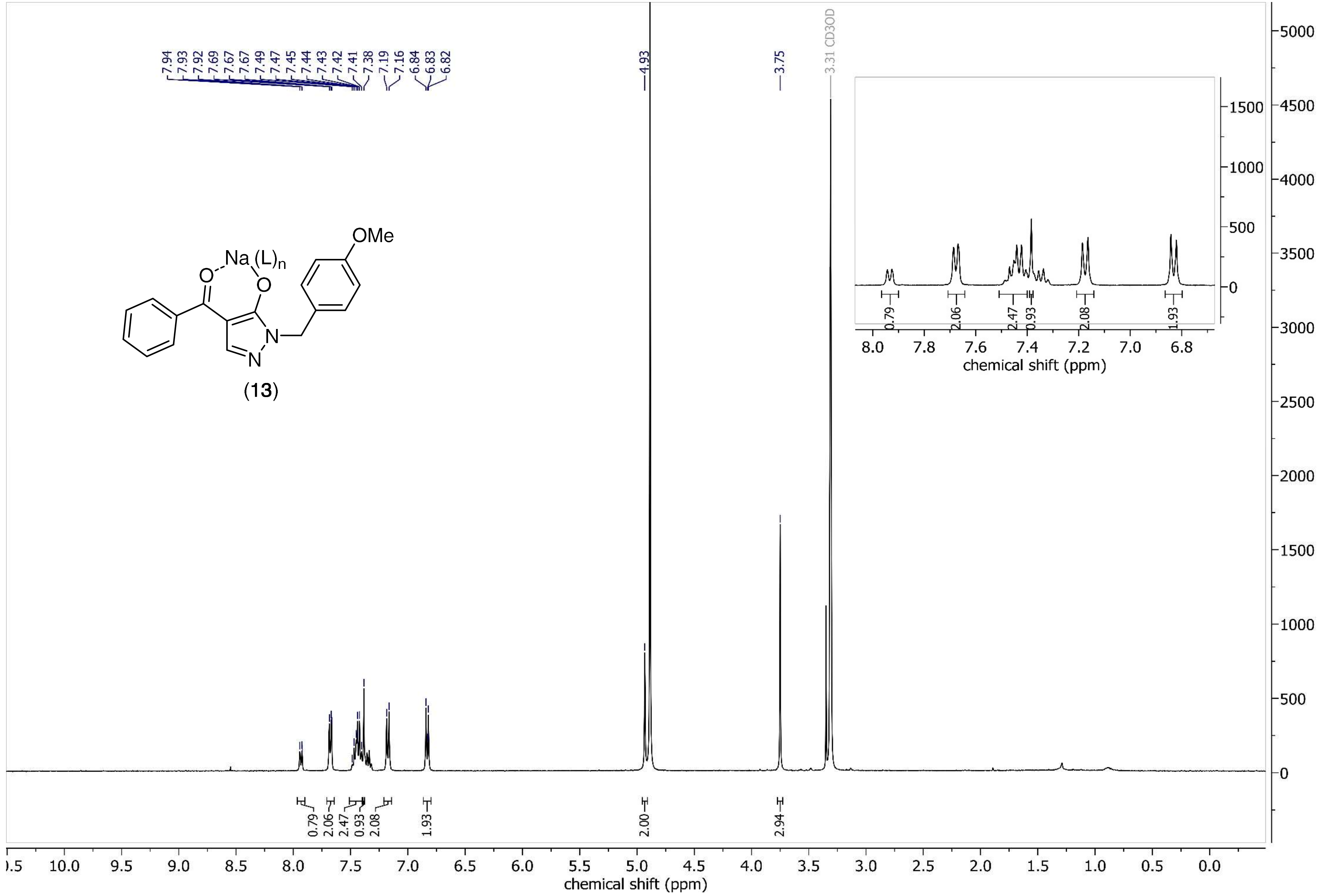

Figure S23: ^{1}H-NMR spectrum of (4-benzoyl-1-(4-methoxybenzyl)-1*H*-pyrazol-5-olate)sodium (**13**).

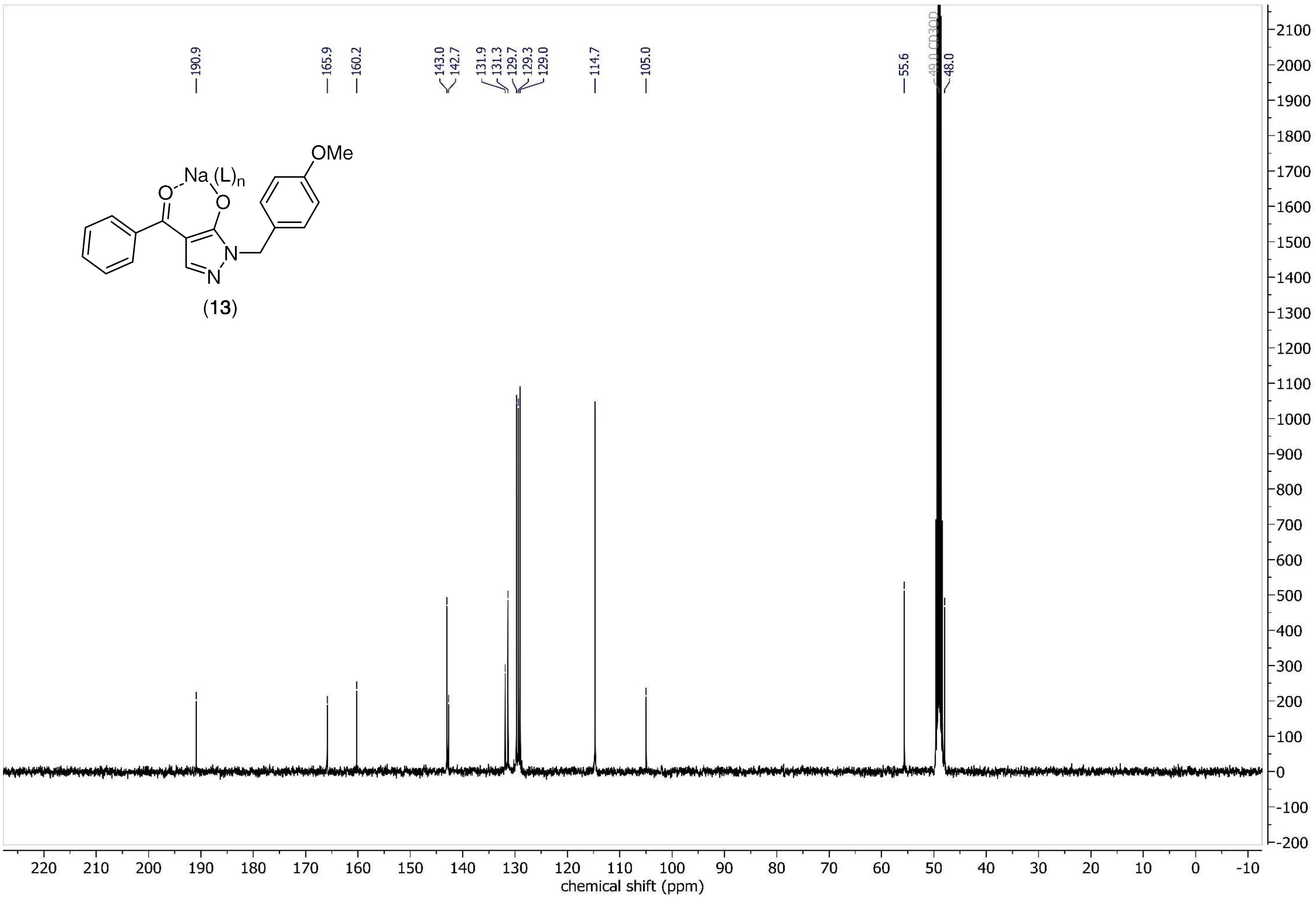

Figure S24: $^{13}C\{^{1}H\}$-NMR spectrum of (4-benzoyl-1-(4-methoxybenzyl)-1*H*-pyrazol-5-olate)sodium (**13**).

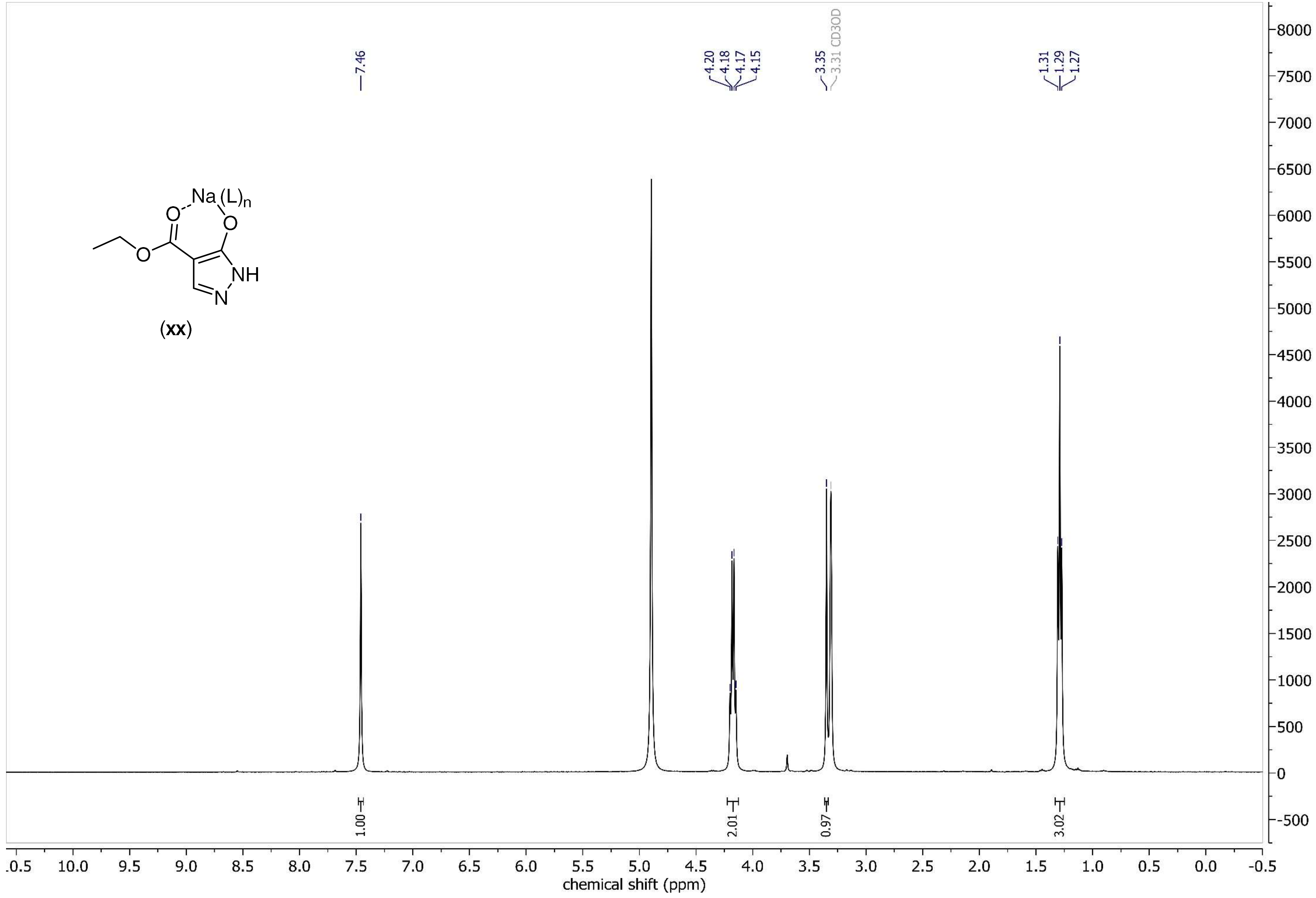

Figure S25: ^{1}H-NMR spectrum of (4-(ethoxycarbonyl)-1*H*-pyrazol-5-olate)sodium (**14**).

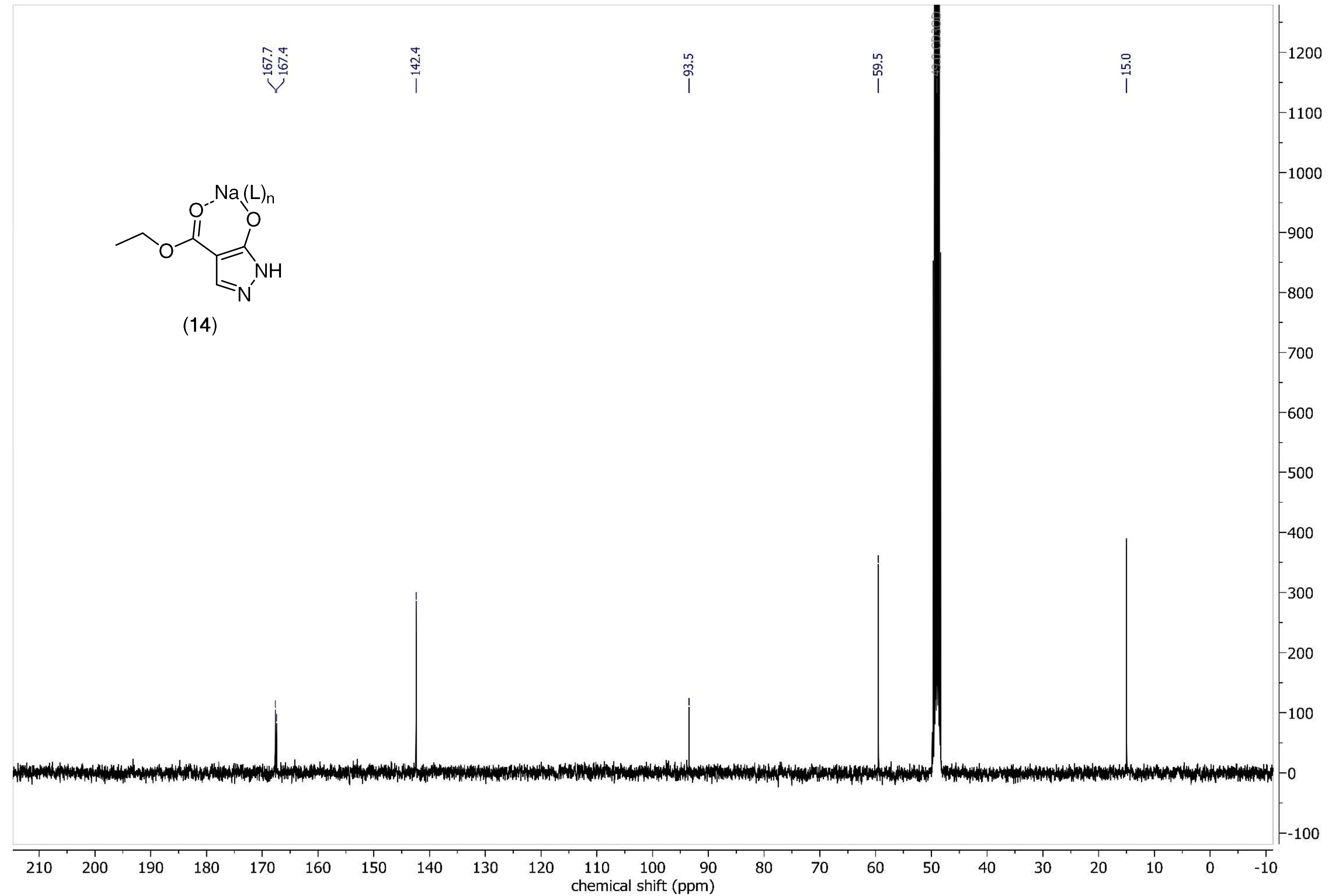

Figure S26: $^{13}C\{^{1}H\}$-NMR spectrum of (4-(ethoxycarbonyl)-1H-pyrazol-5-olate)sodium (**14**).

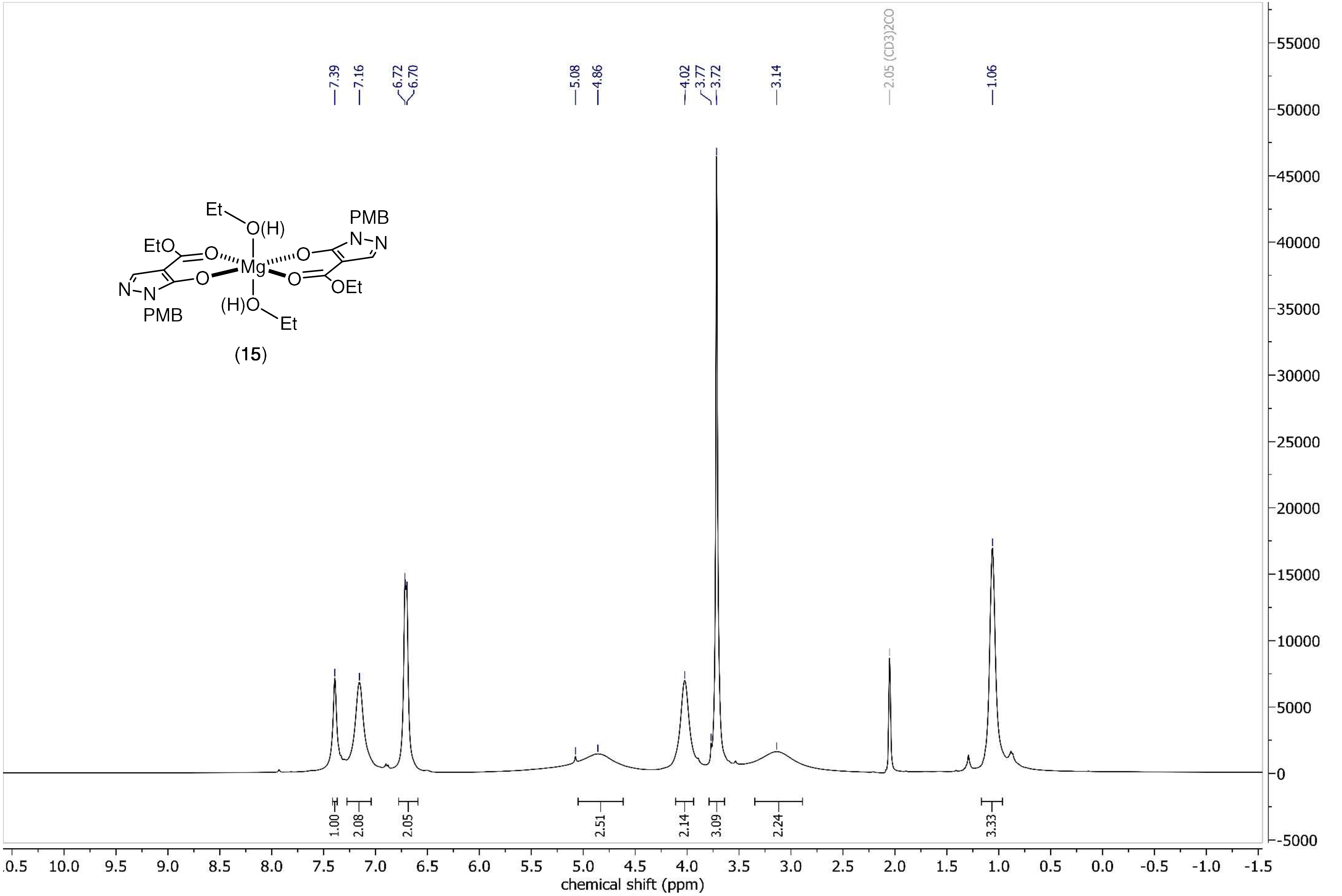

Figure S27: ^{1}H-NMR spectrum of bis(ethanol) bis(4-(ethoxycarbonyl)-1-(4-methoxybenzyl)-1*H*-pyrazol-5-olate)magnesium (**15**).

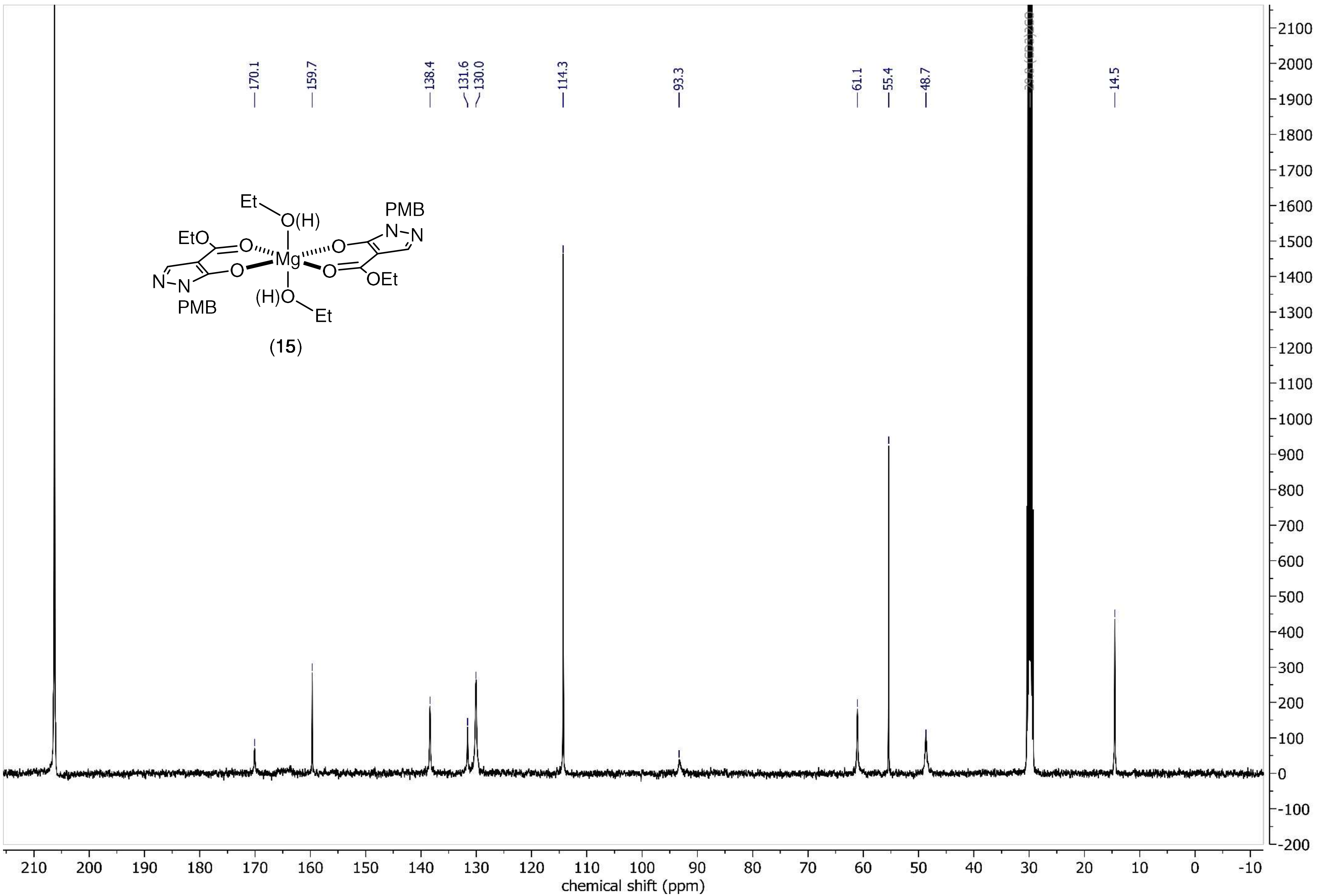

Figure S28: $^{13}C\{^{1}H\}$-NMR spectrum of bis(ethanol) bis(4-(ethoxycarbonyl)-1-(4-methoxybenzyl)-1*H*-pyrazol-5-olate)magnesium (**15**).

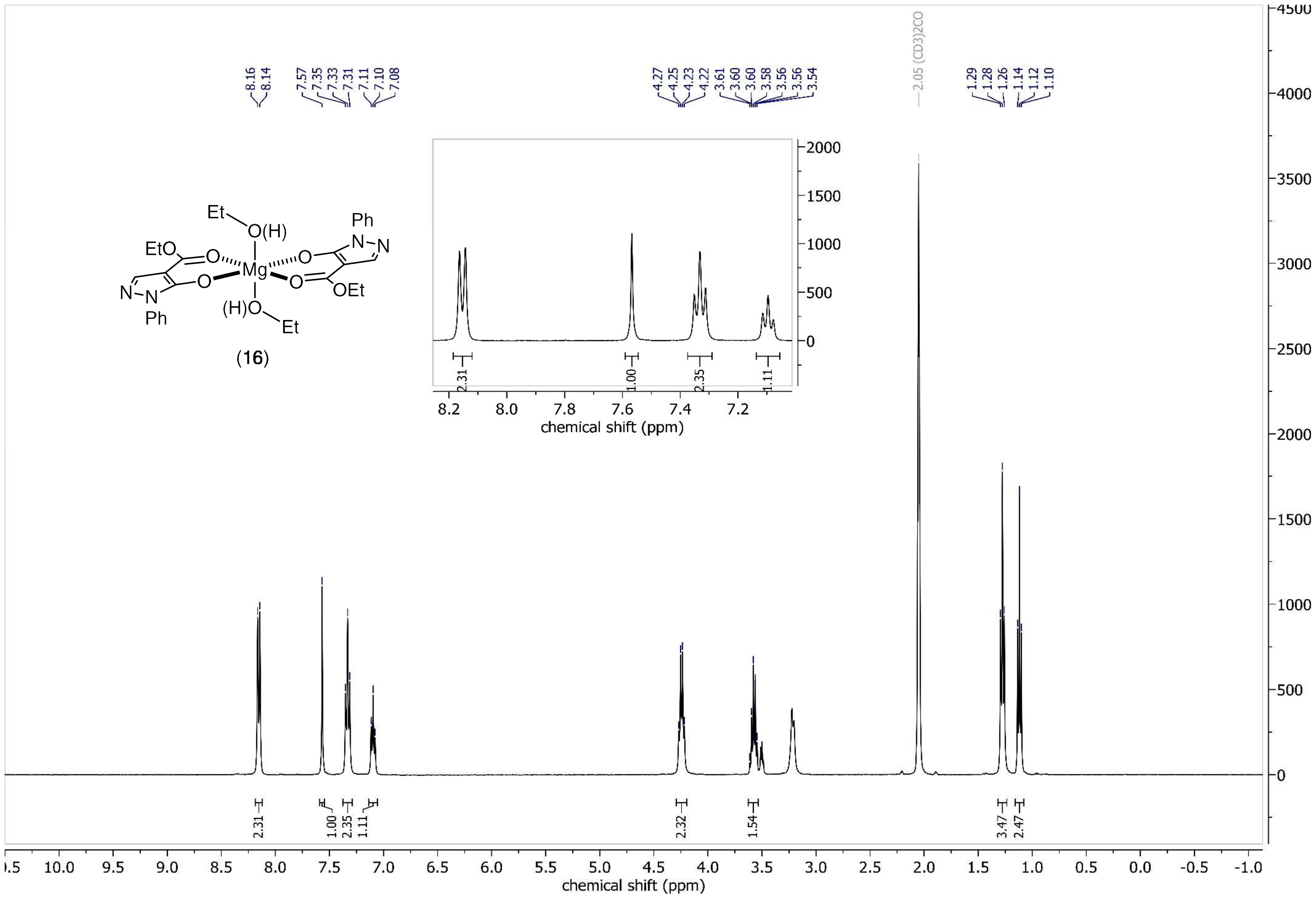

Figure S29: ^{1}H-NMR spectrum of bis(ethanol) bis(4-(ethoxycarbonyl)-1-phenyl-1*H*-pyrazol-5-olate)magnesium (**16**).

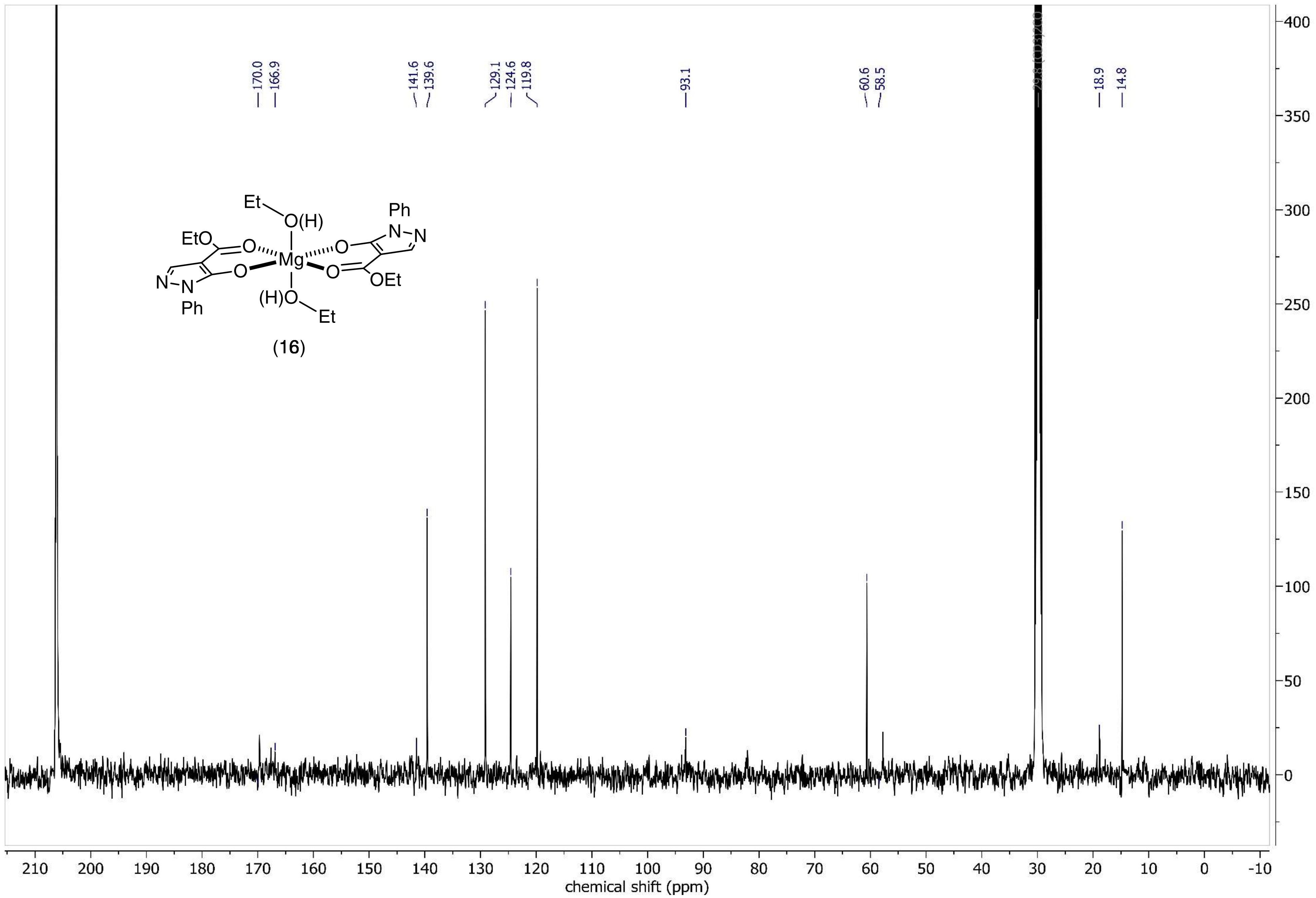

Figure S30: $^{13}C\{^{1}H\}$-NMR spectrum of bis(ethanol) bis(4-(ethoxycarbonyl)-1-phenyl-1*H*-pyrazol-5-olate)magnesium (**16**).

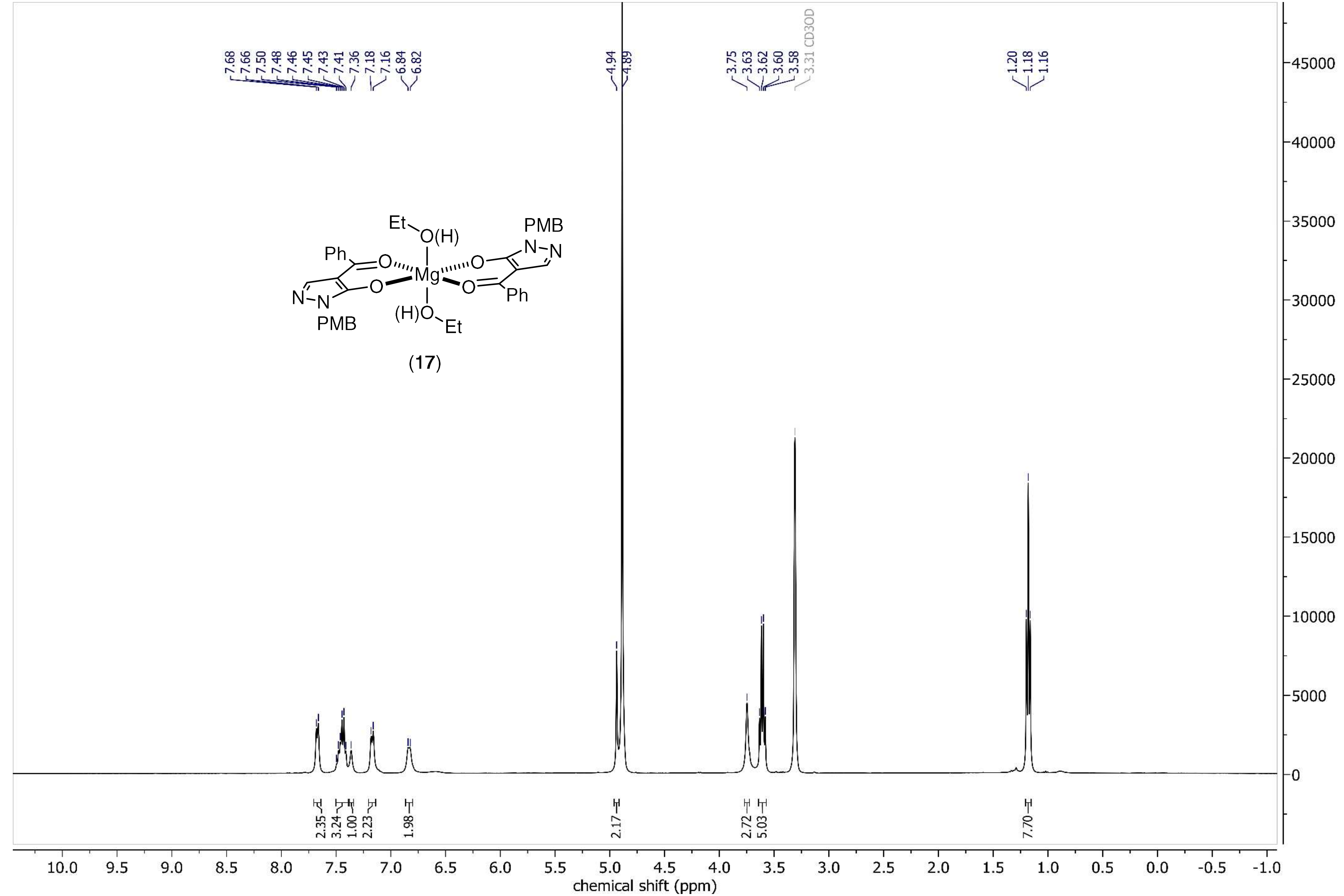

Figure S31: ^{1}H-NMR spectrum of bis(ethanol) bis(4-benzoyl-1-(4-methoxybenzyl)-1*H*-pyrazol-5-olate)magnesium (**17**).

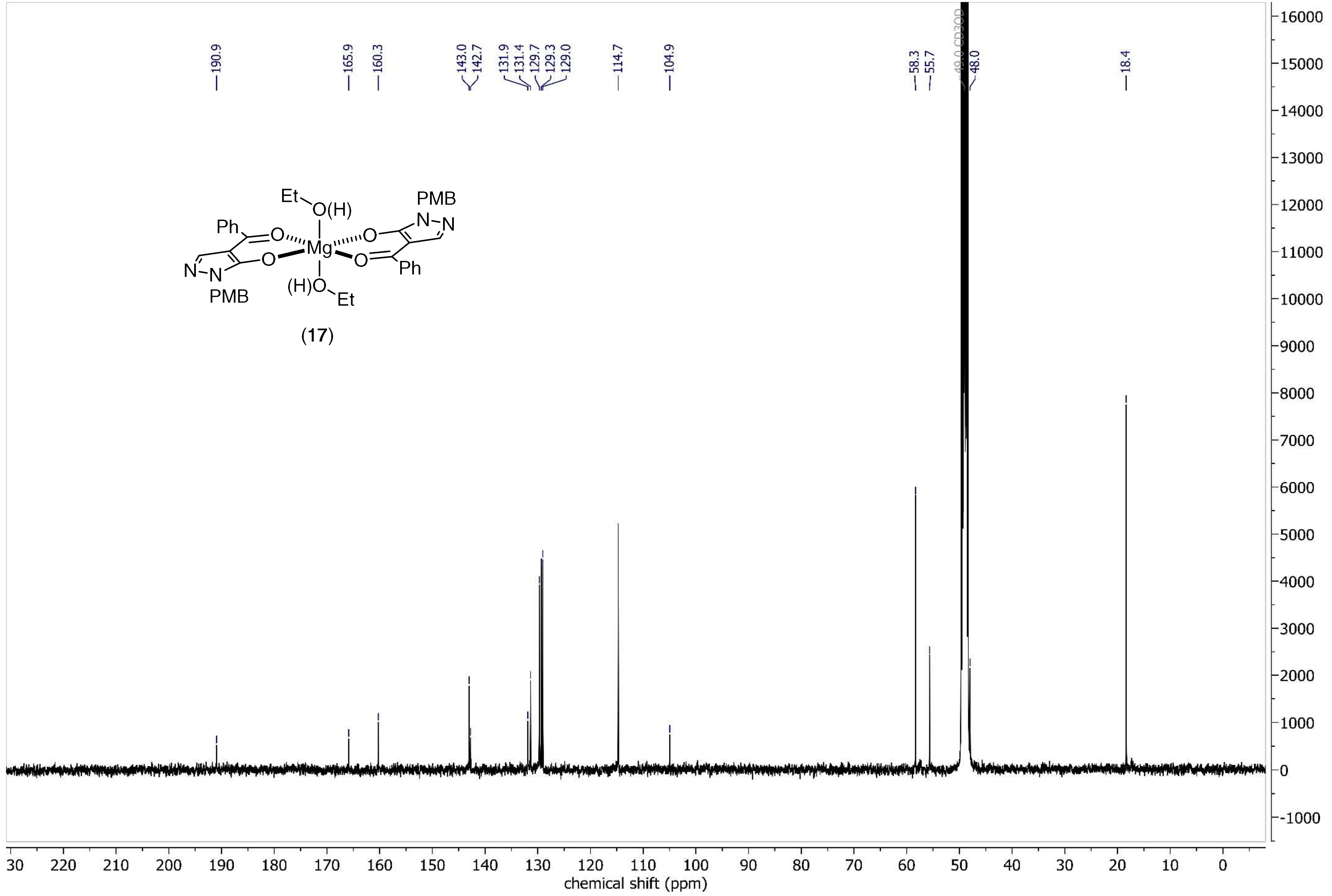

Figure S32: $^{13}C\{^1H\}$-NMR spectrum of bis(ethanol) bis(4-benzoyl-1-(4-methoxybenzyl)-1*H*-pyrazol-5-olate)magnesium (**17**).

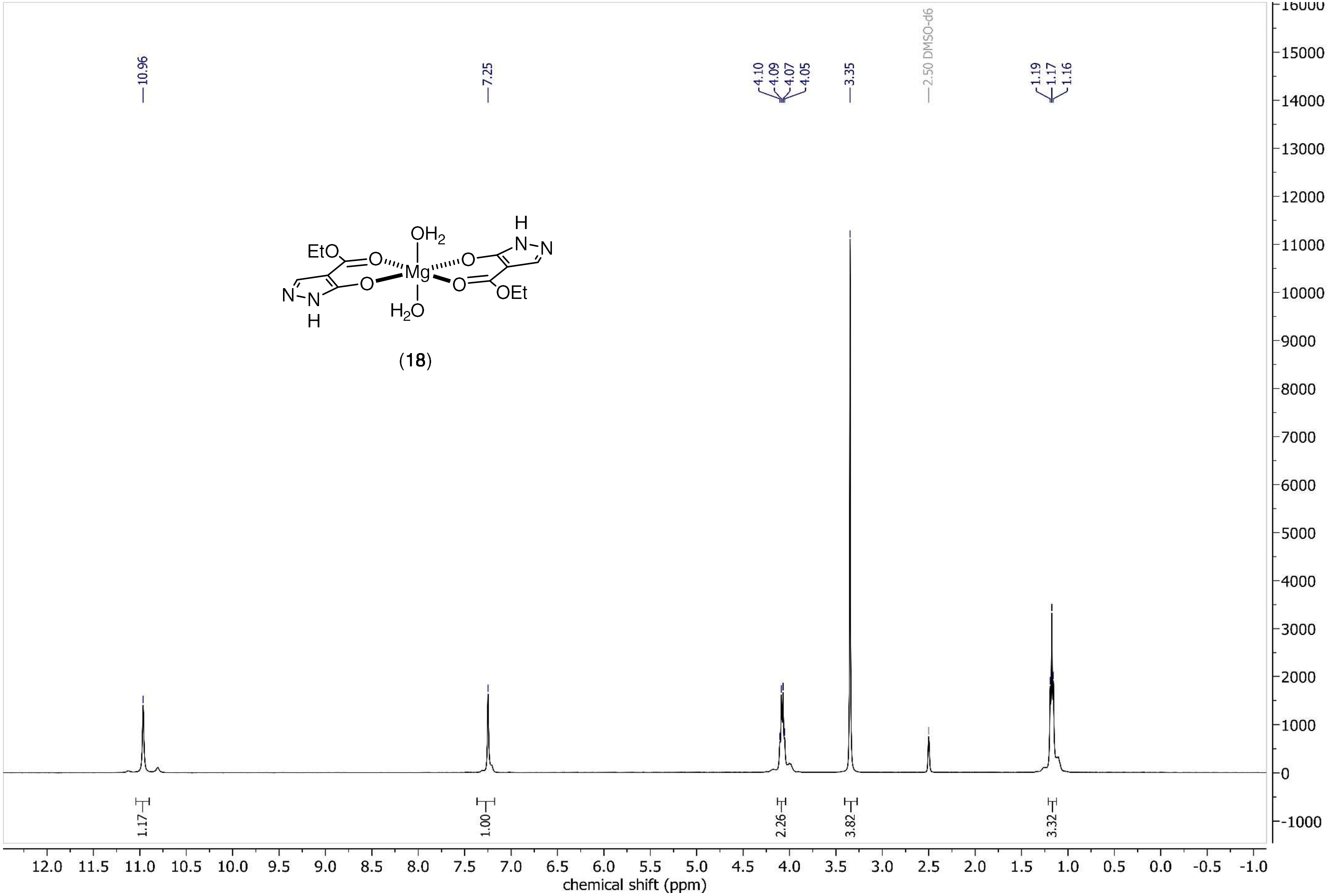

Figure S33: ^{1}H-NMR spectrum of bis(aqua) bis(4-(ethoxycarbonyl)-1*H*-pyrazol-5-olate)magnesium (**18**).

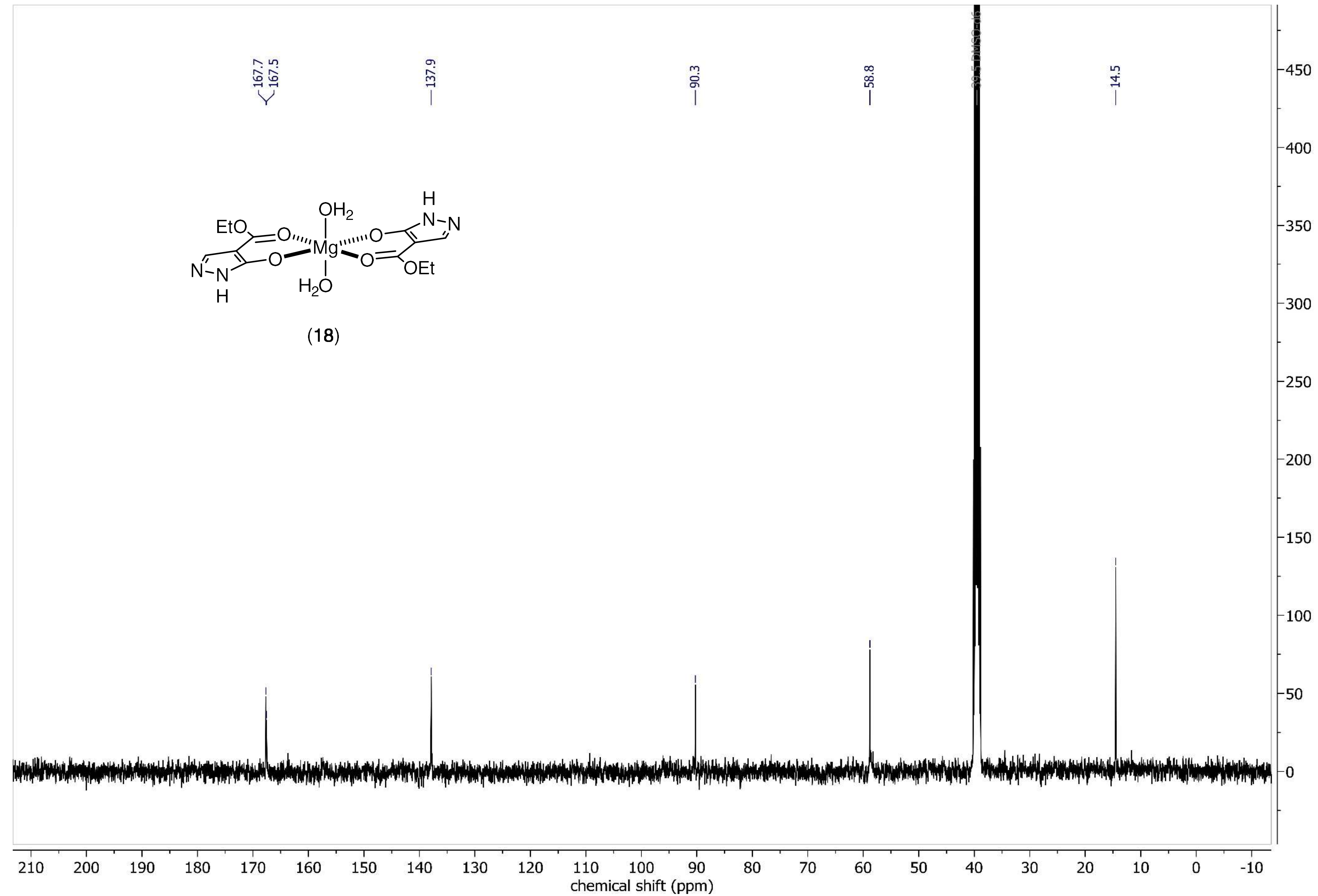

Figure S34: $^{13}C\{^{1}H\}$-NMR spectrum of bis(aqua) bis(4-(ethoxycarbonyl)-1*H*-pyrazol-5-olate)magnesium (**18**).

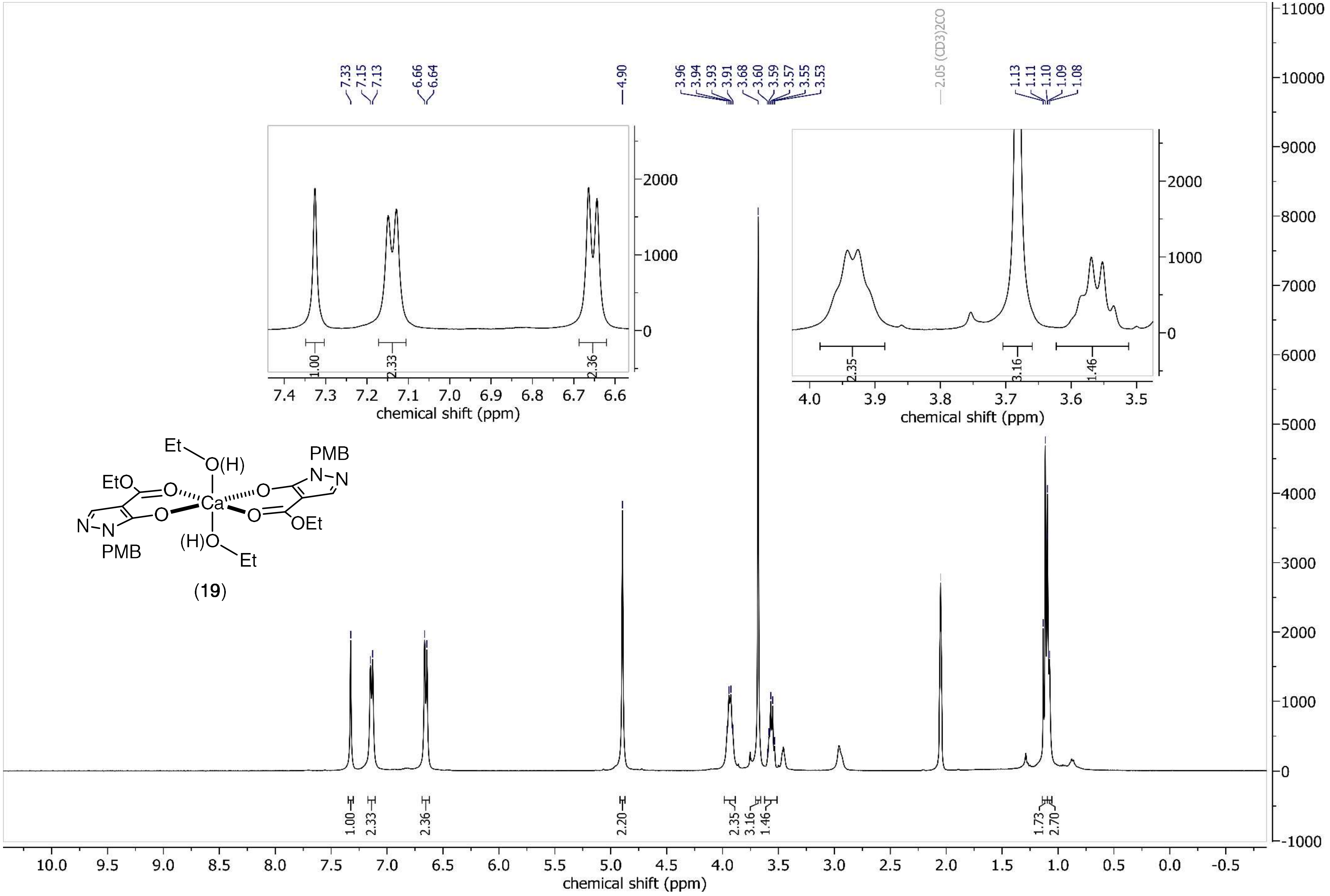

Figure S35: ^{1}H-NMR spectrum of bis(ethanol) bis(4-(ethoxycarbonyl)-1-(4-methoxybenzyl)-1*H*-pyrazol-5-olate)calcium (**19**).

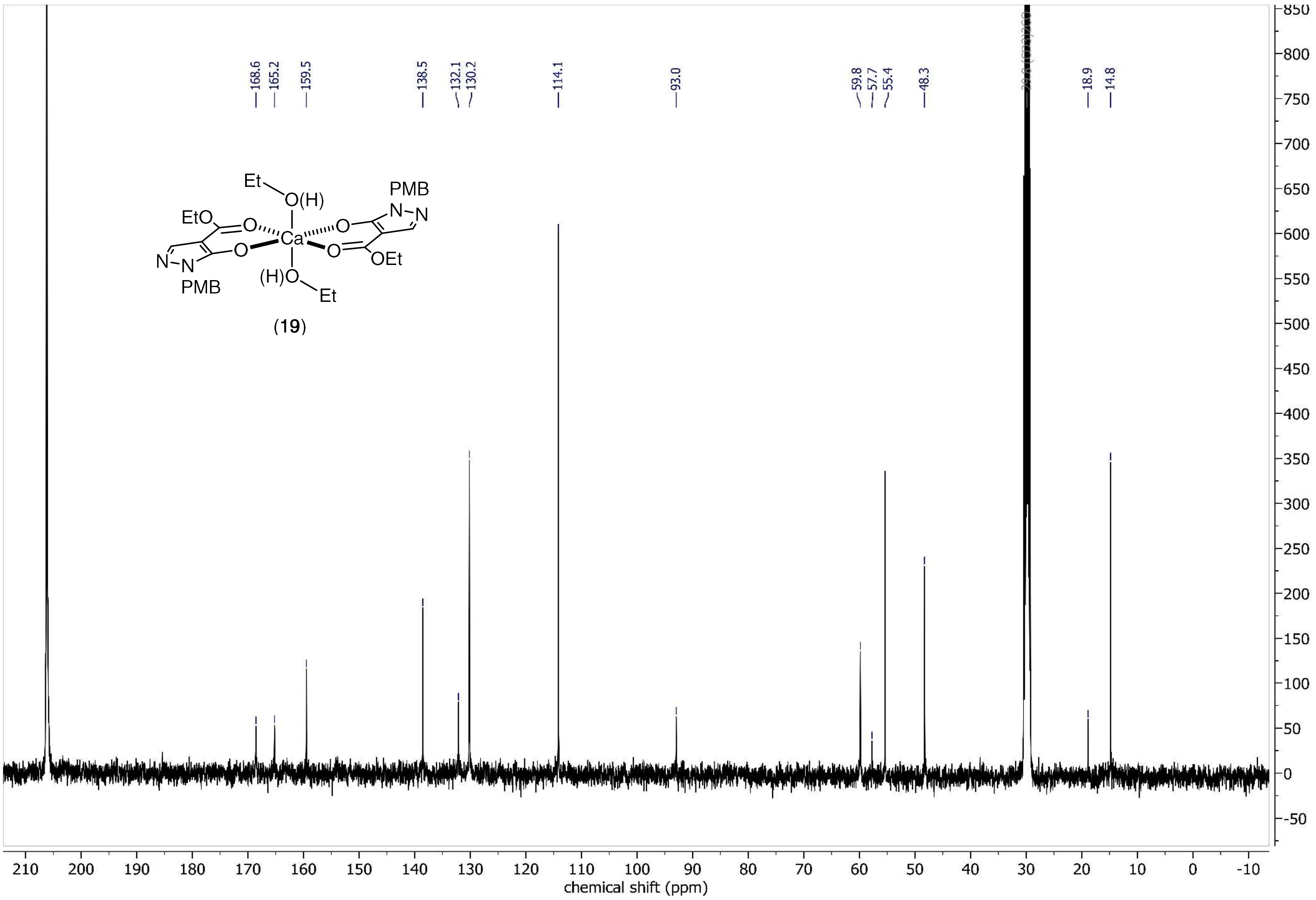

Figure S36: $^{13}C\{^1H\}$-NMR spectrum of bis(ethanol) bis(4-(ethoxycarbonyl)-1-(4-methoxybenzyl)-1*H*-pyrazol-5-olate)calcium (**19**).

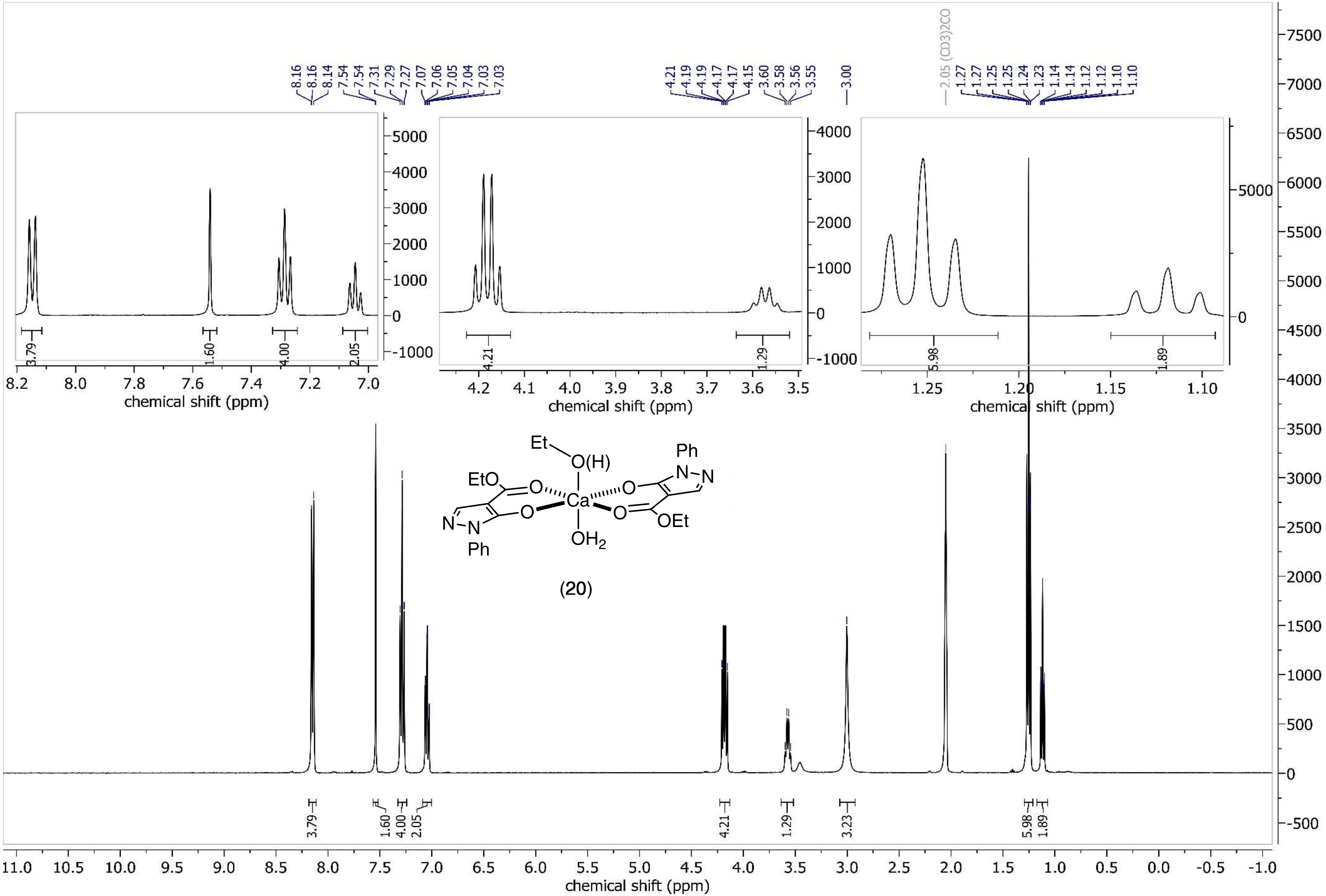

Figure S37: ^{1}H-NMR spectrum of (aqua)(ethanol) bis(4-(ethoxycarbonyl)-1-phenyl-1*H*-pyrazol-5-olate)calcium (**20**).

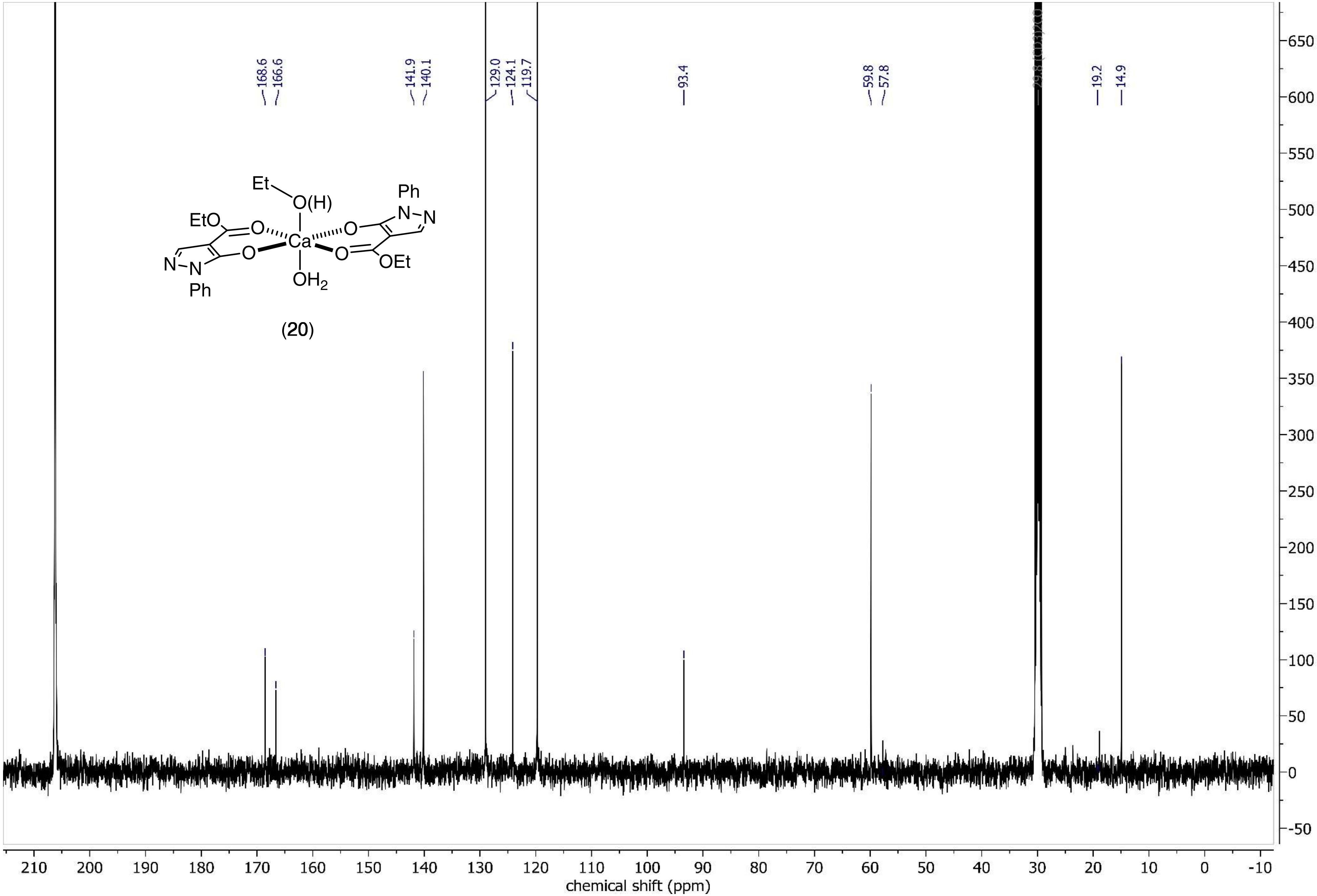

Figure S38: $^{13}C\{^1H\}$-NMR spectrum of (aqua)(ethanol) bis(4-(ethoxycarbonyl)-1-phenyl-1*H*-pyrazol-5-olate)calcium (**20**).

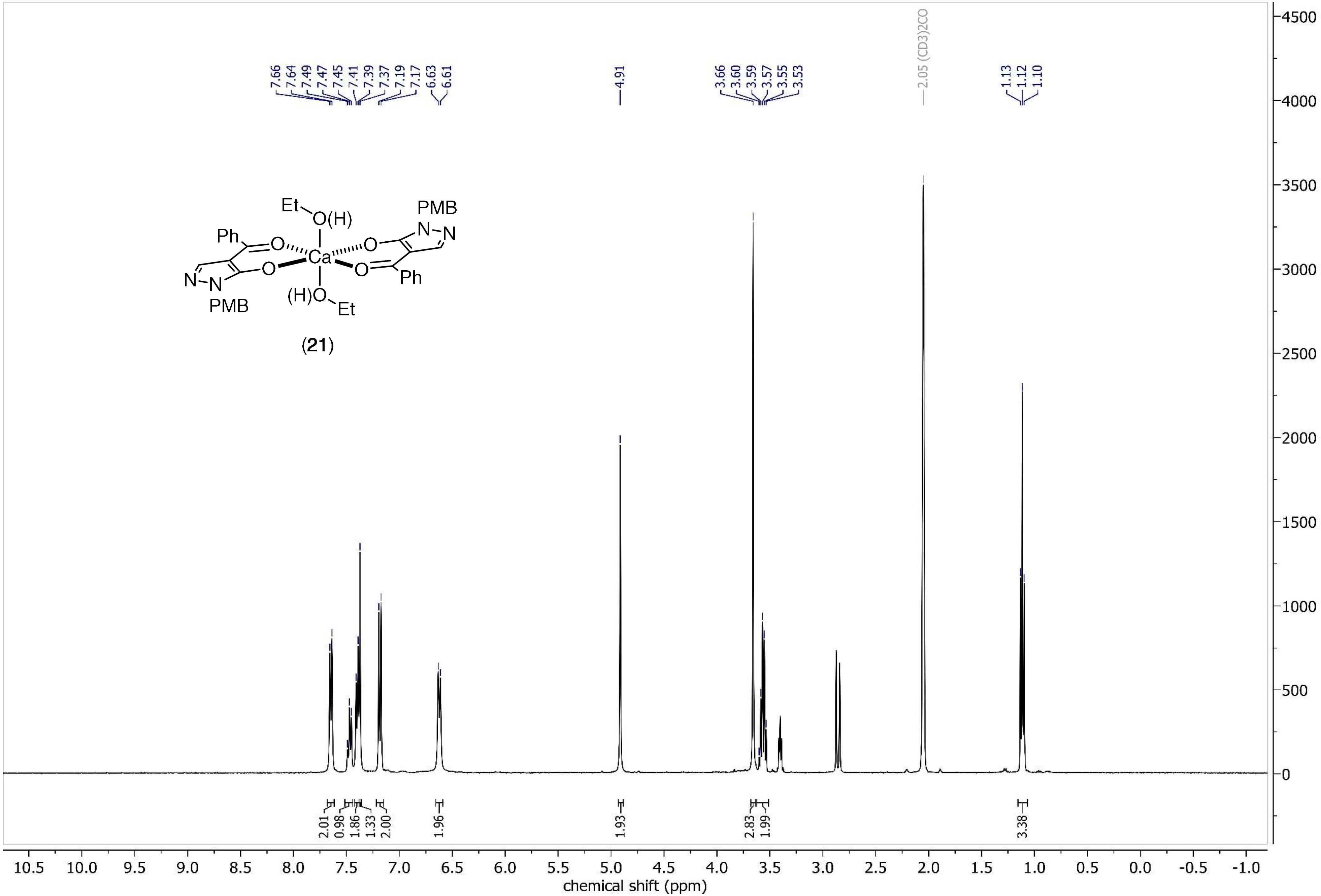

Figure S39: ^{1}H-NMR spectrum of bis(ethanol) bis(4-benzoyl-1-(4-methoxybenzyl)-1*H*-pyrazol-5-olate)calcium (**21**).

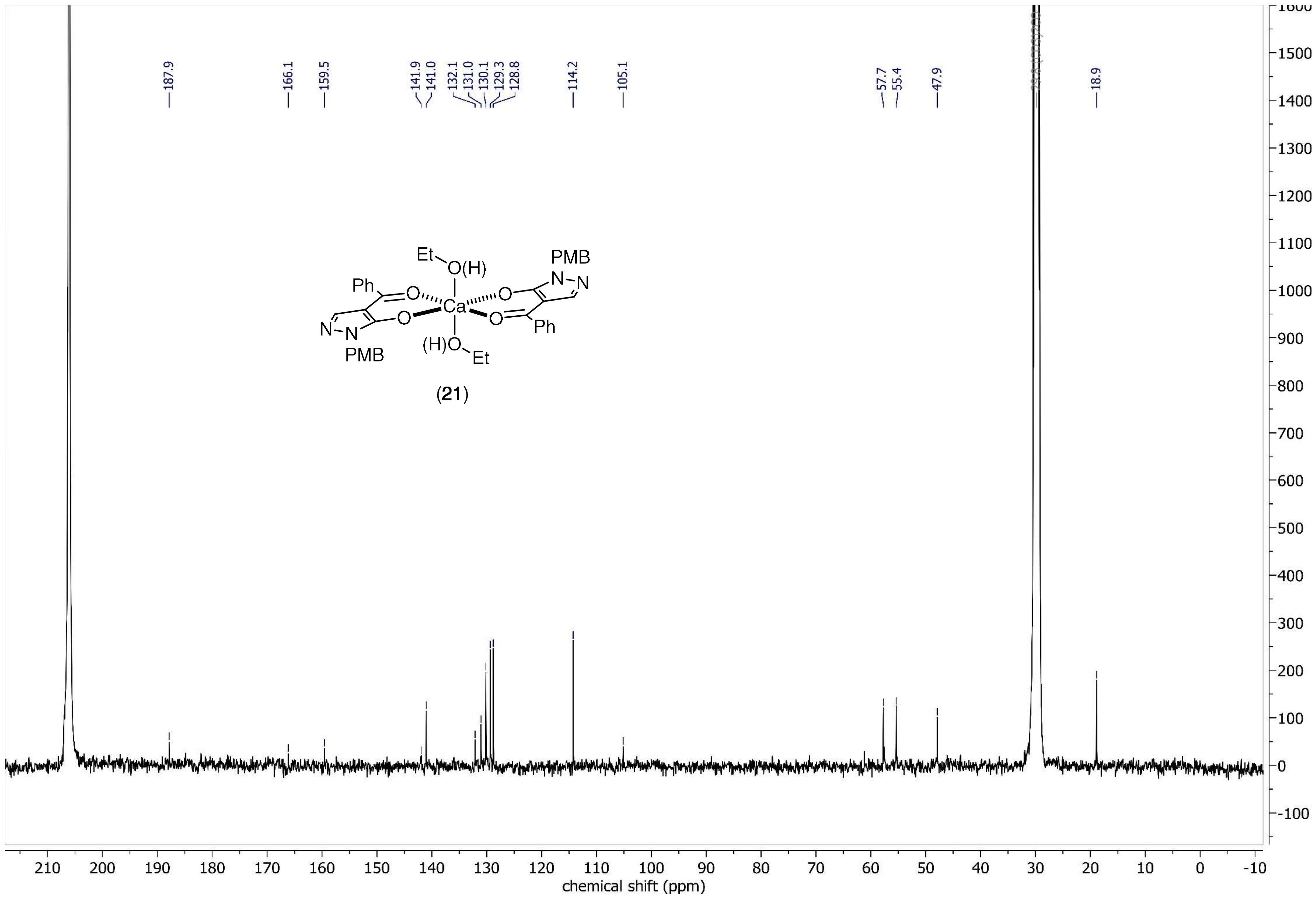

Figure S40: $^{13}C\{^1H\}$-NMR spectrum of bis(ethanol) bis(4-benzoyl-1-(4-methoxybenzyl)-1*H*-pyrazol-5-olate)calcium (**21**).

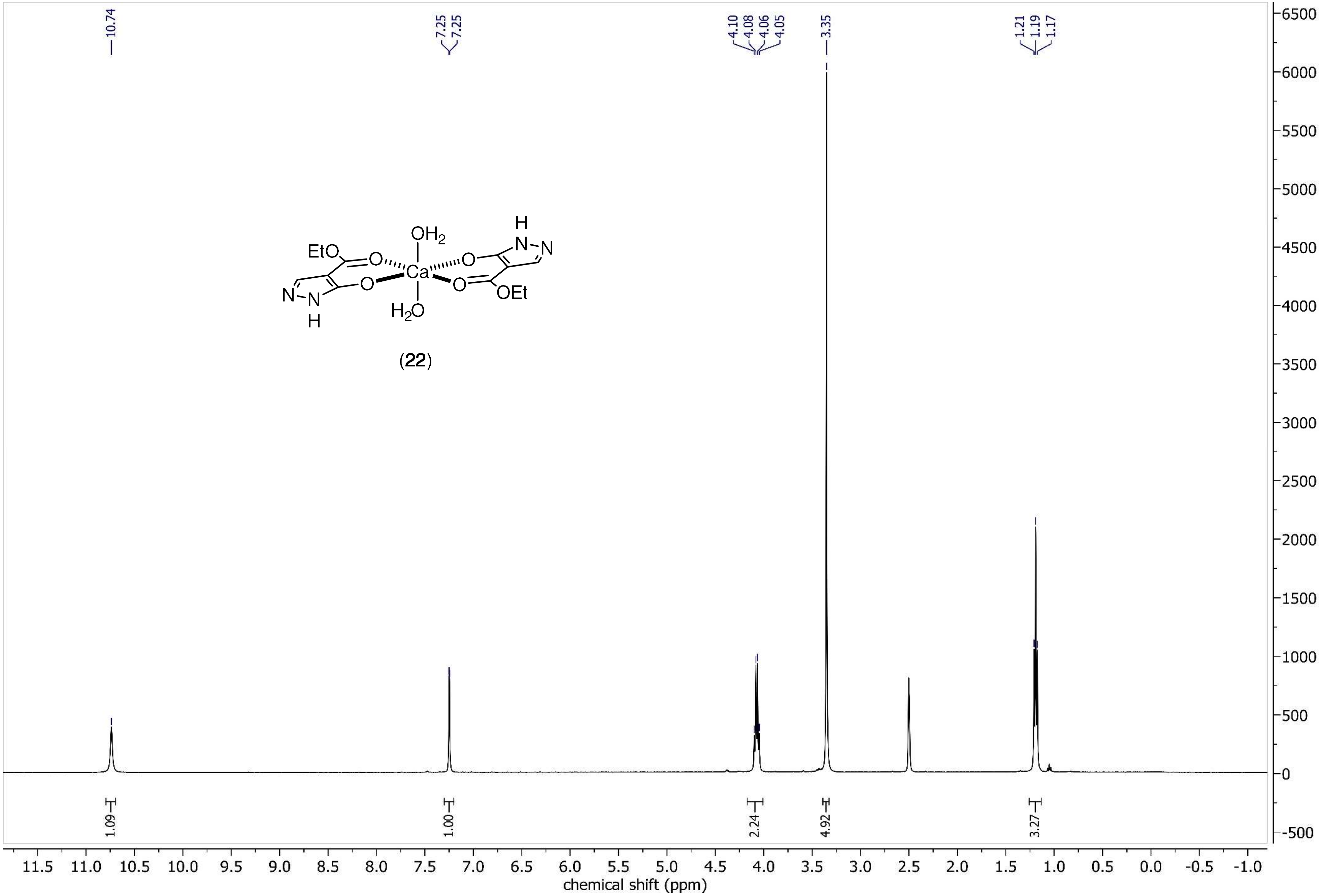

Figure S41: ^{1}H-NMR spectrum of bis(aqua) bis(4-(ethoxycarbonyl)-1*H*-pyrazol-5-olate)calcium (**22**).

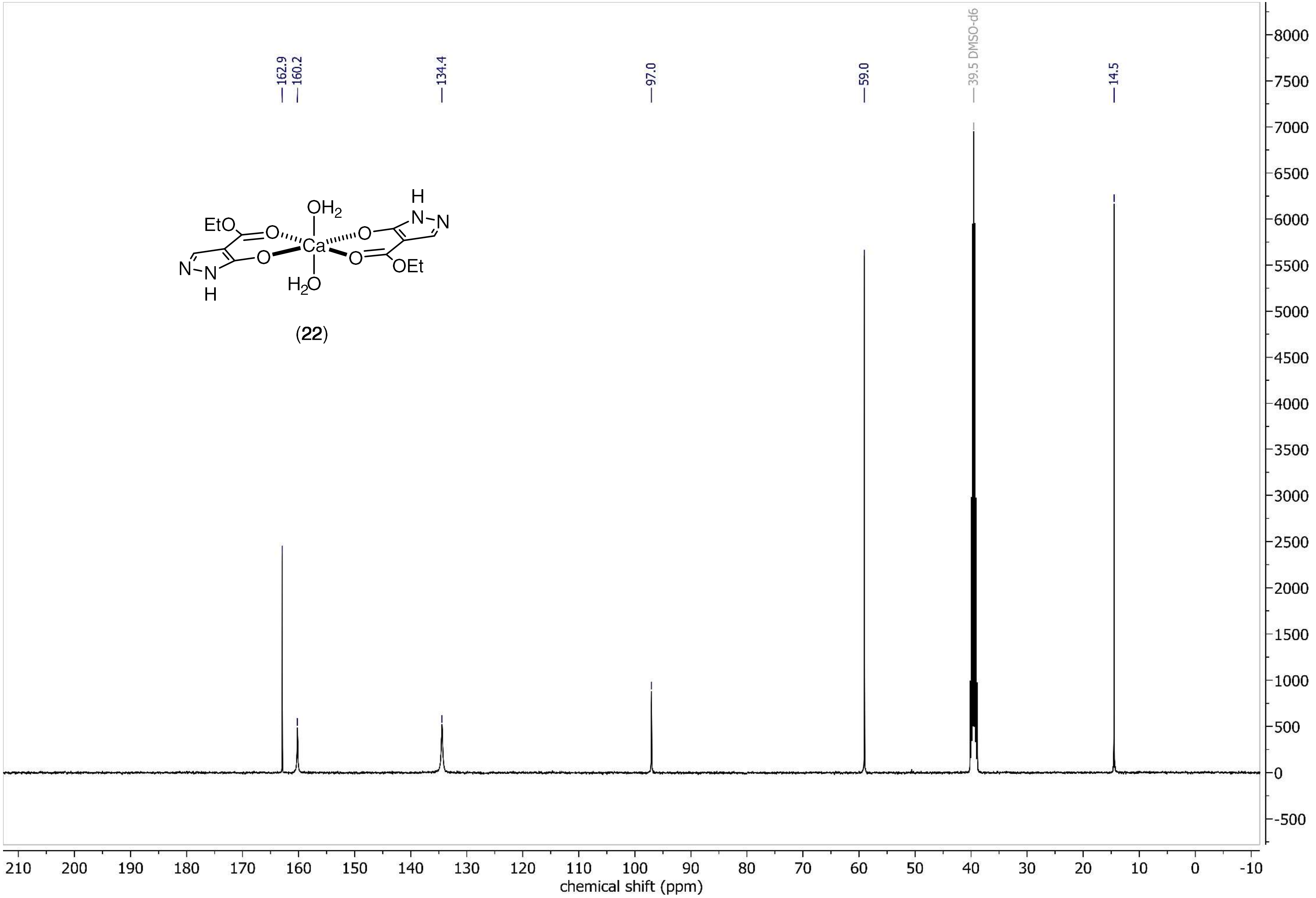

Figure S42: $^{13}C\{^{1}H\}$-NMR spectrum of bis(aqua) bis(4-(ethoxycarbonyl)-1*H*-pyrazol-5-olate)calcium (**22**).

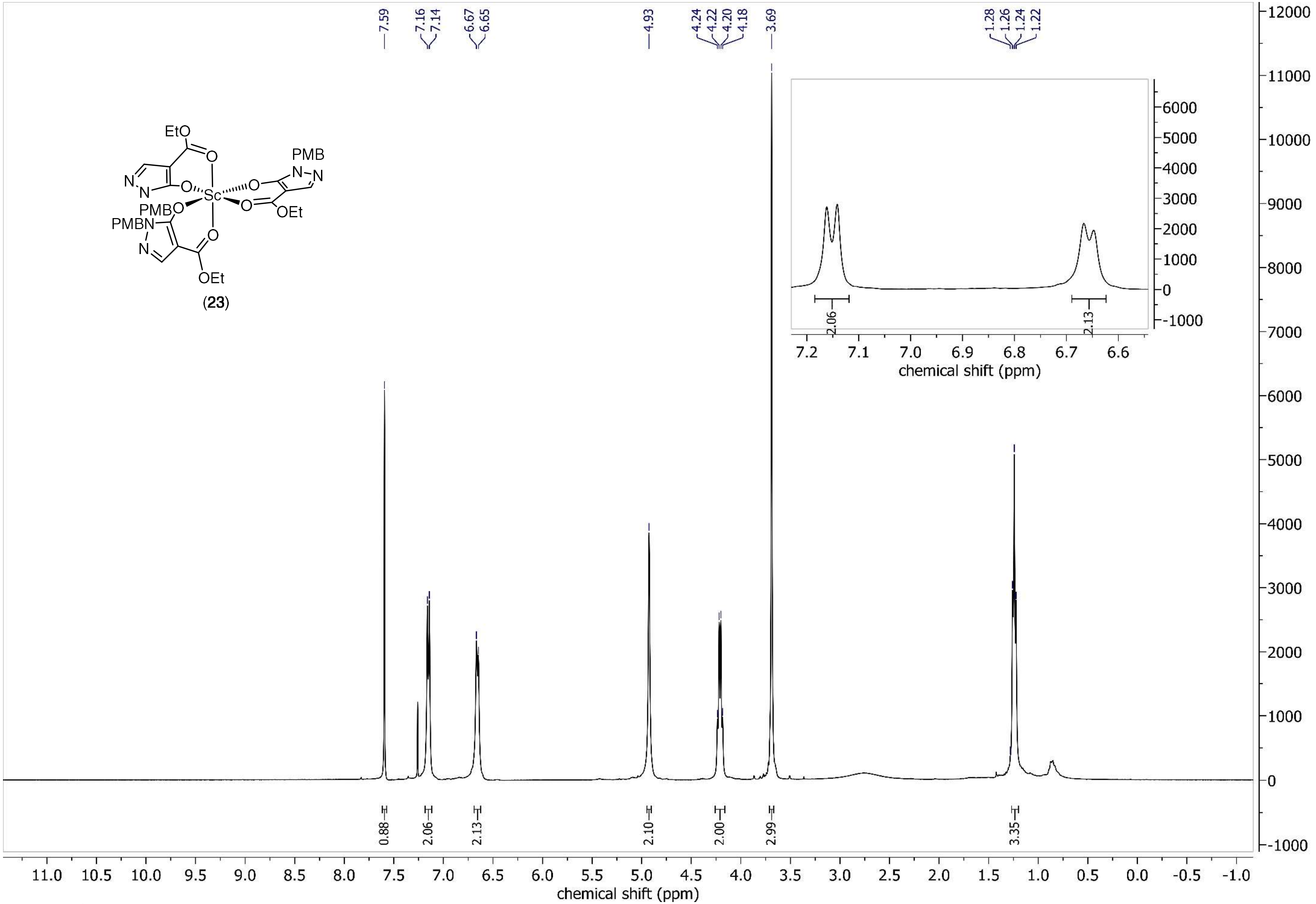

(**23**)

Figure S43: ^{1}H-NMR spectrum of tris((4-(ethoxycarbonyl)-1-(4-methoxybenzyl)-1*H*-pyrazol-5-yl)oxy)scandium (**23**).

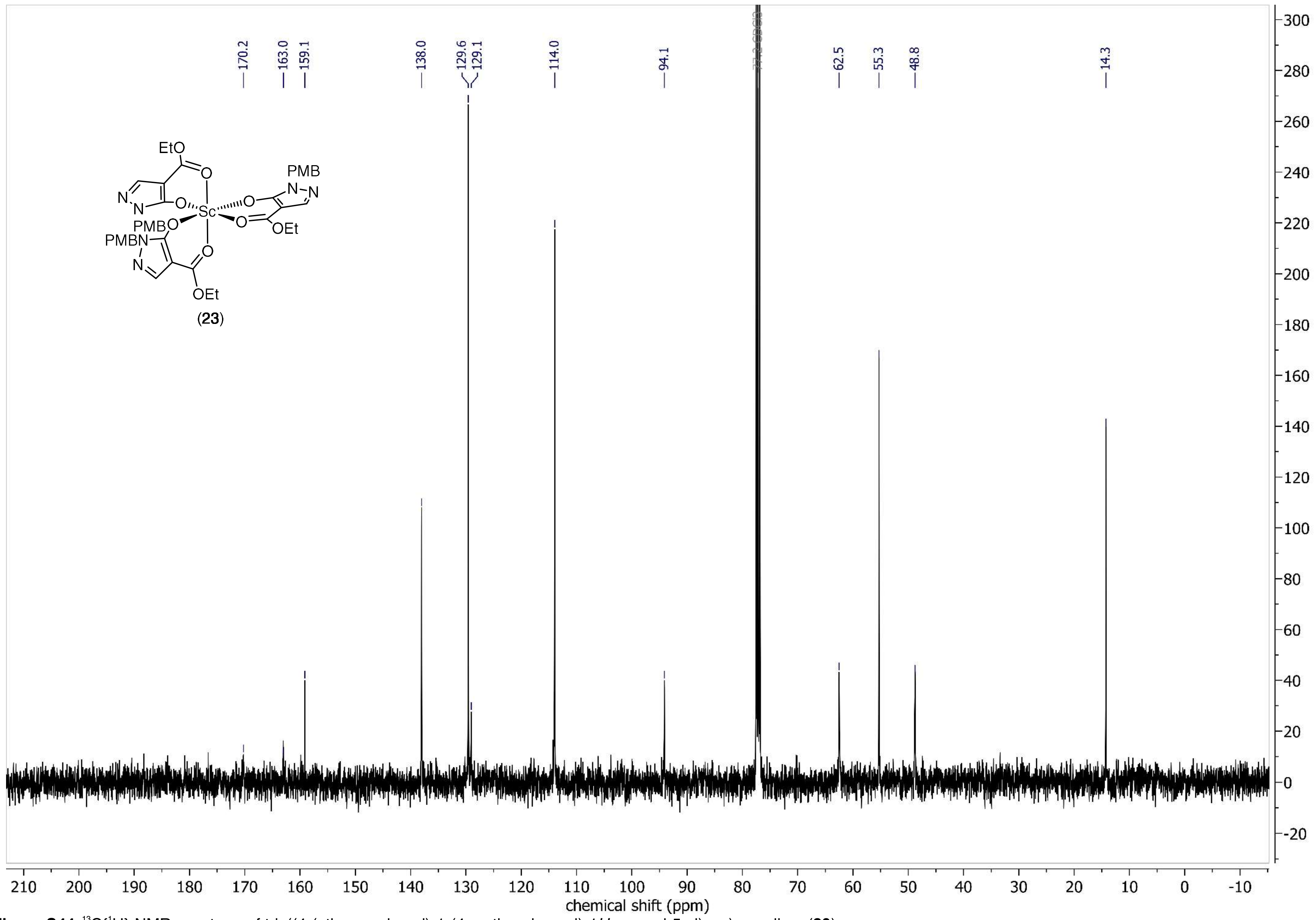

Figure S44: $^{13}C\{^{1}H\}$-NMR spectrum of tris((4-(ethoxycarbonyl)-1-(4-methoxybenzyl)-1*H*-pyrazol-5-yl)oxy)scandium (**23**).

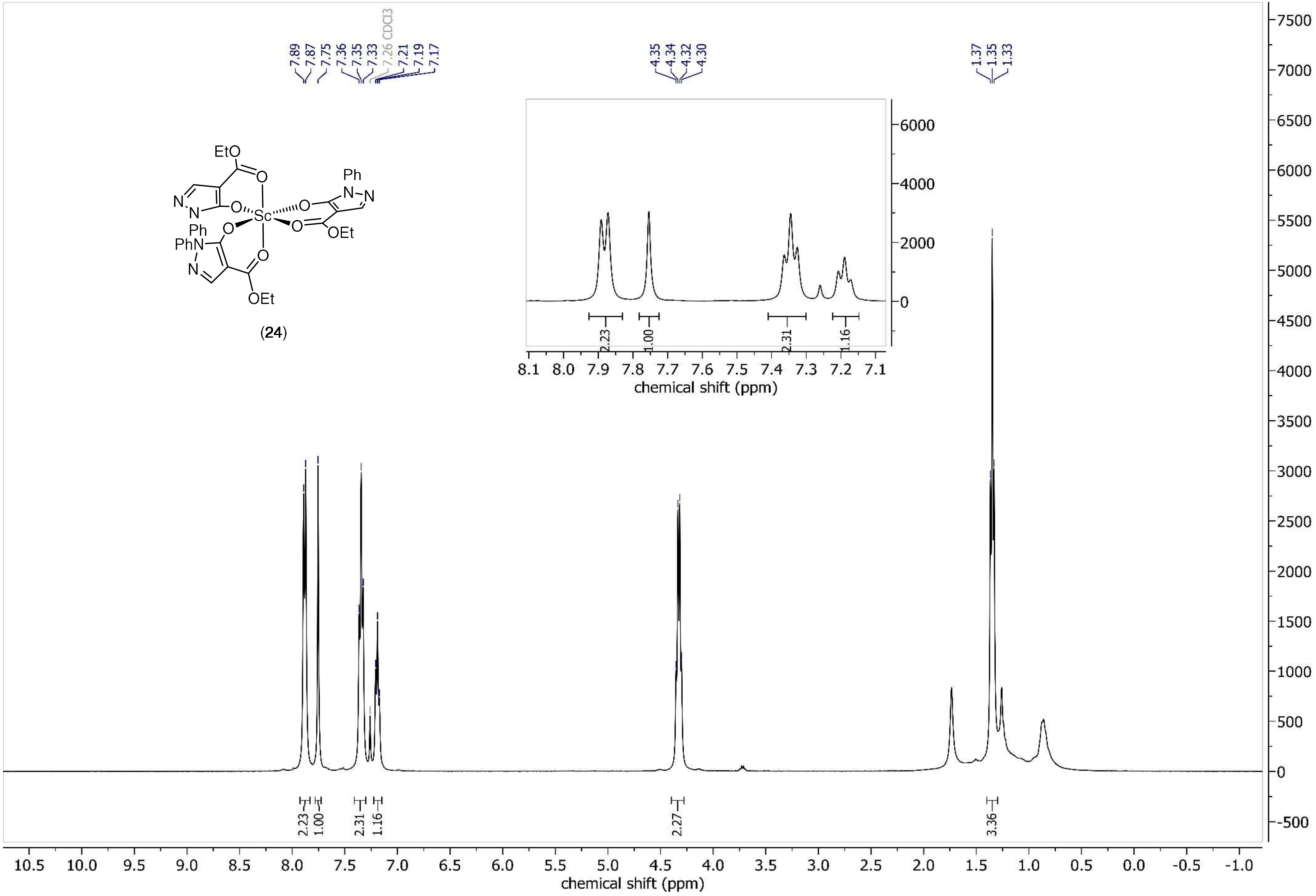

Figure S45: ^{1}H-NMR spectrum of tris((4-(ethoxycarbonyl)-1-phenyl-1*H*-pyrazol-5-yl)oxy)scandium (**24**).

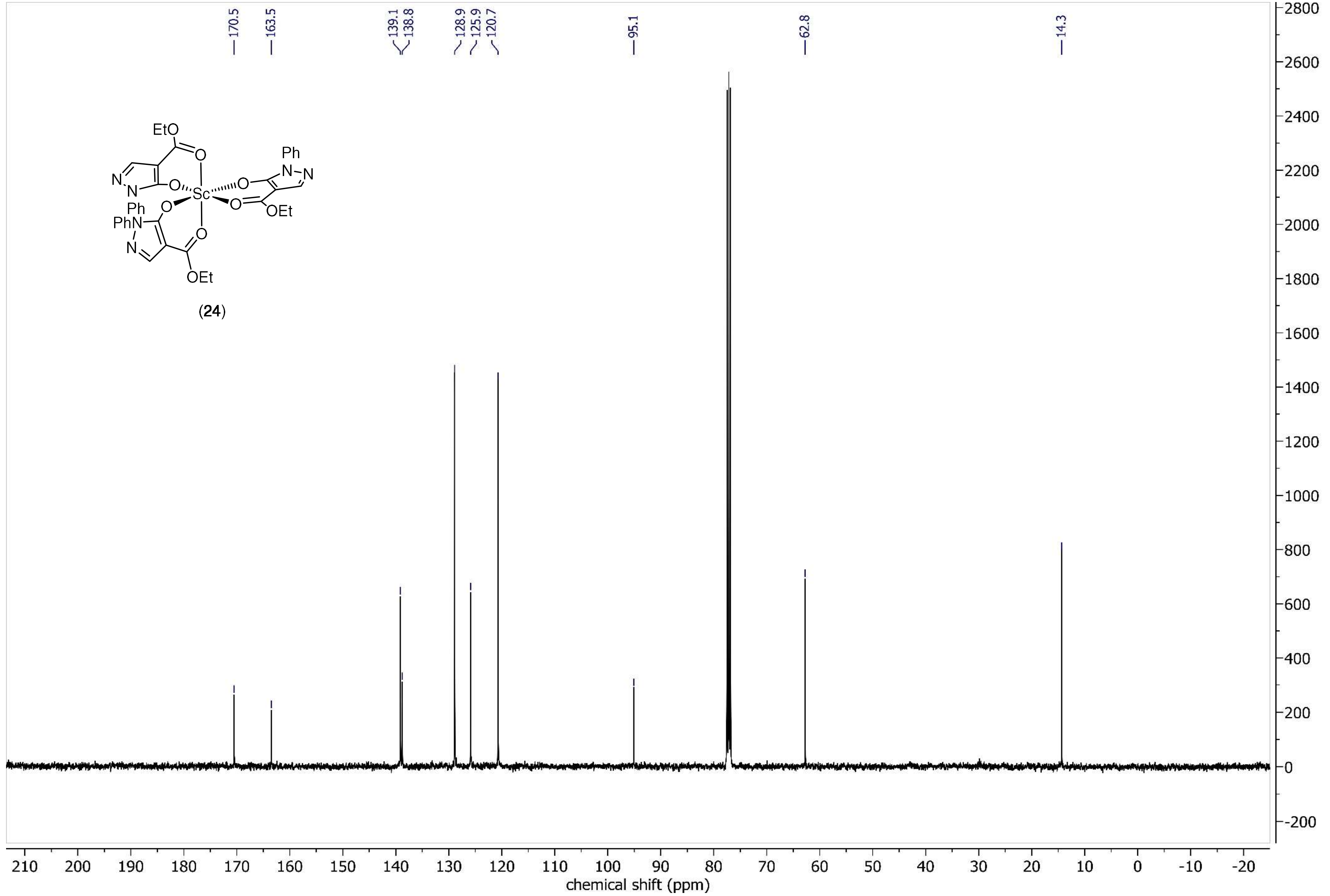

Figure S46: $^{13}C\{^{1}H\}$-NMR spectrum of tris((4-(ethoxycarbonyl)-1-phenyl-1*H*-pyrazol-5-yl)oxy)scandium (**24**).

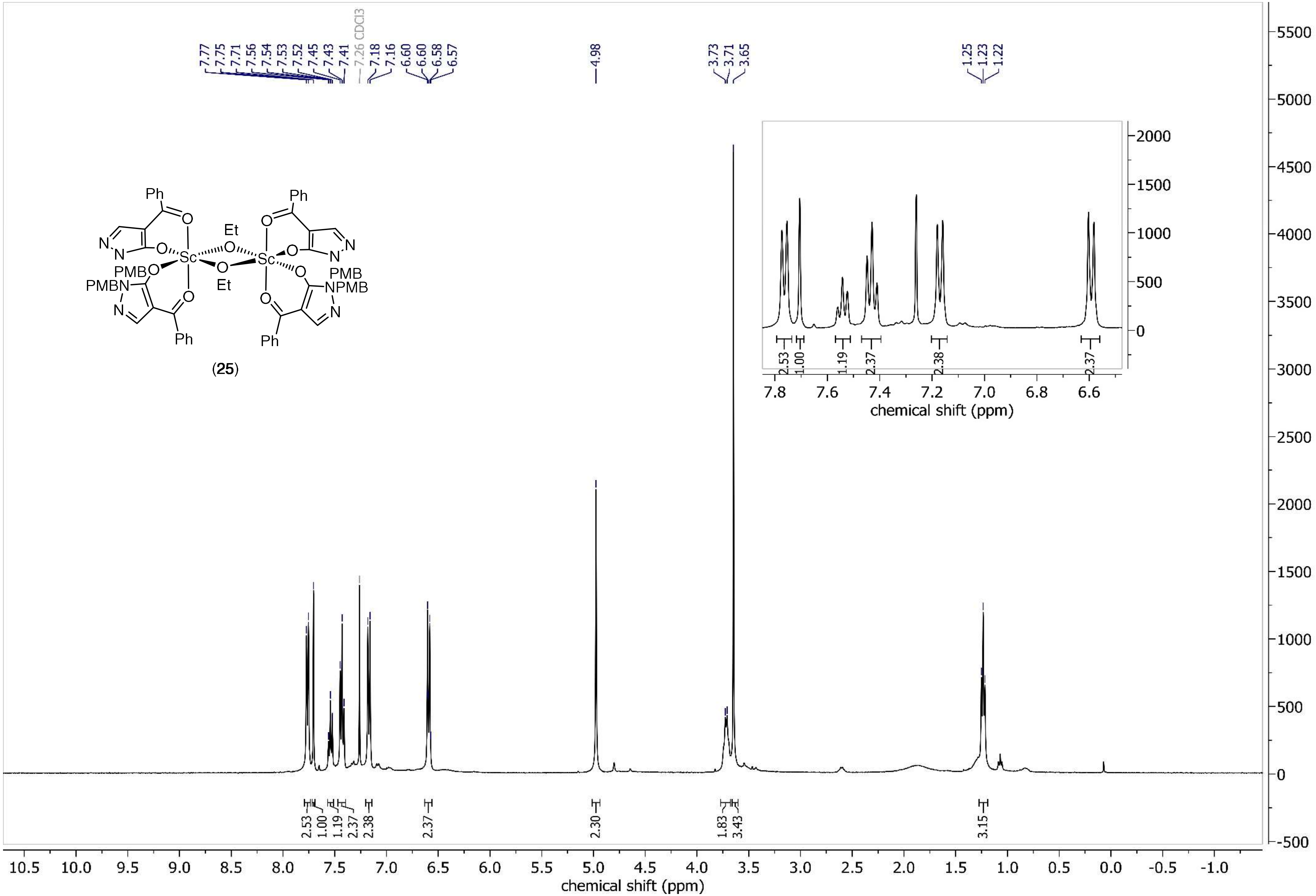

Figure S47: ^{1}H-NMR spectrum of tris((4-benzoyl-1-(4-methoxybenzyl)-1*H*-pyrazol-5-yl)oxy)scandium ethanol dimer (**25**).

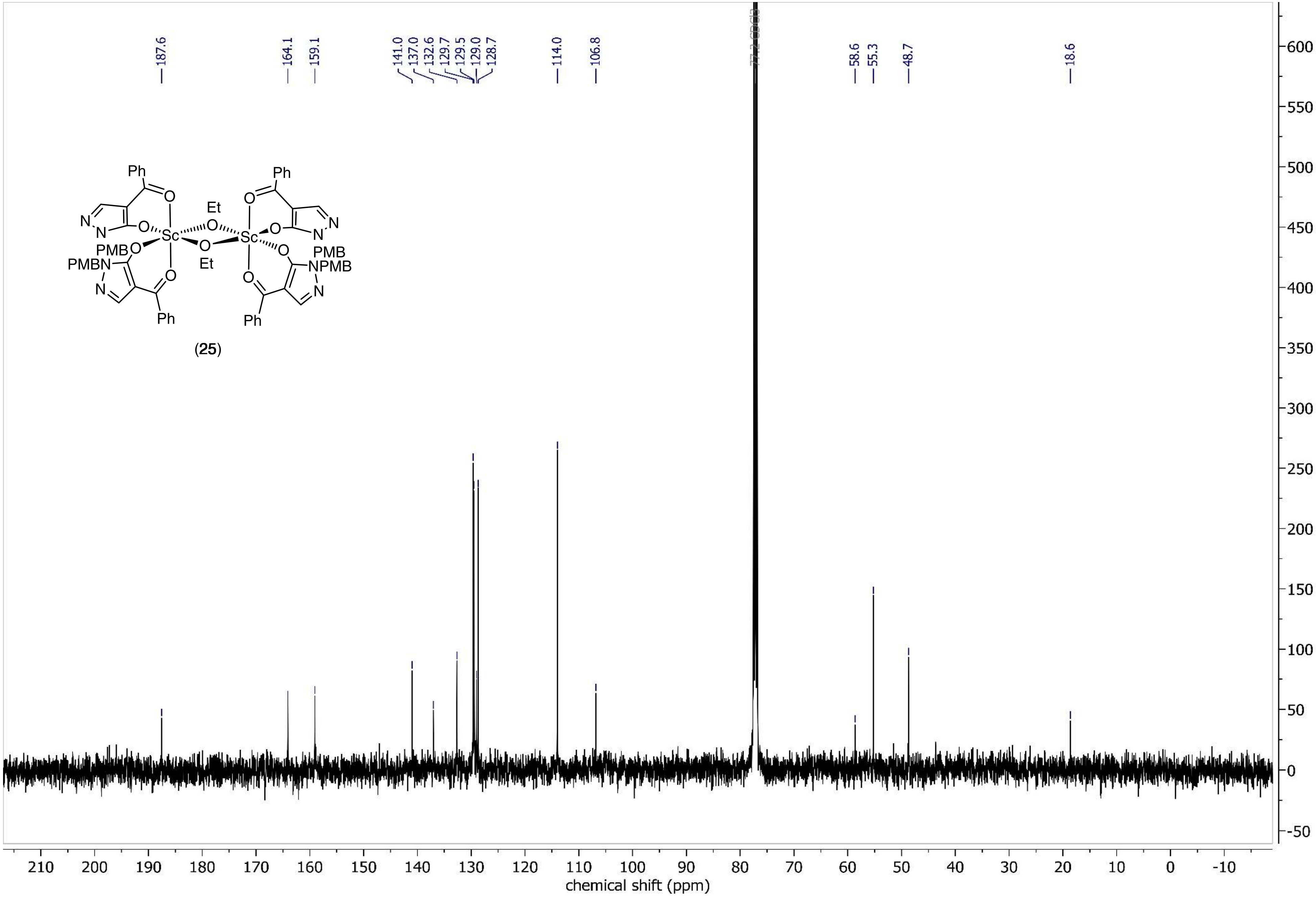

Figure S48: $^{13}C\{^{1}H\}$-NMR spectrum of tris((4-benzoyl-1-(4-methoxybenzyl)-1*H*-pyrazol-5-yl)oxy)scandium ethanol dimer (**25**).

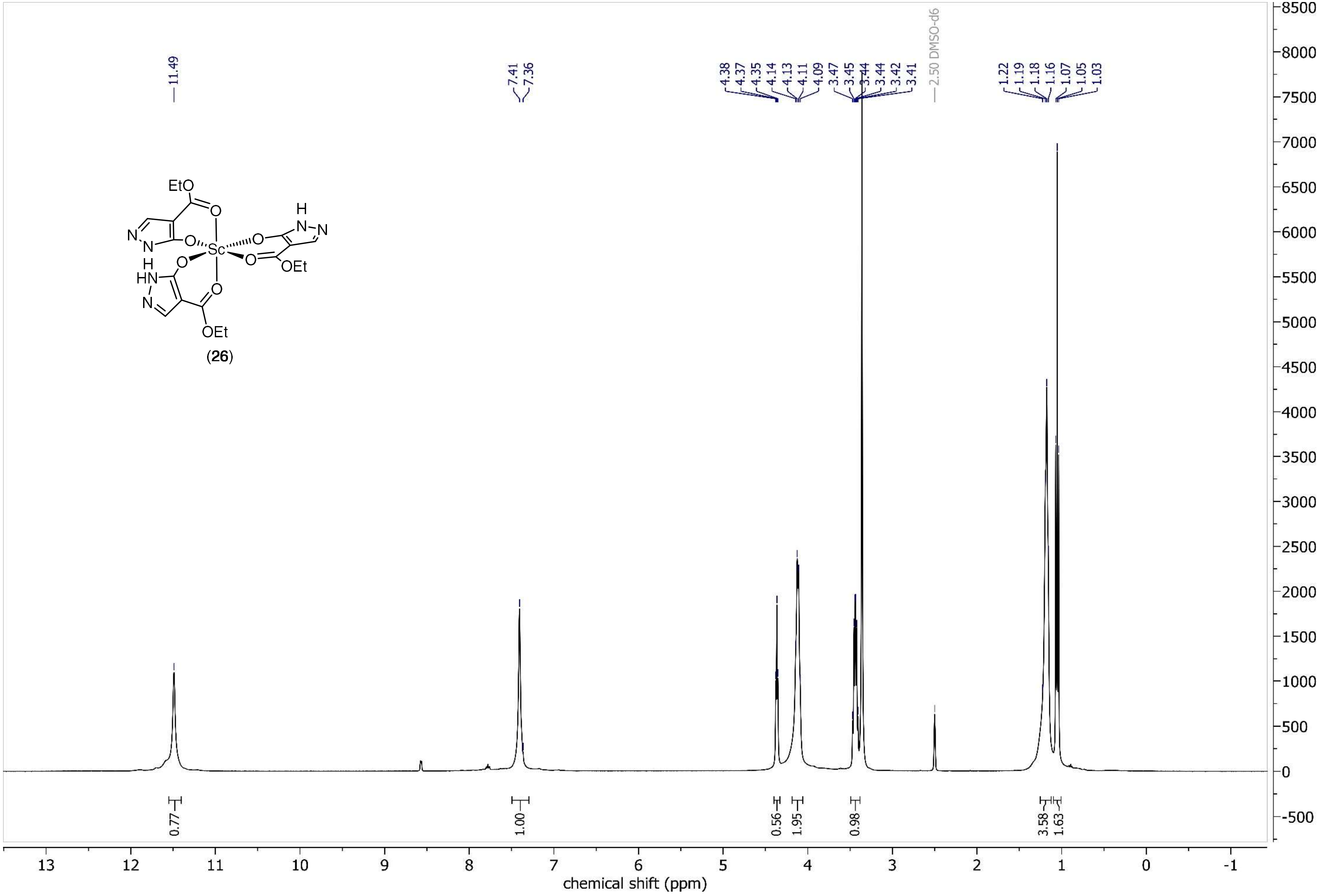

Figure S49: ^{1}H-NMR spectrum of tris((4-(ethoxycarbonyl)-1*H*-pyrazol-5-yl)oxy)scandium (**26**).

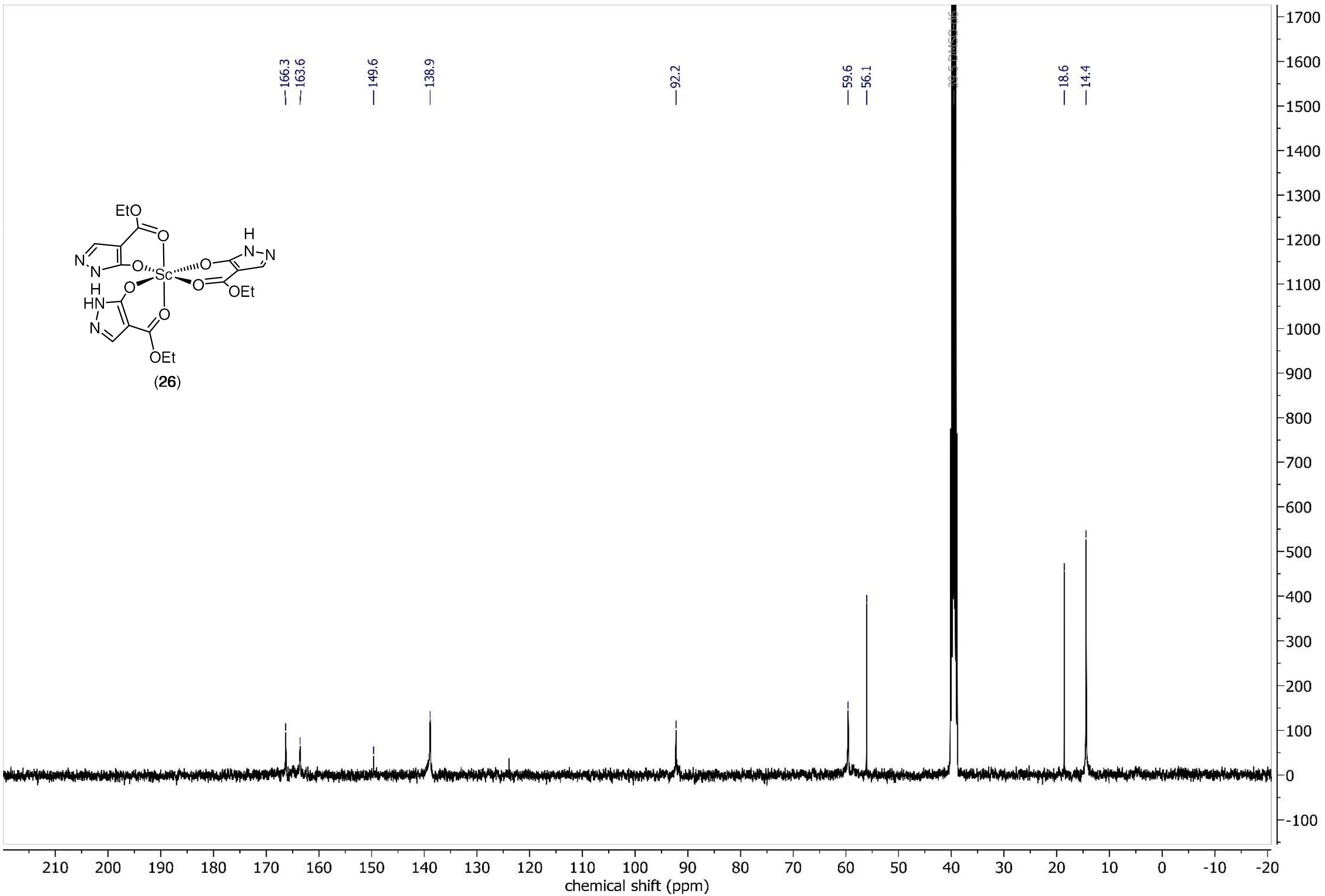

(**26**)

Figure S50: $^{13}C\{^{1}H\}$-NMR spectrum of tris((4-(ethoxycarbonyl)-1*H*-pyrazol-5-yl)oxy)scandium (**26**).

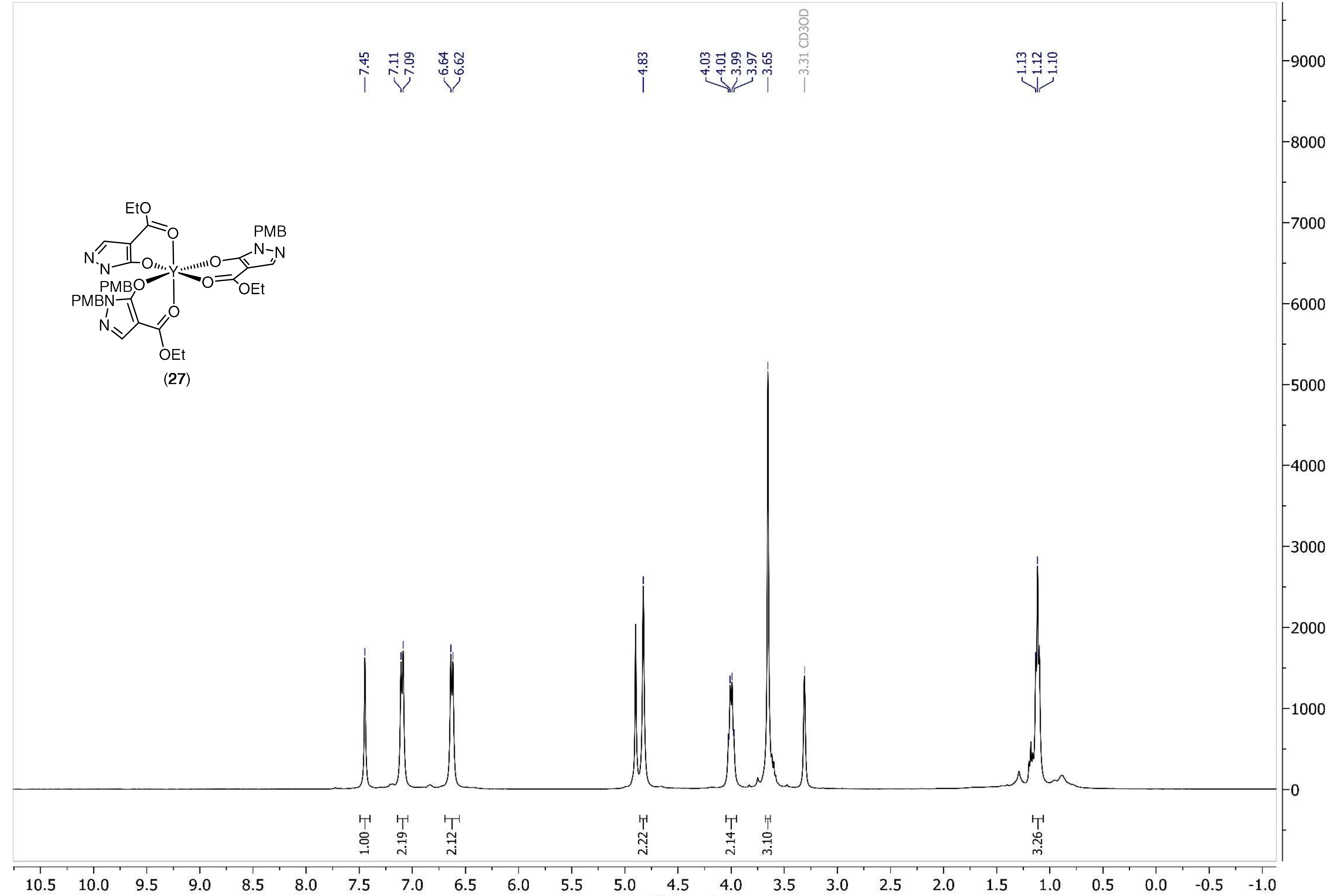

Figure S51: ^{1}H-NMR spectrum of tris((4-(ethoxycarbonyl)-1-(4-methoxybenzyl)-1*H*-pyrazol-5-yl)oxy)yttrium (**27**).

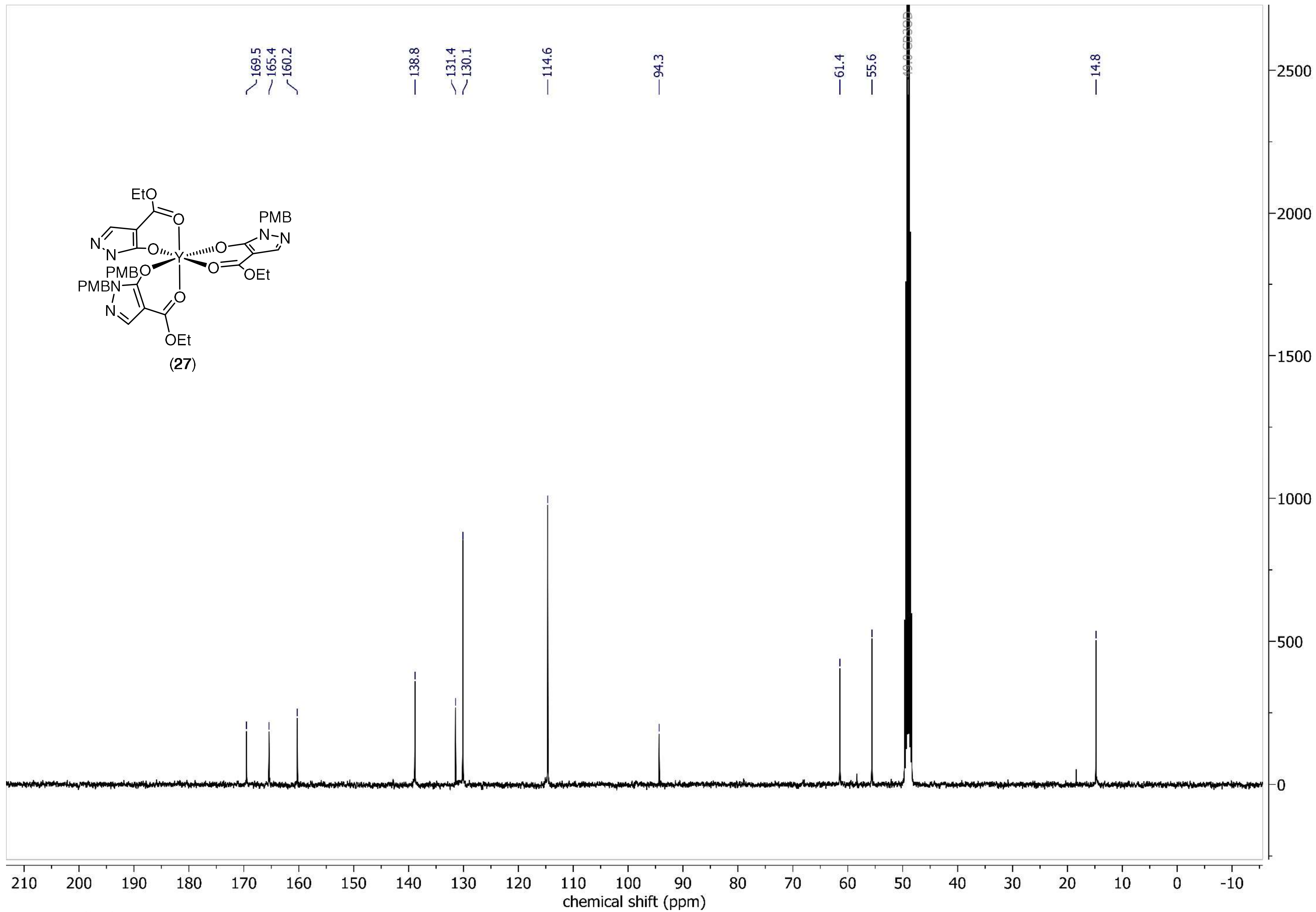

Figure S52: $^{13}C\{^1H\}$-NMR spectrum of tris((4-(ethoxycarbonyl)-1-(4-methoxybenzyl)-1*H*-pyrazol-5-yl)oxy)yttrium (**27**).

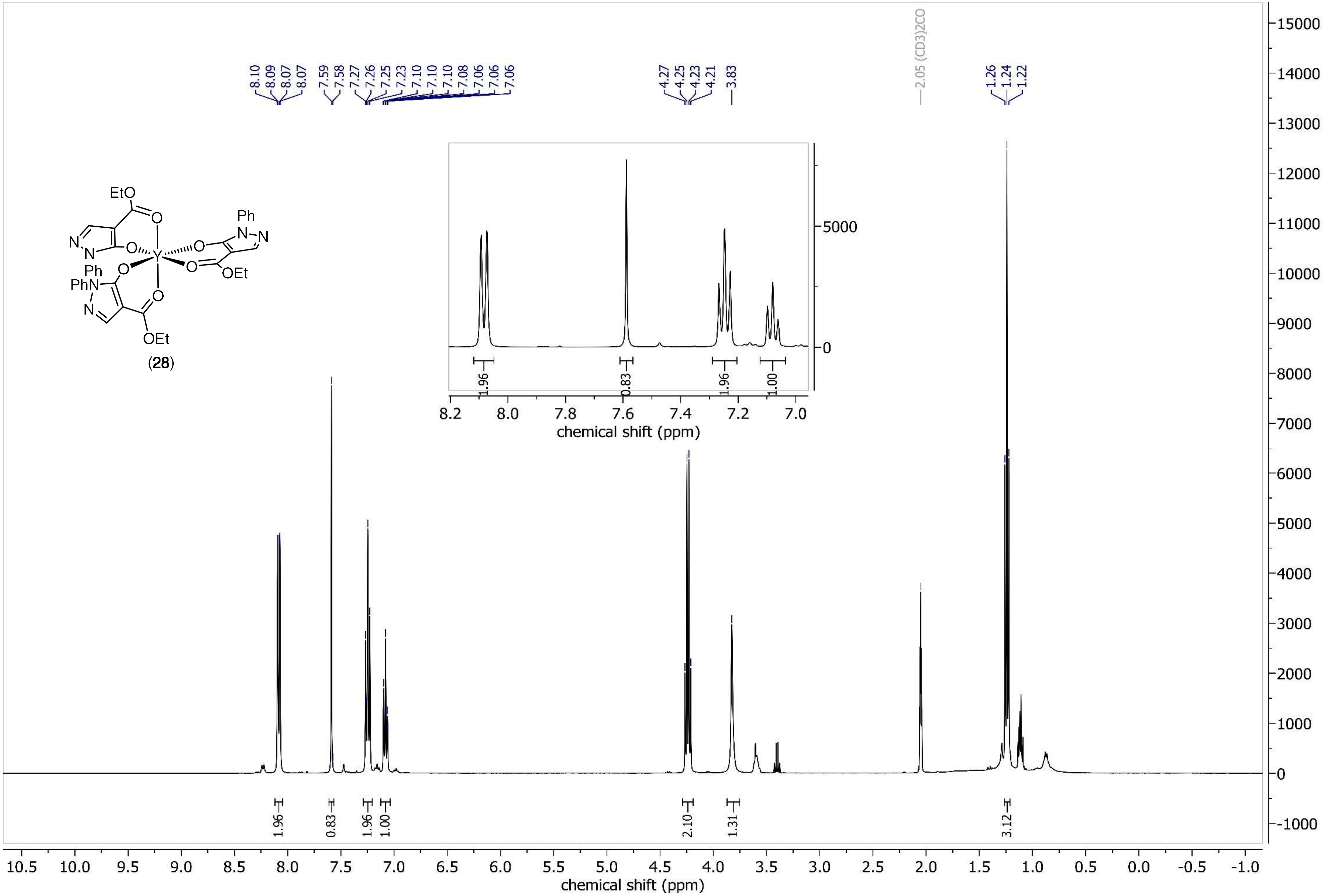

Figure S53: ^{1}H-NMR spectrum of tris((4-(ethoxycarbonyl)-1-phenyl-1H-pyrazol-5-yl)oxy)yttrium (**28**).

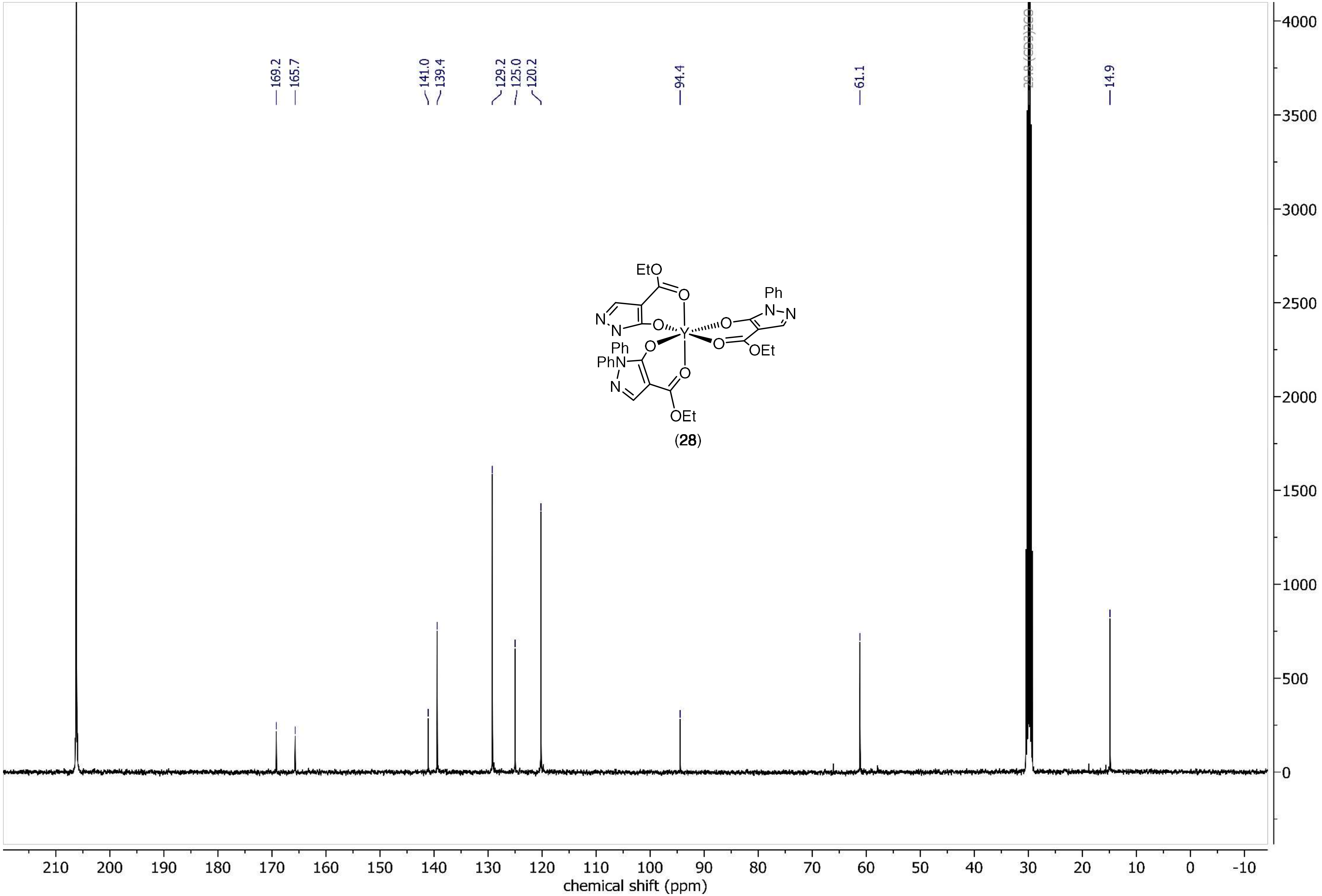

Figure S54: $^{13}C\{^{1}H\}$-NMR spectrum of tris((4-(ethoxycarbonyl)-1-phenyl-1H-pyrazol-5-yl)oxy)yttrium (**28**).

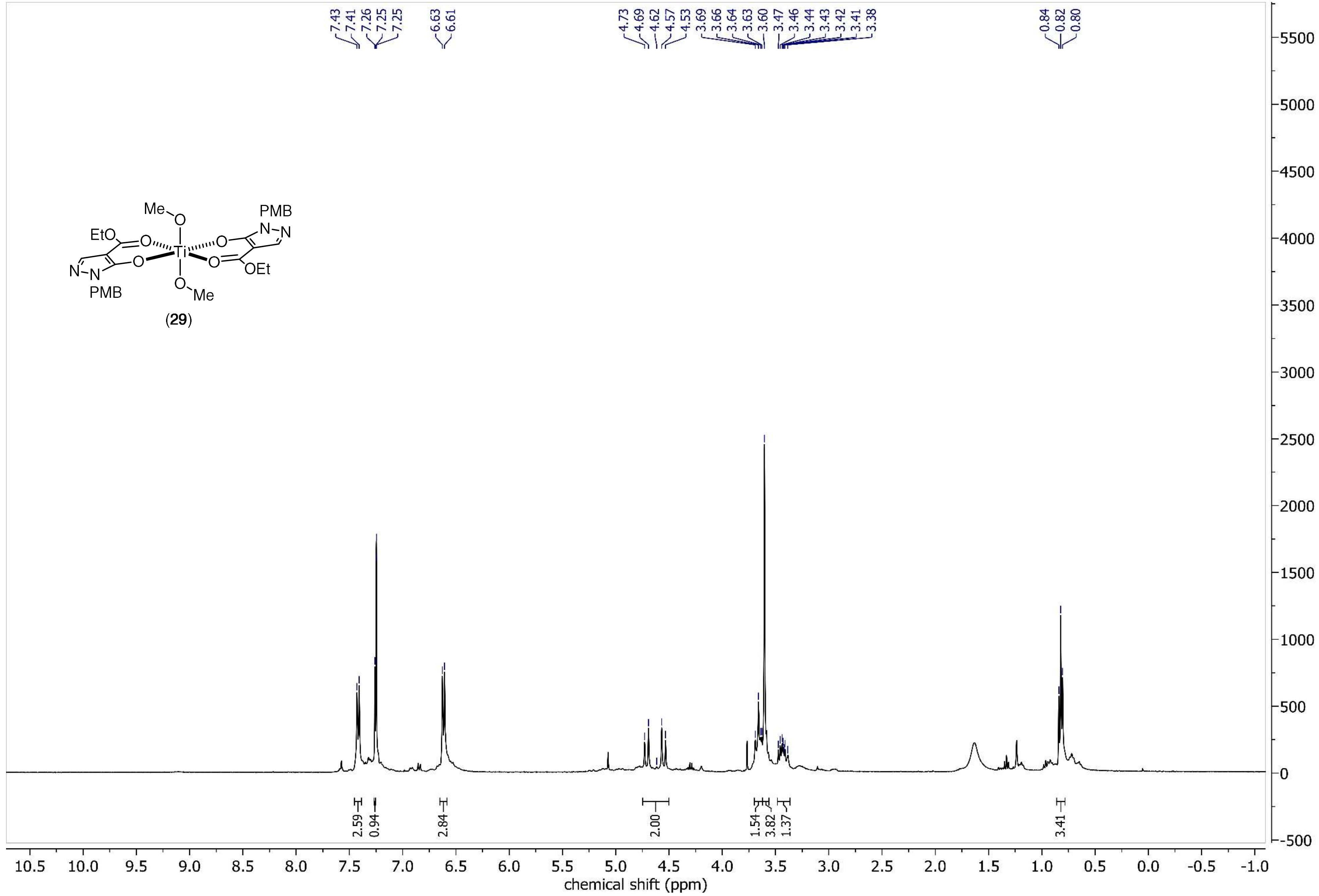

Figure S55: ^{1}H-NMR spectrum of bis(methoxy) bis(4-(ethoxycarbonyl)-1-(4-methoxybenzyl)-1*H*-pyrazol-5-olate)titanium (**29**).

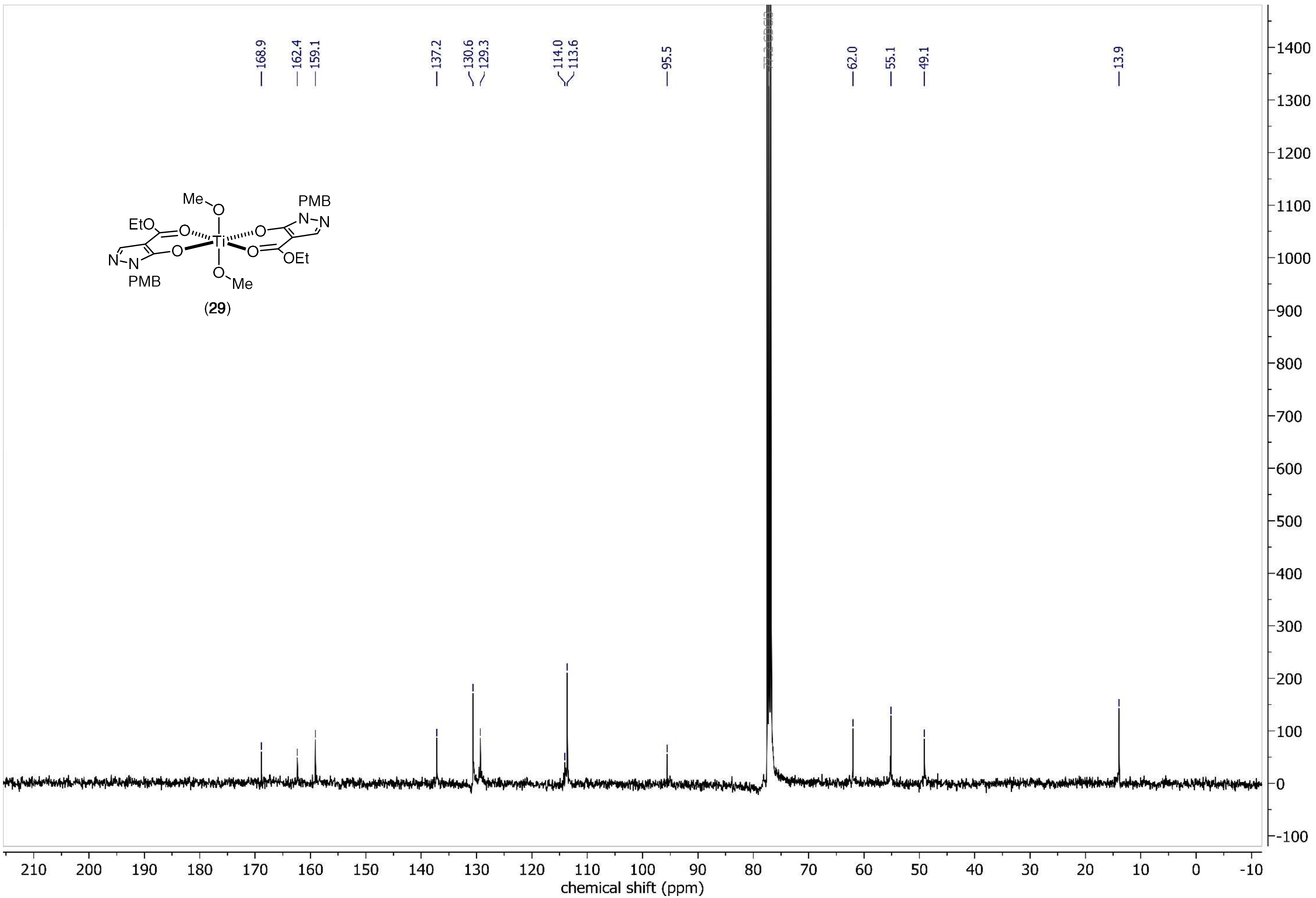

Figure S56: $^{13}C\{^{1}H\}$-NMR spectrum of bis(methoxy) bis(4-(ethoxycarbonyl)-1-(4-methoxybenzyl)-1*H*-pyrazol-5-olate)titanium (**29**).

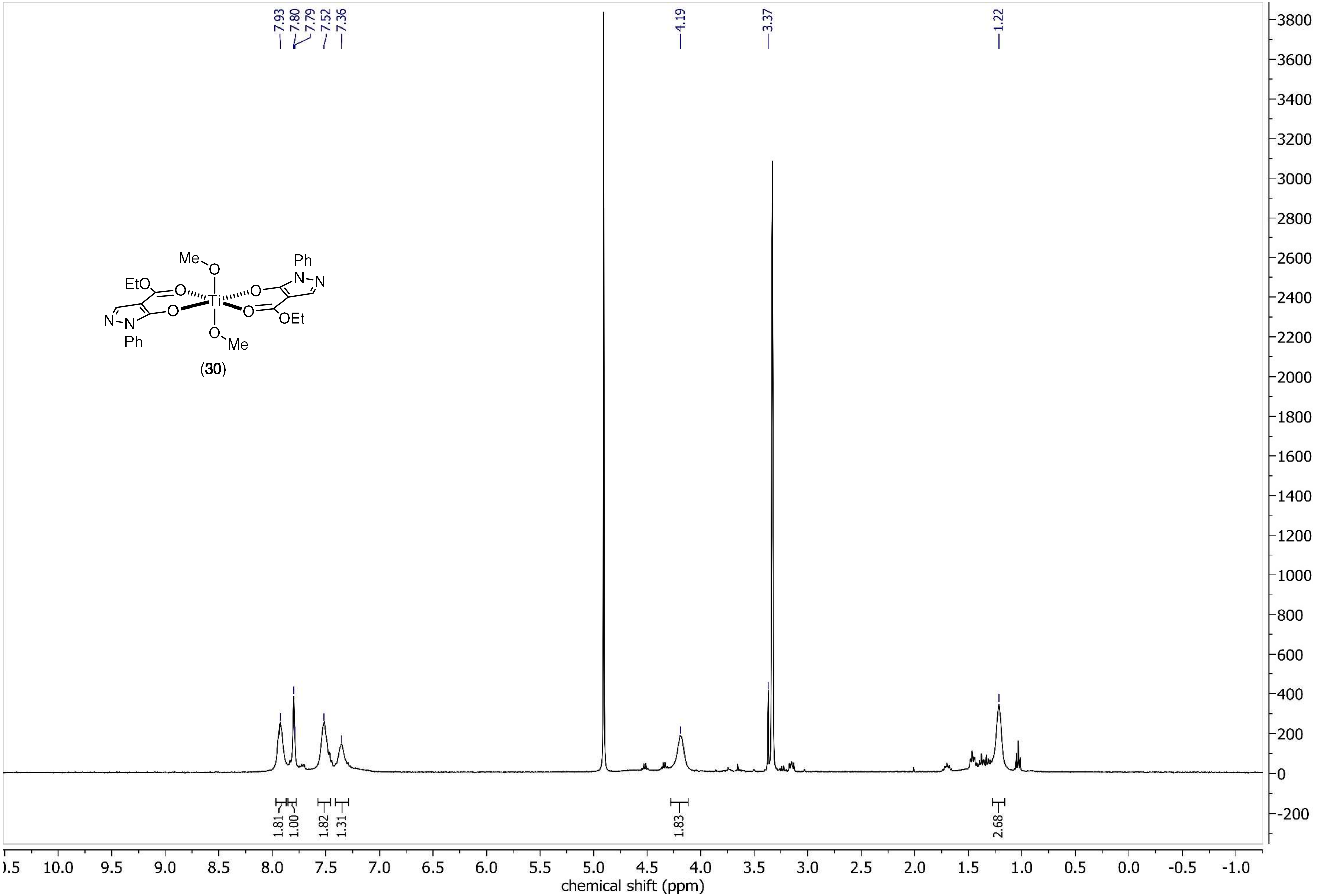

Figure S57: ^{1}H-NMR spectrum of bis(methoxy) bis(4-(ethoxycarbonyl)-1-phenyl-1*H*-pyrazol-5-olate)titanium (**30**).

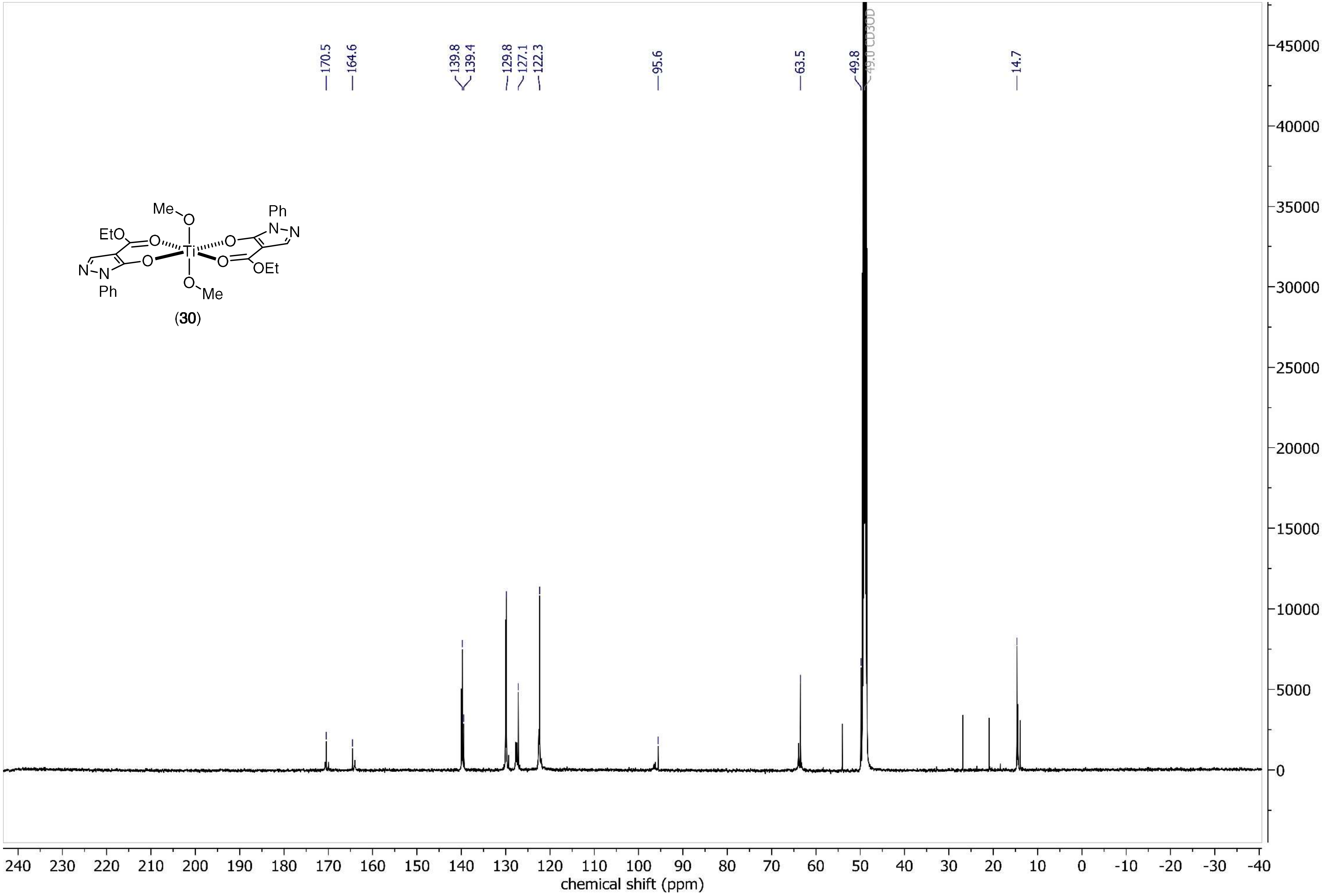

Figure S58: $^{13}C\{^1H\}$-NMR spectrum of bis(methoxy) bis(4-(ethoxycarbonyl)-1-phenyl-1*H*-pyrazol-5-olate)titanium (**30**).

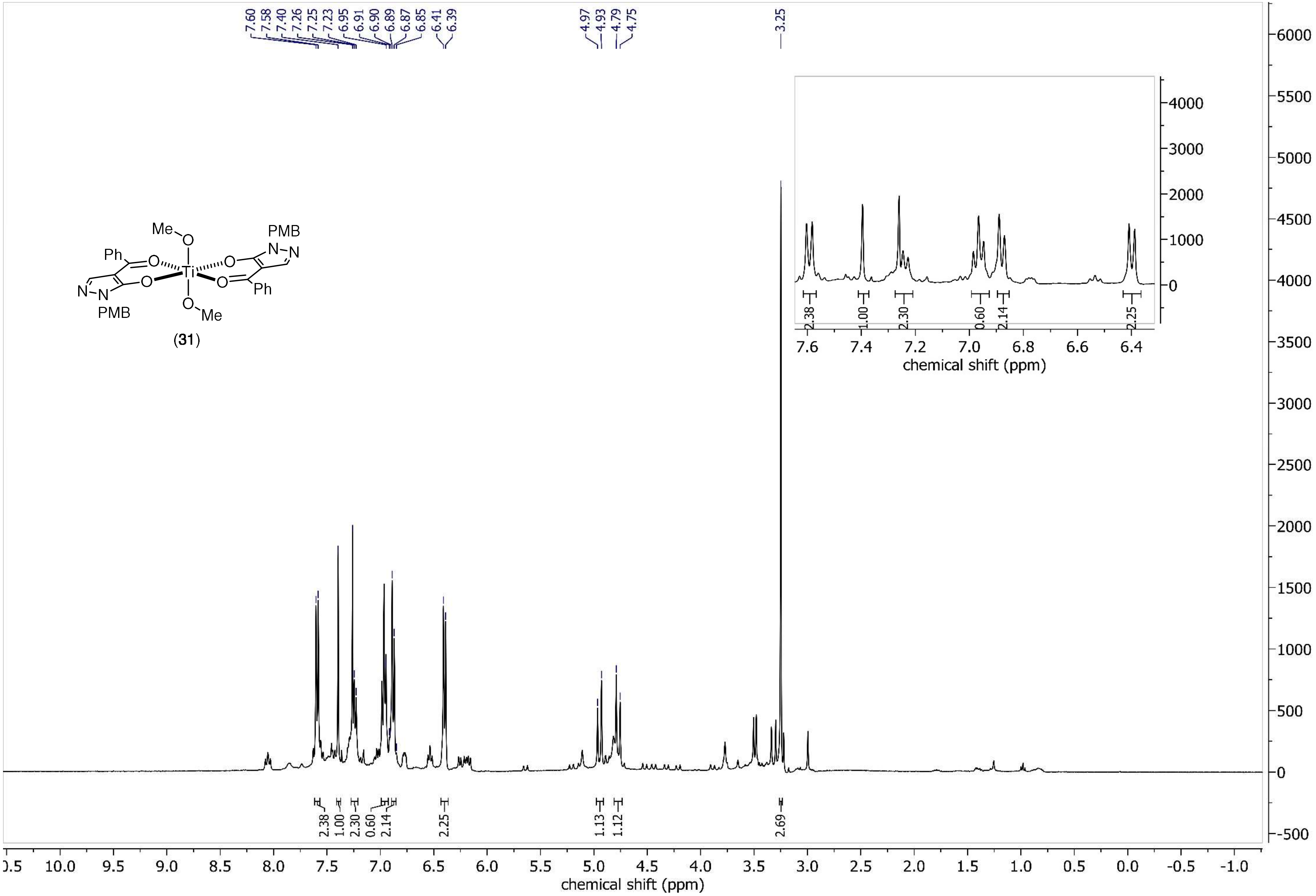

Figure S59: ^{1}H-NMR spectrum of bis(methoxy) bis(4-benzoyl-1-(4-methoxybenzyl)-1*H*-pyrazol-5-olate)titanium (**31**).

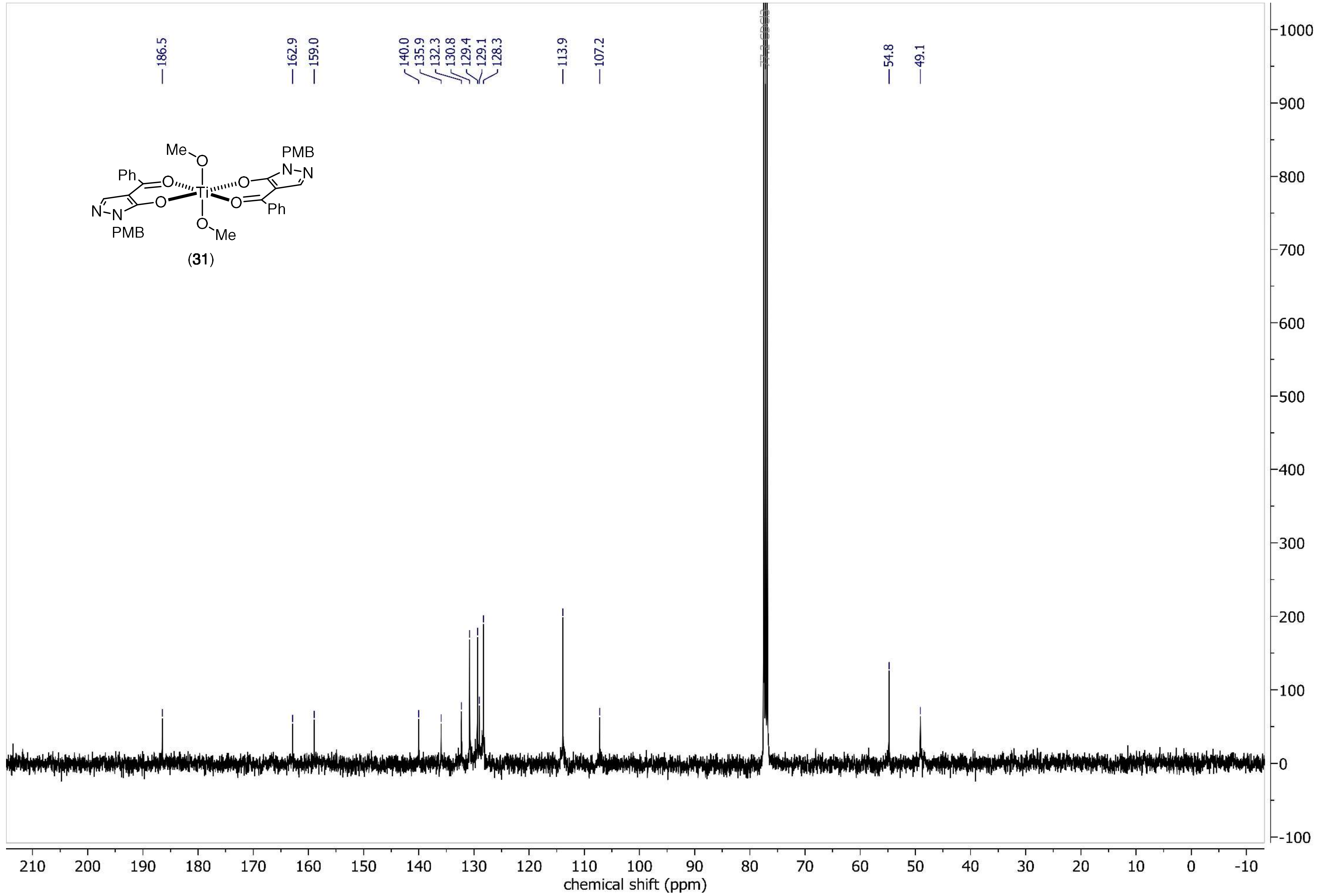

Figure S60: $^{13}C\{^1H\}$-NMR spectrum of bis(methoxy) bis(4-benzoyl-1-(4-methoxybenzyl)-1*H*-pyrazol-5-olate)titanium (**31**).

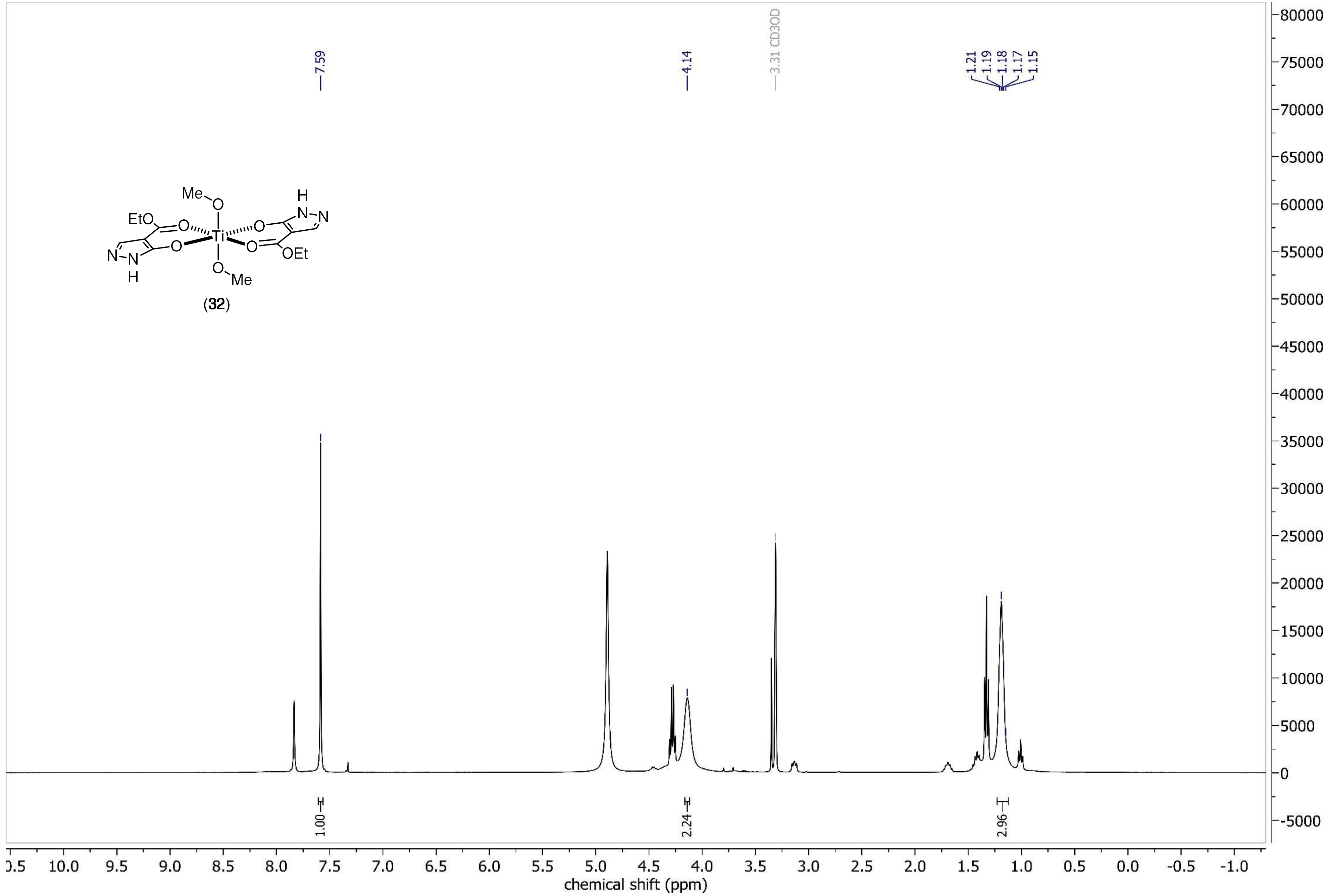

Figure S61: ^{1}H-NMR spectrum of bis(methoxy) bis(4-(ethoxycarbonyl)-1*H*-pyrazol-5-olate)titanium (**32**).

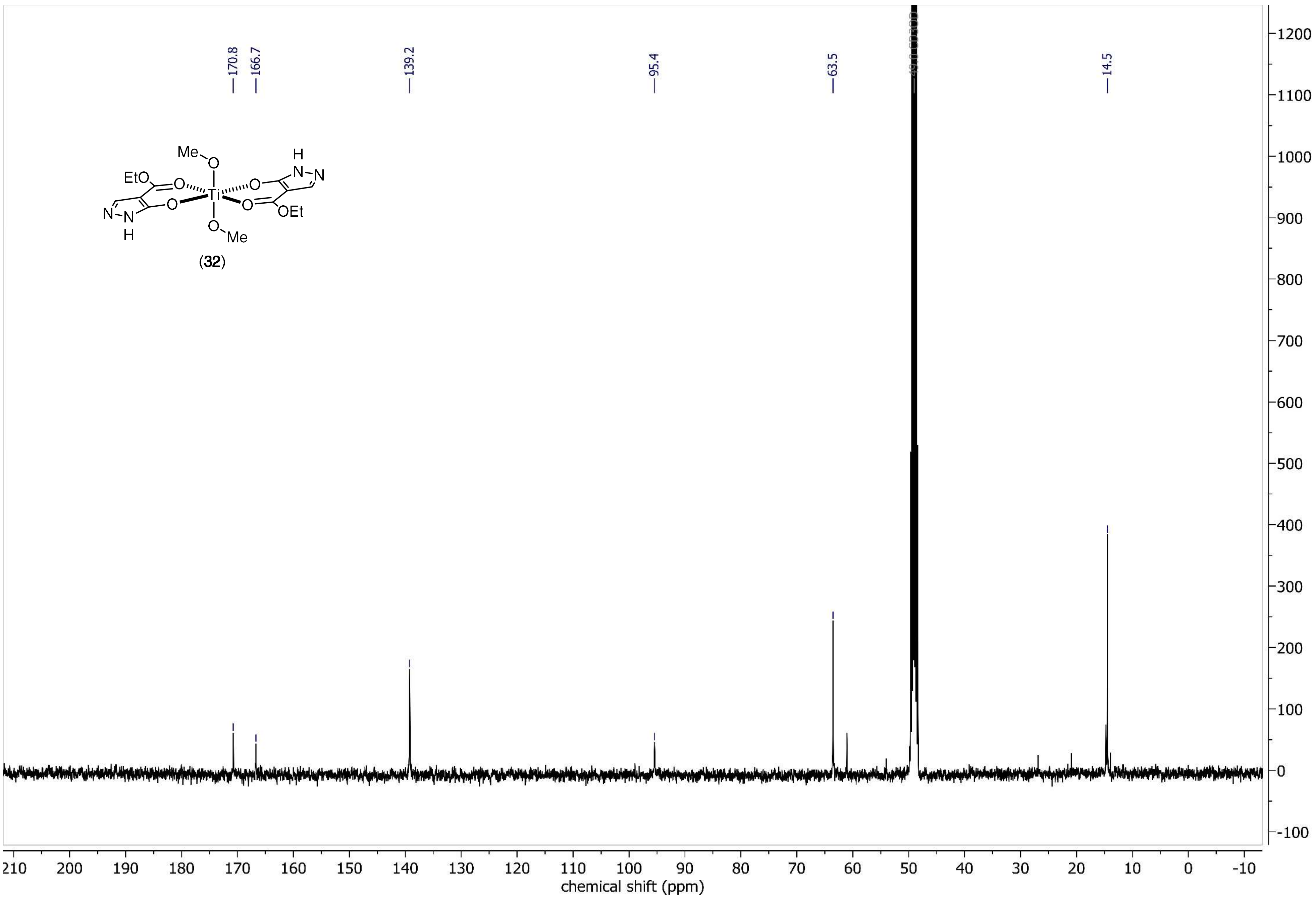

Figure S62: $^{13}C\{^{1}H\}$-NMR spectrum of bis(methoxy) bis(4-(ethoxycarbonyl)-1*H*-pyrazol-5-olate)titanium (**32**).

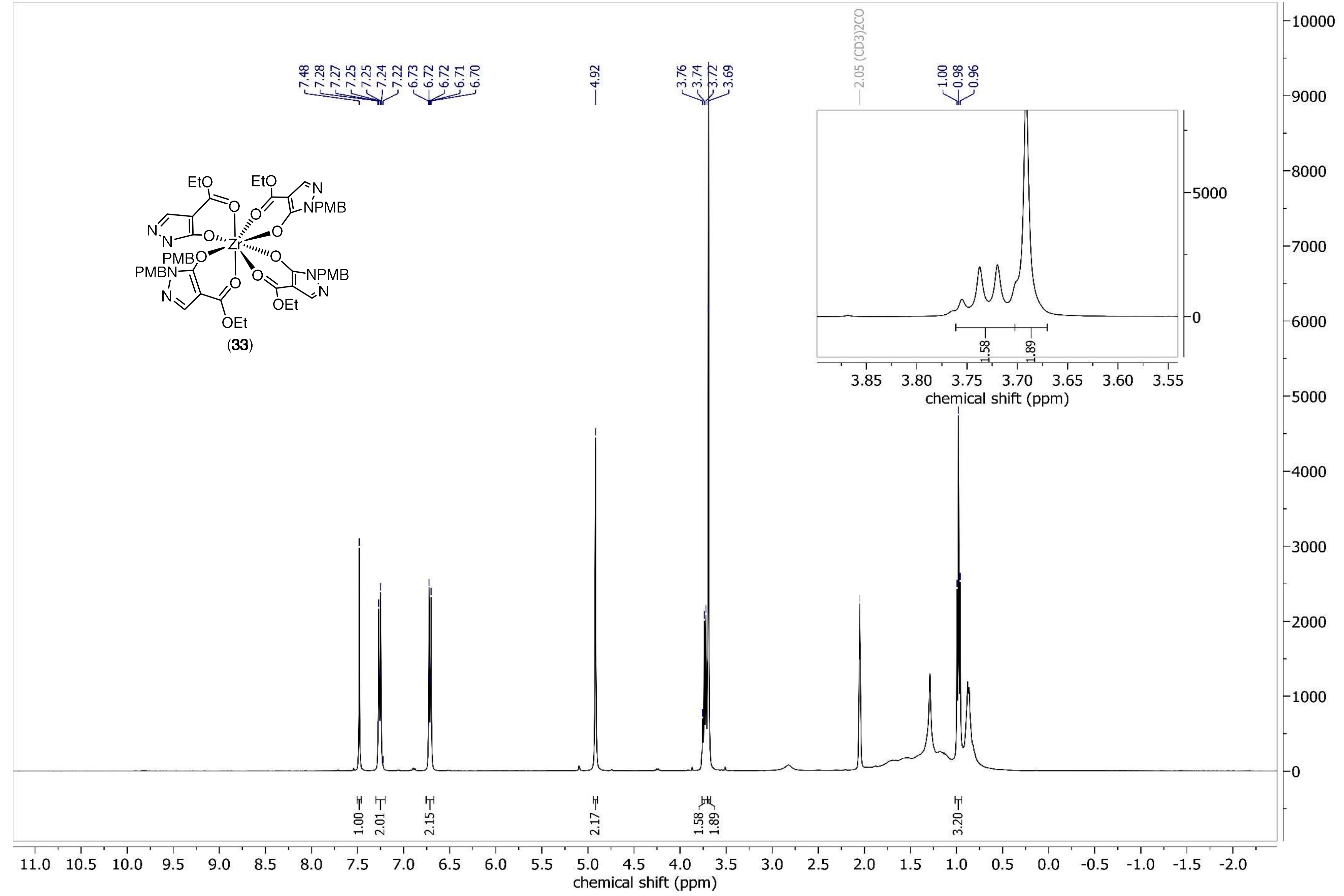

Figure S63: ^{1}H-NMR spectrum of tetrakis((4-(ethoxycarbonyl)-1-(4-methoxybenzyl)-1*H*-pyrazol-5-yl)oxy)zirconium (**33**) in acetone-d_6.

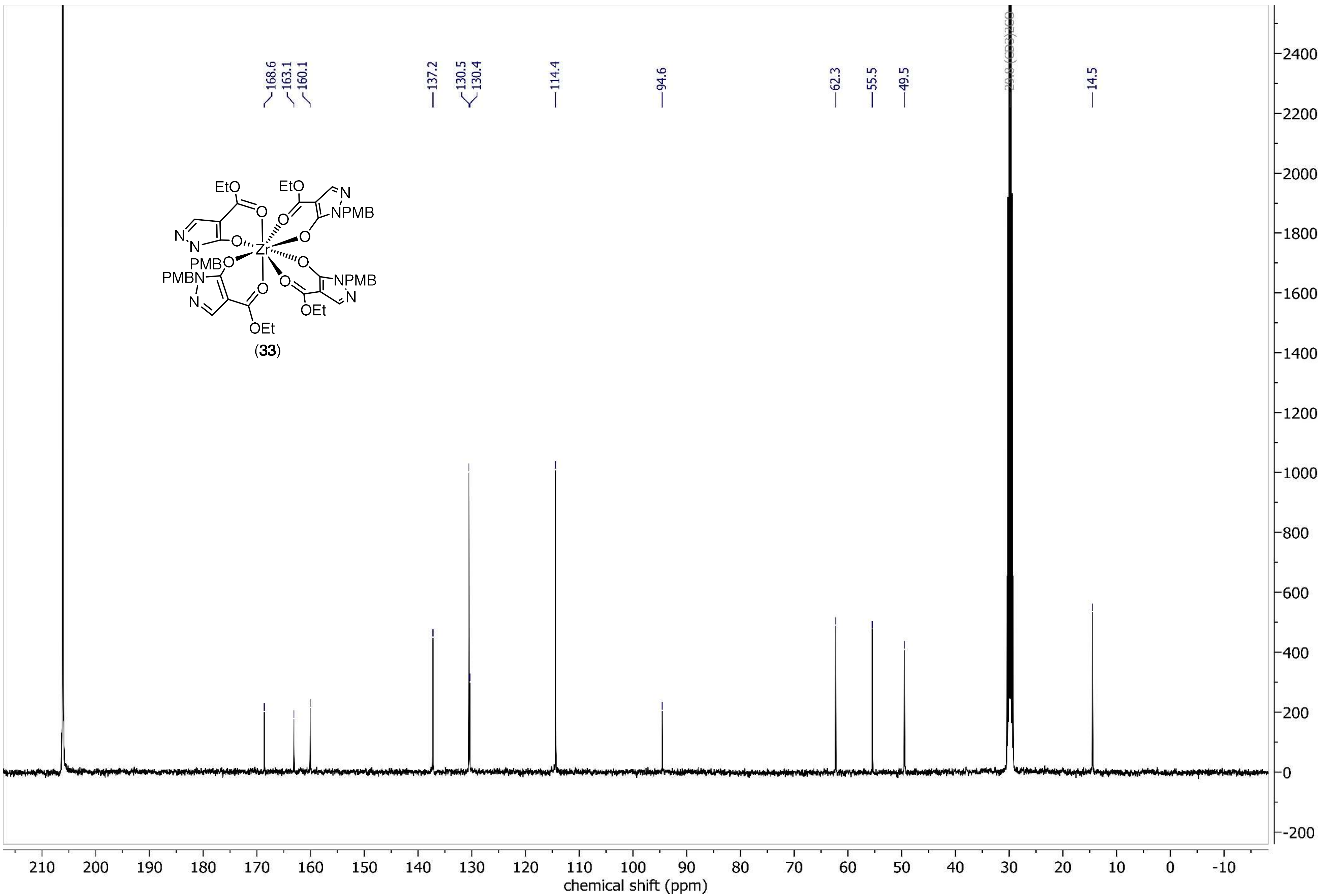

Figure S64: $^{13}C\{^{1}H\}$-NMR spectrum of tetrakis((4-(ethoxycarbonyl)-1-(4-methoxybenzyl)-1*H*-pyrazol-5-yl)oxy)zirconium (**33**) in acetone-d_6.

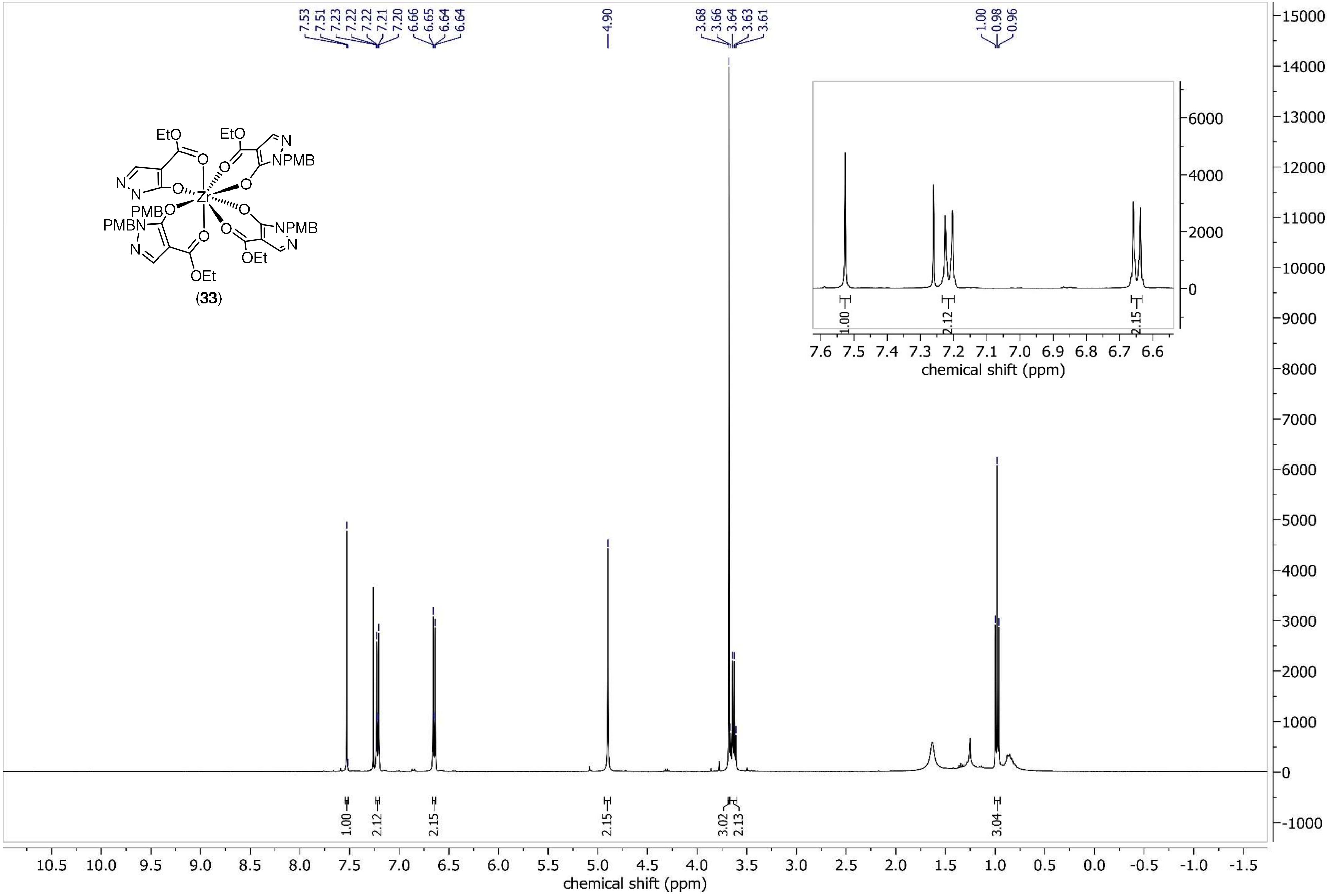

Figure S65: ^{1}H-NMR spectrum of tetrakis((4-(ethoxycarbonyl)-1-(4-methoxybenzyl)-1*H*-pyrazol-5-yl)oxy)zirconium (**33**) in $CDCl_3$.

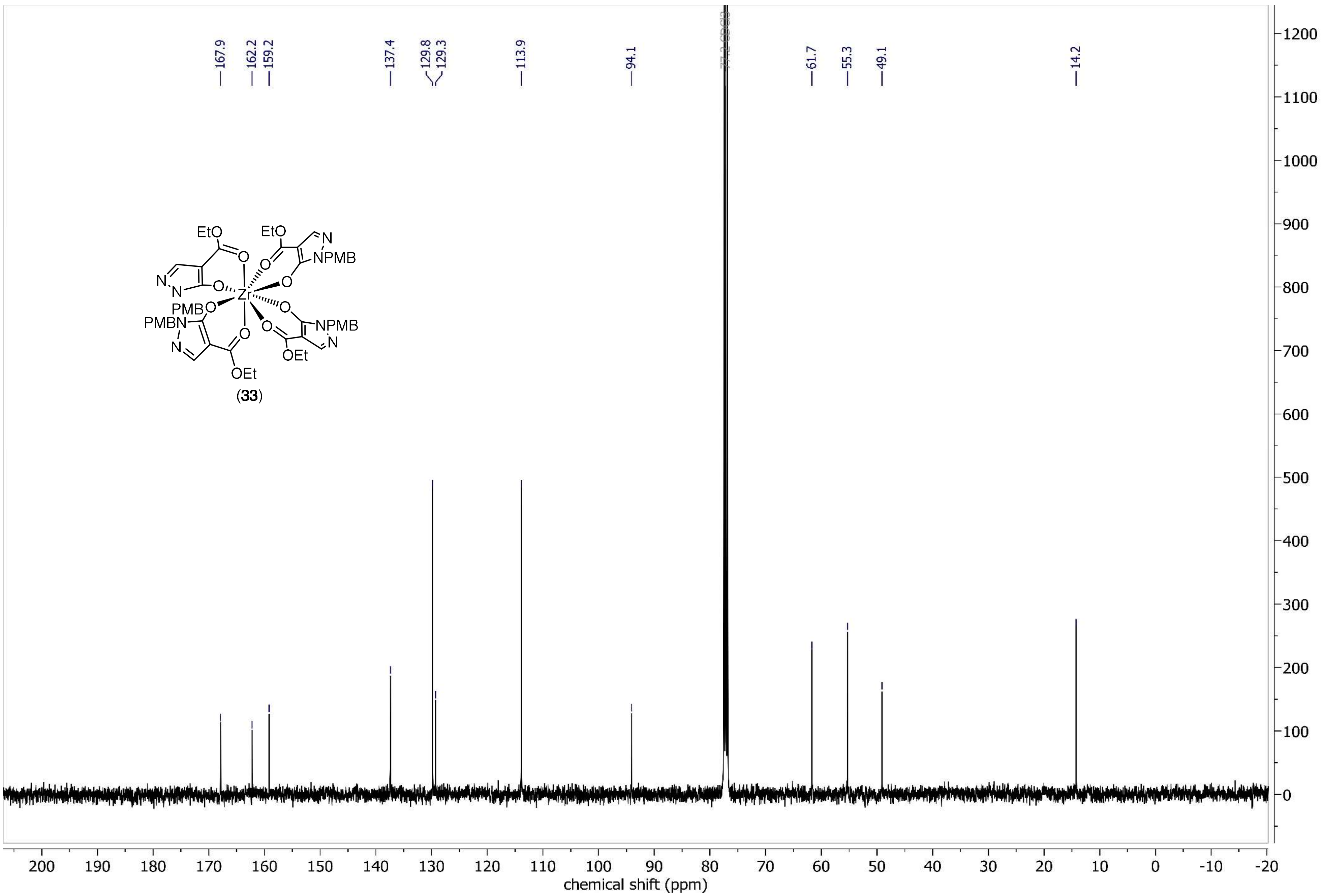

Figure S66: $^{13}C\{^1H\}$-NMR spectrum of tetrakis((4-(ethoxycarbonyl)-1-(4-methoxybenzyl)-1*H*-pyrazol-5-yl)oxy)zirconium (**33**) in $CDCl_3$.

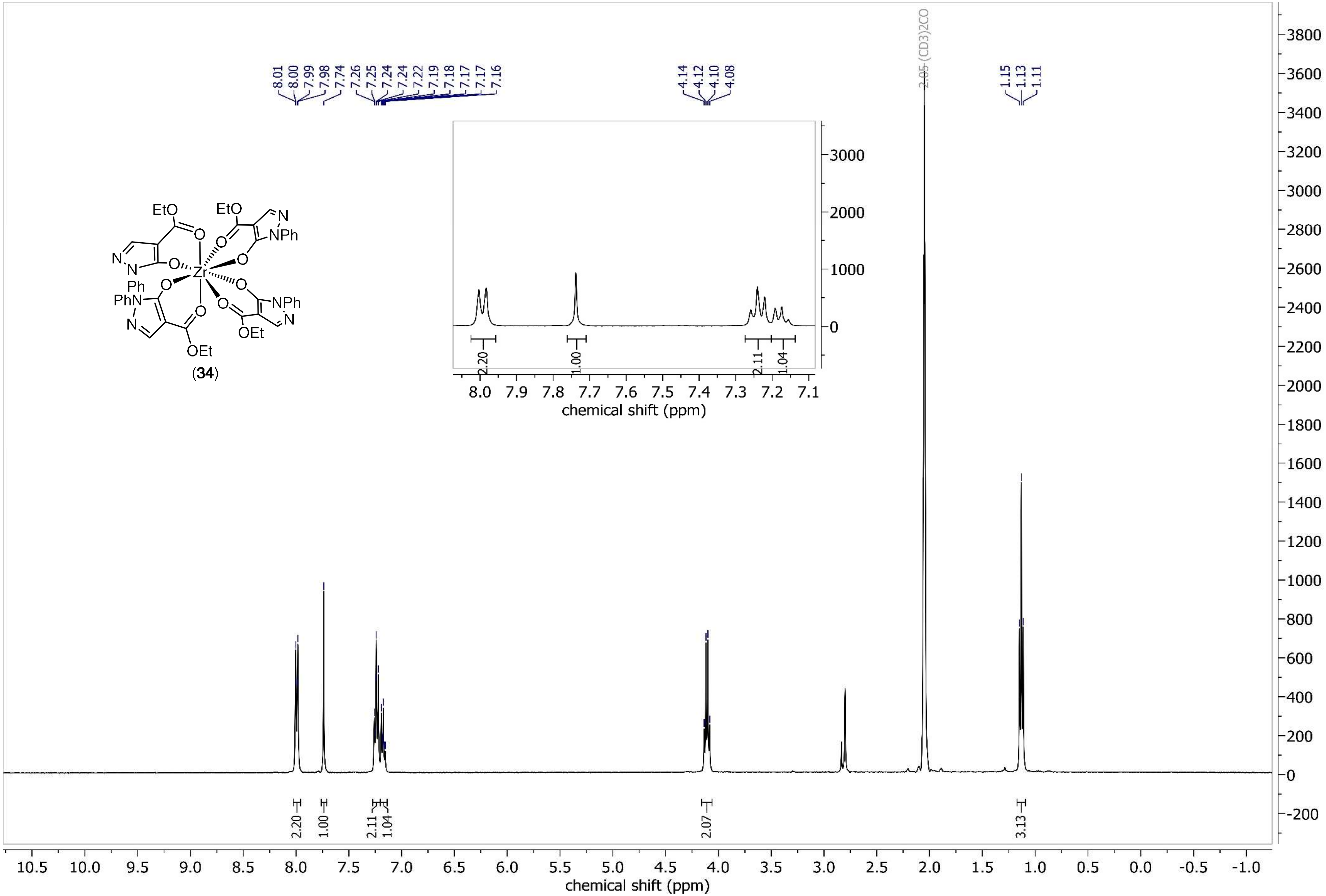

Figure S67: ^{1}H-NMR spectrum of tetrakis((4-(ethoxycarbonyl)-1-phenyl-1*H*-pyrazol-5-yl)oxy)zirconium (**34**) in acetone-d_6.

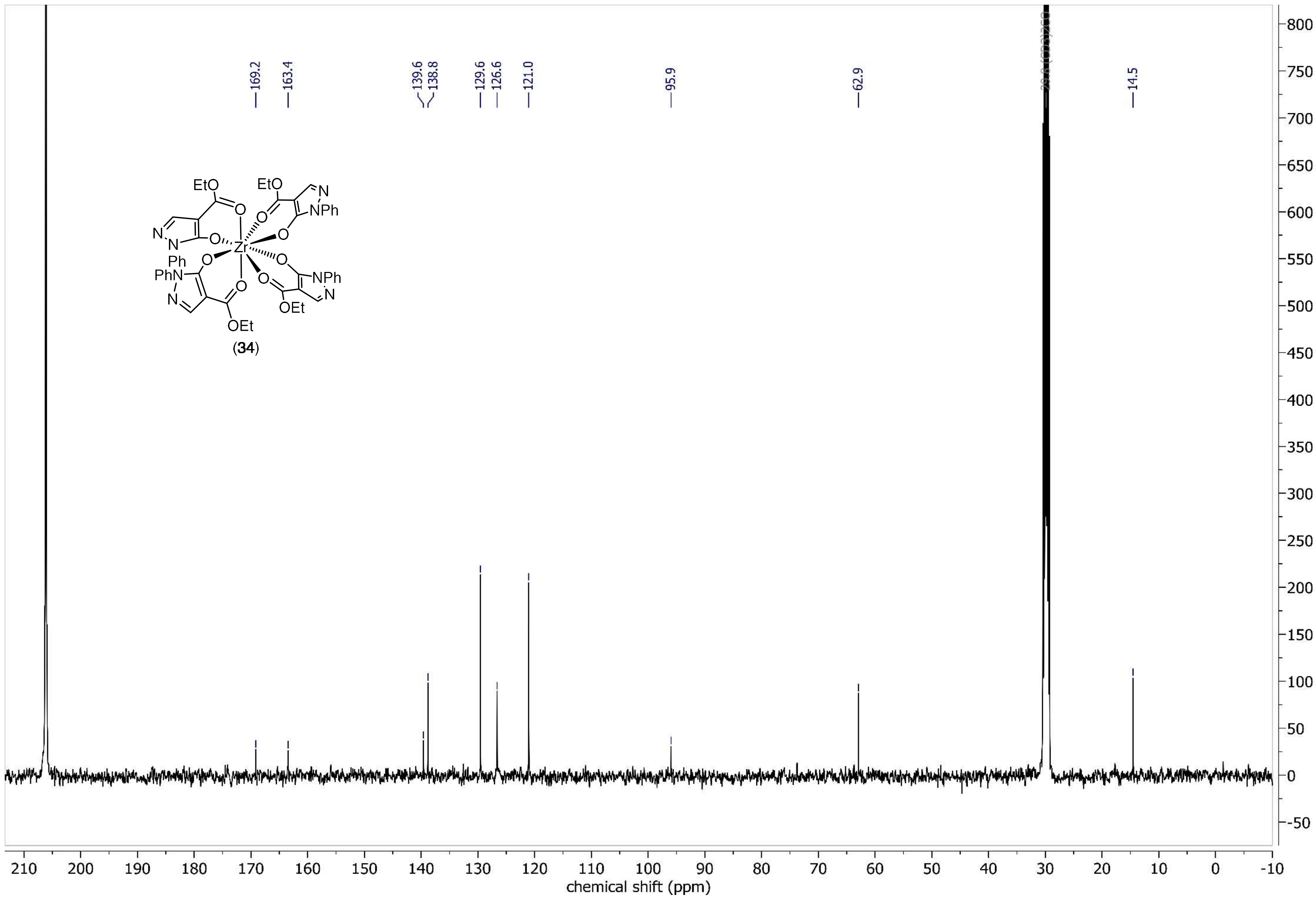

Figure S68: $^{13}C\{^{1}H\}$-NMR spectrum of tetrakis((4-(ethoxycarbonyl)-1-phenyl-1*H*-pyrazol-5-yl)oxy)zirconium (**34**) in acetone-d_6.

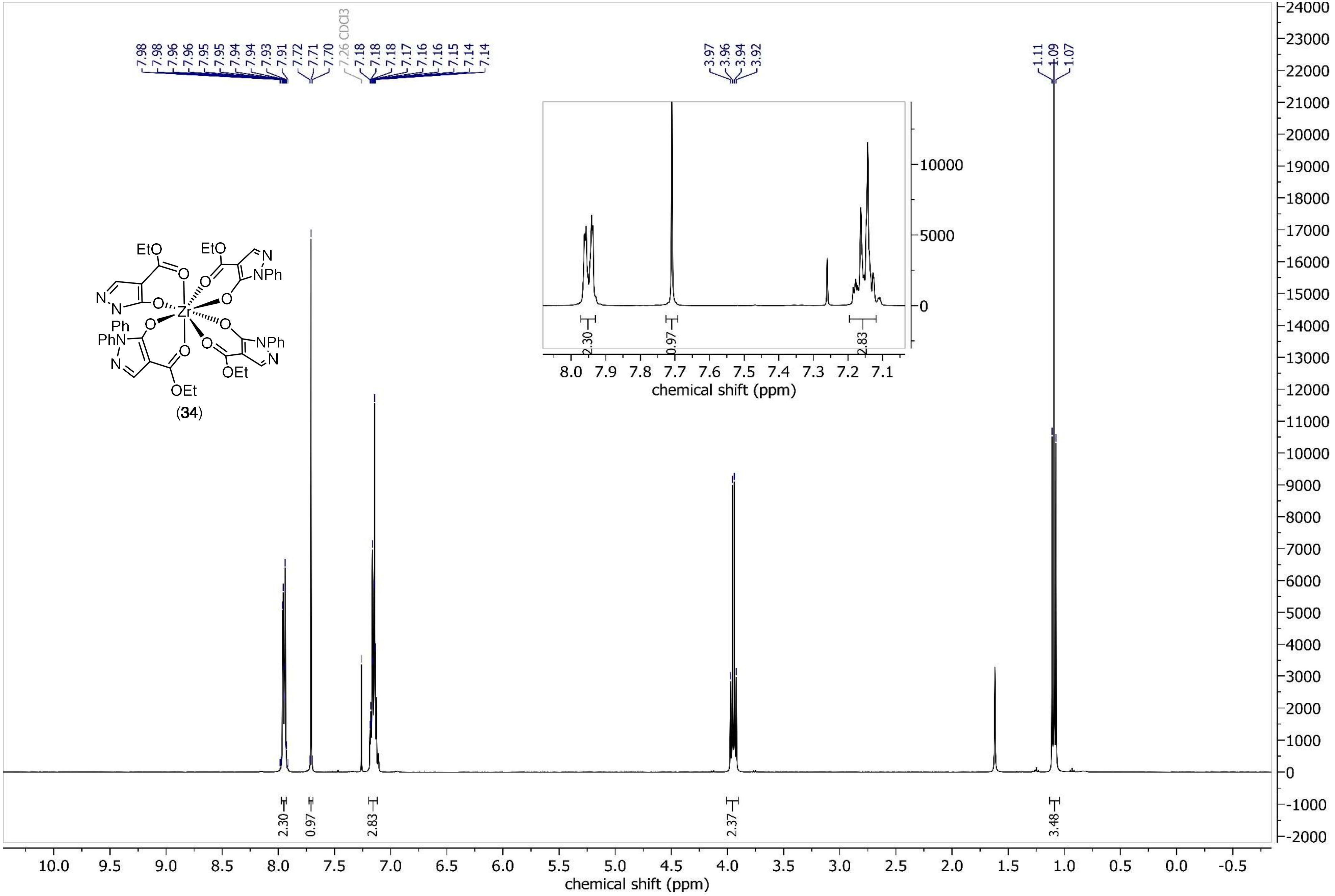

Figure S69: ^{1}H-NMR spectrum of tetrakis((4-(ethoxycarbonyl)-1-phenyl-1*H*-pyrazol-5-yl)oxy)zirconium (**34**) in $CDCl_3$.

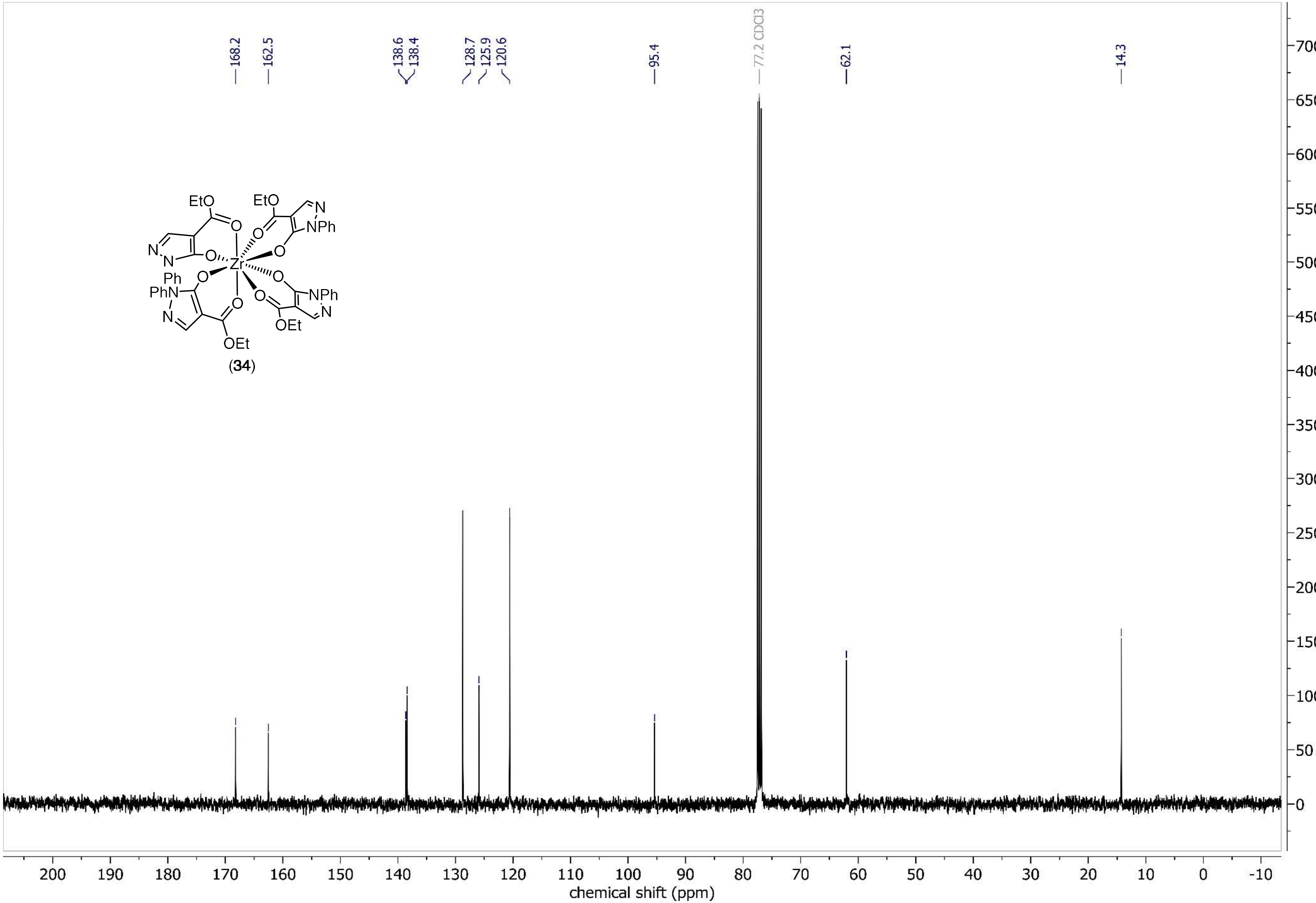

Figure S70: $^{13}C\{^{1}H\}$-NMR spectrum of tetrakis((4-(ethoxycarbonyl)-1-phenyl-1*H*-pyrazol-5-yl)oxy)zirconium (**34**) in $CDCl_3$.

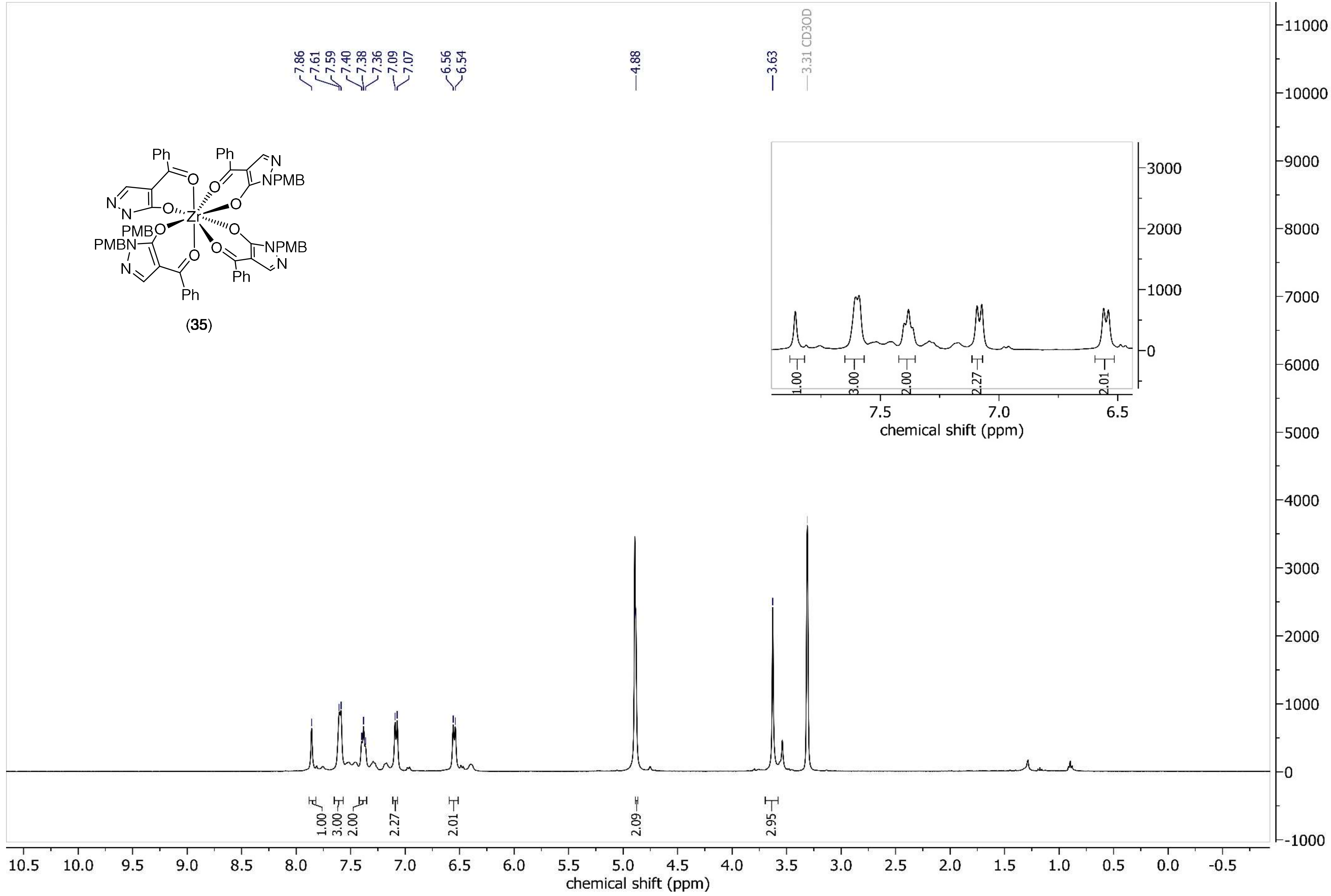

Figure S71: ^{1}H-NMR spectrum of tetrakis((4-benzoyl-1-(4-methoxybenzyl)-1*H*-pyrazol-5-yl)oxy)zirconium (**35**).

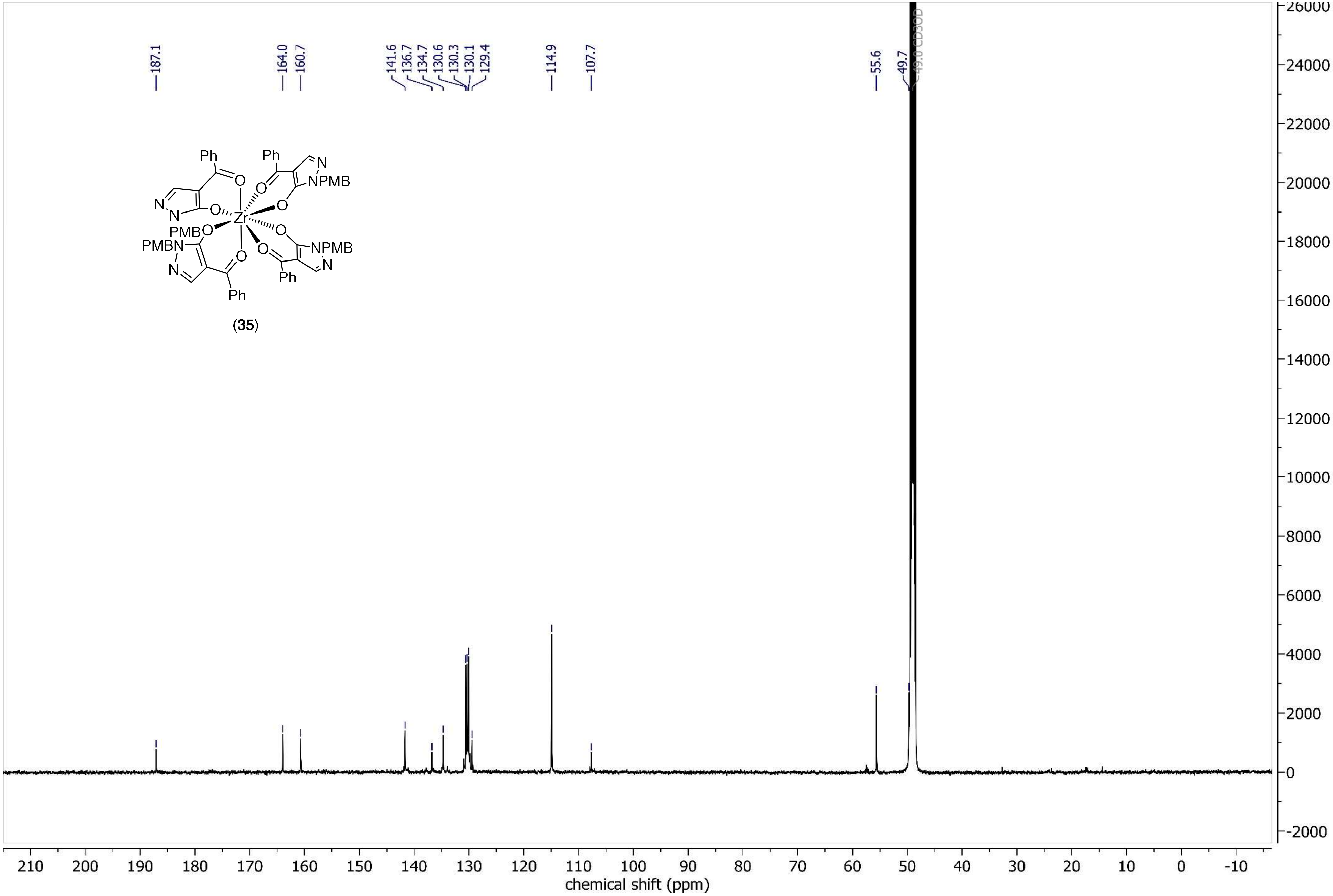

Figure S72: $^{13}C\{^{1}H\}$-NMR spectrum of tetrakis((4-benzoyl-1-(4-methoxybenzyl)-1*H*-pyrazol-5-yl)oxy)zirconium (**35**).

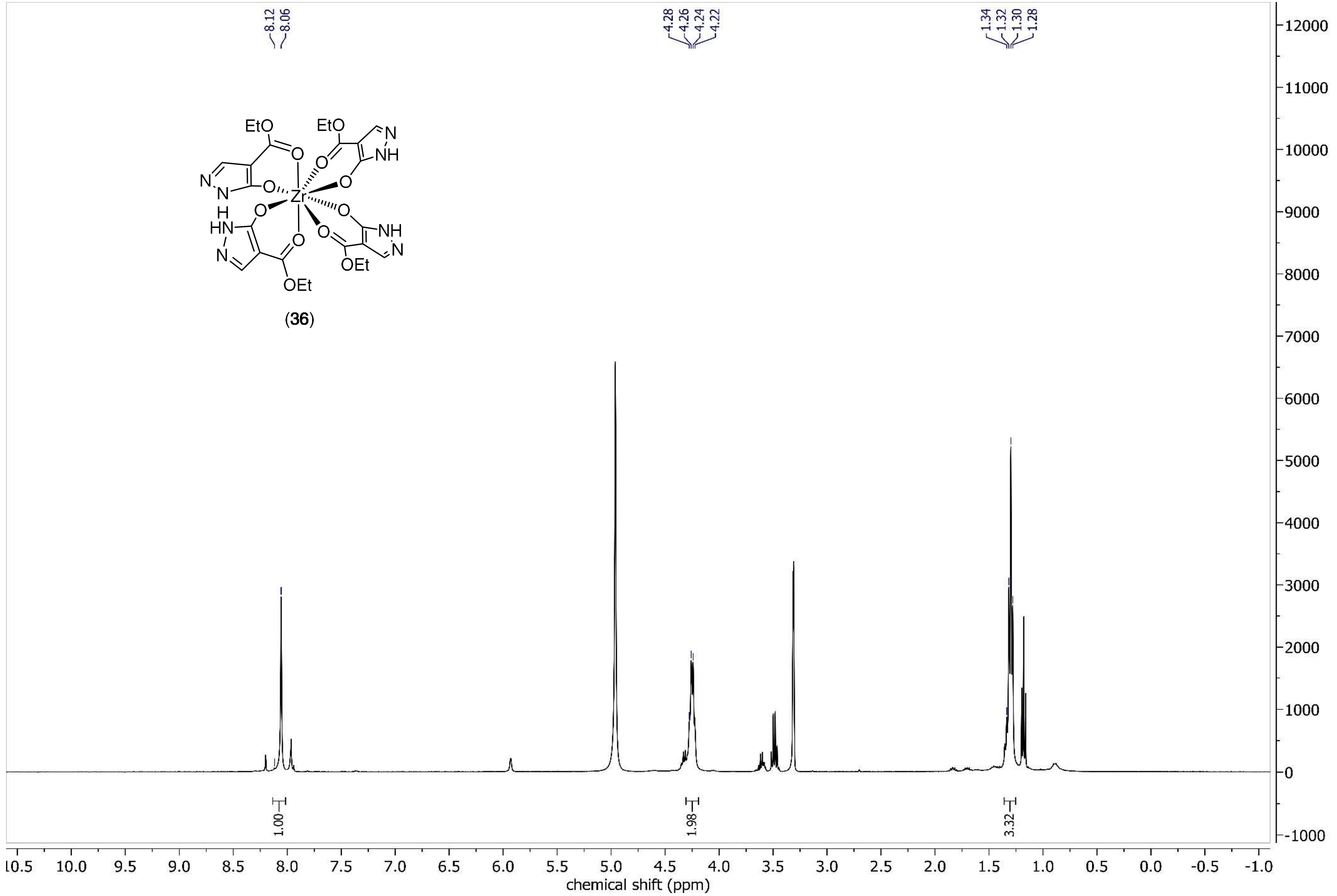

Figure S73: ^{1}H-NMR spectrum of tetrakis((4-(ethoxycarbonyl)-1*H*-pyrazol-5-yl)oxy)zirconium (**36**).

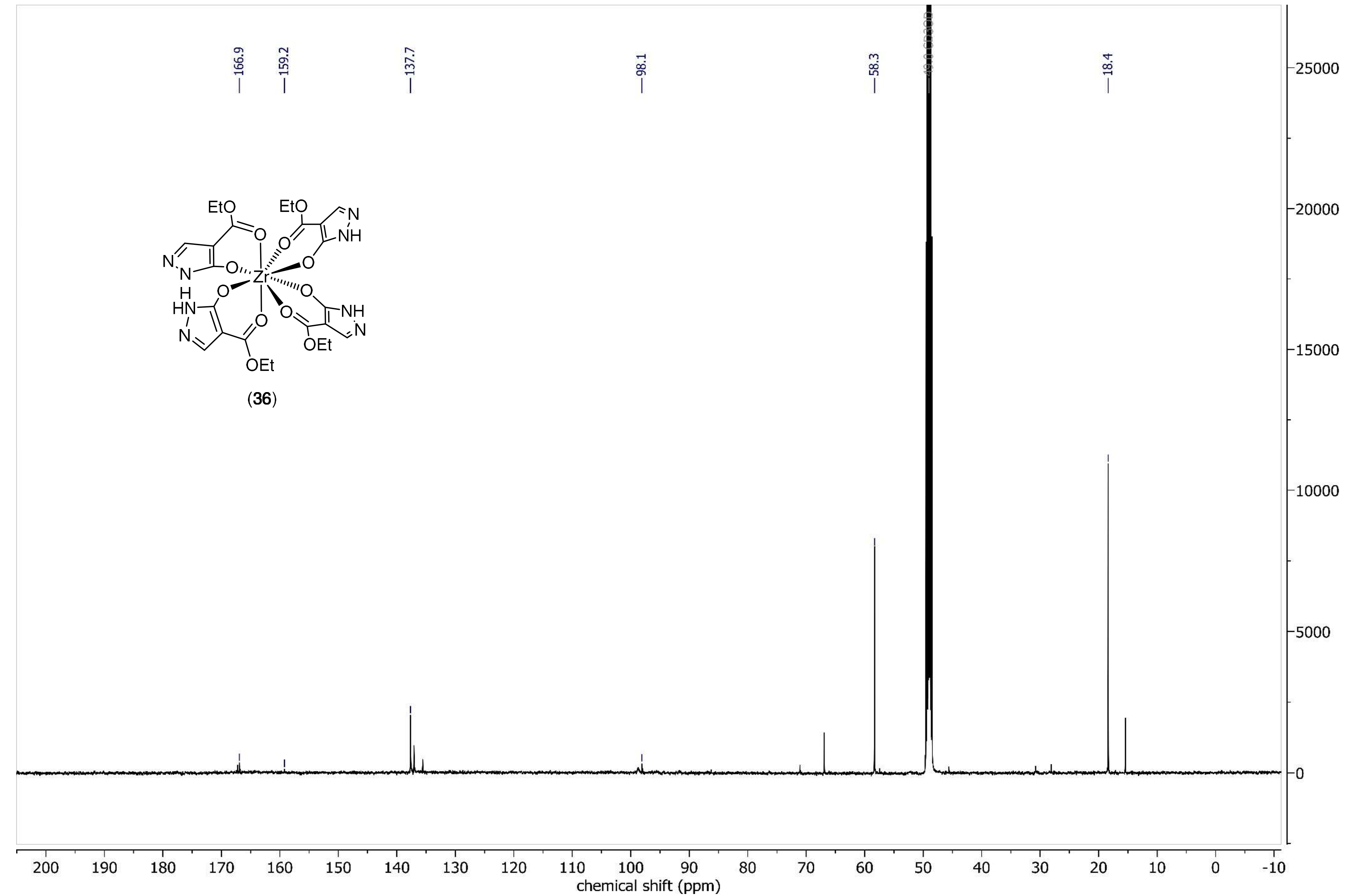

Figure S74: $^{13}C\{^{1}H\}$-NMR spectrum of tetrakis((4-(ethoxycarbonyl)-1*H*-pyrazol-5-yl)oxy)zirconium (**36**).

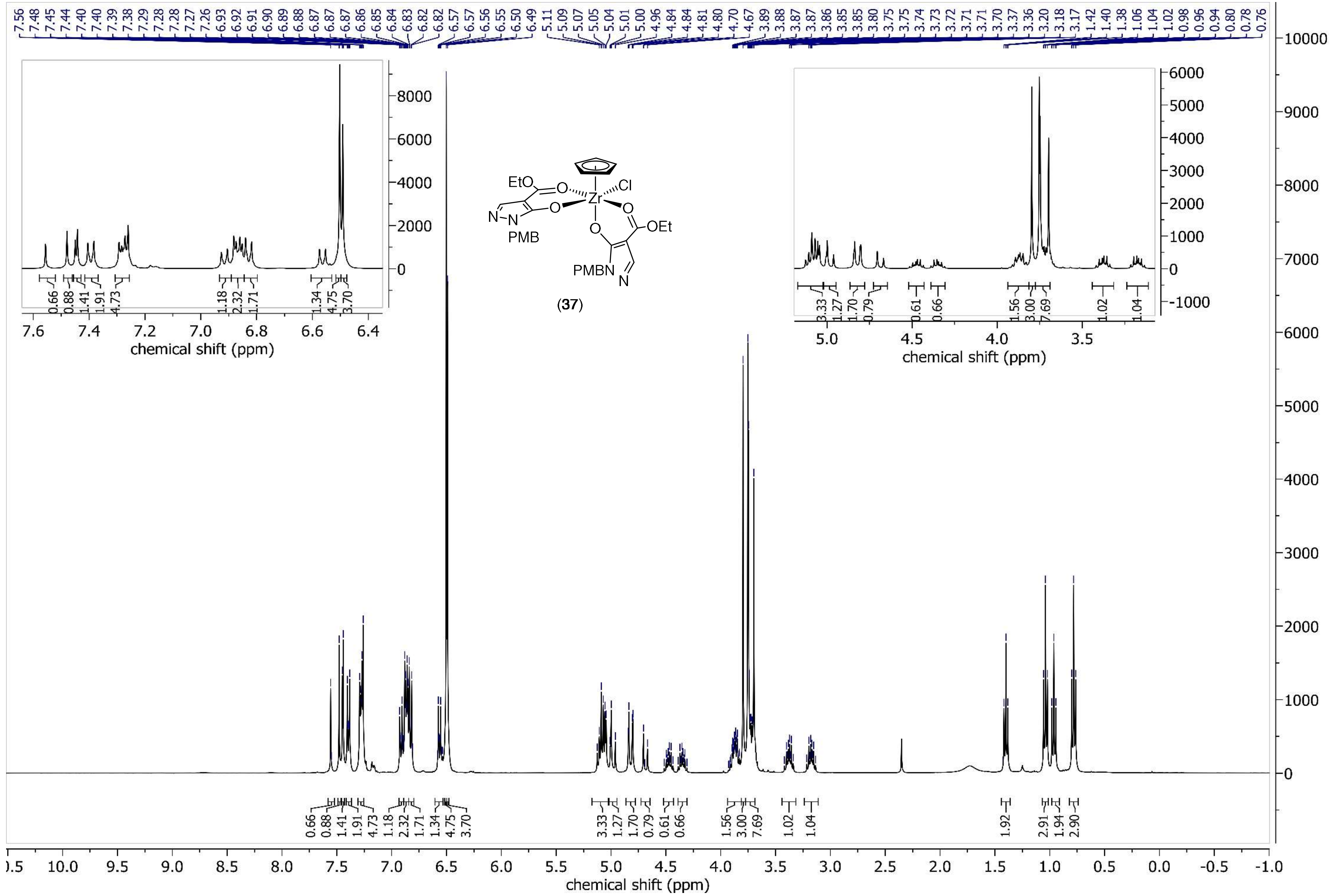

Figure S75: ^{1}H-NMR spectrum of Zr(Cl)Cp-PMB-pyr complex (**37**).

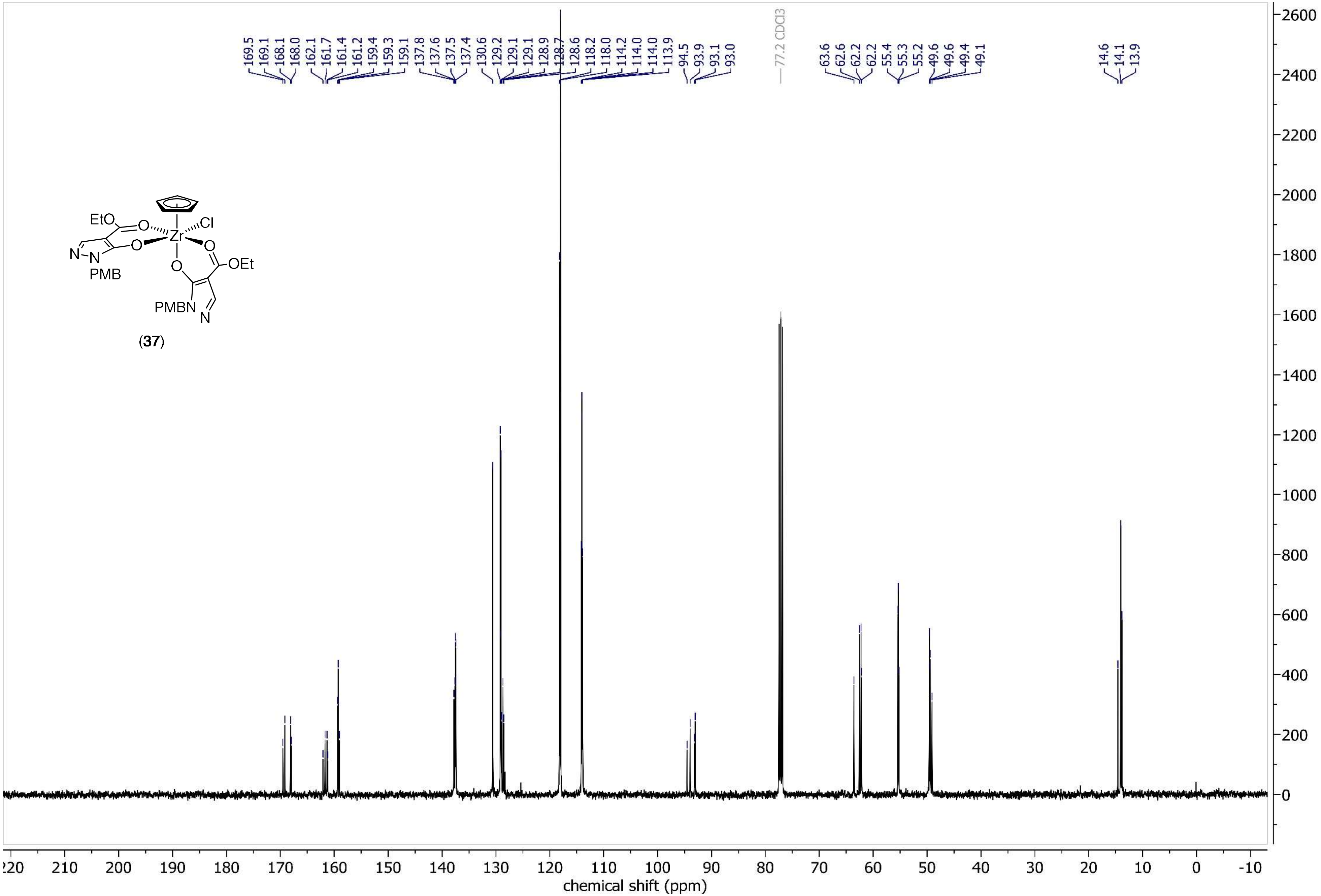

Figure S76: $^{13}C\{^{1}H\}$-NMR spectrum of Zr(Cl)Cp-PMB-pyr complex (**37**).

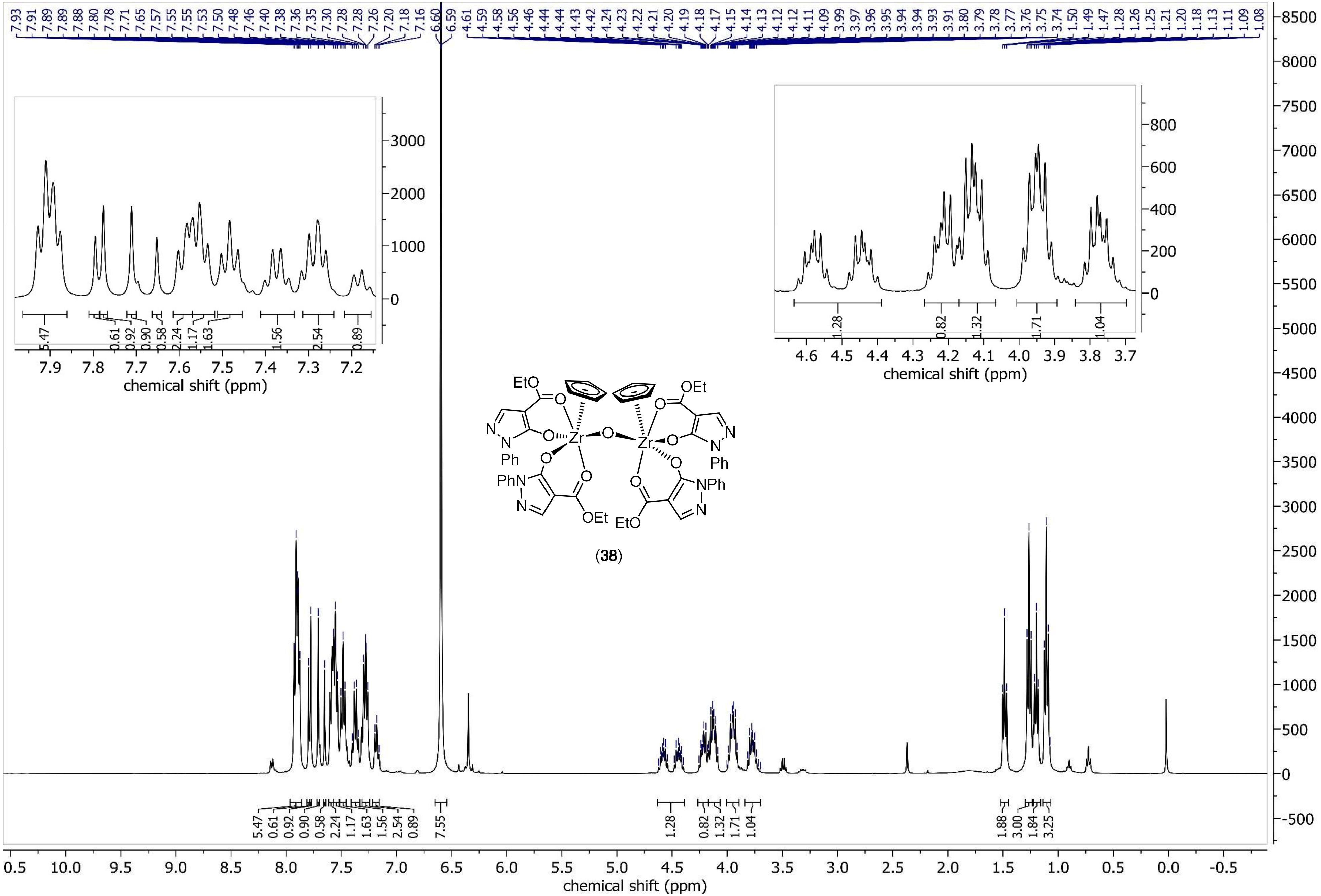

(**38**)

Figure S77: ^{1}H-NMR spectrum of μ-O-[ZrCp(Ph-pyr)$_2$]$_2$ complex (**38**).

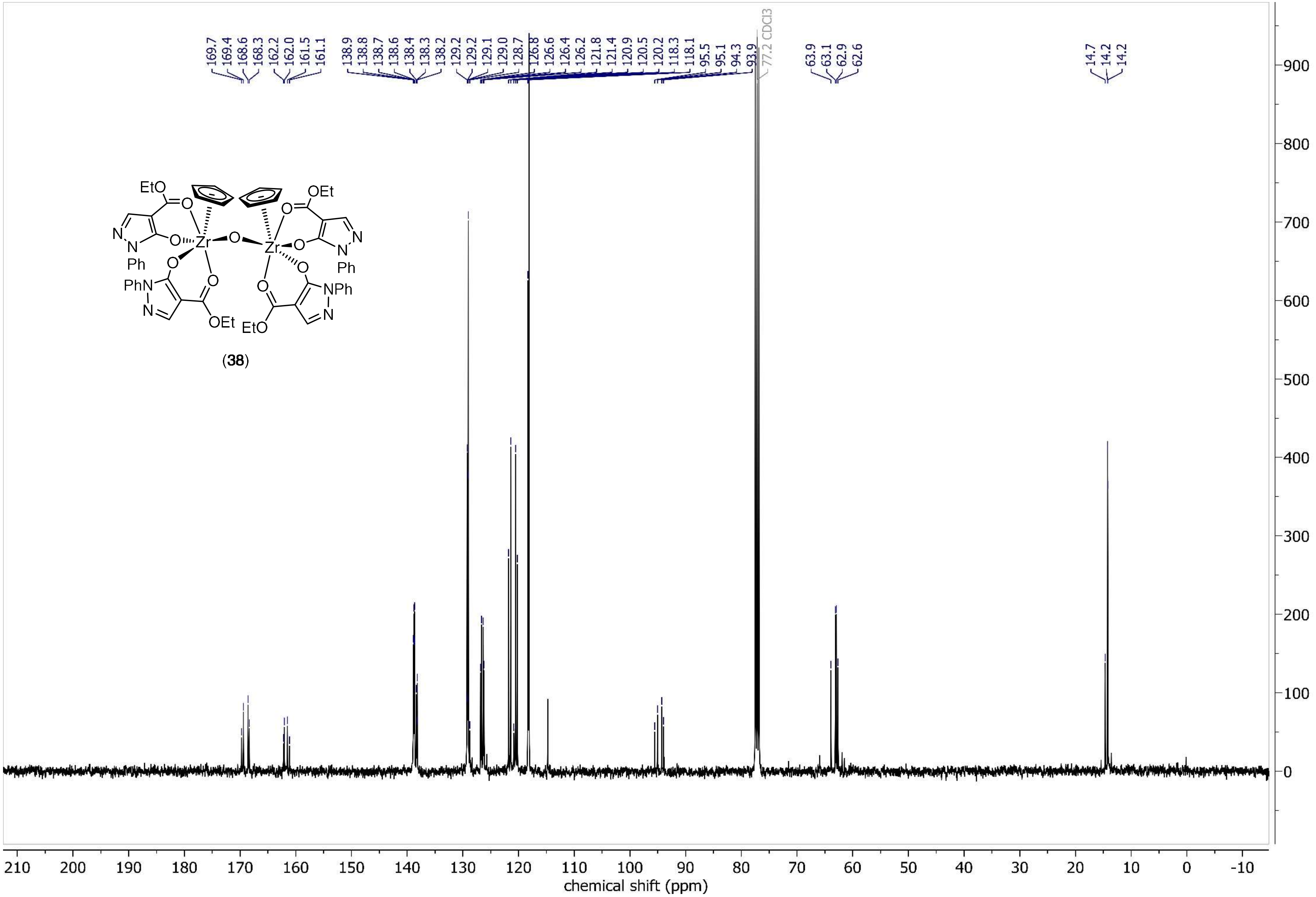

Figure S78: $^{13}C\{^{1}H\}$-NMR spectrum of μ-O-[ZrCp(Ph-pyr)$_2$]$_2$ complex (**38**).

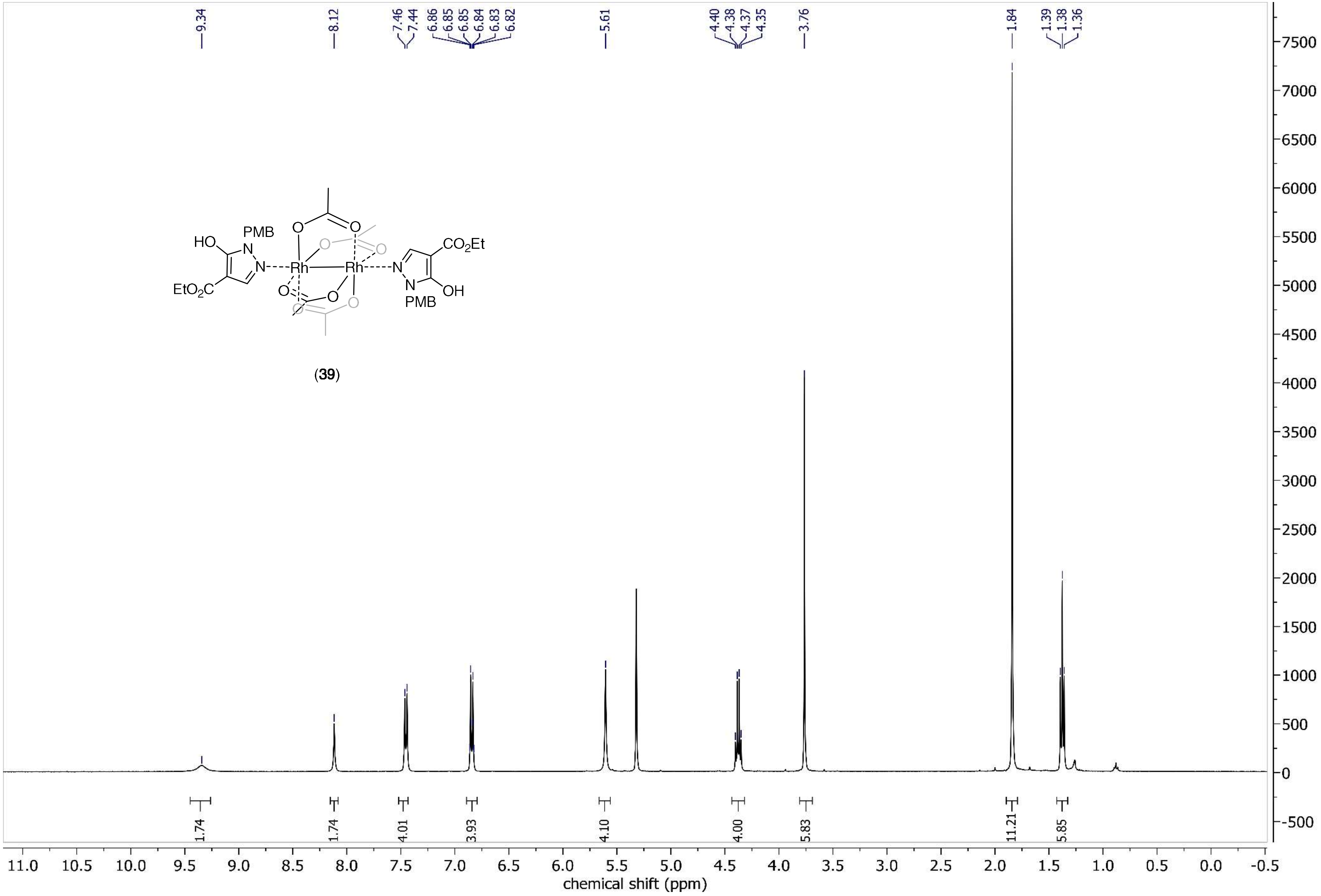

Figure S79: ^{1}H-NMR spectrum of bis(ethyl 5-hydroxy-1-(4-methoxybenzyl)-1*H*-pyrazole-4-carboxylate)tetrakis(acetato)dirhodium (**39**) in CD_2Cl_2.

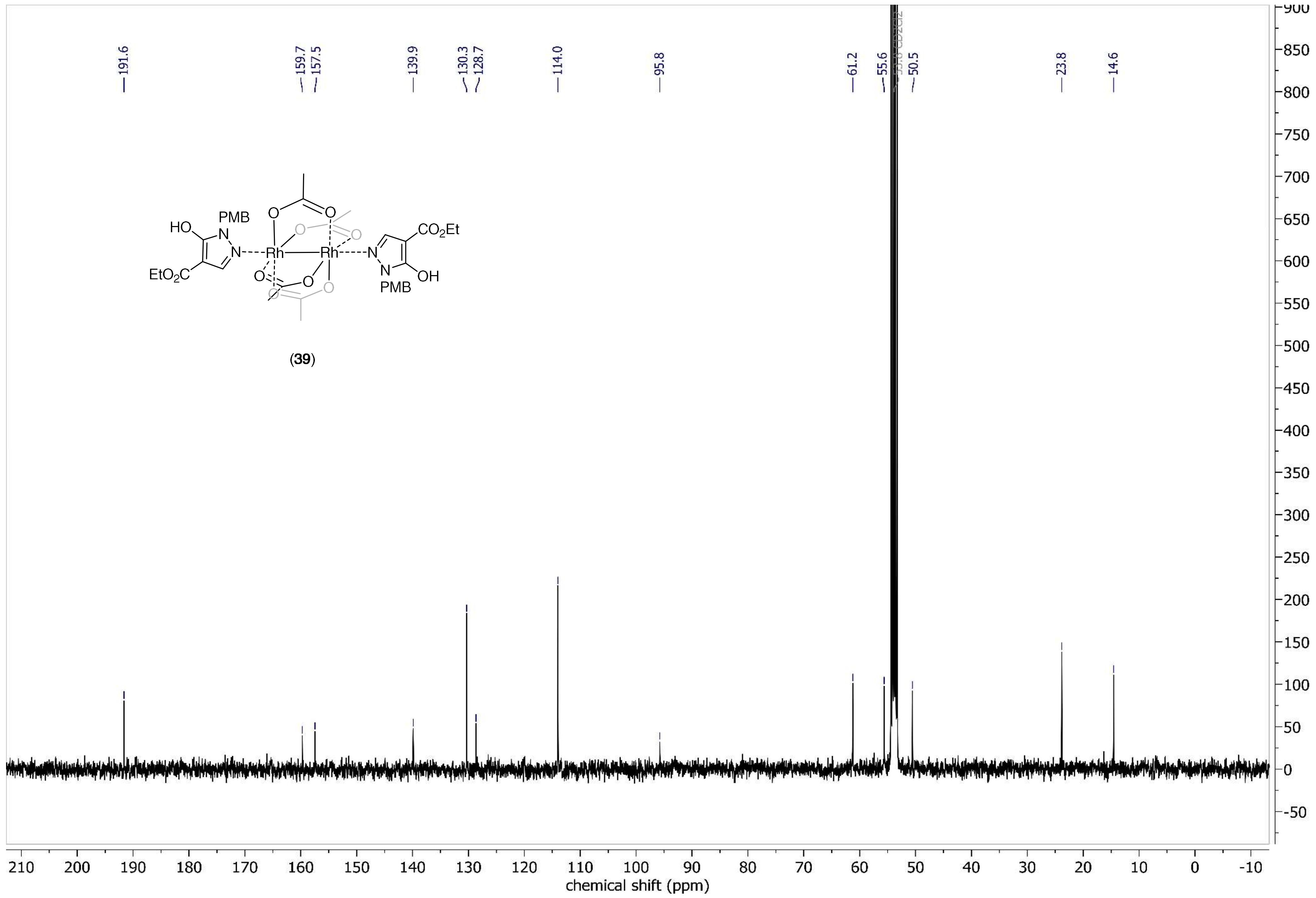

Figure S80: $^{13}C\{^{1}H\}$-NMR spectrum of bis(ethyl 5-hydroxy-1-(4-methoxybenzyl)-1*H*-pyrazole-4-carboxylate)tetrakis(acetato)dirhodium (**39**) in CD_2Cl_2.

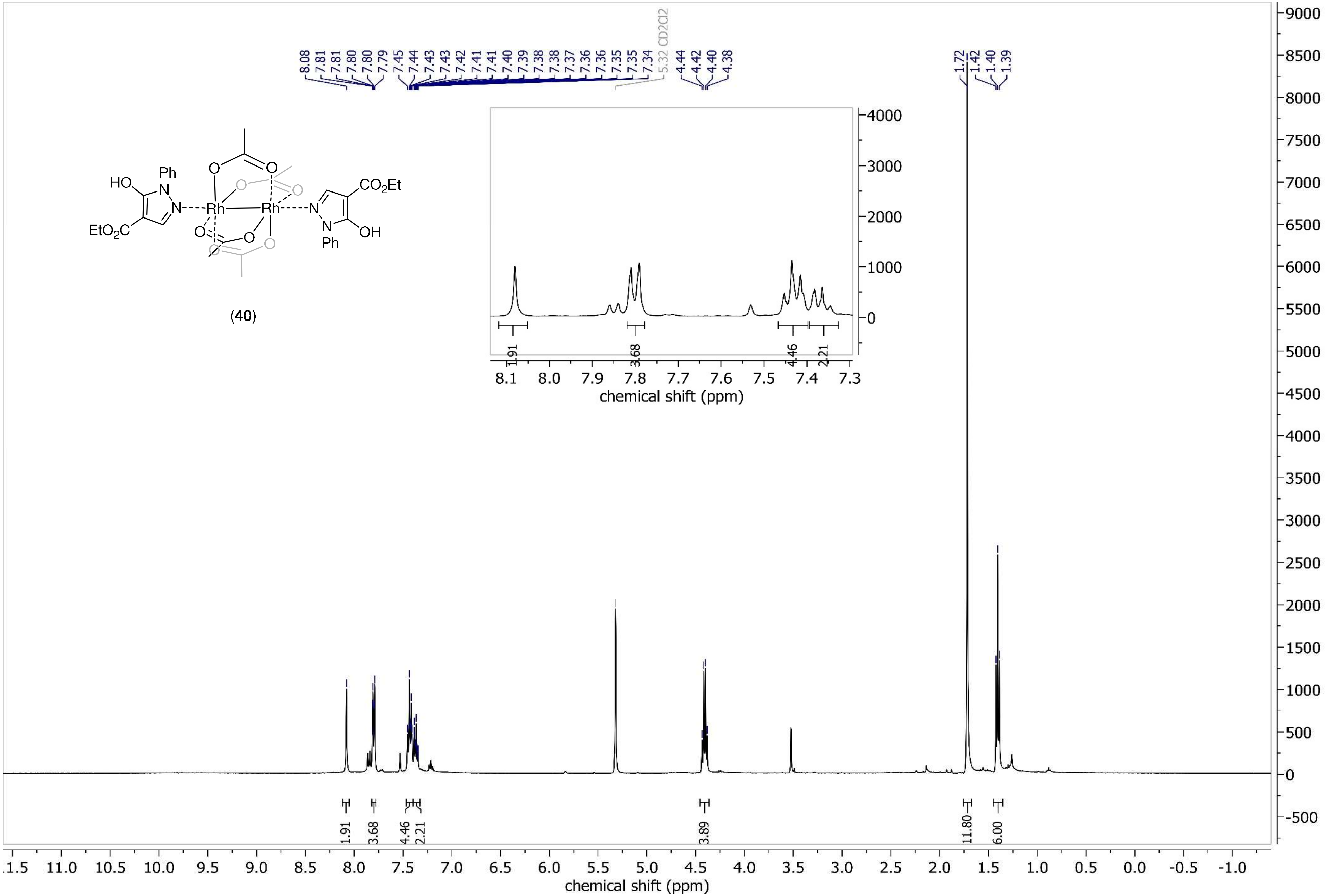

Figure S81: ^{1}H-NMR spectrum of bis(ethyl 5-hydroxy-1-phenyl-1*H*-pyrazole-4-carboxylate)tetrakis(acetato)dirhodium (**40**) in CD_2Cl_2.

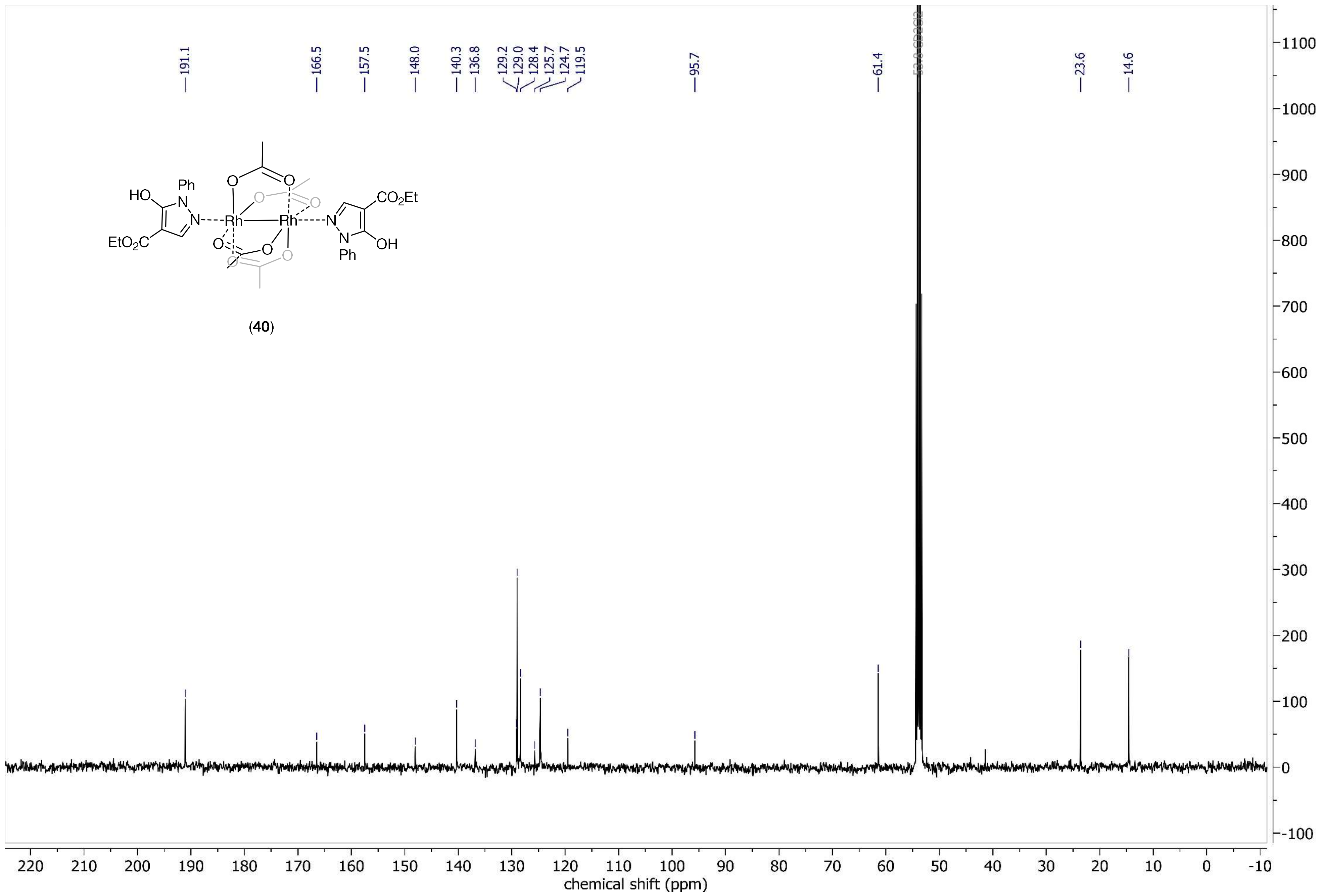

Figure S82: $^{13}C\{^{1}H\}$-NMR spectrum of bis(ethyl 5-hydroxy-1-phenyl-1*H*-pyrazole-4-carboxylate)tetrakis(acetato)dirhodium (**40**) in CD_2Cl_2.

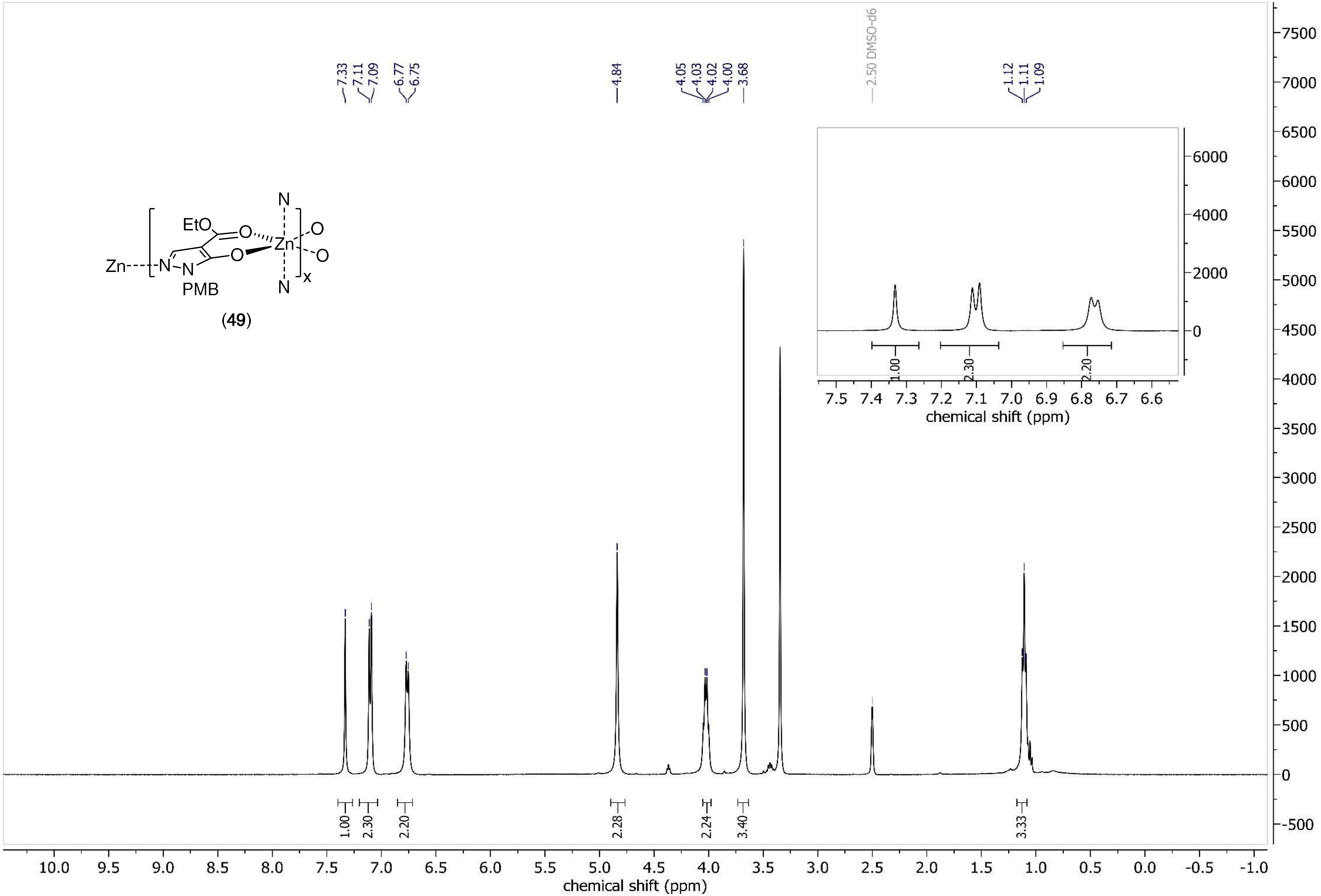

Figure S83: ^{1}H-NMR spectrum of ((4-(ethoxycarbonyl)-1-(4-methoxybenzyl)-1*H*-pyrazol-5-yl)oxy)zinc (**49**) in dmso-d_6.

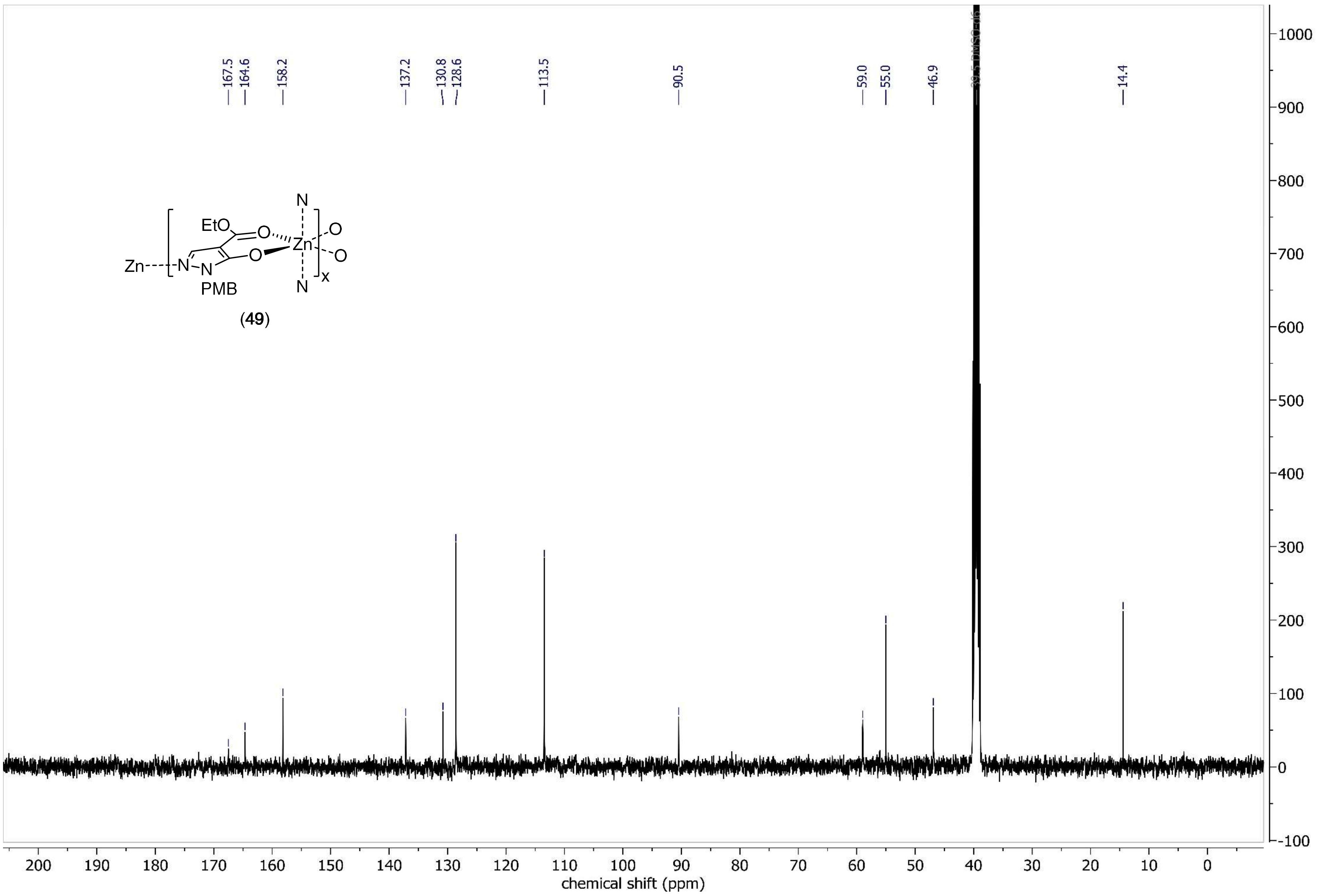

Figure S84: $^{13}C\{^1H\}$-NMR spectrum of ((4-(ethoxycarbonyl)-1-(4-methoxybenzyl)-1*H*-pyrazol-5-yl)oxy)zinc (**49**) in dmso-d_6.

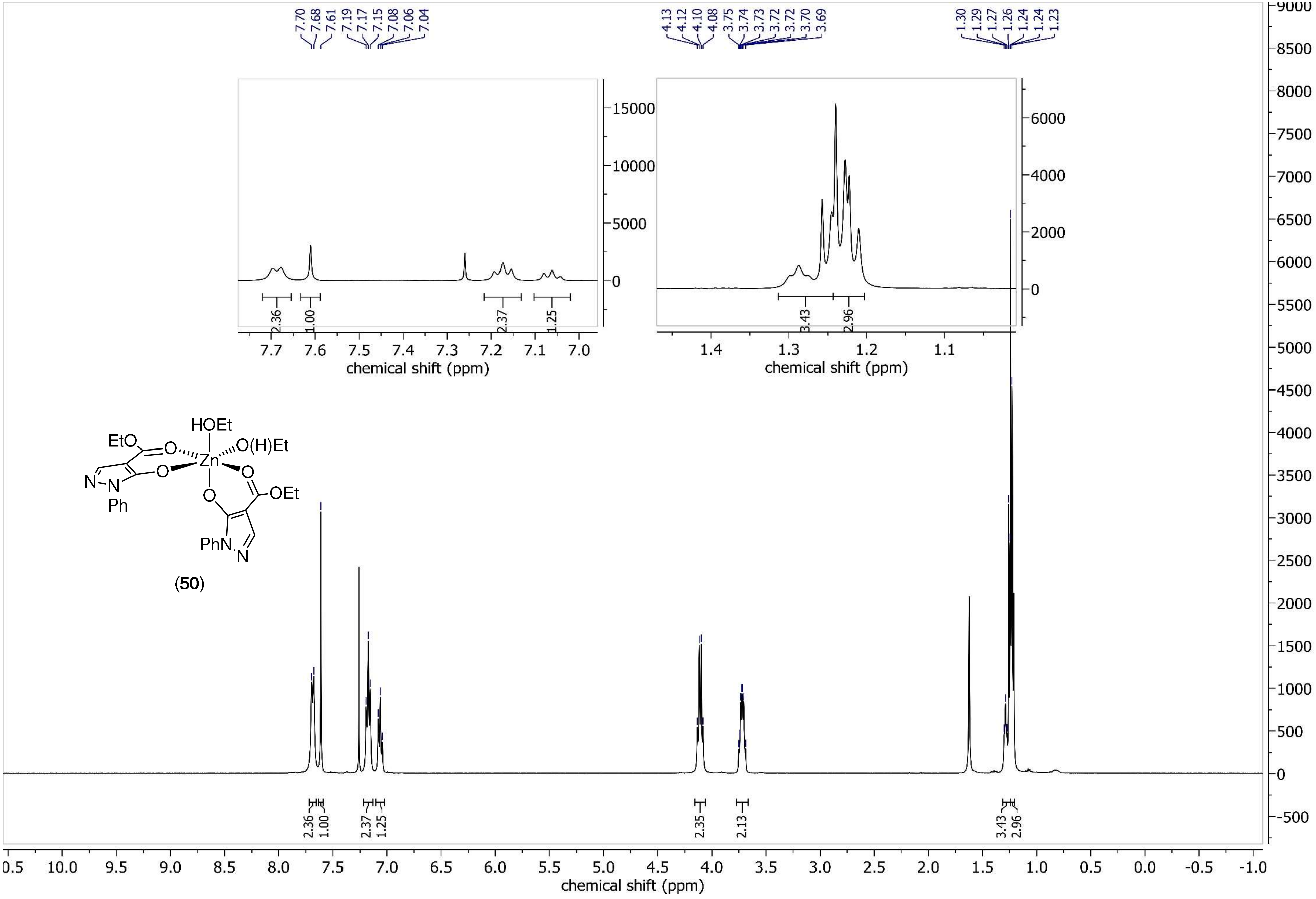

Figure S85: ^{1}H-NMR spectrum of bis(ethanol) bis((4-(ethoxycarbonyl)-1-phenyl-1*H*-pyrazol-5-yl)oxy)zinc (**50**).

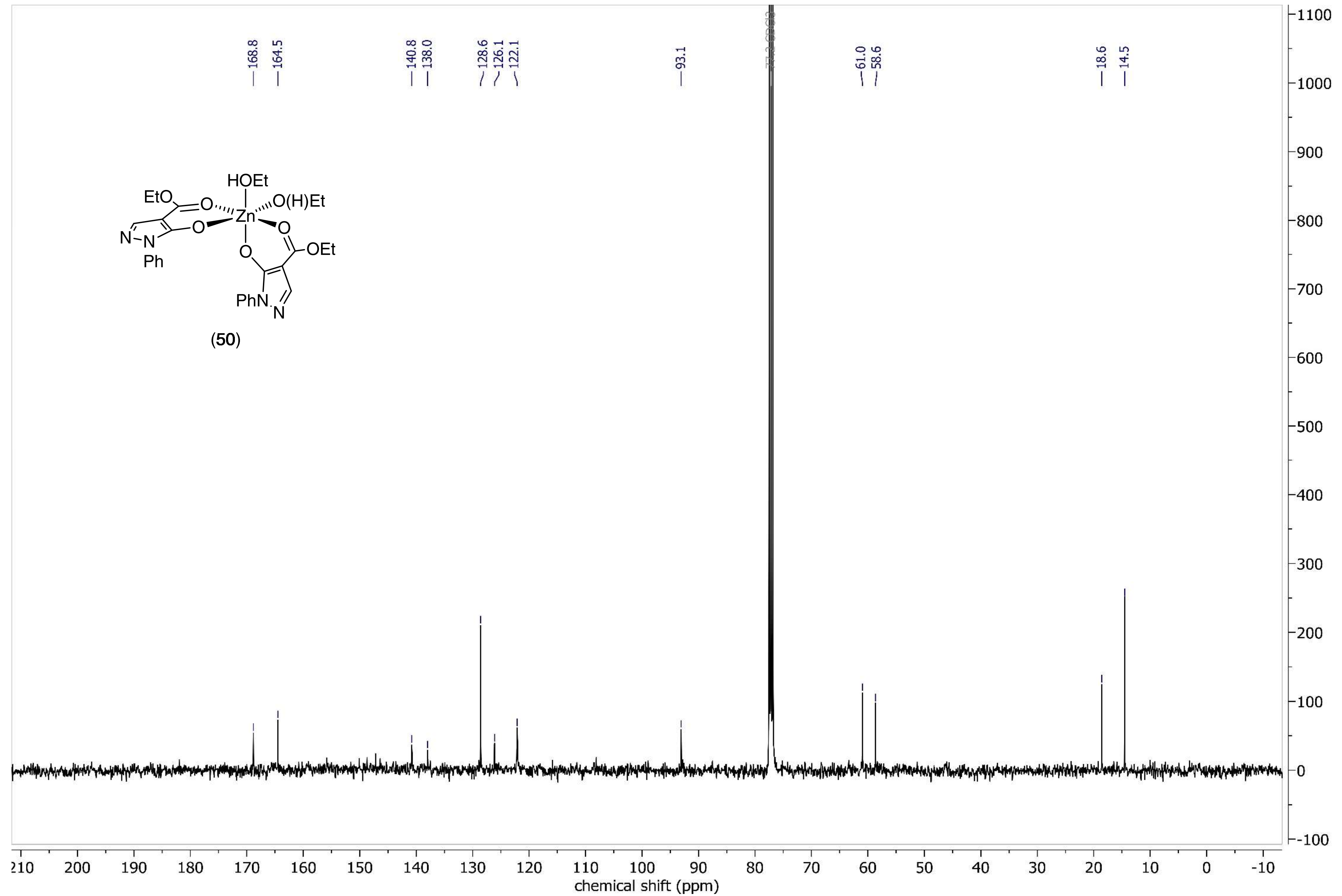

Figure S86: $^{13}C\{^{1}H\}$-NMR spectrum of bis(ethanol) bis((4-(ethoxycarbonyl)-1-phenyl-1*H*-pyrazol-5-yl)oxy)zinc (**50**).

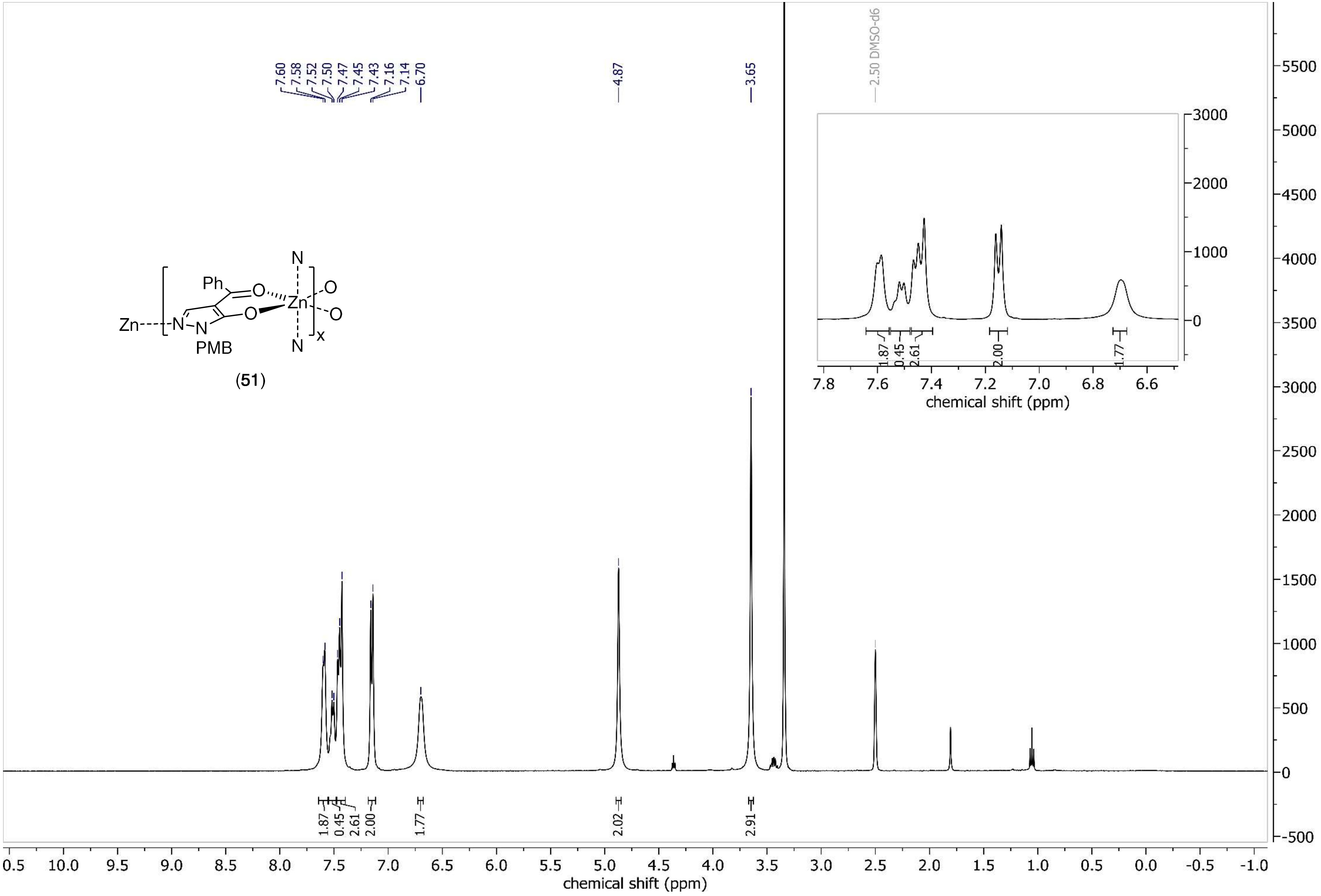

Figure S87: ^{1}H-NMR spectrum of ((4-benzoyl-1-(4-methoxybenzyl)-1*H*-pyrazol-5-yl)oxy)zinc polymer (**51**) in dmso-d_6.

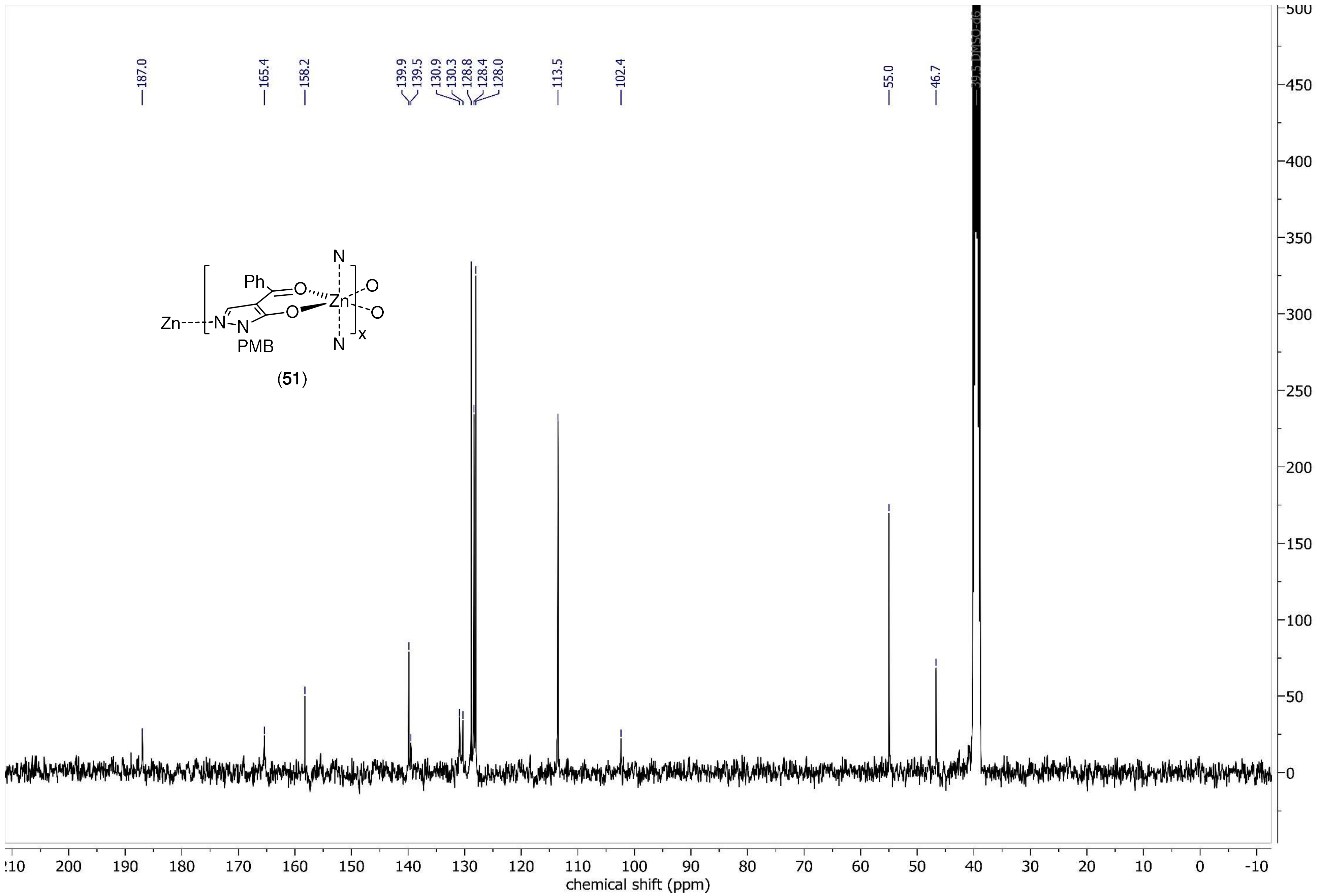

Figure S88: $^{13}C\{^{1}H\}$-NMR spectrum of ((4-benzoyl-1-(4-methoxybenzyl)-1*H*-pyrazol-5-yl)oxy)zinc polymer (**51**) in dmso-d_6.

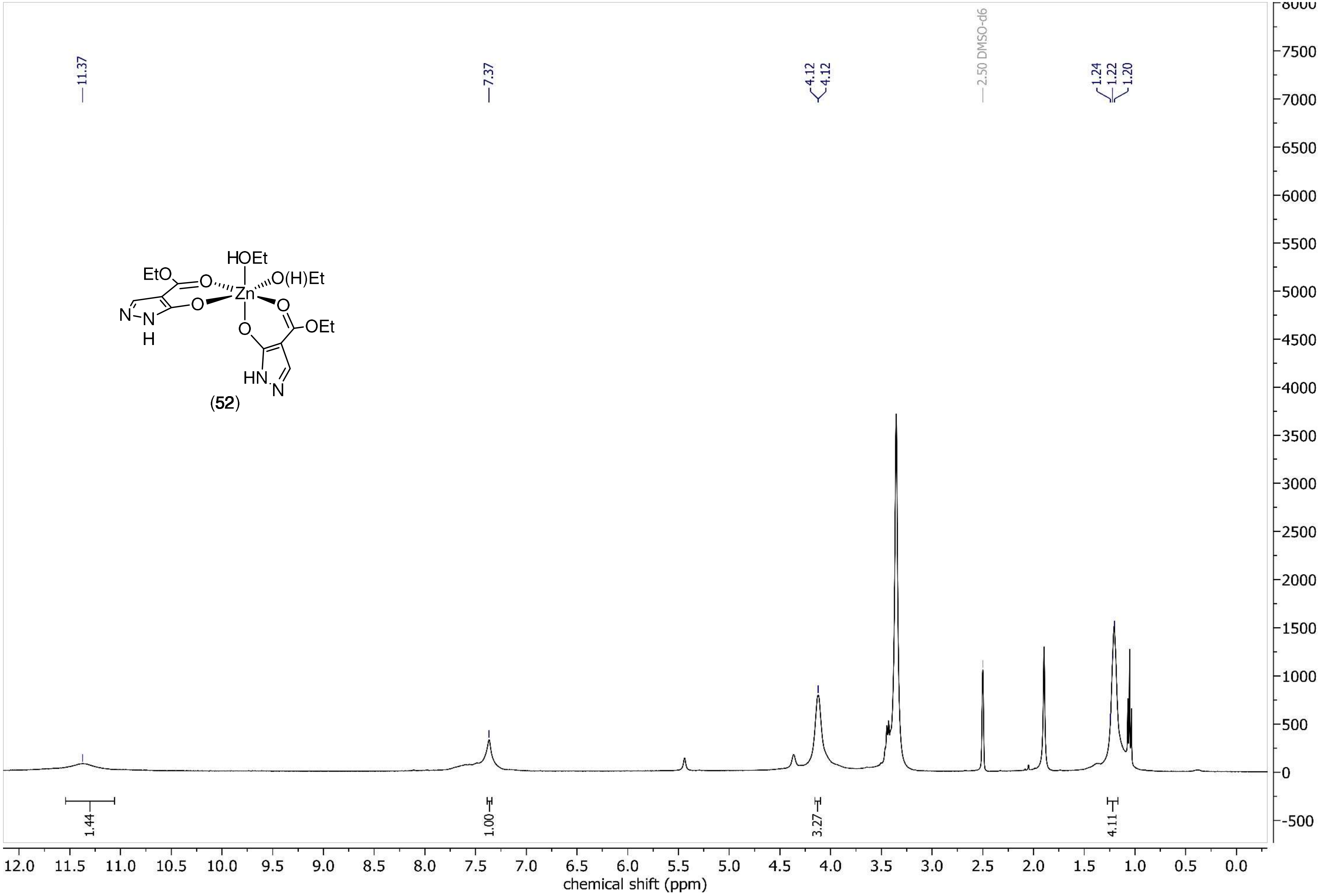

Figure S89: ^{1}H-NMR spectrum of bis(ethanol) bis((4-(ethoxycarbonyl)-1*H*-pyrazol-5-yl)oxy)zinc (**52**).

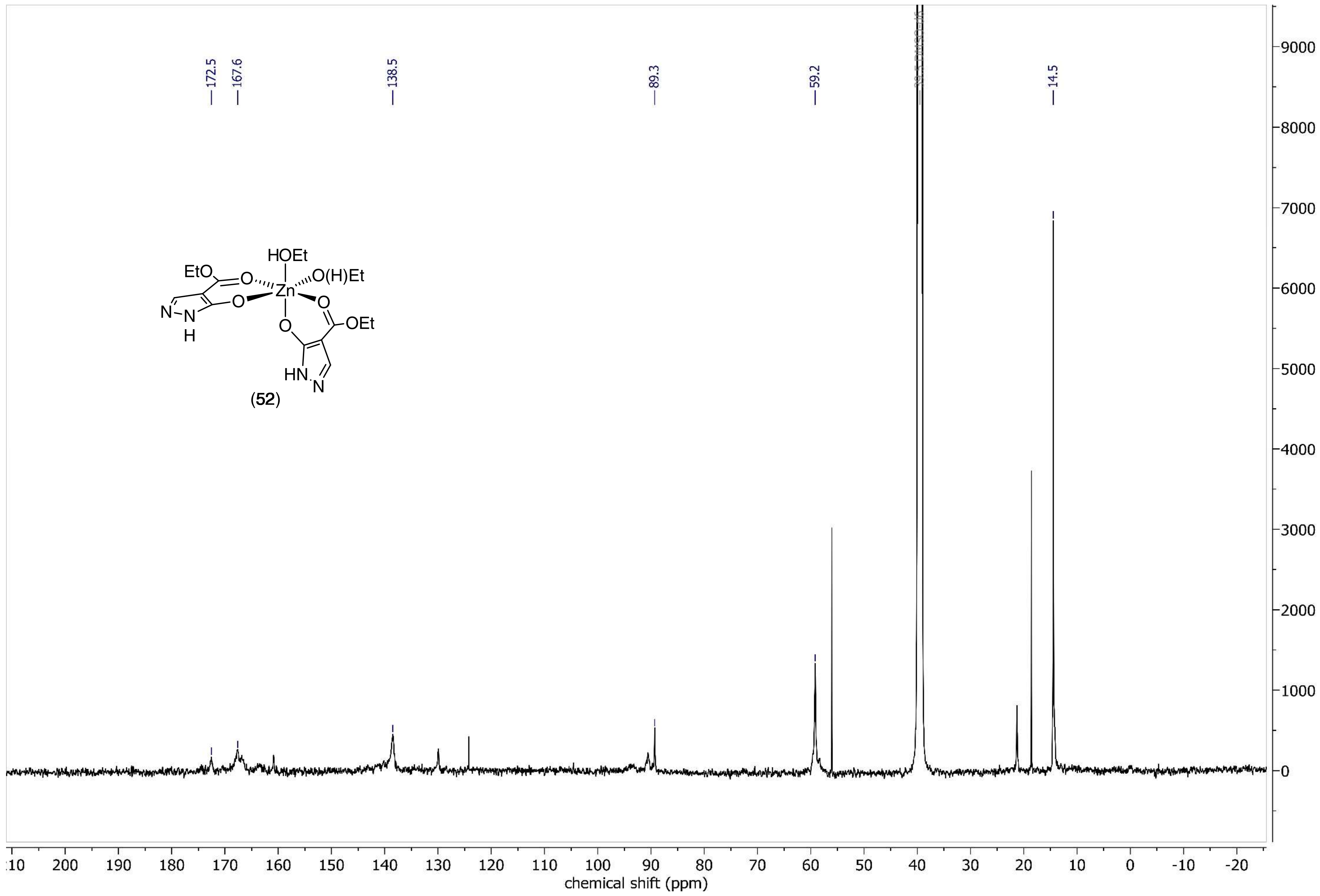

Figure S90: $^{13}C\{^1H\}$-NMR spectrum of bis(ethanol) bis((4-(ethoxycarbonyl)-1*H*-pyrazol-5-yl)oxy)zinc (**52**).

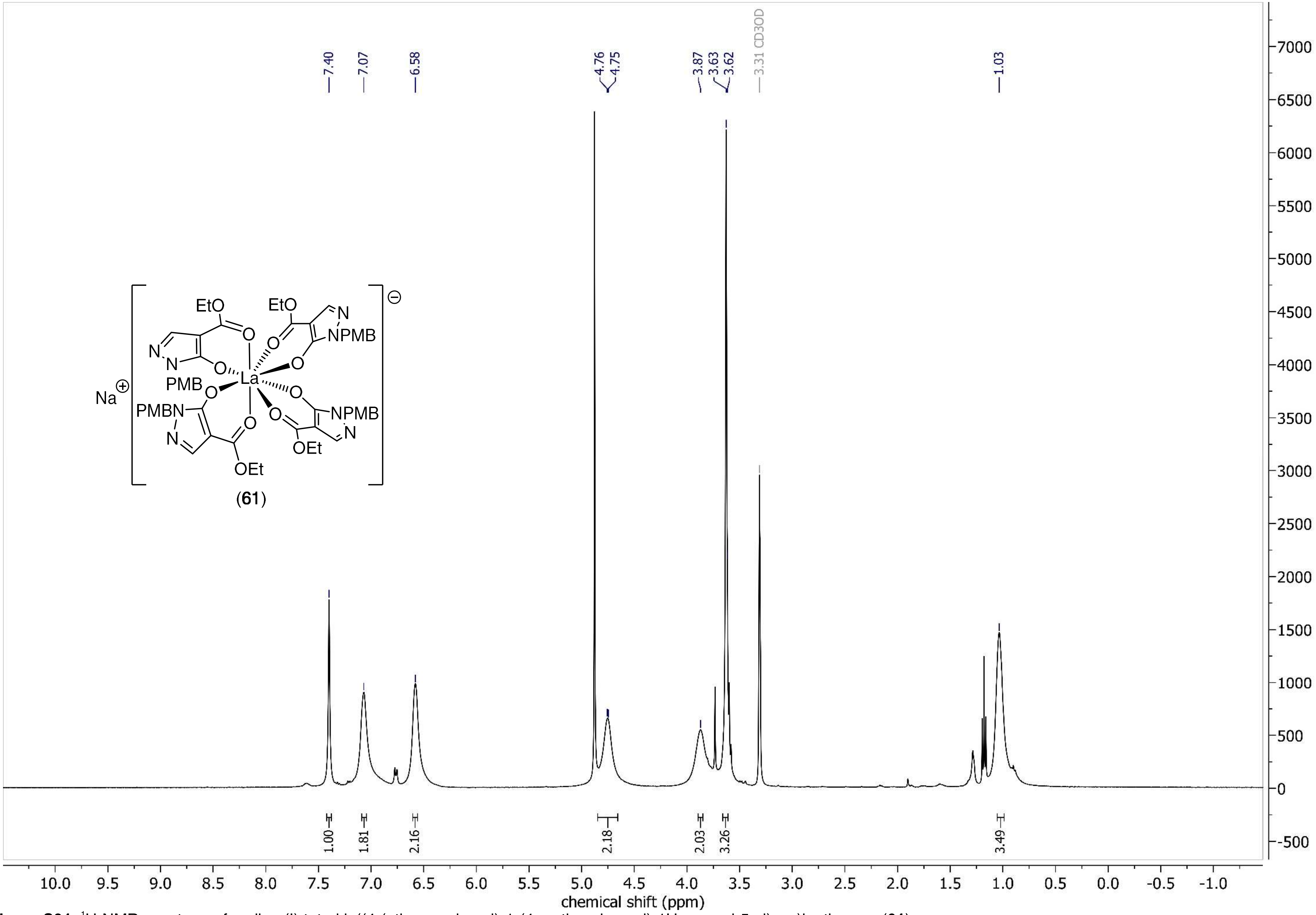

Figure S91: ^{1}H-NMR spectrum of sodium(I) tetrakis((4-(ethoxycarbonyl)-1-(4-methoxybenzyl)-1H-pyrazol-5-yl)oxy)lanthanum (**61**).

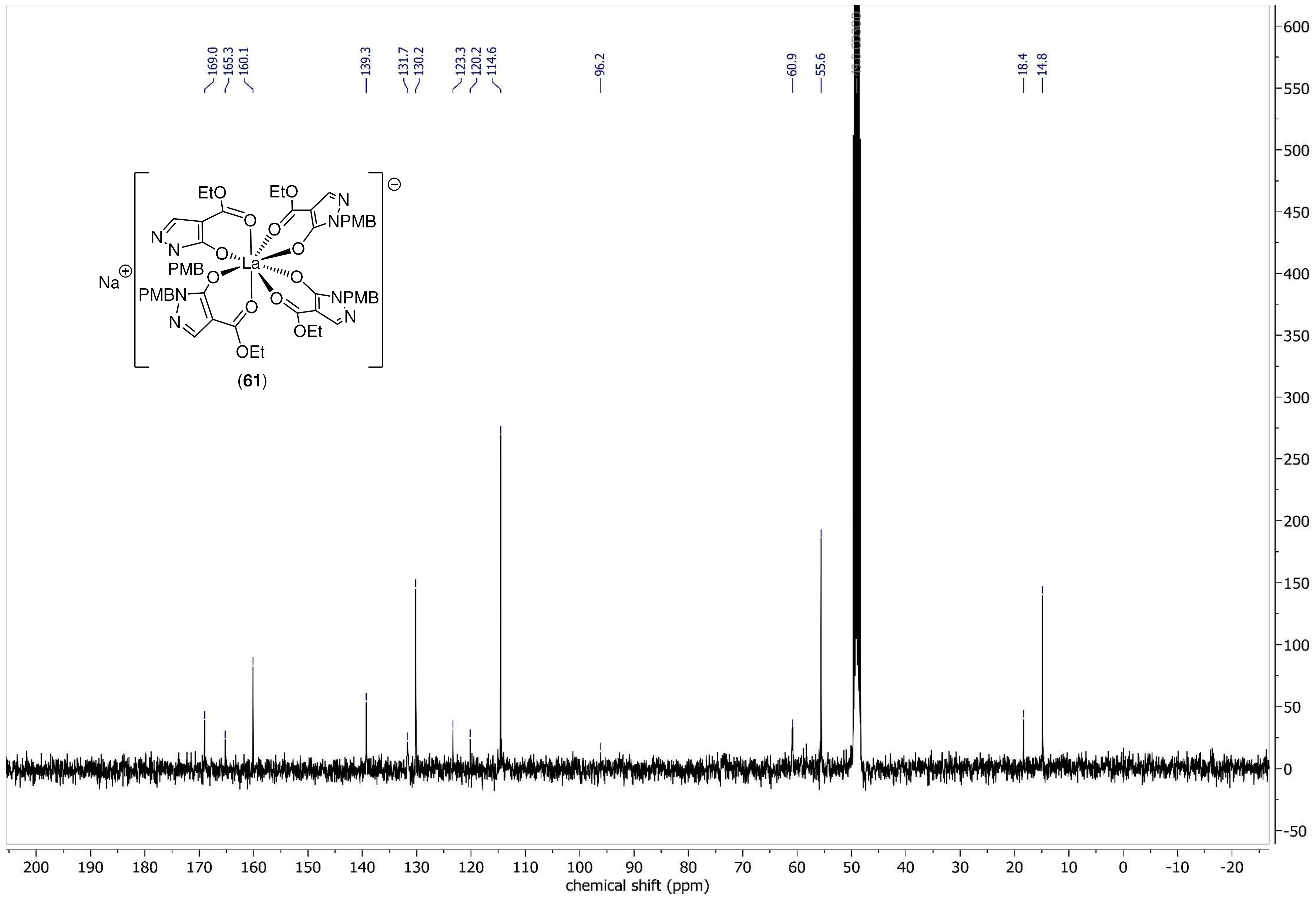

Figure S92: $^{13}C\{^{1}H\}$-NMR spectrum of (aqua)(ethanol) tris((4-(ethoxycarbonyl)-1-(4-methoxybenzyl)-1*H*-pyrazol-5-yl)oxy)lanthanum (**61**).

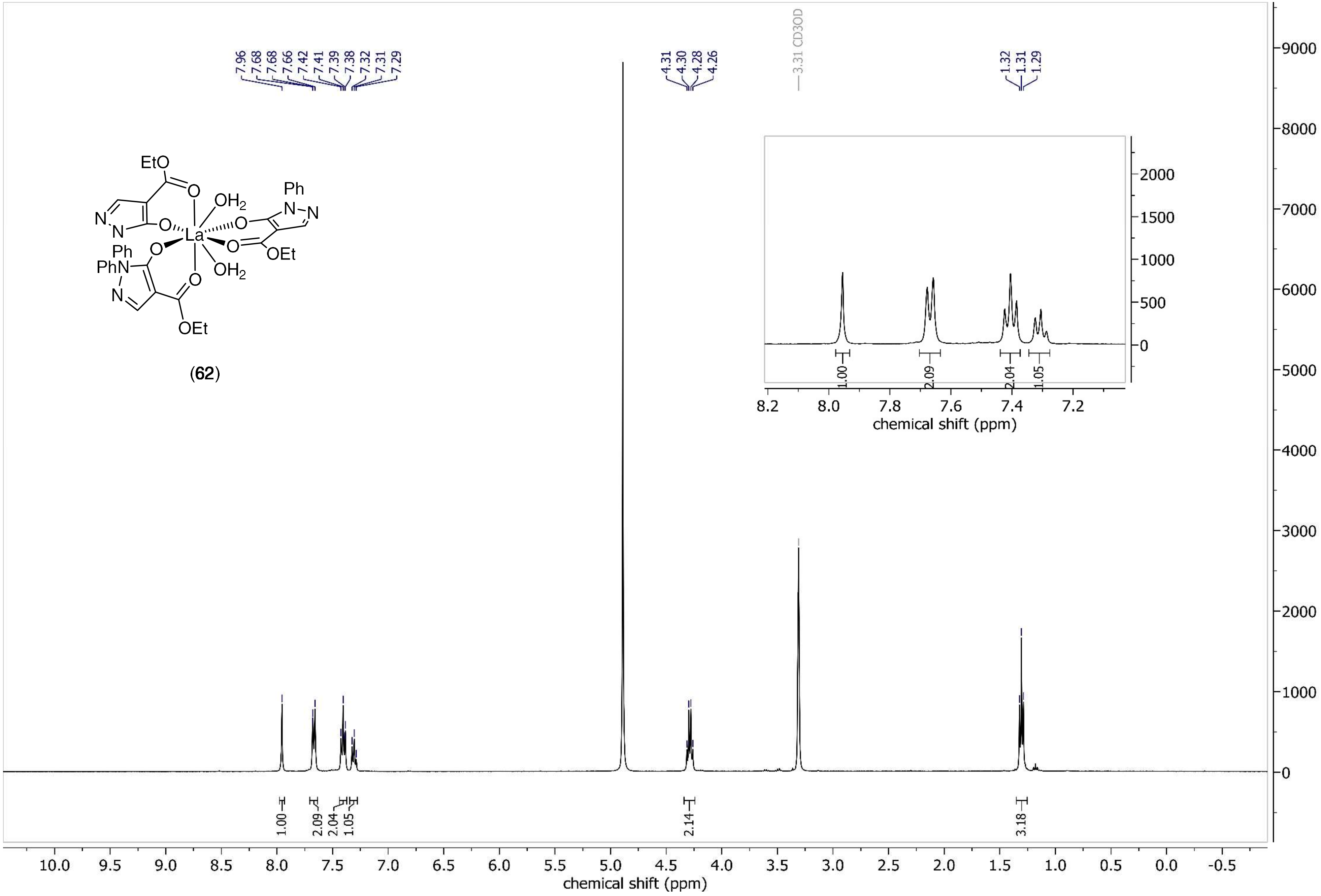

Figure S93: ^{1}H-NMR spectrum of bis(aqua) tris((4-(ethoxycarbonyl)-1-phenyl-1*H*-pyrazol-5-yl)oxy)lanthanum (**62**).

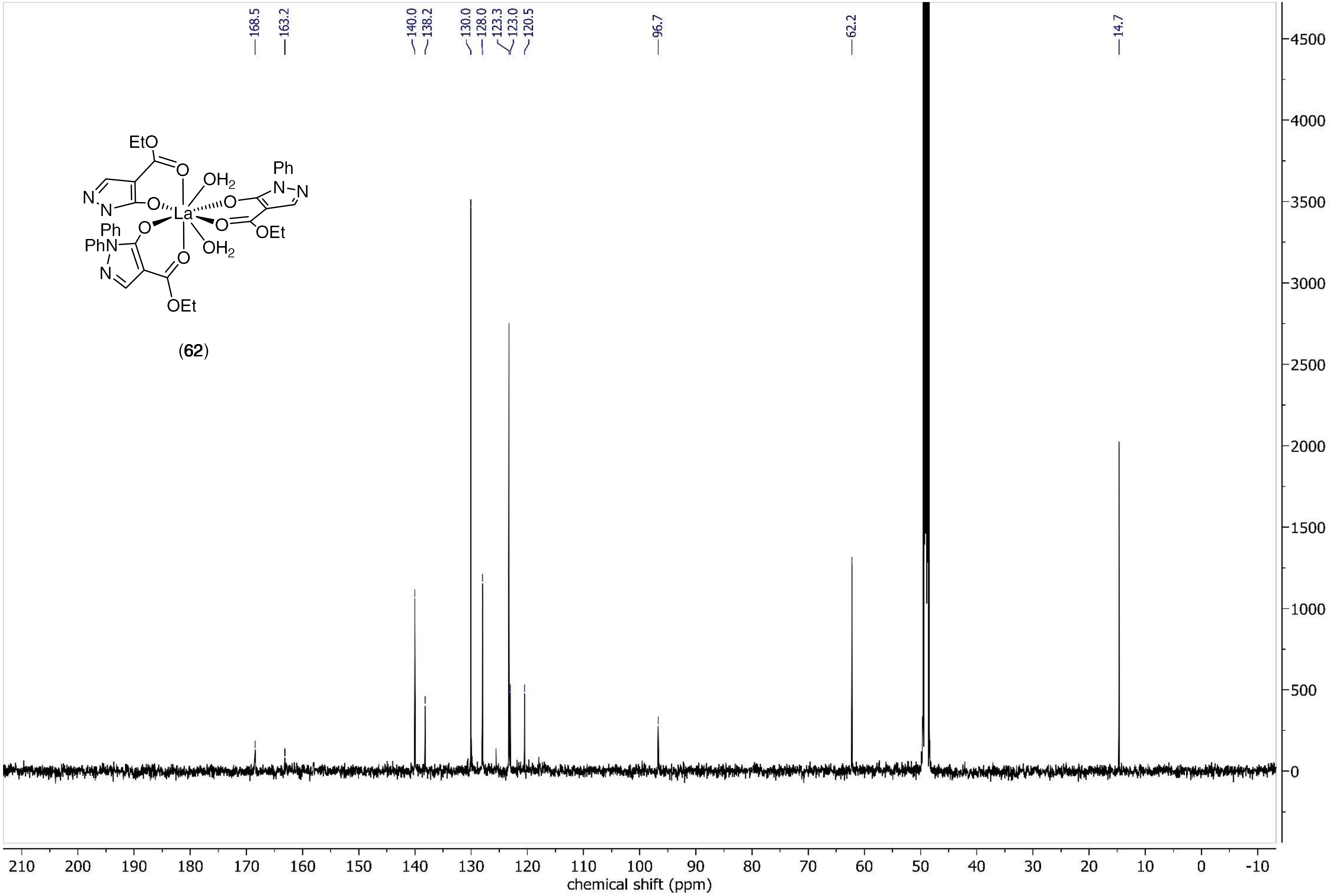

Figure S94: $^{13}C\{^1H\}$-NMR spectrum of bis(aqua) tris((4-(ethoxycarbonyl)-1-phenyl-1*H*-pyrazol-5-yl)oxy)lanthanum (**62**).

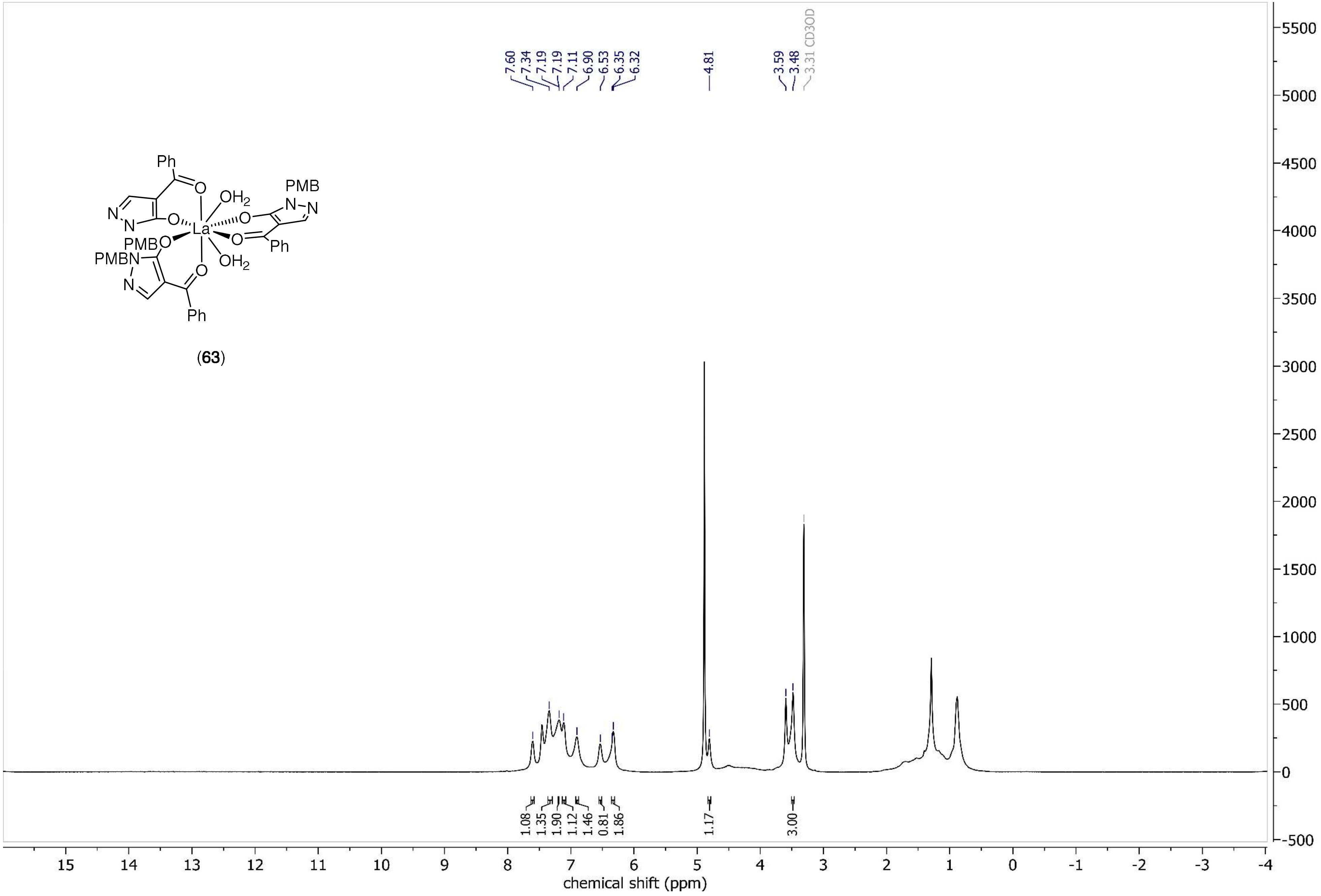

Figure S95: ^{1}H-NMR spectrum of bis(aqua) tris((4-benzoyl-1-(4-methoxybenzyl)-1*H*-pyrazol-5-yl)oxy)lanthanum (**63**).

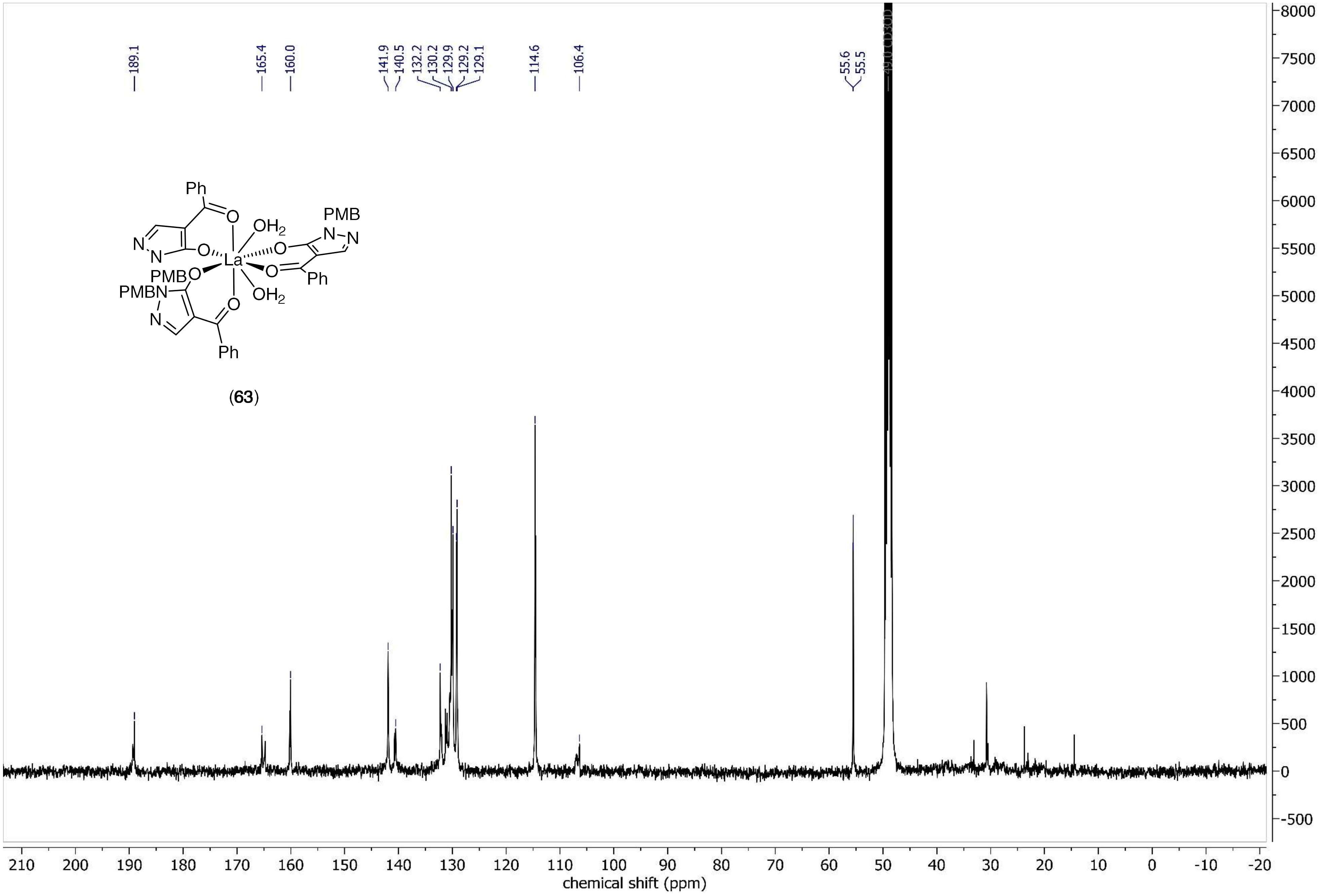

Figure S96: $^{13}C\{^{1}H\}$-NMR spectrum of bis(aqua) tris((4-benzoyl-1-(4-methoxybenzyl)-1*H*-pyrazol-5-yl)oxy)lanthanum (**63**).

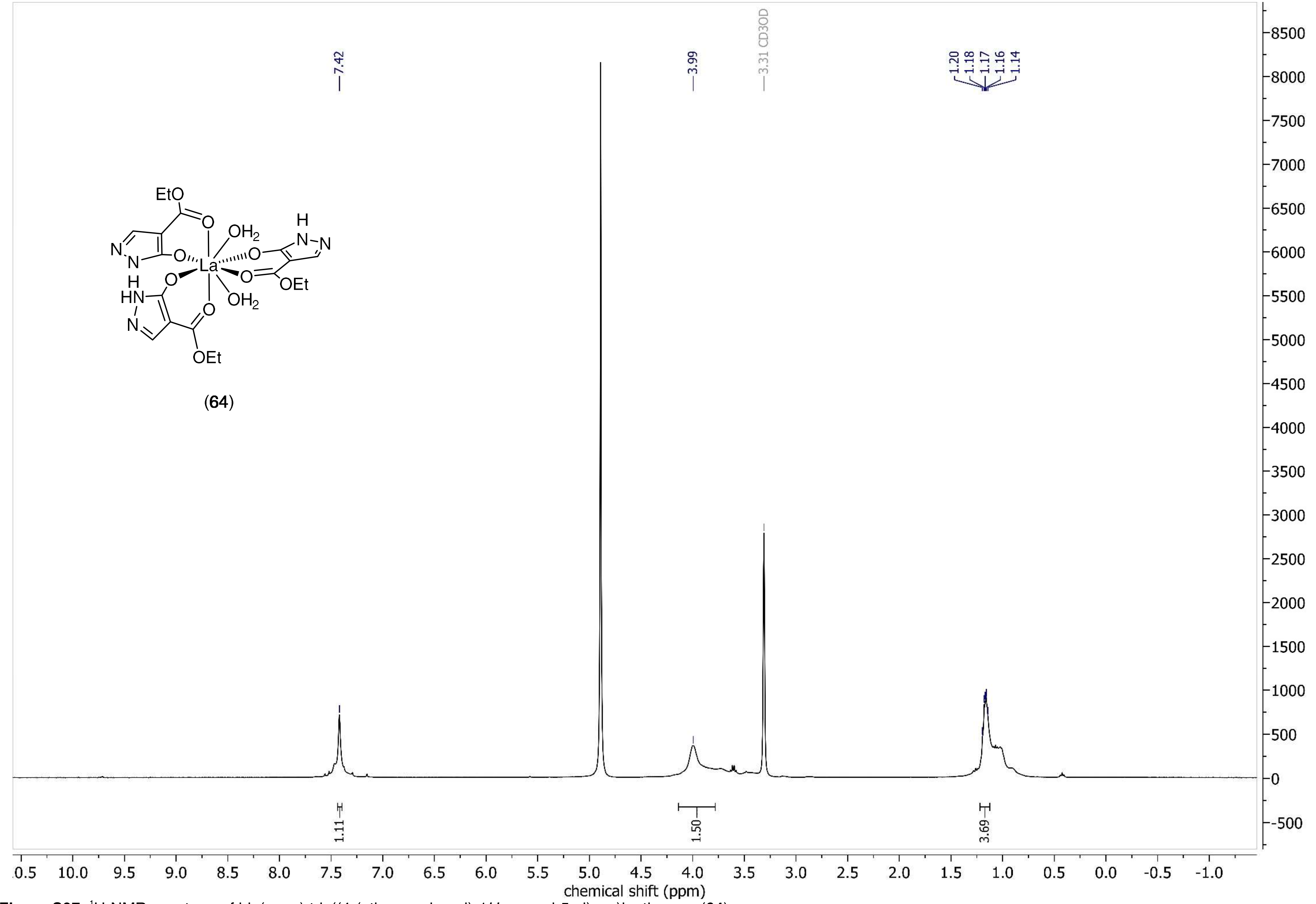

Figure S97: ^{1}H-NMR spectrum of bis(aqua) tris((4-(ethoxycarbonyl)-1*H*-pyrazol-5-yl)oxy)lanthanum (**64**).

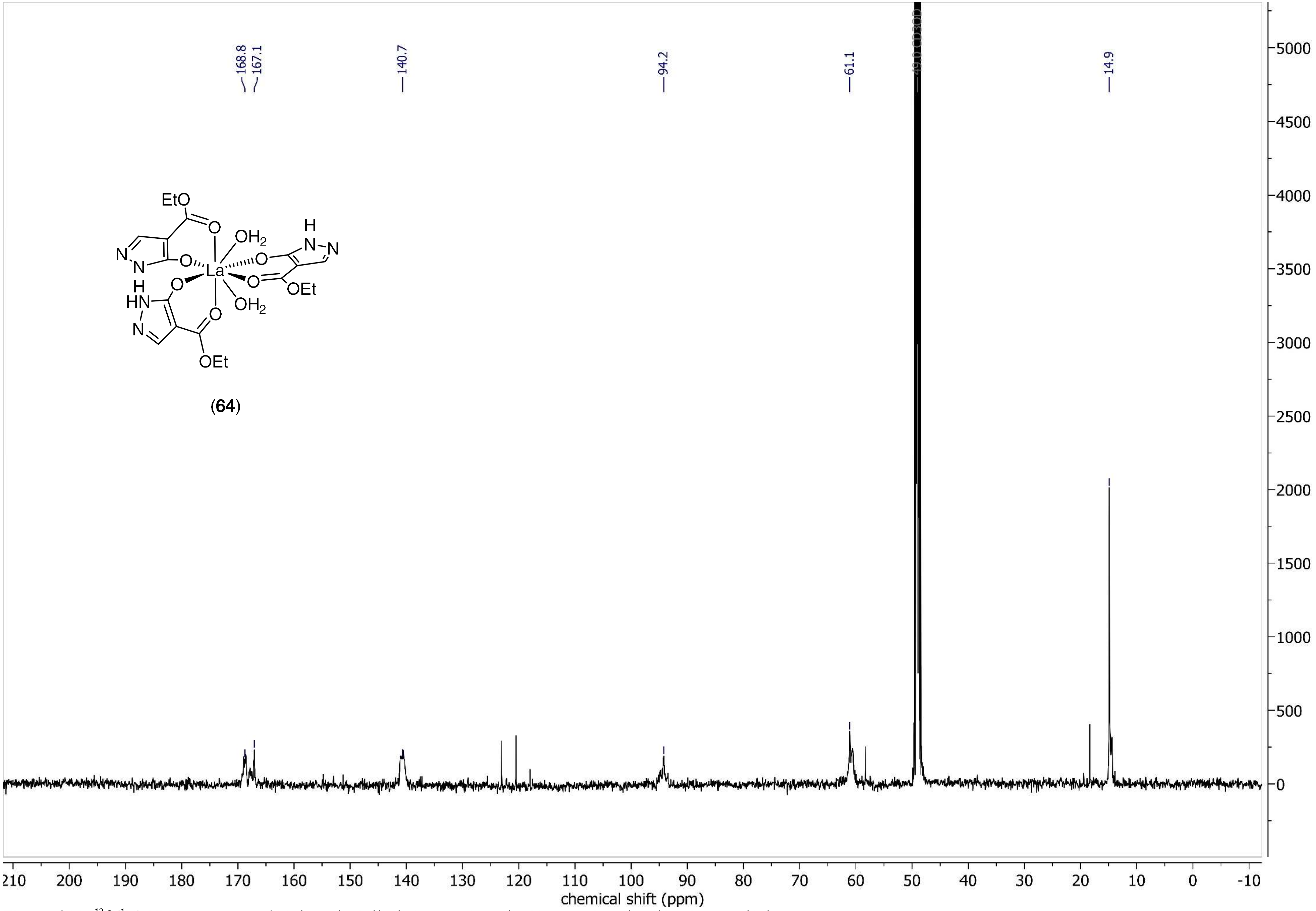

Figure S98: $^{13}C\{^1H\}$-NMR spectrum of bis(aqua) tris((4-(ethoxycarbonyl)-1*H*-pyrazol-5-yl)oxy)lanthanum (**64**).

II. DSC and TG/DTA Measurements

DSC measurements were performed according to the following procedure: ~5-10 mg of sample was weighed into either aluminium pans (Perkin Elmer, UK) or hermetically O-ring sealed stainless-steel pans (Perkin Elmer, UK) and sealed. A Perkin Elmer Diamond DSC was used for all DSC measurements and was calibrated to an Indium standard. Samples were placed into the DSC and referenced against an empty DSC pan, where the difference in power to heat/cool the sample was measured. Heat cycles ranged from -30.0 to 140.0 °C depending on the temperature range of transitions, taking care that samples do not burn/decompose at higher temperatures.
Heating and cooling scans of each sample were repeated a minimum of three times until repeatable data was obtained. Hysteresis was observed between the heating and cooling scans for all samples. In some cases, repeated cycles were performed to understand the mixing of materials. Heating rates varied between 1.0 and 30.0 °C/min. Faster heating/cooling rates caused larger and sharper transitions but with lower accuracy. Slower heating rates are more accurate transitions but at a lower magnitude and may not be observed. The data analysis was performed with Perkin Elmer PYRIS software.

TG/DTA measurements were performed on Netzch Jupiter STA 449C (TA/DTA configuration). The gas flow was 60ml/mins for the instrument during the whole measurement session. The data analysis was performed with Netzsch Proteus software.

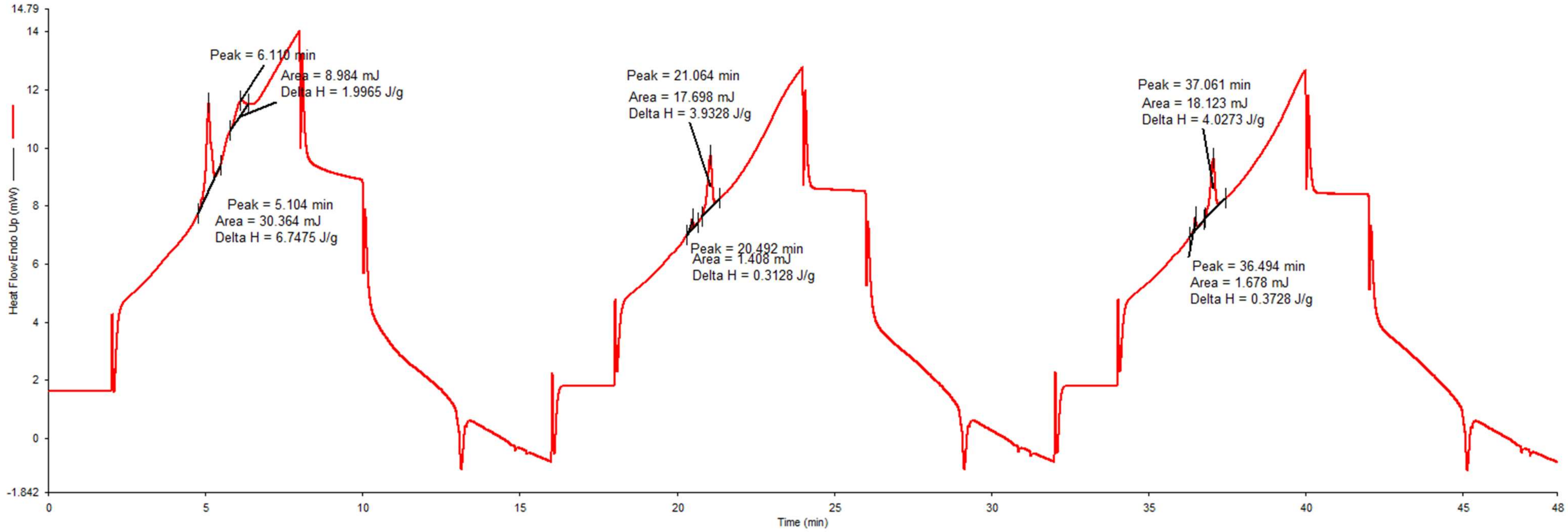

Figure S99: DSC measurement of Na-complex **11**.

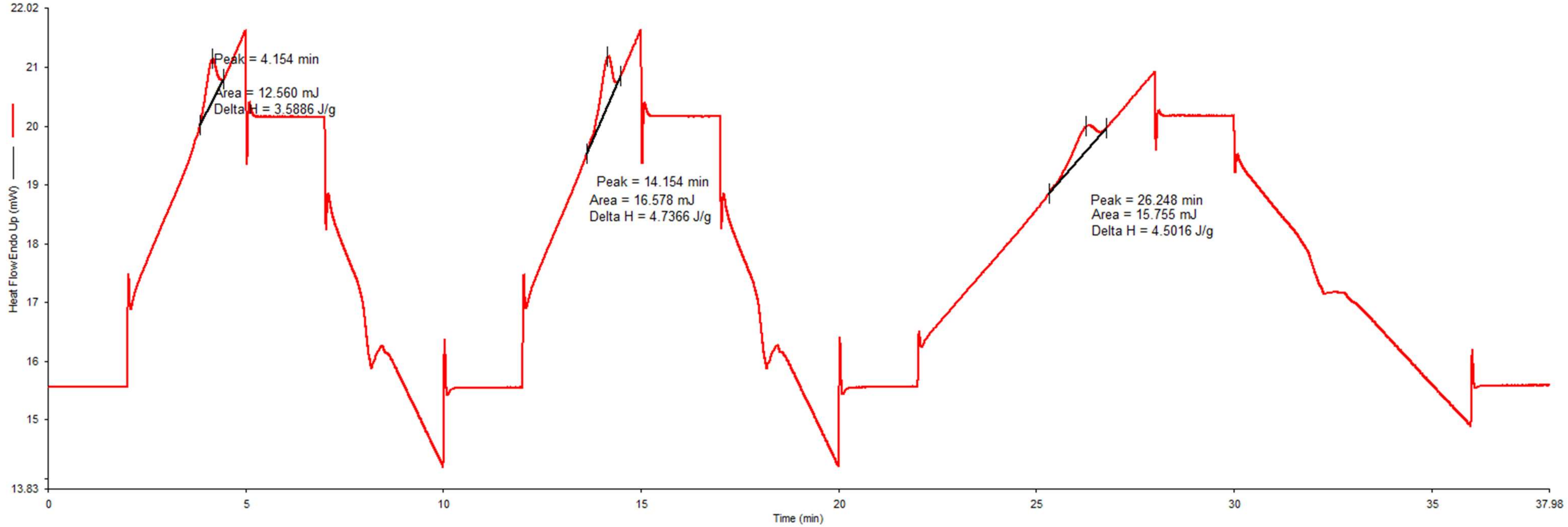

Figure S100: DSC measurement of Na-complex **12**.

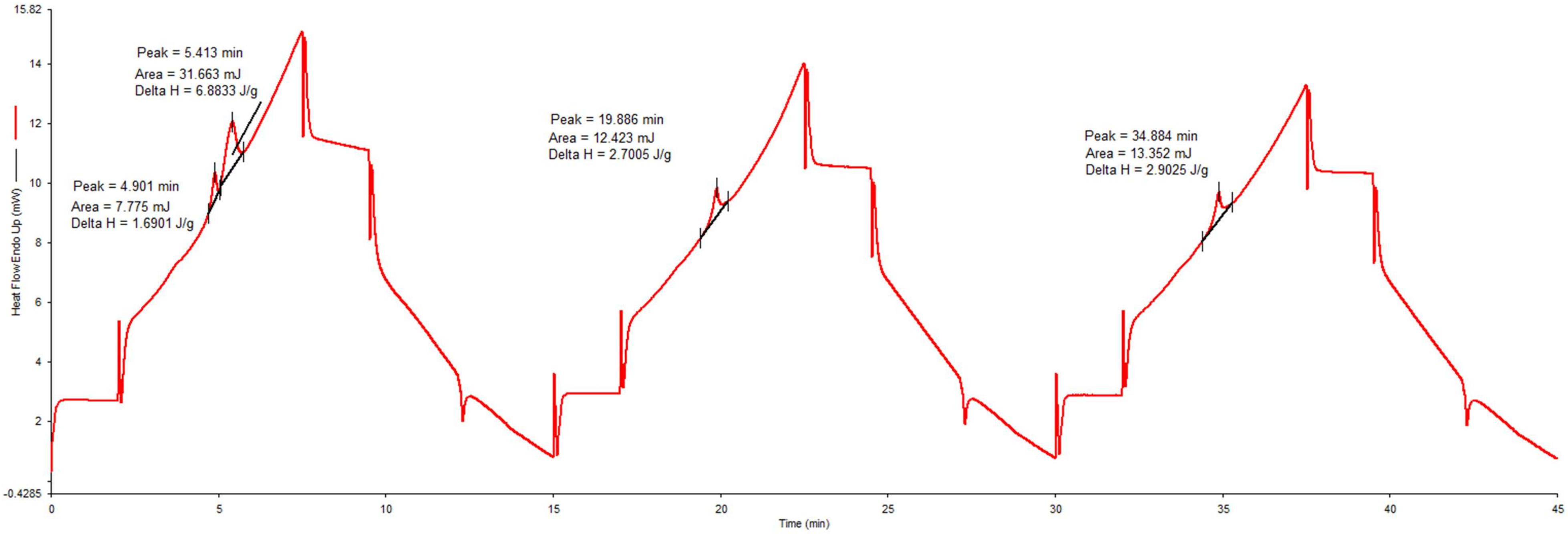

Figure 101: DSC measurement of Na-complex **13**.

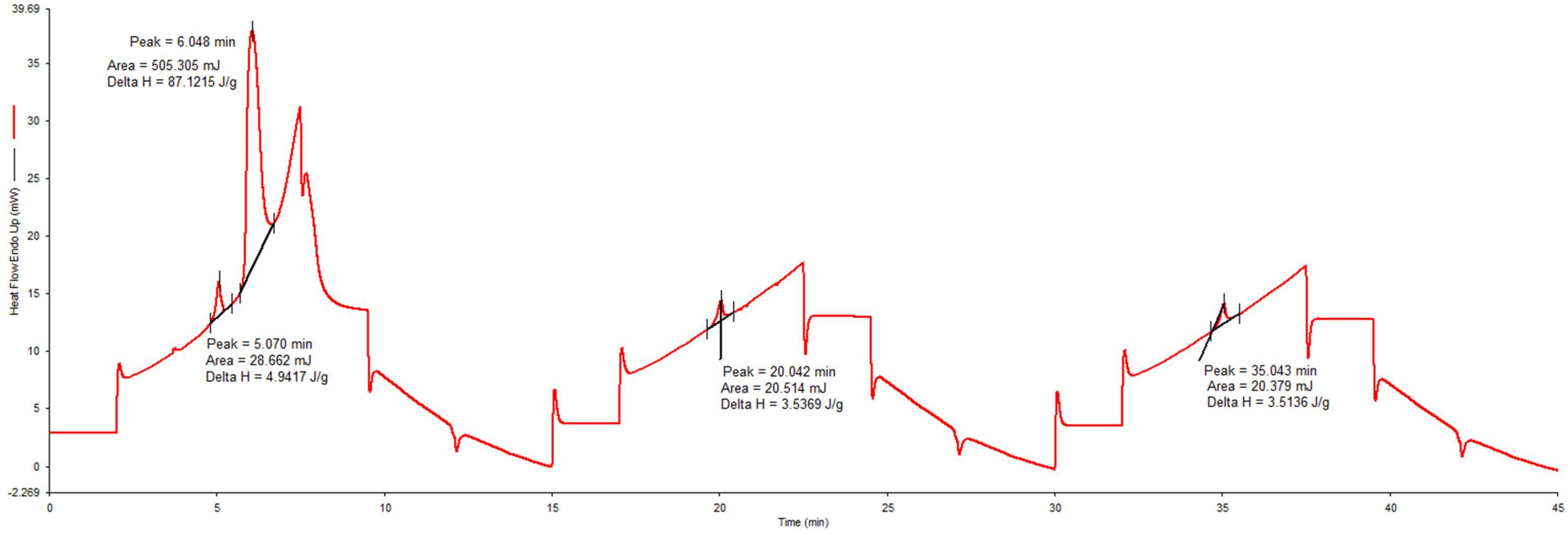

Figure S102: DSC measurement of Na-complex 14.

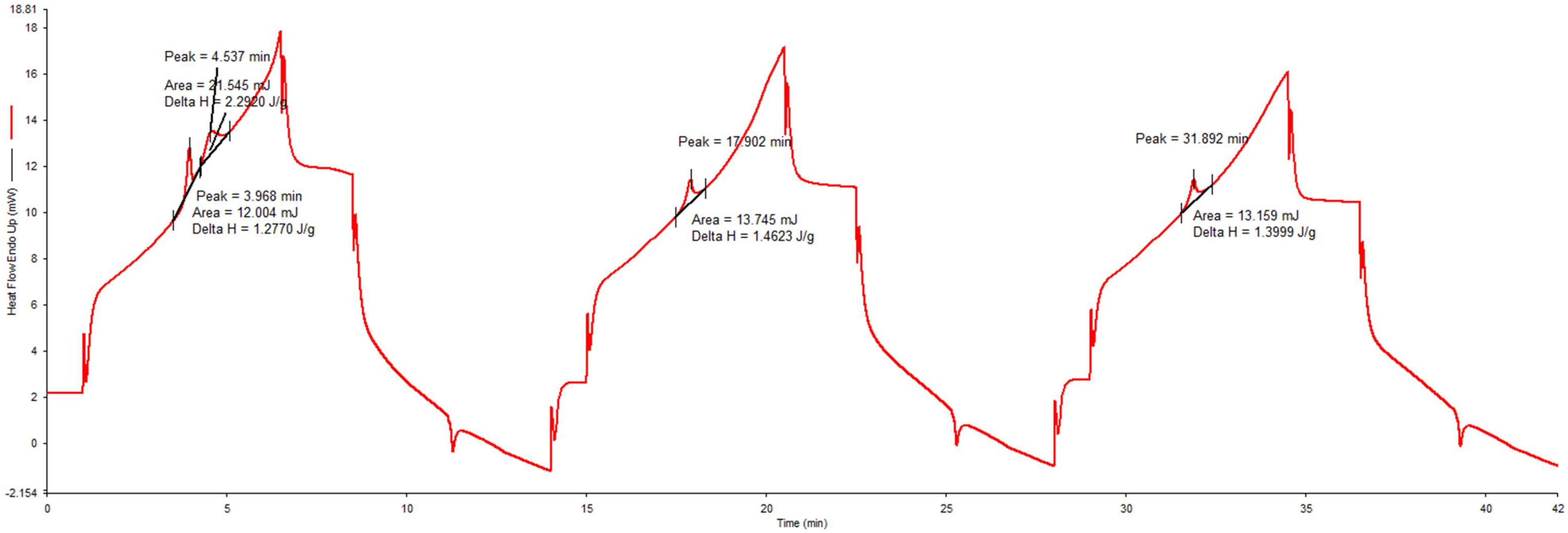

Figure S103: DSC measurement of mixed Na-La complex **61**.

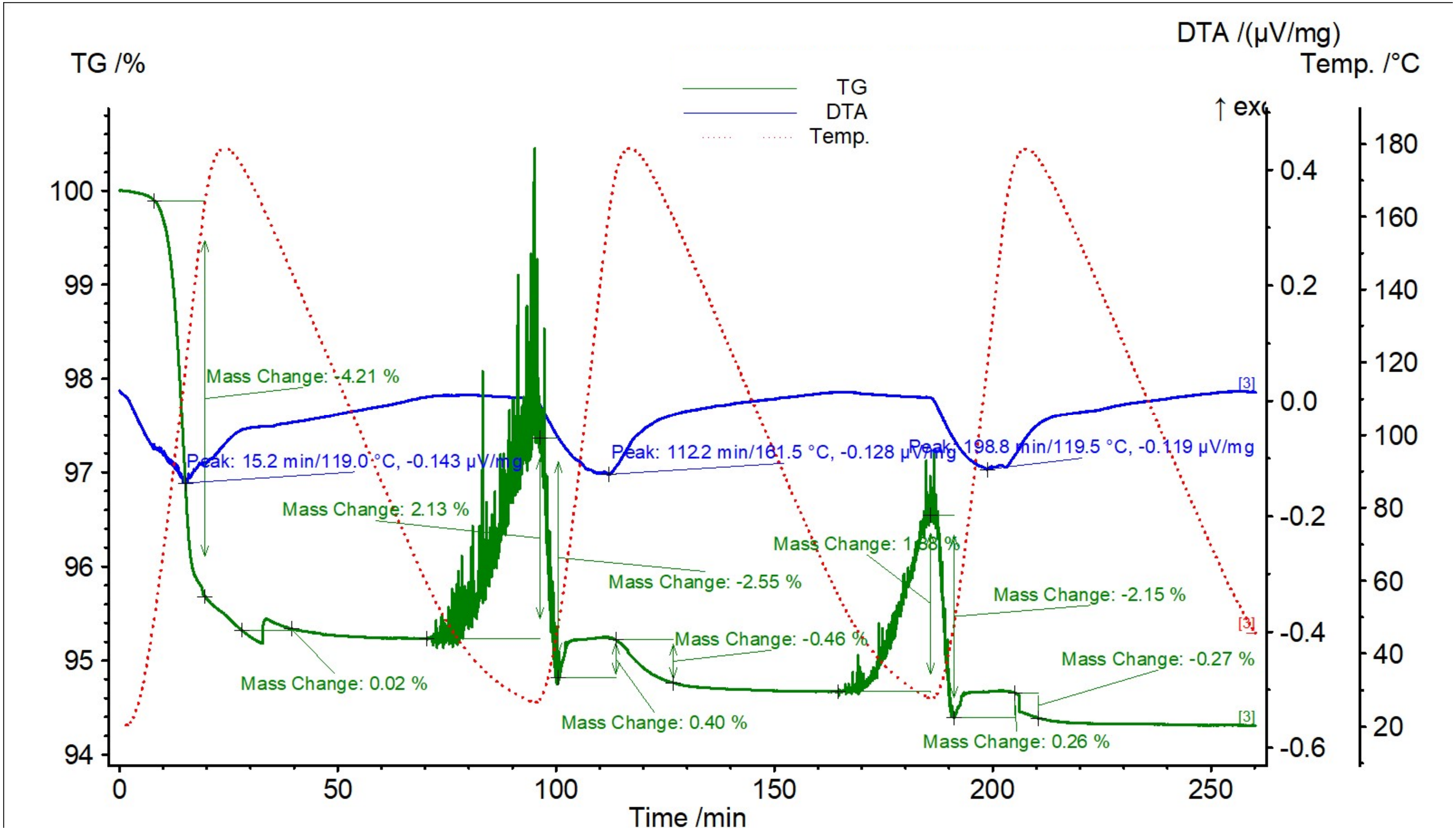

Figure S104: TG/DTA measurement of mixed Na-La complex **61**.

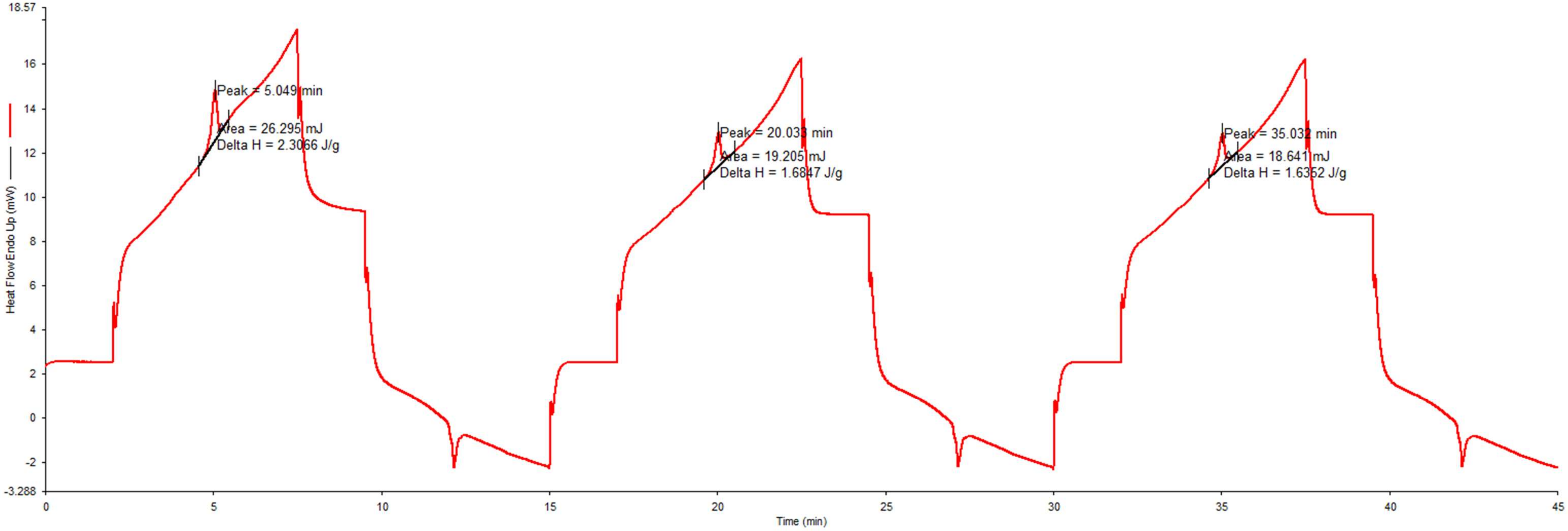

Figure S105: DSC measurement of mixed Na-Dy complex **65**.

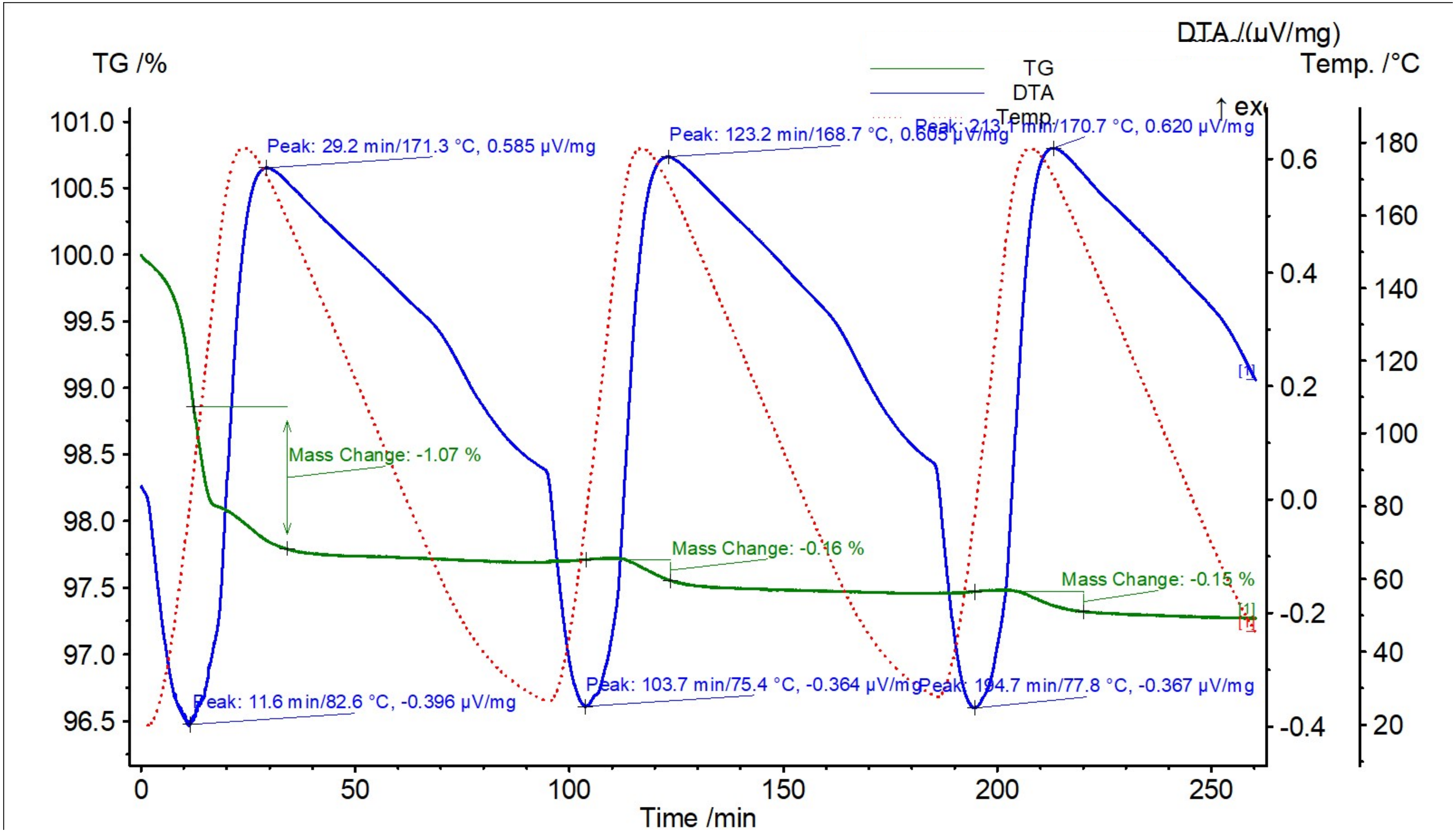

Figure S106: TG/DTA measurement of mixed Na-Dy complex **65**.

III. Computational Calculations

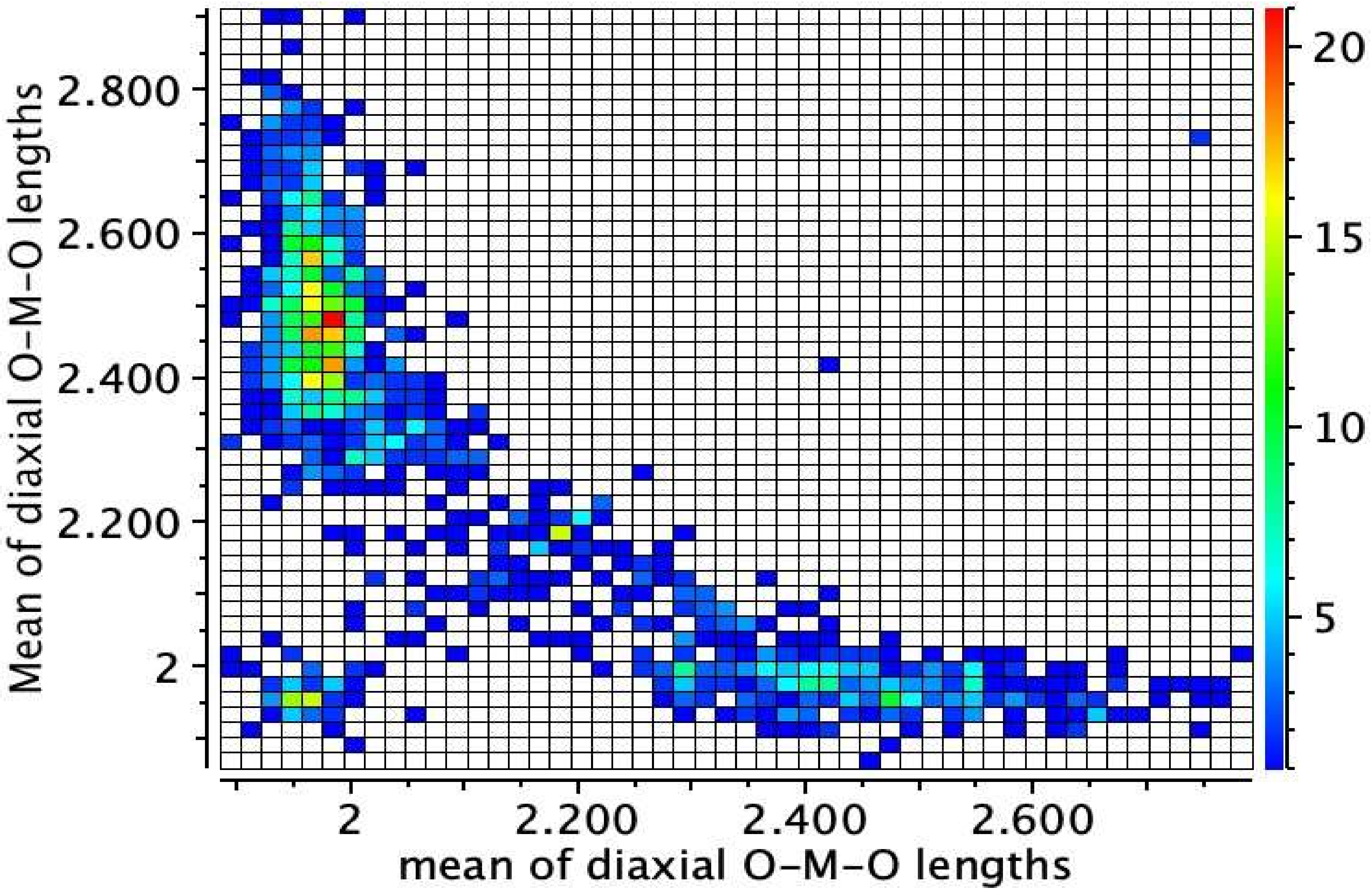

Figure S107: Statistical analysis of crystal structures of octahedral Cu(II) complexes (M = Cu(II)) with average Cu–O relative to Cu–O bonds. Bond length means (Å) for pairs of di-axial bonds for octahedral copper complexes containing four O–Cu and two N–Cu bonds.

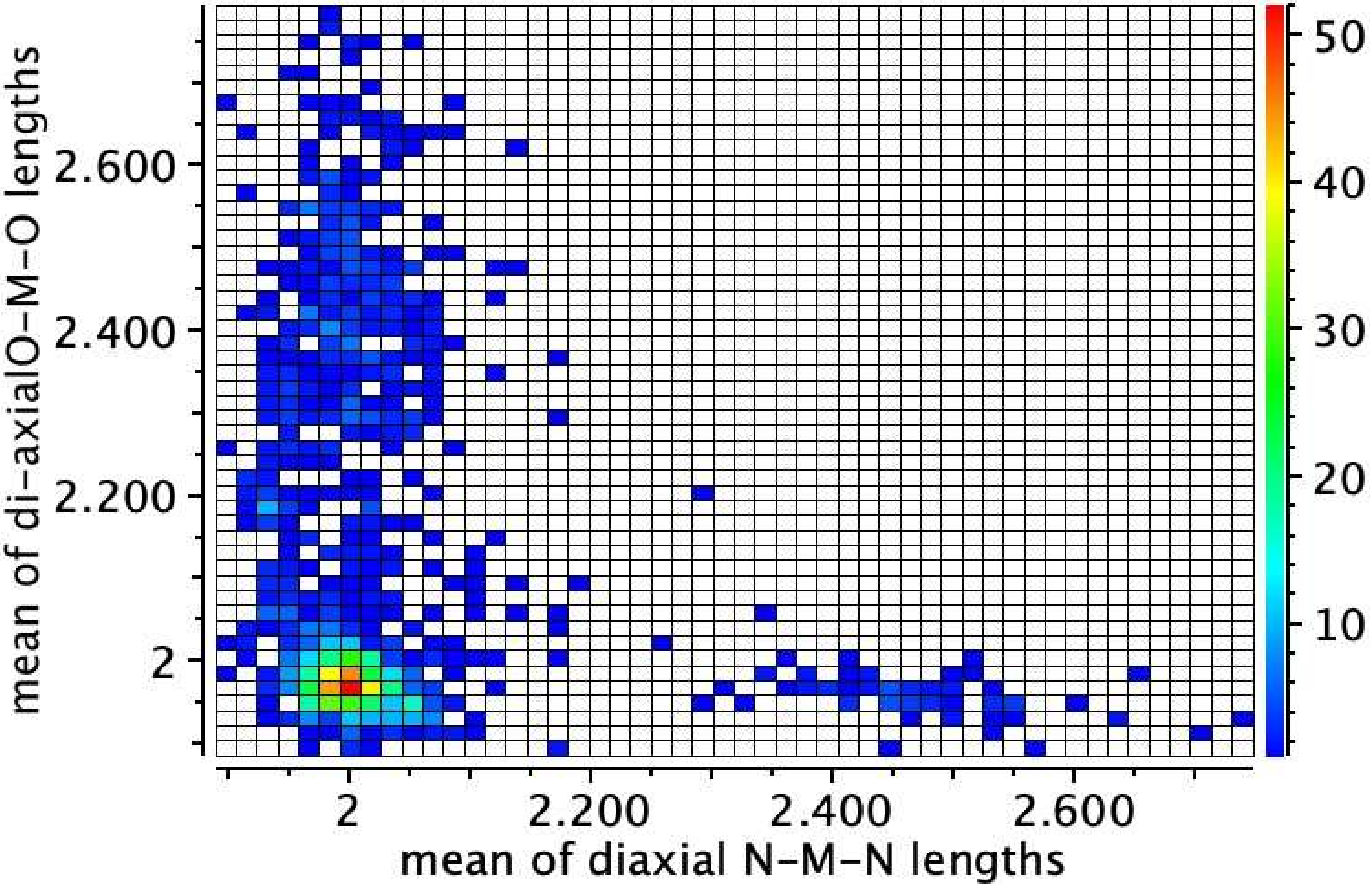

Figure S108: Statistical analysis of crystal structures of octahedral Cu(II) complexes (M = Cu(II)) with average Cu–O relative to Cu–N bonds. Bond length means (Å) for a pair of di-axial O–Cu bonds and a pair of di-axial N–Cu bonds for octahedral copper complexes containing four O–Cu and two N–Cu bonds.

IV. X-Ray Crystallography Data

Table S1. Crystal Data, Data Collection and Refinement Parameters for the structures of **11**, **12**, **14**, **15**, **15b**, **16**, **17**, **18**, **19**, **20**, **21**, **24**, **25**, **28**, **33**, **34**, **37**, **38**, **39**, **39b**, **40**, **42**, **46**, **49**, **50**, **53**, **54**, **55**, **56**, **57**, **58**, **60**, **61**, **62**, **65**, **66**, **70**, **73**, **74, 75** and **76**.

data	11	12	14	15
**formula**	$C_{28}H_{35}N_4NaO_{10}$	$C_{12}H_{13}N_2NaO_4$	$C_{12}H_{15}N_4NaO_6$	$C_{32}H_{42}MgN_4O_{10}$
**solvent**	—	H_2O	—	—
**formula weight**	610.59	290.25	334.27	667.00
**color, habit**	colorless tablets	colorless blocks	colorless needles	colorless blocks
**temperature / K**	173	173	173	173
**crystal system**	monoclinic	trigonal	monoclinic	triclinic
**space group**	*I*2/*a* (no. 15)	*R*3*c* (no. 161)	*I*2/*a* (no. 15)	*P*-1 (no. 2)
a **/ Å**	26.7353(8)	33.8887(5)	11.8935(6)	8.4226(4)
b **/ Å**	4.75334(13)	33.8887(5)	18.1398(9)	10.3319(5)
c **/ Å**	25.8183(9)	6.11875(10)	14.3062(7)	11.1604(5)
**α / deg**	90	90	90	68.692(4)
**β / deg**	113.113(4)	90	104.413(5)	69.160(4)
**γ / deg**	90	120	90	79.405(4)
V **/ Å^3**	3017.68(18)	6085.6(2)	2989.4(3)	843.95(8)
Z	4 [c]	18	8	1 [c]
**D_c / g cm^{-3}**	1.344	1.426	1.485	1.312
**radiation used**	Cu-Kα	Cu-Kα	Cu-Kα	Mo-Kα
**μ / mm^{-1}**	0.982	1.211	1.270	0.114
**no. of unique reflns**				
**measured (R_{int})**	2976 (0.0264)	2091 (0.1048)	2849 (0.0374)	3637 (0.0292)
**obs, $\|F_o\| > 4\sigma(\|F_o\|)$**	2455	2053	2075	2842
**completeness (%)** [a]	99.9	99.8	98.5	99.9
**no. of variables**	211	193	222	221
**R_1(obs), wR_2(all)** [b]	0.0335, 0.0926	0.1332, 0.2918	0.0442, 0.1195	0.0427, 0.1033
**CCDC code**	2210976	2210977	2210978	2210979

[a] Completeness to 0.84 Å resolution. [b] $R_1 = \Sigma||F_o| - |F_c||/\Sigma|F_o|$; $wR_2 = \{\Sigma[w(F_o^2 - F_c^2)^2] / \Sigma[w(F_o^2)^2]\}^{1/2}$; $w^{-1} = \sigma^2(F_o^2) + (aP)^2 + bP$. [c] The complex has crystallographic C_i symmetry. [d] The complex has crystallographic C_2 symmetry. [e] There are two independent C_i-symmetric complexes. [f] The complex has crystallographic D_2 symmetry.

Table S1. …part 2

data	15b	16	17	18
**formula**	$C_{30}H_{38}MgN_4O_{10}$	0.5($C_{28}H_{34}MgN_4O_8$)<br>0.5($C_{24}H_{30}MgN_4O_{10}$)	$C_{40}H_{42}MgN_4O_8$	0.75($C_{16}H_{26}MgN_4O_8$)<br>0.25($C_{14}H_{22}MgN_4O_8$)
**solvent**	—	—	—	—
**formula weight**	638.95	568.86	731.08	419.70
**color, habit**	colorless tabular needles	colorless tablets	colorless needles	colorless blocks
**temperature / K**	173	173	173	173
**crystal system**	triclinic	monoclinic	triclinic	tetragonal
**space group**	*P*-1 (no. 2)	$P2_1/n$ (no. 14)	*P*-1 (no. 2)	$I4_1/a$ (no. 88)
***a* / Å**	8.3930(5)	7.5519(4)	8.6660(10)	16.24728(12)
***b* / Å**	10.5274(6)	8.6190(2)	10.0202(15)	16.24728(12)
***c* / Å**	10.7207(7)	22.3086(9)	10.7952(10)	15.18287(17)
**α / deg**	101.264(5)	90	93.210(10)	90
**β / deg**	112.597(6)	98.225(4)	95.409(9)	90
**γ / deg**	102.885(5)	90	100.868(11)	90
**V / Å^3**	809.87(9)	1437.13(10)	913.89(19)	4007.89(8)
Z	1 [c]	2 [c]	1 [c]	8 [c]
**D_c / g cm^{-3}**	1.310	1.315	1.328	1.391
**radiation used**	Cu-Kα	Cu-Kα	Cu-Kα	Cu-Kα
**μ / mm^{-1}**	0.996	1.029	0.915	1.226
**no. of unique reflns**				
**measured (R_{int})**	3209 (0.0420)	2756 (0.0317)	3461 (0.0485)	2023 (0.0383)
**obs, $\|F_o\| > 4\sigma(\|F_o\|)$**	2390	1896	2553	1843
**completeness (%)** [a]	99.8	98.3	97.9	100
**no. of variables**	220	212	247	148
**R_1(obs), wR_2(all)** [b]	0.0430, 0.1203	0.0673, 0.2249	0.0624, 0.1933	0.0329, 0.0855
**CCDC code**	2210980	2210981	2210982	2211021

Table S1. …part 3

**data**	**19**	**20**	**21**	**24**
**formula**	$C_{32}H_{42}CaN_4O_{10}$	$C_{52}H_{60}Ca_2N_8O_{16}$	$C_{40}H_{42}CaN_4O_8$	$C_{36}H_{35}N_6O_{10}Sc$
**solvent**	—	—	—	—
**formula weight**	682.77	1133.24	746.85	756.66
**color, habit**	yellow tablets	colorless plates	colorless platy needles	colorless needles
**temperature / K**	173	173	173	173
**crystal system**	triclinic	triclinic	triclinic	triclinic
**space group**	*P*-1 (no. 2)	*P*-1 (no. 2)	*P*-1 (no. 2)	*P*-1 (no. 2)
***a* / Å**	8.3922(2)	9.5084(2)	8.9649(7)	11.921(3)
***b* / Å**	10.6222(4)	11.1483(3)	9.8594(7)	12.991(2)
***c* / Å**	11.4899(5)	13.6539(4)	10.9919(6)	13.445(3)
**α / deg**	65.671(4)	79.399(2)	91.671(5)	89.814(15)
**β / deg**	89.753(3)	76.183(2)	93.659(5)	70.778(19)
**γ / deg**	69.888(3)	85.877(2)	101.084(6)	66.000(19)
**V / Å^3**	865.01(6)	1380.89(6)	950.65(11)	1775.3(7)
Z	1 [c]	1 [c]	1 [c]	2
**D_c / g cm^{-3}**	1.311	1.363	1.305	1.416
**radiation used**	Mo-Kα	Mo-Kα	Mo-Kα	Cu-Kα
**μ / mm^{-1}**	0.241	0.282	0.222	2.347
**no. of unique reflns**				
**measured (R_{int})**	3874 (0.0384)	5901 (0.0281)	3772 (0.0232)	6802 (0.1338)
**obs, $\|F_o\| > 4\sigma(\|F_o\|)$**	2972	4820	2690	3141
**completeness (%)** [a]	99.9	99.8	98.8	98.4
**no. of variables**	221	377	247	489
**R_1(obs), wR_2(all)** [b]	0.0424, 0.1027	0.0366, 0.0926	0.0493, 0.1276	0.1035, 0.3325
**CCDC code**	2211022	2211023	2211024	2211025

Table S1. …part 4

data	25	28	33	34
**formula**	$C_{76}H_{70}N_8O_{14}Sc_2$	$C_{38}H_{41}N_6O_{11}Y$	$C_{56}H_{60}N_8O_{16}Zr$	$C_{48}H_{44}N_8O_{12}Zr$
**solvent**	—	—	—	0.75($CHCl_3$)
**formula weight**	1409.32	846.68	1192.34	1105.66
**color, habit**	pale yellow plates	colorless tablets	colorless plates	colorless blocky needles
**temperature / K**	173	173	173	173
**crystal system**	monoclinic	orthorhombic	monoclinic	monoclinic
**space group**	$P2_1/n$ (no. 14)	*Pbca* (no. 61)	*I*2/*a* (no. 15)	*C*2/*c* (no. 15)
a **/ Å**	19.2167(4)	17.14288(10)	22.5461(10)	16.2738(5)
b **/ Å**	9.81529(14)	17.05613(14)	12.3749(5)	19.3360(6)
c **/ Å**	20.1952(4)	26.44498(17)	41.985(2)	16.7882(5)
**α / deg**	90	90	90	90
**β / deg**	114.102(2)	90	99.689(5)	108.415(3)
**γ / deg**	90	90	90	90
V **/ Å**^{3}	3477.08(12)	7732.28(9)	11547.0(10)	5012.2(3)
Z	2 [c]	8	8	4 [d]
D_c **/ g cm**$^{-3}$	1.346	1.455	2.191	1.465
**radiation used**	Cu-Kα	Cu-Kα	Cu-Kα	Mo-Kα
**μ / mm**$^{-1}$	2.273	2.703	2.191	0.409
**no. of unique reflns**				
**measured (**R_{int}**)**	6681 (0.0282)	7798 (0.0485)	11053 (0.0471)	5014 (0.0153)
**obs, $\|F_o\| > 4\sigma(\|F_o\|)$**	5252	6598	9404	4424
**completeness (%)** [a]	98.7	100	98.4	98.7
**no. of variables**	462	518	763	363
R_1**(obs),** wR_2**(all)** [b]	0.0399, 0.1145	0.0407, 0.1203	0.0542, 0.1208	0.0334, 0.0842
**CCDC code**	2211026	2210984	2210985	2210986

Table S1. …part 5

**data**	**37**	**38**	**39**	**39b**
**formula**	$C_{33}H_{35}ClN_4O_8Zr$	$C_{58}H_{54}N_8O_{13}Zr_2$	$C_{36}H_{44}N_4O_{16}Rh_2$	$C_{36}H_{44}N_4O_{16}Rh_2$
**solvent**	—	0.5($C_4H_8O_2$)	—	C_6H_5Cl
**formula weight**	742.32	1297.58	994.57	1107.12
**color, habit**	colorless tabular needles	colorless needles	purple plates	purple tablets
**temperature / K**	173	173	173	173
**crystal system**	monoclinic	tetragonal	triclinic	triclinic
**space group**	*I*2/*a* (no. 15)	*P*4$_2$/*n* (no.86)	*P*-1 (no. 2)	*P*-1 (no. 2)
a **/ Å**	14.0287(2)	16.9933(2)	8.1670(4)	9.1058(3)
b **/ Å**	9.49643(13)	16.9933(2)	8.8751(4)	10.9723(4)
c **/ Å**	24.8679(3)	20.7250(4)	14.4104(6)	12.3084(4)
**α / deg**	90	90	81.575(4)	86.651(3)
**β / deg**	92.3238(13)	90	79.844(4)	88.222(3)
**γ / deg**	90	90	85.308(4)	74.366(3)
V **/ Å^3**	3310.24(9)	5984.8(2)	1015.36(8)	1182.09(7)
Z	4 [d]	4 [d]	1 [c]	1 [c]
D_c **/ g cm^{-3}**	1.489	1.440	1.627	1.555
**radiation used**	Mo-Kα	Cu-Kα	Cu-Kα	Cu-Kα
**μ / mm^{-1}**	0.470	3.454	7.231	6.785
**no. of unique reflns**				
**measured (**R_{int}**)**	3917 (0.0331)	5718 (0.0304)	4035 (0.0488)	4701 (0.0563)
**obs,** $\|F_o\| > 4\sigma(\|F_o\|)$	3583	4125	3375	3943
**completeness (%)** [a]	99.9	98.4	99.9	99.9
**no. of variables**	242	368	278	308
R_1**(obs),** wR_2**(all)** [b]	0.0292, 0.0707	0.0343, 0.0825	0.0331, 0.0737	0.0366, 0.0974
**CCDC code**	2210987	2210988	2210989	2210990

Table S1. …part 6

data	40	42	46	49
formula	$C_{32}H_{36}N_4O_{14}Rh_2$	$C_{28}H_{34}MnN_4O_8$	$C_{36}H_{33}FeN_6O_9$	$C_{28}H_{30}N_4O_8Zn$
solvent	—	—	—	—
formula weight	906.47	609.53	749.53	615.93
color, habit	purple dichroic blocks	colorless tablets	black blocks	colorless tablets
temperature / K	173	173	173	173
crystal system	triclinic	triclinic	triclinic	monoclinic
space group	*P*-1 (no. 2)	*P*-1 (no. 2)	*P*-1 (no. 2)	*C*2/*c* (no. 15)
a / Å	9.4423(6)	9.34441(19)	12.1885(9)	26.4733(6)
b / Å	9.8753(5)	10.5665(6)	12.9190(10)	11.1675(3)
c / Å	11.5911(6)	16.2518(7)	12.9573(8)	9.42187(19)
α / deg	108.310(5)	103.728(4)	61.574(7)	90
β / deg	95.302(5)	96.923(3)	80.293(6)	92.7079(18)
γ / deg	114.600(5)	108.869(4)	78.169(6)	90
V / Å^3	901.23(9)	1440.69(11)	1750.2(2)	2782.39(10)
Z	1 [c]	2	2	4
D_c / g cm^{-3}	1.670	1.405	1.422	1.470
radiation used	Cu-Kα	Mo-Kα	Mo-Kα	Cu-Kα
μ / mm^{-1}	8.039	0.514	0.495	1.718
no. of unique reflns				
measured (R_{int})	3546 (0.0367)	10593 (0.0500)	6885 (0.0275)	2674 (0.0264)
obs, $\|F_o\| > 4\sigma(\|F_o\|)$	2984	7279	4815	2288
completeness (%) [a]	99.4	99.9	98.5	98.5
no. of variables	243	393	482	199
R_1(obs), wR_2(all) [b]	0.0297, 0.0619	0.0468, 0.1314	0.0473, 0.0982	0.0320, 0.0821
CCDC code	2210991	2210992	2210993	2210994

Table S1. …part 7

**data**	**50**	**53**	**54**	**55**
**formula**	$C_{28}H_{34}N_4O_8Zn$	$C_{38}H_{40}CuN_6O_8$	$C_{24}H_{22}CuN_4O_6$	$C_{36}H_{30}CuN_4O_6$
**solvent**	—	—	—	—
**formula weight**	619.96	772.30	525.99	678.18
**color, habit**	colorless blocks	yellow plates	yellow tablets	yellow tablets
**temperature / K**	173	173	173	173
**crystal system**	triclinic	monoclinic	triclinic	orthorhombic
**space group**	*P*-1 (no. 2)	$P2_1/n$ (no. 14)	*P*-1 (no. 2)	$Pca2_1$ (no. 29)
***a* / Å**	9.3541(6)	9.3398(4)	7.3453(5)	8.8955(3)
***b* / Å**	10.6399(7)	9.5425(3)	12.1217(8)	12.1896(3)
***c* / Å**	16.0002(10)	21.4826(18)	14.3414(12)	28.0133(8)
**α / deg**	103.228(6)	90	110.961(7)	90
**β / deg**	97.216(5)	98.861(6)	99.156(6)	90
**γ / deg**	109.679(6)	90	90.661(5)	90
**V / Å^3**	1423.52(17)	1891.78(19)	1173.99(16)	3037.54(15)
Z	2	2 [c]	2 [e]	4
**D_c / g cm^{-3}**	1.446	1.356	1.488	1.483
**radiation used**	Mo-Kα	Cu-Kα	Cu-Kα	Cu-Kα
**μ / mm^{-1}**	0.919	1.309	1.731	1.480
**no. of unique reflns**				
**measured (R_{int})**	9782 (0.0569)	3727 (0.0470)	4468 (0.0360)	4207 (0.0382)
**obs, $\|F_o\| > 4\sigma(\|F_o\|)$**	7193	2503	3168	3544
**completeness (%)** [a]	99.9	99.8	98.3	98.7
**no. of variables**	392	478	321	427
**R_1(obs), wR_2(all)** [b]	0.0679, 0.1910	0.0460, 0.1395	0.0433, 0.1213	0.0407, 0.1058
**CCDC code**	2210995	2210999	2211000	2211001

Table S1. …part 8

data	**56**	**57**	**58**	**60**
**formula**	$C_{22}H_{24}CuN_6O_6$	$C_{38}H_{40}N_6NiO_8$	$C_{34}H_{32}N_6NiO_6$	$C_{22}H_{24}N_6NiO_6$
**solvent**	—	—	—	—
**formula weight**	532.01	767.47	679.36	527.18
**color, habit**	yellow blocks	colorless thin plates	light blue tablets	pale blue canoe-shaped blocks
**temperature / K**	173	173	173	173
**crystal system**	monoclinic	monoclinic	triclinic	monoclinic
**space group**	$P2_1/n$ (no. 14)	$P2_1/n$ (no. 14)	P-1 (no. 2)	$P2_1/c$ (no. 14)
***a* / Å**	11.2026(2)	9.3367(5)	9.7097(8)	9.9278(2)
***b* / Å**	7.77280(10)	9.7167(4)	11.4404(11)	15.0829(4)
***c* / Å**	13.5209(2)	21.086(3)	15.6275(15)	15.8467(4)
**α / deg**	90	90	99.373(8)	90
**β / deg**	103.0985(18)	100.631(9)	94.280(7)	90.7481(19)
**γ / deg**	90	90	104.048(8)	90
**V / Å^3**	1146.71(3)	1880.2(3)	1649.7(3)	2372.68(9)
Z	2 [c]	2 [c]	2	4
**D_c / g cm^{-3}**	1.541	1.356	1.368	1.476
**radiation used**	Cu-Kα	Mo-Kα	Cu-Kα	Cu-Kα
**μ / mm^{-1}**	1.801	0.575	1.297	1.621
**no. of unique reflns**				
**measured (R_{int})**	2304 (0.0455)	3775 (0.0300)	6268 (0.0557)	4522 (0.0268)
**obs, $\|F_o\| > 4\sigma(\|F_o\|)$**	1995	2364	3882	3552
**completeness (%)** [a]	100	98.6	98.3	98.1
**no. of variables**	165	478	434	326
**R_1(obs), wR_2(all)** [b]	0.0325, 0.0909	0.0471, 0.1019	0.0583, 0.1724	0.0400, 0.1189
**CCDC code**	2211002	2211003	2211004	2211005

Table S1. …part 9

**data**	**61**	**62**	**65**	**66**
**formula**	$C_{56}H_{52}LaN_8NaO_{16}$	$C_{38}H_{41}LaN_6O_{11}$	$C_{56}H_{52}DyN_8NaO_{16}$	$C_{36}H_{37}DyN_6O_{11}$
**solvent**	1.25(CH_4O)	—	0.75(CH_4O)	—
**formula weight**	1295.01	896.68	1302.57	892.21
**color, habit**	colorless tablets	colorless blocks	colorless blocks	colorless blocks
**temperature / K**	173	173	173	173
**crystal system**	tetragonal	triclinic	tetragonal	triclinic
**space group**	*P*-4*n*2 (no. 118)	*P*-1 (no. 2)	*P*-4*n*2 (no. 118)	*P*-1 (no. 2)
***a* / Å**	12.8320(7)	10.11619(19)	12.5821(4)	9.6822(3)
***b* / Å**	12.8320(7)	15.2659(3)	12.5821(4)	15.0267(5)
***c* / Å**	18.3558(16)	15.3379(2)	19.0063(10)	15.0409(5)
**α / deg**	90	111.6697(16)	90	110.887(3)
**β / deg**	90	106.9777(15)	90	98.160(3)
**γ / deg**	90	99.5723(15)	90	105.058(3)
**V / Å^3**	3022.5(4)	2002.72(7)	3008.9(2)	1906.44(12)
Z	2 [f]	2	2 [f]	2
**D_c / g cm^{-3}**	1.423	1.487	1.438	1.554
**radiation used**	Mo-Kα	Mo-Kα	Mo-Kα	Mo-Kα
**μ / mm^{-1}**	0.789	1.132	1.323	2.026
**no. of unique reflns**				
**measured (R_{int})**	2889 (0.0295)	9153 (0.0397)	2651 (0.0289)	13501 (0.0346)
**obs, $\|F_o\| > 4\sigma(\|F_o\|)$**	1820	8134	1893	6989
**completeness (%)** [a]	99.3	99.9	99.1	98.3
**no. of variables**	216	529	208	507
**R_1(obs), wR_2(all)** [b]	0.0924, 0.2790	0.0278, 0.0597	0.0473, 0.1321	0.0385, 0.0777
**CCDC code**	2211006	2211007	2211008	2211009

Table S1. …part 10

data	70	73	74	75	76
formula	$C_{38}H_{41}N_6O_{11}Yb$	$C_{30}H_{36}N_4O_{11}U$	$C_{29}H_{27}N_5O_8U$	$C_{41}H_{35}N_5O_8U \cdot C_{37}H_{34}N_4O_9U$	$C_{17}H_{19}N_5O_8U$
solvent	—	H_2O	—	C_5H_5N	—
formula weight	930.81	884.67	811.58	1959.58	659.40
color, habit	colorless tablets	yellow platy needles	yellow plates	orange tablets	light orange plate
temperature / K	173	173	100	173	100
crystal system	orthorhombic	triclinic	triclinic	triclinic	triclinic
space group	*Pbca* (no. 61)	*P*-1 (no. 2)	*P*-1 (no. 2)	*P*-1 (no. 2)	*P*-1 (no. 2)
a **/ Å**	17.0404(2)	9.0575(7)	8.4921(3)	13.7499(7)	9.0978(2)
b **/ Å**	17.0297(3)	9.1343(7)	9.9003(4)	16.0992(6)	9.7058(3)
c **/ Å**	26.4571(3)	23.2937(15)	17.7436(8)	18.3318(11)	12.4838(3)
α / deg	90	79.065(6)	85.718(4)	95.199(4)	76.269(2)
β / deg	90	83.148(6)	79.551(4)	104.557(5)	77.734(2)
γ / deg	90	62.129(8)	78.564(4)	90.954(4)	81.793(2)
V / Å^3	7677.69(18)	1671.7(2)	1436.75(11)	3908.3(4)	1041.43(5)
Z	8	2	2	2	2
D_c / g cm^{-3}	1.611	1.758	1.876	1.665	2.103
radiation used	Cu-Kα	Mo-Kα	Cu-Kα	Mo-Kα	Cu-Kα
μ / mm^{-1}	5.087	4.922	16.415	4.214	22.438
no. of unique reflns					
measured (R_{int})	7629 (0.0341)	7830 (0.0855)	5139 (0.0744)	15451 (0.0563)	3633 (0.0368)
obs, $\|F_o\| > 4\sigma(\|F_o\|)$	6232	4548	4573	8537	3619
completeness (%) [a]	99.9	99.7	98.8	98.4	98.0
no. of variables	518	456	408	1019	282
R_1(obs), wR_2(all) [b]	0.0297, 0.0749	0.0941, 0.2527	0.0603, 0.1671	0.0436, 0.1446	0.0249, 0.0674
CCDC code	2211010	2244132	2250004	2258759	2223051

Table S1 provides a summary of the crystallographic data for the structures of **11**, **12**, **14**, **15**, **15b**, **16**, **17**, **18**, **19**, **20**, **21**, **24**, **25**, **28**, **33**, **34**, **37**, **38**, **39**, **39b**, **40**, **42**, **46**, **49**, **50**, **53**, **54**, **55**, **56**, **57**, **58**, **60**, **61**, **62**, **65**, **66**, **70**, **73**, **74**, **75** and **76**. Data were collected using Agilent Xcalibur PX Ultra A (**11**, **12**, **14**, **15b**, **16**, **17**, **18**, **24**, **25**, **28**, **33**, **38**, **39**, **39b**, **40**, **49**, **53**, **54**, **55**, **56**, **58**, **60** and **70**), Agilent Xcalibur 3 E (**15**, **19**, **20**, **21**, **34**, **37**, **42**, **46**, **50**, **57**, **61**, **62**, **65**, **66**, **73** and **75**), and XtaLAB AFC11 (RCD3) (**74** and **76**) diffractometers, and the structures were solved and refined using the OLEX2,[X1] SHELXTL[X2] and SHELX-2013[X3] program systems.

The absolute structures of **61** and **65** were unambiguously determined by use of the Flack parameter [*x* = –0.014(19) and –0.019(14) respectively], whilst the structures of **12** and **55** were refined as two component inversion twins [Flack parameter *x* = 0.3(6) and 0.38(5) respectively].

The X-ray crystal structure of 11

The structure of **11** was found to sit across a center of symmetry at the sodium atom. The hydrogen atoms of the O20-based water ligand were located from a Δ*F* map and refined freely subject to an O–H distance constraint of 0.90 Å. The N1–H hydrogen atom was also located from a Δ*F* map and refined freely subject to an N–H distance constraint of 0.90 Å, but at 50% occupancy due to being involved in an intermolecular N–H···N hydrogen bond with its counterpart across an independent center of symmetry.

The X-ray crystal structure of 12

Reciprocal space analysis of the data set for the structure of **12** clearly showed the crystal to be twinned, with the initial indexing using only *ca*. 75% of the observed spots. Unfortunately, despite numerous efforts, attempts to model this twinning proved unsatisfactory, with the best results coming from the standard, non-twin, data processing, though this data set in turn was best modelled as resulting from a two component inversion twin [Flack parameter *x* = 0.3(6)]. The O13-bound ethyl group in the structure of **12** was found to be disordered, and two orientations were identified of *ca*. 51 and 49% occupancy. The geometries of the two orientations were optimized, the thermal parameters of adjacent atoms were restrained to be similar, and only the non-hydrogen atoms of the major occupancy orientation were refined anisotropically (those of the minor occupancy orientation were refined isotropically). The four hydrogen atoms of the O20- and O30-based presumed water molecules could not be located from Δ*F* maps and so were omitted, and as a result the atom list for the asymmetric unit is low by 4H (and that for the unit cell low by 72H) compared to what is actually presumed to be present.

The X-ray crystal structure of 14

The N2–H, N11–H and O13–H hydrogen atoms in the structure of **14** were located from Δ*F* maps and refined freely subject to X–H distance constraints of 0.90 Å.

The X-ray crystal structure of 15

The structure of **15** was found to sit across a center of symmetry at the magnesium atom. The O20–H hydrogen atom was located from a Δ*F* map and refined freely subject to an O–H distance constraint of 0.90 Å.

The X-ray crystal structure of 15b

The structure of **15b** was found to sit across a center of symmetry at the magnesium atom. The O16-bound ethyl group was found to be disordered, and two orientations were identified of *ca*. 64 and 36% occupancy. The geometries of the two orientations were optimized, the thermal parameters of adjacent atoms were restrained to be similar, and only the non-hydrogen atoms of the major occupancy orientation were refined anisotropically (those of the minor occupancy orientation were refined isotropically). The O20–H hydrogen atom was located from a Δ*F* map and refined freely subject to an O–H distance constraint of 0.90 Å.

The X-ray crystal structure of 16

The structure of **16** was found to sit across a center of symmetry at the magnesium atom. The O13-bound ethyl group was found to be disordered, and two orientations were identified of *ca*. 65 and 35% occupancy. The geometries of the two orientations were optimized, the thermal parameters of adjacent atoms were restrained to be similar, and only the non-hydrogen atoms of the major occupancy orientation were refined anisotropically (those of the minor occupancy orientation were refined isotropically). The

O20-based coordinated solvent molecule was modelled as a mixture of ethanol and water in a 50:50 ratio, with an additional 50% occupancy included water molecule (based on O30) only being present when the adjacent ligated solvent is the water molecule; all of the non-hydrogen atoms were refined anisotropically. The O20–H hydrogen atom position that is common to both the ethanol and water "variants" (and thus 100% occupancy) was located from a Δ*F* map and refined freely subject to an O–H distance constraint of 0.90 Å. The second hydrogen atom of the coordinated water molecule, as well as both of those of the 50% occupancy included O30-based water molecule, could not be located and so the atom list for the asymmetric unit is low by 1.5H (and that for the unit cell low by 6H) compared to what is actually presumed to be present.

The X-ray crystal structure of 17

The structure of **17** was found to sit across a center of symmetry at the magnesium atom. The O30–H hydrogen atom was located from a Δ*F* map and refined freely subject to an O–H distance constraint of 0.90 Å.

The X-ray crystal structure of 18

The structure of **18** was found to sit across a center of symmetry at the magnesium atom. The O10-based coordinated solvent molecule was modelled as a mixture of ethanol and methanol in a fixed 75:25 ratio with a common oxygen position (when refined freely the occupancies settled at *ca*. 74.4 and 25.6, and so were set to 75:25 for simplicity). The geometries of the two moieties were optimized, the thermal parameters of adjacent atoms were restrained to be similar, and only the non-hydrogen atoms of the major occupancy molecule were refined anisotropically (those of the minor occupancy molecule were refined isotropically). The N2–H and O10–H hydrogen atoms were located from Δ*F* maps and refined freely subject to X–H distance constraints of 0.90 Å.

The X-ray crystal structure of 19

The structure of **19** was found to sit across a center of symmetry at the calcium atom. The O20–H hydrogen atom was located from a Δ*F* map and refined freely subject to an O–H distance constraint of 0.90 Å.

The X-ray crystal structure of 20

The structure of **20** was found to sit across a center of symmetry at the middle of the Ca_2O_2 ring. The O40-bound ethyl group was found to be disordered, and two orientations were identified of *ca*. 85 and 15% occupancy. The geometries of the two orientations were optimized, the thermal parameters of adjacent atoms were restrained to be similar, and only the non-hydrogen atoms of the major occupancy orientation were refined anisotropically (those of the minor occupancy orientation were refined isotropically). The O–H hydrogen atoms of the O40-based ethanol ligand and O50-based included water molecule were located from Δ*F* maps and refined freely subject to O–H distance constraints of 0.90 Å.

The X-ray crystal structure of 21

The structure of **21** was found to sit across a center of symmetry at the calcium atom. The O30–H hydrogen atom was located from a Δ*F* map and refined freely subject to an O–H distance constraint of 0.90 Å.

The X-ray crystal structure of 24

Reciprocal space analysis of the data set for the structure of **24** clearly showed the crystal to be twinned, with the initial indexing using only *ca*. 72% of the observed spots. Unfortunately, despite numerous efforts, attempts to model this twinning proved unsatisfactory, with the best results coming from the standard, non-twin, data processing. Only one (H60A) of the hydrogen atoms of the presumed O60-based water molecule could be located from a Δ*F* map, with the second (H60B) being added in a likely hydrogen bonding position directed towards N21 in an adjacent molecule. Both were refined freely subject to an O–H distance constraint of 0.90 Å.

The X-ray crystal structure of 25

The structure of **25** was found to sit across a center of symmetry at the middle of the Sc_2O_2 ring. The O60-bound ethyl group was found to be disordered, and two orientations were identified of *ca*. 78 and 22% occupancy. The geometries of the two orientations were optimized, the thermal parameters of adjacent atoms were restrained to be similar, and only the non-hydrogen atoms of the major occupancy orientation were refined anisotropically (those of the minor occupancy orientation were refined isotropically).

The X-ray crystal structure of 28

The O–H hydrogen atoms of the O60-and O70-based methanol ligands in the structure of **28** were located from Δ*F* maps and refined freely subject to O–H distance constraints of 0.90 Å.

The X-ray crystal structure of 33

The O16- and O56-bound ethyl groups and the C26-bound *para*-methoxyphenyl moiety in the structure of **33** were found to be disordered, and in each case two orientations were identified, of *ca*. 86:14, 51:49, and 82:18 occupancy respectively. The geometries of each pair of orientations were optimized, the thermal parameters of adjacent atoms were restrained to be similar, and only the non-hydrogen atoms of the major occupancy orientations were refined anisotropically (those of the minor occupancy orientations were refined isotropically).

The X-ray crystal structure of 34

The C40-based included chloroform solvent molecule in the structure of **34** was found to be disordered across a C_2 axis, and two unique orientations were identified of *ca*. 33.8 and 3.7% occupancy — based on the thermal parameters the total occupancy of the unique orientations was set at 37.5% (making the total site occupancy 75%) — with two further orientations of the same occupancies being generated by operation of the C_2 axis. The geometries of the two unique orientations were optimized, the thermal parameters of adjacent atoms were restrained to be similar, and only the chlorine atom of the major occupancy unique orientation was refined anisotropically (the remaining atoms were refined isotropically).

The X-ray crystal structure of 37

The structure of **37** was found to sit across a C_2 axis that passes through the zirconium center and bisects the O3···O3A and O15···O15A vectors. As a consequence the cyclopentadienyl and chlorine positions are inherently disordered, and so the unique sites were refined at 50% occupancy.

The X-ray crystal structure of 38

The structure of **38** was found to sit across a C_2 axis that passes through O50 and bisects the O3···O3A and O23···O23A vectors. The included solvent was found to be highly disordered, and the best approach to handling this diffuse electron density was found to be the SQUEEZE routine of PLATON.[X4] This suggested a total of 110 electrons per unit cell, equivalent to 27.5 electrons per complex. Before the use of SQUEEZE the solvent most resembled ethyl acetate ($C_4H_8O_2$, 48 electrons), and 0.5 ethyl acetate molecules corresponds to 24 electrons, so this was used as the solvent present. As a result, the atom list for the asymmetric unit is low by 0.5 x 0.5($C_4H_8O_2$) = $CH_2O_{0.5}$ (and that for the unit cell low by $C_8H_{16}O_4$) compared to what is actually presumed to be present.

The X-ray crystal structure of 39

The structure of **39** was found to sit across a center of symmetry at the middle of the $Rh_2(OAc)_4$ cluster. The O16-bound ethyl group was found to be disordered, and two orientations were identified of *ca*. 72 and 28% occupancy. The geometries of the two orientations were optimized, the thermal parameters of adjacent atoms were restrained to be similar, and only the non-hydrogen atoms of the major occupancy orientation were refined anisotropically (those of the minor occupancy orientation were refined isotropically). The O3–H hydrogen atom was located from a Δ*F* map and refined freely subject to an O–H distance constraint of 0.90 Å.

The X-ray crystal structure of 39b

The structure of **39b** was found to sit across a center of symmetry at the middle of the $Rh_2(OAc)_4$ cluster. The C41-based included chlorobenzene solvent molecule was found to be disordered across a center of symmetry, and two unique orientations were identified of *ca*. 35 and 15% occupancy (with two further orientations of the same occupancies being generated by operation of the inversion center). The geometries of the two unique orientations were optimized, the thermal parameters of adjacent atoms were restrained to be similar, and only the chlorine atom of the major occupancy orientation was refined anisotropically (the remaining atoms were refined isotropically). The O3–H hydrogen atom was located from a Δ*F* map and refined freely subject to an O–H distance constraint of 0.90 Å.

The X-ray crystal structure of 40

The structure of **40** was found to sit across a center of symmetry at the middle of the $Rh_2(OAc)_4$ cluster. The O3–H hydrogen atom was located from a Δ*F* map and refined freely subject to an O–H distance constraint of 0.90 Å.

The X-ray crystal structure of 42

The crystal of **42** that was studied was found to be a two component twin in a *ca*. 63:37 ratio, with the two lattices related by the approximate twin law [–1.00 0.00 0.00 0.50 0.60 –0.40 –0.50 –1.61 –0.60]. The O40-bound ethyl group was found to be disordered, and two orientations were identified of *ca*. 64 and 36% occupancy. The geometries of the two orientations were optimized, the thermal parameters of adjacent atoms were restrained to be similar, and only the non-hydrogen atoms of the major occupancy orientation were refined anisotropically (those of the minor occupancy orientation were refined isotropically). The O–H hydrogen atoms of the O40- and O50-based ethanol ligands were located from Δ*F* maps and refined freely subject to O–H distance constraints of 0.90 Å.

The X-ray crystal structure of 46

The O33-bound ethyl group in the structure of **46** was found to be disordered, and two orientations were identified of *ca*. 64 and 36% occupancy. The geometries of the two orientations were optimized, the thermal parameters of adjacent atoms were restrained to be similar, and only the non-hydrogen atoms of the major occupancy orientation were refined anisotropically (those of the minor occupancy orientation were refined isotropically).

The X-ray crystal structure of 49

The O16-bound ethyl group in the structure of **49** was found to be disordered, and two orientations were identified of *ca*. 53 and 47% occupancy. The geometries of the two orientations were optimized, the thermal parameters of adjacent atoms were restrained to be similar, and only the non-hydrogen atoms of the major occupancy orientation were refined anisotropically (those of the minor occupancy orientation were refined isotropically).

The X-ray crystal structure of 50

The crystal of **50** that was studied was found to be a two component twin in a *ca*. 51:49 ratio, with the two lattices related by the approximate twin law [–1.00 0.00 0.00 0.51 0.58 –0.42 –0.52 –1.59 –0.58]. The O40-bound ethyl group was found to be disordered, and two orientations were identified of *ca*. 83 and 17% occupancy. The geometries of the two orientations were optimized, the thermal parameters of adjacent atoms were restrained to be similar, and only the non-hydrogen atoms of the major occupancy orientation were refined anisotropically (those of the minor occupancy orientation were refined isotropically). The O40 and O50 O–H hydrogen atoms were located from Δ*F* maps and refined freely subject to O–H distance constraints of 0.90 Å.

The X-ray crystal structure of 53

The whole of the complex in the structure of **53** was found to be disordered across a center of symmetry – refinements with the central copper atom on the special position led to a significant proportion of the atoms having elongated thermal ellipsoids with many adjacent atoms having the major axes of their thermal ellipsoids pointed in the same direction, suggesting a “slippage” disorder. This was modelled using one complete, 50% occupancy, complex slightly displaced from the center of symmetry (*ca*. 0.4 Å), with a

second 50% occupancy orientation being generated by operation of the inversion center. The geometries of the two chelating O,O' ligands are restrained to be similar, (as were a handful of the worst pairs of correlated thermal parameters) and all of the non-hydrogen atoms were refined anisotropically.

The X-ray crystal structure of 54

The structure of **54** was found to contain two crystallographically independent complexes (**54-A** and **54-B**) in the asymmetric unit, each of which sits across a center of symmetry at the copper atom.

The X-ray crystal structure of 55

The structure of **55** was refined as a two component inversion twin [Flack parameter *x* = 0.38(5)].

The X-ray crystal structure of 56

The structure of **56** was found to sit across a center of symmetry at the copper atom. The N2–H hydrogen atom was located from a Δ*F* map and refined freely subject to an N–H distance constraint of 0.90 Å.

The X-ray crystal structure of 57

The whole of the complex in the structure of **57** was found to be disordered across a center of symmetry – refinements with the central nickel atom on the special position led to a significant proportion of the atoms having elongated thermal ellipsoids with many adjacent atoms having the major axes of their thermal ellipsoids pointed in the same direction, suggesting a "slippage" disorder. This was modelled using one complete, 50% occupancy, complex slightly displaced from the center of symmetry (*ca*. 0.3 Å), with a second 50% occupancy orientation being generated by operation of the inversion center. The geometries of the two chelating O,O' ligands are restrained to be similar, a handful of the worst behaving thermal parameters were restrained to be less anisotropic, and all of the non-hydrogen atoms were refined anisotropically.

The X-ray crystal structure of 58

The O13-bound ethyl group in the structure of **58** was found to be disordered, and two orientations were identified of *ca*. 86 and 14% occupancy. The geometries of the two orientations were optimized, the thermal parameters of adjacent atoms were restrained to be similar, and only the non-hydrogen atoms of the major occupancy orientation were refined anisotropically (those of the minor occupancy orientation were refined isotropically).

The X-ray crystal structure of 60

The N2– and N12–H hydrogen atoms in the structure of **60** were located from Δ*F* maps and refined freely subject to an N–H distance constraint of 0.90 Å.

The X-ray crystal structure of 61

The two metal atoms in the structure of **61** were both found to sit on special positions, with the lanthanum atom at a site with D_2 symmetry and the sodium atom at a site with S_4 symmetry. Both the C7-based methoxyphenyl unit and the O15-based ethyl ester group were found to be disordered, and in each case two orientations were identified, of *ca*. 54:46 and 57:43 occupancy respectively. The geometries of each pair of orientations were optimized, the thermal parameters of adjacent atoms were restrained to be similar, and only the non-hydrogen atoms of the major occupancy orientations were refined anisotropically (those of the minor occupancy orientations were refined isotropically). The included solvent was found to be highly disordered, and the best approach to handling this diffuse electron density was found to be the SQUEEZE routine of PLATON.[X4] This suggested a total of 46 electrons per unit cell, equivalent to 23 electrons per lanthanum atom. Before the use of SQUEEZE the solvent was unclear, but most resembled methanol (CH_4O, 18 electrons), and 1.25 methanol molecules corresponds to 22.5 electrons, so this was used as the solvent present. As a result, the atom list for the asymmetric unit is low by 0.25 x 1.25(CH_4O) = $C_{0.31}H_{1.25}O_{0.31}$ (and that for the unit cell low by $C_{2.5}H_{10}O_{2.5}$) compared to what is actually presumed to be present. The absolute structure was unambiguously determined by use of the Flack parameter [*x* = –0.014(19)].

The X-ray crystal structure of 62

The O60-bound ethyl group in the structure of **62** was found to be disordered, and two orientations were identified of *ca*. 68 and 32% occupancy. The geometries of the two orientations were optimized, the thermal parameters of adjacent atoms were restrained to be similar, and only the non-hydrogen atoms of the major occupancy orientation were refined anisotropically (those of the minor occupancy orientation were refined isotropically). The O–H hydrogen atoms of the O60-based ethanol ligand and O70-based included water molecule were all located from Δ*F* maps and refined freely subject to O–H distance constraints of 0.90 Å.

The X-ray crystal structure of 65

The two metal atoms in the structure of **65** were both found to sit on special positions, with the dysprosium atom at a site with D_2 symmetry and the sodium atom at a site with S_4 symmetry. Both the C7-based methoxyphenyl unit and the O16-based ethoxide group were found to be disordered, and in each case two orientations were identified, of *ca*. 83:17 and 63:37 occupancy respectively. The geometries of each pair of orientations were optimized, the thermal parameters of adjacent atoms were restrained to be similar, and only the non-hydrogen atoms of the major occupancy orientations were refined anisotropically (those of the minor occupancy orientations were refined isotropically). The included solvent was found to be highly disordered, and the best approach to handling this diffuse electron density was found to be the SQUEEZE routine of PLATON.[X4] This suggested a total of 26 electrons per unit cell, equivalent to 13 electrons per dysprosium atom. Before the use of SQUEEZE the solvent was unclear, but most resembled methanol (CH_4O, 18 electrons), and 0.75 methanol molecules corresponds to 13.5 electrons, so this was used as the solvent present. As a result, the atom list for the asymmetric unit is low by 0.25 x 0.75(CH_4O) = $C_{0.19}H_{0.75}O_{0.19}$ (and that for the unit cell low by $C_{1.5}H_6O_{1.5}$) compared to what is actually presumed to be present. The absolute structure was unambiguously determined by use of the Flack parameter [x = –0.019(14)].

The X-ray crystal structure of 66

The crystal of **66** that was studied was found to be a two component twin in a *ca*. 95:5 ratio, with the two lattices related by the approximate twin law [0.92 –0.05 –0.12 0.02 1.01 0.02 0.25 0.02 1.04]. The N42-bound phenyl ring was found to be disordered, and two orientations were identified of *ca*. 73 and 27% occupancy. The geometries of the two orientations were idealized, the thermal parameters of adjacent atoms were restrained to be similar, and only the non-hydrogen atoms of the major occupancy orientation were refined anisotropically (those of the minor occupancy orientation were refined isotropically). Three of the four presumed hydrogen atoms of the O60- and O70-based coordinated water molecules were located from Δ*F* maps and refined freely subject to O–H distance constraints of 0.90 Å. The fourth, H60B, could not be reliably located and so was added in a likely hydrogen bonding position directed towards O23 and refined with O–H and Dy···H distance constraints (to keep a sensible geometry) and with a riding thermal parameter.

The X-ray crystal structure of 70

The O–H hydrogen atoms of the O60-and O70-based methanol ligands in the structure of **70** were located from Δ*F* maps and refined freely subject to O–H distance constraints of 0.90 Å.

The X-ray crystal structure of 73

The crystal of **73** that was studied was found to be severely twinned. Although the best results came from using a two component twin model in a *ca*. 50:50 ratio with the two lattices related by the approximate twin law [1.00 0.02 –0.05 0.00 1.01 –0.03 0.30 0.03 0.98], between them they still only used *ca*. 44% of the spots, so the lowered precision of the final results is hardly a surprise. The C7- and C27-based *p*-methoxyphenyl groups, and the O40-based coordinated ethanol solvent molecule, were all found to be disordered. In each case two orientations were identified, of *ca*. 59:41, 58:42 and 55:45% occupancy respectively, the geometries of the two orientations were idealized, the thermal parameters of adjacent atoms were restrained to be similar, and only the non-hydrogen atoms of the major occupancy orientations were refined anisotropically (those of the minor occupancy orientation were refined isotropically). The three hydrogen atoms of the O40-based ethanol ligand and O70-based water molecule could not be located from Δ*F* maps and so were omitted, and as a result the atom list for the asymmetric unit is low by 3H (and that for the unit cell low by 6H) compared to what is actually presumed to be present.

The X-ray crystal structure of 74

The crystal of **74** that was studied was found to be severely twinned. Although the best results came from not modelling any twinning, this used only *ca*. 31% of the spots, so the lowered precision of the final results is hardly a surprise. Unfortunately a hardware malfunction meant that a movie of the crystal was not measured, and this was not noticed until well after the crystal had been removed from the diffractometer and discarded. This made absorption correction a tricky issue – the preferred face-indexing approach could not be used due to the lack of the movie, and a spherical absorption correction was applied using a radius chosen to give the best results rather than one that might have matched the unknown size of the actual crystal studied. Combined with the very severe twinning, it is not surprising that the magnitude of the final residual electron density background (both positive and negative) is rather large (at *ca*. 3 eÅ^{-3}). However, despite these issues, the identity of the compound, and the arrangement of the ligands around the central metal atom, are entirely clear.

The C12-bound ethoxy group (and C12 itself) was found to be disordered, and two orientations were identified of *ca*. 51 and 49% occupancy. The geometries of the two orientations were idealized, the thermal parameters of adjacent atoms were restrained to be similar, and only the non-hydrogen atoms of the major occupancy orientation were refined anisotropically (those of the minor occupancy orientation were refined isotropically).

The X-ray crystal structure of 75

The crystal of **75** that was studied was found to be a two component twin in a *ca*. 62:38 ratio, with the two lattices related by the approximate twin law [1.01 0.00 0.01 –0.01 1.00 –0.01 –0.02 0.00 0.99]. The structure was found to contain two distinct complexes that differ in the coordinated solvent molecule with complex **75-A** having a coordinated pyridine whereas complex **75-B** has a coordinated methanol. The O60–H hydrogen atom was located from a Δ*F* map and refined freely subject to an O–H distance constraint of 0.90 Å.

The X-ray crystal structure of 76

The N–H hydrogen atoms on N1 and N3 in the structure of **76** were added in idealized positions and allowed to ride on their parent atoms with an N–H distance of 0.88 Å (the SHELXL AFIX 43 command).

V. References

[X1] Dolomanov, O.V.; Bourhis, L.J.; Gildea, R.J.; Howard, J.A.K.; Puschmann, H. *OLEX2*: a complete structure solution, refinement and analysis program. *J. Appl. Cryst.* 2009, **42**, 339-341.
[X2] SHELXTL v5.1, Bruker AXS, Madison, WI, 1998.
[X3] SHELX-2013, Sheldrick, G.M. Crystal structure refinement with *SHELXL*. *Acta Cryst.* 2015, **C71**, 3-8.
[X4] Spek, A.L. (2003, 2009) PLATON, A Multipurpose Crystallographic Tool, Utrecht University, Utrecht, The Netherlands. See also Spek, A.L. *PLATON SQUEEZE*: a tool for the calculation of the disordered solvent contribution to the calculated structure factors. *Acta. Cryst.* 2015, **C71**, 9-18.

VI. X-Ray Figures

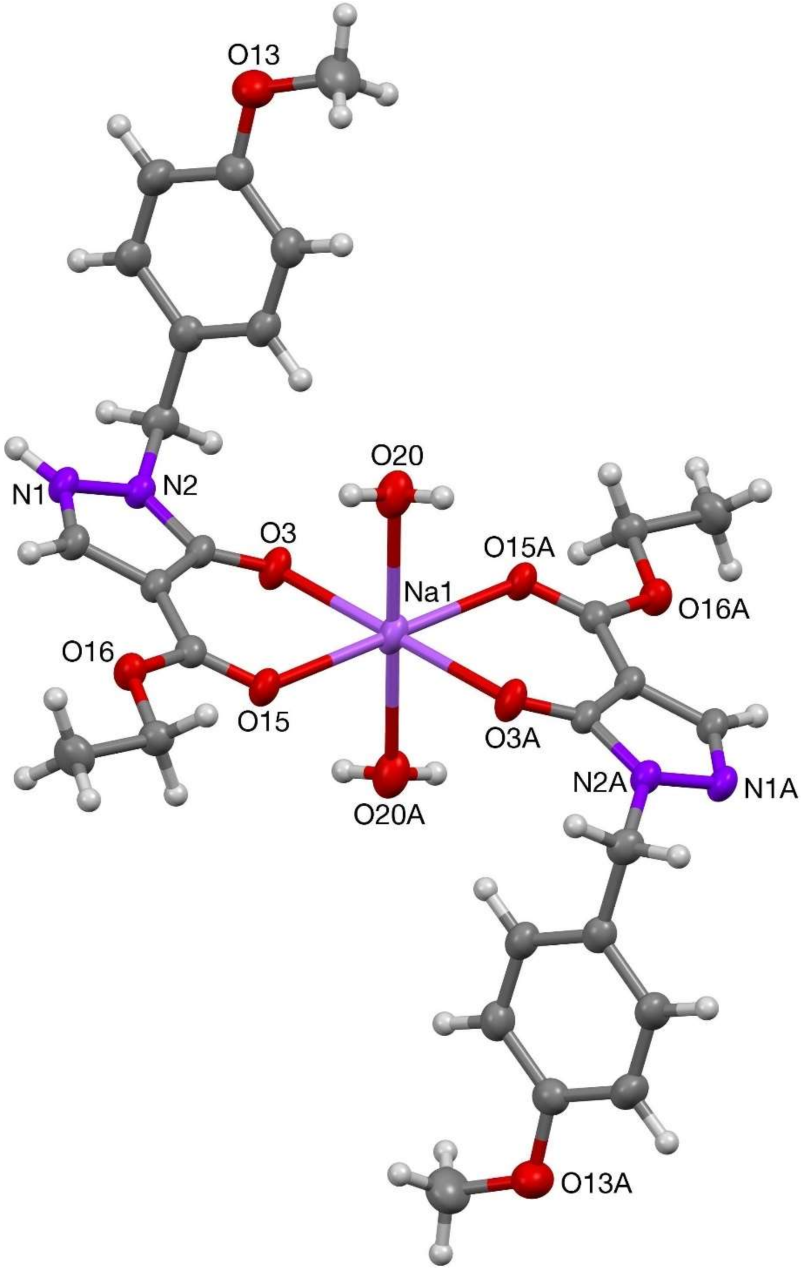

Figure S109: The crystal structure of the C_i-symmetric complex **11** (50% probability ellipsoids).

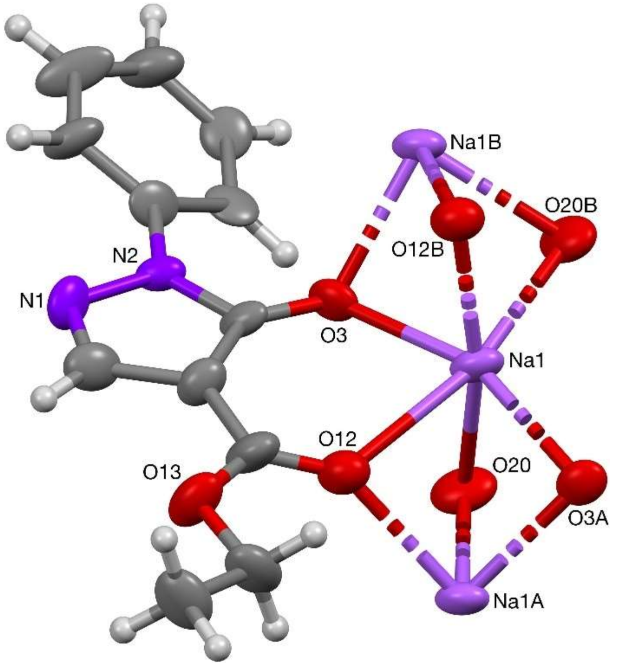

Figure S110: The structure of the asymmetric unit present in the crystal of **12** (50% probability ellipsoids).

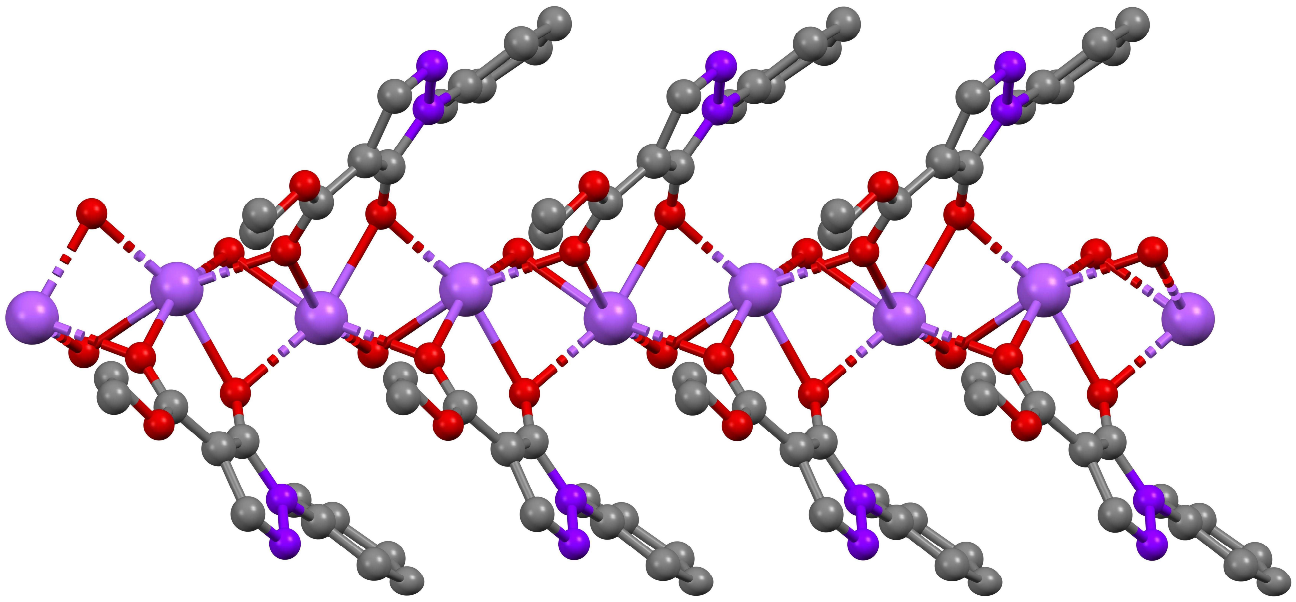

Figure S111:Part of one of the extended chains present in the crystal structure of **12**.

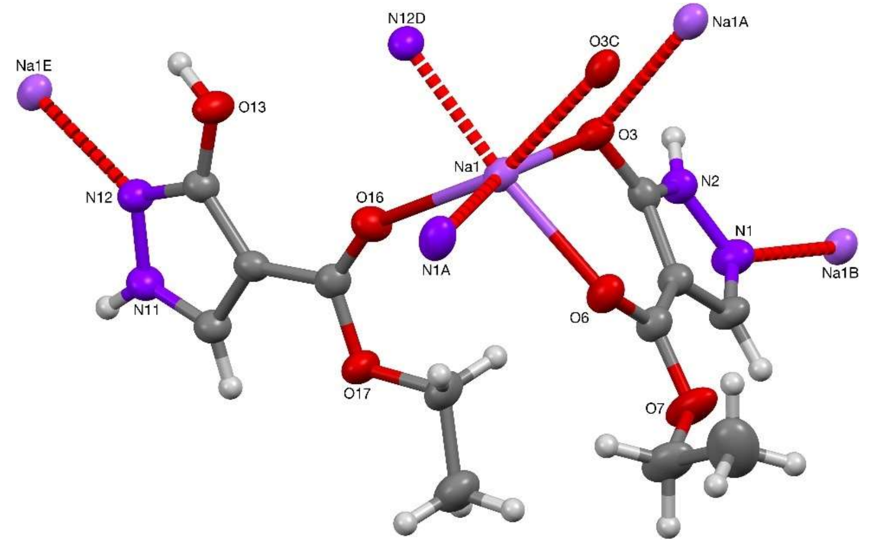

Figure S112: The structure of the asymmetric unit present in the crystal of **14** (50% probability ellipsoids).

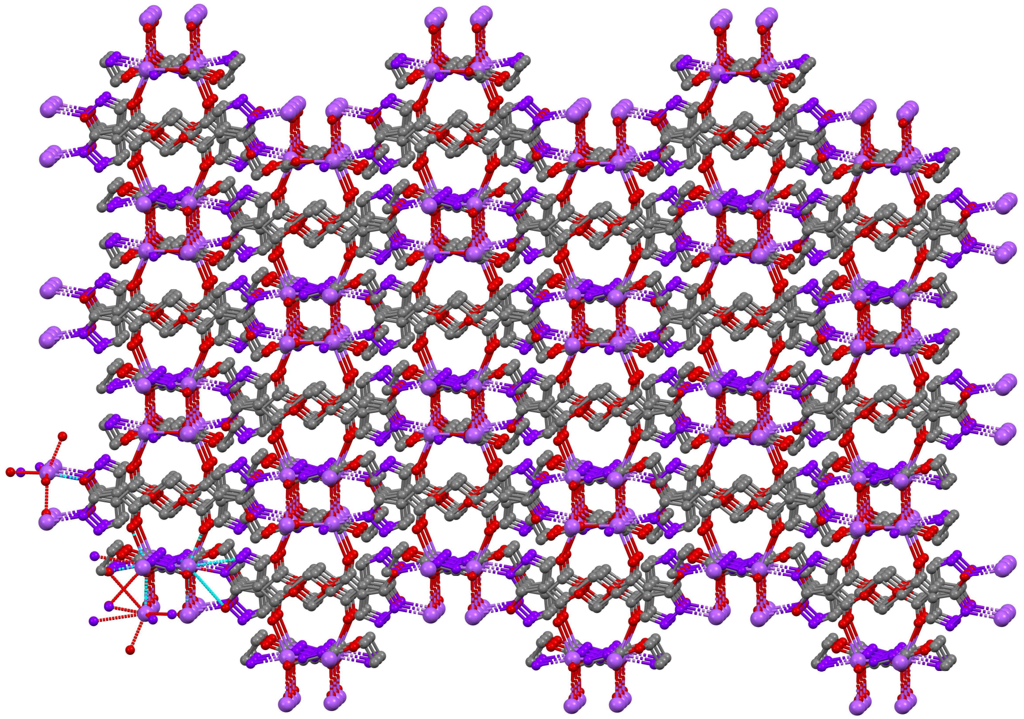

Figure S113: Part of the 3D polymer network present in the crystal structure of **14**.

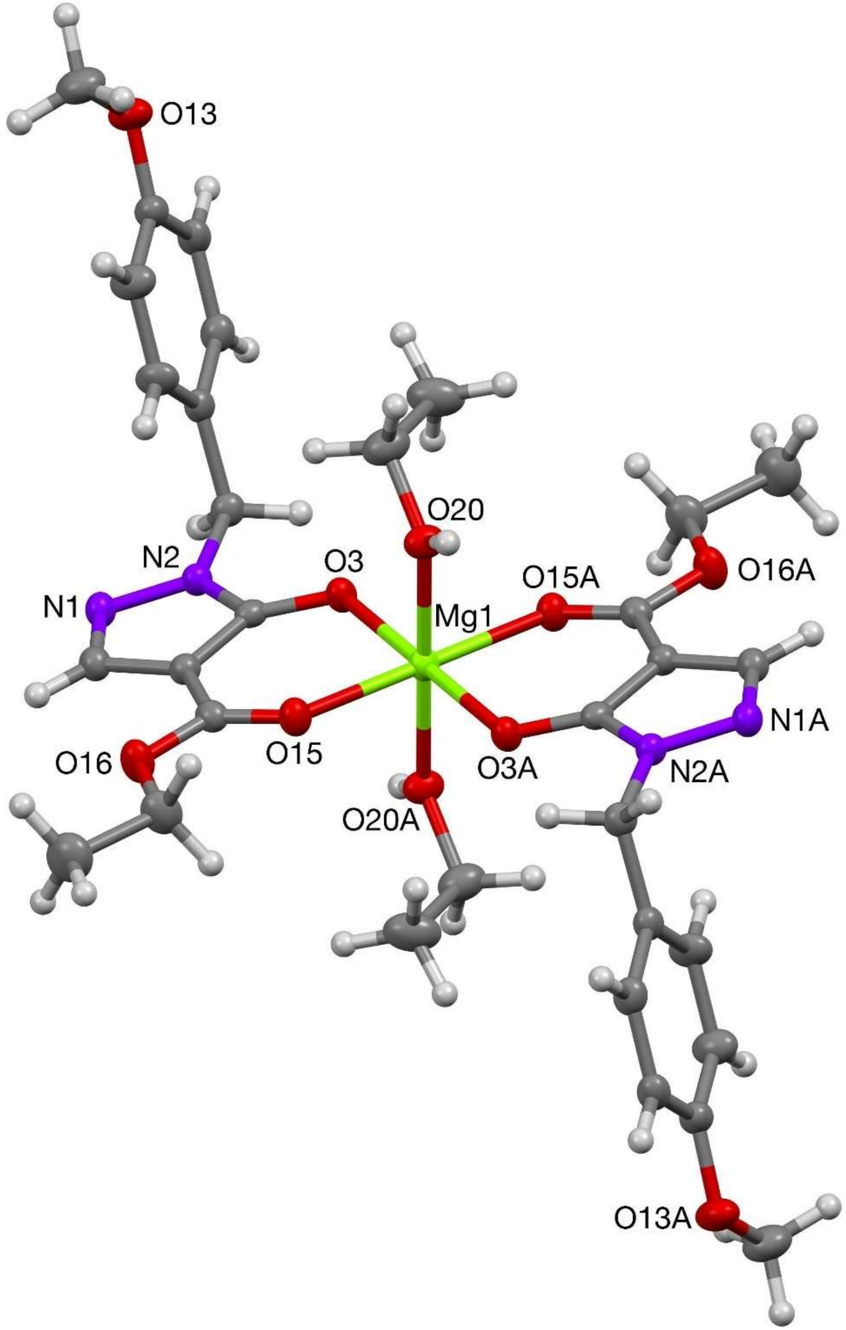

Figure S114: The crystal structure of the C_i-symmetric complex **15** (50% probability ellipsoids).

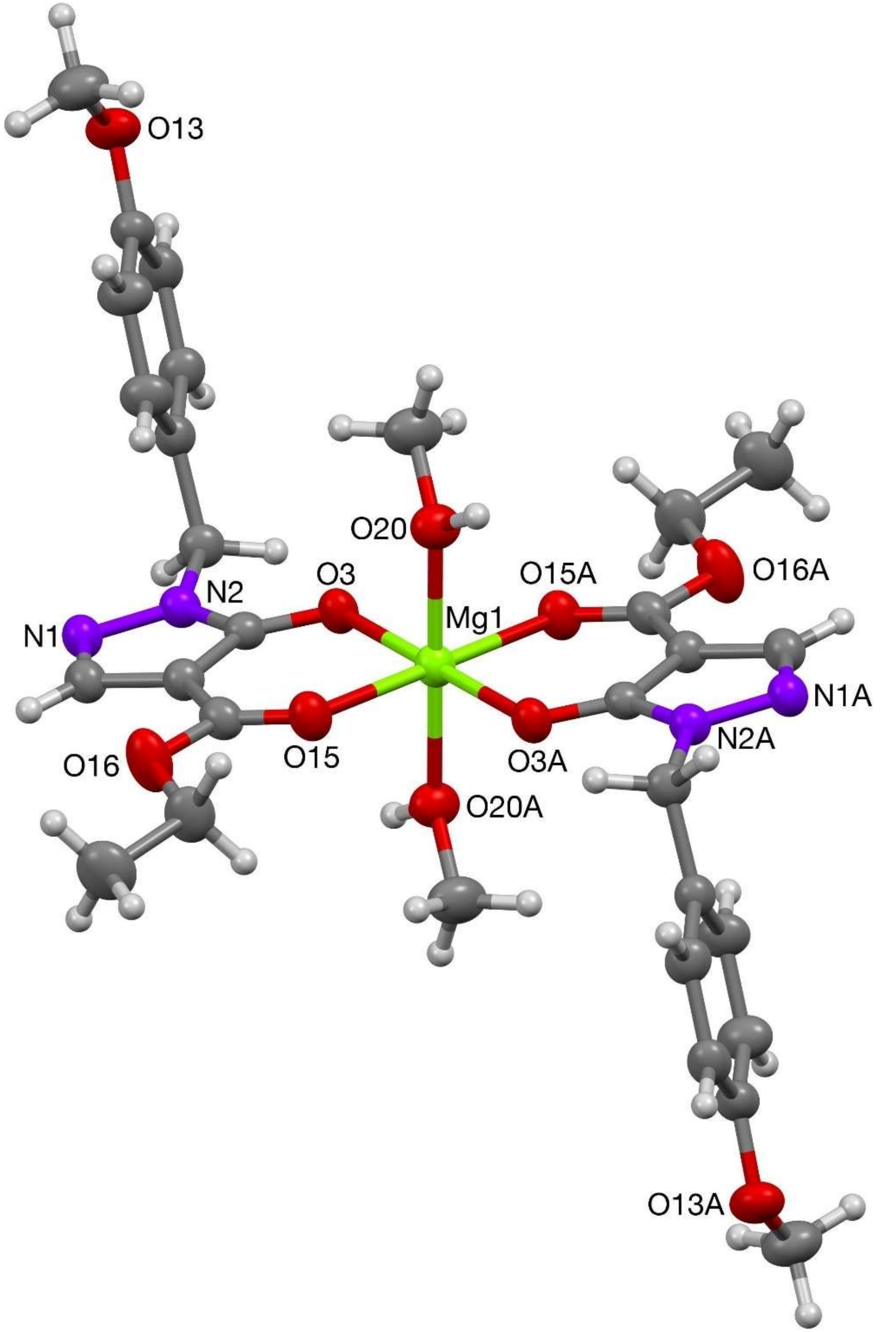

Figure S115: The crystal structure of the C_i-symmetric complex **15** (50% probability ellipsoids).

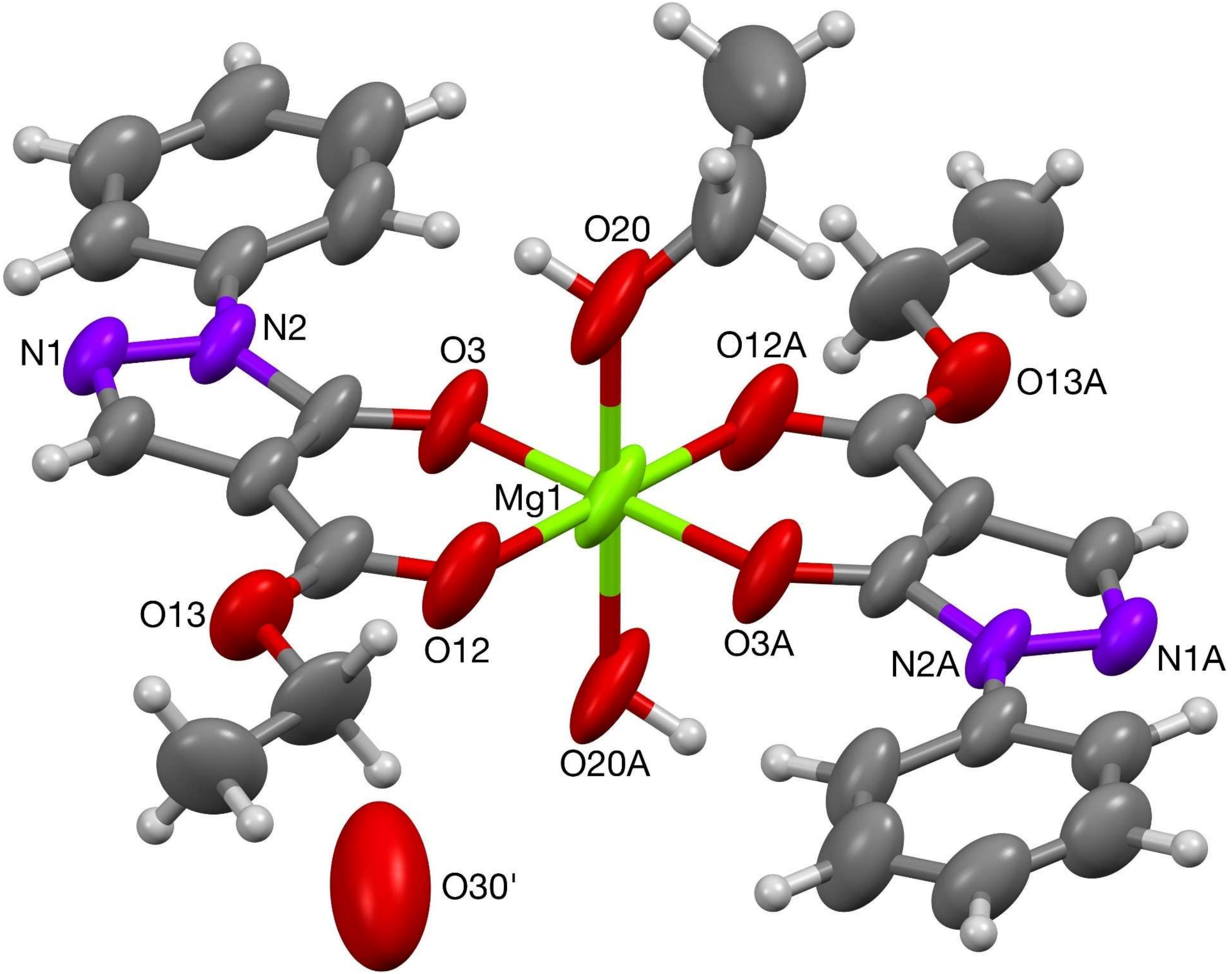

Figure S116: The crystal structure of the C_i-symmetric complex **16** (the ethanol and the aqua/hydrate moieties are disordered about the center of symmetry - 50% probability ellipsoids).

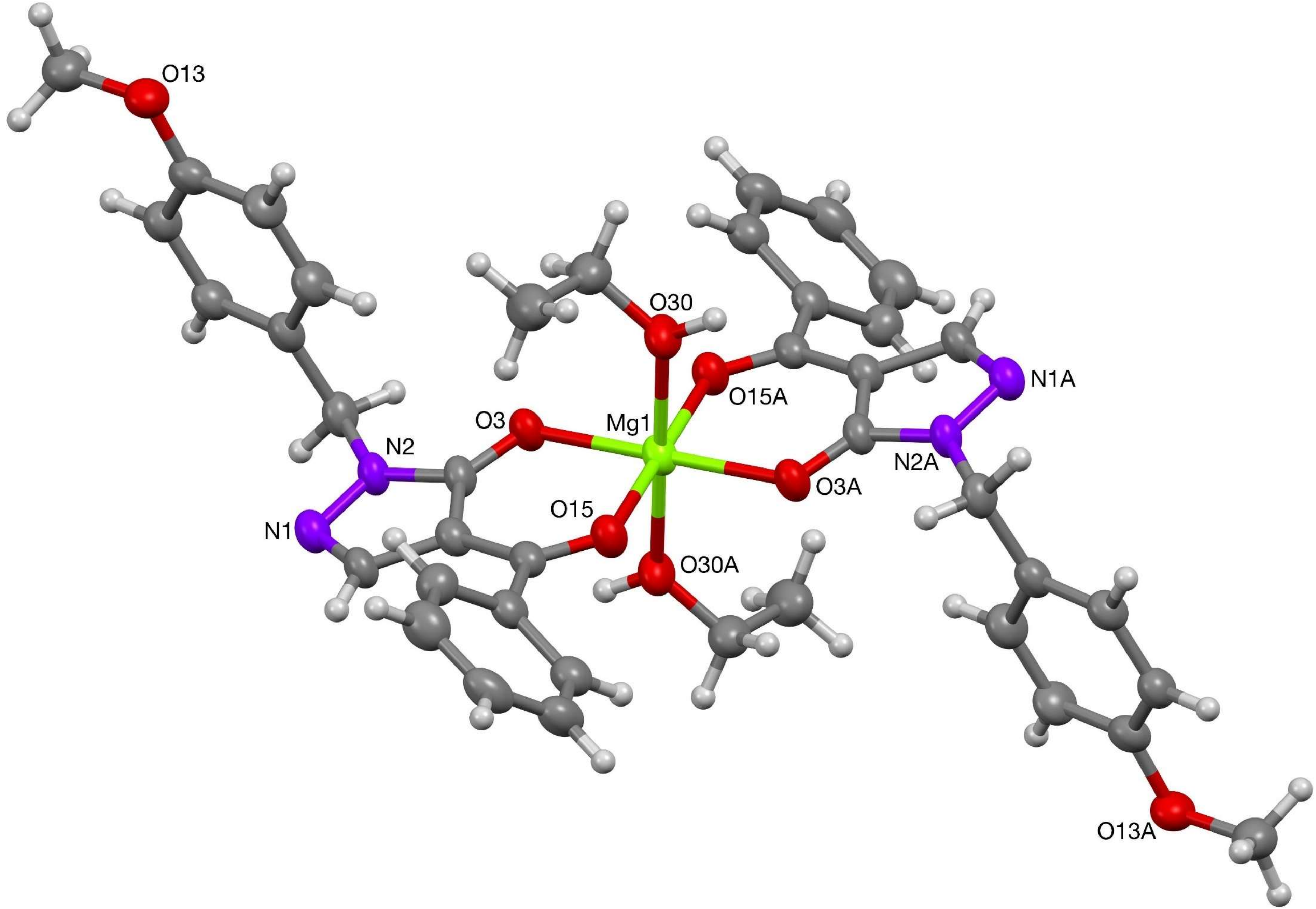

Figure S117: The crystal structure of the C_i-symmetric complex **17** (50% probability ellipsoids).

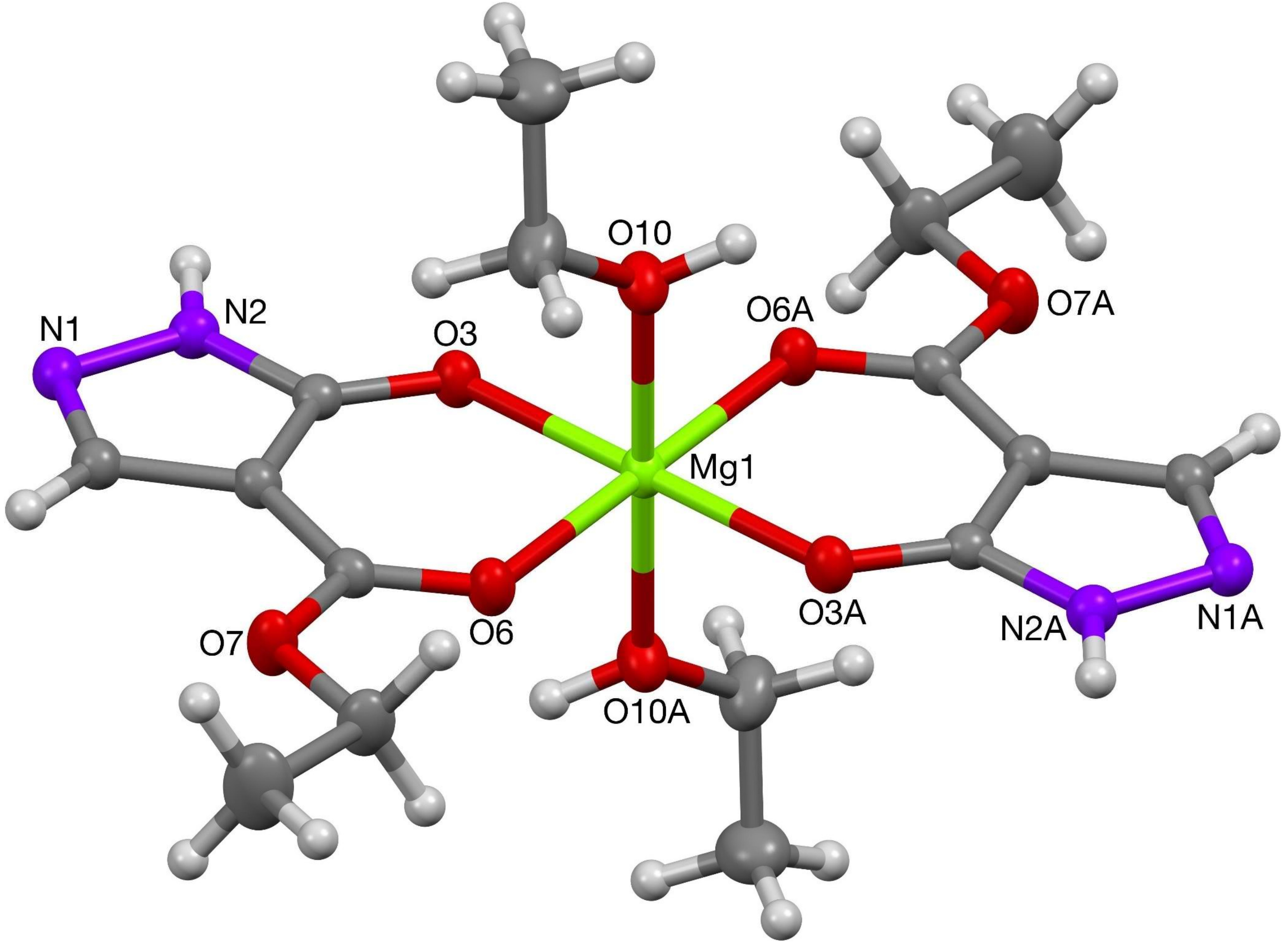

Figure S118: The crystal structure of the C_i-symmetric complex **18** (50% probability ellipsoids).

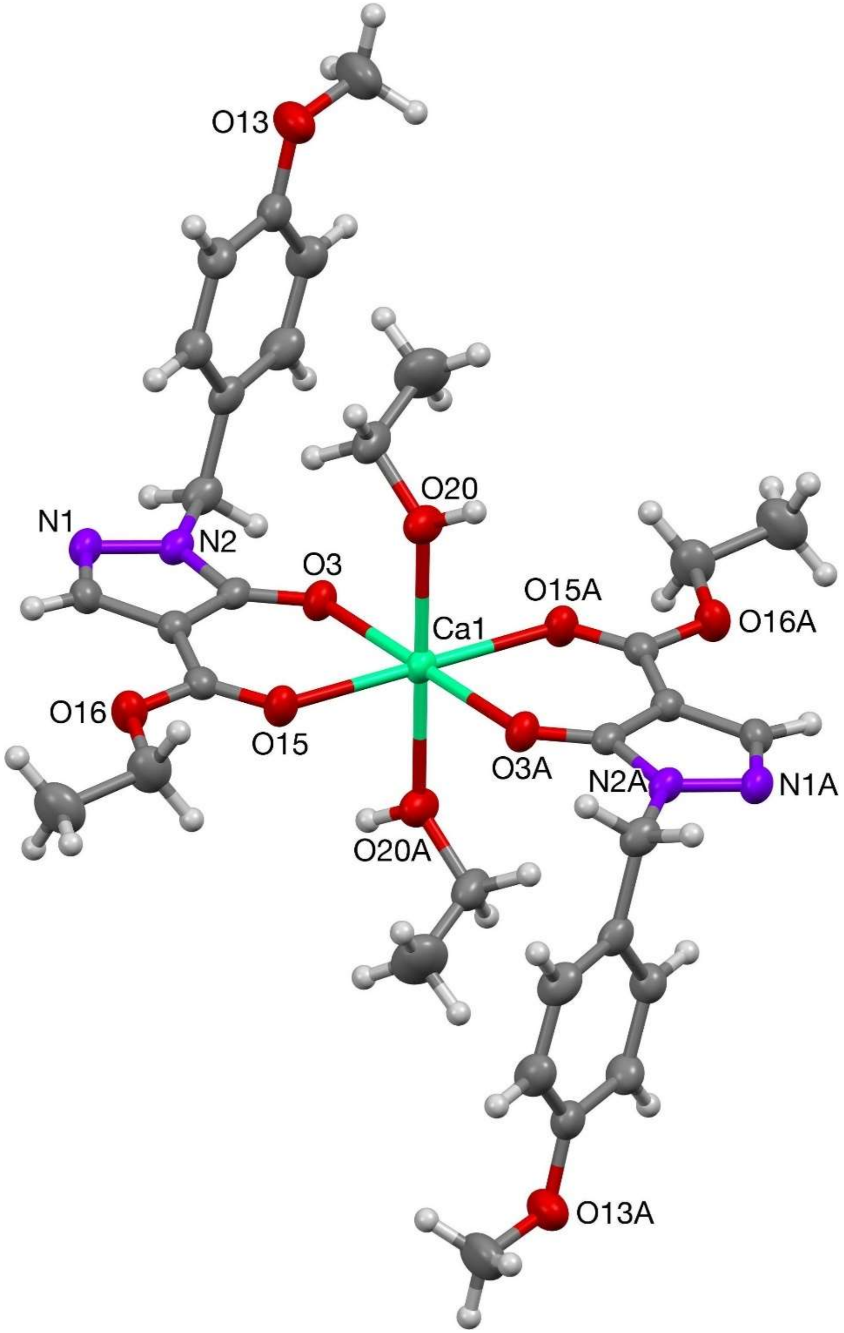

Figure S119: The crystal structure of the C_i-symmetric complex **19** (50% probability ellipsoids).

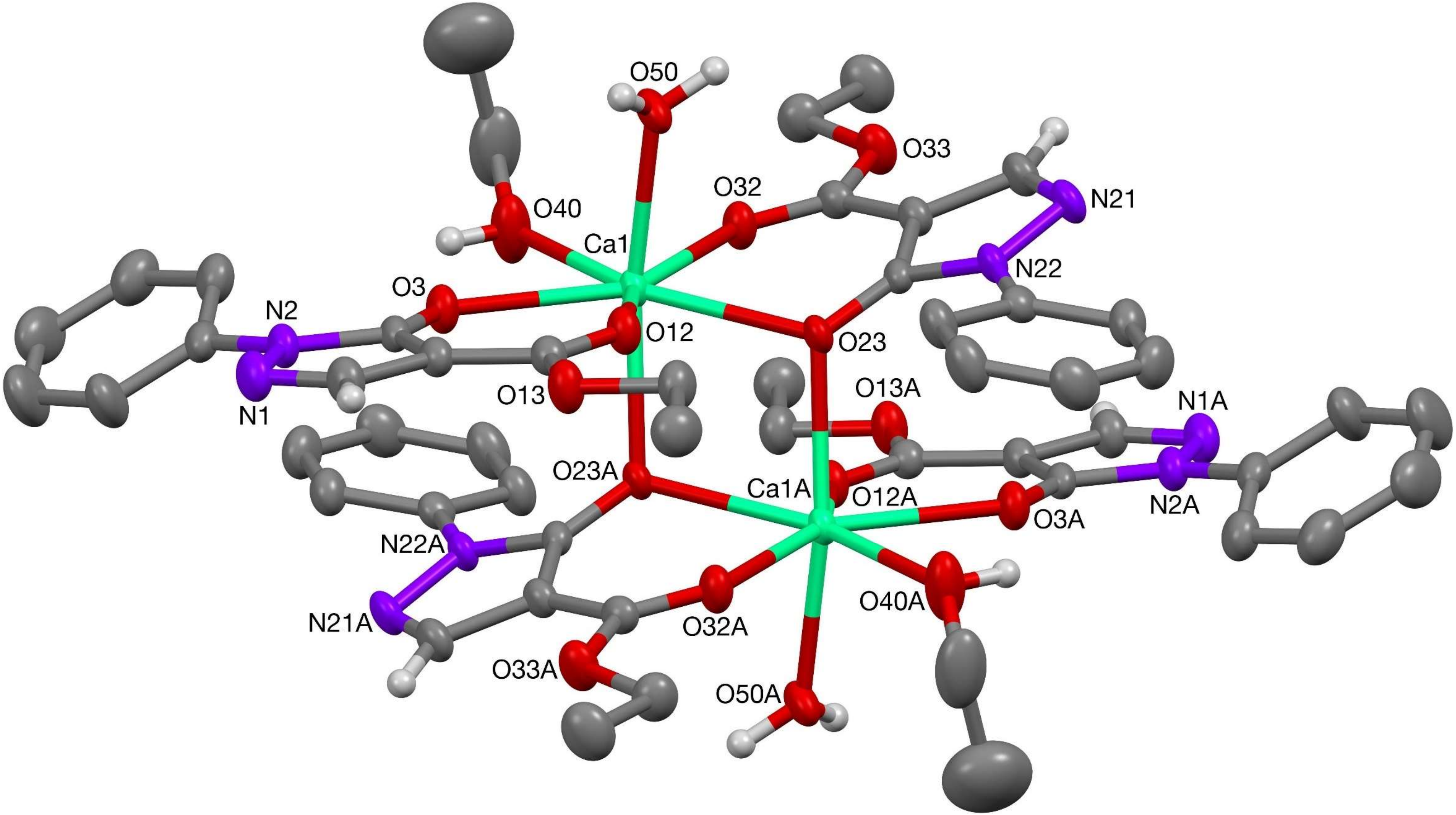

Figure S120: The crystal structure of the C_i-symmetric complex **20** (50% probability ellipsoids).

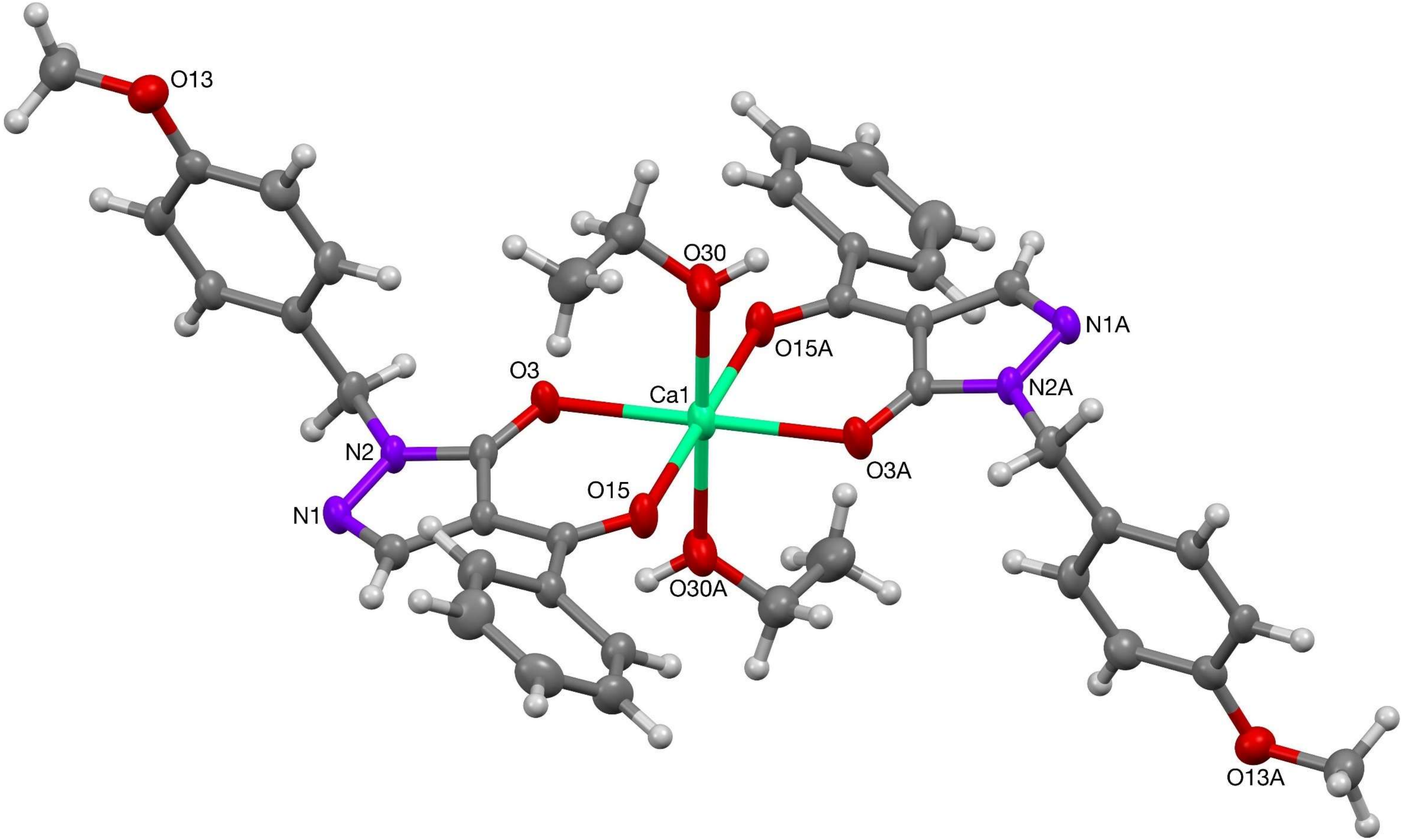

Figure S121: The crystal structure of the C_i-symmetric complex **21** (50% probability ellipsoids).

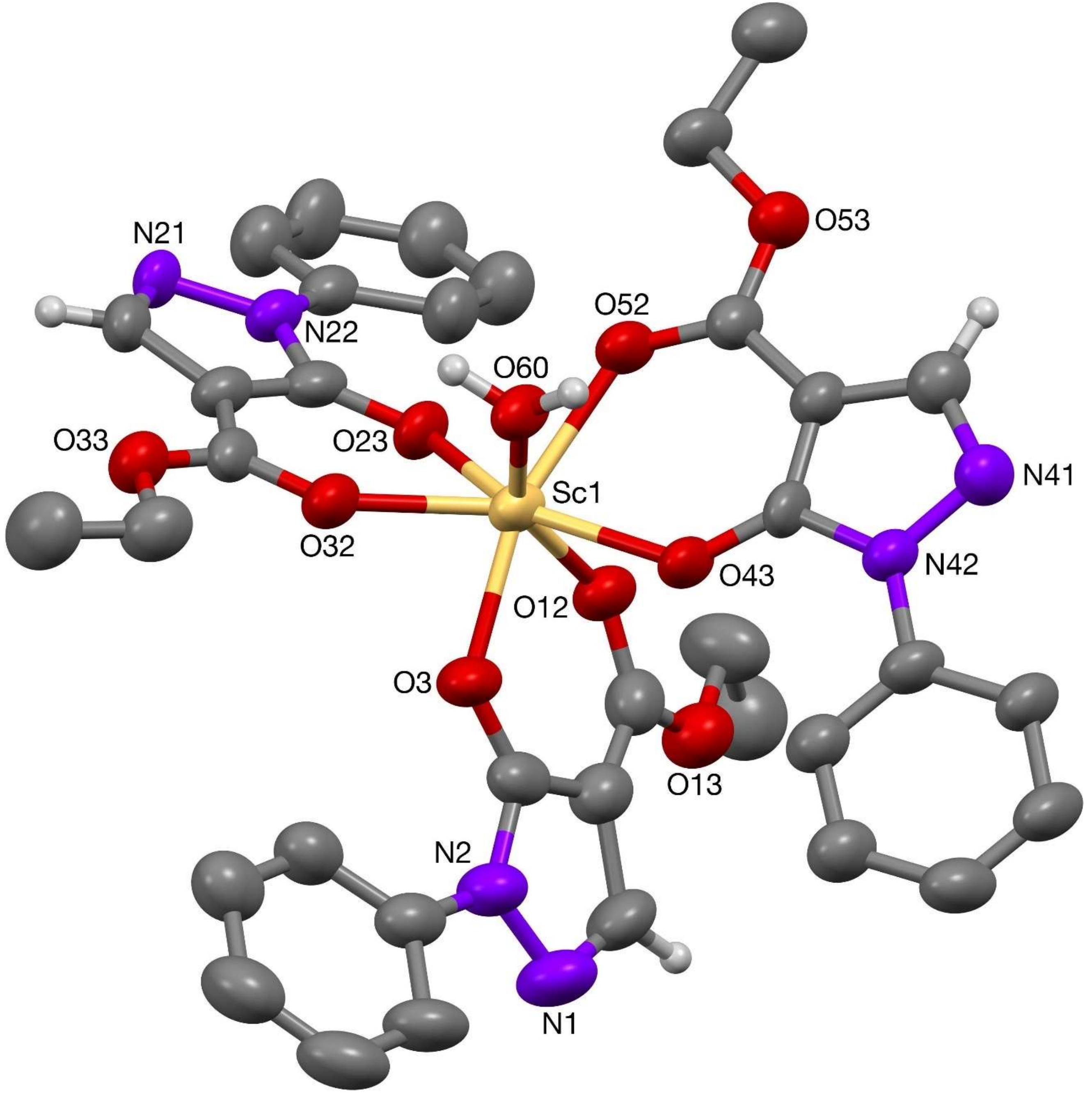

Figure S122: The crystal structure of **24** (50% probability ellipsoids).

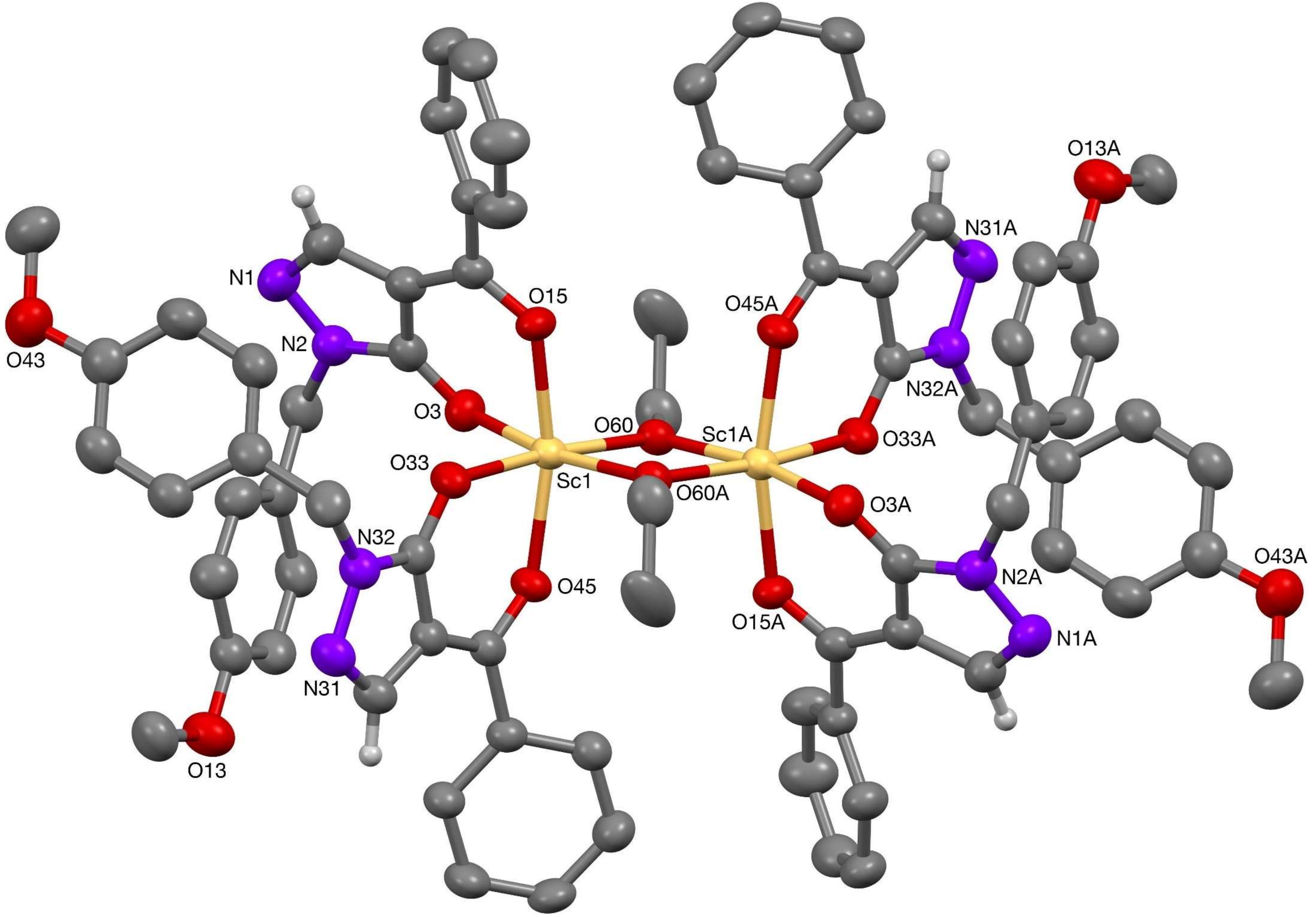

Figure S123: The crystal structure of the C_i-symmetric complex **25** (50% probability ellipsoids).

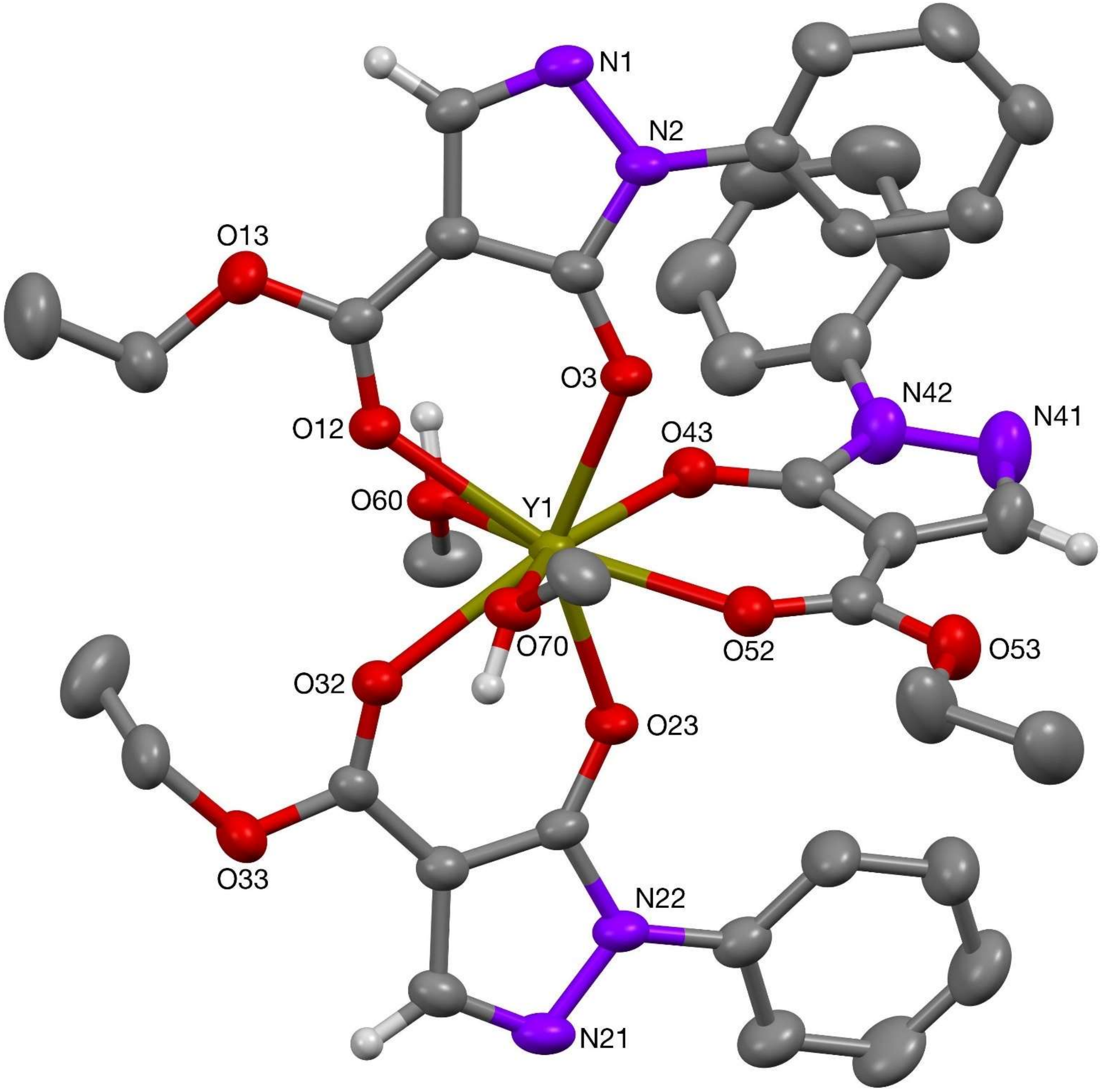

Figure S124: The crystal structure of **28** (50% probability ellipsoids).

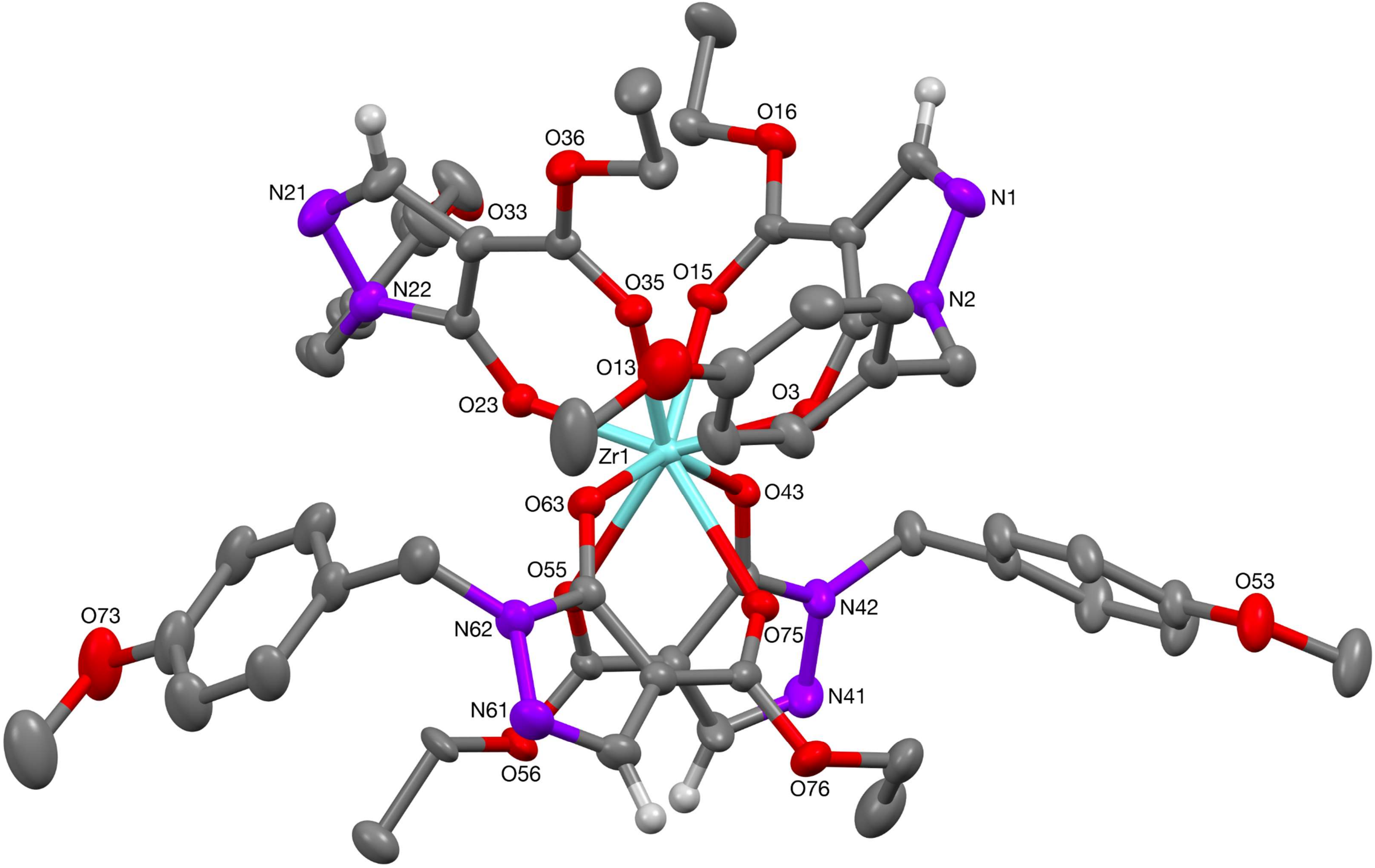

Figure S125: The crystal structure of **33** (50% probability ellipsoids).

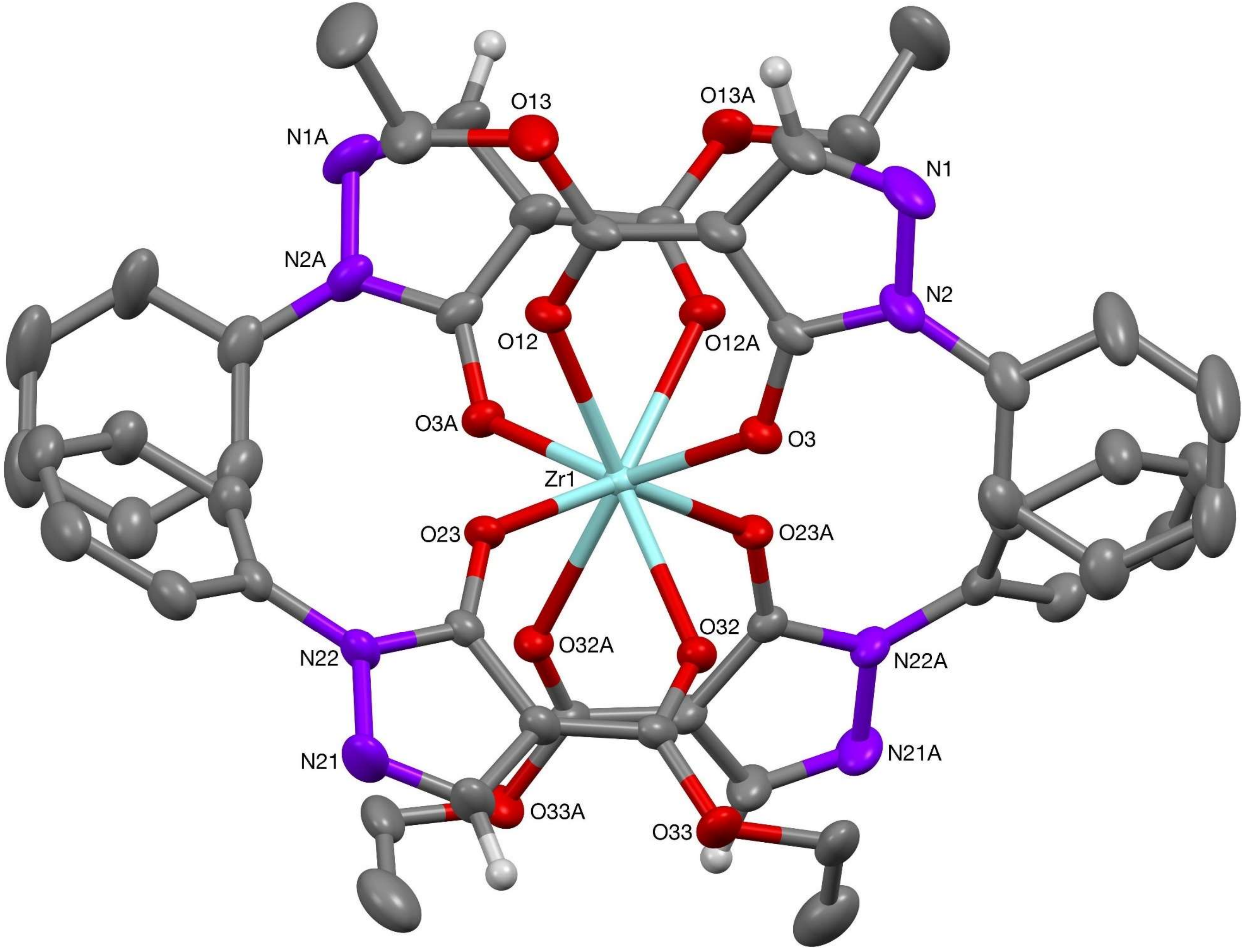

Figure S126: The crystal structure of the C_2-symmetric complex **34** (50% probability ellipsoids). The C2 axis is approximately vertical in the plane of the picture, passing through the metal center and bisecting the O12···O12A and O32···O32A vectors.

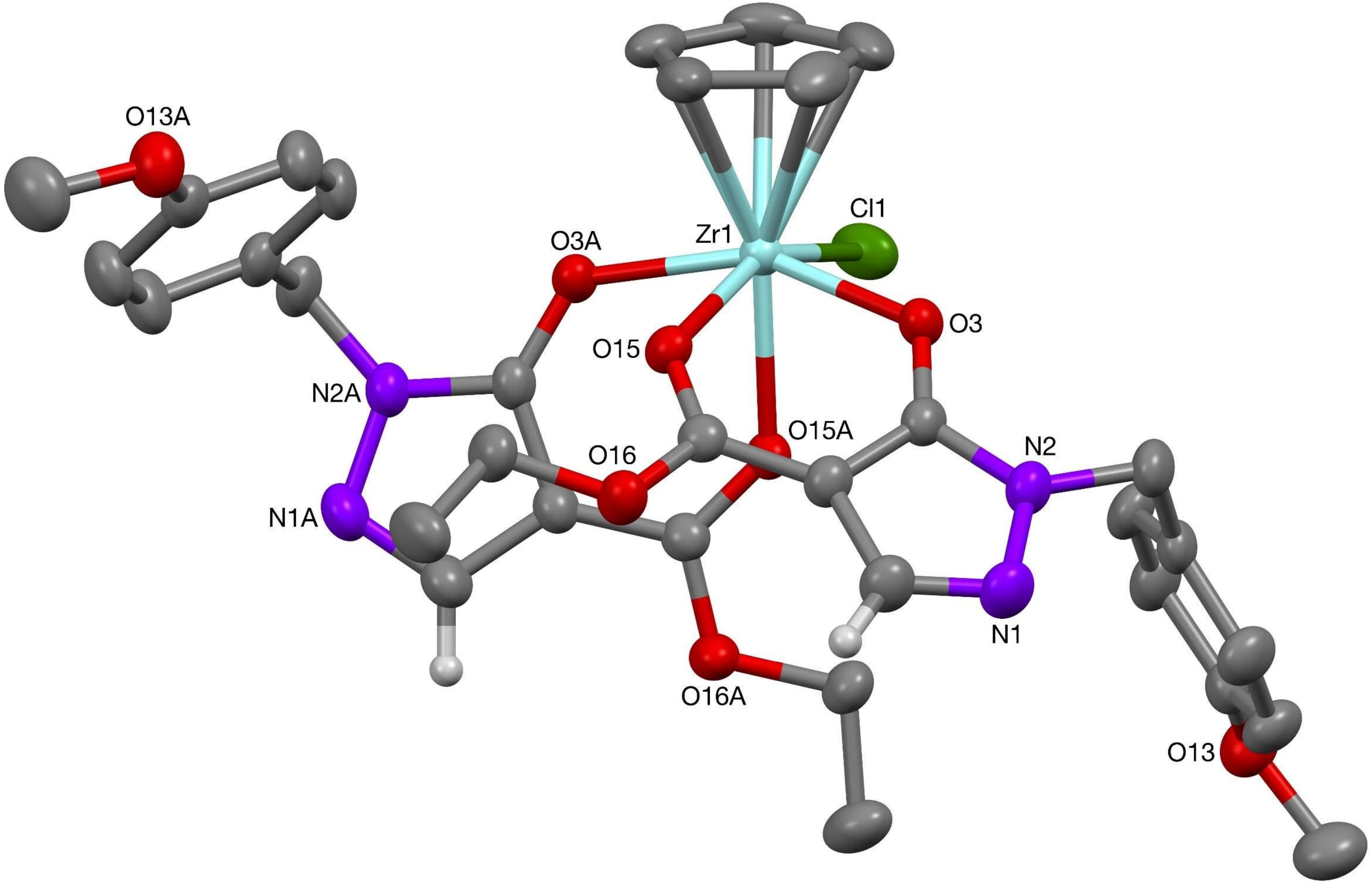

Figure S127: The crystal structure of the C_2-symmetric complex **37** (50% probability ellipsoids). The C2 axis passes through the metal center and bisects the O3···O3A and O15···O15A vectors (the cyclopentadienyl and chloride ligands are disordered about the C2 axis.

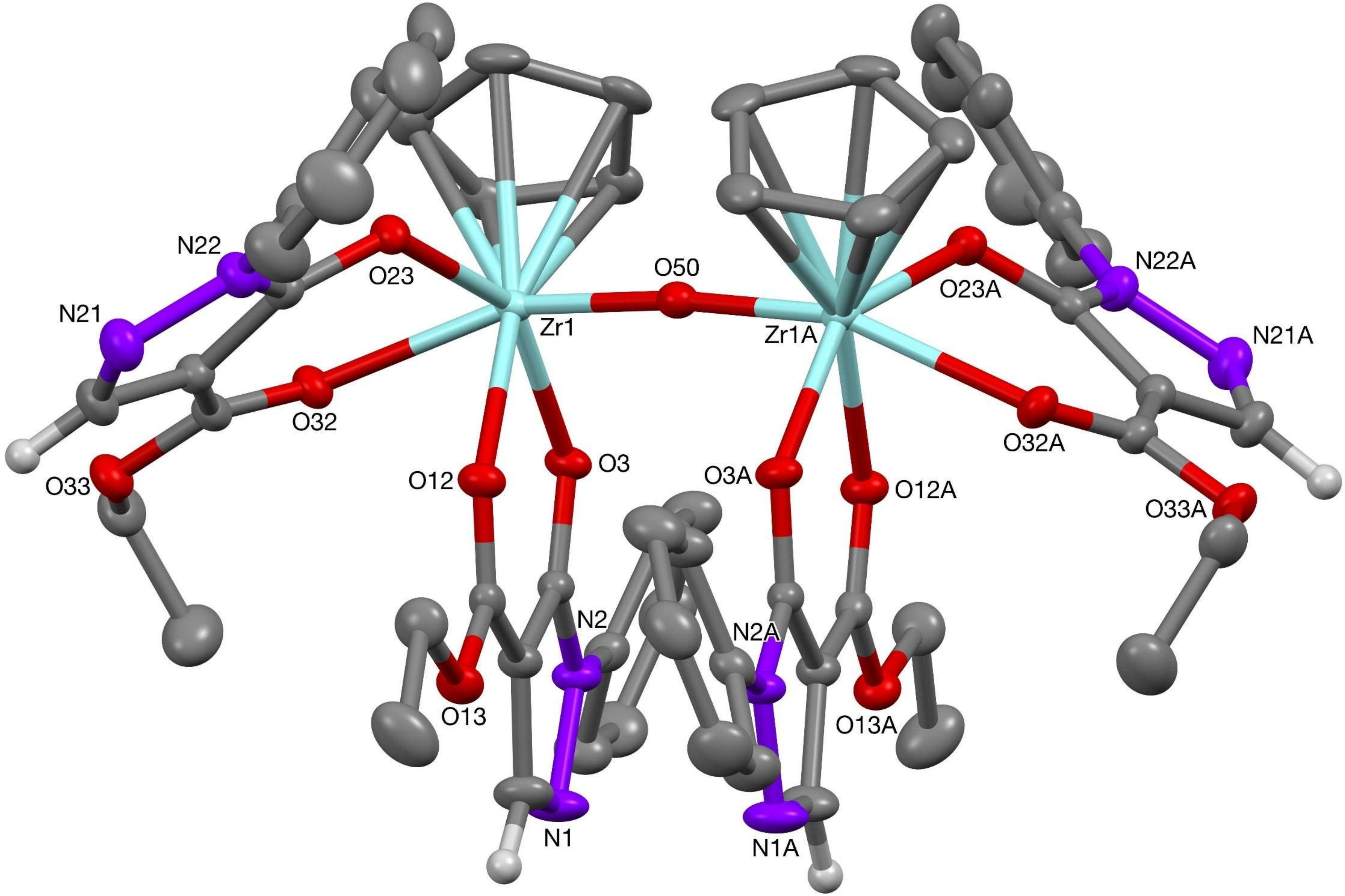

Figure S128: The crystal structure of the C_2-symmetric complex **38** (30% probability ellipsoids). The C2 axis is approximately vertical in the plane of the picture, passing through O50 and bisecting the O23···O23A and O3···O3A vectors.

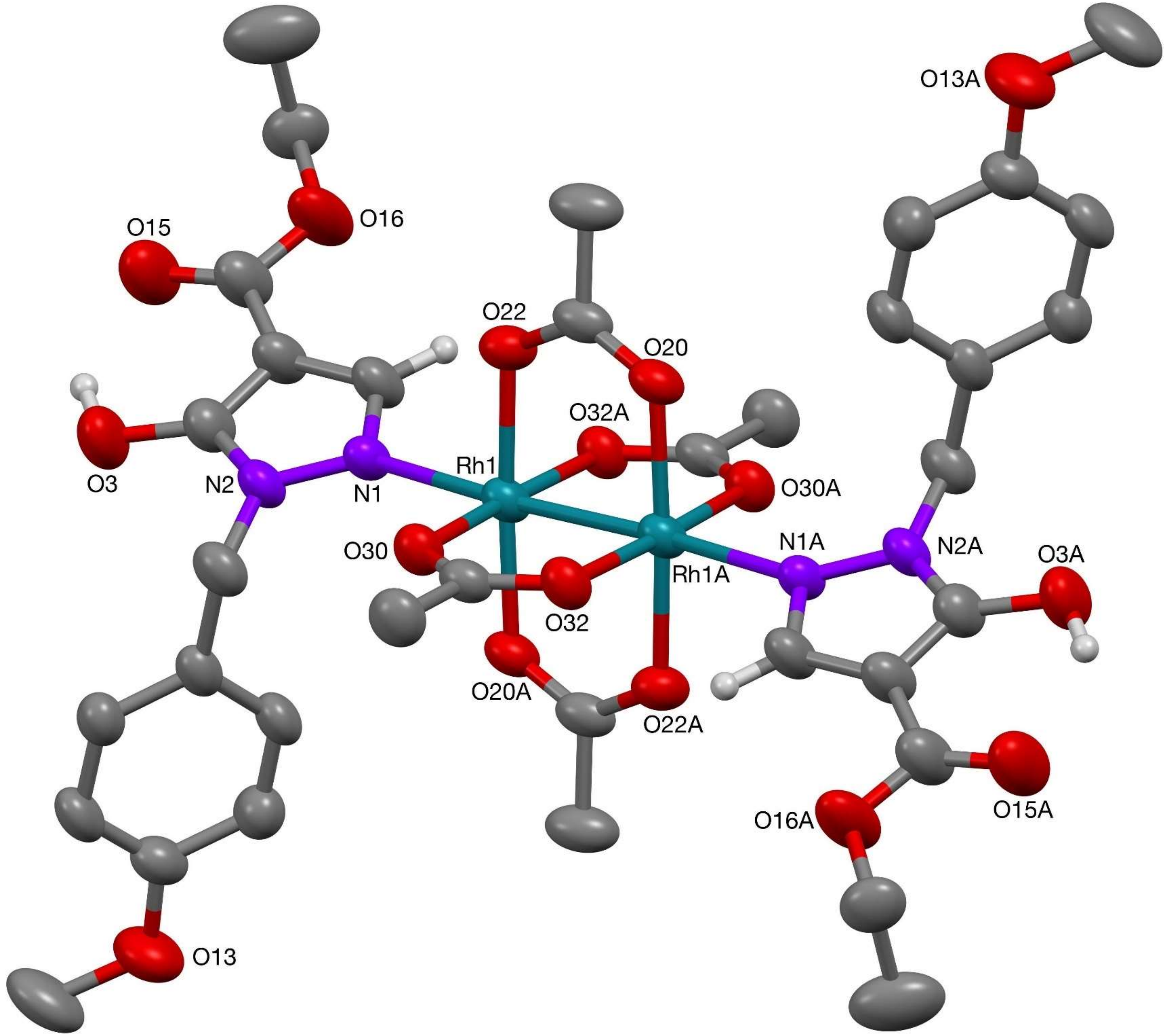

Figure S129:The crystal structure of the C_i-symmetric complex **39** (50% probability ellipsoids).

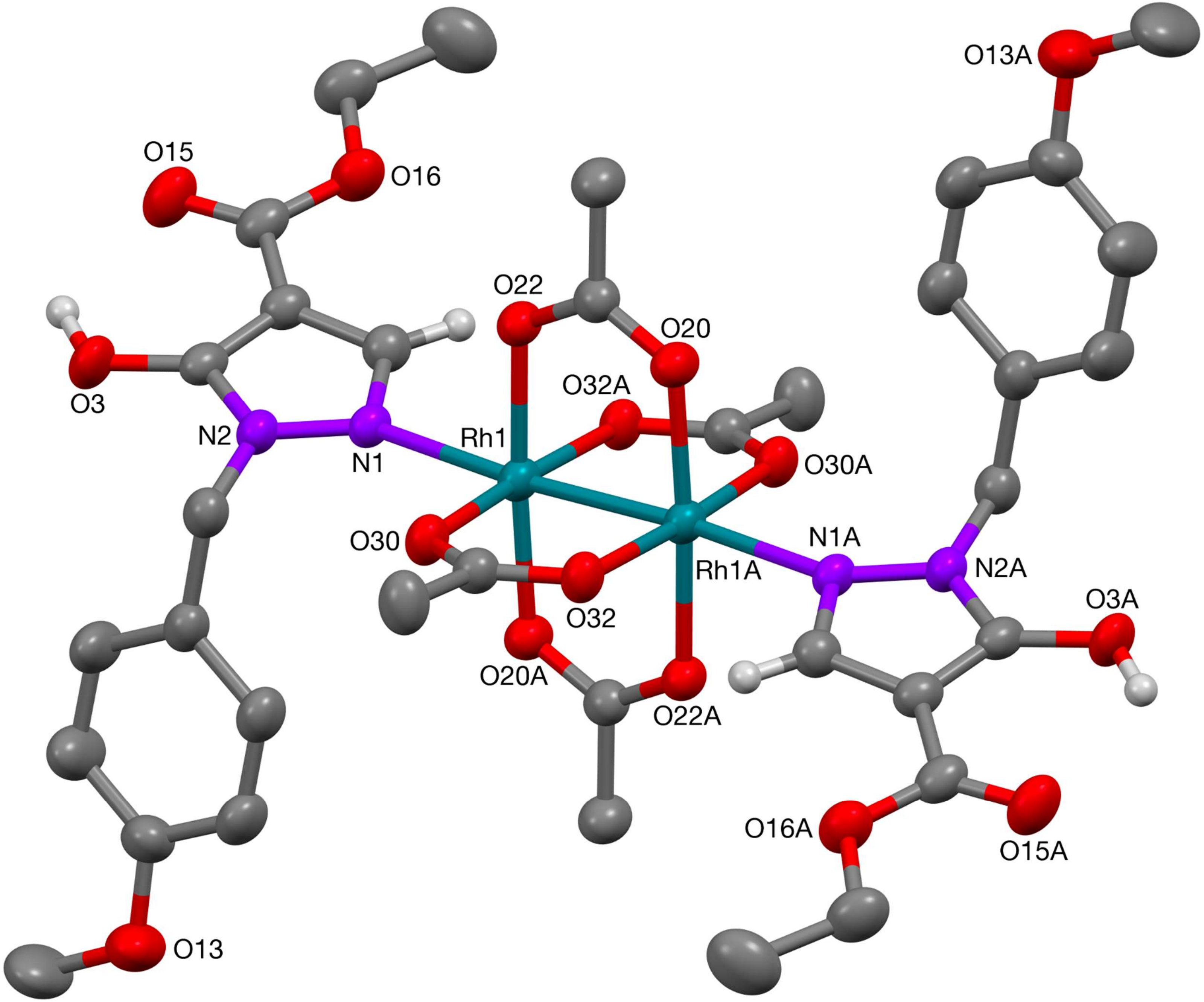

Figure S130: The crystal structure of the C_i-symmetric complex **39** (50% probability ellipsoids).

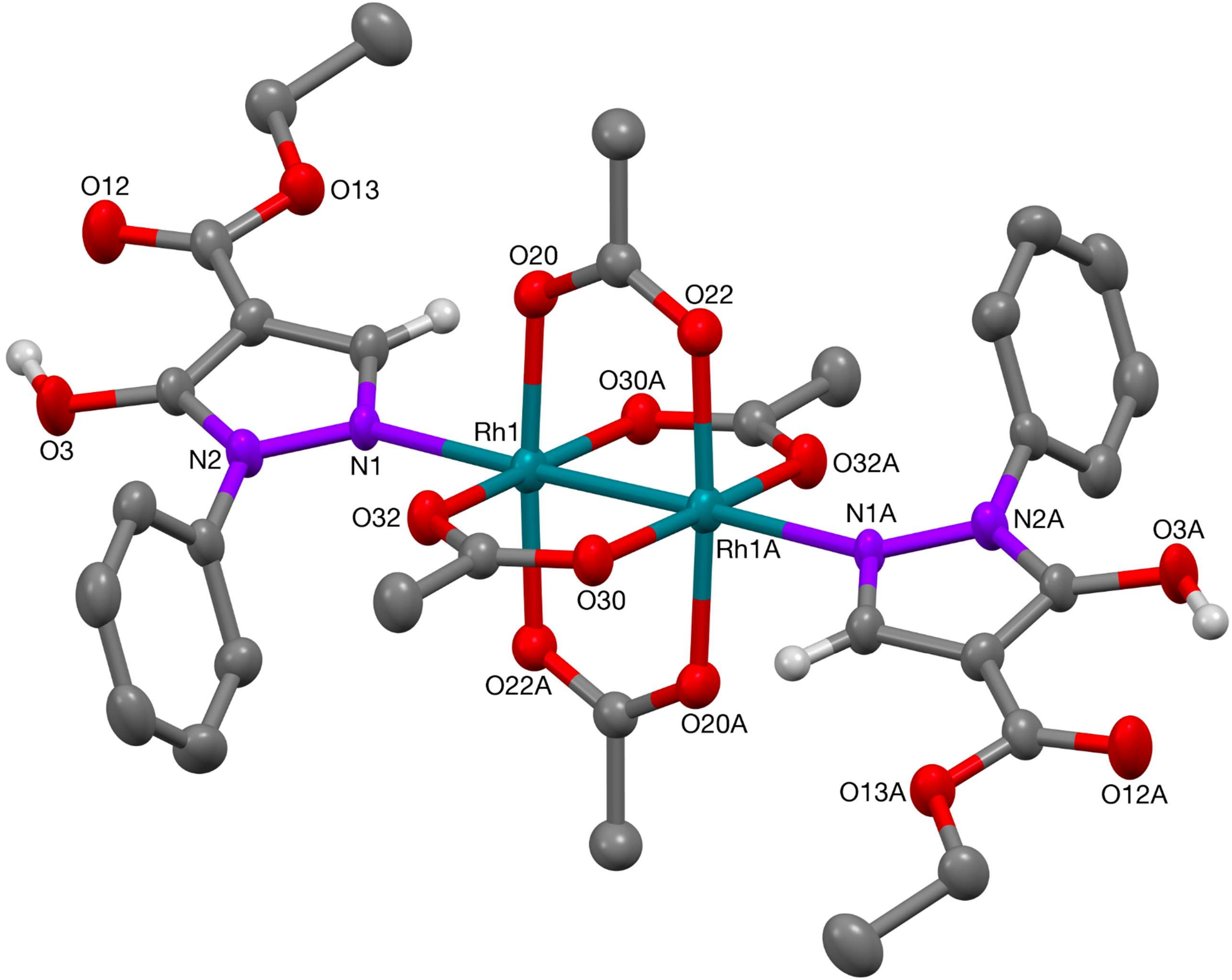

Figure S131: The crystal structure of the C_i-symmetric complex **40** (50% probability ellipsoids).

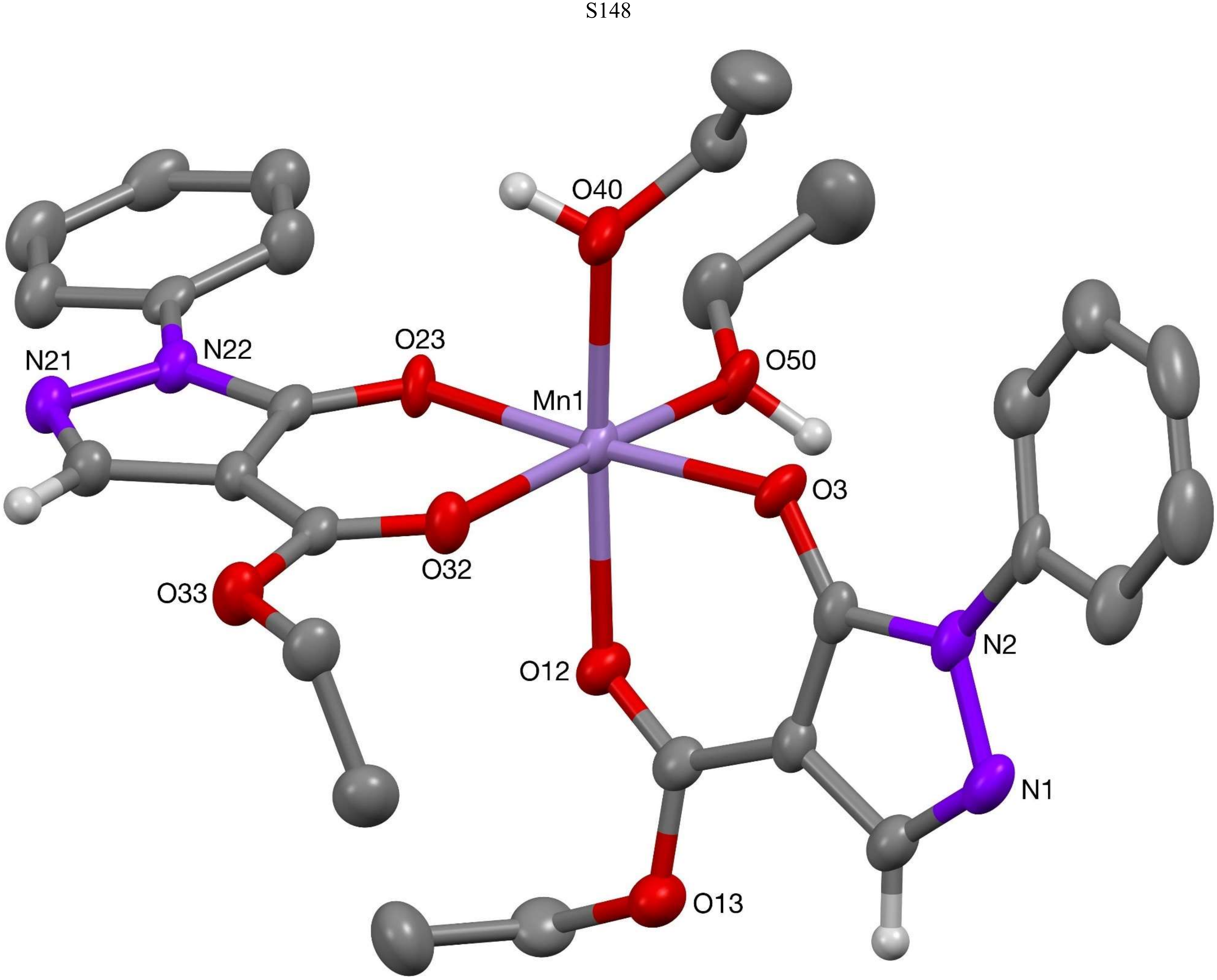

Figure S132: The crystal structure of **42** (50% probability ellipsoids).

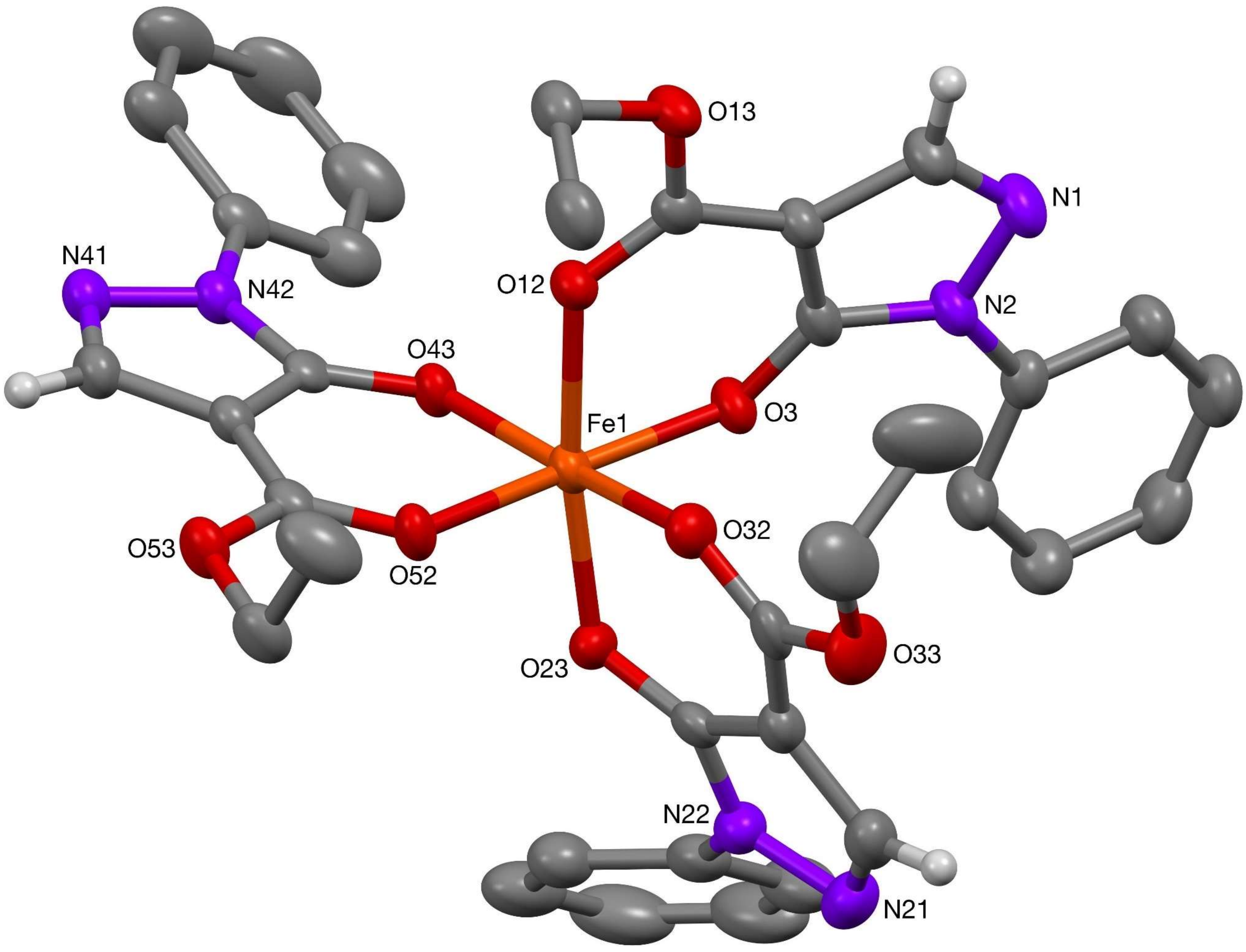

Figure S133: The crystal structure of **46** (50% probability ellipsoids).

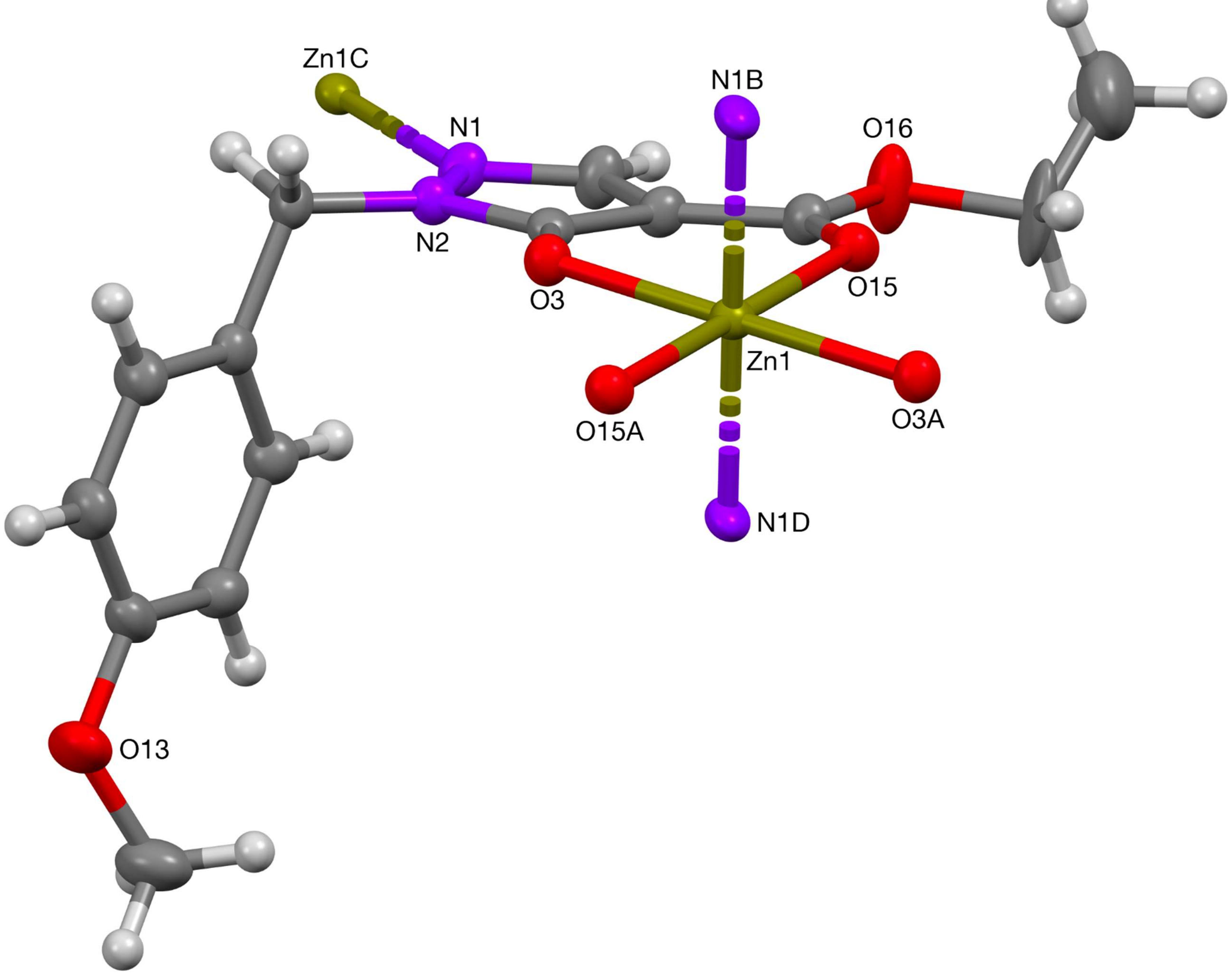

Figure S134: The structure of the asymmetric unit present in the crystal of **49** (50% probability ellipsoids).

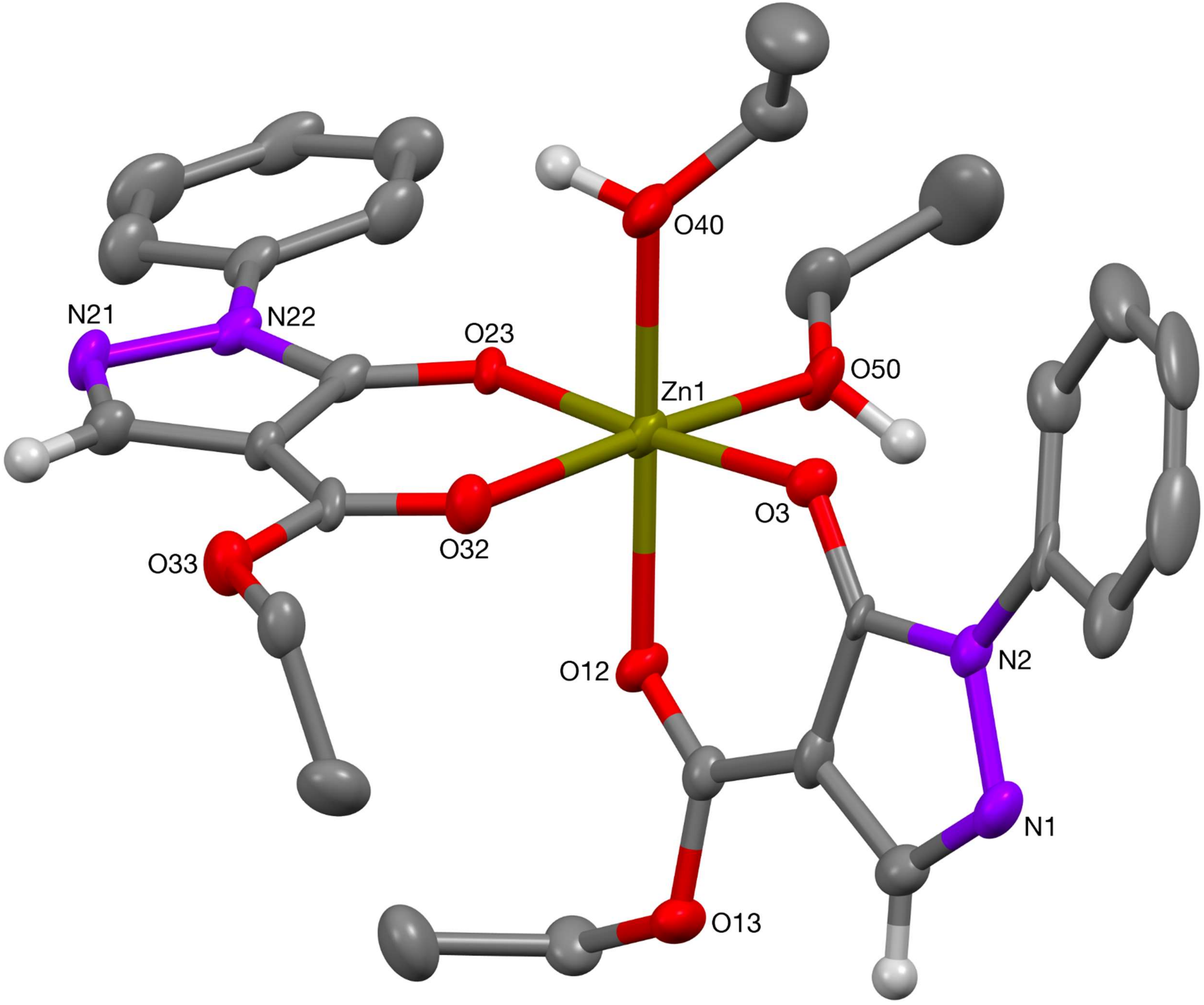

Figure S135: The crystal structure of **50** (50% probability ellipsoids).

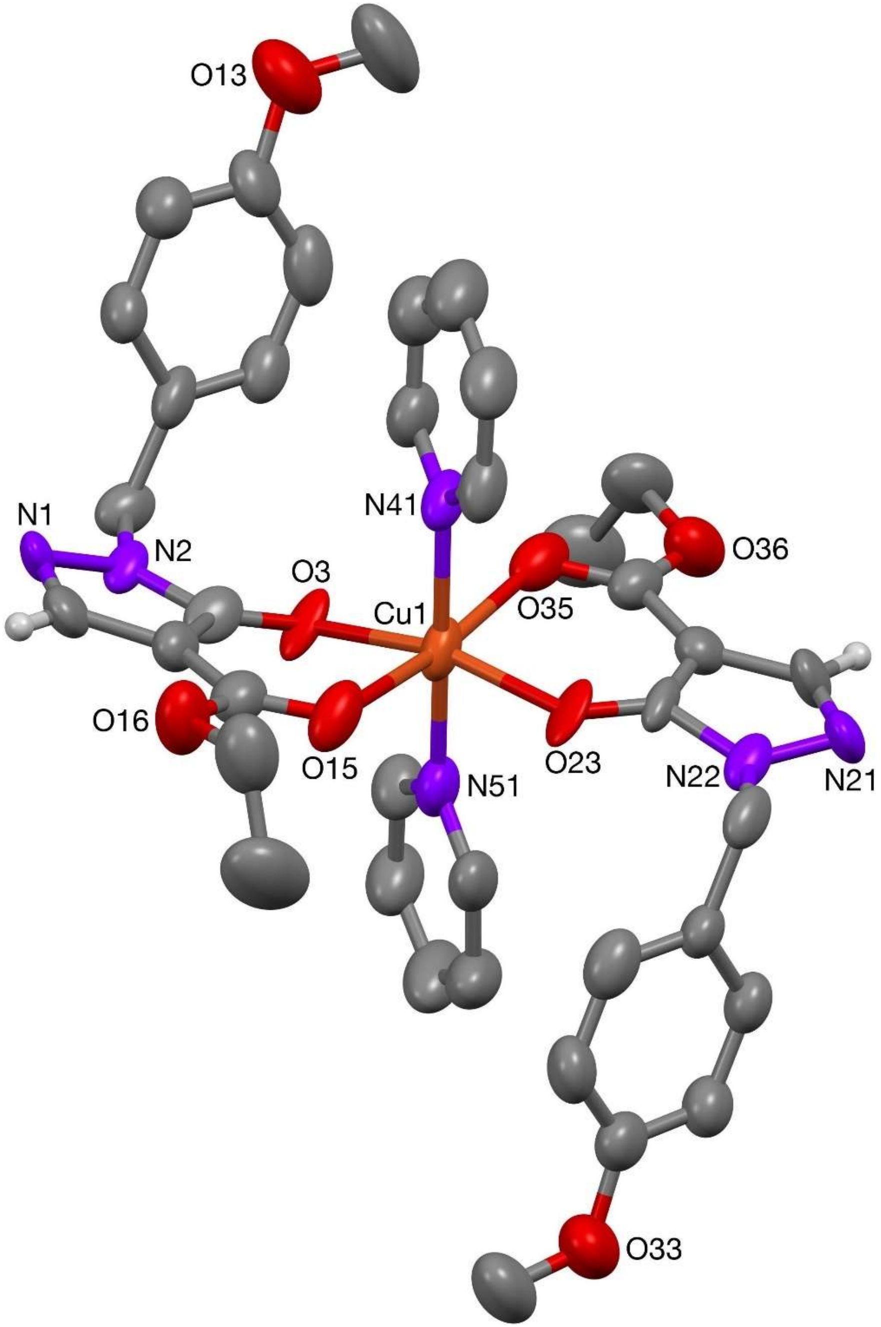

Figure S136: The crystal structure of the C_i-symmetric complex **53**. (The whole of the complex is disordered across the center of symmetry - 50% probability ellipsoids).

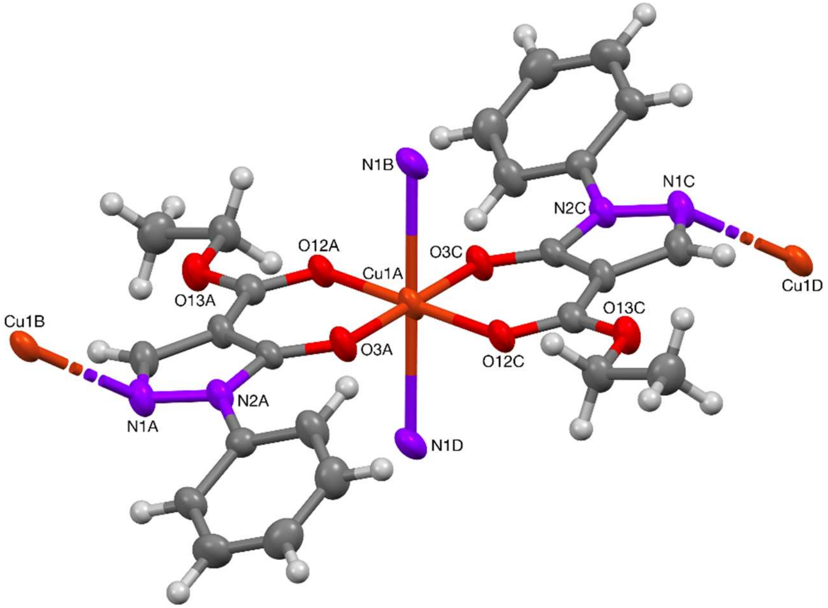

Figure S137: The coordination sphere around **Cu1A**, one of the two independent copper centers present in the crystal of **54** (50% probability ellipsoids). The copper atom sits on a center of symmetry.

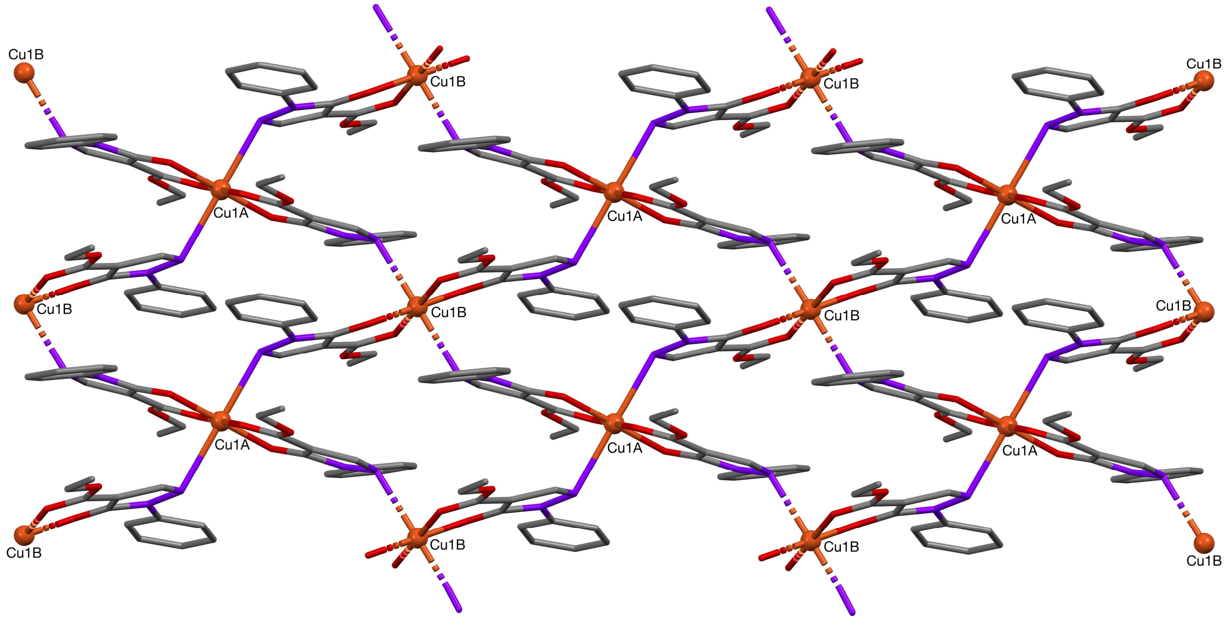

Figure S138: Part of one of the 2D sheets present in the crystal structure of **54**.

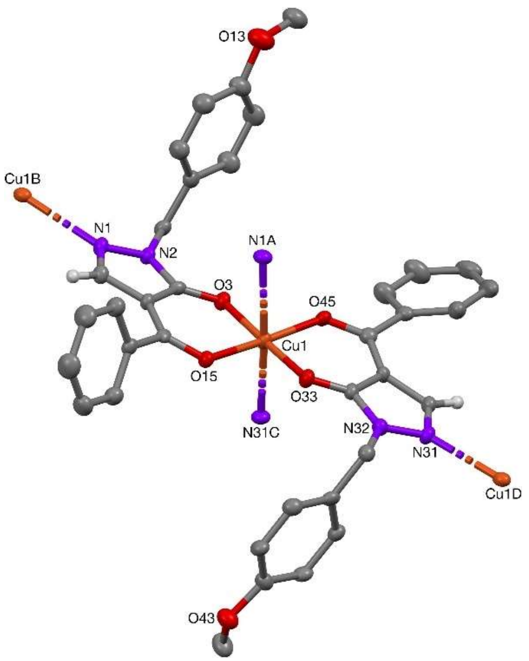

Figure S139: The structure of the asymmetric unit present in the crystal of **55** (50% probability ellipsoids).

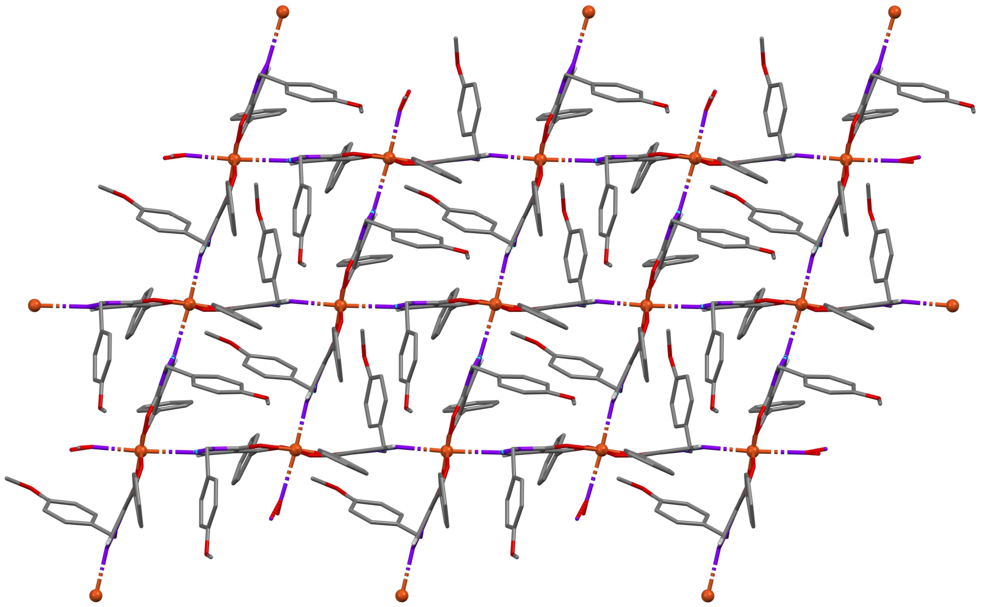

Figure S140: Part of one of the 2D sheets present in the crystal structure of **55**.

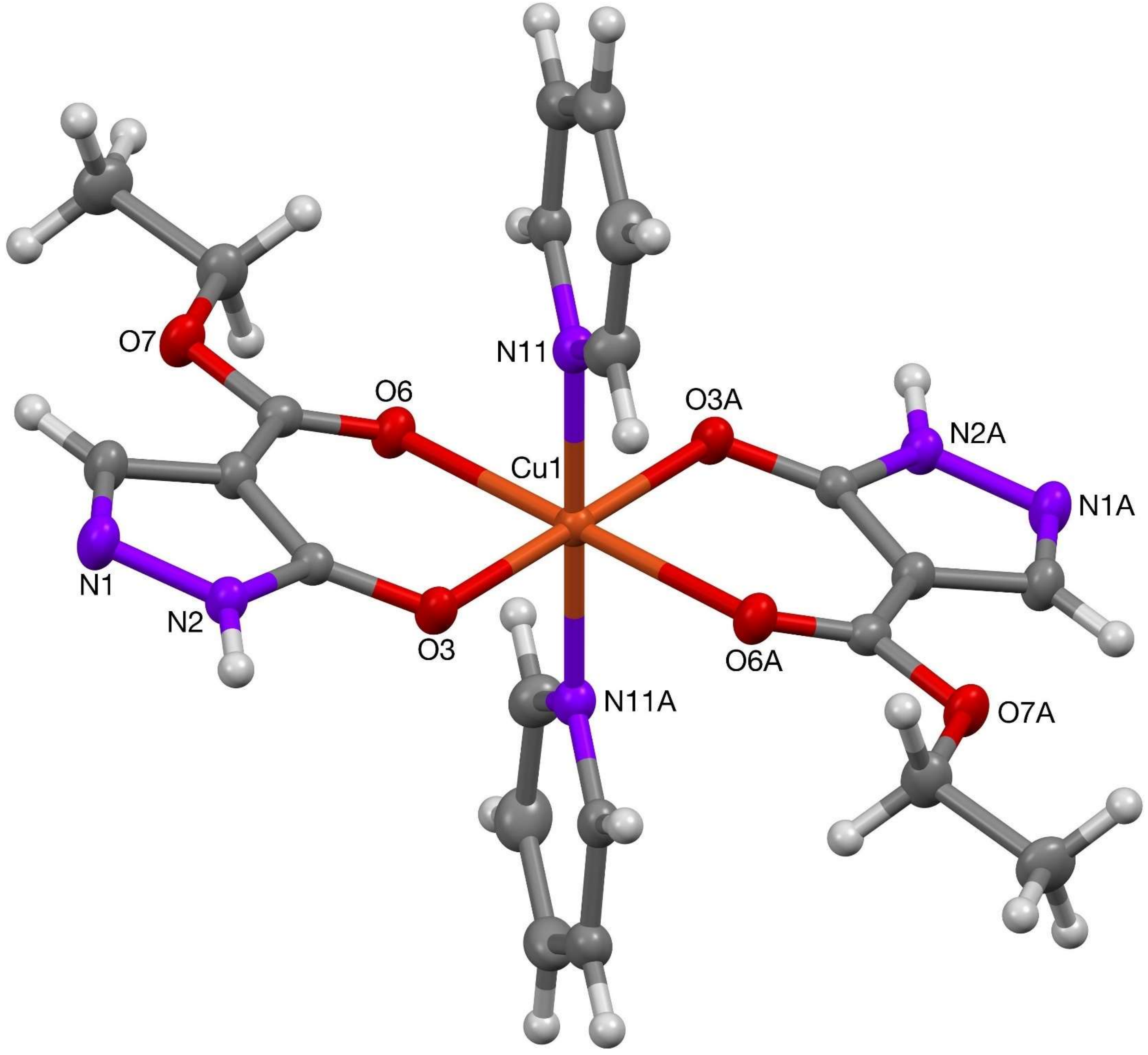

Figure S141: The crystal structure of the C_i-symmetric complex **56** (50% probability ellipsoids).

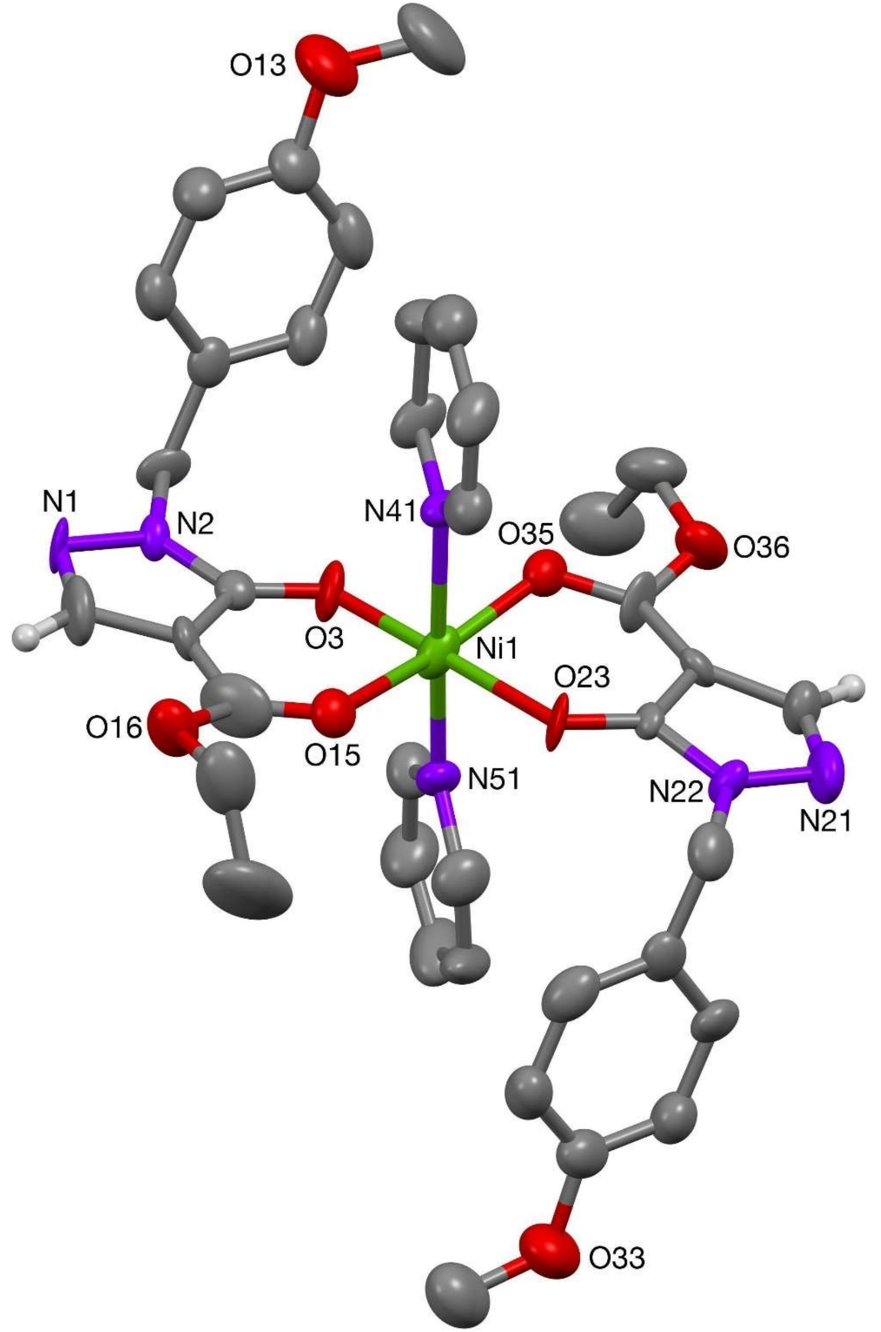

Figure S142: The crystal structure of the C_i-symmetric complex **57**. (The whole of the complex is disordered across the center of symmetry - 50% probability ellipsoids).

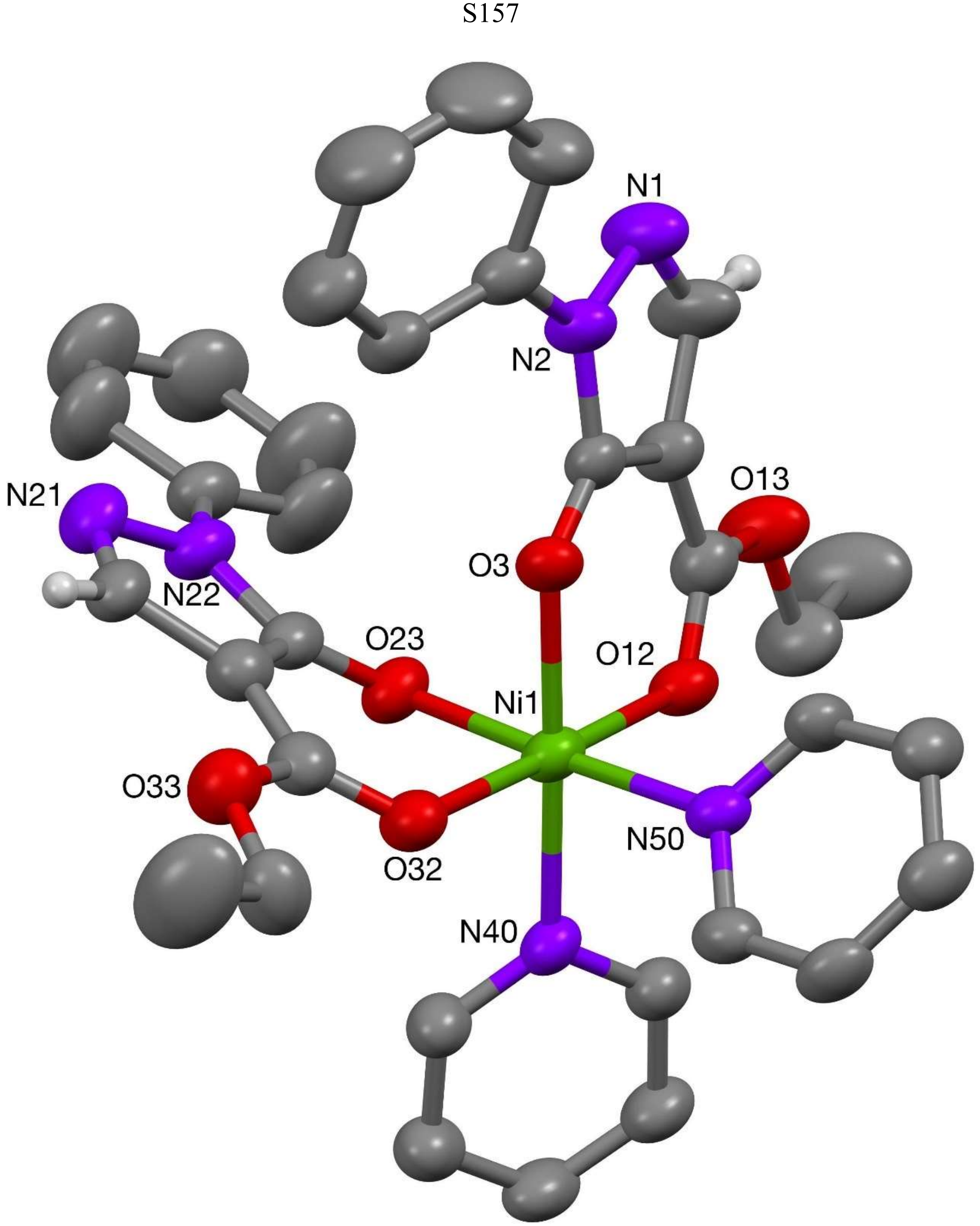

Figure S143: The crystal structure of **58** (50% probability ellipsoids).

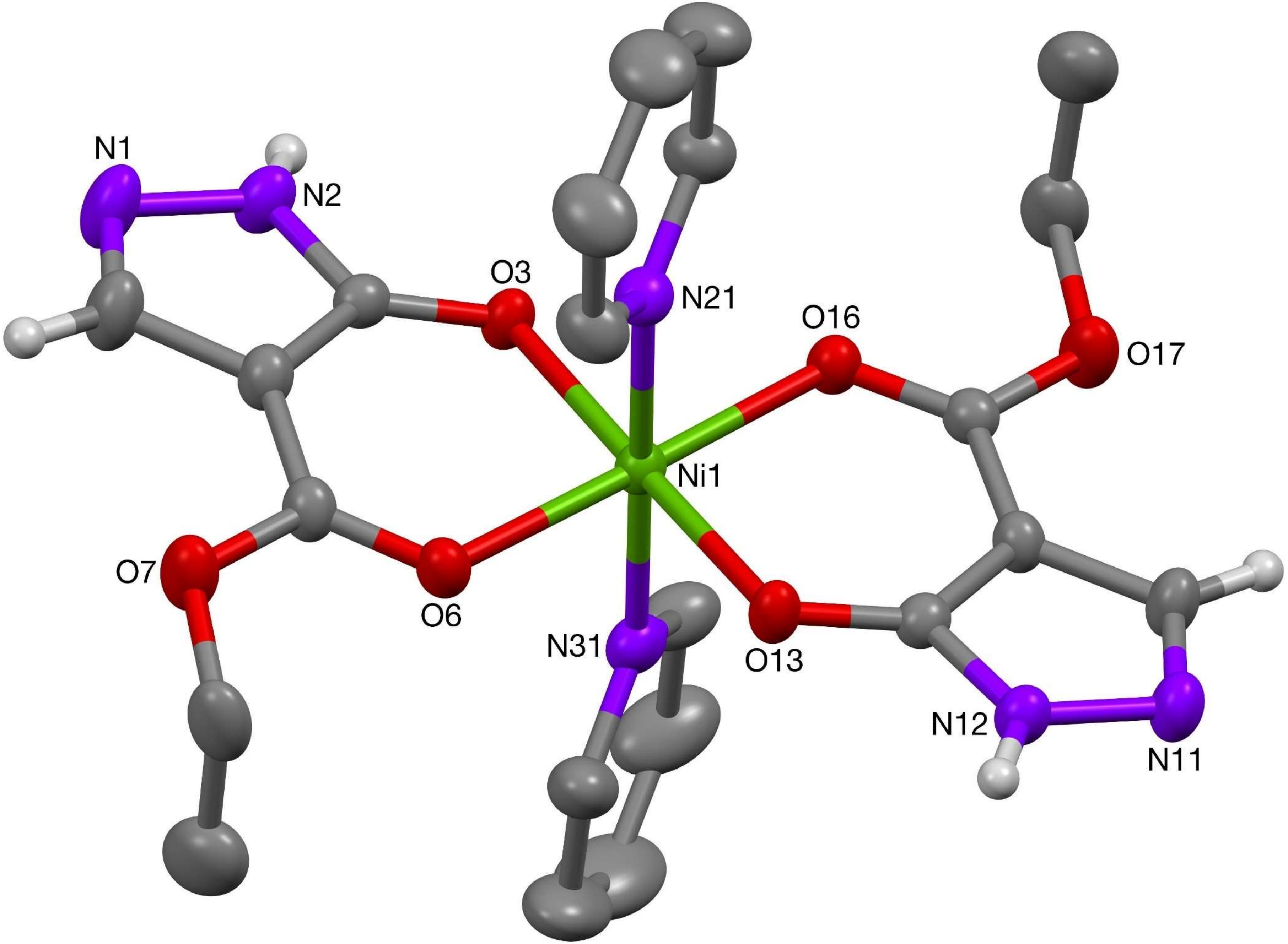

Figure S144: The crystal structure of **60** (50% probability ellipsoids).

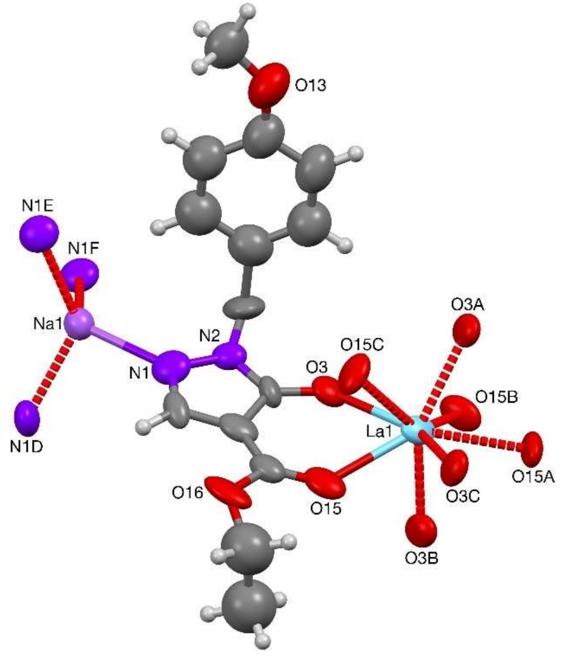

Figure S145: The structure of the asymmetric unit present in the crystal of **61** (30% probability ellipsoids).

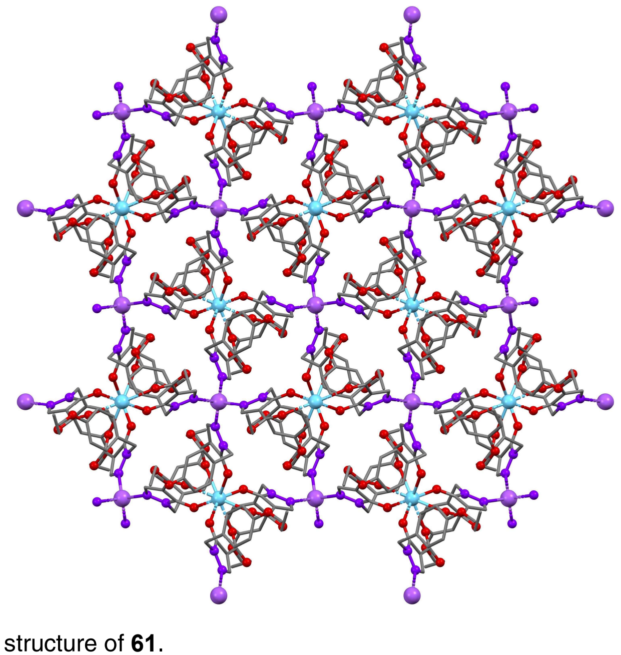

Figure S146: Part of the 3D polymer network present in the crystal structure of **61**.

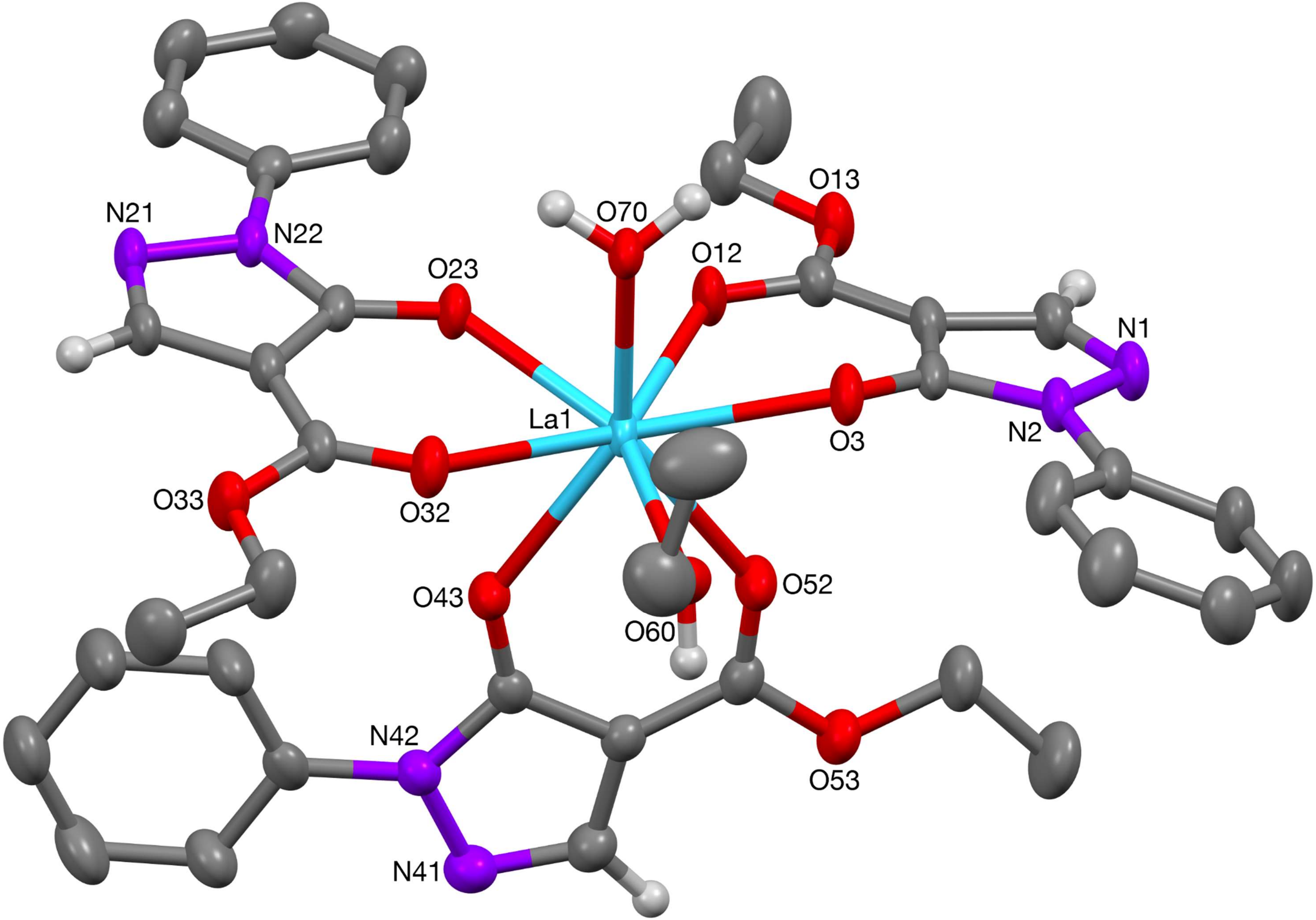

Figure S147: The crystal structure of **62** (50% probability ellipsoids).

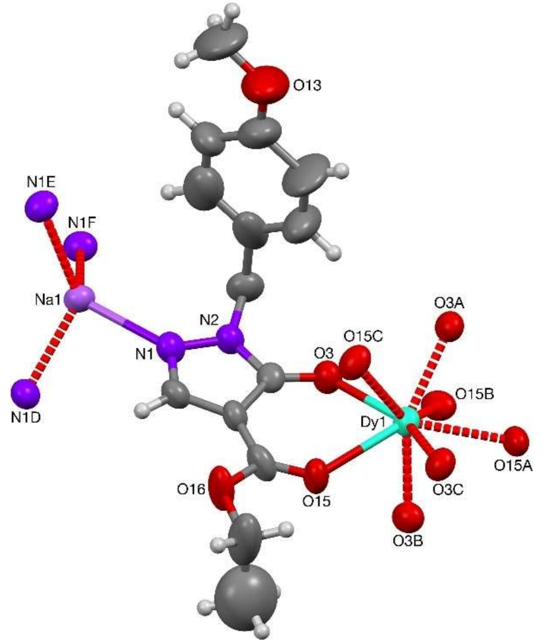

Figure S148: The structure of the asymmetric unit present in the crystal of **65** (50% probability ellipsoids).

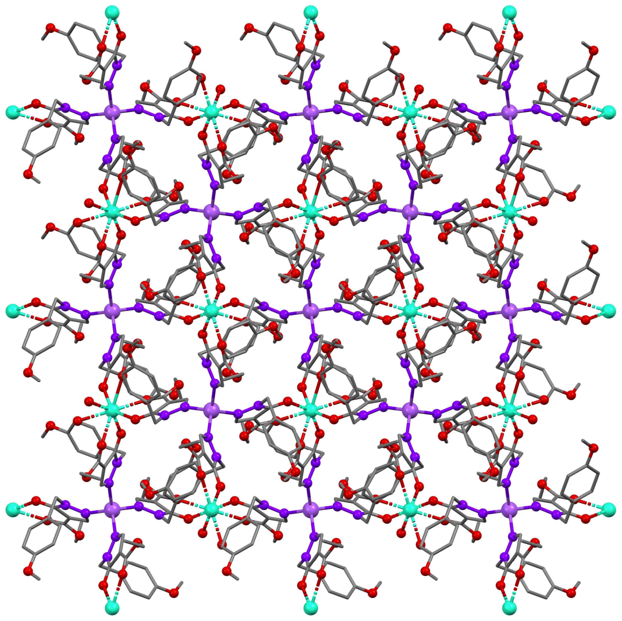

Figure S149: Part of the 3D polymer network present in the crystal structure of **65**.

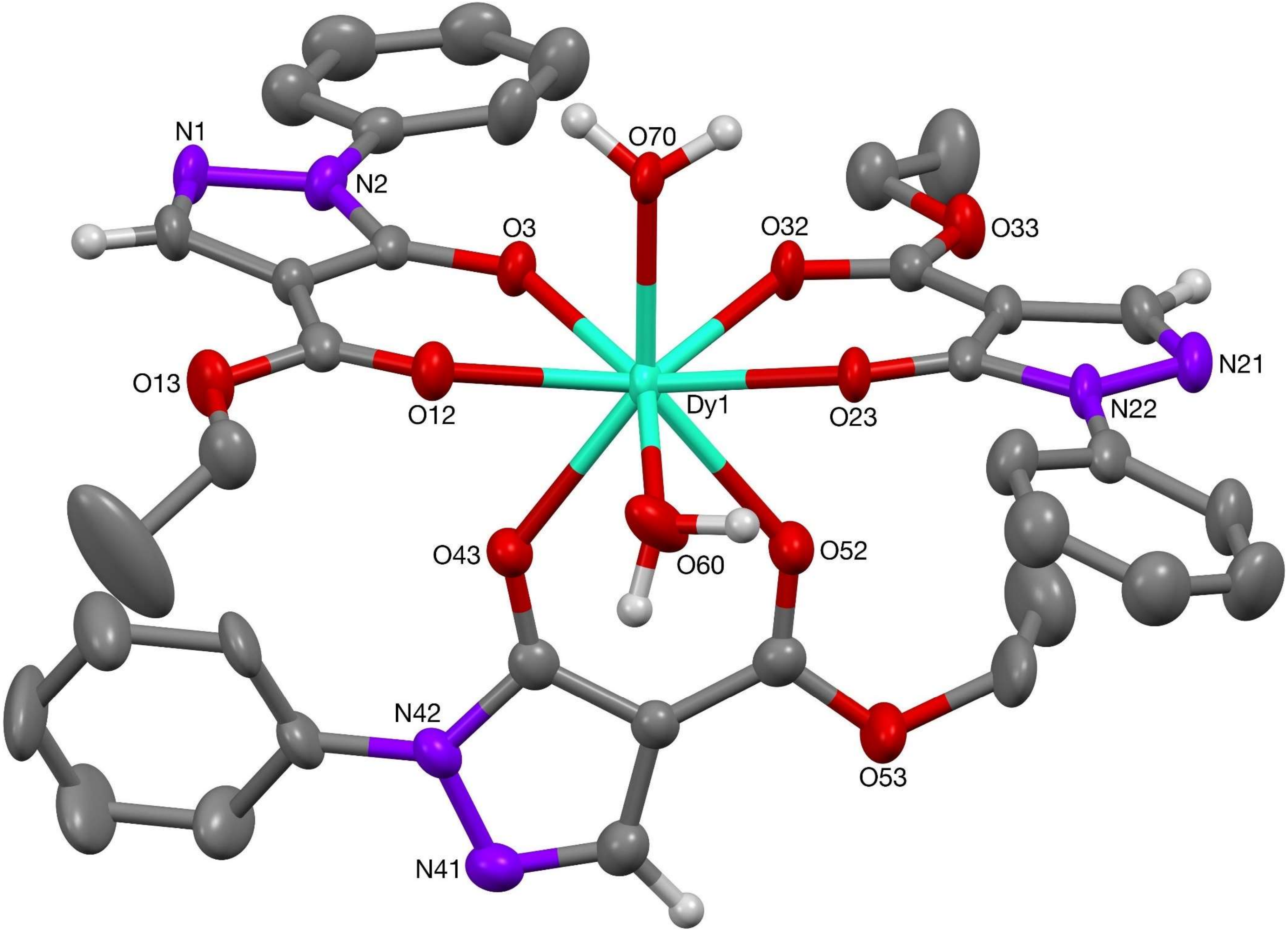

Figure S150: The crystal structure of **66** (50% probability ellipsoids).

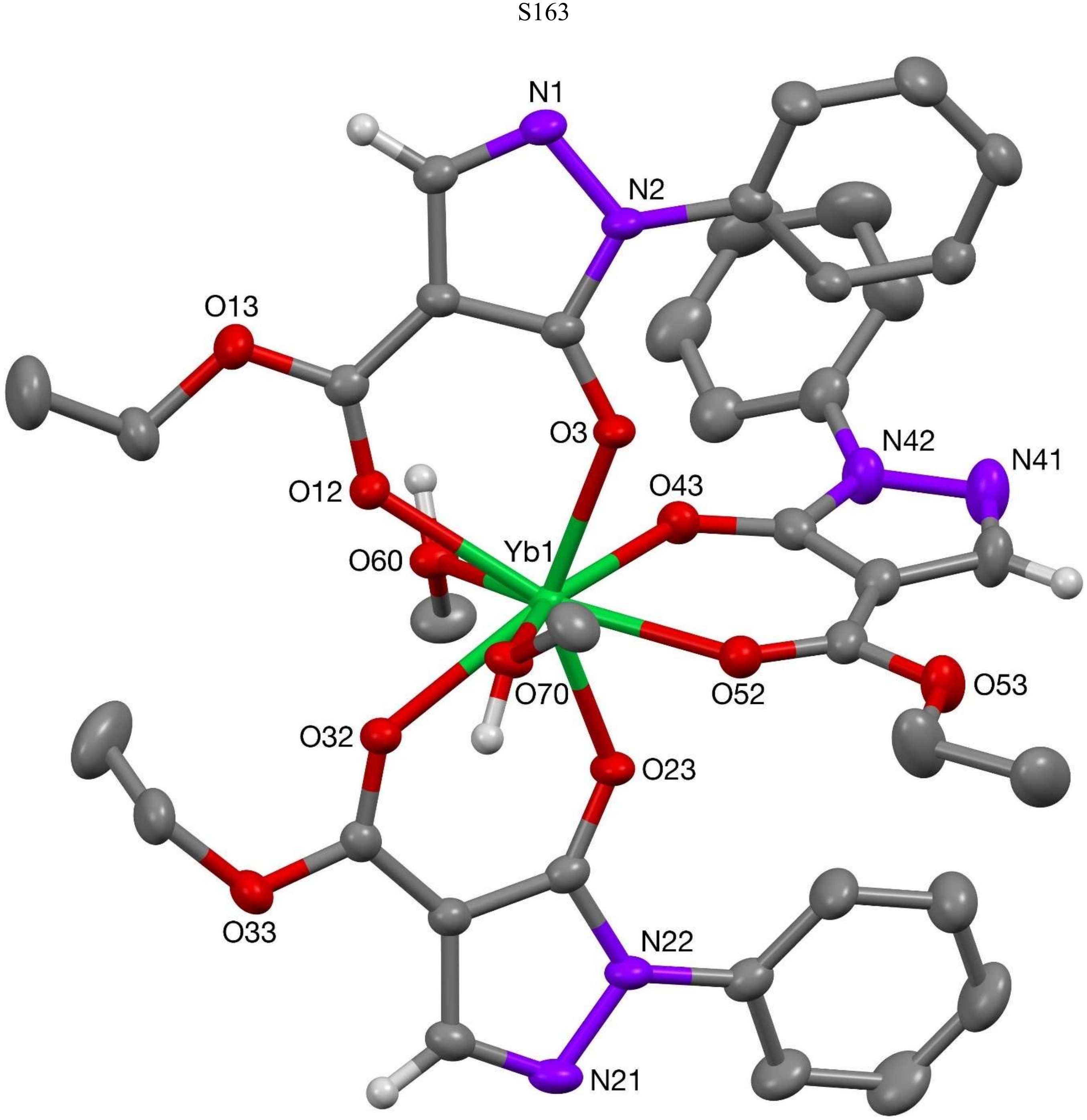

Figure S151: The crystal structure of **70** (50% probability ellipsoids).

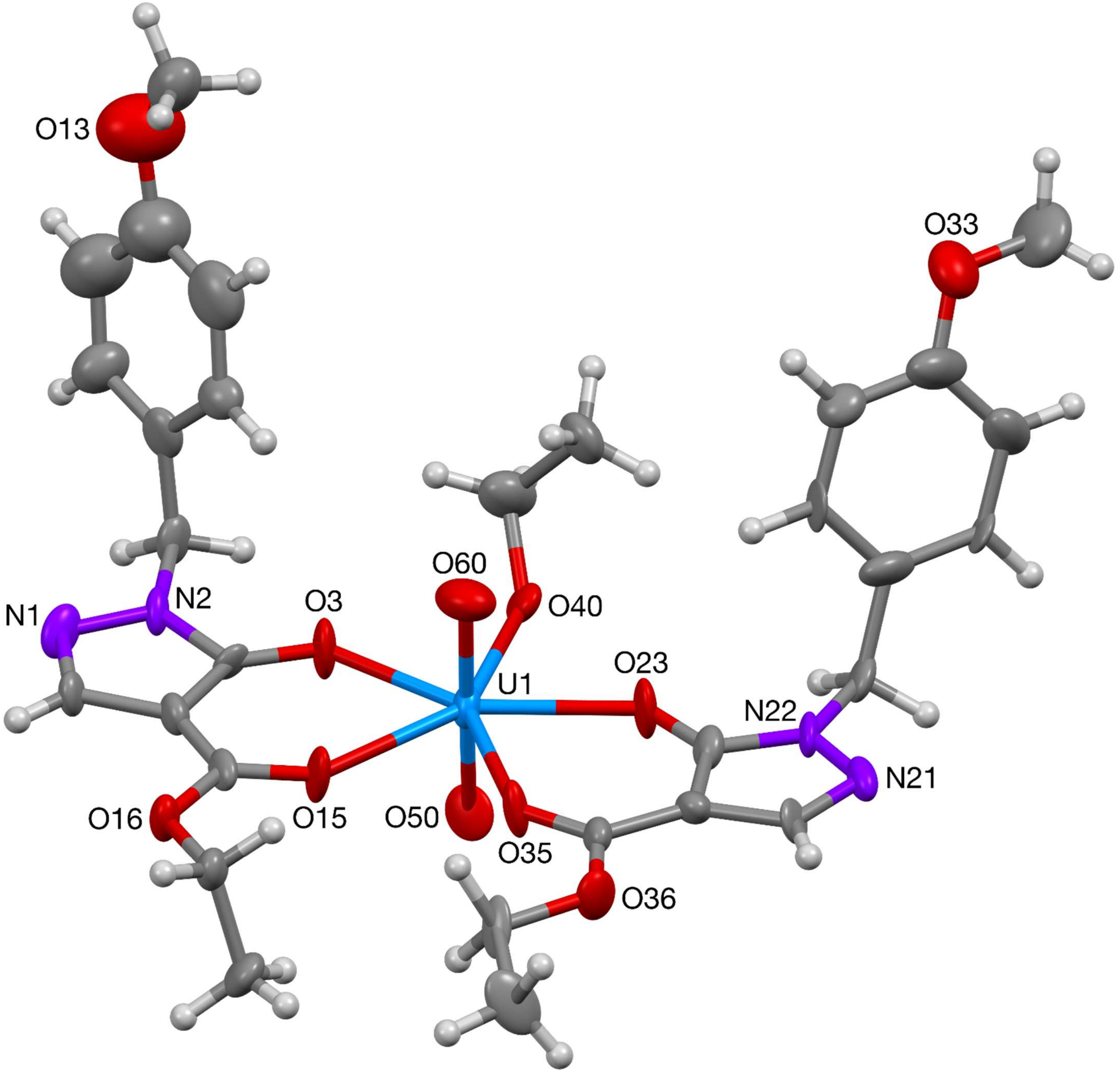

Figure S152: The crystal structure of **73** (50% probability ellipsoids).

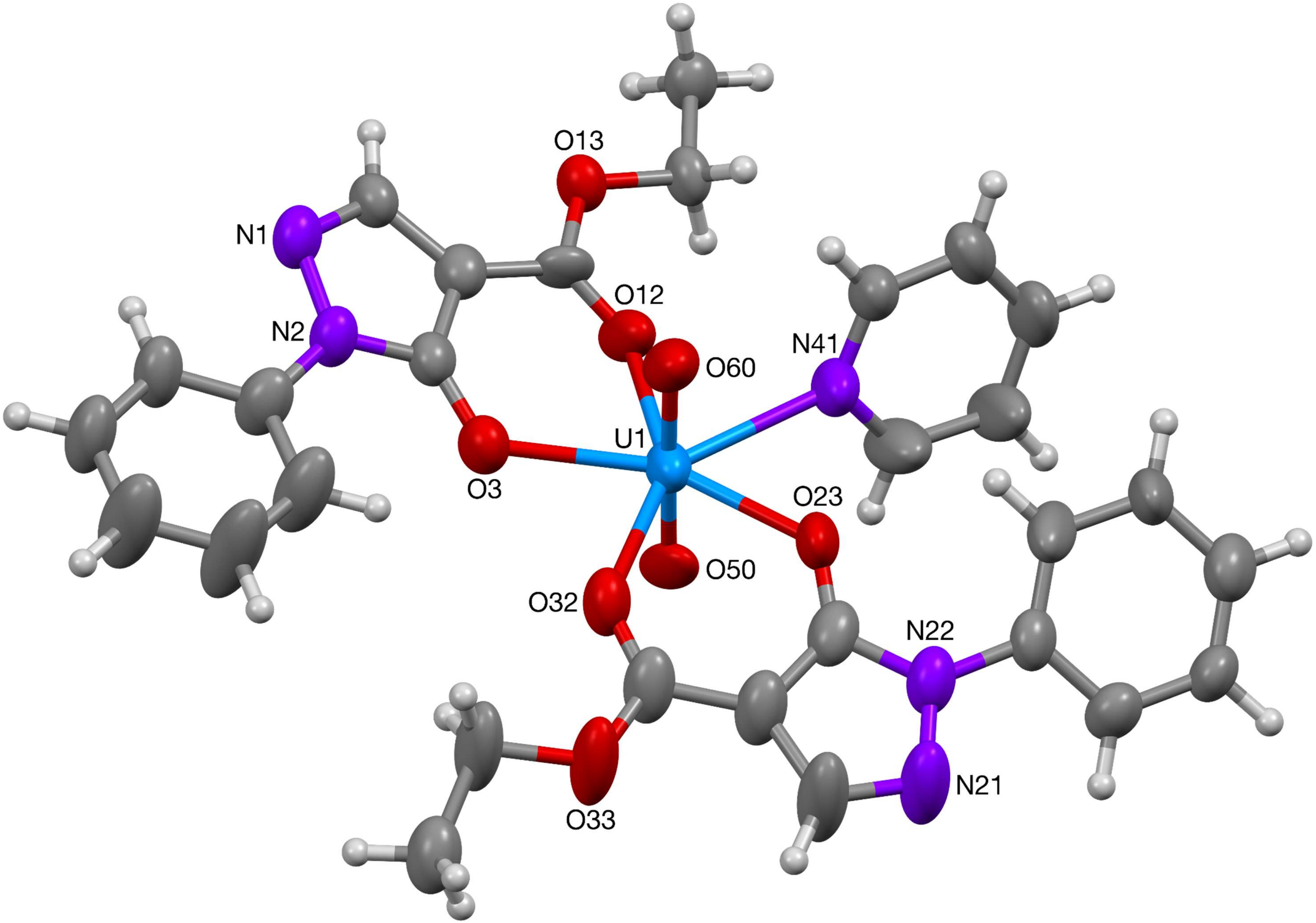

Figure S153:The crystal structure of **74** (50% probability ellipsoids).

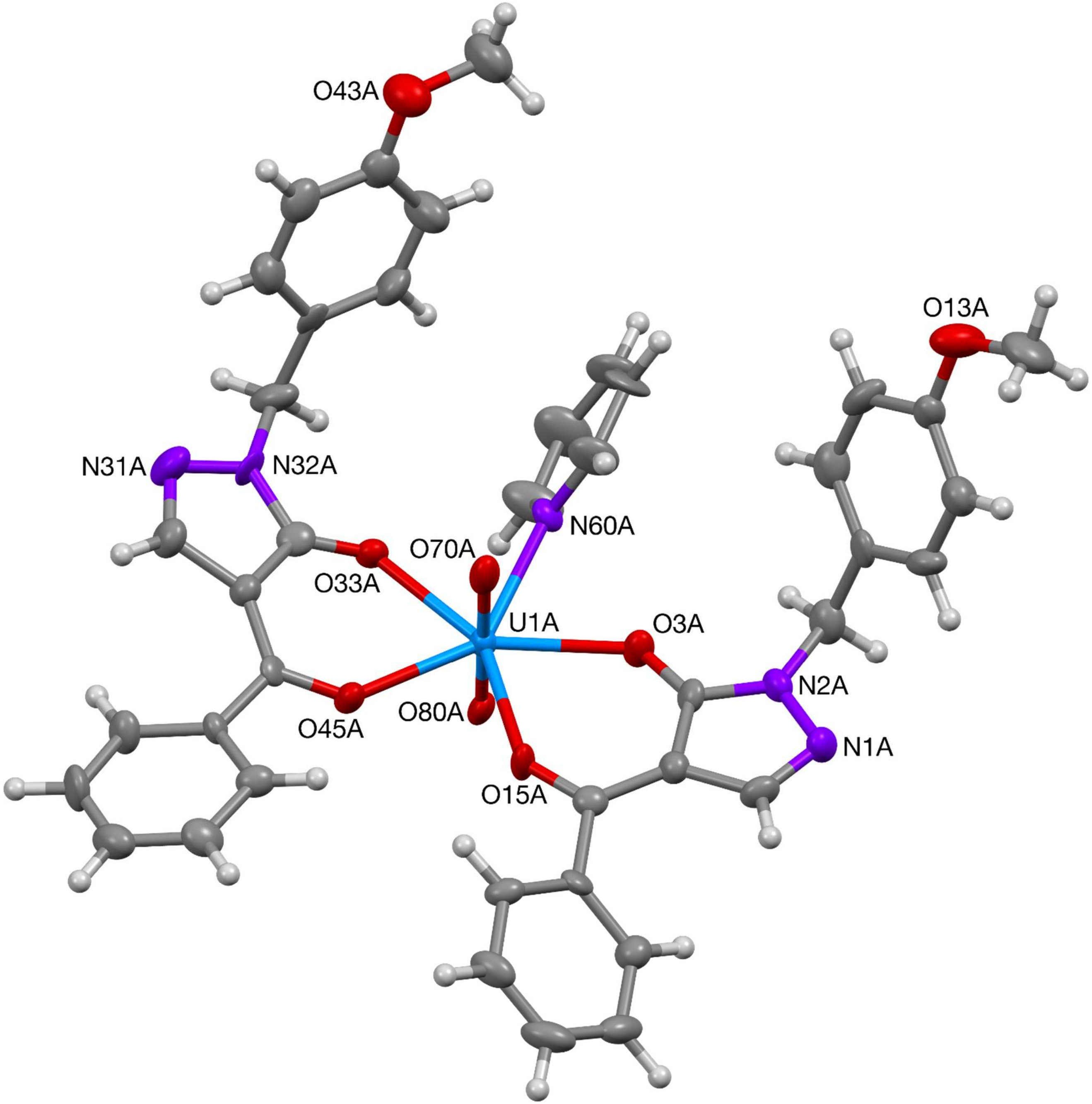

Figure S154: The structure of **75-A**, one of the two complexes present in the crystal of **75** (50% probability ellipsoids).

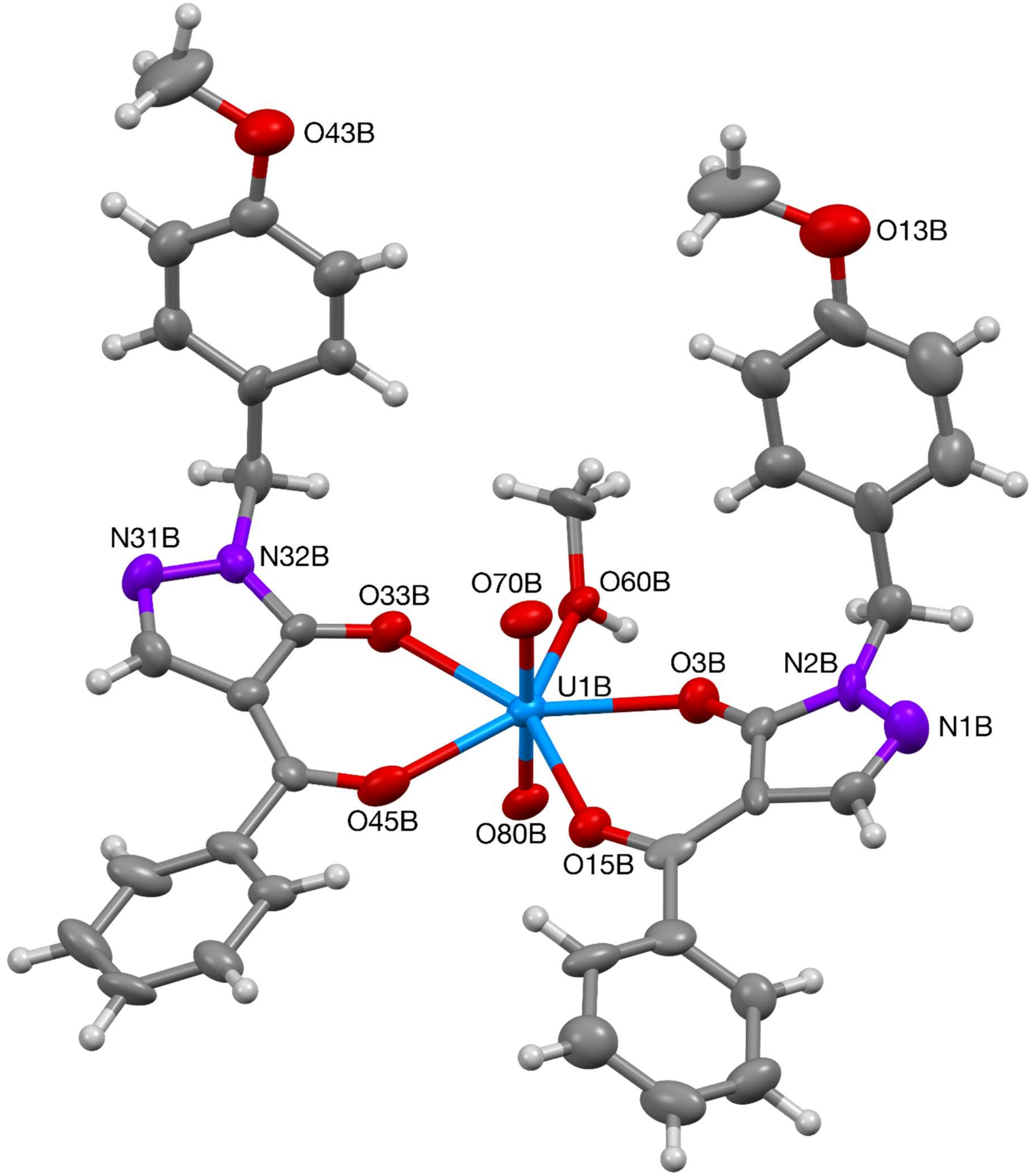

Figure S155: The structure of **75-B**, one of the two complexes present in the crystal of **75** (50% probability ellipsoids).

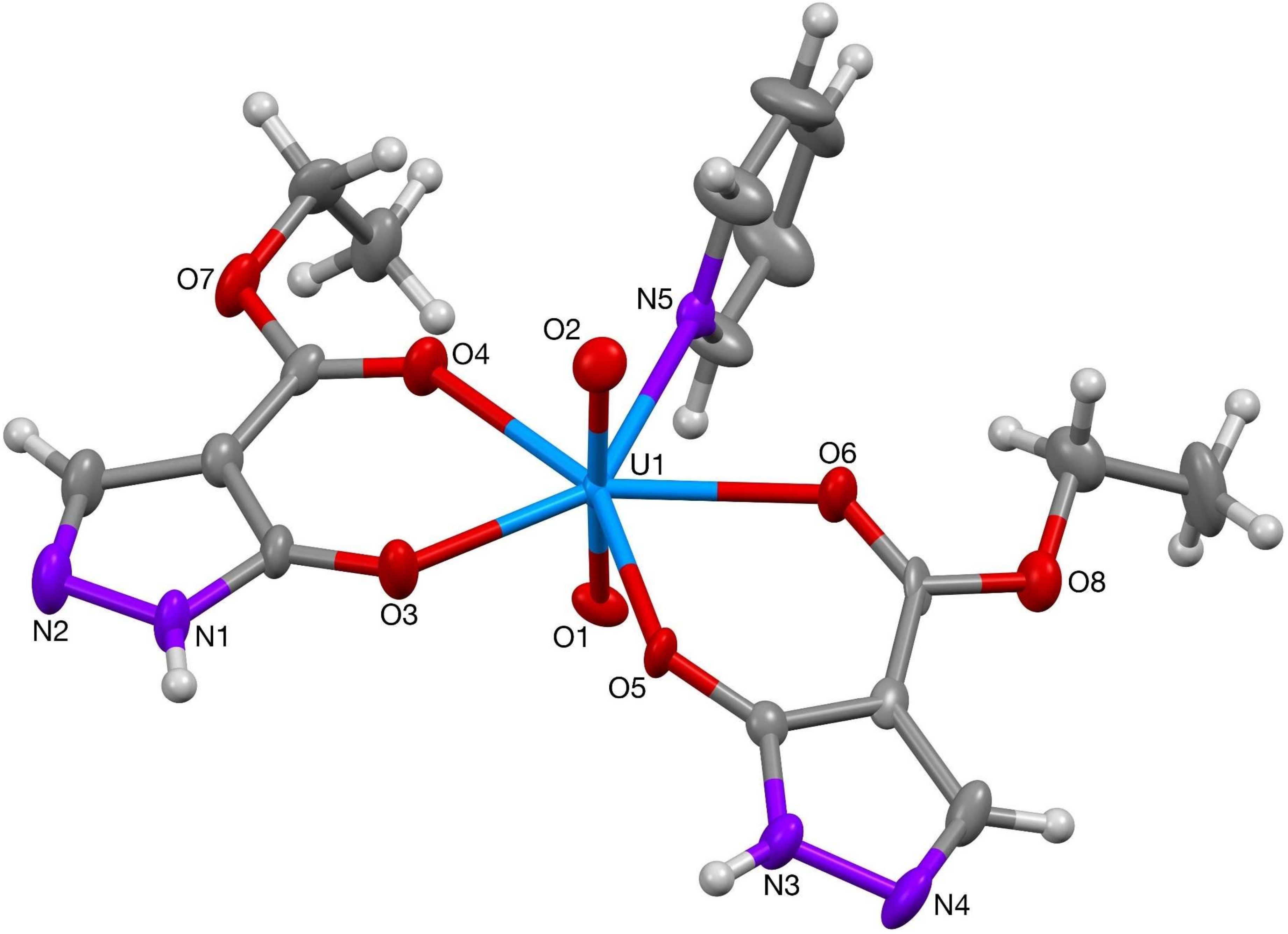

Figure S156: The crystal structure of **76** (80% probability ellipsoids).

Table 2. Bond lengths [Å] and angles [°] for **11**.

Na(1)-O(3)	2.3480(9)
Na(1)-O(3)#1	2.3480(9)
Na(1)-O(20)#1	*2.4496(12)*
Na(1)-O(20)	*2.4497(12)*
Na(1)-O(15)	*2.4555(10)*
Na(1)-O(15)#1	*2.4555(10)*
N(1)-C(5)	1.3196(19)
N(1)-N(2)	1.3777(16)
N(2)-C(3)	1.3787(17)
N(2)-C(6)	1.4551(18)
C(3)-O(3)	*1.2522(16)*
C(3)-C(4)	1.431(2)
C(4)-C(5)	1.3888(19)
C(4)-C(15)	1.4419(19)
C(6)-C(7)	1.513(2)
C(7)-C(12)	1.389(2)
C(7)-C(8)	1.393(2)
C(8)-C(9)	1.381(2)
C(9)-C(10)	1.389(2)
C(10)-O(13)	1.3701(19)
C(10)-C(11)	1.388(2)
C(11)-C(12)	1.390(2)
O(13)-C(14)	1.425(2)
C(15)-O(15)	*1.2227(16)*
C(15)-O(16)	1.3441(16)
O(16)-C(17)	1.4477(17)
C(17)-C(18)	1.503(2)
O(3)-Na(1)-O(3)#1	180.0
O(3)-Na(1)-O(20)#1	*94.26(4)*
O(3)#1-Na(1)-O(20)#1	85.74(4)
O(3)-Na(1)-O(20)	*85.74(4)*
O(3)#1-Na(1)-O(20)	94.26(4)
O(20)#1-Na(1)-O(20)	180.0
O(3)-Na(1)-O(15)	*80.33(3)*
O(3)#1-Na(1)-O(15)	99.67(3)
O(20)#1-Na(1)-O(15)	90.34(3)
O(20)-Na(1)-O(15)	89.66(3)
O(3)-Na(1)-O(15)#1	*99.67(3)*
O(3)#1-Na(1)-O(15)#1	80.33(3)
O(20)#1-Na(1)-O(15)#1	89.67(3)
O(20)-Na(1)-O(15)#1	90.34(3)
O(15)-Na(1)-O(15)#1	180.0
C(5)-N(1)-N(2)	106.73(10)
N(1)-N(2)-C(3)	111.15(11)
N(1)-N(2)-C(6)	120.19(11)
C(3)-N(2)-C(6)	126.02(11)
O(3)-C(3)-N(2)	123.20(12)
O(3)-C(3)-C(4)	132.28(12)
N(2)-C(3)-C(4)	104.51(11)
C(3)-O(3)-Na(1)	122.44(9)
C(5)-C(4)-C(3)	106.25(12)
C(5)-C(4)-C(15)	127.06(13)
C(3)-C(4)-C(15)	126.69(12)
N(1)-C(5)-C(4)	111.32(12)
N(2)-C(6)-C(7)	112.06(12)
C(12)-C(7)-C(8)	118.04(14)
C(12)-C(7)-C(6)	120.49(12)
C(8)-C(7)-C(6)	121.45(13)
C(9)-C(8)-C(7)	121.20(14)
C(8)-C(9)-C(10)	120.12(14)
O(13)-C(10)-C(11)	124.80(14)
O(13)-C(10)-C(9)	115.59(13)
C(11)-C(10)-C(9)	119.61(14)
C(10)-C(11)-C(12)	119.60(14)
C(7)-C(12)-C(11)	121.41(13)
C(10)-O(13)-C(14)	117.40(13)
O(15)-C(15)-O(16)	122.75(12)
O(15)-C(15)-C(4)	125.81(13)
O(16)-C(15)-C(4)	111.43(11)
C(15)-O(15)-Na(1)	125.99(9)
C(15)-O(16)-C(17)	117.04(10)
O(16)-C(17)-C(18)	106.39(12)

Table S3. Bond lengths [Å] and angles [°] for **12**.

Na(1)-O(3)#1	*2.353(13)*	O(3)#1-Na(1)-O(12)#2	83.4(4)	N(1)-N(2)-C(6)	115.5(12)
Na(1)-O(3)	*2.355(12)*	O(3)-Na(1)-O(12)#2	109.1(5)	O(3)-C(3)-N(2)	123.8(13)
Na(1)-O(12)#2	*2.377(13)*	O(3)#1-Na(1)-O(20)	82.1(4)	O(3)-C(3)-C(4)	131.6(15)
Na(1)-O(20)	*2.383(13)*	*O(3)-Na(1)-O(20)*	*85.3(4)*	N(2)-C(3)-C(4)	104.5(12)
Na(1)-O(20)#1	*2.443(13)*	O(12)#2-Na(1)-O(20)	165.4(5)	C(3)-O(3)-Na(1)#3	125.0(10)
Na(1)-O(12)#1	*2.444(13)*	O(3)#1-Na(1)-O(20)#1	84.0(4)	C(3)-O(3)-Na(1)	127.1(9)
Na(1)-Na(1)#1	3.110(2)	*O(3)-Na(1)-O(20)#1*	*114.5(5)*	Na(1)#3-O(3)-Na(1)	82.7(4)
Na(1)-Na(1)#3	3.110(2)	O(12)#2-Na(1)-O(20)#1	79.4(4)	C(12)-C(4)-C(3)	127.3(15)
N(1)-C(5)	1.310(19)	O(20)-Na(1)-O(20)#1	97.0(5)	C(12)-C(4)-C(5)	126.8(15)
N(1)-N(2)	1.399(18)	*O(3)#1-Na(1)-O(12)#1*	*79.0(4)*	C(3)-C(4)-C(5)	105.5(13)
N(2)-C(3)	1.382(19)	*O(3)-Na(1)-O(12)#1*	*81.9(4)*	N(1)-C(5)-C(4)	111.1(15)
N(2)-C(6)	1.408(18)	O(12)#2-Na(1)-O(12)#1	99.9(5)	C(7)-C(6)-N(2)	122.6(14)
C(3)-O(3)	*1.250(18)*	O(20)-Na(1)-O(12)#1	79.3(5)	C(7)-C(6)-C(11)	119.1(14)
C(3)-C(4)	1.42(2)	O(20)#1-Na(1)-O(12)#1	163.0(5)	N(2)-C(6)-C(11)	118.2(14)
C(4)-C(12)	1.40(2)	O(3)#1-Na(1)-Na(1)#1	48.7(3)	C(6)-C(7)-C(8)	117.6(16)
C(4)-C(5)	1.47(2)	O(3)-Na(1)-Na(1)#1	152.0(5)	C(9)-C(8)-C(7)	122.6(18)
C(6)-C(7)	1.37(2)	O(12)#2-Na(1)-Na(1)#1	50.8(3)	C(10)-C(9)-C(8)	119.6(17)
C(6)-C(11)	1.45(2)	O(20)-Na(1)-Na(1)#1	116.5(4)	C(9)-C(10)-C(11)	123.4(16)
C(7)-C(8)	1.43(2)	O(20)#1-Na(1)-Na(1)#1	49.0(3)	C(10)-C(11)-C(6)	117.3(16)
C(8)-C(9)	1.33(3)	O(12)#1-Na(1)-Na(1)#1	117.7(4)	O(12)-C(12)-O(13)	120.3(16)
C(9)-C(10)	1.33(2)	O(3)#1-Na(1)-Na(1)#3	110.7(4)	O(12)-C(12)-C(4)	124.7(17)
C(10)-C(11)	1.38(2)	O(3)-Na(1)-Na(1)#3	48.6(3)	O(13)-C(12)-C(4)	114.9(15)
C(12)-O(12)	*1.24(2)*	O(12)#2-Na(1)-Na(1)#3	138.2(3)	C(12)-O(12)-Na(1)#4	144.2(13)
C(12)-O(13)	1.32(2)	O(20)-Na(1)-Na(1)#3	50.7(3)	C(12)-O(12)-Na(1)#3	127.2(11)
O(13)-C(14')	1.44(3)	O(20)#1-Na(1)-Na(1)#3	139.1(3)	Na(1)#4-O(12)-Na(1)#3	80.3(4)
O(13)-C(14)	1.45(3)	O(12)#1-Na(1)-Na(1)#3	48.9(3)	C(12)-O(13)-C(14')	124(2)
C(14)-C(15)	1.47(3)	Na(1)#1-Na(1)-Na(1)#3	159.3(5)	C(12)-O(13)-C(14)	112.5(17)
C(14')-C(15')	1.47(3)	C(5)-N(1)-N(2)	105.7(13)	O(13)-C(14)-C(15)	105(2)
		C(3)-N(2)-N(1)	113.2(11)	O(13)-C(14')-C(15')	105(2)
O(3)#1-Na(1)-O(3)	158.8(5)	C(3)-N(2)-C(6)	131.3(12)	Na(1)-O(20)-Na(1)#3	80.2(4)

Table S4. Bond lengths [Å] and angles [°] for **14**.

Na(1)-O(3)	*2.3133(17)*			C(3)-O(3)-Na(1)#3	112.34(14)
Na(1)-O(6)	*2.3542(19)*	*O(3)-Na(1)-O(6)*	*83.21(6)*	Na(1)-O(3)-Na(1)#3	93.93(6)
Na(1)-N(1)#1	*2.375(2)*	O(3)-Na(1)-N(1)#1	165.25(8)	C(3)-C(4)-C(5)	105.2(2)
Na(1)-N(12)#2	*2.399(2)*	O(6)-Na(1)-N(1)#1	82.75(7)	C(3)-C(4)-C(6)	126.9(2)
Na(1)-O(16)	*2.5258(19)*	*O(3)-Na(1)-N(12)#2*	*100.62(7)*	C(5)-C(4)-C(6)	127.9(2)
Na(1)-O(3)#3	2.6246(19)	O(6)-Na(1)-N(12)#2	169.15(7)	N(1)-C(5)-C(4)	113.0(2)
Na(1)-Na(1)#3	3.6155(18)	N(1)#1-Na(1)-N(12)#2	92.42(8)	O(6)-C(6)-O(7)	121.6(2)
N(1)-C(5)	1.313(3)	*O(3)-Na(1)-O(16)*	*95.02(7)*	O(6)-C(6)-C(4)	126.0(2)
N(1)-N(2)	1.378(3)	O(6)-Na(1)-O(16)	110.18(7)	O(7)-C(6)-C(4)	112.4(2)
N(2)-C(3)	1.367(3)	N(1)#1-Na(1)-O(16)	94.06(7)	C(6)-O(6)-Na(1)	125.33(16)
C(3)-O(3)	*1.282(3)*	N(12)#2-Na(1)-O(16)	79.76(7)	C(6)-O(7)-C(8)	117.6(2)
C(3)-C(4)	1.412(3)	O(3)-Na(1)-O(3)#3	85.99(6)	O(7)-C(8)-C(9)	111.6(3)
C(4)-C(5)	1.414(3)	N(1)#1-Na(1)-O(3)#3	89.64(7)	C(15)-N(11)-N(12)	113.18(18)
C(4)-C(6)	1.434(3)	N(12)#2-Na(1)-O(3)#3	79.62(6)	C(13)-N(12)-N(11)	103.85(19)
C(6)-O(6)	1.228(3)	O(16)-Na(1)-O(3)#3	159.18(7)	C(13)-N(12)-Na(1)#5	133.52(16)
C(6)-O(7)	1.351(3)	O(3)-Na(1)-Na(1)#3	46.40(4)	N(11)-N(12)-Na(1)#5	121.91(14)
O(7)-C(8)	1.456(3)	O(6)-Na(1)-Na(1)#3	88.18(5)	N(12)-C(13)-O(13)	121.2(2)
C(8)-C(9)	1.481(5)	N(1)#1-Na(1)-Na(1)#3	128.47(7)	N(12)-C(13)-C(14)	111.9(2)
N(11)-C(15)	1.329(3)	N(12)#2-Na(1)-Na(1)#3	87.26(5)	O(13)-C(13)-C(14)	127.0(2)
N(11)-N(12)	1.362(3)	O(16)-Na(1)-Na(1)#3	136.11(6)	C(15)-C(14)-C(13)	104.1(2)
N(12)-C(13)	1.330(3)	O(3)#3-Na(1)-Na(1)#3	39.67(4)	C(15)-C(14)-C(16)	126.7(2)
C(13)-O(13)	1.338(3)	C(5)-N(1)-N(2)	103.77(19)	C(13)-C(14)-C(16)	128.9(2)
C(13)-C(14)	1.411(3)	C(5)-N(1)-Na(1)#4	130.18(18)	N(11)-C(15)-C(14)	107.0(2)
C(14)-C(15)	1.385(3)	N(2)-N(1)-Na(1)#4	122.74(15)	O(16)-C(16)-O(17)	123.5(2)
C(14)-C(16)	1.452(3)	C(3)-N(2)-N(1)	113.87(18)	O(16)-C(16)-C(14)	125.6(2)
C(16)-O(16)	1.217(3)	O(3)-C(3)-N(2)	123.4(2)	O(17)-C(16)-C(14)	110.90(19)
C(16)-O(17)	1.343(3)	O(3)-C(3)-C(4)	132.3(2)	C(16)-O(16)-Na(1)	151.32(17)
O(17)-C(18)	1.447(3)	N(2)-C(3)-C(4)	104.2(2)	C(16)-O(17)-C(18)	116.96(18)
C(18)-C(19)	1.509(4)	C(3)-O(3)-Na(1)	119.31(14)	O(17)-C(18)-C(19)	106.8(2)

Table S5. Bond lengths [Å] and angles [°] for **15**.

Mg(1)-O(3)	*2.0211(10)*
Mg(1)-O(3)#1	*2.0211(10)*
Mg(1)-O(20)	*2.0835(11)*
Mg(1)-O(20)#1	*2.0835(11)*
Mg(1)-O(15)	*2.1295(11)*
Mg(1)-O(15)#1	*2.1295(11)*
N(1)-C(5)	1.314(2)
N(1)-N(2)	1.3866(18)
N(2)-C(3)	1.3639(19)
N(2)-C(6)	1.4434(19)
C(3)-O(3)	*1.2725(18)*
C(3)-C(4)	1.418(2)
C(4)-C(5)	1.411(2)
C(4)-C(15)	1.421(2)
C(6)-C(7)	1.513(2)
C(7)-C(8)	1.379(2)
C(7)-C(12)	1.392(2)
C(8)-C(9)	1.395(2)
C(9)-C(10)	1.381(2)
C(10)-O(13)	1.373(2)
C(10)-C(11)	1.386(2)
C(11)-C(12)	1.379(2)
O(13)-C(14)	1.425(2)
C(15)-O(15)	*1.2376(18)*
C(15)-O(16)	1.3424(19)
O(16)-C(17)	1.4506(19)
C(17)-C(18)	1.496(3)
O(20)-C(21)	1.432(2)
C(21)-C(22)	1.501(3)
O(3)-Mg(1)-O(3)#1	180.0
O(3)-Mg(1)-O(20)	*91.32(4)*
O(3)#1-Mg(1)-O(20)	*88.68(4)*
O(3)-Mg(1)-O(20)#1	*88.68(4)*
O(3)#1-Mg(1)-O(20)#1	*91.32(4)*
O(20)-Mg(1)-O(20)#1	180.0
O(3)-Mg(1)-O(15)	*89.77(4)*
O(3)#1-Mg(1)-O(15)	*90.23(4)*
O(20)-Mg(1)-O(15)	90.77(5)
O(20)#1-Mg(1)-O(15)	89.22(5)
O(3)-Mg(1)-O(15)#1	*90.23(4)*
O(3)#1-Mg(1)-O(15)#1	*89.77(4)*
O(20)-Mg(1)-O(15)#1	89.23(5)
O(20)#1-Mg(1)-O(15)#1	90.78(5)
O(15)-Mg(1)-O(15)#1	180.0
C(5)-N(1)-N(2)	105.06(12)
C(3)-N(2)-N(1)	112.57(12)
C(3)-N(2)-C(6)	126.36(13)
N(1)-N(2)-C(6)	120.61(12)
O(3)-C(3)-N(2)	123.27(14)
O(3)-C(3)-C(4)	131.91(14)
N(2)-C(3)-C(4)	104.82(13)
C(3)-O(3)-Mg(1)	121.87(10)
C(5)-C(4)-C(3)	105.48(13)
C(5)-C(4)-C(15)	131.32(15)
C(3)-C(4)-C(15)	123.20(14)
N(1)-C(5)-C(4)	112.06(14)
N(2)-C(6)-C(7)	113.84(13)
C(8)-C(7)-C(12)	117.90(15)
C(8)-C(7)-C(6)	123.14(14)
C(12)-C(7)-C(6)	118.95(14)
C(7)-C(8)-C(9)	121.39(15)
C(10)-C(9)-C(8)	119.51(16)
O(13)-C(10)-C(9)	124.62(16)
O(13)-C(10)-C(11)	115.40(14)
C(9)-C(10)-C(11)	119.98(16)
C(12)-C(11)-C(10)	119.64(15)
C(11)-C(12)-C(7)	121.58(15)
C(10)-O(13)-C(14)	117.31(13)
O(15)-C(15)-O(16)	121.27(14)
O(15)-C(15)-C(4)	124.64(14)
O(16)-C(15)-C(4)	114.08(13)
C(15)-O(15)-Mg(1)	126.47(10)
C(15)-O(16)-C(17)	116.27(12)
O(16)-C(17)-C(18)	107.77(14)
C(21)-O(20)-Mg(1)	130.31(10)
O(20)-C(21)-C(22)	111.46(15)

Table S6. Bond lengths [Å] and angles [°] for **16**.

Mg(1)-O(3)	*2.0473(19)*
Mg(1)-O(3)#1	*2.0473(19)*
Mg(1)-O(12)	*2.075(3)*
Mg(1)-O(12)#1	*2.075(3)*
Mg(1)-O(20)	*2.107(3)*
Mg(1)-O(20)#1	*2.107(3)*
N(1)-C(5)	1.323(4)
N(1)-N(2)	1.384(3)
N(2)-C(3)	1.390(3)
N(2)-C(6)	1.403(4)
C(3)-O(3)	*1.268(4)*
C(3)-C(4)	1.399(5)
C(4)-C(5)	1.415(4)
C(4)-C(12)	1.424(4)
C(6)-C(7)	1.392(4)
C(6)-C(11)	1.393(5)
C(7)-C(8)	1.382(5)
C(8)-C(9)	1.367(6)
C(9)-C(10)	1.391(6)
C(10)-C(11)	1.391(5)
C(12)-O(12)	*1.233(4)*
C(12)-O(13)	1.327(4)
O(13)-C(14')	1.449(14)
O(13)-C(14)	1.463(5)
C(14)-C(15)	1.452(12)
C(14')-C(15')	1.465(18)
O(20)-C(21)	1.502(12)
C(21)-C(22)	1.53(2)
O(3)-Mg(1)-O(3)#1	180.0
O(3)-Mg(1)-O(12)	*89.49(9)*
O(3)#1-Mg(1)-O(12)	*90.51(9)*
O(3)-Mg(1)-O(12)#1	*90.51(9)*
O(3)#1-Mg(1)-O(12)#1	*89.49(9)*
O(12)-Mg(1)-O(12)#1	180.0
O(3)-Mg(1)-O(20)	*90.17(10)*
O(3)#1-Mg(1)-O(20)	*89.83(10)*
O(12)-Mg(1)-O(20)	90.34(14)
O(12)#1-Mg(1)-O(20)	89.65(14)
O(3)-Mg(1)-O(20)#1	*89.83(10)*
O(3)#1-Mg(1)-O(20)#1	*90.17(10)*
O(12)-Mg(1)-O(20)#1	89.66(14)
O(12)#1-Mg(1)-O(20)#1	90.35(14)
O(20)-Mg(1)-O(20)#1	180.0
C(5)-N(1)-N(2)	105.9(2)
N(1)-N(2)-C(3)	111.0(2)
N(1)-N(2)-C(6)	119.6(2)
C(3)-N(2)-C(6)	129.4(2)
O(3)-C(3)-N(2)	123.5(3)
O(3)-C(3)-C(4)	130.8(2)
N(2)-C(3)-C(4)	105.7(2)
C(3)-O(3)-Mg(1)	121.93(19)
C(3)-C(4)-C(5)	105.7(2)
C(3)-C(4)-C(12)	124.0(3)
C(5)-C(4)-C(12)	130.2(3)
N(1)-C(5)-C(4)	111.7(3)
C(7)-C(6)-C(11)	119.4(3)
C(7)-C(6)-N(2)	119.3(3)
C(11)-C(6)-N(2)	121.3(3)
C(8)-C(7)-C(6)	119.8(4)
C(9)-C(8)-C(7)	121.6(3)
C(8)-C(9)-C(10)	119.0(4)
C(9)-C(10)-C(11)	120.7(4)
C(10)-C(11)-C(6)	119.6(3)
O(12)-C(12)-O(13)	121.5(3)
O(12)-C(12)-C(4)	123.8(3)
O(13)-C(12)-C(4)	114.6(3)
C(12)-O(12)-Mg(1)	128.6(2)
C(12)-O(13)-C(14')	118.1(10)
C(12)-O(13)-C(14)	116.4(5)
C(15)-C(14)-O(13)	107.2(7)
O(13)-C(14')-C(15')	107.2(13)
C(21)-O(20)-Mg(1)	128.8(4)
O(20)-C(21)-C(22)	122.3(11)

Table S7. Bond lengths [Å] and angles [°] for **17**.

Mg(1)-O(3)#1	*2.050(2)*	C(19)-C(20)	1.373(6)	C(15)-C(4)-C(3)	124.9(3)
Mg(1)-O(3)	*2.050(2)*	C(20)-C(21)	1.391(5)	C(5)-C(4)-C(3)	105.0(3)
Mg(1)-O(30)#1	*2.099(2)*	O(30)-C(31)	1.429(4)	N(1)-C(5)-C(4)	112.2(3)
Mg(1)-O(30)	*2.099(2)*	C(31)-C(32)	1.506(6)	N(2)-C(6)-C(7)	114.1(3)
Mg(1)-O(15)#1	*2.107(2)*			C(8)-C(7)-C(12)	117.6(3)
Mg(1)-O(15)	*2.107(2)*	O(3)#1-Mg(1)-O(3)	180.0	C(8)-C(7)-C(6)	120.6(3)
N(1)-C(5)	1.315(4)	*O(3)#1-Mg(1)-O(30)#1*	*92.40(8)*	C(12)-C(7)-C(6)	121.7(3)
N(1)-N(2)	1.395(4)	*O(3)-Mg(1)-O(30)#1*	*87.60(8)*	C(7)-C(8)-C(9)	122.5(3)
N(2)-C(3)	1.370(4)	*O(3)#1-Mg(1)-O(30)*	*87.60(8)*	C(10)-C(9)-C(8)	118.2(3)
N(2)-C(6)	1.444(4)	*O(3)-Mg(1)-O(30)*	*92.40(8)*	C(11)-C(10)-O(13)	116.1(3)
C(3)-O(3)	*1.268(4)*	O(30)#1-Mg(1)-O(30)	180.00(8)	C(11)-C(10)-C(9)	120.4(3)
C(3)-C(4)	1.424(4)	*O(3)#1-Mg(1)-O(15)#1*	*89.35(8)*	O(13)-C(10)-C(9)	123.5(3)
C(4)-C(15)	1.415(4)	*O(3)-Mg(1)-O(15)#1*	*90.66(8)*	C(10)-C(11)-C(12)	120.3(3)
C(4)-C(5)	1.422(4)	O(30)#1-Mg(1)-O(15)#1	91.88(9)	C(11)-C(12)-C(7)	120.9(3)
C(6)-C(7)	1.507(4)	O(30)-Mg(1)-O(15)#1	88.12(9)	C(10)-O(13)-C(14)	116.6(3)
C(7)-C(8)	1.382(4)	*O(3)#1-Mg(1)-O(15)*	*90.66(8)*	O(15)-C(15)-C(4)	122.0(3)
C(7)-C(12)	1.397(4)	*O(3)-Mg(1)-O(15)*	*89.34(8)*	O(15)-C(15)-C(16)	118.0(3)
C(8)-C(9)	1.393(4)	O(30)#1-Mg(1)-O(15)	88.12(9)	C(4)-C(15)-C(16)	120.0(3)
C(9)-C(10)	1.392(4)	O(30)-Mg(1)-O(15)	91.88(9)	C(15)-O(15)-Mg(1)	129.2(2)
C(10)-C(11)	1.378(5)	O(15)#1-Mg(1)-O(15)	180.00(14)	C(21)-C(16)-C(17)	119.0(3)
C(10)-O(13)	1.380(4)	C(5)-N(1)-N(2)	105.5(2)	C(21)-C(16)-C(15)	121.7(3)
C(11)-C(12)	1.381(5)	C(3)-N(2)-N(1)	111.9(2)	C(17)-C(16)-C(15)	119.0(3)
O(13)-C(14)	1.422(4)	C(3)-N(2)-C(6)	127.7(3)	C(18)-C(17)-C(16)	119.9(3)
C(15)-O(15)	*1.255(4)*	N(1)-N(2)-C(6)	120.0(2)	C(19)-C(18)-C(17)	120.2(3)
C(15)-C(16)	1.503(4)	O(3)-C(3)-N(2)	123.4(3)	C(20)-C(19)-C(18)	120.4(3)
C(16)-C(21)	1.381(5)	O(3)-C(3)-C(4)	131.1(3)	C(19)-C(20)-C(21)	119.5(4)
C(16)-C(17)	1.397(4)	N(2)-C(3)-C(4)	105.4(2)	C(16)-C(21)-C(20)	121.0(3)
C(17)-C(18)	1.385(5)	C(3)-O(3)-Mg(1)	121.62(19)	C(31)-O(30)-Mg(1)	128.10(17)
C(18)-C(19)	1.383(6)	C(15)-C(4)-C(5)	130.1(3)	O(30)-C(31)-C(32)	110.8(3)

Table S8. Bond lengths [Å] and angles [°] for **18**.

Mg(1)-O(3)	*2.0635(9)*	*O(3)#1-Mg(1)-O(10)#1*	*90.15(4)*
Mg(1)-O(3)#1	*2.0635(9)*	O(6)#1-Mg(1)-O(10)#1	90.62(4)
Mg(1)-O(6)#1	*2.0866(9)*	O(6)-Mg(1)-O(10)#1	89.38(4)
Mg(1)-O(6)	*2.0866(9)*	*O(3)-Mg(1)-O(10)*	*90.15(4)*
Mg(1)-O(10)#1	*2.1063(10)*	*O(3)#1-Mg(1)-O(10)*	*89.85(4)*
Mg(1)-O(10)	*2.1063(10)*	O(6)#1-Mg(1)-O(10)	89.38(4)
N(1)-C(5)	1.3163(17)	O(6)-Mg(1)-O(10)	90.62(4)
N(1)-N(2)	1.3813(15)	O(10)#1-Mg(1)-O(10)	180.0
N(2)-C(3)	1.3535(17)	C(5)-N(1)-N(2)	105.25(10)
C(3)-O(3)	*1.2816(16)*	C(3)-N(2)-N(1)	112.58(10)
C(3)-C(4)	1.4180(18)	O(3)-C(3)-N(2)	124.26(11)
C(4)-C(5)	1.4108(18)	O(3)-C(3)-C(4)	130.53(12)
C(4)-C(6)	1.4225(18)	N(2)-C(3)-C(4)	105.21(11)
C(6)-O(6)	*1.2386(16)*	C(3)-O(3)-Mg(1)	123.04(8)
C(6)-O(7)	1.3393(16)	C(5)-C(4)-C(3)	105.21(11)
O(7)-C(8)	1.4545(16)	C(5)-C(4)-C(6)	131.31(12)
C(8)-C(9)	1.503(2)	C(3)-C(4)-C(6)	123.40(12)
O(10)-C(11)	1.436(4)	N(1)-C(5)-C(4)	111.74(12)
O(10)-C(11')	1.450(13)	O(6)-C(6)-O(7)	121.06(12)
C(11)-C(12)	1.458(7)	O(6)-C(6)-C(4)	124.50(12)
		O(7)-C(6)-C(4)	114.44(11)
O(3)-Mg(1)-O(3)#1	180.0	C(6)-O(6)-Mg(1)	129.01(8)
O(3)-Mg(1)-O(6)#1	*91.08(3)*	C(6)-O(7)-C(8)	115.57(11)
O(3)#1-Mg(1)-O(6)#1	*88.92(3)*	O(7)-C(8)-C(9)	107.17(12)
O(3)-Mg(1)-O(6)	*88.92(3)*	C(11)-O(10)-Mg(1)	128.5(3)
O(3)#1-Mg(1)-O(6)	*91.08(3)*	C(11')-O(10)-Mg(1)	129.2(8)
O(6)#1-Mg(1)-O(6)	180.0	O(10)-C(11)-C(12)	116.6(4)
O(3)-Mg(1)-O(10)#1	*89.85(4)*		

Table S9. Bond lengths [Å] and angles [°] for **19**.

Ca(1)-O(3)	*2.3116(11)*	O(20)-Ca(1)-O(15)#1	89.14(4)
Ca(1)-O(3)#1	*2.3117(11)*	*O(3)-Ca(1)-O(15)*	*81.28(4)*
Ca(1)-O(20)#1	*2.3296(12)*	*O(3)#1-Ca(1)-O(15)*	*98.72(4)*
Ca(1)-O(20)	*2.3297(12)*	O(20)#1-Ca(1)-O(15)	89.14(4)
Ca(1)-O(15)#1	*2.3724(11)*	O(20)-Ca(1)-O(15)	90.86(4)
Ca(1)-O(15)	*2.3724(11)*	O(15)#1-Ca(1)-O(15)	180.0
N(1)-C(5)	1.309(2)	C(5)-N(1)-N(2)	104.97(13)
N(1)-N(2)	1.3875(19)	C(3)-N(2)-N(1)	112.60(13)
N(2)-C(3)	1.372(2)	C(3)-N(2)-C(6)	127.16(14)
N(2)-C(6)	1.438(2)	N(1)-N(2)-C(6)	120.09(13)
C(3)-O(3)	*1.2669(19)*	O(3)-C(3)-N(2)	123.29(15)
C(3)-C(4)	1.420(2)	O(3)-C(3)-C(4)	132.40(15)
C(4)-C(5)	1.407(2)	N(2)-C(3)-C(4)	104.30(13)
C(4)-C(15)	1.419(2)	C(3)-O(3)-Ca(1)	124.46(10)
C(6)-C(7)	1.514(2)	C(5)-C(4)-C(15)	128.32(16)
C(7)-C(12)	1.378(3)	C(5)-C(4)-C(3)	105.67(15)
C(7)-C(8)	1.389(2)	C(15)-C(4)-C(3)	125.97(15)
C(8)-C(9)	1.379(2)	N(1)-C(5)-C(4)	112.44(15)
C(9)-C(10)	1.386(3)	N(2)-C(6)-C(7)	114.31(14)
C(10)-O(13)	1.371(2)	C(12)-C(7)-C(8)	117.66(17)
C(10)-C(11)	1.380(3)	C(12)-C(7)-C(6)	120.17(16)
C(11)-C(12)	1.382(3)	C(8)-C(7)-C(6)	122.18(16)
O(13)-C(14)	1.431(2)	C(9)-C(8)-C(7)	121.02(17)
C(15)-O(15)	*1.2385(19)*	C(8)-C(9)-C(10)	120.29(17)
C(15)-O(16)	1.341(2)	O(13)-C(10)-C(11)	124.34(17)
O(16)-C(17)	1.448(2)	O(13)-C(10)-C(9)	116.18(16)
C(17)-C(18)	1.494(3)	C(11)-C(10)-C(9)	119.48(17)
O(20)-C(21)	1.426(2)	C(10)-C(11)-C(12)	119.33(18)
C(21)-C(22)	1.480(3)	C(7)-C(12)-C(11)	122.21(17)
		C(10)-O(13)-C(14)	117.12(16)
O(3)-Ca(1)-O(3)#1	180.0	O(15)-C(15)-O(16)	121.34(16)
O(3)-Ca(1)-O(20)#1	*92.72(4)*	O(15)-C(15)-C(4)	125.50(16)
O(3)#1-Ca(1)-O(20)#1	*87.28(4)*	O(16)-C(15)-C(4)	113.15(14)
O(3)-Ca(1)-O(20)	*87.28(4)*	C(15)-O(15)-Ca(1)	129.63(11)
O(3)#1-Ca(1)-O(20)	*92.72(4)*	C(15)-O(16)-C(17)	117.50(14)
O(20)#1-Ca(1)-O(20)	180.0	O(16)-C(17)-C(18)	107.58(17)
O(3)-Ca(1)-O(15)#1	*98.72(4)*	C(21)-O(20)-Ca(1)	131.90(10)
O(3)#1-Ca(1)-O(15)#1	*81.28(4)*	O(20)-C(21)-C(22)	112.14(17)
O(20)#1-Ca(1)-O(15)#1	90.86(4)		

Table S10. Bond lengths [Å] and angles [°] for **20**.

Ca(1)-O(50)	2.3314(11)	C(28)-C(29)	1.378(3)	C(12)-O(13)-C(14)	116.73(12)
Ca(1)-O(23)#1	*2.3829(10)*	C(29)-C(30)	1.383(3)	O(13)-C(14)-C(15)	106.48(14)
Ca(1)-O(3)	*2.4047(11)*	C(30)-C(31)	1.387(2)	C(25)-N(21)-N(22)	105.10(12)
Ca(1)-O(23)	*2.4179(10)*	C(32)-O(32)	1.2265(18)	C(23)-N(22)-N(21)	111.64(12)
Ca(1)-O(12)	*2.4314(11)*	C(32)-O(33)	1.3466(18)	C(23)-N(22)-C(26)	129.27(13)
Ca(1)-O(32)	*2.4397(11)*	O(33)-C(34)	1.440(2)	N(21)-N(22)-C(26)	119.09(12)
Ca(1)-O(40)	2.4553(13)	C(34)-C(35)	1.503(2)	O(23)-C(23)-N(22)	124.02(14)
Ca(1)-C(23)#1	3.2270(15)	O(40)-C(41)	1.397(3)	O(23)-C(23)-C(24)	130.70(14)
Ca(1)-Ca(1)#1	*3.7523(6)*	O(40)-C(41')	1.497(17)	N(22)-C(23)-C(24)	105.24(13)
N(1)-C(5)	1.308(2)	C(41)-C(42)	1.527(5)	O(23)-C(23)-Ca(1)#1	39.68(7)
N(1)-N(2)	1.3954(19)	C(41')-C(42')	1.503(17)	N(22)-C(23)-Ca(1)#1	110.06(9)
N(2)-C(3)	1.3898(19)			C(24)-C(23)-Ca(1)#1	124.11(10)
N(2)-C(6)	1.413(2)	O(50)-Ca(1)-O(23)#1	165.77(4)	C(23)-O(23)-Ca(1)#1	120.15(9)
C(3)-O(3)	1.2673(18)	O(50)-Ca(1)-O(3)	98.38(4)	C(23)-O(23)-Ca(1)	121.56(9)
C(3)-C(4)	1.404(2)	O(23)#1-Ca(1)-O(3)	86.46(4)	*Ca(1)#1-O(23)-Ca(1)*	*102.81(4)*
C(4)-C(5)	1.403(2)	O(50)-Ca(1)-O(23)	92.19(4)	C(23)-C(24)-C(25)	105.42(14)
C(4)-C(12)	1.428(2)	*O(23)#1-Ca(1)-O(23)*	*77.19(4)*	C(23)-C(24)-C(32)	124.99(14)
C(6)-C(11)	1.387(2)	O(3)-Ca(1)-O(23)	148.62(4)	C(25)-C(24)-C(32)	129.48(14)
C(6)-C(7)	1.388(2)	O(50)-Ca(1)-O(12)	83.17(4)	N(21)-C(25)-C(24)	112.56(14)
C(7)-C(8)	1.383(3)	O(23)#1-Ca(1)-O(12)	85.03(4)	C(31)-C(26)-C(27)	120.30(15)
C(8)-C(9)	1.373(3)	O(3)-Ca(1)-O(12)	76.22(4)	C(31)-C(26)-N(22)	120.44(14)
C(9)-C(10)	1.379(3)	O(23)-Ca(1)-O(12)	75.83(4)	C(27)-C(26)-N(22)	119.26(14)
C(10)-C(11)	1.392(3)	O(50)-Ca(1)-O(32)	84.50(4)	C(28)-C(27)-C(26)	119.45(16)
C(12)-O(12)	1.2304(18)	O(23)#1-Ca(1)-O(32)	101.82(4)	C(29)-C(28)-C(27)	120.92(16)
C(12)-O(13)	1.3486(19)	O(3)-Ca(1)-O(32)	133.92(4)	C(28)-C(29)-C(30)	119.16(17)
O(13)-C(14)	1.4427(19)	O(23)-Ca(1)-O(32)	76.27(4)	C(29)-C(30)-C(31)	121.07(17)
C(14)-C(15)	1.506(2)	O(12)-Ca(1)-O(32)	148.94(4)	C(30)-C(31)-C(26)	119.09(16)
N(21)-C(25)	1.308(2)	O(50)-Ca(1)-O(40)	91.45(5)	O(32)-C(32)-O(33)	121.81(15)
N(21)-N(22)	1.3880(17)	O(23)#1-Ca(1)-O(40)	102.65(5)	O(32)-C(32)-C(24)	125.87(14)
N(22)-C(23)	1.3759(19)	O(3)-Ca(1)-O(40)	63.48(4)	O(33)-C(32)-C(24)	112.31(13)
N(22)-C(26)	1.416(2)	O(23)-Ca(1)-O(40)	146.04(4)	C(32)-O(32)-Ca(1)	130.10(10)
C(23)-O(23)	1.2865(17)	O(12)-Ca(1)-O(40)	138.11(4)	C(32)-O(33)-C(34)	116.94(13)
C(23)-C(24)	1.405(2)	O(32)-Ca(1)-O(40)	70.49(4)	O(33)-C(34)-C(35)	106.52(14)
C(24)-C(25)	1.406(2)	O(50)-Ca(1)-C(23)#1	156.81(4)	C(41)-O(40)-Ca(1)	142.57(18)
C(24)-C(32)	1.425(2)	O(23)#1-Ca(1)-C(23)#1	20.17(3)	C(41')-O(40)-Ca(1)	119.1(8)
C(26)-C(31)	1.388(2)	O(3)-Ca(1)-C(23)#1	66.95(4)	O(40)-C(41)-C(42)	111.3(3)
C(26)-C(27)	1.390(2)	O(23)-Ca(1)-C(23)#1	92.58(4)	O(40)-C(41')-C(42')	108.7(14)
C(27)-C(28)	1.384(2)				

Table S11. Bond lengths [Å] and angles [°] for **21**.

Ca(1)-O(3)#1	*2.2895(16)*	C(19)-C(20)	1.379(4)	C(5)-C(4)-C(3)	104.6(2)
Ca(1)-O(3)	*2.2895(16)*	C(20)-C(21)	1.387(4)	C(15)-C(4)-C(3)	125.3(2)
Ca(1)-O(15)#1	*2.3370(17)*	O(30)-C(31)	1.432(3)	N(1)-C(5)-C(4)	112.8(2)
Ca(1)-O(15)	*2.3370(17)*	C(31)-C(32)	1.486(4)	N(2)-C(6)-C(7)	113.11(19)
Ca(1)-O(30)#1	*2.3490(18)*			C(8)-C(7)-C(12)	117.3(2)
Ca(1)-O(30)	*2.3490(18)*	O(3)#1-Ca(1)-O(3)	180.0	C(8)-C(7)-C(6)	121.4(2)
N(1)-C(5)	1.314(3)	*O(3)#1-Ca(1)-O(15)#1*	*80.68(6)*	C(12)-C(7)-C(6)	121.3(2)
N(1)-N(2)	1.383(3)	*O(3)-Ca(1)-O(15)#1*	*99.32(6)*	C(7)-C(8)-C(9)	121.9(2)
N(2)-C(3)	1.368(3)	*O(3)#1-Ca(1)-O(15)*	*99.32(6)*	C(10)-C(9)-C(8)	119.5(2)
N(2)-C(6)	1.457(3)	*O(3)-Ca(1)-O(15)*	*80.68(6)*	C(9)-C(10)-O(13)	124.7(2)
C(3)-O(3)	*1.261(3)*	O(15)#1-Ca(1)-O(15)	180.0	C(9)-C(10)-C(11)	119.5(2)
C(3)-C(4)	1.440(3)	*O(3)#1-Ca(1)-O(30)#1*	*90.99(6)*	O(13)-C(10)-C(11)	115.9(2)
C(4)-C(5)	1.413(3)	*O(3)-Ca(1)-O(30)#1*	*89.01(6)*	C(12)-C(11)-C(10)	120.4(2)
C(4)-C(15)	1.416(3)	O(15)#1-Ca(1)-O(30)#1	93.25(7)	C(11)-C(12)-C(7)	121.3(2)
C(6)-C(7)	1.509(3)	O(15)-Ca(1)-O(30)#1	86.75(7)	C(10)-O(13)-C(14)	117.2(2)
C(7)-C(8)	1.379(3)	*O(3)#1-Ca(1)-O(30)*	*89.01(6)*	O(15)-C(15)-C(4)	122.7(2)
C(7)-C(12)	1.395(3)	*O(3)-Ca(1)-O(30)*	*90.99(6)*	O(15)-C(15)-C(16)	117.0(2)
C(8)-C(9)	1.396(3)	O(15)#1-Ca(1)-O(30)	86.75(7)	C(4)-C(15)-C(16)	120.3(2)
C(9)-C(10)	1.375(3)	O(15)-Ca(1)-O(30)	93.25(7)	C(15)-O(15)-Ca(1)	132.32(16)
C(10)-O(13)	1.379(3)	O(30)#1-Ca(1)-O(30)	180.0	C(21)-C(16)-C(17)	118.4(2)
C(10)-C(11)	1.385(4)	C(5)-N(1)-N(2)	105.16(18)	C(21)-C(16)-C(15)	122.3(2)
C(11)-C(12)	1.378(4)	C(3)-N(2)-N(1)	112.79(19)	C(17)-C(16)-C(15)	119.2(2)
O(13)-C(14)	1.418(3)	C(3)-N(2)-C(6)	126.8(2)	C(18)-C(17)-C(16)	120.5(2)
C(15)-O(15)	*1.253(3)*	N(1)-N(2)-C(6)	119.97(18)	C(19)-C(18)-C(17)	120.4(3)
C(15)-C(16)	1.495(3)	O(3)-C(3)-N(2)	123.2(2)	C(18)-C(19)-C(20)	119.9(3)
C(16)-C(21)	1.386(3)	O(3)-C(3)-C(4)	132.1(2)	C(19)-C(20)-C(21)	119.9(3)
C(16)-C(17)	1.395(3)	N(2)-C(3)-C(4)	104.71(19)	C(16)-C(21)-C(20)	120.8(3)
C(17)-C(18)	1.383(4)	C(3)-O(3)-Ca(1)	124.42(16)	C(31)-O(30)-Ca(1)	127.10(15)
C(18)-C(19)	1.374(4)	C(5)-C(4)-C(15)	130.2(2)	O(30)-C(31)-C(32)	111.1(3)

Table S12. Bond lengths [Å] and angles [°] for **22**.

Ca(1)-O(3)	*2.3048(9)*	*O(3)#1-Ca(1)-O(10)*	*89.87(3)*
Ca(1)-O(3)#1	*2.3049(9)*	O(6)-Ca(1)-O(10)	93.77(4)
Ca(1)-O(6)	*2.3055(10)*	O(6)#1-Ca(1)-O(10)	86.23(4)
Ca(1)-O(6)#1	*2.3055(10)*	*O(3)-Ca(1)-O(10)#1*	*89.87(3)*
Ca(1)-O(10)	*2.3623(10)*	*O(3)#1-Ca(1)-O(10)#1*	*90.13(3)*
Ca(1)-O(10)#1	*2.3623(10)*	O(6)-Ca(1)-O(10)#1	86.23(4)
N(1)-C(5)	1.3135(19)	O(6)#1-Ca(1)-O(10)#1	93.77(4)
N(1)-N(2)	1.3769(16)	O(10)-Ca(1)-O(10)#1	180.0
N(2)-C(3)	1.3556(17)	C(5)-N(1)-N(2)	105.13(11)
C(3)-O(3)	*1.2761(16)*	C(3)-N(2)-N(1)	113.04(11)
C(3)-C(4)	1.4262(18)	O(3)-C(3)-N(2)	123.45(12)
C(4)-C(5)	1.4120(19)	O(3)-C(3)-C(4)	131.76(12)
C(4)-C(6)	1.4259(18)	N(2)-C(3)-C(4)	104.79(11)
C(6)-O(6)	*1.2330(17)*	C(3)-O(3)-Ca(1)	124.65(8)
C(6)-O(7)	1.3394(17)	C(5)-C(4)-C(6)	130.31(12)
O(7)-C(8)	1.4593(18)	C(5)-C(4)-C(3)	104.92(11)
C(8)-C(9)	1.491(2)	C(6)-C(4)-C(3)	124.69(12)
O(10)-C(11)	1.4317(19)	N(1)-C(5)-C(4)	112.10(12)
C(11)-C(12)	1.495(2)	O(6)-C(6)-O(7)	120.09(12)
		O(6)-C(6)-C(4)	124.98(13)
O(3)-Ca(1)-O(3)#1	180.0	O(7)-C(6)-C(4)	114.92(12)
O(3)-Ca(1)-O(6)	*81.06(3)*	C(6)-O(6)-Ca(1)	131.49(9)
O(3)#1-Ca(1)-O(6)	*98.94(3)*	C(6)-O(7)-C(8)	114.67(12)
O(3)-Ca(1)-O(6)#1	*98.94(3)*	O(7)-C(8)-C(9)	107.76(14)
O(3)#1-Ca(1)-O(6)#1	*81.06(3)*	C(11)-O(10)-Ca(1)	129.93(9)
O(6)-Ca(1)-O(6)#1	180.0	O(10)-C(11)-C(12)	112.97(14)
O(3)-Ca(1)-O(10)	*90.13(3)*		

Table S13. Bond lengths [Å] and angles [°] for **24**.

Sc(1)-O(3)	*2.062(5)*	O(33)-C(34)	1.447(9)	O(12)-Sc(1)-O(32)	136.1(2)	N(22)-C(26)-C(31)	123.0(7)
Sc(1)-O(23)	*2.096(5)*	C(34)-C(35)	1.450(11)	O(52)-Sc(1)-O(32)	125.08(18)	N(22)-C(26)-C(27)	120.0(7)
Sc(1)-O(43)	*2.144(4)*	N(41)-C(45)	1.320(9)	C(5)-N(1)-N(2)	105.1(6)	C(31)-C(26)-C(27)	117.0(8)
Sc(1)-O(60)	*2.183(4)*	N(41)-N(42)	1.392(8)	C(3)-N(2)-C(6)	129.3(7)	C(28)-C(27)-C(26)	119.8(8)
Sc(1)-O(12)	*2.232(6)*	N(42)-C(43)	1.376(8)	C(3)-N(2)-N(1)	110.2(7)	C(29)-C(28)-C(27)	122.1(9)
Sc(1)-O(52)	*2.239(5)*	N(42)-C(46)	1.421(8)	C(6)-N(2)-N(1)	120.4(6)	C(28)-C(29)-C(30)	118.7(10)
Sc(1)-O(32)	*2.249(5)*	*C(43)-O(43)*	*1.275(8)*	O(3)-C(3)-N(2)	121.6(8)	C(31)-C(30)-C(29)	120.5(9)
N(1)-C(5)	1.317(12)	C(43)-C(44)	1.400(10)	O(3)-C(3)-C(4)	130.2(7)	C(30)-C(31)-C(26)	121.8(8)
N(1)-N(2)	1.402(9)	C(44)-C(45)	1.389(10)	N(2)-C(3)-C(4)	108.2(7)	O(32)-C(32)-O(33)	120.8(7)
N(2)-C(3)	1.372(10)	C(44)-C(52)	1.424(9)	C(3)-O(3)-Sc(1)	126.7(5)	O(32)-C(32)-C(24)	119.9(7)
N(2)-C(6)	1.378(11)	C(46)-C(47)	1.375(10)	C(3)-C(4)-C(12)	123.7(7)	O(33)-C(32)-C(24)	119.4(6)
C(3)-O(3)	*1.307(9)*	C(46)-C(51)	1.387(11)	C(3)-C(4)-C(5)	103.8(8)	C(32)-O(32)-Sc(1)	136.1(6)
C(3)-C(4)	1.389(12)	C(47)-C(48)	1.396(11)	C(12)-C(4)-C(5)	131.8(9)	C(32)-O(33)-C(34)	115.8(6)
C(4)-C(12)	1.397(12)	C(48)-C(49)	1.335(12)	N(1)-C(5)-C(4)	112.8(8)	O(33)-C(34)-C(35)	108.5(7)
C(4)-C(5)	1.431(11)	C(49)-C(50)	1.382(11)	N(2)-C(6)-C(11)	121.9(7)	C(45)-N(41)-N(42)	105.9(5)
C(6)-C(11)	1.381(12)	C(50)-C(51)	1.371(11)	N(2)-C(6)-C(7)	120.3(8)	C(43)-N(42)-N(41)	110.2(6)
C(6)-C(7)	1.401(11)	*C(52)-O(52)*	*1.259(8)*	C(11)-C(6)-C(7)	117.8(9)	C(43)-N(42)-C(46)	130.0(6)
C(7)-C(8)	1.371(13)	C(52)-O(53)	1.325(9)	C(8)-C(7)-C(6)	121.3(9)	N(41)-N(42)-C(46)	119.8(5)
C(8)-C(9)	1.386(14)	O(53)-C(54)	1.447(8)	C(7)-C(8)-C(9)	119.8(9)	O(43)-C(43)-N(42)	123.7(6)
C(9)-C(10)	1.386(13)	C(54)-C(55)	1.480(11)	C(10)-C(9)-C(8)	118.6(10)	O(43)-C(43)-C(44)	130.3(6)
C(10)-C(11)	1.351(13)			C(11)-C(10)-C(9)	121.6(10)	N(42)-C(43)-C(44)	106.0(6)
C(12)-O(12)	*1.274(9)*	O(3)-Sc(1)-O(23)	114.7(2)	C(10)-C(11)-C(6)	121.0(9)	C(43)-O(43)-Sc(1)	129.1(4)
C(12)-O(13)	1.326(10)	O(3)-Sc(1)-O(43)	82.9(2)	O(12)-C(12)-O(13)	121.0(8)	C(45)-C(44)-C(43)	106.1(6)
O(13)-C(14)	1.451(11)	O(23)-Sc(1)-O(43)	148.5(2)	O(12)-C(12)-C(4)	123.0(8)	C(45)-C(44)-C(52)	130.4(7)
C(14)-C(15)	1.487(14)	*O(3)-Sc(1)-O(60)*	*106.0(2)*	O(13)-C(12)-C(4)	115.9(7)	C(43)-C(44)-C(52)	123.4(6)
N(21)-C(25)	1.304(10)	O(23)-Sc(1)-O(60)	117.86(18)	C(12)-O(12)-Sc(1)	129.1(6)	N(41)-C(45)-C(44)	111.7(7)
N(21)-N(22)	1.391(8)	O(43)-Sc(1)-O(60)	78.15(17)	C(12)-O(13)-C(14)	119.3(7)	C(47)-C(46)-C(51)	119.2(7)
N(22)-C(23)	1.382(9)	*O(3)-Sc(1)-O(12)*	*83.0(2)*	O(13)-C(14)-C(15)	106.6(9)	C(47)-C(46)-N(42)	119.8(7)
N(22)-C(26)	1.399(10)	O(23)-Sc(1)-O(12)	78.8(2)	C(25)-N(21)-N(22)	106.6(6)	C(51)-C(46)-N(42)	121.0(6)
C(23)-O(23)	*1.295(8)*	O(43)-Sc(1)-O(12)	77.8(2)	C(23)-N(22)-N(21)	109.9(6)	C(46)-C(47)-C(48)	119.8(8)
C(23)-C(24)	1.379(11)	O(60)-Sc(1)-O(12)	152.96(16)	C(23)-N(22)-C(26)	129.7(6)	C(49)-C(48)-C(47)	121.1(7)
C(24)-C(25)	1.427(9)	O(3)-Sc(1)-O(52)	161.5(2)	N(21)-N(22)-C(26)	120.2(6)	C(48)-C(49)-C(50)	119.2(8)
C(24)-C(32)	1.427(11)	O(23)-Sc(1)-O(52)	75.52(19)	O(23)-C(23)-C(24)	130.3(7)	C(51)-C(50)-C(49)	121.3(8)
C(26)-C(31)	1.406(10)	*O(43)-Sc(1)-O(52)*	*81.39(18)*	O(23)-C(23)-N(22)	122.9(8)	C(50)-C(51)-C(46)	119.4(7)
C(26)-C(27)	1.408(11)	O(60)-Sc(1)-O(52)	80.28(19)	C(24)-C(23)-N(22)	106.8(6)	O(52)-C(52)-O(53)	121.1(6)
C(27)-C(28)	1.366(12)	O(12)-Sc(1)-O(52)	84.1(2)	C(23)-O(23)-Sc(1)	130.8(6)	O(52)-C(52)-C(44)	123.2(7)
C(28)-C(29)	1.361(12)	O(3)-Sc(1)-O(32)	73.20(19)	C(23)-C(24)-C(25)	105.5(7)	O(53)-C(52)-C(44)	115.7(6)
C(29)-C(30)	1.390(12)	*O(23)-Sc(1)-O(32)*	*78.6(2)*	C(23)-C(24)-C(32)	122.7(6)	C(52)-O(52)-Sc(1)	132.5(4)
C(30)-C(31)	1.344(12)	O(43)-Sc(1)-O(32)	132.8(2)	C(25)-C(24)-C(32)	131.7(8)	C(52)-O(53)-C(54)	118.4(6)
C(32)-O(32)	*1.265(8)*	O(60)-Sc(1)-O(32)	70.61(17)	N(21)-C(25)-C(24)	111.2(7)	O(53)-C(54)-C(55)	107.5(6)
C(32)-O(33)	1.307(9)						

Table S14. Bond lengths [Å] and angles [°] for **25**.

Sc(1)-O(60)#1	2.0698(14)	C(37)-C(38)	1.377(4)	O(45)-Sc(1)-Sc(1)#1	94.75(4)	C(33)-N(32)-C(36)	126.23(19)
Sc(1)-O(60)	2.0705(14)	C(37)-C(42)	1.394(3)	O(15)-Sc(1)-Sc(1)#1	97.85(4)	N(31)-N(32)-C(36)	120.94(18)
Sc(1)-O(3)	*2.0885(15)*	C(38)-C(39)	1.402(4)	C(5)-N(1)-N(2)	104.68(19)	O(33)-C(33)-N(32)	122.66(19)
Sc(1)-O(33)	*2.0960(15)*	C(39)-C(40)	1.374(4)	C(3)-N(2)-N(1)	112.54(19)	O(33)-C(33)-C(34)	131.27(19)
Sc(1)-O(45)	*2.1106(14)*	C(40)-C(41)	1.377(4)	C(3)-N(2)-C(6)	125.0(2)	N(32)-C(33)-C(34)	106.02(18)
Sc(1)-O(15)	*2.1297(14)*	C(40)-O(43)	1.377(3)	N(1)-N(2)-C(6)	121.26(19)	C(33)-O(33)-Sc(1)	126.29(13)
Sc(1)-Sc(1)#1	3.2508(7)	C(41)-C(42)	1.382(4)	O(3)-C(3)-N(2)	122.8(2)	C(45)-C(34)-C(33)	122.09(19)
N(1)-C(5)	1.311(3)	O(43)-C(44)	1.424(3)	O(3)-C(3)-C(4)	131.0(2)	C(45)-C(34)-C(35)	134.0(2)
N(1)-N(2)	1.391(3)	*C(45)-O(45)*	*1.268(2)*	N(2)-C(3)-C(4)	106.22(19)	C(33)-C(34)-C(35)	103.85(18)
N(2)-C(3)	1.345(3)	C(45)-C(46)	1.488(3)	C(3)-O(3)-Sc(1)	120.00(14)	N(31)-C(35)-C(34)	112.58(19)
N(2)-C(6)	1.451(3)	C(46)-C(47)	1.389(3)	C(15)-C(4)-C(5)	132.9(2)	N(32)-C(36)-C(37)	113.49(19)
C(3)-O(3)	*1.281(3)*	C(46)-C(51)	1.398(3)	C(15)-C(4)-C(3)	123.3(2)	C(38)-C(37)-C(42)	117.9(2)
C(3)-C(4)	1.426(3)	C(47)-C(48)	1.381(3)	C(5)-C(4)-C(3)	103.7(2)	C(38)-C(37)-C(36)	120.9(2)
C(4)-C(15)	1.408(3)	C(48)-C(49)	1.376(3)	N(1)-C(5)-C(4)	112.8(2)	C(42)-C(37)-C(36)	121.1(2)
C(4)-C(5)	1.423(3)	C(49)-C(50)	1.381(3)	N(2)-C(6)-C(7)	112.3(2)	C(37)-C(38)-C(39)	121.1(2)
C(6)-C(7)	1.514(3)	C(50)-C(51)	1.383(3)	C(8)-C(7)-C(12)	117.4(2)	C(40)-C(39)-C(38)	119.6(2)
C(7)-C(8)	1.386(3)	*O(60)-C(61)*	*1.422(4)*	C(8)-C(7)-C(6)	121.6(2)	C(39)-C(40)-C(41)	120.1(2)
C(7)-C(12)	1.390(3)	*O(60)-C(61')*	*1.433(15)*	C(12)-C(7)-C(6)	121.0(2)	C(39)-C(40)-O(43)	124.4(2)
C(8)-C(9)	1.387(4)	C(61)-C(62)	1.510(6)	C(7)-C(8)-C(9)	122.0(2)	C(41)-C(40)-O(43)	115.4(2)
C(9)-C(10)	1.387(3)	C(61')-C(62')	1.508(15)	C(8)-C(9)-C(10)	119.5(2)	C(40)-C(41)-C(42)	119.9(2)
C(10)-O(13)	1.371(3)			O(13)-C(10)-C(11)	116.5(2)	C(41)-C(42)-C(37)	121.3(2)
C(10)-C(11)	1.385(4)	*O(60)#1-Sc(1)-O(60)*	*76.53(6)*	O(13)-C(10)-C(9)	124.2(2)	C(40)-O(43)-C(44)	116.7(2)
C(11)-C(12)	1.380(4)	O(60)#1-Sc(1)-O(3)	167.13(6)	C(11)-C(10)-C(9)	119.3(2)	O(45)-C(45)-C(34)	121.05(18)
O(13)-C(14)	1.428(3)	O(60)-Sc(1)-O(3)	91.67(6)	C(12)-C(11)-C(10)	120.3(2)	O(45)-C(45)-C(46)	116.35(18)
C(15)-O(15)	*1.265(3)*	O(60)#1-Sc(1)-O(33)	96.32(6)	C(11)-C(12)-C(7)	121.4(2)	C(34)-C(45)-C(46)	122.45(18)
C(15)-C(16)	1.493(3)	O(60)-Sc(1)-O(33)	172.08(6)	C(10)-O(13)-C(14)	116.6(2)	C(45)-O(45)-Sc(1)	135.78(14)
C(16)-C(17)	1.376(4)	O(3)-Sc(1)-O(33)	95.73(6)	O(15)-C(15)-C(4)	121.5(2)	C(47)-C(46)-C(51)	119.25(19)
C(16)-C(21)	1.386(3)	O(60)#1-Sc(1)-O(45)	93.71(6)	O(15)-C(15)-C(16)	116.64(19)	C(47)-C(46)-C(45)	118.08(18)
C(17)-C(18)	1.386(4)	O(60)-Sc(1)-O(45)	93.75(6)	C(4)-C(15)-C(16)	121.79(19)	C(51)-C(46)-C(45)	122.67(19)
C(18)-C(19)	1.384(4)	O(3)-Sc(1)-O(45)	92.17(6)	C(15)-O(15)-Sc(1)	129.34(13)	C(48)-C(47)-C(46)	120.6(2)
C(19)-C(20)	1.369(4)	*O(33)-Sc(1)-O(45)*	*83.18(6)*	C(17)-C(16)-C(21)	119.6(2)	C(49)-C(48)-C(47)	119.8(2)
C(20)-C(21)	1.389(3)	O(60)#1-Sc(1)-O(15)	91.95(6)	C(17)-C(16)-C(15)	118.8(2)	C(48)-C(49)-C(50)	120.3(2)
N(31)-C(35)	1.310(3)	O(60)-Sc(1)-O(15)	100.40(6)	C(21)-C(16)-C(15)	121.6(2)	C(49)-C(50)-C(51)	120.5(2)
N(31)-N(32)	1.390(3)	*O(3)-Sc(1)-O(15)*	*85.06(6)*	C(16)-C(17)-C(18)	120.6(2)	C(50)-C(51)-C(46)	119.5(2)
N(32)-C(33)	1.345(3)	O(33)-Sc(1)-O(15)	83.08(6)	C(19)-C(18)-C(17)	119.6(3)	C(61)-O(60)-Sc(1)#1	128.1(2)
N(32)-C(36)	1.459(3)	O(45)-Sc(1)-O(15)	165.64(6)	C(20)-C(19)-C(18)	120.0(2)	C(61)-O(60)-Sc(1)	128.4(2)
C(33)-O(33)	*1.282(2)*	O(60)#1-Sc(1)-Sc(1)#1	38.27(4)	C(19)-C(20)-C(21)	120.4(2)	C(61')-O(60)-Sc(1)	128.3(10)
C(33)-C(34)	1.423(3)	O(60)-Sc(1)-Sc(1)#1	38.26(4)	C(16)-C(21)-C(20)	119.7(2)	*Sc(1)#1-O(60)-Sc(1)*	*103.47(6)*
C(34)-C(45)	1.403(3)	O(3)-Sc(1)-Sc(1)#1	129.74(5)	C(35)-N(31)-N(32)	104.72(17)	O(60)-C(61)-C(62)	111.8(3)
C(34)-C(35)	1.428(3)	O(33)-Sc(1)-Sc(1)#1	134.51(5)	C(33)-N(32)-N(31)	112.82(17)	O(60)-C(61')-C(62')	108.4(11)
C(36)-C(37)	1.505(3)						

Table S15. Bond lengths [Å] and angles [°] for **28**.

Y(1)-O(3)	*2.2899(18)*	N(41)-C(45)	1.305(5)	O(23)-Y(1)-O(52)	73.01(7)	N(21)-C(25)-C(24)	111.8(2)
Y(1)-O(23)	*2.2952(18)*	N(41)-N(42)	1.399(4)	*O(43)-Y(1)-O(52)*	*77.48(7)*	C(31)-C(26)-C(27)	120.2(3)
Y(1)-O(43)	*2.2992(19)*	N(42)-C(43)	1.376(4)	O(60)-Y(1)-O(52)	147.37(7)	C(31)-C(26)-N(22)	120.2(2)
Y(1)-O(60)	*2.3656(18)*	N(42)-C(46)	1.408(4)	O(70)-Y(1)-O(52)	73.62(7)	C(27)-C(26)-N(22)	119.6(3)
Y(1)-O(70)	*2.3761(19)*	*C(43)-O(43)*	*1.277(3)*	O(32)-Y(1)-O(52)	121.18(6)	C(28)-C(27)-C(26)	119.7(3)
Y(1)-O(32)	*2.3933(18)*	C(43)-C(44)	1.411(4)	O(12)-Y(1)-O(52)	140.39(7)	C(27)-C(28)-C(29)	120.8(3)
Y(1)-O(12)	*2.4026(18)*	C(44)-C(52)	1.409(4)	C(5)-N(1)-N(2)	105.2(2)	C(30)-C(29)-C(28)	119.3(3)
Y(1)-O(52)	*2.4340(19)*	C(44)-C(45)	1.411(4)	C(3)-N(2)-N(1)	111.1(2)	C(29)-C(30)-C(31)	120.4(4)
N(1)-C(5)	1.311(3)	C(46)-C(51)	1.370(5)	C(3)-N(2)-C(6)	128.5(2)	C(26)-C(31)-C(30)	119.6(3)
N(1)-N(2)	1.394(3)	C(46)-C(47)	1.400(5)	N(1)-N(2)-C(6)	120.4(2)	O(32)-C(32)-O(33)	121.9(2)
N(2)-C(3)	1.384(3)	C(47)-C(48)	1.380(6)	O(3)-C(3)-N(2)	123.7(2)	O(32)-C(32)-C(24)	123.5(2)
N(2)-C(6)	1.414(3)	C(48)-C(49)	1.372(7)	O(3)-C(3)-C(4)	130.8(2)	O(33)-C(32)-C(24)	114.5(2)
C(3)-O(3)	*1.268(3)*	C(49)-C(50)	1.367(6)	N(2)-C(3)-C(4)	105.5(2)	C(32)-O(32)-Y(1)	136.13(17)
C(3)-C(4)	1.404(4)	C(50)-C(51)	1.381(5)	C(3)-O(3)-Y(1)	130.84(17)	C(32)-O(33)-C(34)	116.8(2)
C(4)-C(5)	1.404(3)	*C(52)-O(52)*	*1.236(3)*	C(5)-C(4)-C(3)	105.5(2)	O(33)-C(34)-C(35)	110.8(3)
C(4)-C(12)	1.425(4)	C(52)-O(53)	1.372(4)	C(5)-C(4)-C(12)	132.4(2)	C(45)-N(41)-N(42)	104.1(3)
C(6)-C(7)	1.387(4)	O(53)-C(54)	1.439(4)	C(3)-C(4)-C(12)	121.9(2)	C(43)-N(42)-N(41)	112.0(3)
C(6)-C(11)	1.397(4)	C(54)-C(55)	1.514(5)	N(1)-C(5)-C(4)	112.8(2)	C(43)-N(42)-C(46)	126.8(3)
C(7)-C(8)	1.385(4)	O(60)-C(61)	1.419(4)	C(7)-C(6)-C(11)	119.6(3)	N(41)-N(42)-C(46)	120.2(3)
C(8)-C(9)	1.378(5)	O(70)-C(71)	1.436(3)	C(7)-C(6)-N(2)	120.3(2)	O(43)-C(43)-N(42)	122.4(3)
C(9)-C(10)	1.388(5)			C(11)-C(6)-N(2)	120.0(2)	O(43)-C(43)-C(44)	132.3(3)
C(10)-C(11)	1.383(4)	O(3)-Y(1)-O(23)	142.83(7)	C(8)-C(7)-C(6)	119.7(3)	N(42)-C(43)-C(44)	105.3(3)
C(12)-O(12)	*1.239(3)*	O(3)-Y(1)-O(43)	76.65(7)	C(9)-C(8)-C(7)	121.2(3)	C(43)-O(43)-Y(1)	128.57(19)
C(12)-O(13)	1.331(3)	O(23)-Y(1)-O(43)	77.98(7)	C(8)-C(9)-C(10)	118.8(3)	C(52)-C(44)-C(45)	130.8(3)
O(13)-C(14)	1.464(4)	O(3)-Y(1)-O(60)	111.39(6)	C(11)-C(10)-C(9)	121.0(3)	C(52)-C(44)-C(43)	124.5(3)
C(14)-C(15)	1.496(5)	O(23)-Y(1)-O(60)	86.54(7)	C(10)-C(11)-C(6)	119.6(3)	C(45)-C(44)-C(43)	104.8(3)
N(21)-C(25)	1.326(4)	O(43)-Y(1)-O(60)	73.49(7)	O(12)-C(12)-O(13)	121.8(2)	N(41)-C(45)-C(44)	113.7(3)
N(21)-N(22)	1.396(3)	O(3)-Y(1)-O(70)	82.47(6)	O(12)-C(12)-C(4)	123.1(2)	C(51)-C(46)-C(47)	119.2(3)
N(22)-C(23)	1.379(3)	O(23)-Y(1)-O(70)	106.31(6)	O(13)-C(12)-C(4)	115.0(2)	C(51)-C(46)-N(42)	121.8(3)
N(22)-C(26)	1.419(4)	O(43)-Y(1)-O(70)	147.79(7)	C(12)-O(12)-Y(1)	136.14(17)	C(47)-C(46)-N(42)	119.0(3)
C(23)-O(23)	*1.273(3)*	*O(60)-Y(1)-O(70)*	*137.84(6)*	C(12)-O(13)-C(14)	116.4(2)	C(48)-C(47)-C(46)	119.7(4)
C(23)-C(24)	1.399(4)	O(3)-Y(1)-O(32)	140.16(7)	O(13)-C(14)-C(15)	106.3(3)	C(49)-C(48)-C(47)	120.8(4)
C(24)-C(25)	1.399(4)	*O(23)-Y(1)-O(32)*	*74.62(6)*	C(25)-N(21)-N(22)	105.1(2)	C(50)-C(49)-C(48)	118.8(4)
C(24)-C(32)	1.432(4)	O(43)-Y(1)-O(32)	139.15(7)	C(23)-N(22)-N(21)	111.3(2)	C(49)-C(50)-C(51)	121.6(4)
C(26)-C(31)	1.375(4)	O(60)-Y(1)-O(32)	75.15(6)	C(23)-N(22)-C(26)	129.2(2)	C(46)-C(51)-C(50)	119.7(4)
C(26)-C(27)	1.391(4)	O(70)-Y(1)-O(32)	70.29(6)	N(21)-N(22)-C(26)	119.5(2)	O(52)-C(52)-O(53)	121.8(3)
C(27)-C(28)	1.371(5)	*O(3)-Y(1)-O(12)*	*74.12(6)*	O(23)-C(23)-N(22)	124.0(2)	O(52)-C(52)-C(44)	124.7(3)
C(28)-C(29)	1.383(6)	O(23)-Y(1)-O(12)	142.68(6)	O(23)-C(23)-C(24)	130.6(2)	O(53)-C(52)-C(44)	113.5(3)
C(29)-C(30)	1.381(5)	O(43)-Y(1)-O(12)	118.38(7)	N(22)-C(23)-C(24)	105.4(2)	C(52)-O(52)-Y(1)	132.5(2)
C(30)-C(31)	1.392(4)	O(60)-Y(1)-O(12)	68.91(6)	C(23)-O(23)-Y(1)	131.82(16)	C(52)-O(53)-C(54)	116.9(2)
C(32)-O(32)	*1.237(3)*	O(70)-Y(1)-O(12)	77.97(6)	C(23)-C(24)-C(25)	106.4(2)	O(53)-C(54)-C(55)	106.9(3)
C(32)-O(33)	1.329(3)	O(32)-Y(1)-O(12)	72.20(6)	C(23)-C(24)-C(32)	122.1(2)	C(61)-O(60)-Y(1)	127.19(18)
O(33)-C(34)	1.457(4)	O(3)-Y(1)-O(52)	75.28(6)	C(25)-C(24)-C(32)	131.3(2)	C(71)-O(70)-Y(1)	128.70(16)
C(34)-C(35)	1.481(6)						

Table S16. Bond lengths [Å] and angles [°] for **33**.

Zr(1)-O(43)	*2.093(2)*	C(29)-C(30)	1.3900	*C(63)-O(63)*	*1.296(4)*	O(55)-Zr(1)-O(75)	70.85(9)
Zr(1)-O(3)	*2.100(2)*	C(30)-O(33)	1.378(5)	C(63)-C(64)	1.404(5)	O(15)-Zr(1)-O(75)	133.95(9)
Zr(1)-O(63)	*2.103(2)*	C(30)-C(31)	1.3900	C(64)-C(65)	1.407(5)	C(5)-N(1)-N(2)	104.3(3)
Zr(1)-O(23)	*2.107(2)*	C(31)-C(32)	1.3900	C(64)-C(75)	1.415(6)	C(3)-N(2)-N(1)	111.8(3)
Zr(1)-O(35)	*2.273(2)*	O(33)-C(34)	1.400(7)	C(66)-C(67)	1.501(7)	C(3)-N(2)-C(6)	127.3(3)
Zr(1)-O(55)	*2.305(2)*	C(27')-C(28')	1.3900	C(67)-C(72)	1.377(7)	N(1)-N(2)-C(6)	120.8(3)
Zr(1)-O(15)	*2.312(2)*	C(27')-C(32')	1.3900	C(67)-C(68)	1.394(6)	O(3)-C(3)-N(2)	122.3(3)
Zr(1)-O(75)	*2.365(2)*	C(28')-C(29')	1.3900	C(68)-C(69)	1.375(8)	O(3)-C(3)-C(4)	130.4(3)
N(1)-C(5)	1.321(5)	C(29')-C(30')	1.3900	C(69)-C(70)	1.379(8)	N(2)-C(3)-C(4)	107.3(3)
N(1)-N(2)	1.394(4)	C(30')-O(33')	1.381(17)	C(70)-O(73)	1.365(7)	C(3)-O(3)-Zr(1)	128.1(2)
N(2)-C(3)	1.339(5)	C(30')-C(31')	1.3900	C(70)-C(71)	1.394(7)	C(3)-C(4)-C(5)	104.1(3)
N(2)-C(6)	1.456(5)	C(31')-C(32')	1.3900	C(71)-C(72)	1.390(7)	C(3)-C(4)-C(15)	121.7(3)
C(3)-O(3)	*1.303(4)*	O(33')-C(34')	1.405(18)	O(73)-C(74)	1.422(8)	C(5)-C(4)-C(15)	133.9(3)
C(3)-C(4)	1.400(5)	*C(35)-O(35)*	*1.239(4)*	*C(75)-O(75)*	*1.235(5)*	N(1)-C(5)-C(4)	112.5(3)
C(4)-C(5)	1.412(5)	C(35)-O(36)	1.336(4)	C(75)-O(76)	1.353(4)	N(2)-C(6)-C(7)	113.8(3)
C(4)-C(15)	1.424(5)	O(36)-C(37)	1.469(5)	O(76)-C(77)	1.445(6)	C(12)-C(7)-C(8)	118.0(4)
C(6)-C(7)	1.511(6)	C(37)-C(38)	1.478(6)	C(77)-C(78)	1.459(8)	C(12)-C(7)-C(6)	121.0(4)
C(7)-C(12)	1.381(6)	N(41)-C(45)	1.329(6)			C(8)-C(7)-C(6)	121.0(4)
C(7)-C(8)	1.394(6)	N(41)-N(42)	1.388(4)	O(43)-Zr(1)-O(3)	90.36(10)	C(9)-C(8)-C(7)	120.5(4)
C(8)-C(9)	1.380(7)	N(42)-C(43)	1.347(5)	O(43)-Zr(1)-O(63)	148.07(9)	C(8)-C(9)-C(10)	121.0(5)
C(9)-C(10)	1.383(7)	N(42)-C(46)	1.467(5)	O(3)-Zr(1)-O(63)	98.12(10)	O(13)-C(10)-C(11)	125.6(5)
C(10)-O(13)	1.368(6)	*C(43)-O(43)*	*1.293(4)*	O(43)-Zr(1)-O(23)	99.35(10)	O(13)-C(10)-C(9)	115.4(5)
C(10)-C(11)	1.376(7)	C(43)-C(44)	1.395(5)	*O(3)-Zr(1)-O(23)*	*148.78(9)*	C(11)-C(10)-C(9)	119.1(5)
C(11)-C(12)	1.393(7)	C(44)-C(45)	1.407(6)	O(63)-Zr(1)-O(23)	89.18(10)	C(10)-C(11)-C(12)	120.0(4)
O(13)-C(14)	1.441(8)	C(44)-C(55)	1.415(6)	O(43)-Zr(1)-O(35)	140.45(9)	C(7)-C(12)-C(11)	121.4(5)
C(15)-O(15)	*1.243(4)*	C(46)-C(47)	1.517(6)	O(3)-Zr(1)-O(35)	74.31(9)	C(10)-O(13)-C(14)	117.3(5)
C(15)-O(16)	1.333(4)	C(47)-C(52)	1.372(6)	O(63)-Zr(1)-O(35)	71.25(9)	O(15)-C(15)-O(16)	121.1(3)
O(16)-C(17')	1.464(18)	C(47)-C(48)	1.393(6)	*O(23)-Zr(1)-O(35)*	*79.54(9)*	O(15)-C(15)-C(4)	123.5(3)
O(16)-C(17)	1.468(5)	C(48)-C(49)	1.386(6)	*O(43)-Zr(1)-O(55)*	*77.95(9)*	O(16)-C(15)-C(4)	115.3(3)
C(17)-C(18)	1.502(8)	C(49)-C(50)	1.387(6)	O(3)-Zr(1)-O(55)	139.83(9)	C(15)-O(15)-Zr(1)	131.6(2)
C(17')-C(18')	1.51(2)	C(50)-O(53)	1.374(5)	O(63)-Zr(1)-O(55)	75.78(10)	C(15)-O(16)-C(17')	118.3(18)
N(21)-C(25)	1.315(6)	C(50)-C(51)	1.384(6)	O(23)-Zr(1)-O(55)	71.39(9)	C(15)-O(16)-C(17)	116.6(3)
N(21)-N(22)	1.389(5)	C(51)-C(52)	1.385(6)	O(35)-Zr(1)-O(55)	135.87(9)	O(16)-C(17)-C(18)	107.3(5)
N(22)-C(23)	1.354(5)	O(53)-C(54)	1.427(6)	O(43)-Zr(1)-O(15)	70.76(9)	O(16)-C(17')-C(18')	106.3(18)
N(22)-C(26)	1.434(5)	*C(55)-O(55)*	*1.239(5)*	*O(3)-Zr(1)-O(15)*	*79.59(9)*	C(25)-N(21)-N(22)	104.7(3)
C(23)-O(23)	*1.288(4)*	C(55)-O(56)	1.335(4)	O(63)-Zr(1)-O(15)	141.00(9)	C(23)-N(22)-N(21)	111.9(3)
C(23)-C(24)	1.398(5)	O(56)-C(57')	1.401(10)	O(23)-Zr(1)-O(15)	75.86(9)	C(23)-N(22)-C(26)	127.4(3)
C(24)-C(35)	1.419(5)	O(56)-C(57)	1.577(9)	O(35)-Zr(1)-O(15)	70.70(9)	N(21)-N(22)-C(26)	120.3(3)
C(24)-C(25)	1.422(5)	C(57)-C(58)	1.490(9)	O(55)-Zr(1)-O(15)	129.60(9)	O(23)-C(23)-N(22)	122.3(3)
C(26)-C(27)	1.520(5)	C(57')-C(58')	1.506(11)	O(43)-Zr(1)-O(75)	76.44(10)	O(23)-C(23)-C(24)	131.0(3)
C(26)-C(27')	1.526(11)	N(61)-C(65)	1.316(6)	O(3)-Zr(1)-O(75)	69.06(9)	N(22)-C(23)-C(24)	106.6(3)
C(27)-C(28)	1.3900	N(61)-N(62)	1.384(5)	*O(63)-Zr(1)-O(75)*	*78.03(9)*	C(23)-O(23)-Zr(1)	130.6(2)
C(27)-C(32)	1.3900	N(62)-C(63)	1.350(5)	O(23)-Zr(1)-O(75)	142.04(9)	C(23)-C(24)-C(35)	121.8(3)
C(28)-C(29)	1.3900	N(62)-C(66)	1.468(5)	O(35)-Zr(1)-O(75)	127.47(9)	C(23)-C(24)-C(25)	104.4(3)

Table S16. Part 2.

C(35)-C(24)-C(25)	133.7(4)	C(45)-N(41)-N(42)	103.8(3)	O(56)-C(57')-C(58')	102.4(7)
N(21)-C(25)-C(24)	112.3(4)	C(43)-N(42)-N(41)	112.1(3)	C(65)-N(61)-N(62)	104.3(3)
N(22)-C(26)-C(27)	114.0(3)	C(43)-N(42)-C(46)	126.4(3)	C(63)-N(62)-N(61)	112.4(3)
N(22)-C(26)-C(27')	116.6(8)	N(41)-N(42)-C(46)	121.4(3)	C(63)-N(62)-C(66)	125.4(3)
C(28)-C(27)-C(32)	120.0	O(43)-C(43)-N(42)	122.0(3)	N(61)-N(62)-C(66)	121.6(3)
C(28)-C(27)-C(26)	122.1(3)	O(43)-C(43)-C(44)	131.0(3)	O(63)-C(63)-N(62)	122.5(3)
C(32)-C(27)-C(26)	117.8(3)	N(42)-C(43)-C(44)	107.0(3)	O(63)-C(63)-C(64)	131.5(3)
C(29)-C(28)-C(27)	120.0	C(43)-O(43)-Zr(1)	130.6(2)	N(62)-C(63)-C(64)	106.0(3)
C(28)-C(29)-C(30)	120.0	C(43)-C(44)-C(45)	104.3(3)	C(63)-O(63)-Zr(1)	131.3(2)
O(33)-C(30)-C(31)	124.7(4)	C(43)-C(44)-C(55)	120.3(3)	C(63)-C(64)-C(65)	104.7(3)
O(33)-C(30)-C(29)	115.2(4)	C(45)-C(44)-C(55)	135.3(3)	C(63)-C(64)-C(75)	120.5(3)
C(31)-C(30)-C(29)	120.0	N(41)-C(45)-C(44)	112.7(3)	C(65)-C(64)-C(75)	134.9(4)
C(30)-C(31)-C(32)	120.0	N(42)-C(46)-C(47)	112.0(3)	N(61)-C(65)-C(64)	112.6(4)
C(31)-C(32)-C(27)	120.0	C(52)-C(47)-C(48)	117.8(4)	N(62)-C(66)-C(67)	111.6(4)
C(30)-O(33)-C(34)	119.3(6)	C(52)-C(47)-C(46)	121.5(4)	C(72)-C(67)-C(68)	118.1(5)
C(28')-C(27')-C(32')	120.0	C(48)-C(47)-C(46)	120.7(4)	C(72)-C(67)-C(66)	120.6(4)
C(28')-C(27')-C(26)	121.5(12)	C(49)-C(48)-C(47)	121.3(4)	C(68)-C(67)-C(66)	121.3(4)
C(32')-C(27')-C(26)	118.5(12)	C(48)-C(49)-C(50)	119.7(4)	C(69)-C(68)-C(67)	121.2(5)
C(29')-C(28')-C(27')	120.0	O(53)-C(50)-C(51)	124.7(4)	C(68)-C(69)-C(70)	120.2(4)
C(28')-C(29')-C(30')	120.0	O(53)-C(50)-C(49)	115.7(4)	O(73)-C(70)-C(69)	115.9(4)
O(33')-C(30')-C(29')	117.8(19)	C(51)-C(50)-C(49)	119.6(4)	O(73)-C(70)-C(71)	124.4(5)
O(33')-C(30')-C(31')	121.9(19)	C(50)-C(51)-C(52)	119.6(4)	C(69)-C(70)-C(71)	119.7(5)
C(29')-C(30')-C(31')	120.0	C(47)-C(52)-C(51)	122.0(4)	C(72)-C(71)-C(70)	119.2(5)
C(32')-C(31')-C(30')	120.0	C(50)-O(53)-C(54)	117.5(4)	C(67)-C(72)-C(71)	121.6(4)
C(31')-C(32')-C(27')	120.0	O(55)-C(55)-O(56)	120.1(4)	C(70)-O(73)-C(74)	118.1(4)
C(30')-O(33')-C(34')	116(3)	O(55)-C(55)-C(44)	123.5(3)	O(75)-C(75)-O(76)	121.6(4)
O(35)-C(35)-O(36)	120.5(3)	O(56)-C(55)-C(44)	116.4(4)	O(75)-C(75)-C(64)	124.6(3)
O(35)-C(35)-C(24)	122.7(3)	C(55)-O(55)-Zr(1)	133.5(2)	O(76)-C(75)-C(64)	113.8(3)
O(36)-C(35)-C(24)	116.9(3)	C(55)-O(56)-C(57')	125.8(6)	C(75)-O(75)-Zr(1)	132.1(2)
C(35)-O(35)-Zr(1)	134.3(2)	C(55)-O(56)-C(57)	109.4(4)	C(75)-O(76)-C(77)	117.4(3)
C(35)-O(36)-C(37)	115.9(3)	C(58)-C(57)-O(56)	104.8(7)	O(76)-C(77)-C(78)	111.9(4)
O(36)-C(37)-C(38)	107.5(3)				

Table S17. Bond lengths [Å] and angles [°] for **34**.

Zr(1)-O(3)	*2.0878(13)*	C(26)-C(31)	1.387(3)	O(12)-Zr(1)-O(32)#1	134.79(5)	C(12)-O(13)-C(14)	115.99(17)
Zr(1)-O(3)#1	*2.0879(13)*	C(27)-C(28)	1.380(3)	O(12)#1-Zr(1)-O(32)#1	128.10(5)	O(13)-C(14)-C(15)	107.8(2)
Zr(1)-O(23)#1	*2.0941(13)*	C(28)-C(29)	1.376(4)	O(3)-Zr(1)-O(32)	71.65(5)	C(25)-N(21)-N(22)	104.85(17)
Zr(1)-O(23)	*2.0941(13)*	C(29)-C(30)	1.381(3)	O(3)#1-Zr(1)-O(32)	141.81(5)	C(23)-N(22)-N(21)	111.01(17)
Zr(1)-O(12)	*2.2982(13)*	C(30)-C(31)	1.378(3)	O(23)#1-Zr(1)-O(32)	76.62(5)	C(23)-N(22)-C(26)	128.81(17)
Zr(1)-O(12)#1	*2.2983(13)*	*C(32)-O(32)*	*1.242(2)*	*O(23)-Zr(1)-O(32)*	*77.52(5)*	N(21)-N(22)-C(26)	120.15(16)
Zr(1)-O(32)#1	*2.3135(13)*	C(32)-O(33)	1.321(2)	O(12)-Zr(1)-O(32)	128.10(5)	O(23)-C(23)-N(22)	123.53(18)
Zr(1)-O(32)	*2.3135(13)*	O(33)-C(34)	1.458(3)	O(12)#1-Zr(1)-O(32)	134.79(5)	O(23)-C(23)-C(24)	129.64(18)
N(1)-C(5)	1.311(3)	C(34)-C(35)	1.491(4)	O(32)#1-Zr(1)-O(32)	71.06(7)	N(22)-C(23)-C(24)	106.76(17)
N(1)-N(2)	1.400(3)	C(40)-Cl(41)	1.703(8)	C(5)-N(1)-N(2)	105.01(18)	C(23)-O(23)-Zr(1)	133.59(13)
N(2)-C(3)	1.361(3)	C(40)-Cl(42)	1.723(9)	C(3)-N(2)-N(1)	111.00(18)	C(23)-C(24)-C(25)	104.57(18)
N(2)-C(6)	1.421(3)	C(40)-Cl(43)	1.754(8)	C(3)-N(2)-C(6)	129.29(18)	C(23)-C(24)-C(32)	121.14(18)
C(3)-O(3)	*1.289(2)*	C(50)-Cl(53)	1.708(17)	N(1)-N(2)-C(6)	119.71(18)	C(25)-C(24)-C(32)	134.27(19)
C(3)-C(4)	1.397(3)	C(50)-Cl(51)	1.709(17)	O(3)-C(3)-N(2)	123.63(19)	N(21)-C(25)-C(24)	112.80(19)
C(4)-C(5)	1.413(3)	C(50)-Cl(52)	1.713(16)	O(3)-C(3)-C(4)	129.6(2)	C(27)-C(26)-C(31)	120.2(2)
C(4)-C(12)	1.414(3)			N(2)-C(3)-C(4)	106.72(18)	C(27)-C(26)-N(22)	119.72(19)
C(6)-C(11)	1.378(3)	O(3)-Zr(1)-O(3)#1	146.35(8)	C(3)-O(3)-Zr(1)	131.13(13)	C(31)-C(26)-N(22)	120.00(18)
C(6)-C(7)	1.388(3)	O(3)-Zr(1)-O(23)#1	86.26(5)	C(3)-C(4)-C(5)	104.9(2)	C(28)-C(27)-C(26)	119.4(2)
C(7)-C(8)	1.379(4)	*O(3)#1-Zr(1)-O(23)#1*	*102.97(5)*	C(3)-C(4)-C(12)	120.13(19)	C(29)-C(28)-C(27)	120.8(2)
C(8)-C(9)	1.373(4)	*O(3)-Zr(1)-O(23)*	*102.97(5)*	C(5)-C(4)-C(12)	134.9(2)	C(28)-C(29)-C(30)	119.4(2)
C(9)-C(10)	1.381(4)	O(3)#1-Zr(1)-O(23)	86.26(5)	N(1)-C(5)-C(4)	112.4(2)	C(31)-C(30)-C(29)	120.7(2)
C(10)-C(11)	1.384(3)	O(23)#1-Zr(1)-O(23)	148.08(7)	C(11)-C(6)-C(7)	120.1(2)	C(30)-C(31)-C(26)	119.4(2)
C(12)-O(12)	*1.240(2)*	*O(3)-Zr(1)-O(12)*	*76.90(5)*	C(11)-C(6)-N(2)	121.2(2)	O(32)-C(32)-O(33)	121.98(19)
C(12)-O(13)	1.335(2)	O(3)#1-Zr(1)-O(12)	75.91(5)	C(7)-C(6)-N(2)	118.7(2)	O(32)-C(32)-C(24)	123.12(18)
O(13)-C(14)	1.463(3)	O(23)#1-Zr(1)-O(12)	141.15(5)	C(8)-C(7)-C(6)	119.2(3)	O(33)-C(32)-C(24)	114.90(17)
C(14)-C(15)	1.496(3)	O(23)-Zr(1)-O(12)	70.60(5)	C(9)-C(8)-C(7)	121.4(3)	C(32)-O(32)-Zr(1)	133.99(13)
N(21)-C(25)	1.310(3)	O(3)-Zr(1)-O(12)#1	75.91(5)	C(8)-C(9)-C(10)	118.9(3)	C(32)-O(33)-C(34)	117.91(17)
N(21)-N(22)	1.397(2)	*O(3)#1-Zr(1)-O(12)#1*	*76.90(5)*	C(9)-C(10)-C(11)	120.8(3)	O(33)-C(34)-C(35)	109.8(2)
N(22)-C(23)	1.361(2)	O(23)#1-Zr(1)-O(12)#1	70.60(5)	C(6)-C(11)-C(10)	119.6(2)	Cl(41)-C(40)-Cl(42)	111.8(4)
N(22)-C(26)	1.422(3)	O(23)-Zr(1)-O(12)#1	141.15(5)	O(12)-C(12)-O(13)	119.8(2)	Cl(41)-C(40)-Cl(43)	111.6(5)
C(23)-O(23)	*1.282(2)*	O(12)-Zr(1)-O(12)#1	71.42(7)	O(12)-C(12)-C(4)	123.23(19)	Cl(42)-C(40)-Cl(43)	110.9(5)
C(23)-C(24)	1.398(3)	O(3)-Zr(1)-O(32)#1	141.82(5)	O(13)-C(12)-C(4)	116.98(18)	Cl(53)-C(50)-Cl(51)	110.7(15)
C(24)-C(25)	1.409(3)	O(3)#1-Zr(1)-O(32)#1	71.65(5)	O(12)-C(12)-C(4)	123.23(19)	Cl(53)-C(50)-Cl(52)	112.8(16)
C(24)-C(32)	1.420(3)	*O(23)#1-Zr(1)-O(32)#1*	*77.52(5)*	O(13)-C(12)-C(4)	116.98(18)	Cl(51)-C(50)-Cl(52)	111.2(15)
C(26)-C(27)	1.385(3)	O(23)-Zr(1)-O(32)#1	76.62(5)	C(12)-O(12)-Zr(1)	133.11(14)		

Table S18. Bond lengths [Å] and angles [°] for **37**.

Zr(1)-O(3)#1	2.1136(12)	C(21)-C(22)	1.42(2)	C(23)-Zr(1)-C(25)	53.61(15)	C(8)-C(7)-C(6)	121.51(18)
Zr(1)-O(3)	2.1136(12)	C(22)-C(23)	1.408(18)	O(3)#1-Zr(1)-Cl(1)	97.76(13)	C(12)-C(7)-C(6)	120.59(17)
Zr(1)-O(15)#1	2.2323(12)	C(23)-C(24)	1.387(6)	O(3)-Zr(1)-Cl(1)	94.53(14)	C(7)-C(8)-C(9)	121.69(19)
Zr(1)-O(15)	2.2323(12)	C(24)-C(25)	1.407(7)	O(15)#1-Zr(1)-Cl(1)	75.77(11)	C(10)-C(9)-C(8)	119.23(18)
Zr(1)-C(21)	2.469(4)			O(15)-Zr(1)-Cl(1)	153.44(11)	O(13)-C(10)-C(9)	124.86(17)
Zr(1)-C(22)	2.478(18)	O(3)#1-Zr(1)-O(3)	150.23(7)	C(21)-Zr(1)-Cl(1)	115.38(17)	O(13)-C(10)-C(11)	115.45(17)
Zr(1)-C(23)	2.495(4)	O(3)#1-Zr(1)-O(15)#1	80.25(4)	C(22)-Zr(1)-Cl(1)	130.6(4)	C(9)-C(10)-C(11)	119.69(18)
Zr(1)-C(25)	2.497(4)	O(3)-Zr(1)-O(15)#1	76.65(5)	C(23)-Zr(1)-Cl(1)	102.82(15)	C(12)-C(11)-C(10)	120.26(18)
Zr(1)-Cl(1)	2.514(4)	O(3)#1-Zr(1)-O(15)	76.65(5)	C(25)-Zr(1)-Cl(1)	83.09(16)	C(11)-C(12)-C(7)	121.20(17)
Zr(1)-C(24)	2.530(4)	O(3)-Zr(1)-O(15)	80.25(4)	O(3)#1-Zr(1)-C(24)	93.14(13)	C(10)-O(13)-C(14)	118.15(16)
N(1)-C(5)	1.312(2)	O(15)#1-Zr(1)-O(15)	77.69(6)	O(3)-Zr(1)-C(24)	116.16(13)	O(15)-C(15)-O(16)	121.21(15)
N(1)-N(2)	1.382(2)	O(3)#1-Zr(1)-C(21)	120.67(13)	O(15)#1-Zr(1)-C(24)	149.92(12)	O(15)-C(15)-C(4)	122.46(16)
N(2)-C(3)	1.349(2)	O(3)-Zr(1)-C(21)	77.05(12)	O(15)-Zr(1)-C(24)	129.59(11)	O(16)-C(15)-C(4)	116.32(15)
N(2)-C(6)	1.461(2)	O(15)#1-Zr(1)-C(21)	152.16(11)	C(21)-Zr(1)-C(24)	53.99(17)	C(15)-O(15)-Zr(1)	131.71(11)
C(3)-O(3)	1.296(2)	O(15)-Zr(1)-C(21)	89.01(14)	C(22)-Zr(1)-C(24)	54.5(5)	C(15)-O(16)-C(17)	116.25(14)
C(3)-C(4)	1.402(2)	O(3)#1-Zr(1)-C(22)	87.7(5)	C(23)-Zr(1)-C(24)	32.03(15)	O(16)-C(17)-C(18)	106.85(16)
C(4)-C(5)	1.411(2)	O(3)-Zr(1)-C(22)	104.6(5)	C(25)-Zr(1)-C(24)	32.49(17)	C(25)-C(21)-C(22)	108.9(8)
C(4)-C(15)	1.420(2)	O(15)#1-Zr(1)-C(22)	152.6(5)	Cl(1)-Zr(1)-C(24)	76.13(15)	C(25)-C(21)-Zr(1)	74.7(2)
C(6)-C(7)	1.502(3)	O(15)-Zr(1)-C(22)	75.6(5)	C(5)-N(1)-N(2)	105.00(15)	C(22)-C(21)-Zr(1)	73.7(8)
C(7)-C(8)	1.381(3)	C(21)-Zr(1)-C(22)	33.3(5)	C(3)-N(2)-N(1)	112.23(15)	C(23)-C(22)-C(21)	105.6(14)
C(7)-C(12)	1.389(3)	O(3)#1-Zr(1)-C(23)	72.24(10)	C(3)-N(2)-C(6)	127.63(16)	C(23)-C(22)-Zr(1)	74.2(7)
C(8)-C(9)	1.390(3)	O(3)-Zr(1)-C(23)	130.87(10)	N(1)-N(2)-C(6)	120.01(15)	C(21)-C(22)-Zr(1)	73.0(8)
C(9)-C(10)	1.381(3)	O(15)#1-Zr(1)-C(23)	152.06(10)	O(3)-C(3)-N(2)	123.40(16)	C(24)-C(23)-C(22)	110.3(11)
C(10)-O(13)	1.365(2)	O(15)-Zr(1)-C(23)	100.11(11)	O(3)-C(3)-C(4)	130.76(15)	C(24)-C(23)-Zr(1)	75.4(2)
C(10)-C(11)	1.387(3)	C(21)-Zr(1)-C(23)	53.93(14)	N(2)-C(3)-C(4)	105.84(15)	C(22)-C(23)-Zr(1)	72.9(8)
C(11)-C(12)	1.372(3)	C(22)-Zr(1)-C(23)	32.9(4)	C(3)-O(3)-Zr(1)	125.21(10)	C(23)-C(24)-C(25)	107.4(4)
O(13)-C(14)	1.410(3)	O(3)#1-Zr(1)-C(25)	124.13(12)	C(3)-C(4)-C(5)	105.03(15)	C(23)-C(24)-Zr(1)	72.6(2)
C(15)-O(15)	1.246(2)	O(3)-Zr(1)-C(25)	84.16(13)	C(3)-C(4)-C(15)	121.75(16)	C(25)-C(24)-Zr(1)	72.5(2)
C(15)-O(16)	1.320(2)	O(15)#1-Zr(1)-C(25)	150.00(11)	C(5)-C(4)-C(15)	133.20(17)	C(21)-C(25)-C(24)	107.9(4)
O(16)-C(17)	1.463(2)	O(15)-Zr(1)-C(25)	121.78(14)	N(1)-C(5)-C(4)	111.89(17)	C(21)-C(25)-Zr(1)	72.5(2)
C(17)-C(18)	1.495(3)	C(21)-Zr(1)-C(25)	32.77(18)	N(2)-C(6)-C(7)	112.96(15)	C(24)-C(25)-Zr(1)	75.0(2)
C(21)-C(25)	1.401(8)	C(22)-Zr(1)-C(25)	54.9(6)	C(8)-C(7)-C(12)	117.89(18)		

Table S19. Bond lengths [Å] and angles [°] for **38**.

Zr(1)-O(50)	*1.9406(3)*	*C(32)-O(32)*	*1.247(4)*	O(12)-Zr(1)-C(42)	151.10(10)	C(23)-N(22)-N(21)	112.0(3)
Zr(1)-O(3)	*2.137(2)*	C(32)-O(33)	1.332(4)	O(32)-Zr(1)-C(42)	104.10(11)	C(23)-N(22)-C(26)	128.6(3)
Zr(1)-O(23)	*2.139(2)*	O(33)-C(34)	1.459(4)	C(45)-Zr(1)-C(42)	52.99(12)	N(21)-N(22)-C(26)	119.2(3)
Zr(1)-O(12)	*2.280(2)*	C(34)-C(35)	1.481(6)	C(44)-Zr(1)-C(42)	53.16(12)	O(23)-C(23)-N(22)	123.3(3)
Zr(1)-O(32)	*2.293(2)*	*C(41)-C(42)*	*1.387(5)*	C(43)-Zr(1)-C(42)	32.21(12)	O(23)-C(23)-C(24)	130.7(3)
Zr(1)-C(45)	*2.526(3)*	*C(41)-C(45)*	*1.399(5)*	O(50)-Zr(1)-C(41)	76.54(11)	N(22)-C(23)-C(24)	105.9(3)
Zr(1)-C(44)	*2.526(3)*	*C(42)-C(43)*	*1.415(5)*	O(3)-Zr(1)-C(41)	107.78(10)	C(23)-O(23)-Zr(1)	125.90(18)
Zr(1)-C(43)	*2.549(3)*	*C(43)-C(44)*	*1.395(5)*	O(23)-Zr(1)-C(41)	102.99(10)	C(23)-C(24)-C(25)	105.3(3)
Zr(1)-C(42)	*2.552(3)*	*C(44)-C(45)*	*1.400(5)*	O(12)-Zr(1)-C(41)	156.19(10)	C(23)-C(24)-C(32)	121.7(3)
Zr(1)-C(41)	*2.562(3)*			O(32)-Zr(1)-C(41)	126.84(10)	C(25)-C(24)-C(32)	132.9(3)
N(1)-C(5)	1.305(4)	O(50)-Zr(1)-O(3)	96.15(9)	C(45)-Zr(1)-C(41)	31.92(11)	N(21)-C(25)-C(24)	112.2(3)
N(1)-N(2)	1.396(4)	O(50)-Zr(1)-O(23)	96.50(6)	C(44)-Zr(1)-C(41)	52.74(11)	C(27)-C(26)-C(31)	120.0(3)
N(2)-C(3)	1.371(4)	O(3)-Zr(1)-O(23)	148.68(8)	C(43)-Zr(1)-C(41)	52.46(12)	C(27)-C(26)-N(22)	119.5(3)
N(2)-C(6)	1.415(4)	O(50)-Zr(1)-O(12)	80.23(9)	C(42)-Zr(1)-C(41)	31.46(12)	C(31)-C(26)-N(22)	120.5(3)
C(3)-O(3)	*1.277(3)*	*O(3)-Zr(1)-O(12)*	*79.72(7)*	C(5)-N(1)-N(2)	104.6(3)	C(26)-C(27)-C(28)	119.8(4)
C(3)-C(4)	1.400(4)	O(23)-Zr(1)-O(12)	74.39(8)	C(3)-N(2)-N(1)	111.6(2)	C(29)-C(28)-C(27)	120.5(4)
C(4)-C(12)	1.414(4)	O(50)-Zr(1)-O(32)	156.62(9)	C(3)-N(2)-C(6)	128.8(2)	C(30)-C(29)-C(28)	119.4(4)
C(4)-C(5)	1.414(4)	O(3)-Zr(1)-O(32)	77.75(8)	N(1)-N(2)-C(6)	119.5(2)	C(29)-C(30)-C(31)	121.4(4)
C(6)-C(7)	1.385(5)	*O(23)-Zr(1)-O(32)*	*79.31(8)*	O(3)-C(3)-N(2)	122.9(3)	C(30)-C(31)-C(26)	119.0(4)
C(6)-C(11)	1.386(5)	O(12)-Zr(1)-O(32)	76.48(8)	O(3)-C(3)-C(4)	131.2(3)	O(32)-C(32)-O(33)	120.5(3)
C(7)-C(8)	1.394(5)	O(50)-Zr(1)-C(45)	90.65(9)	N(2)-C(3)-C(4)	105.9(2)	O(32)-C(32)-C(24)	123.4(3)
C(8)-C(9)	1.372(6)	O(3)-Zr(1)-C(45)	77.96(10)	C(3)-O(3)-Zr(1)	129.50(19)	O(33)-C(32)-C(24)	116.0(3)
C(9)-C(10)	1.373(5)	O(23)-Zr(1)-C(45)	130.33(10)	C(3)-C(4)-C(12)	122.2(3)	C(32)-O(32)-Zr(1)	130.7(2)
C(10)-C(11)	1.392(5)	O(12)-Zr(1)-C(45)	154.80(10)	C(3)-C(4)-C(5)	104.8(3)	C(32)-O(33)-C(34)	117.6(3)
C(12)-O(12)	*1.240(4)*	O(32)-Zr(1)-C(45)	109.72(10)	C(12)-C(4)-C(5)	133.0(3)	O(33)-C(34)-C(35)	112.9(3)
C(12)-O(13)	1.327(4)	O(50)-Zr(1)-C(44)	122.75(9)	N(1)-C(5)-C(4)	113.1(3)	C(42)-C(41)-C(45)	108.8(3)
O(13)-C(14)	1.462(4)	O(3)-Zr(1)-C(44)	78.49(10)	C(7)-C(6)-C(11)	119.9(3)	C(42)-C(41)-Zr(1)	73.88(19)
C(14)-C(15)	1.502(5)	O(23)-Zr(1)-C(44)	117.29(10)	C(7)-C(6)-N(2)	118.9(3)	C(45)-C(41)-Zr(1)	72.60(18)
N(21)-C(25)	1.317(4)	O(12)-Zr(1)-C(44)	149.75(9)	C(11)-C(6)-N(2)	121.2(3)	C(41)-C(42)-C(43)	107.4(3)
N(21)-N(22)	1.398(4)	O(32)-Zr(1)-C(44)	78.58(10)	C(6)-C(7)-C(8)	119.4(4)	C(41)-C(42)-Zr(1)	74.66(19)
N(22)-C(23)	1.357(4)	C(45)-Zr(1)-C(44)	32.18(11)	C(9)-C(8)-C(7)	121.1(4)	C(43)-C(42)-Zr(1)	73.76(18)
N(22)-C(26)	1.426(4)	O(50)-Zr(1)-C(43)	127.72(12)	C(8)-C(9)-C(10)	119.0(3)	C(44)-C(43)-C(42)	107.9(3)
C(23)-O(23)	*1.291(3)*	O(3)-Zr(1)-C(43)	108.50(10)	C(9)-C(10)-C(11)	121.3(4)	C(44)-C(43)-Zr(1)	73.13(19)
C(23)-C(24)	1.400(4)	O(23)-Zr(1)-C(43)	85.70(10)	C(6)-C(11)-C(10)	119.3(3)	C(42)-C(43)-Zr(1)	74.0(2)
C(24)-C(25)	1.412(4)	O(12)-Zr(1)-C(43)	147.90(10)	O(12)-C(12)-O(13)	120.7(3)	C(43)-C(44)-C(45)	108.1(3)
C(24)-C(32)	1.425(5)	O(32)-Zr(1)-C(43)	75.23(10)	O(12)-C(12)-C(4)	123.4(3)	C(43)-C(44)-Zr(1)	74.96(19)
C(26)-C(27)	1.381(5)	C(45)-Zr(1)-C(43)	52.96(11)	O(13)-C(12)-C(4)	115.9(3)	C(45)-C(44)-Zr(1)	73.90(19)
C(26)-C(31)	1.392(5)	C(44)-Zr(1)-C(43)	31.91(12)	C(12)-O(12)-Zr(1)	133.3(2)	C(41)-C(45)-C(44)	107.7(3)
C(27)-C(28)	1.386(6)	O(50)-Zr(1)-C(42)	97.26(13)	C(12)-O(13)-C(14)	116.7(3)	C(41)-C(45)-Zr(1)	75.48(19)
C(28)-C(29)	1.376(7)	O(3)-Zr(1)-C(42)	129.02(10)	O(13)-C(14)-C(15)	107.2(3)	C(44)-C(45)-Zr(1)	73.92(18)
C(29)-C(30)	1.369(6)	O(23)-Zr(1)-C(42)	77.34(10)	C(25)-N(21)-N(22)	104.5(3)	Zr(1)#1-O(50)-Zr(1)	173.92(17)
C(30)-C(31)	1.382(5)						

Table S20. Bond lengths [Å] and angles [°] for **39**.

Rh(1)-O(20)	*2.031(2)*	C(21)-C(23)	1.501(5)	C(5)-C(4)-C(15)	134.9(4)
Rh(1)-O(32)#1	*2.034(2)*	*O(30)-C(31)*	*1.263(4)*	N(1)-C(5)-C(4)	111.1(3)
Rh(1)-O(22)#1	*2.039(2)*	*C(31)-O(32)*	*1.264(4)*	N(2)-C(6)-C(7)	112.7(3)
Rh(1)-O(30)	*2.049(2)*	C(31)-C(33)	1.510(5)	C(12)-C(7)-C(8)	118.4(3)
Rh(1)-N(1)	*2.278(3)*			C(12)-C(7)-C(6)	120.3(3)
Rh(1)-Rh(1)#1	*2.3956(5)*	*O(20)-Rh(1)-O(32)#1*	*91.35(10)*	C(8)-C(7)-C(6)	121.2(3)
N(1)-C(5)	1.332(4)	*O(20)-Rh(1)-O(22)#1*	*176.09(10)*	C(9)-C(8)-C(7)	121.0(3)
N(1)-N(2)	1.357(4)	*O(32)#1-Rh(1)-O(22)#1*	*89.07(10)*	C(8)-C(9)-C(10)	119.6(4)
N(2)-C(3)	1.329(5)	*O(20)-Rh(1)-O(30)*	*88.69(10)*	C(11)-C(10)-O(13)	124.4(4)
N(2)-C(6)	1.455(5)	O(32)#1-Rh(1)-O(30)	175.84(9)	C(11)-C(10)-C(9)	120.5(4)
C(3)-O(3)	*1.349(4)*	*O(22)#1-Rh(1)-O(30)*	*90.61(10)*	O(13)-C(10)-C(9)	115.1(4)
C(3)-C(4)	1.380(6)	*O(20)-Rh(1)-N(1)*	*93.53(10)*	C(10)-C(11)-C(12)	118.6(4)
C(4)-C(5)	1.397(5)	O(32)#1-Rh(1)-N(1)	86.38(10)	C(7)-C(12)-C(11)	121.9(4)
C(4)-C(15)	1.447(5)	O(22)#1-Rh(1)-N(1)	90.38(10)	C(10)-O(13)-C(14)	117.9(4)
C(6)-C(7)	1.511(5)	O(30)-Rh(1)-N(1)	97.77(10)	O(15)-C(15)-O(16)	124.1(4)
C(7)-C(12)	1.375(5)	O(20)-Rh(1)-Rh(1)#1	87.76(7)	O(15)-C(15)-C(4)	121.9(4)
C(7)-C(8)	1.386(5)	O(32)#1-Rh(1)-Rh(1)#1	88.70(7)	O(16)-C(15)-C(4)	114.0(4)
C(8)-C(9)	1.369(5)	O(22)#1-Rh(1)-Rh(1)#1	88.36(7)	C(15)-O(16)-C(17)	120.4(4)
C(9)-C(10)	1.397(5)	O(30)-Rh(1)-Rh(1)#1	87.14(6)	C(15)-O(16)-C(17')	104.3(6)
C(10)-C(11)	1.370(6)	N(1)-Rh(1)-Rh(1)#1	174.94(8)	O(16)-C(17)-C(18)	105.2(5)
C(10)-O(13)	1.375(4)	C(5)-N(1)-N(2)	106.1(3)	C(18')-C(17')-O(16)	103.0(11)
C(11)-C(12)	1.392(5)	C(5)-N(1)-Rh(1)	120.2(2)	C(21)-O(20)-Rh(1)	119.6(2)
O(13)-C(14)	1.421(5)	N(2)-N(1)-Rh(1)	132.9(2)	O(22)-C(21)-O(20)	125.7(3)
C(15)-O(15)	*1.224(5)*	C(3)-N(2)-N(1)	109.9(3)	O(22)-C(21)-C(23)	117.8(3)
C(15)-O(16)	1.332(5)	C(3)-N(2)-C(6)	128.1(3)	O(20)-C(21)-C(23)	116.5(3)
O(16)-C(17)	1.475(5)	N(1)-N(2)-C(6)	121.8(3)	C(21)-O(22)-Rh(1)#1	118.5(2)
O(16)-C(17')	1.545(14)	N(2)-C(3)-O(3)	120.2(4)	C(31)-O(30)-Rh(1)	119.7(2)
C(17)-C(18)	1.497(9)	N(2)-C(3)-C(4)	109.4(3)	O(30)-C(31)-O(32)	125.6(3)
C(17')-C(18')	1.477(16)	O(3)-C(3)-C(4)	130.4(3)	O(30)-C(31)-C(33)	117.3(3)
O(20)-C(21)	*1.269(4)*	C(3)-C(4)-C(5)	103.4(3)	O(32)-C(31)-C(33)	117.1(3)
C(21)-O(22)	*1.269(4)*	C(3)-C(4)-C(15)	121.4(4)	C(31)-O(32)-Rh(1)#1	118.8(2)

Table S21. Bond lengths [Å] and angles [°] for **40**.

Rh(1)-O(32)#1	*2.033(2)*	*C(31)-O(32)*	*1.263(4)*	C(3)-C(4)-C(5)	104.0(3)
Rh(1)-O(20)	*2.037(2)*	C(31)-C(33)	1.506(5)	C(3)-C(4)-C(12)	124.1(3)
Rh(1)-O(22)#1	*2.039(2)*			C(5)-C(4)-C(12)	131.9(3)
Rh(1)-O(30)	*2.045(2)*	*O(32)#1-Rh(1)-O(20)*	*91.21(10)*	N(1)-C(5)-C(4)	111.5(3)
Rh(1)-N(1)	*2.276(2)*	*O(32)#1-Rh(1)-O(22)#1*	*89.79(10)*	C(7)-C(6)-C(11)	121.9(3)
Rh(1)-Rh(1)#1	*2.4015(5)*	*O(20)-Rh(1)-O(22)#1*	*175.70(9)*	C(7)-C(6)-N(2)	119.0(3)
N(1)-C(5)	1.319(4)	O(32)#1-Rh(1)-O(30)	175.58(9)	C(11)-C(6)-N(2)	119.2(3)
N(1)-N(2)	1.369(3)	*O(20)-Rh(1)-O(30)*	*87.09(9)*	C(6)-C(7)-C(8)	118.6(4)
N(2)-C(3)	1.352(4)	*O(22)#1-Rh(1)-O(30)*	*91.61(10)*	C(9)-C(8)-C(7)	120.3(4)
N(2)-C(6)	1.438(4)	O(32)#1-Rh(1)-N(1)	94.13(9)	C(8)-C(9)-C(10)	120.5(4)
C(3)-O(3)	*1.328(4)*	*O(20)-Rh(1)-N(1)*	*90.90(9)*	C(9)-C(10)-C(11)	119.9(4)
C(3)-C(4)	1.389(5)	O(22)#1-Rh(1)-N(1)	93.20(9)	C(6)-C(11)-C(10)	118.8(4)
C(4)-C(5)	1.413(4)	O(30)-Rh(1)-N(1)	89.98(9)	O(12)-C(12)-O(13)	124.2(3)
C(4)-C(12)	1.437(4)	O(32)#1-Rh(1)-Rh(1)#1	87.34(6)	O(12)-C(12)-C(4)	122.9(3)
C(6)-C(7)	1.378(5)	O(20)-Rh(1)-Rh(1)#1	88.13(6)	O(13)-C(12)-C(4)	112.9(3)
C(6)-C(11)	1.381(5)	O(22)#1-Rh(1)-Rh(1)#1	87.74(6)	C(12)-O(13)-C(14)	117.6(3)
C(7)-C(8)	1.390(5)	O(30)-Rh(1)-Rh(1)#1	88.52(6)	O(13)-C(14)-C(15)	106.5(3)
C(8)-C(9)	1.375(6)	N(1)-Rh(1)-Rh(1)#1	178.26(7)	C(21)-O(20)-Rh(1)	118.7(2)
C(9)-C(10)	1.382(6)	C(5)-N(1)-N(2)	106.2(2)	O(22)-C(21)-O(20)	126.3(3)
C(10)-C(11)	1.387(5)	C(5)-N(1)-Rh(1)	122.5(2)	O(22)-C(21)-C(23)	116.9(3)
C(12)-O(12)	*1.220(4)*	N(2)-N(1)-Rh(1)	130.26(19)	O(20)-C(21)-C(23)	116.8(3)
C(12)-O(13)	1.329(4)	C(3)-N(2)-N(1)	110.4(3)	C(21)-O(22)-Rh(1)#1	119.1(2)
O(13)-C(14)	1.454(4)	C(3)-N(2)-C(6)	127.5(3)	C(31)-O(30)-Rh(1)	118.0(2)
C(14)-C(15)	1.486(5)	N(1)-N(2)-C(6)	121.9(3)	O(32)-C(31)-O(30)	126.2(3)
O(20)-C(21)	*1.266(4)*	O(3)-C(3)-N(2)	119.6(3)	O(32)-C(31)-C(33)	116.3(3)
C(21)-O(22)	*1.262(4)*	O(3)-C(3)-C(4)	132.4(3)	O(30)-C(31)-C(33)	117.5(3)
C(21)-C(23)	1.504(5)	N(2)-C(3)-C(4)	107.9(3)	C(31)-O(32)-Rh(1)#1	119.8(2)
O(30)-C(31)	*1.266(4)*				

Table S22. Bond lengths [Å] and angles [°] for **42**.

Mn(1)-O(3)	*2.1013(19)*	C(34)-C(35)	1.501(4)	C(9)-C(10)-C(11)	120.9(4)
Mn(1)-O(23)	*2.1029(18)*	O(40)-C(41)	1.412(5)	C(6)-C(11)-C(10)	119.5(3)
Mn(1)-O(40)	*2.196(2)*	O(40)-C(41')	1.457(10)	O(12)-C(12)-O(13)	120.8(3)
Mn(1)-O(50)	*2.201(2)*	C(41)-C(42)	1.482(8)	O(12)-C(12)-C(4)	125.5(3)
Mn(1)-O(12)	*2.210(2)*	C(41')-C(42')	1.508(13)	O(13)-C(12)-C(4)	113.7(3)
Mn(1)-O(32)	*2.220(2)*	O(50)-C(51)	1.415(4)	C(12)-O(12)-Mn(1)	126.5(2)
N(1)-C(5)	1.312(4)	C(51)-C(52)	1.471(5)	C(12)-O(13)-C(14)	116.5(2)
N(1)-N(2)	1.394(3)			O(13)-C(14)-C(15)	110.9(3)
N(2)-C(3)	1.379(4)	O(3)-Mn(1)-O(23)	175.92(8)	C(25)-N(21)-N(22)	105.2(2)
N(2)-C(6)	1.420(4)	O(3)-Mn(1)-O(40)	86.70(8)	C(23)-N(22)-N(21)	111.0(2)
C(3)-O(3)	*1.268(3)*	O(23)-Mn(1)-O(40)	89.62(8)	C(23)-N(22)-C(26)	129.6(2)
C(3)-C(4)	1.403(4)	O(3)-Mn(1)-O(50)	87.98(8)	N(21)-N(22)-C(26)	119.2(2)
C(4)-C(5)	1.409(4)	O(23)-Mn(1)-O(50)	90.46(7)	O(23)-C(23)-N(22)	124.3(3)
C(4)-C(12)	1.433(4)	*O(40)-Mn(1)-O(50)*	*93.25(8)*	O(23)-C(23)-C(24)	129.8(3)
C(6)-C(11)	1.382(5)	*O(3)-Mn(1)-O(12)*	*86.94(8)*	N(22)-C(23)-C(24)	105.9(2)
C(6)-C(7)	1.389(4)	O(23)-Mn(1)-O(12)	96.87(8)	C(23)-O(23)-Mn(1)	125.54(18)
C(7)-C(8)	1.379(5)	O(40)-Mn(1)-O(12)	172.17(8)	C(23)-C(24)-C(25)	105.0(2)
C(8)-C(9)	1.372(5)	O(50)-Mn(1)-O(12)	91.08(8)	C(23)-C(24)-C(32)	126.1(3)
C(9)-C(10)	1.377(5)	*O(3)-Mn(1)-O(32)*	*95.23(8)*	C(25)-C(24)-C(32)	128.8(3)
C(10)-C(11)	1.390(5)	*O(23)-Mn(1)-O(32)*	*86.58(8)*	N(21)-C(25)-C(24)	112.9(3)
C(12)-O(12)	*1.238(3)*	O(40)-Mn(1)-O(32)	90.82(8)	C(31)-C(26)-C(27)	119.3(3)
C(12)-O(13)	1.344(4)	O(50)-Mn(1)-O(32)	174.95(8)	C(31)-C(26)-N(22)	121.2(3)
O(13)-C(14)	1.456(3)	O(12)-Mn(1)-O(32)	85.23(7)	C(27)-C(26)-N(22)	119.5(3)
C(14)-C(15)	1.509(5)	C(5)-N(1)-N(2)	105.1(2)	C(28)-C(27)-C(26)	119.5(3)
N(21)-C(25)	1.309(4)	C(3)-N(2)-N(1)	111.6(2)	C(29)-C(28)-C(27)	121.1(3)
N(21)-N(22)	1.395(3)	C(3)-N(2)-C(6)	129.0(3)	C(28)-C(29)-C(30)	118.9(3)
N(22)-C(23)	1.382(3)	N(1)-N(2)-C(6)	119.1(2)	C(29)-C(30)-C(31)	121.0(3)
N(22)-C(26)	1.423(4)	O(3)-C(3)-N(2)	124.0(3)	C(26)-C(31)-C(30)	120.1(3)
C(23)-O(23)	*1.265(3)*	O(3)-C(3)-C(4)	130.8(3)	O(32)-C(32)-O(33)	121.9(3)
C(23)-C(24)	1.405(4)	N(2)-C(3)-C(4)	105.2(2)	O(32)-C(32)-C(24)	124.3(3)
C(24)-C(25)	1.412(4)	C(3)-O(3)-Mn(1)	124.75(19)	O(33)-C(32)-C(24)	113.8(3)
C(24)-C(32)	1.424(4)	C(3)-C(4)-C(5)	105.8(2)	C(32)-O(32)-Mn(1)	127.18(18)
C(26)-C(31)	1.370(4)	C(3)-C(4)-C(12)	124.5(3)	C(32)-O(33)-C(34)	117.3(2)
C(26)-C(27)	1.398(4)	C(5)-C(4)-C(12)	129.6(3)	O(33)-C(34)-C(35)	110.6(3)
C(27)-C(28)	1.382(4)	N(1)-C(5)-C(4)	112.3(3)	C(41)-O(40)-Mn(1)	124.8(2)
C(28)-C(29)	1.366(5)	C(11)-C(6)-C(7)	119.4(3)	C(41')-O(40)-Mn(1)	134.3(4)
C(29)-C(30)	1.377(4)	C(11)-C(6)-N(2)	121.0(3)	O(40)-C(41)-C(42)	112.7(5)
C(30)-C(31)	1.383(4)	C(7)-C(6)-N(2)	119.6(3)	O(40)-C(41')-C(42')	106.4(8)
C(32)-O(32)	*1.244(3)*	C(8)-C(7)-C(6)	120.2(4)	C(51)-O(50)-Mn(1)	131.87(19)
C(32)-O(33)	1.330(4)	C(9)-C(8)-C(7)	120.7(3)	O(50)-C(51)-C(52)	113.7(3)
O(33)-C(34)	1.451(3)	C(8)-C(9)-C(10)	119.2(4)		

Table S23. Bond lengths [Å] and angles [°] for **46**.

Fe(1)-O(23)	*1.9484(18)*	O(33)-C(34)	1.461(6)	C(3)-N(2)-C(6)	129.3(2)	C(26)-C(27)-C(28)	119.5(3)
Fe(1)-O(43)	*1.9548(16)*	O(33)-C(34')	1.518(11)	N(1)-N(2)-C(6)	119.3(2)	C(29)-C(28)-C(27)	119.9(3)
Fe(1)-O(3)	*1.9761(15)*	C(34)-C(35)	1.499(8)	O(3)-C(3)-N(2)	123.9(2)	C(28)-C(29)-C(30)	120.1(3)
Fe(1)-O(52)	*2.0500(15)*	C(34')-C(35')	1.536(12)	O(3)-C(3)-C(4)	129.8(2)	C(31)-C(30)-C(29)	120.7(3)
Fe(1)-O(32)	*2.0679(17)*	N(41)-C(45)	1.312(3)	N(2)-C(3)-C(4)	106.2(2)	C(30)-C(31)-C(26)	119.2(3)
Fe(1)-O(12)	*2.0815(18)*	N(41)-N(42)	1.393(3)	C(3)-O(3)-Fe(1)	122.00(16)	O(32)-C(32)-O(33)	120.6(3)
N(1)-C(5)	1.315(3)	N(42)-C(43)	1.365(3)	C(3)-C(4)-C(12)	122.7(2)	O(32)-C(32)-C(24)	123.3(3)
N(1)-N(2)	1.392(3)	N(42)-C(46)	1.422(3)	C(3)-C(4)-C(5)	105.1(2)	O(33)-C(32)-C(24)	116.1(3)
N(2)-C(3)	1.377(3)	*C(43)-O(43)*	*1.299(3)*	C(12)-C(4)-C(5)	132.1(3)	C(32)-O(32)-Fe(1)	126.92(19)
N(2)-C(6)	1.424(3)	C(43)-C(44)	1.395(3)	N(1)-C(5)-C(4)	111.8(2)	C(32)-O(33)-C(34)	118.0(4)
C(3)-O(3)	*1.286(3)*	C(44)-C(45)	1.415(3)	C(11)-C(6)-C(7)	120.1(3)	C(32)-O(33)-C(34')	115.0(6)
C(3)-C(4)	1.397(3)	C(44)-C(52)	1.417(3)	C(11)-C(6)-N(2)	121.6(2)	O(33)-C(34)-C(35)	105.4(6)
C(4)-C(12)	1.411(3)	C(46)-C(51)	1.379(3)	C(7)-C(6)-N(2)	118.4(3)	O(33)-C(34')-C(35')	110.2(9)
C(4)-C(5)	1.428(3)	C(46)-C(47)	1.386(4)	C(8)-C(7)-C(6)	119.1(3)	C(45)-N(41)-N(42)	104.74(19)
C(6)-C(11)	1.386(4)	C(47)-C(48)	1.382(4)	C(9)-C(8)-C(7)	121.3(3)	C(43)-N(42)-N(41)	111.79(18)
C(6)-C(7)	1.391(3)	C(48)-C(49)	1.368(4)	C(8)-C(9)-C(10)	119.5(3)	C(43)-N(42)-C(46)	129.6(2)
C(7)-C(8)	1.386(4)	C(49)-C(50)	1.376(5)	C(9)-C(10)-C(11)	120.3(3)	N(41)-N(42)-C(46)	118.5(2)
C(8)-C(9)	1.374(4)	C(50)-C(51)	1.399(4)	C(6)-C(11)-C(10)	119.7(3)	O(43)-C(43)-N(42)	123.7(2)
C(9)-C(10)	1.380(4)	*C(52)-O(52)*	*1.254(3)*	O(12)-C(12)-O(13)	120.6(2)	O(43)-C(43)-C(44)	130.4(2)
C(10)-C(11)	1.393(4)	C(52)-O(53)	1.329(3)	O(12)-C(12)-C(4)	123.9(3)	N(42)-C(43)-C(44)	105.9(2)
C(12)-O(12)	*1.254(3)*	O(53)-C(54)	1.459(3)	O(13)-C(12)-C(4)	115.5(2)	C(43)-O(43)-Fe(1)	123.47(14)
C(12)-O(13)	1.330(3)	C(54)-C(55)	1.489(4)	C(12)-O(12)-Fe(1)	126.21(17)	C(43)-C(44)-C(45)	105.3(2)
O(13)-C(14)	1.455(3)			C(12)-O(13)-C(14)	116.9(2)	C(43)-C(44)-C(52)	122.7(2)
C(14)-C(15)	1.498(3)	O(23)-Fe(1)-O(43)	98.31(7)	O(13)-C(14)-C(15)	110.1(2)	C(45)-C(44)-C(52)	132.0(2)
N(21)-C(25)	1.306(4)	O(23)-Fe(1)-O(3)	87.58(7)	C(25)-N(21)-N(22)	104.3(2)	N(41)-C(45)-C(44)	112.2(2)
N(21)-N(22)	1.399(3)	O(43)-Fe(1)-O(3)	96.39(7)	C(23)-N(22)-N(21)	111.9(2)	C(51)-C(46)-C(47)	120.4(2)
N(22)-C(23)	1.361(3)	O(23)-Fe(1)-O(52)	95.04(7)	C(23)-N(22)-C(26)	128.2(2)	C(51)-C(46)-N(42)	120.6(2)
N(22)-C(26)	1.422(3)	*O(43)-Fe(1)-O(52)*	*89.80(6)*	N(21)-N(22)-C(26)	119.5(2)	C(47)-C(46)-N(42)	119.0(2)
C(23)-O(23)	*1.300(3)*	O(3)-Fe(1)-O(52)	172.87(7)	O(23)-C(23)-N(22)	123.1(2)	C(48)-C(47)-C(46)	119.7(3)
C(23)-C(24)	1.411(4)	*O(23)-Fe(1)-O(32)*	*91.01(7)*	O(23)-C(23)-C(24)	130.8(3)	C(49)-C(48)-C(47)	120.4(3)
C(24)-C(32)	1.411(4)	O(43)-Fe(1)-O(32)	168.39(7)	N(22)-C(23)-C(24)	106.1(2)	C(48)-C(49)-C(50)	120.3(3)
C(24)-C(25)	1.414(4)	O(3)-Fe(1)-O(32)	90.87(7)	C(23)-O(23)-Fe(1)	120.83(17)	C(49)-C(50)-C(51)	120.1(3)
C(26)-C(31)	1.387(4)	O(52)-Fe(1)-O(32)	82.46(6)	C(32)-C(24)-C(23)	123.0(2)	C(46)-C(51)-C(50)	119.1(3)
C(26)-C(27)	1.387(4)	O(23)-Fe(1)-O(12)	171.35(7)	C(32)-C(24)-C(25)	132.8(3)	O(52)-C(52)-O(53)	120.8(2)
C(27)-C(28)	1.388(5)	O(43)-Fe(1)-O(12)	89.79(7)	C(23)-C(24)-C(25)	104.2(3)	O(52)-C(52)-C(44)	123.0(2)
C(28)-C(29)	1.380(5)	*O(3)-Fe(1)-O(12)*	*88.49(7)*	N(21)-C(25)-C(24)	113.4(3)	O(53)-C(52)-C(44)	116.2(2)
C(29)-C(30)	1.382(4)	O(52)-Fe(1)-O(12)	88.00(7)	C(31)-C(26)-C(27)	120.6(3)	C(52)-O(52)-Fe(1)	128.99(16)
C(30)-C(31)	1.377(4)	O(32)-Fe(1)-O(12)	81.35(7)	C(31)-C(26)-N(22)	120.4(3)	C(52)-O(53)-C(54)	117.28(18)
C(32)-O(32)	*1.252(3)*	C(5)-N(1)-N(2)	105.5(2)	C(27)-C(26)-N(22)	119.0(3)	O(53)-C(54)-C(55)	110.9(2)
C(32)-O(33)	1.330(3)	C(3)-N(2)-N(1)	111.3(2)				

Table S24. Bond lengths [Å] and angles [°] for **49**.

Zn(1)-O(3)#1	*2.0737(13)*	*O(3)#1-Zn(1)-N(1)#3*	*91.19(6)*
Zn(1)-O(3)	*2.0737(13)*	*O(3)-Zn(1)-N(1)#3*	*88.81(6)*
Zn(1)-O(15)	*2.1381(13)*	*O(15)-Zn(1)-N(1)#3*	*93.22(6)*
Zn(1)-O(15)#1	*2.1382(13)*	*O(15)#1-Zn(1)-N(1)#3*	*86.78(6)*
Zn(1)-N(1)#2	*2.1719(16)*	N(1)#2-Zn(1)-N(1)#3	180.0
Zn(1)-N(1)#3	*2.1719(16)*	C(5)-N(1)-N(2)	105.29(16)
N(1)-C(5)	1.316(3)	C(5)-N(1)-Zn(1)#4	121.05(13)
N(1)-N(2)	1.389(2)	N(2)-N(1)-Zn(1)#4	133.06(12)
N(2)-C(3)	1.372(2)	C(3)-N(2)-N(1)	112.05(15)
N(2)-C(6)	1.446(2)	C(3)-N(2)-C(6)	126.13(16)
C(3)-O(3)	*1.272(2)*	N(1)-N(2)-C(6)	121.81(15)
C(3)-C(4)	1.419(3)	O(3)-C(3)-N(2)	122.80(17)
C(4)-C(5)	1.403(3)	O(3)-C(3)-C(4)	132.47(18)
C(4)-C(15)	1.424(3)	N(2)-C(3)-C(4)	104.72(16)
C(6)-C(7)	1.515(3)	C(3)-O(3)-Zn(1)	118.39(12)
C(7)-C(8)	1.386(3)	C(5)-C(4)-C(3)	105.85(17)
C(7)-C(12)	1.394(3)	C(5)-C(4)-C(15)	128.47(19)
C(8)-C(9)	1.399(3)	C(3)-C(4)-C(15)	125.53(18)
C(9)-C(10)	1.390(3)	N(1)-C(5)-C(4)	112.08(18)
C(10)-O(13)	1.379(2)	N(2)-C(6)-C(7)	113.46(16)
C(10)-C(11)	1.385(3)	C(8)-C(7)-C(12)	118.25(19)
C(11)-C(12)	1.379(3)	C(8)-C(7)-C(6)	122.34(18)
O(13)-C(14)	1.421(3)	C(12)-C(7)-C(6)	119.41(19)
C(15)-O(15)	*1.235(2)*	C(7)-C(8)-C(9)	121.5(2)
C(15)-O(16)	1.338(2)	C(10)-C(9)-C(8)	118.7(2)
O(16)-C(17)	1.455(6)	O(13)-C(10)-C(11)	115.7(2)
O(16)-C(17')	1.473(9)	O(13)-C(10)-C(9)	123.8(2)
C(17)-C(18)	1.488(10)	C(11)-C(10)-C(9)	120.41(19)
C(17')-C(18')	1.495(10)	C(12)-C(11)-C(10)	119.9(2)
		C(11)-C(12)-C(7)	121.2(2)
O(3)#1-Zn(1)-O(3)	180.0	C(10)-O(13)-C(14)	117.01(19)
O(3)#1-Zn(1)-O(15)	*87.60(5)*	O(15)-C(15)-O(16)	121.09(18)
O(3)-Zn(1)-O(15)	*92.40(5)*	O(15)-C(15)-C(4)	125.43(18)
O(3)#1-Zn(1)-O(15)#1	*92.40(5)*	O(16)-C(15)-C(4)	113.46(17)
O(3)-Zn(1)-O(15)#1	*87.60(5)*	C(15)-O(15)-Zn(1)	123.70(13)
O(15)-Zn(1)-O(15)#1	180.0	C(15)-O(16)-C(17)	117.2(3)
O(3)#1-Zn(1)-N(1)#2	*88.81(6)*	C(15)-O(16)-C(17')	118.3(5)
O(3)-Zn(1)-N(1)#2	*91.19(6)*	O(16)-C(17)-C(18)	104.7(6)
O(15)-Zn(1)-N(1)#2	*86.78(6)*	O(16)-C(17')-C(18')	105.0(7)
O(15)#1-Zn(1)-N(1)#2	*93.22(6)*		

Table S25. Bond lengths [Å] and angles [°] for **50**.

Zn(1)-O(23)	*1.969(3)*	C(34)-C(35)	1.500(8)	C(9)-C(10)-C(11)	120.9(7)
Zn(1)-O(3)	*1.992(3)*	O(40)-C(41)	1.401(7)	C(6)-C(11)-C(10)	119.6(6)
Zn(1)-O(40)	*2.123(4)*	O(40)-C(41')	1.43(2)	O(12)-C(12)-O(13)	122.0(5)
Zn(1)-O(50)	*2.139(3)*	C(41)-C(42)	1.496(10)	O(12)-C(12)-C(4)	125.4(5)
Zn(1)-O(12)	*2.146(3)*	C(41')-C(42')	1.51(2)	O(13)-C(12)-C(4)	112.6(4)
Zn(1)-O(32)	*2.169(3)*	O(50)-C(51)	1.432(6)	C(12)-O(12)-Zn(1)	124.6(3)
N(1)-C(5)	1.326(6)	C(51)-C(52)	1.471(9)	C(12)-O(13)-C(14)	116.0(4)
N(1)-N(2)	1.409(6)			O(13)-C(14)-C(15)	110.4(4)
N(2)-C(3)	1.372(6)	O(23)-Zn(1)-O(3)	175.69(12)	C(25)-N(21)-N(22)	105.3(4)
N(2)-C(6)	1.408(7)	O(23)-Zn(1)-O(40)	90.32(13)	C(23)-N(22)-N(21)	112.2(4)
C(3)-O(3)	*1.277(5)*	O(3)-Zn(1)-O(40)	86.28(13)	C(23)-N(22)-C(26)	129.6(4)
C(3)-C(4)	1.399(7)	O(23)-Zn(1)-O(50)	90.13(13)	N(21)-N(22)-C(26)	118.2(4)
C(4)-C(5)	1.408(7)	O(3)-Zn(1)-O(50)	87.36(13)	O(23)-C(23)-N(22)	127.8(4)
C(4)-C(12)	1.449(7)	*O(40)-Zn(1)-O(50)*	*92.31(13)*	O(23)-C(23)-C(24)	126.5(4)
C(6)-C(7)	1.386(7)	O(23)-Zn(1)-O(12)	93.11(14)	N(22)-C(23)-C(24)	105.6(4)
C(6)-C(11)	1.390(8)	*O(3)-Zn(1)-O(12)*	*90.46(14)*	C(23)-O(23)-Zn(1)	127.1(3)
C(7)-C(8)	1.381(8)	O(40)-Zn(1)-O(12)	174.80(12)	C(25)-C(24)-C(32)	128.5(4)
C(8)-C(9)	1.401(9)	O(50)-Zn(1)-O(12)	91.58(13)	C(25)-C(24)-C(23)	104.5(4)
C(9)-C(10)	1.360(9)	*O(23)-Zn(1)-O(32)*	*90.18(13)*	C(32)-C(24)-C(23)	126.7(4)
C(10)-C(11)	1.396(8)	*O(3)-Zn(1)-O(32)*	*92.52(13)*	N(21)-C(25)-C(24)	112.4(4)
C(12)-O(12)	*1.227(6)*	O(40)-Zn(1)-O(32)	91.06(13)	C(27)-C(26)-C(31)	118.8(5)
C(12)-O(13)	1.348(6)	O(50)-Zn(1)-O(32)	176.62(13)	C(27)-C(26)-N(22)	120.7(5)
O(13)-C(14)	1.458(6)	O(12)-Zn(1)-O(32)	85.04(11)	C(31)-C(26)-N(22)	120.5(5)
C(14)-C(15)	1.519(8)	C(5)-N(1)-N(2)	105.0(4)	C(26)-C(27)-C(28)	120.4(5)
N(21)-C(25)	1.318(6)	C(3)-N(2)-C(6)	129.4(4)	C(29)-C(28)-C(27)	120.9(5)
N(21)-N(22)	1.383(6)	C(3)-N(2)-N(1)	111.2(4)	C(28)-C(29)-C(30)	119.5(5)
N(22)-C(23)	1.363(6)	C(6)-N(2)-N(1)	119.0(4)	C(29)-C(30)-C(31)	120.3(5)
N(22)-C(26)	1.432(6)	O(3)-C(3)-N(2)	124.2(4)	C(26)-C(31)-C(30)	120.0(5)
C(23)-O(23)	*1.272(6)*	O(3)-C(3)-C(4)	129.9(4)	O(32)-C(32)-O(33)	120.9(5)
C(23)-C(24)	1.424(6)	N(2)-C(3)-C(4)	105.8(4)	O(32)-C(32)-C(24)	124.7(5)
C(24)-C(25)	1.414(7)	C(3)-O(3)-Zn(1)	124.3(3)	O(33)-C(32)-C(24)	114.5(4)
C(24)-C(32)	1.415(7)	C(3)-C(4)-C(5)	106.4(4)	C(32)-O(32)-Zn(1)	124.3(3)
C(26)-C(27)	1.374(7)	C(3)-C(4)-C(12)	124.0(5)	C(32)-O(33)-C(34)	119.0(4)
C(26)-C(31)	1.380(8)	C(5)-C(4)-C(12)	129.5(5)	O(33)-C(34)-C(35)	111.4(4)
C(27)-C(28)	1.384(8)	N(1)-C(5)-C(4)	111.6(5)	C(41)-O(40)-Zn(1)	126.9(4)
C(28)-C(29)	1.358(9)	C(7)-C(6)-C(11)	119.9(6)	C(41')-O(40)-Zn(1)	134.3(16)
C(29)-C(30)	1.367(8)	C(7)-C(6)-N(2)	119.7(5)	O(40)-C(41)-C(42)	112.4(6)
C(30)-C(31)	1.393(7)	C(11)-C(6)-N(2)	120.4(5)	O(40)-C(41')-C(42')	111(2)
C(32)-O(32)	*1.246(6)*	C(8)-C(7)-C(6)	119.3(7)	C(51)-O(50)-Zn(1)	130.4(3)
C(32)-O(33)	1.330(6)	C(7)-C(8)-C(9)	121.0(6)	O(50)-C(51)-C(52)	113.4(6)
O(33)-C(34)	1.434(6)	C(10)-C(9)-C(8)	119.0(6)		

Table S26. Bond lengths [Å] and angles [°] for **53**.

Cu(1)-O(3)	1.964(15)	C(28)-C(29)	1.378(10)	C(5)-N(1)-N(2)	104.3(9)	C(23)-C(24)-C(25)	104.3(9)
Cu(1)-O(23)	1.978(15)	C(29)-C(30)	1.380(12)	C(3)-N(2)-N(1)	112.0(9)	C(23)-C(24)-C(35)	125.0(10)
Cu(1)-N(51)	1.980(17)	C(30)-O(33)	1.369(9)	C(3)-N(2)-C(6)	126.9(11)	C(25)-C(24)-C(35)	130.6(11)
Cu(1)-N(41)	2.063(14)	C(30)-C(31)	1.400(11)	N(1)-N(2)-C(6)	119.6(10)	N(21)-C(25)-C(24)	114.7(11)
Cu(1)-O(35)	2.248(11)	C(31)-C(32)	1.393(11)	O(3)-C(3)-N(2)	121.1(12)	N(22)-C(26)-C(27)	111.8(12)
Cu(1)-O(15)	2.439(11)	O(33)-C(34)	1.414(11)	O(3)-C(3)-C(4)	134.1(12)	C(32)-C(27)-C(28)	118.6(9)
N(1)-C(5)	1.307(11)	C(35)-O(35)	1.228(9)	N(2)-C(3)-C(4)	104.7(9)	C(32)-C(27)-C(26)	122.0(10)
N(1)-N(2)	1.408(11)	C(35)-O(36)	1.351(10)	C(3)-O(3)-Cu(1)	121.5(11)	C(28)-C(27)-C(26)	119.3(10)
N(2)-C(3)	1.369(11)	O(36)-C(37)	1.442(9)	C(3)-C(4)-C(5)	105.9(9)	C(29)-C(28)-C(27)	119.9(9)
N(2)-C(6)	1.454(11)	C(37)-C(38)	1.495(11)	C(3)-C(4)-C(15)	126.2(10)	C(28)-C(29)-C(30)	121.2(8)
C(3)-O(3)	1.289(11)	N(41)-C(46)	1.305(16)	C(5)-C(4)-C(15)	127.8(11)	O(33)-C(30)-C(29)	116.4(8)
C(3)-C(4)	1.408(12)	N(41)-C(42)	1.333(16)	N(1)-C(5)-C(4)	112.3(10)	O(33)-C(30)-C(31)	124.0(9)
C(4)-C(5)	1.414(10)	C(42)-C(43)	1.364(15)	N(2)-C(6)-C(7)	114.5(12)	C(29)-C(30)-C(31)	119.7(7)
C(4)-C(15)	1.439(10)	C(43)-C(44)	1.365(13)	C(12)-C(7)-C(8)	116.0(10)	C(32)-C(31)-C(30)	118.1(9)
C(6)-C(7)	1.503(11)	C(44)-C(45)	1.385(15)	C(12)-C(7)-C(6)	122.7(11)	C(27)-C(32)-C(31)	122.2(10)
C(7)-C(12)	1.371(12)	C(45)-C(46)	1.413(14)	C(8)-C(7)-C(6)	121.2(11)	C(30)-O(33)-C(34)	118.6(9)
C(7)-C(8)	1.418(11)	N(51)-C(52)	1.335(18)	C(9)-C(8)-C(7)	121.4(9)	O(35)-C(35)-O(36)	123.3(9)
C(8)-C(9)	1.383(10)	N(51)-C(56)	1.378(18)	C(10)-C(9)-C(8)	120.4(8)	O(35)-C(35)-C(24)	125.3(10)
C(9)-C(10)	1.377(12)	C(52)-C(53)	1.403(15)	O(13)-C(10)-C(9)	116.0(9)	O(36)-C(35)-C(24)	111.4(8)
C(10)-O(13)	1.376(10)	C(53)-C(54)	1.381(17)	O(13)-C(10)-C(11)	124.0(9)	C(35)-O(35)-Cu(1)	120.5(7)
C(10)-C(11)	1.403(12)	C(54)-C(55)	1.390(16)	C(9)-C(10)-C(11)	120.1(8)	C(35)-O(36)-C(37)	116.6(7)
C(11)-C(12)	1.389(12)	C(55)-C(56)	1.370(13)	C(12)-C(11)-C(10)	117.7(10)	O(36)-C(37)-C(38)	113.1(8)
O(13)-C(14)	1.417(12)			C(7)-C(12)-C(11)	124.4(11)	C(46)-N(41)-C(42)	120.1(11)
C(15)-O(15)	1.221(9)	O(3)-Cu(1)-O(23)	165.4(9)	C(10)-O(13)-C(14)	118.7(11)	C(46)-N(41)-Cu(1)	122.6(10)
C(15)-O(16)	1.342(10)	O(3)-Cu(1)-N(51)	81.6(6)	O(15)-C(15)-O(16)	120.4(9)	C(42)-N(41)-Cu(1)	117.1(9)
O(16)-C(17)	1.465(9)	O(23)-Cu(1)-N(51)	85.0(6)	O(15)-C(15)-C(4)	125.5(10)	N(41)-C(42)-C(43)	122.8(10)
C(17)-C(18)	1.493(12)	O(3)-Cu(1)-N(41)	96.0(6)	O(16)-C(15)-C(4)	114.0(8)	C(42)-C(43)-C(44)	118.0(10)
N(21)-C(25)	1.309(12)	O(23)-Cu(1)-N(41)	97.7(6)	C(15)-O(15)-Cu(1)	119.5(7)	C(43)-C(44)-C(45)	120.5(9)
N(21)-N(22)	1.404(11)	N(51)-Cu(1)-N(41)	175.6(7)	C(15)-O(16)-C(17)	115.9(6)	C(44)-C(45)-C(46)	117.2(7)
N(22)-C(23)	1.380(10)	O(3)-Cu(1)-O(35)	92.5(6)	O(16)-C(17)-C(18)	108.7(8)	N(41)-C(46)-C(45)	121.4(10)
N(22)-C(26)	1.453(11)	O(23)-Cu(1)-O(35)	94.0(5)	C(25)-N(21)-N(22)	102.6(9)	C(52)-N(51)-C(56)	115.9(12)
C(23)-O(23)	1.281(11)	N(51)-Cu(1)-O(35)	93.1(5)	C(23)-N(22)-N(21)	112.1(10)	C(52)-N(51)-Cu(1)	123.8(11)
C(23)-C(24)	1.403(12)	N(41)-Cu(1)-O(35)	83.3(4)	C(23)-N(22)-C(26)	123.2(11)	C(56)-N(51)-Cu(1)	120.3(9)
C(24)-C(25)	1.408(11)	O(3)-Cu(1)-O(15)	89.1(5)	N(21)-N(22)-C(26)	122.5(10)	N(51)-C(52)-C(53)	124.9(12)
C(24)-C(35)	1.443(10)	O(23)-Cu(1)-O(15)	86.7(5)	O(23)-C(23)-N(22)	118.9(12)	C(54)-C(53)-C(52)	117.5(10)
C(26)-C(27)	1.510(11)	N(51)-Cu(1)-O(15)	96.3(4)	O(23)-C(23)-C(24)	135.8(11)	C(53)-C(54)-C(55)	119.1(8)
C(27)-C(32)	1.375(12)	N(41)-Cu(1)-O(15)	87.4(5)	N(22)-C(23)-C(24)	105.3(9)	C(56)-C(55)-C(54)	119.7(9)
C(27)-C(28)	1.392(11)	O(35)-Cu(1)-O(15)	170.6(5)	C(23)-O(23)-Cu(1)	117.8(9)	C(55)-C(56)-N(51)	122.9(10)

Table S27. Bond lengths [Å] and angles [°] for **54**.

Cu(1A)-O(3A)	*1.927(2)*	C(3B)-O(3B)	1.275(4)	N(1A)-N(2A)-C(6A)	119.7(2)	O(3B)#2-Cu(1B)-N(1A)#3	94.12(10)
Cu(1A)-O(3A)#1	*1.927(2)*	C(3B)-C(4B)	1.401(4)	O(3A)-C(3A)-N(2A)	123.0(3)	O(3B)-Cu(1B)-N(1A)#3	85.88(10)
Cu(1A)-O(12A)	*2.001(2)*	C(4B)-C(12B)	1.416(4)	O(3A)-C(3A)-C(4A)	131.3(3)	O(12B)-Cu(1B)-N(1A)#3	91.31(9)
Cu(1A)-O(12A)#1	*2.001(2)*	C(4B)-C(5B)	1.417(4)	N(2A)-C(3A)-C(4A)	105.7(3)	O(12B)#2-Cu(1B)-N(1A)#3	88.69(9)
Cu(1A)-N(1B)#1	*2.519(3)*	C(6B)-C(11B)	1.390(5)	C(3A)-O(3A)-Cu(1A)	120.2(2)	N(1A)#4-Cu(1B)-N(1A)#3	180.0
Cu(1A)-N(1B)	*2.519(3)*	C(6B)-C(7B)	1.394(5)	C(3A)-C(4A)-C(5A)	105.6(3)	C(5B)-N(1B)-N(2B)	104.9(3)
N(1A)-C(5A)	1.307(4)	C(7B)-C(8B)	1.382(5)	C(3A)-C(4A)-C(12A)	123.2(3)	C(5B)-N(1B)-Cu(1A)	106.1(2)
N(1A)-N(2A)	1.402(4)	C(8B)-C(9B)	1.379(6)	C(5A)-C(4A)-C(12A)	131.2(3)	N(2B)-N(1B)-Cu(1A)	120.3(2)
N(2A)-C(3A)	1.379(4)	C(9B)-C(10B)	1.384(6)	N(1A)-C(5A)-C(4A)	112.7(3)	C(3B)-N(2B)-N(1B)	111.6(3)
N(2A)-C(6A)	1.411(4)	C(10B)-C(11B)	1.384(5)	C(11A)-C(6A)-C(7A)	119.4(3)	C(3B)-N(2B)-C(6B)	128.3(3)
C(3A)-O(3A)	*1.275(4)*	C(12B)-O(12B)	1.251(4)	C(11A)-C(6A)-N(2A)	121.2(3)	N(1B)-N(2B)-C(6B)	119.2(3)
C(3A)-C(4A)	1.398(4)	C(12B)-O(13B)	1.327(4)	C(7A)-C(6A)-N(2A)	119.5(3)	O(3B)-C(3B)-N(2B)	123.9(3)
C(4A)-C(5A)	1.410(4)	O(13B)-C(14B)	1.452(4)	C(8A)-C(7A)-C(6A)	119.6(3)	O(3B)-C(3B)-C(4B)	130.3(3)
C(4A)-C(12A)	1.423(4)	C(14B)-C(15B)	1.510(5)	C(9A)-C(8A)-C(7A)	121.0(3)	N(2B)-C(3B)-C(4B)	105.8(3)
C(6A)-C(11A)	1.389(5)			C(10A)-C(9A)-C(8A)	119.0(4)	C(3B)-O(3B)-Cu(1B)	119.7(2)
C(6A)-C(7A)	1.396(4)	O(3A)-Cu(1A)-O(3A)#1	180.0	C(11A)-C(10A)-C(9A)	121.2(4)	C(3B)-C(4B)-C(12B)	124.0(3)
C(7A)-C(8A)	1.381(5)	*O(3A)-Cu(1A)-O(12A)*	*94.59(9)*	C(10A)-C(11A)-C(6A)	119.8(3)	C(3B)-C(4B)-C(5B)	105.3(3)
C(8A)-C(9A)	1.379(6)	*O(3A)#1-Cu(1A)-O(12A)*	*85.41(9)*	O(12A)-C(12A)-O(13A)	120.9(3)	C(12B)-C(4B)-C(5B)	130.7(3)
C(9A)-C(10A)	1.379(5)	*O(3A)-Cu(1A)-O(12A)#1*	*85.41(9)*	O(12A)-C(12A)-C(4A)	123.8(3)	N(1B)-C(5B)-C(4B)	112.5(3)
C(10A)-C(11A)	1.378(5)	*O(3A)#1-Cu(1A)-O(12A)#1*	*94.59(9)*	O(13A)-C(12A)-C(4A)	115.3(3)	C(11B)-C(6B)-C(7B)	120.0(3)
C(12A)-O(12A)	*1.249(4)*	O(12A)-Cu(1A)-O(12A)#1	180.0	C(12A)-O(12A)-Cu(1A)	125.4(2)	C(11B)-C(6B)-N(2B)	120.9(3)
C(12A)-O(13A)	1.328(4)	*O(3A)-Cu(1A)-N(1B)#1*	*94.44(10)*	C(12A)-O(13A)-C(14A)	116.7(2)	C(7B)-C(6B)-N(2B)	119.0(3)
O(13A)-C(14A)	1.447(4)	*O(3A)#1-Cu(1A)-N(1B)#1*	*85.56(10)*	O(13A)-C(14A)-C(15A)	106.7(3)	C(8B)-C(7B)-C(6B)	119.4(4)
C(14A)-C(15A)	1.499(5)	*O(12A)-Cu(1A)-N(1B)#1*	*87.63(9)*	O(3B)#2-Cu(1B)-O(3B)	180.0	C(9B)-C(8B)-C(7B)	121.0(4)
Cu(1B)-O(3B)#2	1.932(2)	*O(12A)#1-Cu(1A)-N(1B)#1*	*92.37(9)*	O(3B)#2-Cu(1B)-O(12B)	85.49(9)	C(8B)-C(9B)-C(10B)	119.4(4)
Cu(1B)-O(3B)	1.932(2)	*O(3A)-Cu(1A)-N(1B)*	*85.56(10)*	O(3B)-Cu(1B)-O(12B)	94.51(9)	C(11B)-C(10B)-C(9B)	120.7(4)
Cu(1B)-O(12B)	2.004(2)	*O(3A)#1-Cu(1A)-N(1B)*	*94.44(10)*	O(3B)#2-Cu(1B)-O(12B)#2	94.51(9)	C(10B)-C(11B)-C(6B)	119.5(3)
Cu(1B)-O(12B)#2	2.004(2)	*O(12A)-Cu(1A)-N(1B)*	*92.37(9)*	O(3B)-Cu(1B)-O(12B)#2	85.49(9)	O(12B)-C(12B)-O(13B)	120.1(3)
Cu(1B)-N(1A)#3	2.501(3)	*O(12A)#1-Cu(1A)-N(1B)*	*87.63(9)*	O(12B)-Cu(1B)-O(12B)#2	180.0	O(12B)-C(12B)-C(4B)	124.1(3)
Cu(1B)-N(1A)#4	2.501(3)	N(1B)#1-Cu(1A)-N(1B)	180.0	O(3B)#2-Cu(1B)-N(1A)#4	85.88(10)	O(13B)-C(12B)-C(4B)	115.8(3)
N(1B)-C(5B)	1.309(4)	C(5A)-N(1A)-N(2A)	104.9(3)	O(3B)-Cu(1B)-N(1A)#4	94.12(10)	C(12B)-O(12B)-Cu(1B)	124.1(2)
N(1B)-N(2B)	1.400(4)	C(3A)-N(2A)-N(1A)	111.2(3)	O(12B)-Cu(1B)-N(1A)#4	88.69(9)	C(12B)-O(13B)-C(14B)	117.1(2)
N(2B)-C(3B)	1.371(4)	C(3A)-N(2A)-C(6A)	128.1(3)	O(12B)#2-Cu(1B)-N(1A)#4	91.31(9)	O(13B)-C(14B)-C(15B)	106.7(3)
N(2B)-C(6B)	1.418(4)						

Table S28. Bond lengths [Å] and angles [°] for **55**.

Cu(1)-O(3)	*1.948(4)*	C(34)-C(45)	1.407(7)	C(3)-N(2)-C(6)	126.2(4)	C(33)-N(32)-N(31)	113.1(4)
Cu(1)-O(33)	*1.948(4)*	C(34)-C(35)	1.433(7)	N(1)-N(2)-C(6)	121.1(4)	C(33)-N(32)-C(36)	127.0(5)
Cu(1)-O(45)	*1.985(4)*	C(36)-C(37)	1.518(8)	O(3)-C(3)-N(2)	123.1(5)	N(31)-N(32)-C(36)	119.9(4)
Cu(1)-O(15)	*2.000(4)*	C(37)-C(38)	1.390(7)	O(3)-C(3)-C(4)	131.8(5)	O(33)-C(33)-N(32)	123.2(5)
Cu(1)-N(31)#1	*2.458(4)*	C(37)-C(42)	1.392(8)	N(2)-C(3)-C(4)	105.1(4)	O(33)-C(33)-C(34)	131.7(5)
Cu(1)-N(1)#2	*2.626(4)*	C(38)-C(39)	1.393(8)	C(3)-O(3)-Cu(1)	118.9(3)	N(32)-C(33)-C(34)	105.1(4)
N(1)-C(5)	1.304(7)	C(39)-C(40)	1.396(8)	C(15)-C(4)-C(3)	123.3(4)	C(33)-O(33)-Cu(1)	118.7(4)
N(1)-N(2)	1.386(6)	C(40)-O(43)	1.371(7)	C(15)-C(4)-C(5)	131.7(5)	C(45)-C(34)-C(35)	133.5(5)
N(2)-C(3)	1.364(6)	C(40)-C(41)	1.387(8)	C(3)-C(4)-C(5)	104.9(5)	C(45)-C(34)-C(33)	122.7(5)
N(2)-C(6)	1.443(7)	C(41)-C(42)	1.375(8)	N(1)-C(5)-C(4)	112.0(5)	C(35)-C(34)-C(33)	103.7(4)
C(3)-O(3)	*1.271(6)*	O(43)-C(44)	1.439(7)	N(2)-C(6)-C(7)	112.9(5)	N(31)-C(35)-C(34)	113.2(5)
C(3)-C(4)	1.421(7)	*C(45)-O(45)*	*1.265(6)*	C(12)-C(7)-C(8)	118.4(5)	N(32)-C(36)-C(37)	114.5(5)
C(4)-C(15)	1.396(7)	C(45)-C(46)	1.496(7)	C(12)-C(7)-C(6)	119.8(5)	C(38)-C(37)-C(42)	118.5(5)
C(4)-C(5)	1.423(7)	C(46)-C(51)	1.384(9)	C(8)-C(7)-C(6)	121.8(5)	C(38)-C(37)-C(36)	122.3(5)
C(6)-C(7)	1.514(8)	C(46)-C(47)	1.396(8)	C(9)-C(8)-C(7)	121.6(5)	C(42)-C(37)-C(36)	119.1(5)
C(7)-C(12)	1.369(8)	C(47)-C(48)	1.397(8)	C(8)-C(9)-C(10)	119.1(6)	C(37)-C(38)-C(39)	120.9(5)
C(7)-C(8)	1.401(8)	C(48)-C(49)	1.381(11)	C(9)-C(10)-O(13)	115.9(5)	C(38)-C(39)-C(40)	119.5(5)
C(8)-C(9)	1.378(8)	C(49)-C(50)	1.371(11)	C(9)-C(10)-C(11)	121.1(6)	O(43)-C(40)-C(41)	115.4(5)
C(9)-C(10)	1.382(8)	C(50)-C(51)	1.385(9)	O(13)-C(10)-C(11)	123.0(6)	O(43)-C(40)-C(39)	125.0(5)
C(10)-O(13)	1.382(7)			C(10)-C(11)-C(12)	118.6(6)	C(41)-C(40)-C(39)	119.6(5)
C(10)-C(11)	1.383(8)	O(3)-Cu(1)-O(33)	178.37(18)	C(7)-C(12)-C(11)	121.1(6)	C(42)-C(41)-C(40)	120.3(5)
C(11)-C(12)	1.416(9)	O(3)-Cu(1)-O(45)	86.06(15)	C(10)-O(13)-C(14)	117.1(5)	C(41)-C(42)-C(37)	121.2(5)
O(13)-C(14)	1.415(8)	O(33)-Cu(1)-O(45)	93.43(15)	O(15)-C(15)-C(4)	124.3(5)	C(40)-O(43)-C(44)	116.7(4)
C(15)-O(15)	*1.268(6)*	*O(3)-Cu(1)-O(15)*	*94.83(15)*	O(15)-C(15)-C(16)	116.4(4)	O(45)-C(45)-C(34)	121.8(5)
C(15)-C(16)	1.504(7)	*O(33)-Cu(1)-O(15)*	*85.58(16)*	C(4)-C(15)-C(16)	119.3(4)	O(45)-C(45)-C(46)	116.4(5)
C(16)-C(17)	1.382(8)	O(45)-Cu(1)-O(15)	175.91(16)	C(15)-O(15)-Cu(1)	124.0(3)	C(34)-C(45)-C(46)	121.8(5)
C(16)-C(21)	1.385(8)	O(3)-Cu(1)-N(31)#1	92.49(15)	C(17)-C(16)-C(21)	119.9(5)	C(45)-O(45)-Cu(1)	127.9(3)
C(17)-C(18)	1.400(8)	*O(33)-Cu(1)-N(31)#1*	*89.02(16)*	C(17)-C(16)-C(15)	121.0(5)	C(51)-C(46)-C(47)	119.9(5)
C(18)-C(19)	1.372(9)	O(45)-Cu(1)-N(31)#1	86.78(15)	C(21)-C(16)-C(15)	119.1(5)	C(51)-C(46)-C(45)	118.1(5)
C(19)-C(20)	1.393(9)	*O(15)-Cu(1)-N(31)#1*	*97.17(15)*	C(16)-C(17)-C(18)	119.9(5)	C(47)-C(46)-C(45)	122.0(5)
C(20)-C(21)	1.397(8)	*O(3)-Cu(1)-N(1)#2*	*84.12(15)*	C(19)-C(18)-C(17)	120.5(6)	C(46)-C(47)-C(48)	119.0(6)
N(31)-C(35)	1.308(7)	O(33)-Cu(1)-N(1)#2	94.38(15)	C(18)-C(19)-C(20)	119.8(6)	C(49)-C(48)-C(47)	120.7(6)
N(31)-N(32)	1.385(6)	O(45)-Cu(1)-N(1)#2	94.08(14)	C(19)-C(20)-C(21)	119.7(6)	C(50)-C(49)-C(48)	119.5(6)
N(32)-C(33)	1.369(6)	*O(15)-Cu(1)-N(1)#2*	*82.05(14)*	C(16)-C(21)-C(20)	120.2(5)	C(49)-C(50)-C(51)	120.9(7)
N(32)-C(36)	1.451(7)	N(31)#1-Cu(1)-N(1)#2	176.43(16)	C(35)-N(31)-N(32)	104.9(4)	C(46)-C(51)-C(50)	119.9(6)
C(33)-O(33)	*1.270(6)*	C(5)-N(1)-N(2)	105.6(4)	C(35)-N(31)-Cu(1)#3	125.3(4)		
C(33)-C(34)	1.436(8)	C(3)-N(2)-N(1)	112.3(4)	N(32)-N(31)-Cu(1)#3	128.1(3)		

Table S29. Bond lengths [Å] and angles [°] for **56**.

Cu(1)-O(3)	*1.9790(13)*	N(11)-Cu(1)-O(6)	88.13(6)
Cu(1)-O(3)#1	*1.9790(13)*	N(11)#1-Cu(1)-O(6)	91.87(6)
Cu(1)-N(11)	*2.0310(16)*	*O(3)-Cu(1)-O(6)#1*	*91.01(5)*
Cu(1)-N(11)#1	*2.0311(16)*	*O(3)#1-Cu(1)-O(6)#1*	*88.99(5)*
Cu(1)-O(6)	*2.3783(13)*	N(11)-Cu(1)-O(6)#1	91.87(6)
Cu(1)-O(6)#1	*2.3783(13)*	N(11)#1-Cu(1)-O(6)#1	88.13(6)
N(1)-C(5)	1.311(3)	O(6)-Cu(1)-O(6)#1	180.0
N(1)-N(2)	1.380(2)	C(5)-N(1)-N(2)	104.34(15)
N(2)-C(3)	1.361(2)	C(3)-N(2)-N(1)	112.99(15)
C(3)-O(3)	*1.295(2)*	O(3)-C(3)-N(2)	121.57(16)
C(3)-C(4)	1.409(3)	O(3)-C(3)-C(4)	133.25(17)
C(4)-C(5)	1.416(3)	N(2)-C(3)-C(4)	105.18(15)
C(4)-C(6)	1.438(3)	C(3)-O(3)-Cu(1)	121.47(11)
C(6)-O(6)	*1.225(2)*	C(3)-C(4)-C(5)	104.74(16)
C(6)-O(7)	1.348(2)	C(3)-C(4)-C(6)	125.67(17)
O(7)-C(8)	1.453(2)	C(5)-C(4)-C(6)	129.48(18)
C(8)-C(9)	1.504(3)	N(1)-C(5)-C(4)	112.74(17)
N(11)-C(16)	1.338(3)	O(6)-C(6)-O(7)	121.98(17)
N(11)-C(12)	1.343(2)	O(6)-C(6)-C(4)	125.37(17)
C(12)-C(13)	1.382(3)	O(7)-C(6)-C(4)	112.64(16)
C(13)-C(14)	1.387(3)	C(6)-O(6)-Cu(1)	119.69(12)
C(14)-C(15)	1.379(3)	C(6)-O(7)-C(8)	115.92(15)
C(15)-C(16)	1.386(3)	O(7)-C(8)-C(9)	107.75(16)
		C(16)-N(11)-C(12)	117.73(17)
O(3)-Cu(1)-O(3)#1	180.0	C(16)-N(11)-Cu(1)	120.38(13)
O(3)-Cu(1)-N(11)	*92.14(6)*	C(12)-N(11)-Cu(1)	121.60(13)
O(3)#1-Cu(1)-N(11)	*87.86(6)*	N(11)-C(12)-C(13)	122.73(18)
O(3)-Cu(1)-N(11)#1	*87.86(6)*	C(12)-C(13)-C(14)	119.02(19)
O(3)#1-Cu(1)-N(11)#1	*92.14(6)*	C(15)-C(14)-C(13)	118.63(19)
N(11)-Cu(1)-N(11)#1	180.0	C(14)-C(15)-C(16)	118.9(2)
O(3)-Cu(1)-O(6)	*88.99(5)*	N(11)-C(16)-C(15)	123.00(19)
O(3)#1-Cu(1)-O(6)	*91.01(5)*		

Table S30. Bond lengths [Å] and angles [°] for **57**.

Ni(1)-O(3)	*1.96(3)*	C(28)-C(29)	1.384(10)	C(5)-N(1)-N(2)	104.3(9)	C(23)-C(24)-C(25)	104.6(8)
Ni(1)-N(51)	*2.01(2)*	C(29)-C(30)	1.352(11)	C(3)-N(2)-N(1)	113.1(9)	C(23)-C(24)-C(35)	121.2(9)
Ni(1)-O(35)	*2.052(16)*	C(30)-O(33)	1.372(9)	C(3)-N(2)-C(6)	126.1(11)	C(25)-C(24)-C(35)	133.5(11)
Ni(1)-O(15)	*2.090(16)*	C(30)-C(31)	1.387(11)	N(1)-N(2)-C(6)	120.5(10)	N(21)-C(25)-C(24)	115.3(11)
Ni(1)-O(23)	*2.11(3)*	C(31)-C(32)	1.405(11)	O(3)-C(3)-N(2)	125.1(12)	N(22)-C(26)-C(27)	113.4(12)
Ni(1)-N(41)	*2.16(2)*	O(33)-C(34)	1.421(11)	O(3)-C(3)-C(4)	131.5(12)	C(32)-C(27)-C(28)	116.6(9)
N(1)-C(5)	1.282(11)	*C(35)-O(35)*	*1.249(10)*	N(2)-C(3)-C(4)	103.4(9)	C(32)-C(27)-C(26)	122.9(10)
N(1)-N(2)	1.401(10)	C(35)-O(36)	1.354(11)	C(3)-O(3)-Ni(1)	118.7(12)	C(28)-C(27)-C(26)	120.5(11)
N(2)-C(3)	1.369(10)	O(36)-C(37)	1.460(9)	C(3)-C(4)-C(5)	106.0(9)	C(27)-C(28)-C(29)	121.3(10)
N(2)-C(6)	1.446(10)	C(37)-C(38)	1.483(10)	C(3)-C(4)-C(15)	126.2(10)	C(30)-C(29)-C(28)	121.6(9)
C(3)-O(3)	*1.267(11)*	N(41)-C(46)	1.32(2)	C(5)-C(4)-C(15)	127.8(11)	C(29)-C(30)-O(33)	116.5(9)
C(3)-C(4)	1.413(10)	N(41)-C(42)	1.333(18)	N(1)-C(5)-C(4)	112.8(11)	C(29)-C(30)-C(31)	119.2(8)
C(4)-C(5)	1.417(11)	C(42)-C(43)	1.44(2)	N(2)-C(6)-C(7)	113.6(12)	O(33)-C(30)-C(31)	124.3(9)
C(4)-C(15)	1.439(10)	C(43)-C(44)	1.323(19)	C(12)-C(7)-C(8)	119.2(9)	C(30)-C(31)-C(32)	118.6(10)
C(6)-C(7)	1.508(10)	C(44)-C(45)	1.367(16)	C(12)-C(7)-C(6)	119.6(11)	C(27)-C(32)-C(31)	122.6(10)
C(7)-C(12)	1.376(11)	C(45)-C(46)	1.376(19)	C(8)-C(7)-C(6)	121.1(11)	C(30)-O(33)-C(34)	118.7(9)
C(7)-C(8)	1.379(11)	N(51)-C(56)	1.35(2)	C(7)-C(8)-C(9)	119.5(10)	O(35)-C(35)-O(36)	117.9(9)
C(8)-C(9)	1.387(10)	N(51)-C(52)	1.35(2)	C(10)-C(9)-C(8)	121.2(9)	O(35)-C(35)-C(24)	127.2(10)
C(9)-C(10)	1.354(11)	C(52)-C(53)	1.32(2)	C(9)-C(10)-O(13)	115.6(9)	O(36)-C(35)-C(24)	111.8(7)
C(10)-O(13)	1.375(9)	C(53)-C(54)	1.42(2)	C(9)-C(10)-C(11)	120.7(9)	C(35)-O(35)-Ni(1)	123.1(10)
C(10)-C(11)	1.385(11)	C(54)-C(55)	1.359(18)	O(13)-C(10)-C(11)	123.6(10)	C(35)-O(36)-C(37)	117.6(7)
C(11)-C(12)	1.403(11)	C(55)-C(56)	1.38(2)	C(10)-C(11)-C(12)	117.9(10)	O(36)-C(37)-C(38)	111.4(8)
O(13)-C(14)	1.422(12)			C(7)-C(12)-C(11)	121.4(11)	C(46)-N(41)-C(42)	121.9(12)
C(15)-O(15)	*1.238(12)*	O(3)-Ni(1)-N(51)	87.6(13)	C(10)-O(13)-C(14)	116.5(10)	C(46)-N(41)-Ni(1)	122.4(10)
C(15)-O(16)	1.382(11)	O(3)-Ni(1)-O(35)	86.2(8)	O(15)-C(15)-O(16)	119.8(12)	C(42)-N(41)-Ni(1)	115.6(11)
O(16)-C(17)	1.485(9)	N(51)-Ni(1)-O(35)	92.6(8)	O(15)-C(15)-C(4)	122.9(11)	N(41)-C(42)-C(43)	118.7(12)
C(17)-C(18)	1.468(11)	*O(3)-Ni(1)-O(15)*	*96.0(9)*	O(16)-C(15)-C(4)	113.2(11)	C(44)-C(43)-C(42)	118.1(10)
N(21)-C(25)	1.305(11)	N(51)-Ni(1)-O(15)	96.6(7)	C(15)-O(15)-Ni(1)	123.2(11)	C(43)-C(44)-C(45)	122.0(11)
N(21)-N(22)	1.397(10)	O(35)-Ni(1)-O(15)	170.6(12)	C(15)-O(16)-C(17)	116.1(8)	C(44)-C(45)-C(46)	118.6(11)
N(22)-C(23)	1.374(10)	O(3)-Ni(1)-O(23)	176.8(17)	C(18)-C(17)-O(16)	110.4(8)	N(41)-C(46)-C(45)	120.7(11)
N(22)-C(26)	1.450(10)	N(51)-Ni(1)-O(23)	89.7(12)	C(25)-N(21)-N(22)	101.0(10)	C(56)-N(51)-C(52)	112.5(13)
C(23)-O(23)	*1.266(10)*	*O(35)-Ni(1)-O(23)*	*92.2(9)*	C(23)-N(22)-N(21)	115.4(9)	C(56)-N(51)-Ni(1)	121.8(11)
C(23)-C(24)	1.406(10)	*O(15)-Ni(1)-O(23)*	*86.0(8)*	C(23)-N(22)-C(26)	121.4(11)	C(52)-N(51)-Ni(1)	125.6(13)
C(24)-C(25)	1.418(11)	*O(3)-Ni(1)-N(41)*	*90.2(13)*	N(21)-N(22)-C(26)	123.3(11)	C(53)-C(52)-N(51)	129.0(13)
C(24)-C(35)	1.439(9)	N(51)-Ni(1)-N(41)	175.3(9)	O(23)-C(23)-N(22)	121.5(11)	C(52)-C(53)-C(54)	116.2(10)
C(26)-C(27)	1.511(10)	O(35)-Ni(1)-N(41)	83.1(6)	O(23)-C(23)-C(24)	135.0(11)	C(55)-C(54)-C(53)	118.2(10)
C(27)-C(32)	1.377(10)	O(15)-Ni(1)-N(41)	87.8(8)	N(22)-C(23)-C(24)	103.5(9)	C(54)-C(55)-C(56)	119.8(12)
C(27)-C(28)	1.383(11)	*O(23)-Ni(1)-N(41)*	*92.4(12)*	C(23)-O(23)-Ni(1)	116.4(11)	N(51)-C(56)-C(55)	124.2(11)

Table S31. Bond lengths [Å] and angles [°] for **58**.

Ni(1)-O(23)	*2.025(3)*	C(27)-C(28)	1.402(7)	C(5)-N(1)-N(2)	104.6(3)	C(23)-O(23)-Ni(1)	115.5(2)
Ni(1)-O(3)	*2.050(2)*	C(28)-C(29)	1.361(8)	N(1)-N(2)-C(3)	111.7(3)	C(23)-C(24)-C(32)	124.0(4)
Ni(1)-N(50)	*2.071(3)*	C(29)-C(30)	1.341(8)	N(1)-N(2)-C(6)	118.6(3)	C(23)-C(24)-C(25)	105.0(3)
Ni(1)-O(12)	*2.080(3)*	C(30)-C(31)	1.389(7)	C(3)-N(2)-C(6)	129.6(3)	C(32)-C(24)-C(25)	130.9(4)
Ni(1)-N(40)	*2.084(3)*	*C(32)-O(32)*	*1.236(5)*	O(3)-C(3)-N(2)	123.1(3)	N(21)-C(25)-C(24)	112.1(4)
Ni(1)-O(32)	*2.107(3)*	C(32)-O(33)	1.361(5)	O(3)-C(3)-C(4)	132.1(3)	C(31)-C(26)-C(27)	119.6(4)
N(1)-C(5)	1.300(6)	O(33)-C(34)	1.442(5)	N(2)-C(3)-C(4)	104.8(3)	C(31)-C(26)-N(22)	122.8(4)
N(1)-N(2)	1.393(4)	C(34)-C(35)	1.476(7)	C(3)-O(3)-Ni(1)	119.0(2)	C(27)-C(26)-N(22)	117.6(4)
N(2)-C(3)	1.394(4)	N(40)-C(41)	1.336(5)	C(3)-C(4)-C(5)	105.3(4)	C(26)-C(27)-C(28)	118.5(5)
N(2)-C(6)	1.412(5)	N(40)-C(45)	1.337(5)	C(3)-C(4)-C(12)	125.0(3)	C(29)-C(28)-C(27)	121.0(5)
C(3)-O(3)	*1.272(4)*	C(41)-C(42)	1.392(6)	C(5)-C(4)-C(12)	129.7(4)	C(30)-C(29)-C(28)	119.0(5)
C(3)-C(4)	1.402(5)	C(42)-C(43)	1.379(6)	N(1)-C(5)-C(4)	113.6(4)	C(29)-C(30)-C(31)	122.0(6)
C(4)-C(5)	1.412(6)	C(43)-C(44)	1.376(6)	C(11)-C(6)-C(7)	120.9(4)	C(26)-C(31)-C(30)	120.0(5)
C(4)-C(12)	1.421(5)	C(44)-C(45)	1.384(5)	C(11)-C(6)-N(2)	120.3(4)	O(32)-C(32)-O(33)	122.1(4)
C(6)-C(11)	1.378(6)	N(50)-C(55)	1.327(5)	C(7)-C(6)-N(2)	118.8(4)	O(32)-C(32)-C(24)	124.8(4)
C(6)-C(7)	1.386(6)	N(50)-C(51)	1.346(5)	C(8)-C(7)-C(6)	119.5(5)	O(33)-C(32)-C(24)	113.1(4)
C(7)-C(8)	1.379(7)	C(51)-C(52)	1.382(6)	C(7)-C(8)-C(9)	120.1(5)	C(32)-O(32)-Ni(1)	122.2(3)
C(8)-C(9)	1.381(8)	C(52)-C(53)	1.372(6)	C(10)-C(9)-C(8)	119.6(5)	C(32)-O(33)-C(34)	116.4(3)
C(9)-C(10)	1.374(7)	C(53)-C(54)	1.376(6)	C(9)-C(10)-C(11)	121.3(5)	O(33)-C(34)-C(35)	109.1(4)
C(10)-C(11)	1.380(6)	C(54)-C(55)	1.377(6)	C(6)-C(11)-C(10)	118.7(4)	C(41)-N(40)-C(45)	117.3(3)
C(12)-O(12)	*1.250(5)*			O(12)-C(12)-O(13)	120.8(4)	C(41)-N(40)-Ni(1)	121.6(3)
C(12)-O(13)	1.330(5)	O(23)-Ni(1)-O(3)	87.50(11)	O(12)-C(12)-C(4)	124.5(4)	C(45)-N(40)-Ni(1)	121.0(3)
O(13)-C(14)	1.446(6)	O(23)-Ni(1)-N(50)	177.60(12)	O(13)-C(12)-C(4)	114.7(3)	N(40)-C(41)-C(42)	123.3(4)
O(13)-C(14')	1.454(19)	O(3)-Ni(1)-N(50)	90.65(11)	C(12)-O(12)-Ni(1)	124.5(3)	C(43)-C(42)-C(41)	118.3(4)
C(14)-C(15)	1.480(7)	O(23)-Ni(1)-O(12)	87.09(11)	C(12)-O(13)-C(14)	117.4(4)	C(44)-C(43)-C(42)	119.2(4)
C(14')-C(15')	1.478(19)	*O(3)-Ni(1)-O(12)*	*92.35(10)*	C(12)-O(13)-C(14')	108.5(17)	C(43)-C(44)-C(45)	118.7(4)
N(21)-C(25)	1.307(5)	N(50)-Ni(1)-O(12)	91.46(11)	O(13)-C(14)-C(15)	107.9(4)	N(40)-C(45)-C(44)	123.2(4)
N(21)-N(22)	1.391(5)	O(23)-Ni(1)-N(40)	90.45(11)	O(13)-C(14')-C(15')	108.0(18)	C(55)-N(50)-C(51)	117.5(3)
N(22)-C(23)	1.376(5)	O(3)-Ni(1)-N(40)	177.94(12)	C(25)-N(21)-N(22)	105.7(3)	C(55)-N(50)-Ni(1)	119.5(2)
N(22)-C(26)	1.424(5)	*N(50)-Ni(1)-N(40)*	*91.41(12)*	C(23)-N(22)-N(21)	111.4(3)	C(51)-N(50)-Ni(1)	122.9(3)
C(23)-O(23)	*1.285(5)*	O(12)-Ni(1)-N(40)	87.70(11)	C(23)-N(22)-C(26)	127.6(4)	N(50)-C(51)-C(52)	122.4(4)
C(23)-C(24)	1.410(6)	*O(23)-Ni(1)-O(32)*	*90.33(11)*	N(21)-N(22)-C(26)	121.0(3)	C(53)-C(52)-C(51)	119.3(4)
C(24)-C(32)	1.412(6)	*O(3)-Ni(1)-O(32)*	*87.88(10)*	O(23)-C(23)-N(22)	123.5(4)	C(52)-C(53)-C(54)	118.3(4)
C(24)-C(25)	1.424(5)	N(50)-Ni(1)-O(32)	91.12(11)	O(23)-C(23)-C(24)	130.7(4)	C(53)-C(54)-C(55)	119.3(4)
C(26)-C(31)	1.348(7)	O(12)-Ni(1)-O(32)	177.41(11)	N(22)-C(23)-C(24)	105.7(3)	N(50)-C(55)-C(54)	123.1(3)
C(26)-C(27)	1.402(6)	N(40)-Ni(1)-O(32)	91.98(12)				

Table S32. Bond lengths [Å] and angles [°] for **60**.

Ni(1)-O(13)	*2.0613(16)*	N(31)-C(32)	1.339(3)	C(6)-O(7)-C(8)	117.10(19)
Ni(1)-O(3)	*2.0686(15)*	C(32)-C(33)	1.387(4)	O(7)-C(8)-C(9)	109.9(2)
Ni(1)-O(16)	*2.0726(15)*	C(33)-C(34)	1.370(6)	C(15)-N(11)-N(12)	104.32(18)
Ni(1)-O(6)	*2.0785(15)*	C(34)-C(35)	1.374(6)	C(13)-N(12)-N(11)	113.28(17)
Ni(1)-N(31)	*2.088(2)*	C(35)-C(36)	1.382(5)	O(13)-C(13)-N(12)	123.34(19)
Ni(1)-N(21)	*2.0895(19)*			O(13)-C(13)-C(14)	131.5(2)
N(1)-C(5)	1.309(3)	O(13)-Ni(1)-O(3)	178.81(7)	N(12)-C(13)-C(14)	105.13(18)
N(1)-N(2)	1.386(3)	*O(13)-Ni(1)-O(16)*	*94.11(6)*	C(13)-O(13)-Ni(1)	117.68(13)
N(2)-C(3)	1.354(3)	O(3)-Ni(1)-O(16)	85.27(6)	C(13)-C(14)-C(16)	126.0(2)
C(3)-O(3)	*1.284(3)*	*O(13)-Ni(1)-O(6)*	*86.97(6)*	C(13)-C(14)-C(15)	104.75(19)
C(3)-C(4)	1.414(3)	*O(3)-Ni(1)-O(6)*	*93.65(6)*	C(16)-C(14)-C(15)	128.8(2)
C(4)-C(6)	1.416(3)	O(16)-Ni(1)-O(6)	178.91(6)	N(11)-C(15)-C(14)	112.5(2)
C(4)-C(5)	1.418(3)	O(13)-Ni(1)-N(31)	89.58(7)	O(16)-C(16)-O(17)	120.9(2)
C(6)-O(6)	*1.243(3)*	O(3)-Ni(1)-N(31)	91.44(7)	O(16)-C(16)-C(14)	125.19(19)
C(6)-O(7)	1.341(3)	O(16)-Ni(1)-N(31)	90.45(7)	O(17)-C(16)-C(14)	113.88(19)
O(7)-C(8)	1.446(3)	O(6)-Ni(1)-N(31)	89.76(8)	C(16)-O(16)-Ni(1)	123.86(14)
C(8)-C(9)	1.512(5)	*O(13)-Ni(1)-N(21)*	*89.05(7)*	C(16)-O(17)-C(18)	117.55(18)
N(11)-C(15)	1.312(3)	*O(3)-Ni(1)-N(21)*	*89.94(7)*	O(17)-C(18)-C(19)	109.7(2)
N(11)-N(12)	1.385(3)	O(16)-Ni(1)-N(21)	90.79(7)	C(26)-N(21)-C(22)	117.9(2)
N(12)-C(13)	1.353(3)	O(6)-Ni(1)-N(21)	89.02(7)	C(26)-N(21)-Ni(1)	120.69(16)
C(13)-O(13)	*1.282(3)*	N(31)-Ni(1)-N(21)	178.21(8)	C(22)-N(21)-Ni(1)	121.42(17)
C(13)-C(14)	1.415(3)	C(5)-N(1)-N(2)	104.50(19)	N(21)-C(22)-C(23)	121.7(2)
C(14)-C(16)	1.415(3)	C(3)-N(2)-N(1)	112.94(18)	C(24)-C(23)-C(22)	120.0(3)
C(14)-C(15)	1.419(3)	O(3)-C(3)-N(2)	123.45(19)	C(23)-C(24)-C(25)	119.1(3)
C(16)-O(16)	*1.246(3)*	O(3)-C(3)-C(4)	131.3(2)	C(24)-C(25)-C(26)	117.9(3)
C(16)-O(17)	1.338(3)	N(2)-C(3)-C(4)	105.26(19)	N(21)-C(26)-C(25)	123.4(2)
O(17)-C(18)	1.450(3)	C(3)-O(3)-Ni(1)	117.55(13)	C(36)-N(31)-C(32)	118.2(2)
C(18)-C(19)	1.514(5)	C(3)-C(4)-C(6)	125.8(2)	C(36)-N(31)-Ni(1)	119.67(19)
N(21)-C(26)	1.335(3)	C(3)-C(4)-C(5)	104.7(2)	C(32)-N(31)-Ni(1)	122.08(18)
N(21)-C(22)	1.343(3)	C(6)-C(4)-C(5)	128.7(2)	N(31)-C(32)-C(33)	122.3(3)
C(22)-C(23)	1.385(4)	N(1)-C(5)-C(4)	112.5(2)	C(34)-C(33)-C(32)	118.9(3)
C(23)-C(24)	1.363(5)	O(6)-C(6)-O(7)	120.5(2)	C(33)-C(34)-C(35)	119.2(3)
C(24)-C(25)	1.381(4)	O(6)-C(6)-C(4)	125.6(2)	C(34)-C(35)-C(36)	119.0(3)
C(25)-C(26)	1.389(4)	O(7)-C(6)-C(4)	113.82(19)	N(31)-C(36)-C(35)	122.4(3)
N(31)-C(36)	1.337(4)	C(6)-O(6)-Ni(1)	123.45(14)		

Table S33. Bond lengths [Å] and angles [°] for **61**.

La(1)-O(3)	*2.401(12)*	*C(15)-O(15)*	*1.28(3)*	O(3)#2-La(1)-O(15')#3	66.2(8)	C(3)-O(3)-La(1)	131.4(10)
La(1)-O(3)#1	*2.402(12)*	C(15)-O(16)	1.28(2)	O(3)#3-La(1)-O(15')#3	68.1(9)	C(3)-C(4)-C(5)	104.6(17)
La(1)-O(3)#2	*2.402(12)*	O(16)-C(17)	1.47(2)	O(15)-La(1)-O(15')#3	72.6(14)	C(3)-C(4)-C(15')	126(2)
La(1)-O(3)#3	*2.402(12)*	C(17)-C(18)	1.50(2)	O(15)#3-La(1)-O(15')#3	15.1(8)	C(5)-C(4)-C(15')	124(2)
La(1)-O(15)	*2.546(17)*	C(15')-O(15')	1.26(3)	O(15)#2-La(1)-O(15')#3	122.3(14)	C(3)-C(4)-C(15)	125.0(16)
La(1)-O(15)#3	*2.546(17)*	C(15')-O(16')	1.28(3)	O(15)#1-La(1)-O(15')#3	136.9(8)	C(5)-C(4)-C(15)	129.7(17)
La(1)-O(15)#2	*2.546(17)*	O(16')-C(17')	1.46(2)	O(15')-La(1)-O(15')#3	86.3(16)	N(1)-C(5)-C(4)	110.7(14)
La(1)-O(15)#1	*2.546(17)*	C(17')-C(18')	1.50(2)	O(3)-La(1)-O(15')#2	148.0(8)	N(2)-C(6)-C(7)	108.4(14)
La(1)-O(15')	2.71(4)			O(3)#1-La(1)-O(15')#2	86.0(9)	N(2)-C(6)-C(7')	97.2(14)
La(1)-O(15')#3	2.71(4)	O(3)-La(1)-O(3)#1	97.6(6)	O(3)#2-La(1)-O(15')#2	68.1(9)	C(8)-C(7)-C(12)	120.0
La(1)-O(15')#2	2.71(4)	O(3)-La(1)-O(3)#2	93.1(6)	O(3)#3-La(1)-O(15')#2	66.2(8)	C(8)-C(7)-C(6)	120.5(10)
La(1)-O(15')#1	2.71(4)	O(3)#1-La(1)-O(3)#2	144.6(5)	O(15)-La(1)-O(15')#2	136.9(8)	C(12)-C(7)-C(6)	119.5(10)
Na(1)-N(1)	*2.357(19)*	O(3)-La(1)-O(3)#3	144.6(5)	O(15)#3-La(1)-O(15')#2	122.3(14)	C(9)-C(8)-C(7)	120.0
Na(1)-N(1)#4	*2.357(19)*	O(3)#1-La(1)-O(3)#3	93.1(6)	O(15)#2-La(1)-O(15')#2	15.1(8)	C(8)-C(9)-C(10)	120.0
Na(1)-N(1)#5	*2.357(19)*	O(3)#2-La(1)-O(3)#3	97.6(6)	O(15)#1-La(1)-O(15')#2	72.6(14)	O(13)-C(10)-C(11)	120.1(10)
Na(1)-N(1)#6	*2.357(19)*	*O(3)-La(1)-O(15)*	*74.4(6)*	O(15')-La(1)-O(15')#2	139.6(16)	O(13)-C(10)-C(9)	119.9(10)
N(1)-C(5)	1.27(3)	O(3)#1-La(1)-O(15)	77.8(9)	O(15')#3-La(1)-O(15')#2	107.7(17)	C(11)-C(10)-C(9)	120.0
N(1)-N(2)	1.36(2)	O(3)#2-La(1)-O(15)	137.6(8)	O(3)-La(1)-O(15')#1	66.2(8)	C(10)-C(11)-C(12)	120.0
N(2)-C(3)	1.29(2)	O(3)#3-La(1)-O(15)	75.1(7)	O(3)#1-La(1)-O(15')#1	68.1(9)	C(11)-C(12)-C(7)	120.0
N(2)-C(6)	1.43(2)	O(3)-La(1)-O(15)#3	75.1(7)	O(3)#2-La(1)-O(15')#1	86.0(9)	C(10)-O(13)-C(14)	132(3)
C(3)-O(3)	*1.319(18)*	O(3)#1-La(1)-O(15)#3	137.6(8)	O(3)#3-La(1)-O(15')#1	148.0(8)	C(8')-C(7')-C(12')	120.0
C(3)-C(4)	1.39(2)	O(3)#2-La(1)-O(15)#3	77.8(9)	O(15)-La(1)-O(15')#1	122.3(14)	C(8')-C(7')-C(6)	120.1(10)
C(4)-C(5)	1.40(2)	*O(3)#3-La(1)-O(15)#3*	*74.4(6)*	O(15)#3-La(1)-O(15')#1	136.9(8)	C(12')-C(7')-C(6)	119.9(10)
C(4)-C(15')	1.46(3)	O(15)-La(1)-O(15)#3	60.0(17)	O(15)#2-La(1)-O(15')#1	72.6(14)	C(7')-C(8')-C(9')	120.0
C(4)-C(15)	1.47(3)	O(3)-La(1)-O(15)#2	137.6(8)	O(15)#1-La(1)-O(15')#1	15.1(8)	C(8')-C(9')-C(10')	120.0
C(6)-C(7)	1.59(3)	O(3)#1-La(1)-O(15)#2	75.1(7)	O(15')-La(1)-O(15')#1	107.7(17)	O(13')-C(10')-C(9')	120.5(11)
C(6)-C(7')	1.63(3)	*O(3)#2-La(1)-O(15)#2*	*74.4(6)*	O(15')#3-La(1)-O(15')#1	139.6(16)	O(13')-C(10')-C(11')	119.5(11)
C(7)-C(8)	1.3900	O(3)#3-La(1)-O(15)#2	77.8(9)	O(15')#2-La(1)-O(15')#1	86.3(16)	C(9')-C(10')-C(11')	120.0
C(7)-C(12)	1.3900	O(15)-La(1)-O(15)#2	140.1(12)	*N(1)-Na(1)-N(1)#4*	*90.9(8)*	C(12')-C(11')-C(10')	120.0
C(8)-C(9)	1.3900	O(15)#3-La(1)-O(15)#2	137.1(15)	*N(1)-Na(1)-N(1)#5*	*119.5(5)*	C(11')-C(12')-C(7')	120.0
C(9)-C(10)	1.3900	O(3)-La(1)-O(15)#1	77.8(9)	*N(1)#4-Na(1)-N(1)#5*	*119.5(5)*	C(10')-O(13')-C(14')	135(4)
C(10)-O(13)	1.28(3)	*O(3)#1-La(1)-O(15)#1*	*74.4(6)*	*N(1)-Na(1)-N(1)#6*	*119.5(5)*	O(15)-C(15)-O(16)	123(2)
C(10)-C(11)	1.3900	O(3)#2-La(1)-O(15)#1	75.1(7)	*N(1)#4-Na(1)-N(1)#6*	*119.5(5)*	O(15)-C(15)-C(4)	124.9(19)
C(11)-C(12)	1.3900	O(3)#3-La(1)-O(15)#1	137.6(8)	*N(1)#5-Na(1)-N(1)#6*	*90.9(8)*	O(16)-C(15)-C(4)	112(2)
O(13)-C(14)	1.37(4)	O(15)-La(1)-O(15)#1	137.1(15)	C(5)-N(1)-N(2)	106.0(16)	C(15)-O(15)-La(1)	130.4(18)
C(7')-C(8')	1.3900	O(15)#3-La(1)-O(15)#1	140.1(12)	C(5)-N(1)-Na(1)	127.5(12)	C(15)-O(16)-C(17)	117(3)
C(7')-C(12')	1.3900	O(15)#2-La(1)-O(15)#1	60.0(17)	N(2)-N(1)-Na(1)	126.2(14)	O(16)-C(17)-C(18)	115(3)
C(8')-C(9')	1.3900	O(3)-La(1)-O(15')	68.1(9)	C(3)-N(2)-N(1)	112.7(14)	O(15')-C(15')-O(16')	119(3)
C(9')-C(10')	1.3900	O(3)#1-La(1)-O(15')	66.2(8)	C(3)-N(2)-C(6)	123.6(15)	O(15')-C(15')-C(4)	116(3)
C(10')-O(13')	1.29(3)	O(3)#2-La(1)-O(15')	148.0(8)	N(1)-N(2)-C(6)	123.6(17)	O(16')-C(15')-C(4)	124(3)
C(10')-C(11')	1.3900	O(3)#3-La(1)-O(15')	86.0(9)	N(2)-C(3)-O(3)	124.6(15)	C(15')-O(15')-La(1)	135(3)
C(11')-C(12')	1.3900	O(3)-La(1)-O(15')#3	86.0(9)	N(2)-C(3)-C(4)	105.9(14)	C(15')-O(16')-C(17')	118(4)
O(13')-C(14')	1.37(4)	O(3)#1-La(1)-O(15')#3	148.0(8)	O(3)-C(3)-C(4)	129.4(17)	O(16')-C(17')-C(18')	114(3)

Table S34. Bond lengths [Å] and angles [°] for **62**.

La(1)-O(23)	*2.4117(14)*	C(34)-C(35)	1.496(3)	O(12)-La(1)-O(60)	143.13(5)
La(1)-O(43)	*2.4673(14)*	N(41)-C(45)	1.313(3)	O(52)-La(1)-O(60)	81.70(5)
La(1)-O(3)	*2.4734(14)*	N(41)-N(42)	1.390(2)	*O(23)-La(1)-O(32)*	*73.23(5)*
La(1)-O(70)	*2.4910(14)*	N(42)-C(43)	1.385(3)	O(43)-La(1)-O(32)	71.14(5)
La(1)-O(12)	*2.5234(15)*	N(42)-C(46)	1.424(3)	O(3)-La(1)-O(32)	133.16(5)
La(1)-O(52)	*2.5437(15)*	*C(43)-O(43)*	*1.266(2)*	O(70)-La(1)-O(32)	75.31(5)
La(1)-O(60)	*2.5609(16)*	C(43)-C(44)	1.409(3)	O(12)-La(1)-O(32)	143.43(5)
La(1)-O(32)	*2.5803(14)*	C(44)-C(45)	1.402(3)	O(52)-La(1)-O(32)	132.04(5)
N(1)-C(5)	1.310(3)	C(44)-C(52)	1.426(3)	O(60)-La(1)-O(32)	71.77(5)
N(1)-N(2)	1.396(3)	C(46)-C(51)	1.378(3)	C(5)-N(1)-N(2)	105.09(17)
N(2)-C(3)	1.388(3)	C(46)-C(47)	1.389(3)	C(3)-N(2)-N(1)	111.63(18)
N(2)-C(6)	1.416(3)	C(47)-C(48)	1.382(3)	C(3)-N(2)-C(6)	128.97(19)
C(3)-O(3)	*1.264(3)*	C(48)-C(49)	1.369(4)	N(1)-N(2)-C(6)	118.97(17)
C(3)-C(4)	1.412(3)	C(49)-C(50)	1.376(4)	O(3)-C(3)-N(2)	124.4(2)
C(4)-C(5)	1.407(3)	C(50)-C(51)	1.385(3)	O(3)-C(3)-C(4)	130.83(19)
C(4)-C(12)	1.423(3)	*C(52)-O(52)*	*1.229(3)*	N(2)-C(3)-C(4)	104.75(18)
C(6)-C(11)	1.372(3)	C(52)-O(53)	1.336(2)	C(3)-O(3)-La(1)	126.94(13)
C(6)-C(7)	1.394(3)	O(53)-C(54)	1.452(3)	C(5)-C(4)-C(3)	105.79(19)
C(7)-C(8)	1.380(4)	C(54)-C(55)	1.503(4)	C(5)-C(4)-C(12)	130.3(2)
C(8)-C(9)	1.379(4)	O(60)-C(61')	1.319(8)	C(3)-C(4)-C(12)	123.90(19)
C(9)-C(10)	1.371(4)	O(60)-C(61)	1.517(5)	N(1)-C(5)-C(4)	112.7(2)
C(10)-C(11)	1.387(4)	C(61)-C(62)	1.449(6)	C(11)-C(6)-C(7)	119.4(2)
C(12)-O(12)	*1.238(2)*	C(61')-C(62')	1.509(12)	C(11)-C(6)-N(2)	121.7(2)
C(12)-O(13)	1.336(3)			C(7)-C(6)-N(2)	118.9(2)
O(13)-C(14)	1.452(3)	O(23)-La(1)-O(43)	78.04(5)	C(8)-C(7)-C(6)	119.9(3)
C(14)-C(15)	1.491(4)	O(23)-La(1)-O(3)	135.14(5)	C(9)-C(8)-C(7)	120.9(2)
N(21)-C(25)	1.309(3)	O(43)-La(1)-O(3)	138.68(5)	C(10)-C(9)-C(8)	118.7(3)
N(21)-N(22)	1.391(2)	O(23)-La(1)-O(70)	81.34(5)	C(9)-C(10)-C(11)	121.3(3)
N(22)-C(23)	1.380(3)	O(43)-La(1)-O(70)	144.42(4)	C(6)-C(11)-C(10)	119.8(2)
N(22)-C(26)	1.418(3)	O(3)-La(1)-O(70)	74.80(5)	O(12)-C(12)-O(13)	121.3(2)
C(23)-O(23)	*1.273(2)*	O(23)-La(1)-O(12)	71.26(5)	O(12)-C(12)-C(4)	124.6(2)
C(23)-C(24)	1.408(3)	O(43)-La(1)-O(12)	109.17(5)	O(13)-C(12)-C(4)	114.10(18)
C(24)-C(25)	1.406(3)	*O(3)-La(1)-O(12)*	*71.73(5)*	C(12)-O(12)-La(1)	133.23(14)
C(24)-C(32)	1.421(3)	O(70)-La(1)-O(12)	90.90(5)	C(12)-O(13)-C(14)	115.96(17)
C(26)-C(31)	1.384(3)	O(23)-La(1)-O(52)	123.30(5)	O(13)-C(14)-C(15)	107.3(2)
C(26)-C(27)	1.389(3)	*O(43)-La(1)-O(52)*	*69.94(5)*	C(25)-N(21)-N(22)	105.54(16)
C(27)-C(28)	1.380(3)	O(3)-La(1)-O(52)	70.30(5)	C(23)-N(22)-N(21)	111.05(17)
C(28)-C(29)	1.380(4)	O(70)-La(1)-O(52)	145.06(4)	C(23)-N(22)-C(26)	129.59(17)
C(29)-C(30)	1.371(4)	O(12)-La(1)-O(52)	76.77(5)	N(21)-N(22)-C(26)	119.35(16)
C(30)-C(31)	1.382(3)	O(23)-La(1)-O(60)	145.00(5)	O(23)-C(23)-N(22)	124.01(19)
C(32)-O(32)	*1.234(2)*	O(43)-La(1)-O(60)	90.61(5)	O(23)-C(23)-C(24)	130.42(19)
C(32)-O(33)	1.343(2)	O(3)-La(1)-O(60)	72.98(5)	N(22)-C(23)-C(24)	105.57(17)
O(33)-C(34)	1.447(3)	*O(70)-La(1)-O(60)*	*89.93(5)*	C(23)-O(23)-La(1)	131.91(13)

Table S34. Part 2.

C(25)-C(24)-C(23)	105.35(19)	N(42)-C(43)-C(44)	105.27(18)
C(25)-C(24)-C(32)	129.17(19)	C(43)-O(43)-La(1)	134.41(13)
C(23)-C(24)-C(32)	125.47(18)	C(45)-C(44)-C(43)	105.61(18)
N(21)-C(25)-C(24)	112.47(19)	C(45)-C(44)-C(52)	131.2(2)
C(31)-C(26)-C(27)	119.3(2)	C(43)-C(44)-C(52)	123.15(19)
C(31)-C(26)-N(22)	121.59(19)	N(41)-C(45)-C(44)	112.5(2)
C(27)-C(26)-N(22)	119.08(19)	C(51)-C(46)-C(47)	119.1(2)
C(28)-C(27)-C(26)	120.3(2)	C(51)-C(46)-N(42)	121.22(19)
C(29)-C(28)-C(27)	120.5(2)	C(47)-C(46)-N(42)	119.7(2)
C(30)-C(29)-C(28)	118.8(2)	C(48)-C(47)-C(46)	120.1(2)
C(29)-C(30)-C(31)	121.8(2)	C(49)-C(48)-C(47)	120.8(2)
C(30)-C(31)-C(26)	119.3(2)	C(48)-C(49)-C(50)	119.3(2)
O(32)-C(32)-O(33)	121.6(2)	C(49)-C(50)-C(51)	120.6(3)
O(32)-C(32)-C(24)	125.33(19)	C(46)-C(51)-C(50)	120.2(2)
O(33)-C(32)-C(24)	113.01(17)	O(52)-C(52)-O(53)	121.11(19)
C(32)-O(32)-La(1)	131.48(14)	O(52)-C(52)-C(44)	124.19(19)
C(32)-O(33)-C(34)	117.18(17)	O(53)-C(52)-C(44)	114.70(19)
O(33)-C(34)-C(35)	107.37(19)	C(52)-O(52)-La(1)	138.17(13)
C(45)-N(41)-N(42)	105.44(17)	C(52)-O(53)-C(54)	116.29(18)
C(43)-N(42)-N(41)	111.12(16)	O(53)-C(54)-C(55)	106.8(2)
C(43)-N(42)-C(46)	129.38(18)	C(61')-O(60)-La(1)	129.6(4)
N(41)-N(42)-C(46)	119.42(17)	C(61)-O(60)-La(1)	132.3(2)
O(43)-C(43)-N(42)	124.62(18)	C(62)-C(61)-O(60)	112.0(4)
O(43)-C(43)-C(44)	130.11(19)	O(60)-C(61')-C(62')	106.1(8)

Table S35. Bond lengths [Å] and angles [°] for **65**.

Dy(1)-O(3)	*2.289(7)*	O(16')-C(17')	1.464(18)	O(3)-C(3)-N(2)	124.1(9)
Dy(1)-O(3)#1	*2.289(7)*	C(17')-C(18')	1.53(2)	O(3)-C(3)-C(4)	131.8(9)
Dy(1)-O(3)#2	*2.289(7)*			N(2)-C(3)-C(4)	104.1(8)
Dy(1)-O(3)#3	*2.289(7)*	O(3)-Dy(1)-O(3)#1	102.3(4)	C(3)-O(3)-Dy(1)	129.3(6)
Dy(1)-O(15)#3	*2.455(6)*	O(3)-Dy(1)-O(3)#2	86.7(3)	C(5)-C(4)-C(15)	133.0(9)
Dy(1)-O(15)#1	*2.455(6)*	O(3)#1-Dy(1)-O(3)#2	147.6(3)	C(5)-C(4)-C(3)	105.1(8)
Dy(1)-O(15)#2	*2.455(6)*	O(3)-Dy(1)-O(3)#3	147.6(3)	C(15)-C(4)-C(3)	121.9(9)
Dy(1)-O(15)	*2.455(6)*	O(3)#1-Dy(1)-O(3)#3	86.7(3)	N(1)-C(5)-C(4)	113.4(9)
Na(1)-N(1)#4	*2.425(9)*	O(3)#2-Dy(1)-O(3)#3	102.3(4)	N(2)-C(6)-C(7')	109.6(10)
Na(1)-N(1)#5	*2.425(9)*	O(3)-Dy(1)-O(15)#3	77.4(2)	N(2)-C(6)-C(7)	106.5(7)
Na(1)-N(1)#6	*2.425(9)*	O(3)#1-Dy(1)-O(15)#3	140.3(2)	C(8)-C(7)-C(12)	120.0
Na(1)-N(1)	*2.425(9)*	O(3)#2-Dy(1)-O(15)#3	71.9(3)	C(8)-C(7)-C(6)	120.1(7)
N(1)-C(5)	1.328(13)	*O(3)#3-Dy(1)-O(15)#3*	*76.1(2)*	C(12)-C(7)-C(6)	119.9(7)
N(1)-N(2)	1.383(12)	O(3)-Dy(1)-O(15)#1	71.9(3)	C(9)-C(8)-C(7)	120.0
N(2)-C(3)	1.348(13)	*O(3)#1-Dy(1)-O(15)#1*	*76.1(2)*	C(8)-C(9)-C(10)	120.0
N(2)-C(6)	1.444(12)	O(3)#2-Dy(1)-O(15)#1	77.4(2)	O(13)-C(10)-C(11)	118.8(7)
C(3)-O(3)	*1.275(11)*	O(3)#3-Dy(1)-O(15)#1	140.3(2)	O(13)-C(10)-C(9)	121.2(7)
C(3)-C(4)	1.430(14)	O(15)#3-Dy(1)-O(15)#1	137.3(3)	C(11)-C(10)-C(9)	120.0
C(4)-C(5)	1.392(13)	O(3)-Dy(1)-O(15)#2	140.3(2)	C(10)-C(11)-C(12)	120.0
C(4)-C(15)	1.423(14)	O(3)#1-Dy(1)-O(15)#2	77.4(2)	C(11)-C(12)-C(7)	120.0
C(6)-C(7')	1.59(2)	*O(3)#2-Dy(1)-O(15)#2*	*76.1(2)*	C(10)-O(13)-C(14)	120.6(16)
C(6)-C(7)	1.598(15)	O(3)#3-Dy(1)-O(15)#2	71.9(3)	C(8')-C(7')-C(12')	120.0
C(7)-C(8)	1.3900	O(15)#3-Dy(1)-O(15)#2	127.9(4)	C(8')-C(7')-C(6)	120.1(10)
C(7)-C(12)	1.3900	O(15)#1-Dy(1)-O(15)#2	69.6(4)	C(12')-C(7')-C(6)	119.9(10)
C(8)-C(9)	1.3900	*O(3)-Dy(1)-O(15)*	*76.1(2)*	C(7')-C(8')-C(9')	120.0
C(9)-C(10)	1.3900	O(3)#1-Dy(1)-O(15)	71.9(3)	C(8')-C(9')-C(10')	120.0
C(10)-O(13)	1.361(15)	O(3)#2-Dy(1)-O(15)	140.3(2)	O(13')-C(10')-C(11')	120.2(10)
C(10)-C(11)	1.3900	O(3)#3-Dy(1)-O(15)	77.4(2)	O(13')-C(10')-C(9')	119.8(10)
C(11)-C(12)	1.3900	O(15)#3-Dy(1)-O(15)	69.6(4)	C(11')-C(10')-C(9')	120.0
O(13)-C(14)	1.38(2)	O(15)#1-Dy(1)-O(15)	127.9(4)	C(10')-C(11')-C(12')	120.0
C(7')-C(8')	1.3900	O(15)#2-Dy(1)-O(15)	137.3(3)	C(11')-C(12')-C(7')	120.0
C(7')-C(12')	1.3900	*N(1)#4-Na(1)-N(1)#5*	*120.5(2)*	C(10')-O(13')-C(14')	116(3)
C(8')-C(9')	1.3900	*N(1)#4-Na(1)-N(1)#6*	*120.5(2)*	O(15)-C(15)-O(16)	121.3(11)
C(9')-C(10')	1.3900	*N(1)#5-Na(1)-N(1)#6*	*89.1(4)*	O(15)-C(15)-O(16')	118.8(18)
C(10')-O(13')	1.36(2)	*N(1)#4-Na(1)-N(1)*	*89.1(4)*	O(15)-C(15)-C(4)	126.1(9)
C(10')-C(11')	1.3900	*N(1)#5-Na(1)-N(1)*	*120.5(2)*	O(16)-C(15)-C(4)	112.2(11)
C(11')-C(12')	1.3900	*N(1)#6-Na(1)-N(1)*	*120.5(2)*	O(16')-C(15)-C(4)	112.6(17)
O(13')-C(14')	1.37(3)	C(5)-N(1)-N(2)	103.0(8)	C(15)-O(15)-Dy(1)	131.1(6)
C(15)-O(15)	*1.233(11)*	C(5)-N(1)-Na(1)	129.0(7)	C(15)-O(16)-C(17)	115.7(18)
C(15)-O(16)	1.346(12)	N(2)-N(1)-Na(1)	127.5(6)	O(16)-C(17)-C(18)	111(3)
C(15)-O(16')	1.36(2)	C(3)-N(2)-N(1)	114.5(8)	C(15)-O(16')-C(17')	121(5)
O(16)-C(17)	1.471(15)	C(3)-N(2)-C(6)	124.8(9)	O(16')-C(17')-C(18')	113(4)
C(17)-C(18)	1.54(2)	N(1)-N(2)-C(6)	120.1(9)		

Table S36. Bond lengths [Å] and angles [°] for **66**.

Dy(1)-O(3)	*2.253(3)*	C(34)-C(35)	1.484(7)	O(43)-Dy(1)-O(12)	71.76(11)
Dy(1)-O(43)	*2.331(3)*	N(41)-C(45)	1.303(6)	O(70)-Dy(1)-O(12)	74.78(9)
Dy(1)-O(70)	*2.358(3)*	N(41)-N(42)	1.393(5)	O(23)-Dy(1)-O(12)	124.14(11)
Dy(1)-O(23)	*2.374(3)*	N(42)-C(43)	1.384(6)	O(60)-Dy(1)-O(12)	72.05(10)
Dy(1)-O(60)	*2.390(3)*	N(42)-C(46)	1.385(6)	O(32)-Dy(1)-O(12)	143.69(10)
Dy(1)-O(32)	*2.392(3)*	N(42)-C(46')	1.450(13)	O(3)-Dy(1)-O(52)	115.86(11)
Dy(1)-O(12)	*2.433(3)*	*C(43)-O(43)*	*1.267(5)*	*O(43)-Dy(1)-O(52)*	*73.24(11)*
Dy(1)-O(52)	*2.436(3)*	C(43)-C(44)	1.404(6)	O(70)-Dy(1)-O(52)	142.24(10)
N(1)-C(5)	1.296(6)	C(44)-C(45)	1.395(6)	O(23)-Dy(1)-O(52)	71.36(11)
N(1)-N(2)	1.386(5)	C(44)-C(52)	1.420(6)	O(60)-Dy(1)-O(52)	84.44(11)
N(2)-C(3)	1.376(5)	C(46)-C(47)	1.3900	O(32)-Dy(1)-O(52)	72.42(10)
N(2)-C(6)	1.419(6)	C(46)-C(51)	1.3900	O(12)-Dy(1)-O(52)	139.70(10)
C(3)-O(3)	*1.288(5)*	C(47)-C(48)	1.3900	C(5)-N(1)-N(2)	106.4(4)
C(3)-C(4)	1.401(6)	C(48)-C(49)	1.3900	C(3)-N(2)-N(1)	110.2(4)
C(4)-C(5)	1.412(6)	C(49)-C(50)	1.3900	C(3)-N(2)-C(6)	128.9(4)
C(4)-C(12)	1.414(6)	C(50)-C(51)	1.3900	N(1)-N(2)-C(6)	120.9(4)
C(6)-C(11)	1.364(6)	C(46')-C(47')	1.3900	O(3)-C(3)-N(2)	123.3(4)
C(6)-C(7)	1.396(6)	C(46')-C(51')	1.3900	O(3)-C(3)-C(4)	130.4(4)
C(7)-C(8)	1.371(7)	C(47')-C(48')	1.3900	N(2)-C(3)-C(4)	106.4(4)
C(8)-C(9)	1.373(8)	C(48')-C(49')	1.3900	C(3)-O(3)-Dy(1)	131.0(3)
C(9)-C(10)	1.366(7)	C(49')-C(50')	1.3900	C(3)-C(4)-C(5)	104.7(4)
C(10)-C(11)	1.372(7)	C(50')-C(51')	1.3900	C(3)-C(4)-C(12)	123.5(4)
C(12)-O(12)	*1.233(5)*	*C(52)-O(52)*	*1.238(5)*	C(5)-C(4)-C(12)	131.7(4)
C(12)-O(13)	1.347(5)	C(52)-O(53)	1.328(5)	N(1)-C(5)-C(4)	112.3(4)
O(13)-C(14)	1.456(5)	O(53)-C(54)	1.455(6)	C(11)-C(6)-C(7)	119.3(5)
C(14)-C(15)	1.457(8)	C(54)-C(55)	1.496(8)	C(11)-C(6)-N(2)	122.3(4)
N(21)-C(25)	1.302(6)			C(7)-C(6)-N(2)	118.4(4)
N(21)-N(22)	1.393(5)	O(3)-Dy(1)-O(43)	79.57(10)	C(8)-C(7)-C(6)	119.5(5)
N(22)-C(23)	1.385(6)	O(3)-Dy(1)-O(70)	81.54(10)	C(7)-C(8)-C(9)	120.9(5)
N(22)-C(26)	1.408(6)	O(43)-Dy(1)-O(70)	144.52(10)	C(10)-C(9)-C(8)	118.9(5)
C(23)-O(23)	*1.259(5)*	O(3)-Dy(1)-O(23)	141.58(10)	C(9)-C(10)-C(11)	121.1(6)
C(23)-C(24)	1.418(7)	O(43)-Dy(1)-O(23)	135.03(10)	C(6)-C(11)-C(10)	120.2(5)
C(24)-C(25)	1.397(6)	O(70)-Dy(1)-O(23)	74.96(9)	O(12)-C(12)-O(13)	121.9(4)
C(24)-C(32)	1.418(7)	O(3)-Dy(1)-O(60)	147.42(10)	O(12)-C(12)-C(4)	125.0(4)
C(26)-C(27)	1.376(7)	O(43)-Dy(1)-O(60)	82.86(10)	O(13)-C(12)-C(4)	113.1(4)
C(26)-C(31)	1.380(7)	*O(70)-Dy(1)-O(60)*	*97.85(10)*	C(12)-O(12)-Dy(1)	130.7(3)
C(27)-C(28)	1.381(8)	O(23)-Dy(1)-O(60)	67.03(9)	C(12)-O(13)-C(14)	117.8(4)
C(28)-C(29)	1.381(8)	O(3)-Dy(1)-O(32)	72.10(10)	O(13)-C(14)-C(15)	108.1(5)
C(29)-C(30)	1.364(8)	O(43)-Dy(1)-O(32)	118.64(11)	C(25)-N(21)-N(22)	105.5(4)
C(30)-C(31)	1.383(7)	O(70)-Dy(1)-O(32)	82.98(10)	C(23)-N(22)-N(21)	111.3(4)
C(32)-O(32)	*1.242(5)*	*O(23)-Dy(1)-O(32)*	*75.17(10)*	C(23)-N(22)-C(26)	129.4(4)
C(32)-O(33)	1.332(5)	O(60)-Dy(1)-O(32)	140.40(10)	N(21)-N(22)-C(26)	119.1(4)
O(33)-C(34)	1.459(6)	*O(3)-Dy(1)-O(12)*	*76.47(10)*	O(23)-C(23)-N(22)	124.3(4)

Table S36. Part 2.

O(23)-C(23)-C(24)	131.0(4)	N(42)-C(43)-C(44)	105.2(4)
N(22)-C(23)-C(24)	104.6(4)	C(43)-O(43)-Dy(1)	133.0(3)
C(23)-O(23)-Dy(1)	127.5(3)	C(45)-C(44)-C(43)	105.8(4)
C(25)-C(24)-C(23)	105.7(4)	C(45)-C(44)-C(52)	131.5(5)
C(25)-C(24)-C(32)	131.0(5)	C(43)-C(44)-C(52)	122.6(4)
C(23)-C(24)-C(32)	123.2(4)	N(41)-C(45)-C(44)	112.6(4)
N(21)-C(25)-C(24)	112.8(4)	N(42)-C(46)-C(47)	121.1(4)
C(27)-C(26)-C(31)	119.0(5)	N(42)-C(46)-C(51)	118.8(4)
C(27)-C(26)-N(22)	119.5(5)	C(47)-C(46)-C(51)	120.0
C(31)-C(26)-N(22)	121.4(4)	C(46)-C(47)-C(48)	120.0
C(26)-C(27)-C(28)	120.5(5)	C(49)-C(48)-C(47)	120.0
C(29)-C(28)-C(27)	120.4(6)	C(48)-C(49)-C(50)	120.0
C(30)-C(29)-C(28)	118.8(6)	C(51)-C(50)-C(49)	120.0
C(29)-C(30)-C(31)	121.2(6)	C(50)-C(51)-C(46)	120.0
C(26)-C(31)-C(30)	119.9(5)	C(47')-C(46')-C(51')	120.0
O(32)-C(32)-O(33)	121.1(4)	C(47')-C(46')-N(42)	117.6(11)
O(32)-C(32)-C(24)	124.7(4)	C(51')-C(46')-N(42)	122.2(11)
O(33)-C(32)-C(24)	114.2(4)	C(48')-C(47')-C(46')	120.0
C(32)-O(32)-Dy(1)	133.1(3)	C(47')-C(48')-C(49')	120.0
C(32)-O(33)-C(34)	116.3(4)	C(48')-C(49')-C(50')	120.0
O(33)-C(34)-C(35)	107.6(4)	C(51')-C(50')-C(49')	120.0
C(45)-N(41)-N(42)	105.6(4)	C(50')-C(51')-C(46')	120.0
C(43)-N(42)-C(46)	130.5(4)	O(52)-C(52)-O(53)	122.3(5)
C(43)-N(42)-N(41)	110.7(4)	O(52)-C(52)-C(44)	123.5(5)
C(46)-N(42)-N(41)	118.5(4)	O(53)-C(52)-C(44)	114.2(4)
C(43)-N(42)-C(46')	126.7(8)	C(52)-O(52)-Dy(1)	136.5(3)
N(41)-N(42)-C(46')	122.6(8)	C(52)-O(53)-C(54)	117.7(4)
O(43)-C(43)-N(42)	124.2(4)	O(53)-C(54)-C(55)	109.0(5)
O(43)-C(43)-C(44)	130.6(4)		

Table S37. Bond lengths [Å] and angles [°] for **70**.

Yb(1)-O(3)	*2.258(2)*	N(41)-C(45)	1.308(5)	O(23)-Yb(1)-O(52)	72.76(8)	N(21)-C(25)-C(24)	112.3(3)
Yb(1)-O(23)	*2.258(2)*	N(41)-N(42)	1.397(4)	*O(43)-Yb(1)-O(52)*	*78.37(8)*	C(31)-C(26)-C(27)	120.1(3)
Yb(1)-O(43)	*2.267(2)*	N(42)-C(43)	1.382(4)	O(60)-Yb(1)-O(52)	147.86(7)	C(31)-C(26)-N(22)	120.2(3)
Yb(1)-O(60)	*2.345(2)*	N(42)-C(46)	1.407(5)	O(70)-Yb(1)-O(52)	73.31(7)	C(27)-C(26)-N(22)	119.8(3)
Yb(1)-O(70)	*2.356(2)*	*C(43)-O(43)*	*1.274(4)*	O(32)-Yb(1)-O(52)	120.91(7)	C(28)-C(27)-C(26)	119.5(4)
Yb(1)-O(32)	*2.3601(19)*	C(43)-C(44)	1.407(5)	O(12)-Yb(1)-O(52)	140.60(8)	C(27)-C(28)-C(29)	120.7(4)
Yb(1)-O(12)	*2.370(2)*	C(44)-C(45)	1.413(5)	C(5)-N(1)-N(2)	104.7(2)	C(30)-C(29)-C(28)	119.7(3)
Yb(1)-O(52)	*2.418(2)*	C(44)-C(52)	1.416(5)	C(3)-N(2)-N(1)	111.5(2)	C(29)-C(30)-C(31)	120.0(4)
N(1)-C(5)	1.300(4)	C(46)-C(51)	1.387(5)	C(3)-N(2)-C(6)	128.5(2)	C(26)-C(31)-C(30)	120.0(3)
N(1)-N(2)	1.399(3)	C(46)-C(47)	1.397(6)	N(1)-N(2)-C(6)	119.9(2)	O(32)-C(32)-O(33)	121.9(3)
N(2)-C(3)	1.376(4)	C(47)-C(48)	1.378(7)	O(3)-C(3)-N(2)	124.1(3)	O(32)-C(32)-C(24)	123.9(3)
N(2)-C(6)	1.422(4)	C(48)-C(49)	1.377(7)	O(3)-C(3)-C(4)	130.5(3)	O(33)-C(32)-C(24)	114.2(3)
C(3)-O(3)	*1.274(4)*	C(49)-C(50)	1.370(7)	N(2)-C(3)-C(4)	105.4(2)	C(32)-O(32)-Yb(1)	135.68(19)
C(3)-C(4)	1.407(4)	C(50)-C(51)	1.385(6)	C(3)-O(3)-Yb(1)	130.82(19)	C(32)-O(33)-C(34)	116.9(3)
C(4)-C(5)	1.408(4)	*C(52)-O(52)*	*1.238(4)*	C(3)-C(4)-C(5)	105.0(2)	O(33)-C(34)-C(35)	110.2(3)
C(4)-C(12)	1.425(4)	C(52)-O(53)	1.361(4)	C(3)-C(4)-C(12)	121.8(3)	C(45)-N(41)-N(42)	104.2(3)
C(6)-C(7)	1.387(4)	O(53)-C(54)	1.448(5)	C(5)-C(4)-C(12)	133.0(3)	C(43)-N(42)-N(41)	112.1(3)
C(6)-C(11)	1.394(4)	C(54)-C(55)	1.505(5)	N(1)-C(5)-C(4)	113.4(3)	C(43)-N(42)-C(46)	127.2(3)
C(7)-C(8)	1.388(5)	O(60)-C(61)	1.421(4)	C(7)-C(6)-C(11)	120.0(3)	N(41)-N(42)-C(46)	119.7(3)
C(8)-C(9)	1.384(5)	O(70)-C(71)	1.435(4)	C(7)-C(6)-N(2)	120.2(3)	O(43)-C(43)-N(42)	122.6(3)
C(9)-C(10)	1.383(5)			C(11)-C(6)-N(2)	119.8(3)	O(43)-C(43)-C(44)	132.2(3)
C(10)-C(11)	1.391(4)	O(3)-Yb(1)-O(23)	142.12(7)	C(6)-C(7)-C(8)	119.8(3)	N(42)-C(43)-C(44)	105.2(3)
C(12)-O(12)	*1.246(4)*	O(3)-Yb(1)-O(43)	76.68(8)	C(9)-C(8)-C(7)	120.7(3)	C(43)-O(43)-Yb(1)	128.7(2)
C(12)-O(13)	1.328(4)	O(23)-Yb(1)-O(43)	77.80(8)	C(10)-C(9)-C(8)	119.3(3)	C(43)-C(44)-C(45)	105.0(3)
O(13)-C(14)	1.459(4)	O(3)-Yb(1)-O(60)	112.37(7)	C(9)-C(10)-C(11)	120.8(3)	C(43)-C(44)-C(52)	124.3(3)
C(14)-C(15)	1.504(5)	O(23)-Yb(1)-O(60)	86.26(7)	C(10)-C(11)-C(6)	119.4(3)	C(45)-C(44)-C(52)	130.7(3)
N(21)-C(25)	1.319(4)	O(43)-Yb(1)-O(60)	73.49(8)	O(12)-C(12)-O(13)	122.0(3)	N(41)-C(45)-C(44)	113.5(3)
N(21)-N(22)	1.399(3)	O(3)-Yb(1)-O(70)	82.03(7)	O(12)-C(12)-C(4)	122.9(3)	C(51)-C(46)-C(47)	119.4(4)
N(22)-C(23)	1.382(4)	O(23)-Yb(1)-O(70)	106.83(7)	O(13)-C(12)-C(4)	115.1(3)	C(51)-C(46)-N(42)	121.2(3)
N(22)-C(26)	1.421(4)	O(43)-Yb(1)-O(70)	148.10(7)	C(12)-O(12)-Yb(1)	136.05(19)	C(47)-C(46)-N(42)	119.4(3)
C(23)-O(23)	*1.271(4)*	*O(60)-Yb(1)-O(70)*	*137.46(7)*	C(12)-O(13)-C(14)	116.3(2)	C(48)-C(47)-C(46)	119.9(4)
C(23)-C(24)	1.411(4)	O(3)-Yb(1)-O(32)	139.76(7)	O(13)-C(14)-C(15)	106.4(3)	C(49)-C(48)-C(47)	120.6(4)
C(24)-C(25)	1.404(4)	*O(23)-Yb(1)-O(32)*	*75.56(7)*	C(25)-N(21)-N(22)	105.2(2)	C(50)-C(49)-C(48)	119.4(4)
C(24)-C(32)	1.423(4)	O(43)-Yb(1)-O(32)	139.44(8)	C(23)-N(22)-N(21)	111.4(2)	C(49)-C(50)-C(51)	121.3(4)
C(26)-C(31)	1.383(5)	O(60)-Yb(1)-O(32)	74.76(7)	C(23)-N(22)-C(26)	129.2(3)	C(50)-C(51)-C(46)	119.3(4)
C(26)-C(27)	1.390(5)	O(70)-Yb(1)-O(32)	70.05(7)	N(21)-N(22)-C(26)	119.4(2)	O(52)-C(52)-O(53)	121.8(3)
C(27)-C(28)	1.381(6)	*O(3)-Yb(1)-O(12)*	*74.97(7)*	O(23)-C(23)-N(22)	124.3(3)	O(52)-C(52)-C(44)	124.8(3)
C(28)-C(29)	1.384(7)	O(23)-Yb(1)-O(12)	142.53(7)	O(23)-C(23)-C(24)	130.5(3)	O(53)-C(52)-C(44)	113.4(3)
C(29)-C(30)	1.381(6)	O(43)-Yb(1)-O(12)	118.20(7)	N(22)-C(23)-C(24)	105.2(2)	C(52)-O(52)-Yb(1)	131.6(2)
C(30)-C(31)	1.391(5)	O(60)-Yb(1)-O(12)	68.74(7)	C(23)-O(23)-Yb(1)	131.77(18)	C(52)-O(53)-C(54)	117.3(3)
C(32)-O(32)	*1.242(4)*	O(70)-Yb(1)-O(12)	77.78(7)	C(25)-C(24)-C(23)	106.0(3)	O(53)-C(54)-C(55)	107.0(3)
C(32)-O(33)	1.334(4)	O(32)-Yb(1)-O(12)	71.28(7)	C(25)-C(24)-C(32)	132.3(3)	C(61)-O(60)-Yb(1)	127.01(19)
O(33)-C(34)	1.456(4)	O(3)-Yb(1)-O(52)	75.01(7)	C(23)-C(24)-C(32)	121.6(3)	C(71)-O(70)-Yb(1)	128.82(17)
C(34)-C(35)	1.500(6)						

Table S38. Bond lengths [Å] and angles [°] for **73**.

U(1)-O(50)	*1.765(16)*	N(21)-C(25)	1.31(3)	O(50)-U(1)-O(3)	88.9(6)	C(9)-C(8)-C(7)	120.0	C(23)-C(24)-C(25)	103.1(18)
U(1)-O(60)	*1.785(17)*	N(21)-N(22)	1.38(2)	O(60)-U(1)-O(3)	91.8(8)	C(8)-C(9)-C(10)	120.0	N(21)-C(25)-C(24)	112(2)
U(1)-O(3)	*2.321(13)*	N(22)-C(23)	1.34(2)	O(50)-U(1)-O(23)	88.8(6)	C(11)-C(10)-C(9)	120.0	N(22)-C(26)-C(27)	119(2)
U(1)-O(23)	*2.361(13)*	N(22)-C(26)	1.44(3)	O(60)-U(1)-O(23)	90.4(7)	C(11)-C(10)-O(13)	121(3)	N(22)-C(26)-C(27')	109(2)
U(1)-O(40)	*2.368(15)*	*C(23)-O(23)*	*1.29(3)*	O(3)-U(1)-O(23)	145.0(5)	C(9)-C(10)-O(13)	119(3)	C(28)-C(27)-C(32)	120.0
U(1)-O(35)	*2.454(13)*	C(23)-C(24)	1.42(3)	*O(50)-U(1)-O(40)*	*89.5(6)*	C(10)-C(11)-C(12)	120.0	C(28)-C(27)-C(26)	120(2)
U(1)-O(15)	*2.458(13)*	C(24)-C(35)	1.41(3)	*O(60)-U(1)-O(40)*	*90.6(7)*	C(10)-C(11)-C(12)	120.0	C(32)-C(27)-C(26)	120(2)
N(1)-C(5)	1.33(3)	C(24)-C(25)	1.43(3)	O(3)-U(1)-O(40)	72.8(5)	C(11)-C(12)-C(7)	120.0	C(29)-C(28)-C(27)	120.0
N(1)-N(2)	1.37(2)	C(26)-C(27)	1.51(3)	O(23)-U(1)-O(40)	72.2(5)	C(14)-O(13)-C(10)	132(5)	C(28)-C(29)-C(30)	120.0
N(2)-C(3)	1.39(2)	C(26)-C(27')	1.57(4)	O(50)-U(1)-O(35)	92.6(6)	C(8')-C(7')-C(12')	120.0	O(33)-C(30)-C(29)	119.1(19)
N(2)-C(6)	1.46(3)	C(27)-C(28)	1.3900	O(60)-U(1)-O(35)	87.0(7)	C(8')-C(7')-C(6)	118(3)	O(33)-C(30)-C(31)	120(2)
C(3)-O(3)	*1.27(2)*	C(27)-C(32)	1.3900	O(3)-U(1)-O(35)	141.2(4)	C(12')-C(7')-C(6)	122(3)	C(29)-C(30)-C(31)	120.0
C(3)-C(4)	1.39(3)	C(28)-C(29)	1.3900	*O(23)-U(1)-O(35)*	*73.8(5)*	C(7')-C(8')-C(9')	120.0	C(32)-C(31)-C(30)	120.0
C(4)-C(5)	1.41(3)	C(29)-C(30)	1.3900	O(40)-U(1)-O(35)	145.9(4)	C(10')-C(9')-C(8')	120.0	C(31)-C(32)-C(27)	120.0
C(4)-C(15)	1.43(3)	C(30)-O(33)	1.33(3)	O(50)-U(1)-O(15)	91.2(6)	C(9')-C(10')-C(11')	120.0	C(30)-O(33)-C(34)	117(3)
C(6)-C(7)	1.50(3)	C(30)-C(31)	1.3900	O(60)-U(1)-O(15)	89.2(7)	C(9')-C(10')-O(13')	119(3)	C(28')-C(27')-C(32')	120.0
C(6)-C(7')	1.52(4)	C(31)-C(32)	1.3900	*O(3)-U(1)-O(15)*	*72.9(5)*	C(11')-C(10')-O(13')	121(3)	C(28')-C(27')-C(26)	119(3)
C(7)-C(8)	1.3900	O(33)-C(34)	1.39(5)	O(23)-U(1)-O(15)	142.0(5)	C(10')-C(11')-C(12')	120.0	C(32')-C(27')-C(26)	120(3)
C(7)-C(12)	1.3900	C(27')-C(28')	1.3900	O(40)-U(1)-O(15)	145.7(4)	C(11')-C(12')-C(7')	120.0	C(27')-C(28')-C(29')	120.0
C(8)-C(9)	1.3900	C(27')-C(32')	1.3900	O(35)-U(1)-O(15)	68.3(4)	C(14')-O(13')-C(10')	138(6)	C(30')-C(29')-C(28')	120.0
C(9)-C(10)	1.3900	C(28')-C(29')	1.3900	C(5)-N(1)-N(2)	106.1(15)	O(15)-C(15)-O(16)	120.8(19)	O(33')-C(30')-C(29')	121(2)
C(10)-C(11)	1.3900	C(29')-C(30')	1.3900	N(1)-N(2)-C(3)	112.0(16)	O(15)-C(15)-C(4)	121.3(18)	O(33')-C(30')-C(31')	119(2)
C(10)-O(13)	1.42(4)	C(30')-O(33')	1.33(3)	N(1)-N(2)-C(6)	120.4(15)	O(16)-C(15)-C(4)	117.9(16)	C(29')-C(30')-C(31')	120.0
C(11)-C(12)	1.3900	C(30')-C(31')	1.3900	C(3)-N(2)-C(6)	127.6(16)	C(15)-O(15)-U(1)	138.1(14)	C(32')-C(31')-C(30')	120.0
O(13)-C(14)	1.07(5)	C(31')-C(32')	1.3900	O(3)-C(3)-C(4)	134.3(18)	C(15)-O(16)-C(17)	119.0(15)	C(31')-C(32')-C(27')	120.0
C(7')-C(8')	1.3900	O(33')-C(34')	1.39(5)	O(3)-C(3)-N(2)	121.5(18)	O(16)-C(17)-C(18)	109.0(19)	C(30')-O(33')-C(34')	115(4)
C(7')-C(12')	1.3900	*C(35)-O(35)*	*1.27(2)*	C(4)-C(3)-N(2)	104.1(17)	C(25)-N(21)-N(22)	105.2(15)	O(35)-C(35)-O(36)	120.1(18)
C(8')-C(9')	1.3900	C(35)-O(36)	1.28(3)	C(3)-O(3)-U(1)	131.6(13)	C(23)-N(22)-N(21)	111.8(18)	O(35)-C(35)-C(24)	122(2)
C(9')-C(10')	1.3900	O(36)-C(37)	1.46(2)	C(3)-C(4)-C(5)	108.1(19)	C(23)-N(22)-C(26)	128.0(18)	O(36)-C(35)-C(24)	117.3(18)
C(10')-C(11')	1.3900	C(37)-C(38)	1.52(3)	C(3)-C(4)-C(15)	121.8(17)	N(21)-N(22)-C(26)	120.1(15)	C(35)-O(35)-U(1)	136.8(13)
C(10')-O(13')	1.42(4)	O(40)-C(41')	1.48(4)	C(5)-C(4)-C(15)	129.9(19)	O(23)-C(23)-N(22)	122(2)	C(35)-O(36)-C(37)	117.3(15)
C(11')-C(12')	1.3900	O(40)-C(41)	1.48(4)	N(1)-C(5)-C(4)	109.8(19)	O(23)-C(23)-C(24)	131.0(19)	O(36)-C(37)-C(38)	108.2(17)
O(13')-C(14')	1.07(5)	C(41)-C(42)	1.59(5)	N(2)-C(6)-C(7)	110.5(19)	N(22)-C(23)-C(24)	107.3(18)	C(41')-O(40)-U(1)	129(2)
C(15)-O(15)	*1.26(2)*	C(41')-C(42')	1.59(5)	N(2)-C(6)-C(7')	115(2)	C(23)-O(23)-U(1)	131.6(13)	C(41)-O(40)-U(1)	132(2)
C(15)-O(16)	1.32(2)			C(8)-C(7)-C(12)	120.0	C(23)-O(23)-U(1)	131.6(13)	O(40)-C(41)-C(42)	103(2)
O(16)-C(17)	1.46(2)	*O(50)-U(1)-O(60)*	*179.2(9)*	C(8)-C(7)-C(6)	118.6(18)	C(35)-C(24)-C(23)	124.3(19)	O(40)-C(41')-C(42')	103(2)
C(17)-C(18)	1.51(3)	O(50)-U(1)-O(3)	88.9(6)	C(12)-C(7)-C(6)	121.2(18)	C(35)-C(24)-C(25)	132(2)		

Table S39. Bond lengths [Å] and angles [°] for **74**.

U(1)-O(50)	*1.731(10)*	C(24)-C(25)	1.41(2)	O(23)-U(1)-N(41)	69.8(4)	C(12')-O(13')-C(14')	118.7(18)
U(1)-O(60)	*1.736(11)*	C(24)-C(32)	1.438(16)	O(3)-U(1)-N(41)	140.0(3)	O(13')-C(14')-C(15')	109.5(17)
U(1)-O(23)	*2.297(9)*	C(26)-C(31)	1.378(19)	O(12)-U(1)-N(41)	67.8(3)	C(25)-N(21)-N(22)	104.2(11)
U(1)-O(3)	*2.335(7)*	C(26)-C(27)	1.383(16)	O(32)-U(1)-N(41)	142.8(3)	C(23)-N(22)-N(21)	112.3(10)
U(1)-O(12)	*2.430(10)*	C(27)-C(28)	1.400(17)	C(5)-N(1)-N(2)	105.2(9)	C(23)-N(22)-C(26)	128.2(11)
U(1)-O(32)	*2.452(8)*	C(28)-C(29)	1.348(19)	C(3)-N(2)-N(1)	111.0(8)	N(21)-N(22)-C(26)	119.3(9)
U(1)-N(41)	*2.551(11)*	C(29)-C(30)	1.366(19)	C(3)-N(2)-C(6)	129.5(9)	O(23)-C(23)-N(22)	124.5(11)
N(1)-C(5)	1.304(14)	C(30)-C(31)	1.38(2)	N(1)-N(2)-C(6)	119.2(8)	O(23)-C(23)-C(24)	130.0(11)
N(1)-N(2)	1.409(13)	*C(32)-O(32)*	*1.244(15)*	O(3)-C(3)-N(2)	124.8(10)	N(22)-C(23)-C(24)	105.5(11)
N(2)-C(3)	1.367(13)	C(32)-O(33)	1.328(16)	O(3)-C(3)-C(4)	129.1(10)	C(23)-O(23)-U(1)	130.5(7)
N(2)-C(6)	1.416(15)	O(33)-C(34)	1.472(15)	N(2)-C(3)-C(4)	106.1(9)	C(23)-C(24)-C(25)	105.2(11)
C(3)-O(3)	*1.279(13)*	C(34)-C(35)	1.50(2)	C(3)-O(3)-U(1)	127.4(7)	C(23)-C(24)-C(32)	122.1(12)
C(3)-C(4)	1.410(16)	N(41)-C(46)	1.340(16)	C(12)-C(4)-C(3)	119.1(12)	C(25)-C(24)-C(32)	132.6(13)
C(4)-C(12)	1.40(3)	N(41)-C(42)	1.377(19)	C(12)-C(4)-C(5)	135.3(13)	N(21)-C(25)-C(24)	112.7(14)
C(4)-C(5)	1.416(15)	C(42)-C(43)	1.39(2)	C(3)-C(4)-C(5)	105.0(9)	C(31)-C(26)-C(27)	119.7(11)
C(4)-C(12')	1.46(3)	C(43)-C(44)	1.37(2)	C(3)-C(4)-C(12')	128.2(14)	C(31)-C(26)-N(22)	121.7(11)
C(6)-C(11)	1.388(18)	C(44)-C(45)	1.36(2)	C(5)-C(4)-C(12')	126.1(14)	C(27)-C(26)-N(22)	118.6(11)
C(6)-C(7)	1.389(16)	C(45)-C(46)	1.369(16)	N(1)-C(5)-C(4)	112.7(10)	C(26)-C(27)-C(28)	119.7(11)
C(7)-C(8)	1.383(17)			C(11)-C(6)-C(7)	121.3(12)	C(29)-C(28)-C(27)	119.7(11)
C(8)-C(9)	1.37(2)	*O(50)-U(1)-O(60)*	*178.0(4)*	C(11)-C(6)-N(2)	120.0(11)	C(28)-C(29)-C(30)	121.0(13)
C(9)-C(10)	1.37(2)	O(50)-U(1)-O(23)	89.4(4)	C(7)-C(6)-N(2)	118.6(10)	C(29)-C(30)-C(31)	120.4(14)
C(10)-C(11)	1.38(2)	O(60)-U(1)-O(23)	90.2(4)	C(8)-C(7)-C(6)	118.7(11)	C(26)-C(31)-C(30)	119.6(12)
O(12)-C(12)	*1.19(3)*	O(50)-U(1)-O(3)	94.8(4)	C(9)-C(8)-C(7)	120.0(12)	O(32)-C(32)-O(33)	122.2(10)
O(12)-C(12')	*1.36(3)*	O(60)-U(1)-O(3)	86.5(4)	C(10)-C(9)-C(8)	120.9(14)	O(32)-C(32)-C(24)	124.1(12)
C(12)-O(13)	1.32(2)	O(23)-U(1)-O(3)	149.8(3)	C(9)-C(10)-C(11)	120.8(14)	O(33)-C(32)-C(24)	113.6(11)
O(13)-C(14)	1.475(18)	O(50)-U(1)-O(12)	89.8(4)	C(10)-C(11)-C(6)	118.3(13)	C(32)-O(32)-U(1)	131.4(7)
C(14)-C(15)	1.48(2)	O(60)-U(1)-O(12)	89.1(4)	C(12)-O(12)-U(1)	125.0(14)	C(32)-O(33)-C(34)	118.0(10)
C(12')-O(13')	1.32(2)	O(23)-U(1)-O(12)	137.5(3)	C(12')-O(12)-U(1)	138.6(11)	O(33)-C(34)-C(35)	106.1(13)
O(13')-C(14')	1.473(19)	*O(3)-U(1)-O(12)*	*72.5(3)*	O(12)-C(12)-O(13)	114(2)	C(46)-N(41)-C(42)	116.5(12)
C(14')-C(15')	1.48(2)	O(50)-U(1)-O(32)	87.7(3)	O(12)-C(12)-C(4)	128.8(16)	C(46)-N(41)-U(1)	122.1(9)
N(21)-C(25)	1.314(17)	O(60)-U(1)-O(32)	94.1(4)	O(13)-C(12)-C(4)	114.6(18)	C(42)-N(41)-U(1)	121.0(9)
N(21)-N(22)	1.381(16)	*O(23)-U(1)-O(32)*	*73.2(3)*	C(12)-O(13)-C(14)	116.6(16)	N(41)-C(42)-C(43)	121.3(16)
N(22)-C(23)	1.370(14)	O(3)-U(1)-O(32)	77.1(3)	O(13)-C(14)-C(15)	108.3(15)	C(44)-C(43)-C(42)	119.2(14)
N(22)-C(26)	1.444(15)	O(12)-U(1)-O(32)	149.2(3)	O(13')-C(12')-O(12)	128(2)	C(45)-C(44)-C(43)	119.9(12)
C(23)-O(23)	*1.268(15)*	*O(50)-U(1)-N(41)*	*89.4(4)*	O(13')-C(12')-C(4)	120(2)	C(44)-C(45)-C(46)	118.4(13)
C(23)-C(24)	1.391(19)	*O(60)-U(1)-N(41)*	*88.6(4)*	O(12)-C(12')-C(4)	111.6(16)	N(41)-C(46)-C(45)	124.4(12)

Table S40. Bond lengths [Å] and angles [°] for **75**.

U(1A)-O(70A)	*1.750(8)*	C(33A)-C(34A)	1.430(14)	C(3B)-O(3B)	*1.266(12)*	O(43B)-C(44B)	1.458(15)	O(3A)-U(1A)-N(60A)	69.3(2)
U(1A)-O(80A)	*1.763(8)*	C(34A)-C(45A)	1.408(13)	C(3B)-C(4B)	1.383(16)	C(45B)-O(45B)	*1.284(12)*	C(5A)-N(1A)-N(2A)	104.5(8)
U(1A)-O(33A)	*2.358(6)*	C(34A)-C(35A)	1.423(13)	C(4B)-C(15B)	1.418(16)	C(45B)-C(46B)	1.482(16)	C(3A)-N(2A)-N(1A)	112.0(8)
U(1A)-O(15A)	*2.367(6)*	C(36A)-C(37A)	1.504(16)	C(4B)-C(5B)	1.458(15)	C(46B)-C(47B)	1.395(17)	C(3A)-N(2A)-C(6A)	128.5(9)
U(1A)-O(45A)	*2.369(7)*	C(37A)-C(42A)	1.371(15)	C(6B)-C(7B)	1.509(18)	C(46B)-C(51B)	1.411(15)	N(1A)-N(2A)-C(6A)	119.5(9)
U(1A)-O(3A)	*2.388(7)*	C(37A)-C(38A)	1.375(15)	C(7B)-C(12B)	1.398(18)	C(47B)-C(48B)	1.388(18)	O(3A)-C(3A)-N(2A)	122.3(9)
U(1A)-N(60A)	*2.568(8)*	C(38A)-C(39A)	1.365(18)	C(7B)-C(8B)	1.402(16)	C(48B)-C(49B)	1.410(16)	O(3A)-C(3A)-C(4A)	130.2(10)
N(1A)-C(5A)	1.302(13)	C(39A)-C(40A)	1.388(16)	C(8B)-C(9B)	1.386(17)	C(49B)-C(50B)	1.374(16)	N(2A)-C(3A)-C(4A)	107.4(9)
N(1A)-N(2A)	1.419(11)	C(40A)-C(41A)	1.359(16)	C(9B)-C(10B)	1.383(18)	C(50B)-C(51B)	1.369(16)	C(3A)-O(3A)-U(1A)	130.5(6)
N(2A)-C(3A)	1.338(13)	C(40A)-O(43A)	1.364(15)	C(10B)-O(13B)	1.378(16)	O(60B)-C(61B)	1.403(13)	C(15A)-C(4A)-C(3A)	124.1(10)
N(2A)-C(6A)	1.451(13)	C(41A)-C(42A)	1.352(17)	C(10B)-C(11B)	1.388(18)	N(91)-C(92)	1.347(16)	C(15A)-C(4A)-C(5A)	132.5(10)
C(3A)-O(3A)	*1.283(11)*	O(43A)-C(44A)	1.419(15)	C(11B)-C(12B)	1.407(18)	N(91)-C(96)	1.353(14)	C(3A)-C(4A)-C(5A)	103.4(9)
C(3A)-C(4A)	1.409(14)	C(45A)-O(45A)	*1.271(11)*	O(13B)-C(14B)	1.488(18)	C(92)-C(93)	1.381(19)	N(1A)-C(5A)-C(4A)	112.6(9)
C(4A)-C(15A)	1.405(14)	C(45A)-C(46A)	1.469(13)	C(15B)-O(15B)	*1.269(12)*	C(93)-C(94)	1.381(19)	N(2A)-C(6A)-C(7A)	115.2(10)
C(4A)-C(5A)	1.452(15)	C(46A)-C(51A)	1.401(13)	C(15B)-C(16B)	1.481(16)	C(94)-C(95)	1.361(18)	C(12A)-C(7A)-C(8A)	117.3(12)
C(6A)-C(7A)	1.489(17)	C(46A)-C(47A)	1.403(14)	C(16B)-C(21B)	1.331(17)	C(95)-C(96)	1.364(17)	C(12A)-C(7A)-C(6A)	121.0(10)
C(7A)-C(12A)	1.358(16)	C(47A)-C(48A)	1.371(15)	C(16B)-C(17B)	1.401(16)			C(8A)-C(7A)-C(6A)	121.6(12)
C(7A)-C(8A)	1.373(15)	C(48A)-C(49A)	1.344(16)	C(17B)-C(18B)	1.394(17)	O(70A)-U(1A)-O(80A)	*179.1(3)*	C(7A)-C(8A)-C(9A)	121.9(12)
C(8A)-C(9A)	1.380(17)	C(49A)-C(50A)	1.371(16)	C(18B)-C(19B)	1.347(18)	O(70A)-U(1A)-O(33A)	88.0(3)	C(10A)-C(9A)-C(8A)	119.9(11)
C(9A)-C(10A)	1.368(16)	C(50A)-C(51A)	1.399(15)	C(19B)-C(20B)	1.431(18)	O(80A)-U(1A)-O(33A)	91.2(3)	O(13A)-C(10A)-C(9A)	115.4(10)
C(10A)-O(13A)	1.362(14)	N(60A)-C(61A)	1.300(14)	C(20B)-C(21B)	1.378(18)	O(70A)-U(1A)-O(15A)	89.6(3)	O(13A)-C(10A)-C(11A)	125.7(12)
C(10A)-C(11A)	1.383(15)	N(60A)-C(65A)	1.305(15)	N(31B)-C(35B)	1.286(15)	O(80A)-U(1A)-O(15A)	91.3(3)	C(9A)-C(10A)-C(11A)	118.9(13)
C(11A)-C(12A)	1.376(17)	C(61A)-C(62A)	1.376(17)	N(31B)-N(32B)	1.400(13)	O(33A)-U(1A)-O(15A)	148.2(2)	C(12A)-C(11A)-C(10A)	119.6(13)
O(13A)-C(14A)	1.413(15)	C(62A)-C(63A)	1.38(2)	N(32B)-C(33B)	1.342(13)	O(70A)-U(1A)-O(45A)	87.1(3)	C(7A)-C(12A)-C(11A)	122.3(11)
C(15A)-O(15A)	*1.269(11)*	C(63A)-C(64A)	1.328(18)	N(32B)-C(36B)	1.470(15)	O(80A)-U(1A)-O(45A)	93.1(3)	C(10A)-O(13A)-C(14A)	116.8(9)
C(15A)-C(16A)	1.475(13)	C(64A)-C(65A)	1.367(15)	C(33B)-O(33B)	*1.289(13)*	O(33A)-U(1A)-O(45A)	*73.1(2)*	O(15A)-C(15A)-C(4A)	121.3(9)
C(16A)-C(17A)	1.388(14)	U(1B)-O(70B)	*1.758(7)*	C(33B)-C(34B)	1.400(16)	O(15A)-U(1A)-O(45A)	75.1(2)	O(15A)-C(15A)-C(16A)	116.8(9)
C(16A)-C(21A)	1.393(14)	U(1B)-O(80B)	*1.768(7)*	C(34B)-C(45B)	1.400(15)	O(70A)-U(1A)-O(3A)	90.8(3)	C(4A)-C(15A)-C(16A)	121.9(9)
C(17A)-C(18A)	1.367(15)	U(1B)-O(33B)	*2.347(7)*	C(34B)-C(35B)	1.433(15)	O(80A)-U(1A)-O(3A)	89.5(3)	C(15A)-O(15A)-U(1A)	139.7(6)
C(18A)-C(19A)	1.377(16)	U(1B)-O(3B)	*2.358(8)*	C(36B)-C(37B)	1.507(16)	O(33A)-U(1A)-O(3A)	138.4(2)	C(17A)-C(16A)-C(21A)	117.7(9)
C(19A)-C(20A)	1.342(15)	U(1B)-O(60B)	*2.391(5)*	C(37B)-C(38B)	1.377(15)	O(15A)-U(1A)-O(3A)	*73.3(2)*	C(17A)-C(16A)-C(15A)	122.2(10)
C(20A)-C(21A)	1.388(14)	U(1B)-O(15B)	*2.393(7)*	C(37B)-C(42B)	1.389(15)	O(45A)-U(1A)-O(3A)	148.4(2)	C(21A)-C(16A)-C(15A)	120.1(9)
N(31A)-C(35A)	1.298(13)	U(1B)-O(45B)	*2.416(9)*	C(38B)-C(39B)	1.365(16)	O(70A)-U(1A)-N(60A)	*88.0(3)*	C(18A)-C(17A)-C(16A)	122.4(11)
N(31A)-N(32A)	1.385(12)	N(1B)-C(5B)	1.276(16)	C(39B)-C(40B)	1.385(15)	O(80A)-U(1A)-N(60A)	*91.3(3)*	C(17A)-C(18A)-C(19A)	118.4(11)
N(32A)-C(33A)	1.331(12)	N(1B)-N(2B)	1.403(13)	C(40B)-C(41B)	1.374(17)	O(33A)-U(1A)-N(60A)	69.2(3)	C(20A)-C(19A)-C(18A)	121.0(11)
N(32A)-C(36A)	1.465(13)	N(2B)-C(3B)	1.369(14)	C(40B)-O(43B)	1.377(14)	O(15A)-U(1A)-N(60A)	142.5(3)	C(19A)-C(20A)-C(21A)	121.2(11)
C(33A)-O(33A)	*1.289(12)*	N(2B)-C(6B)	1.423(15)	C(41B)-C(42B)	1.383(16)	O(45A)-U(1A)-N(60A)	142.1(2)	C(20A)-C(21A)-C(16A)	119.3(10)

Table S40. Part 2.

C(35A)-N(31A)-N(32A)	103.9(8)	C(61A)-N(60A)-C(65A)	116.3(10)	C(3B)-O(3B)-U(1B)	128.5(8)	C(33B)-O(33B)-U(1B)	131.3(7)
C(33A)-N(32A)-N(31A)	112.8(9)	C(61A)-N(60A)-U(1A)	124.0(8)	C(3B)-C(4B)-C(15B)	122.4(10)	C(45B)-C(34B)-C(33B)	123.9(10)
C(33A)-N(32A)-C(36A)	126.7(9)	C(65A)-N(60A)-U(1A)	119.6(8)	C(3B)-C(4B)-C(5B)	104.5(11)	C(45B)-C(34B)-C(35B)	132.8(12)
N(31A)-N(32A)-C(36A)	120.5(8)	N(60A)-C(61A)-C(62A)	124.3(14)	C(15B)-C(4B)-C(5B)	133.1(12)	C(33B)-C(34B)-C(35B)	103.3(10)
O(33A)-C(33A)-N(32A)	122.6(10)	C(61A)-C(62A)-C(63A)	117.1(14)	N(1B)-C(5B)-C(4B)	112.2(11)	N(31B)-C(35B)-C(34B)	113.2(12)
O(33A)-C(33A)-C(34A)	130.3(9)	C(64A)-C(63A)-C(62A)	119.2(12)	N(2B)-C(6B)-C(7B)	115.1(11)	N(32B)-C(36B)-C(37B)	111.0(9)
N(32A)-C(33A)-C(34A)	107.1(9)	C(63A)-C(64A)-C(65A)	118.7(13)	C(12B)-C(7B)-C(8B)	119.7(13)	C(38B)-C(37B)-C(42B)	116.3(11)
C(33A)-O(33A)-U(1A)	127.4(6)	N(60A)-C(65A)-C(64A)	124.3(13)	C(12B)-C(7B)-C(6B)	120.3(12)	C(38B)-C(37B)-C(36B)	123.2(10)
C(45A)-C(34A)-C(35A)	134.7(10)	O(70B)-U(1B)-O(80B)	*179.2(4)*	C(8B)-C(7B)-C(6B)	120.0(12)	C(42B)-C(37B)-C(36B)	120.4(11)
C(45A)-C(34A)-C(33A)	123.6(9)	O(70B)-U(1B)-O(33B)	86.8(3)	C(9B)-C(8B)-C(7B)	119.7(13)	C(39B)-C(38B)-C(37B)	122.4(11)
C(35A)-C(34A)-C(33A)	101.7(9)	O(80B)-U(1B)-O(33B)	92.7(3)	C(10B)-C(9B)-C(8B)	121.2(13)	C(38B)-C(39B)-C(40B)	120.4(12)
N(31A)-C(35A)-C(34A)	114.5(10)	O(70B)-U(1B)-O(3B)	88.9(3)	O(13B)-C(10B)-C(9B)	125.5(14)	C(41B)-C(40B)-O(43B)	125.9(11)
N(32A)-C(36A)-C(37A)	114.3(10)	O(80B)-U(1B)-O(3B)	91.9(3)	O(13B)-C(10B)-C(11B)	115.1(13)	C(41B)-C(40B)-C(39B)	119.0(12)
C(42A)-C(37A)-C(38A)	116.0(11)	O(33B)-U(1B)-O(3B)	139.9(3)	C(9B)-C(10B)-C(11B)	119.4(14)	O(43B)-C(40B)-C(39B)	115.1(12)
C(42A)-C(37A)-C(36A)	121.7(11)	O(70B)-U(1B)-O(60B)	*95.8(3)*	C(10B)-C(11B)-C(12B)	120.6(14)	C(40B)-C(41B)-C(42B)	119.5(11)
C(38A)-C(37A)-C(36A)	122.2(10)	O(80B)-U(1B)-O(60B)	*84.7(3)*	C(7B)-C(12B)-C(11B)	119.4(13)	C(41B)-C(42B)-C(37B)	122.4(12)
C(39A)-C(38A)-C(37A)	123.1(12)	O(33B)-U(1B)-O(60B)	71.4(2)	C(10B)-O(13B)-C(14B)	115.0(12)	C(40B)-O(43B)-C(44B)	113.9(11)
C(38A)-C(39A)-C(40A)	119.1(12)	O(3B)-U(1B)-O(60B)	69.5(2)	O(15B)-C(15B)-C(4B)	119.9(10)	O(45B)-C(45B)-C(34B)	120.8(11)
C(41A)-C(40A)-O(43A)	116.9(11)	O(70B)-U(1B)-O(15B)	90.7(3)	O(15B)-C(15B)-C(16B)	117.5(12)	O(45B)-C(45B)-C(46B)	117.0(9)
C(41A)-C(40A)-C(39A)	117.9(13)	O(80B)-U(1B)-O(15B)	89.3(3)	C(4B)-C(15B)-C(16B)	122.6(10)	C(34B)-C(45B)-C(46B)	122.2(10)
O(43A)-C(40A)-C(39A)	125.2(12)	O(33B)-U(1B)-O(15B)	147.7(3)	C(15B)-O(15B)-U(1B)	139.7(8)	C(45B)-O(45B)-U(1B)	138.7(7)
C(42A)-C(41A)-C(40A)	121.8(12)	O(3B)-U(1B)-O(15B)	*72.2(3)*	C(21B)-C(16B)-C(17B)	120.3(12)	C(47B)-C(46B)-C(51B)	119.5(12)
C(41A)-C(42A)-C(37A)	121.9(12)	O(60B)-U(1B)-O(15B)	140.9(2)	C(21B)-C(16B)-C(15B)	120.6(11)	C(47B)-C(46B)-C(45B)	121.7(11)
C(40A)-O(43A)-C(44A)	118.7(10)	O(70B)-U(1B)-O(45B)	90.9(3)	C(17B)-C(16B)-C(15B)	119.1(12)	C(51B)-C(46B)-C(45B)	118.8(11)
O(45A)-C(45A)-C(34A)	119.9(9)	O(80B)-U(1B)-O(45B)	88.3(3)	C(18B)-C(17B)-C(16B)	119.3(13)	C(48B)-C(47B)-C(46B)	120.9(12)
O(45A)-C(45A)-C(46A)	117.3(8)	O(33B)-U(1B)-O(45B)	*72.6(3)*	C(19B)-C(18B)-C(17B)	120.4(12)	C(47B)-C(48B)-C(49B)	118.6(13)
C(34A)-C(45A)-C(46A)	122.8(9)	O(3B)-U(1B)-O(45B)	147.4(3)	C(18B)-C(19B)-C(20B)	119.8(13)	C(50B)-C(49B)-C(48B)	120.0(12)
C(45A)-O(45A)-U(1A)	138.4(6)	O(60B)-U(1B)-O(45B)	142.86(19)	C(21B)-C(20B)-C(19B)	118.3(14)	C(51B)-C(50B)-C(49B)	121.8(12)
C(51A)-C(46A)-C(47A)	119.8(10)	O(15B)-U(1B)-O(45B)	75.2(2)	C(16B)-C(21B)-C(20B)	121.9(12)	C(50B)-C(51B)-C(46B)	119.0(12)
C(51A)-C(46A)-C(45A)	116.3(9)	C(5B)-N(1B)-N(2B)	105.4(10)	C(35B)-N(31B)-N(32B)	104.7(9)	C(61B)-O(60B)-U(1B)	132.5(7)
C(47A)-C(46A)-C(45A)	123.9(9)	C(3B)-N(2B)-N(1B)	111.5(10)	C(33B)-N(32B)-N(31B)	111.6(10)	C(92)-N(91)-C(96)	115.7(11)
C(48A)-C(47A)-C(46A)	119.3(10)	C(3B)-N(2B)-C(6B)	127.3(10)	C(33B)-N(32B)-C(36B)	128.7(11)	N(91)-C(92)-C(93)	125.0(13)
C(49A)-C(48A)-C(47A)	122.2(12)	N(1B)-N(2B)-C(6B)	120.6(10)	N(31B)-N(32B)-C(36B)	119.5(9)	C(94)-C(93)-C(92)	116.7(13)
C(48A)-C(49A)-C(50A)	119.1(11)	O(3B)-C(3B)-N(2B)	121.4(12)	O(33B)-C(33B)-N(32B)	122.4(12)	C(95)-C(94)-C(93)	119.8(13)
C(49A)-C(50A)-C(51A)	122.2(11)	O(3B)-C(3B)-C(4B)	132.1(12)	O(33B)-C(33B)-C(34B)	130.4(10)	C(94)-C(95)-C(96)	119.8(13)
C(50A)-C(51A)-C(46A)	117.4(11)	N(2B)-C(3B)-C(4B)	106.2(10)	N(32B)-C(33B)-C(34B)	107.2(10)	N(91)-C(96)-C(95)	122.9(12)

Table S41. Bond lengths [Å] and angles [°] for **76**.

U(1)-O(1)	*1.773(3)*	C(6)-C(5)	1.516(7)	C(4)-O(4)-U(1)	137.1(3)	C(5)-C(6)-H(6A)	109.5
U(1)-O(3)	*2.297(3)*	C(9)-H(9)	0.9500	C(7)-O(5)-U(1)	126.8(3)	C(5)-C(6)-H(6B)	109.5
U(1)-O(2)	*1.763(4)*	C(12)-H(12A)	0.9800	C(17)-N(5)-U(1)	123.0(3)	C(5)-C(6)-H(6C)	109.5
U(1)-O(6)	*2.424(3)*	C(12)-H(12B)	0.9800	C(17)-N(5)-C(13)	116.8(4)	O(4)-C(4)-O(7)	120.7(4)
U(1)-O(4)	*2.411(3)*	C(12)-H(12C)	0.9800	C(13)-N(5)-U(1)	120.1(3)	O(4)-C(4)-C(2)	123.7(4)
U(1)-O(5)	*2.359(3)*	C(12)-C(11)	1.503(8)	C(9)-N(4)-N(3)	104.1(4)	O(7)-C(4)-C(2)	115.6(4)
U(1)-N(5)	*2.557(4)*	C(13)-H(13)	0.9500	C(4)-O(7)-C(5)	117.1(4)	N(4)-C(9)-C(8)	112.6(4)
O(3)-C(1)	*1.290(6)*	C(13)-C(14)	1.348(8)	C(10)-O(8)-C(11)	117.0(4)	N(4)-C(9)-H(9)	123.7
O(6)-C(10)	*1.238(6)*	C(14)-H(14)	0.9500	O(3)-C(1)-N(1)	124.3(4)	C(8)-C(9)-H(9)	123.7
O(4)-C(4)	*1.239(6)*	C(5)-H(5A)	0.9900	O(3)-C(1)-C(2)	130.0(4)	H(12A)-C(12)-H(12B)	109.5
O(5)-C(7)	*1.297(6)*	C(5)-H(5B)	0.9900	N(1)-C(1)-C(2)	105.6(4)	H(12A)-C(12)-H(12C)	109.5
N(5)-C(17)	1.331(6)	C(3)-H(3A)	0.9500	N(4)-N(3)-H(3)	123.7	H(12B)-C(12)-H(12C)	109.5
N(5)-C(13)	1.354(7)	C(16)-H(16)	0.9500	C(7)-N(3)-N(4)	112.6(4)	C(11)-C(12)-H(12A)	109.5
N(4)-N(3)	1.403(6)	C(11)-H(11A)	0.9900	C(7)-N(3)-H(3)	123.7	C(11)-C(12)-H(12B)	109.5
N(4)-C(9)	1.318(7)	C(11)-H(11B)	0.9900	C(1)-N(1)-H(1)	124.0	C(11)-C(12)-H(12C)	109.5
O(7)-C(4)	1.330(6)			C(1)-N(1)-N(2)	112.1(4)	N(5)-C(13)-H(13)	118.6
O(7)-C(5)	1.464(6)	O(1)-U(1)-O(3)	91.97(15)	N(2)-N(1)-H(1)	124.0	C(14)-C(13)-N(5)	122.9(5)
O(8)-C(10)	1.327(6)	O(1)-U(1)-O(6)	86.90(15)	C(1)-C(2)-C(4)	121.9(4)	C(14)-C(13)-H(13)	118.6
O(8)-C(11)	1.462(7)	O(1)-U(1)-O(4)	89.53(14)	C(3)-C(2)-C(1)	105.3(4)	C(15)-C(14)-H(14)	120.0
C(1)-N(1)	1.358(6)	O(1)-U(1)-O(5)	91.38(14)	C(3)-C(2)-C(4)	132.8(5)	C(13)-C(14)-C(15)	120.1(5)
C(1)-C(2)	1.415(7)	*O(1)-U(1)-N(5)*	*88.81(14)*	O(5)-C(7)-N(3)	124.0(4)	C(13)-C(14)-H(14)	120.0
N(3)-H(3)	0.8800	O(3)-U(1)-O(6)	150.32(12)	O(5)-C(7)-C(8)	130.1(4)	O(7)-C(5)-C(6)	110.8(4)
N(3)-C(7)	1.348(7)	*O(3)-U(1)-O(4)*	*73.62(12)*	N(3)-C(7)-C(8)	105.9(4)	O(7)-C(5)-H(5A)	109.5
N(1)-H(1)	0.8800	O(3)-U(1)-O(5)	76.35(12)	C(3)-N(2)-N(1)	105.1(4)	O(7)-C(5)-H(5B)	109.5
N(1)-N(2)	1.393(6)	O(3)-U(1)-N(5)	142.32(12)	O(6)-C(10)-O(8)	121.4(4)	C(6)-C(5)-H(5A)	109.5
C(2)-C(4)	1.416(7)	*O(2)-U(1)-O(1)*	*176.14(13)*	O(6)-C(10)-C(8)	123.3(4)	C(6)-C(5)-H(5B)	109.5
C(2)-C(3)	1.414(7)	O(2)-U(1)-O(3)	91.38(15)	O(8)-C(10)-C(8)	115.3(4)	H(5A)-C(5)-H(5B)	108.1
C(7)-C(8)	1.411(6)	O(2)-U(1)-O(6)	91.13(15)	N(5)-C(17)-H(17)	118.3	C(2)-C(3)-H(3A)	124.1
N(2)-C(3)	1.319(7)	O(2)-U(1)-O(4)	89.58(15)	N(5)-C(17)-C(16)	123.4(5)	N(2)-C(3)-C(2)	111.9(4)
C(10)-C(8)	1.425(7)	O(2)-U(1)-O(5)	91.27(14)	C(16)-C(17)-H(17)	118.3	N(2)-C(3)-H(3A)	124.1
C(17)-H(17)	0.9500	*O(2)-U(1)-N(5)*	*87.36(15)*	C(7)-C(8)-C(10)	123.6(4)	C(17)-C(16)-C(15)	118.5(5)
C(17)-C(16)	1.376(8)	O(6)-U(1)-N(5)	67.35(12)	C(7)-C(8)-C(9)	104.9(4)	C(17)-C(16)-H(16)	120.8
C(8)-C(9)	1.416(7)	O(4)-U(1)-O(6)	135.97(12)	C(9)-C(8)-C(10)	131.4(4)	C(15)-C(16)-H(16)	120.8
C(15)-H(15)	0.9500	O(4)-U(1)-N(5)	68.71(12)	C(14)-C(15)-H(15)	120.8	O(8)-C(11)-C(12)	106.0(5)
C(15)-C(14)	1.368(8)	*O(5)-U(1)-O(6)*	*74.03(11)*	C(14)-C(15)-C(16)	118.4(5)	O(8)-C(11)-H(11A)	110.5
C(15)-C(16)	1.382(8)	O(5)-U(1)-O(4)	149.97(11)	C(16)-C(15)-H(15)	120.8	O(8)-C(11)-H(11B)	110.5
C(6)-H(6A)	0.9800	O(5)-U(1)-N(5)	141.31(11)	H(6A)-C(6)-H(6B)	109.5	C(12)-C(11)-H(11A)	110.5
C(6)-H(6B)	0.9800	C(1)-O(3)-U(1)	133.0(3)	H(6A)-C(6)-H(6C)	109.5	C(12)-C(11)-H(11B)	110.5
C(6)-H(6C)	0.9800	C(10)-O(6)-U(1)	132.1(3)	H(6B)-C(6)-H(6C)	109.5	H(11A)-C(11)-H(11B)	108.7
