# Supplementary material for: Syntheses and Characterization of Main Group, Transition Metal, Lanthanide, and Actinide Complexes of Bidentate Acylpyrazolone Ligands
Source: Inorg Chem. 2023 Aug 7;62(33):13253–76. doi: 10.1021/acs.inorgchem.3c01506 (PMC10445273; doi:10.1021/acs.inorgchem.3c01506)
Supplement: Supplementary file 2 — ic3c01506_si_003.pdf [file ic3c01506_si_003.pdf]

Syntheses and Characterization of Main Group, Transition Metal, Lanthanide and Actinide Complexes Bidentate Acylpyrazolone Ligands

Thomas Mies,[1] Andrew J. P. White,[1] Henry S. Rzepa,[1] Luciano Barluzzi,[2] Mohit Devgan,[1] Richard A. Layfield,[2] and Anthony G. M. Barrett[1]*

[1] Department of Chemistry, Imperial College, Molecular Sciences Research Hub, White City Campus, Wood Lane, London W12 0BZ, England.

[2] Department of Chemistry, University of Sussex, Falmer, Brighton, BN1 9QR, England.

Experimental Procedures

Contents:

I. **General Experimental**
II. **Experimental Procedures**
III. **References**

I. General Experimental Details

CH_2Cl_2, EtOH, MeOH, THF, Et_2O, Me_2CO and PhMe were purified by filtration through activated alumina columns or purchased as extra dry solvents and stored over 4 Å molecular sieves. HN*i*Pr_2, NEt_3, pyridine and *iso*-Pr_2NEt were purchased as extra dry reagents and stored over 4 Å molecular sieves. Hexanes refers to the petroleum alkane fraction boiling between 40 °C and 60 °C.

Reactions were carried out in cooled oven-dried (200 °C) glassware under an argon atmosphere using standard Schlenk techniques and with transfers by cannulas and syringes. Unless stated to the contrary, reactions were carried out at room temperature, or at reaction temperatures recorded in the external bath. The progress of reactions was monitored by analytical thin-layer chromatography (TLC) on silica gel coated aluminum oxide F_{254} plates. Components on TLC plates were visualized under UV light or by spraying with aqueous $KMnO_4$ or acidic vanillin and warming. Pyrazolone **5** (**^{1}H-NMR** (400 MHz, CD_3OD): δ (ppm) = 7.83 (s, 1H), 4.27 (q, *J* = 7.1 Hz, 2H), 1.32 (t, *J* = 7.1 Hz, 3H). **^{13}C{^{1}H}-NMR** (101 MHz, CD_3OD): δ (ppm) = 165.7, 162.4, 135.4, 98.2, 61.0, 14.7.) and Ca-complex **22** (**^{1}H-NMR** (400 MHz, $OS(CD_3)_2$): δ (ppm) = 10.74 (s, 1H), 7.25 (d, *J* = 1.9 Hz, 1H), 4.07 (q, *J* = 7.0 Hz, 2H), 3.35 (s, 5H), 1.19 (t, *J* = 7.1 Hz, 3H). **^{13}C{^{1}H}-NMR** (126 MHz, $OS(CD_3)_2$): δ (ppm) = 166.9, 166.9, 138.7, 90.8, 58.5, 14.6.) were prepared according to literature known procedures.[1]

^{1}H-NMR and proton decoupled ^{13}C-NMR spectra were respectively recorded at 400 MHz and 101 MHz in deuterated solvents at ambient temperature with chemical shifts are reported in ppm (δ) relative to Me_4Si and referenced to the residual solvent peak ($CDCl_3$: ^{1}H at 7.26 ppm, ^{13}C at 77.16 ppm; CD_3OD: ^{1}H at 3.31 and 4.87 ppm, ^{13}C at 49.0 ppm; $OC(CD_3)_2$: ^{1}H at 2.05 ppm, ^{13}C at 29.8 and 206.3 ppm; $OS(CD_3)_2$: ^{1}H at 2.50 ppm, ^{13}C at 39.5 ppm; CD_3CN: ^{1}H at 1.94 ppm; ^{13}C at 1.3 and 118.3 ppm). Assignments of the ^{1}H-NMR and ^{13}C-NMR spectra were made by the analysis of chemical shift and coupling constant values, and, as appropriate, using COSY, DEPT-135, HSQC and HMBC. MS spectra were recorded by the Imperial College Mass Spectrometry Service under conditions of electrospray ionization (ES), chemical ionization (CI) or electron ionization (EI). Infra-red spectra of solids and liquids were recorded as thin films. Melting points were recorded using a hot-stage microscope apparatus and were reported uncorrected in degrees Celsius (°C). X-ray diffraction data were recorded at the Imperial College X-ray Crystallography Facility. Elemental microanalyses were recorded at the University of Cambridge Microanalysis Facility.

II. Experimental Procedures

Ethyl 3-Oxo-2-phenyl-2,3-dihydro-1*H*-pyrazole-4-carboxylate (6)

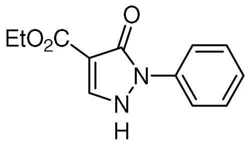

Diethyl ethoxymethylenemalonate (**4**) (8.649 g, 8.082 mL, 40.00 mmol, 1.00 equiv) was added with stirring to K_2CO_3 (5.528 g, 40.00 mmol, 1.00 equiv) and phenylhydrazine (4.326 g, 3.940 mL, 40.00 mmol, 1.00 equiv) in H_2O (150 mL). The mixture was heated to 100 °C for 18 h, after which it was cooled to room temperature and was diluted with EtOAc (40 mL). The organic layer was separated, and the aqueous layer was further extracted with EtOAc (2 × 30 mL). The combined organic layers were washed with aqueous NaOH (2 M; 30 mL). The combined aqueous layers were acidified with aqueous HCl (4 M) to pH = 1 – 2 and extracted with EtOAc (3 x 50 mL). The combined organic layers were washed with brine (40 mL), dried (Na_2SO_4), filtered and concentrated under reduced pressure to give pyrazolone **6** (8.553 g, 36.83 mmol, 92%) as an off-white to brown solid, essentially pure by its ^{1}H NMR spectrum. Analytically pure samples were obtained by recrystallization from EtOH.

$\mathbf{R_f}$ (hexanes : EtOAc 7 : 3) = 0.07.

^{1}H-NMR (400 MHz, $CDCl_3$): δ (ppm) = 9.77 (s, 1H), 7.83 – 7.78 (m, 2H), 7.77 (s, 1H), 7.47 (t, *J* = 7.8 Hz, 2H), 7.37 – 7.29 (m, 1H), 4.37 (q, *J* = 7.1 Hz, 2H), 1.44 – 1.35 (m, 3H).

^{1}H-NMR (400 MHz, CD_3OD): δ (ppm) = 7.81 (s, 1H), 7.71 – 7.64 (m, 2H), 7.47 (t, *J* = 7.8 Hz, 2H), 7.35 (t, *J* = 7.5 Hz, 1H), 4.30 (q, *J* = 7.1 Hz, 2H), 1.34 (t, *J* = 7.1 Hz, 3H).

^{13}C{^{1}H}-NMR (101 MHz, $CDCl_3$): δ (ppm) = 166.5, 156.8, 138.6, 137.6, 129.3, 127.3, 121.5, 95.2, 60.9, 14.5.

^{13}C{^{1}H}-NMR (101 MHz, CD_3OD): δ (ppm) = 165.1, 156.6, 141.1, 138.8, 130.1, 128.5, 123.8, 97.4, 61.1, 14.8.

IR (Diamond-ATR, neat) ν_{max} (cm^{-1}) = 2898, 1714, 1624, 1554, 1498, 1457, 1373, 1342, 1233, 1157, 1113, 1064, 926, 765, 746, 685.

HRMS (ES-ToF) *m/z*: $[M + H]^+$ calc. for $(C_{12}H_{13}N_2O_3)^+$: 233.0921, found: 233.0927.

m.p.: 99 – 101 °C (EtOH) (lit.[2] m.p. 104 °C).

Anal. Calcd for $C_{12}H_{12}N_2O_3$: C, 62.06; H, 5.21; N; 12.06. Found: C, 61.75; H, 5.22; N, 11.81.

The analytic data were in good agreement with literature values.[3]

Ethyl 2-(4-Methoxybenzyl)-3-oxo-2,3-dihydro-1*H*-pyrazole-4-carboxylate (8)

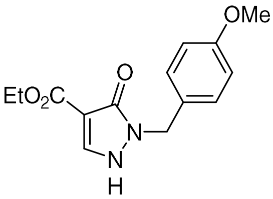

Diethyl ethoxymethylenemalonate (**4**) (8.030 g, 7.510 mL, 37.10 mmol, 1.00 equiv) was added with stirring to K_2CO_3 (5.128 g, 37.10 mmol, 1.00 equiv) and PMB-hydrazine hydrochloride **7** (7.000 g, 37.10 mmol, 1.00 equiv) in H_2O (180 mL). After heating at 100 °C for 18 h, the mixture was cooled to room temperature and diluted with EtOAc (40 mL). The organic layer was separated, and the aqueous layer was further extracted with EtOAc (2 × 30 mL). The combined organic layers were washed with

aqueous NaOH (2 M; 30 mL). The combined aqueous layers were acidified with aqueous HCl (4 M) to pH = 1 – 2, and extracted with EtOAc (3 x 50 mL). The combined organic layers were washed with brine (40 mL), dried (Na_2SO_4), filtered and concentrated under reduced pressure to give pyrazolone **8** (9.486 g, 34.59 mmol, 93%) as an off-white or yellow solid, essentially pure by its ^{1}H NMR spectrum. Analytically pure samples were obtained by recrystallization from EtOH.

R$_f$ (hexanes : EtOAc 1 : 1) = 0.06.

^{1}H-NMR (400 MHz, $CDCl_3$): δ (ppm) = 7.59 (d, *J* = 1.4 Hz, 1H), 7.25 – 7.22 (m, 2H), 6.86 – 6.82 (m, 2H), 5.07 (s, 2H), 4.29 (qd, *J* = 7.1, 1.4 Hz, 2H), 3.76 (d, *J* = 1.4 Hz, 3H), 1.33 (td, *J* = 7.1, 1.4 Hz, 3H).

^{1}H-NMR (400 MHz, CD_3OD): δ (ppm) = 7.65 (s, 1H), 7.21 – 7.14 (m, 2H), 6.89 – 6.81 (m, 2H), 5.04 (s, 2H), 4.25 (q, *J* = 7.1 Hz, 2H), 3.74 (s, 3H), 1.30 (t, *J* = 7.1 Hz, 3H).

^{13}C{^{1}H}-NMR (101 MHz, $CDCl_3$): δ (ppm) = 165.7, 159.3, 156.0, 138.0, 129.4, 127.8, 114.1, 94.3, 60.4, 55.2, 50.0, 15.3.

^{13}C{^{1}H}-NMR (101 MHz, CD_3OD): δ (ppm) = 164.9, 160.8, 156.4, 140.1, 130.1, 129.5, 115.0, 96.8, 60.9, 55.7, 50.3, 14.8.

IR (Diamond-ATR, neat) ν_{max} (cm^{-1}) = 2583, 1685, 1536, 1510, 1370, 1336, 1244, 1163, 1090, 1027, 793, 779, 724, 691.

HRMS (ES-ToF) *m/z*: [M + H]$^+$ calc. for $(C_{14}H_{17}N_2O_4)^+$: 277.1183, found: 277.1188.

m.p.: 102 – 105 °C (EtOH) (lit.[4] m.p. 105 °C).

Anal. Calcd for $C_{14}H_{16}N_2O_4$: C, 60.86; H, 5.84; N; 10.14. Found: C, 60.82; H, 5.81; N, 10.12.

The analytic data were in good agreement with literature values.[4]

2-(4-Methoxybenzyl)-2,4-dihydro-3*H*-pyrazol-3-one (9)

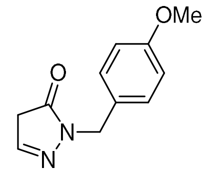

Aqueous KOH (2 M; 50 mL) was added with stirring to pyrazolone **8** (9.486 g, 34.33 mmol, 1.00 equiv) in EtOH and H_2O (1 : 1; 20 mL). After heating at 100 °C for 18 h, the mixture was cooled to room temperature and acidified with aqueous HCl (4 M) to pH = 1 – 2. The mixture was further heated at 100 °C for 18 h, after which the mixture was cooled to room temperature and diluted with EtOAc (40 mL). The organic layer was separated, and the aqueous layer was further extracted with EtOAc (2 × 30 mL). The combined organic layers were washed with brine (40 mL), dried ($MgSO_4$), filtered and concentrated under reduced pressure to give decarboxylated pyrazolone **9** (5.069 g, 24.83 mmol, 72%) as a yellow solid, essentially pure by its ^{1}H NMR spectrum. Analytically pure samples were obtained by recrystallization from EtOH.

R$_f$ (hexanes : EtOAc 1 : 1) = 0.33.

^{1}H-NMR Enol Form (400 MHz, $CDCl_3$): δ (ppm) = 13.90 (s, 1H), 7.27 (d, *J* = 8.5 Hz, 2H), 7.25 (d, *J* = 3.4 Hz, 1H), 6.76 – 6.71 (m, 2H), 5.47 (d, *J* = 3.0 Hz, 1H), 5.10 (s, 2H), 3.69 (s, 3H).

^{1}H-NMR Keto Form (400 MHz, $CDCl_3$): δ (ppm) = 7.28 (d, *J* = 8.9 Hz, 2H), 7.26 (s, 1H), 6.86 (d, *J* = 8.1 Hz, 2H), 4.78 (s, 2H), 3.78 (s, 3H), 3.27 (s, 2H).

^{13}C{^{1}H}-NMR Enol Form (101 MHz, $CDCl_3$): δ (ppm) = 159.4, 158.7, 134.1, 129.6, 126.9, 114.0, 90.7, 55.1, 47.9.

$^{13}C\{^1H\}$-NMR Keto Form (101 MHz, $CDCl_3$): δ (ppm) = 171.5, 159.4, 146.4, 129.9, 128.6, 114.2, 55.4, 47.6, 39.6.

IR (Diamond-ATR, neat) ν_{max} (cm^{-1}) = 2344, 1706, 1610, 1559, 1508, 1402, 1359, 1301, 1280, 1206, 1174, 1115, 1031, 938, 835, 922, 816, 774, 749, 736.

HRMS (ES-ToF) *m*/*z*: $[M + H]^+$ calc. for $(C_{11}H_{13}N_2O_2)^+$: 205.0972, found: 205.0979.

m.p.: 111 – 114 °C (EtOH) (lit.[4] m.p. 135 °C).

Anal. Calcd for $C_{11}H_{12}N_2O_2$: C, 64.69; H, 5.92; N; 13.72. Found: C, 65.24; H, 5.91; N, 12.70.

The analytic data were in good agreement with literature values.[4]

4-Benzoyl-2-(4-methoxybenzyl)-1,2-dihydro-3*H*-pyrazol-3-one (10)

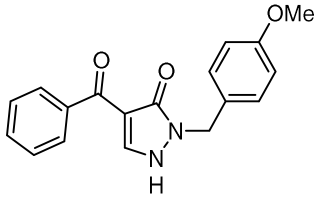

Bz-Cl (2.753 g, 2.270 mL, 19.59 mmol, 1.00 equiv) was added with stirring to $Ca(OH)_2$ (726.0 mg, 9.793 mmol, 2.00 equiv) and pyrazolone **9** (4.000g, 19.59 mmol, 1.00 equiv) in 1,4-dioxane (40 mL). After heating at 100 °C for 4 h, the mixture was cooled to room temperature and acidified with aqueous HCl (1 M) to pH = 1 – 2. The mixture was stirred for 1 , the precipitate was collected by filtration and air dried and subsequently dried *in vacuo*. Additional material was obtained through addition of H_2O to the filtrate. The precipitate was collected by filtration, air dried and subsequently dried *in vacuo*. Pyrazolone **10** (4.134 g, 13.41 mmol, 68%) was obtained as a yellow solid, essentially pure by its ^1H NMR spectrum. Analytically pure samples were obtained by recrystallization from EtOH.

R_f (hexanes : EtOAc 7 : 3) = 0.05.

^1H-NMR (400 MHz, $CDCl_3$): δ (ppm) = 7.94 – 7.86 (m, 2H), 7.78 (s, 1H), 7.60 (dd, *J* = 8.5, 6.0 Hz, 1H), 7.52 (t, *J* = 7.5 Hz, 2H), 7.32 (d, *J* = 8.2 Hz, 2H), 6.91 – 6.85 (m, 2H), 6.14 (s, 2H), 5.14 (s, 2H), 3.79 (s, 3H).

^1H-NMR (400 MHz, CD_3OD): δ (ppm) = 7.85 (d, *J* = 7.5 Hz, 2H), 7.77 (s, 1H), 7.60 (t, *J* = 7.3 Hz, 1H), 7.51 (t, *J* = 7.5 Hz, 2H), 7.24 (d, *J* = 8.2 Hz, 2H), 6.87 (d, *J* = 8.2 Hz, 2H), 4.91 (s, 2H), 3.75 (s, 3H).

$^{13}C\{^1H\}$-NMR (101 MHz, $CDCl_3$): δ (ppm) = 190.3, 159.8, 159.6, 139.1, 137.4, 132.9, 129.8, 128.9, 128.5, 127.7, 114.3, 102.5, 55.4, 49.9.

$^{13}C\{^1H\}$-NMR (101 MHz, CD_3OD): δ (ppm) = 191.3, 161.0, 159.2, 140.8, 139.4, 133.6, 130.3, 129.7, 129.6, 129.2, 115.1, 104.5, 55.7, 50.2.

IR (Diamond-ATR, neat) ν_{max} (cm^{-1}) = 2924, 1623, 1611, 1597, 1528, 1511, 1444, 1304, 1236, 1216, 1178, 1030, 1021, 901, 785, 723, 700.

HRMS (ES-ToF) *m*/*z*: $[M + H]^+$ calc. for $(C_{18}H_{17}N_2O_3)^+$: 309.1234, found: 309.1247.

m.p.: 95 – 97 °C (EtOH) (lit.[4] m.p. 98 – 99 °C).

Anal. Calcd for $C_{18}H_{16}N_2O_3$: C, 70.12; H, 5.23; N; 9.09. Found: C, 68.83; H, 4.95; N, 8.56

The analytic data were in good agreement with literature values.[4]

(4-(Ethoxycarbonyl)-1-(4-methoxybenzyl)-1*H*-pyrazol-5-olate)sodium (11)

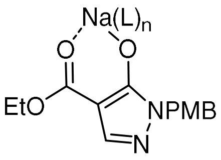

NaOMe (0.5 M in MeOH; 1.62 mL,0.811 mmol, 1.00 equiv) was added with stirring to pyrazolone **8** (224 mg, 0.811 mmol, 1.00 equiv) in MeOH (60 mL) at 65 °C. After heating at reflux for 18 h, the mixture was cooled to room temperature and co-evaporated to dryness with Et_2O (4 x 15 mL). The precipitate was suspended in Et_2O, collected by filtration and dried *in vacuo*. Na-pyrazolone complex **11** (209 mg, 0.701 mmol, 86%) was obtained as a white solid, essentially pure by its ^{1}H NMR spectrum. Crystals suitable for X-ray crystallography were obtained by slow evaporation from a MeOH solution.

^{1}H-NMR (400 MHz, CD_3OD): δ (ppm) = 7.48 (s, 1H), 7.13 (d, *J* = 8.5 Hz, 2H), 6.86 – 6.77 (m, 2H), 4.91 (s, 2H), 4.18 (q, *J* = 7.1 Hz, 2H), 3.74 (s, 3H), 1.30 (t, *J* = 7.1 Hz, 3H).

^{13}C{^{1}H}-NMR (101 MHz, CD_3OD): δ (ppm) = 167.6, 165.2, 160.2, 141.1, 132.2, 129.6, 114.7, 93.8, 59.5, 55.6, 48.1, 15.0.

IR (Diamond-ATR, neat) ν_{max} (cm^{-1}) = 1663, 1653, 1647, 1559, 1508, 1411, 1320, 1243, 1211, 1171, 1094, 1030, 990, 786.

HRMS (ES-ToF) *m/z*: $[M + H + CH_3CN]^+$ calc. for $(C_{16}H_{19}N_3O_4Na)^+$: 340.1268, found: 340.1283.

m.p.: 134 – 136 °C (MeOH).

Anal. Calcd for $C_{14}H_{15}N_2NaO_4{\cdot}H_2O$: C, 53.16; H, 5.42; N, 8.86. Found: C, 52.72; H, 5.03; N, 8.78.

(4-(Ethoxycarbonyl)-1-phenyl-1*H*-pyrazol-5-olate)sodium (12)

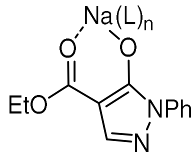

NaOMe in MeOH (0.5 M; 1.71 mL, 0.853 mmol, 1.00 equiv) was added with stirring to pyrazolone **6** (198 mg, 0.853 mmol, 1.00 equiv) in MeOH (5.0 mL) at 65 °C. The mixture was heated at 65 °C for 18 h, after which the mixture was cooled to room temperature, the mixture was co-evaporated to dryness with Et_2O (4 x 15 mL). The precipitate was suspended in Et_2O, collected by filtration and dried *in vacuo*. Na-pyrazolone complex **12** (166 mg, 0.653 mmol, 77%) was obtained as a white solid, essentially pure by its ^{1}H NMR spectrum. Crystals suitable for X-ray crystallography were obtained by slow evaporation from a MeOH solution of complex **12.**

^{1}H-NMR (400 MHz, CD_3OD): δ (ppm) = 7.80 (d, *J* = 8.0 Hz, 2H), 7.65 (s, 1H), 7.37 (t, *J* = 7.8 Hz, 2H), 7.16 (t, *J* = 7.4 Hz, 1H), 4.21 (q, *J* = 7.1 Hz, 2H), 1.32 (t, *J* = 7.1 Hz, 3H).

^{13}C{^{1}H}-NMR (101 MHz, CD_3OD): δ (ppm) = 167.5, 165.6, 142.6, 141.3, 129.5, 125.7, 122.5, 94.3, 59.6, 15.0.

IR (Diamond-ATR, neat) ν_{max} (cm^{-1}) = 1653, 1598, 1560, 1522, 1499, 1454, 1411, 1381, 1352, 1321, 1221, 1191, 1103, 1075, 939, 785, 755, 691.

HRMS (ES-ToF) *m/z*: $[M + H]^+$ calc. for $(C_{12}H_{12}N_2O_3Na)^+$: 255.0740, found: 255.0754. Major peak found at *m/z*: $[M + H + CH_3CN]^+$ calc. for $(C_{14}H_{15}N_3O_3Na)^+$: 296.1006, found: 296.1021.

m.p.: >300 °C (MeOH) (decomp.).

Anal. Calcd for $C_{12}H_{11}N_2O_3Na{\cdot}H_2O$: C, 52.94; H, 4.81; N, 10.29. Found: C, 53.90; H, 4.28; N, 10.14.

(4-Benzoyl-1-(4-methoxybenzyl)-1*H*-pyrazol-5-olate)sodium (13)

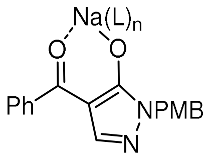

NaOMe (0.5 M in MeOH; 1.19 mL, 0.597 mmol, 1.00 equiv) was added with stirring to pyrazolone **10** (184 mg, 0.597 mmol, 1.00 equiv) in MeOH (3.5 mL) at 65 °C. The mixture was heated at reflux for 14 h, H_2O (20 mL) was added, after which the mixture was cooled to room temperature. The precipitate was collected by filtration, washed with H_2O (3 x 5.0 mL) and dried *in vacuo*. Na-pyrazolone complex **13** (113 mg, 0.342 mmol, 57%) was obtained as a white solid, essentially pure by its ^{1}H NMR spectrum.

^{1}H-NMR (400 MHz, CD_3OD): δ (ppm) = 7.97 – 7.90 (m, 1H), 7.71 – 7.64 (m, 2H), 7.51 – 7.40 (m, 2H), 7.38 (s, 1H), 7.18 (d, *J* = 8.4 Hz, 2H), 6.83 (d, *J* = 8.6 Hz, 2H), 4.93 (s, 2H), 3.75 (s, 3H).

^{13}C{^{1}H}-NMR (101 MHz, CD_3OD): δ (ppm) = 190.9, 165.9, 160.2, 143.0, 142.7, 131.9, 131.3, 129.7, 129.3, 129.0, 114.7, 105.0, 55.6, 48.0.

IR (Diamond-ATR, neat) ν_{max} (cm^{-1}) = 1606, 1575, 1496, 1485, 1430, 1375, 1316, 1241, 1215, 1173, 1024, 891, 803, 743, 705.

HRMS (ES-ToF) *m/z*: [M + H + $CH_3CN]^+$ calc. for $(C_{20}H_{19}N_3O_3Na)^+$: 372.1319, found: 372.1332.

m.p.: >90 °C (MeOH) (decomp.).

Anal. Calcd for $C_{18}H_{15}N_2O_3Na \cdot 1.2\ H_2O$: C, 61.43; H, 4.98; N, 7.96. Found: C, 60.99; H, 4.58; N, 7.79.

(4-(Ethoxycarbonyl)-1*H*-pyrazol-5-olate)sodium (14)

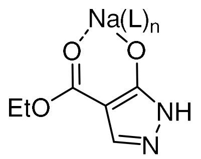

NaOMe (0.5 M in MeOH; 1.81 mL, 0.903 mmol, 1.00 equiv) was added with stirring to pyrazolone **5** (141 mg, 0.903 mmol, 1.00 equiv) in MeOH (5.0 mL) at 65 °C. The mixture was heated at reflux for 30 h, cooled to room temperature and the mixture was co-evaporated to dryness with Et_2O (4 x 15 mL). The precipitate was suspended in Et_2O, collected by filtration and dried *in vacuo*. Na-pyrazolone complex **13** (147 mg, 0.825 mmol, 91%) was obtained as a white solid, essentially pure by its ^{1}H NMR spectrum. Crystals suitable for X-ray crystallography were obtained by slow evaporation from a MeOH solution of complex **14**.

^{1}H-NMR (400 MHz, CD_3OD): δ (ppm) = 7.46 (s, 1H), 4.17 (q, *J* = 7.1 Hz, 2H), 3.35 (s, 1H), 1.29 (t, *J* = 7.1 Hz, 3H).

^{13}C{^{1}H}-NMR (101 MHz, CD_3OD): δ (ppm) = 167.7, 167.4, 142.4, 93.5, 59.5, 15.0.

IR (Diamond-ATR, neat) ν_{max} (cm^{-1}) = 3294, 1664, 1577, 1433, 1327, 1252, 1128, 917, 781, 656.

HRMS (ES-ToF) *m/z*: [M + H + $CH_3CN]^+$ calc. for $(C_8H_{11}N_3O_3Na)^+$: 220.0693, found: 220.0430.

m.p.: >265 °C (MeOH) (decomp.).

Bis(ethanol) bis(4-(ethoxycarbonyl)-1-(4-methoxybenzyl)-1*H*-pyrazol-5-olate)magnesium (15)

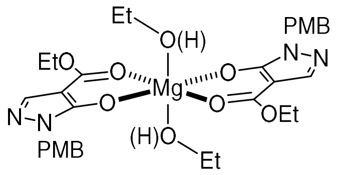

$MgCl_2$ (43.0 mg,0.449 mmol, 1.00 equiv) in EtOH and H_2O (1 : 1; 2.0 mL) was added with stirring to pyrazolone **8** (248 mg,0.987 mmol, 2.00 equiv) in EtOH (3.0 mL) at 79 °C. Aqueous NaOH (2 M; 0.45 mL, 0.987 mmol, 2.00 equiv) was added with stirring and the mixture was heated at reflux for 14 h, cooled to room temperature and the precipitate was collected by filtration and dried *in vacuo*. Mg-pyrazolone complex **15** (225 mg, 0.337 mmol, 75%) was obtained as a white solid, essentially pure by its ^{1}H NMR spectrum. Crystals suitable for X-ray crystallography were obtained by slow evaporation from an EtOH solution of **15**.

^{1}H-NMR (400 MHz, $OC(CD_3)_2$): δ (ppm) = 7.39 (s, 1H), 7.16 (s, 2H), 6.71 (d, *J* = 8.1 Hz, 2H), 4.86 (s, 2H), 4.02 (s, 2H), 3.72 (s, 3H), 3.14 (s, 2H), 1.06 (s, 3H).

^{13}C{^{1}H}-NMR (101 MHz, $OC(CD_3)_2$): δ (ppm) = 170.1, 159.7, 138.4, 131.6, 130.0, 114.3, 93.3, 61.1, 55.4, 48.7, 14.5.

IR (Diamond-ATR, neat) $ν_{max}$ (cm^{-1}) = 1610, 1583, 1567, 1539, 1512, 1441, 1325, 1254, 1234, 1176, 1023, 839, 813.

m.p.: 178 – 182 °C (EtOH); >230 °C (decomp.).

Anal. Calcd for $C_{28}H_{30}MgN_4O_8 \cdot 3\ H_2O$: C, 53.47; H, 5.77; N, 8.91. Found: C, 53.40; H, 5.73; N, 8.39.

Bis(ethanol) bis(4-(ethoxycarbonyl)-1-phenyl-1*H*-pyrazol-5-olate)magnesium (16)

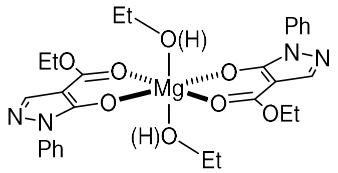

$MgCl_2$ (48.0 mg,0.499 mmol, 1.00 equiv) in EtOH and H_2O (1 : 1; 2.0 mL) was added with stirring to pyrazolone **6** (232 mg, 0.999 mmol, 2.00 equiv) in EtOH (3.0 mL) at 79 °C. Aqueous NaOH (2 M; 0.5 mL, 0.999 mmol, 2.00 equiv) was added with stirring and the mixture was heated at reflux for 15 h, after which the mixture was cooled to room temperature, the precipitate was collected by filtration and dried *in vacuo*. Mg-pyrazolone complex **16** (205 mg, 0.354 mmol, 71%) was obtained as a white solid, essentially pure by its ^{1}H NMR spectrum. Crystals suitable for X-ray crystallography were obtained by slow evaporation from an EtOH solution of complex **16**.

^{1}H-NMR (400 MHz, $OC(CD_3)_2$): δ (ppm) = 8.15 (d, *J* = 8.1 Hz, 2H), 7.57 (s, 1H), 7.33 (t, *J* = 7.8 Hz, 2H), 7.10 (t, *J* = 7.4 Hz, 1H), 4.24 (q, *J* = 7.1 Hz, 2H), 3.63 – 3.53 (m, 2H), 1.28 (t, *J* = 7.1 Hz, 3H), 1.12 (t, *J* = 7.0 Hz, 2H).

^{13}C{^{1}H}-NMR (101 MHz, $OC(CD_3)_2$): δ (ppm) = 170.0, 166.9, 141.6, 139.6, 129.1, 124.6, 119.8, 93.1, 60.6, 58.5, 18.9, 14.8.

IR (Diamond-ATR, neat) $ν_{max}$ (cm^{-1}) = 1680, 1647, 1599, 1566, 1527, 1499, 1465, 1457, 1420, 1360, 1331, 1231, 1208, 1081, 948, 784, 752.

m.p.: 273 – 277 °C (EtOH).

Anal. Calcd for $C_{24}H_{22}MgN_4O_6 \cdot 2$ EtOH $\cdot$ H_2O: C, 56.34; H, 6.08; N, 9.39. Found: C, 56.11; H, 5.39; N, 9.65.

Bis(ethanol) bis(4-benzoyl-1-(4-methoxybenzyl)-1*H*-pyrazol-5-olate)magnesium (17)

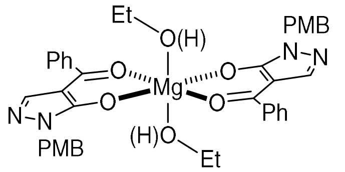

$MgCl_2$ (26.0 mg, 0.271 mmol, 1.00 equiv) was added with stirring to pyrazolone **10** (167 mg, 0.542 mmol, 2.00 equiv) in EtOH (3.0 mL) at 79 °C. Aqueous NaOH (2 M; 0.27 mL, 0.542 mmol, 2.00 equiv) was added with stirring and the mixture was heated at reflux for 16 h, cooled to room temperature and the precipitate was collected by filtration and dried *in vacuo*. Mg-pyrazolone complex **17** (176 mg, 0.241 mmol, 88%) was obtained as a white solid, essentially pure by its ^{1}H NMR spectrum. Crystals suitable for X-ray crystallography were obtained by slow evaporation from an EtOH solution of complex **17**.

^{1}H-NMR (400 MHz, CD_3OD): δ (ppm) = 7.67 (d, *J* = 7.3 Hz, 2H), 7.45 (dd, *J* = 12.9, 7.0 Hz, 3H), 7.36 (s, 1H), 7.17 (d, *J* = 8.1 Hz, 2H), 6.83 (d, *J* = 8.3 Hz, 2H), 4.94 (s, 2H), 3.75 (s, 3H), 3.61 (q, *J* = 7.0 Hz, 5H), 1.18 (t, *J* = 7.0 Hz, 8H).

^{13}C{^{1}H}-NMR (101 MHz, CD_3OD): δ (ppm) = 190.9, 165.9, 160.3, 143.0, 142.7, 131.9, 131.4, 129.7, 129.3, 129.0, 114.7, 104.9, 58.3, 55.7, 48.0, 18.4.

IR (Diamond-ATR, neat) ν_{max} (cm^{-1}) = 1612, 1511, 1491, 1437, 1387, 1324, 1240, 1224, 1176, 1048, 1026, 900, 820, 757, 706.

m.p.: 185 – 190 °C (EtOH).

Bis(ethanol) bis(4-(ethoxycarbonyl)-1*H*-pyrazol-5-olate)magnesium (18)

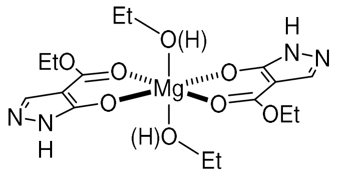

$MgCl_2$ (86.0 mg, 0.900 mmol, 1.00 equiv) in H_2O and EtOH (1 : 2; 3.0 mL) was added with stirring to pyrazolone **5** (281 mg, 1.80 mmol, 2.00 equiv) in EtOH (3.0 mL) at 79 °C. Aqueous NaOH (2 M; 0.90 mL, 1.80 mmol, 2.00 equiv) was added with stirring and the mixture was heated at reflux for 16 h, cooled to room temperature and the precipitate was collected by filtration and dried *in vacuo*. Mg-pyrazolone complex **18** (249 mg, 0.584 mmol, 65%) was obtained as a white solid, essentially pure by its ^{1}H NMR spectrum. Crystals suitable for X-ray crystallography were obtained by slow evaporation from an EtOH solution of complex **18**.

^{1}H-NMR (400 MHz, $OS(CD_3)_2$): δ (ppm) = 10.96 (s, 1H), 7.25 (s, 1H), 4.08 (q, *J* = 7.1 Hz, 2H), 3.35 (s, 4H), 1.17 (t, *J* = 7.1 Hz, 3H).

^{13}C{^{1}H}-NMR (101 MHz, $OS(CD_3)_2$): δ (ppm) = 167.7, 167.5, 137.9, 90.3, 58.8, 14.5.

IR (Diamond-ATR, neat) ν_{max} (cm^{-1}) = 3384, 1648, 1571, 1523, 1511, 1431, 1394, 1327, 1281, 1139, 1115, 1015, 921, 788 762, 648.

m.p.: 269 – 274 °C (EtOH) (decomp.).

Anal. Calcd for $C_{12}H_{14}MgN_4O_6 \cdot 2$ H_2O: C, 38.89; H, 4.9; N, 15.12. Found: C, 38.8; H, 4.49; N, 14.72.

Bis(ethanol) bis(4-(ethoxycarbonyl)-1-(4-methoxybenzyl)-1*H*-pyrazol-5-olate)calcium (19)

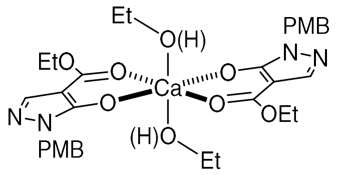

$CaCl_2$ (91.0 mg,0.822 mmol, 1.00 equiv) in H_2O and EtOH (1 : 3; 2.0 mL) was added with stirring to pyrazolone **8** (454 mg, 1.64 mmol, 2.00 equiv) in EtOH (3.0 mL) at 79 °C. Aqueous NaOH (2 M; 0.82 mL, 1.64 mmol, 2.00 equiv) was added with stirring and the mixture was heated at reflux for 15 h, cooled to room temperature and H_2O (10 mL) was added. The precipitate was collected by filtration and dried *in vacuo*. Ca-pyrazolone complex **19** (168 mg, 0.247 mmol, 30%) was obtained as a white solid, essentially pure by its ^{1}H NMR spectrum. Crystals suitable for X-ray crystallography were obtained by slow evaporation from an EtOH solution of complex **19**.
Repeating the reaction with $Ca(OTf)_2$ (210 mg, 0.621 mmol, 1.00 equiv), pyrazolone **8** (343 mg, 1.24 mmol, 2.00 equiv) and aqueous NaOH (2M, 0.62 mL, 1.24 mmol, 2.00 equiv) according to the above procedure resulted in an improved yield of Ca-pyrazolone complex **19** (288 mg, 0.422 mmol, 68%).

^{1}H-NMR (400 MHz, $OC(CD_3)_2$): δ (ppm) = 7.33 (s, 1H), 7.14 (d, *J* = 8.1 Hz, 2H), 6.65 (d, *J* = 8.1 Hz, 2H), 4.90 (s, 2H), 3.93 (q, *J* = 7.3 Hz, 2H), 3.68 (s, 3H), 3.56 (q, *J* = 6.8 Hz, 1H), 1.12 (d, *J* = 7.1 Hz, 2H), 1.08 (d, *J* = 7.1 Hz, 3H).

^{13}C{^{1}H}-NMR (101 MHz, $OC(CD_3)_2$): δ (ppm) = 168.6, 165.2, 159.5, 138.5, 132.1, 130.2, 114.1, 93.0, 59.8, 57.7, 55.4, 48.3, 18.9, 14.8.

IR (Diamond-ATR, neat) ν_{max} (cm^{-1}) = 1638, 1511, 1419, 1464, 1379, 1355, 1318, 1245, 1211,1175, 1030 785.

m.p.: 180 – 185 °C (EtOH), >230 °C (decomp.).

Anal. Calcd for $C_{28}H_{30}CaN_4O_8 \cdot 2\ H_2O$: C, 53.67; H, 5.47; N, 8.94. Found: C, 53.49; H, 5.45; N, 8.31.

(Aqua)(ethanol) bis(4-(ethoxycarbonyl)-1-phenyl-1*H*-pyrazol-5-olate)calcium (20)

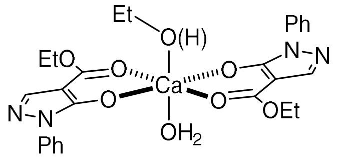

$CaCl_2$ (111 mg,1.00 mmol, 1.00 equiv) in H_2O and EtOH (1 : 2; 3.0 mL) was added with stirring to pyrazolone **6** (464 mg, 2.00 mmol, 2.00 equiv) in EtOH (3.0 mL) at 79 °C. Aqueous NaOH (2 M; 1.0 mL, 2.00 mmol, 2.00 equiv) was added with stirring and the mixture was heated at reflux for 14 h, cooled to room temperature and the precipitate was collected by filtration and dried *in vacuo*. Ca-pyrazolone complex **20** (173 mg,0.305 mmol, 31%) was obtained as a white solid, essentially pure by its ^{1}H NMR spectrum. Crystals suitable for X-ray crystallography were obtained by slow evaporation from an EtOH solution of complex **20**.

^{1}H-NMR (400 MHz, $OC(CD_3)_2$): δ (ppm) = 8.15 (d, *J* = 7.9 Hz, 4H), 7.54 (d, *J* = 1.0 Hz, 2H), 7.29 (t, *J* = 7.8 Hz, 4H), 7.05 (td, *J* = 7.4, 1.3 Hz, 2H), 4.23 – 4.13 (m, 4H), 3.57 (q, *J* = 7.0 Hz, 1H), 3.00 (s, 3H), 1.25 (td, *J* = 7.1, 1.0 Hz, 6H), 1.12 (td, *J* = 7.0, 1.0 Hz, 2H).

^{13}C{^{1}H}-NMR (101 MHz, $OC(CD_3)_2$): δ (ppm) = 168.6, 166.6, 141.9, 140.1, 129.0, 124.1, 119.7, 93.4, 59.8, 57.8, 19.2, 14.9.

IR (Diamond-ATR, neat) ν_{max} (cm^{-1}) = 1653, 1599, 1565, 1543, 1523, 1498, 1420, 1372, 1357, 1325, 11227, 1204, 1081, 951, 751.

m.p.: >270 °C (EtOH) (decomp.).

Anal. Calcd for $C_{24}H_{22}CaN_4O_6 \cdot 2\ H_2O \cdot$ EtOH: C, 53.42; H, 5.52; N, 9.58. Found: C, 53.02; H, 4.70; N, 9.74.

Bis(ethanol) bis(4-benzoyl-1-(4-methoxybenzyl)-1*H*-pyrazol-5-olate)calcium (21)

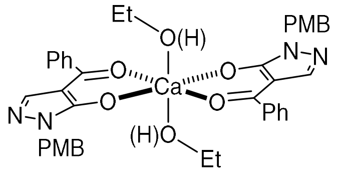

$Ca(OTf)_2$ (85.0 mg, 0.251 mmol, 1.00 equiv) was added with stirring to pyrazolone **10** (155 mg, 0.503 mmol, 2.00 equiv) in EtOH (3.0 mL) at 79 °C. Aqueous NaOH (2 M; 0.25 mL, 0.503 mmol, 2.00 equiv) was added with stirring and the mixture was heated at reflux for 17 h, cooled to room temperature and the precipitate was collected by filtration and dried *in vacuo*. Ca-pyrazolone complex **21** (101 mg, 0.135 mmol, 54%) was obtained as a white solid, essentially pure by its ^{1}H NMR spectrum. Crystals suitable for X-ray crystallography were obtained by slow evaporation from an EtOH solution of complex **21**.

^{1}H-NMR (400 MHz, $OC(CD_3)_2$): δ (ppm) = 7.65 (d, *J* = 7.5 Hz, 2H), 7.47 (t, *J* = 7.3 Hz, 1H), 7.40 (d, *J* = 7.6 Hz, 2H), 7.37 (s, 1H), 7.18 (d, *J* = 8.3 Hz, 2H), 6.62 (d, *J* = 8.1 Hz, 2H), 4.91 (s, 2H), 3.66 (s, 3H), 3.57 (p, *J* = 6.7 Hz, 2H), 1.12 (t, *J* = 7.0 Hz, 3H).

$^{13}C\{^1H\}$-NMR (101 MHz, $OC(CD_3)_2$): δ (ppm) = 187.9, 166.1, 159.5, 141.9, 141.0, 132.1, 131.0, 130.1, 129.3, 128.8, 114.2, 105.1, 57.7, 55.4, 47.9, 18.9.

IR (Diamond-ATR, neat) ν_{max} (cm^{-1}) = 1610, 1501, 1487, 1435, 1383, 1353, 1304, 1238, 1224, 1046, 1032, 897, 794, 744, 706.

m.p.: 193 – 195 °C (EtOH).

Tris((4-(ethoxycarbonyl)-1-(4-methoxybenzyl)-1*H*-pyrazol-5-yl)oxy)scandium (23)

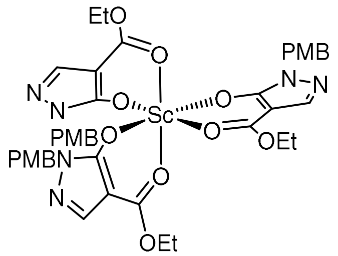

$Sc(OTf)_3$ (200 mg, 0.407 mmol, 1.00 equiv) was added with stirring to pyrazolone **8** (337 mg, 1.22 mmol, 3.00 equiv) in EtOH (3.0 mL) at 79 °C. The mixture was heated at reflux for 16 h, cooled to room temperature and H_2O (10 mL) was added. The precipitate was collected by filtration, washed with H_2O (3 x 10 mL) and dried *in vacuo*. Sc-pyrazolone complex **23** (167 mg, 0.192 mmol, 47%) was obtained as a white solid, essentially pure by its ^{1}H NMR spectrum.

^{1}H-NMR (400 MHz, $CDCl_3$): δ (ppm) = 7.59 (s, 1H), 7.15 (d, *J* = 8.1 Hz, 2H), 6.66 (d, *J* = 8.2 Hz, 2H), 4.93 (s, 2H), 4.21 (q, *J* = 7.1 Hz, 2H), 3.69 (s, 3H), 1.24 (t, *J* = 7.0 Hz, 3H).

$^{13}C\{^1H\}$-NMR (101 MHz, $CDCl_3$): δ (ppm) = 170.2, 163.0, 159.1, 138.0, 129.6, 129.1, 114.0, 94.1, 62.5, 55.3, 48.8, 14.3.

IR (Diamond-ATR, neat) ν_{max} (cm^{-1}) = 2929, 1599, 1525, 1510, 1465, 1437, 1320, 1244, 1224, 1174, 1025.

HRMS (ES-ToF) *m/z*: $[M + H]^+$ calc. for $(C_{42}H_{46}N_6O_{12}Sc)^+$: 871.2727, found: 871.2744.

m.p.: 152 – 155 °C (EtOH).

Anal. Calcd for $C_{42}H_{45}N_6O_{12}Sc \cdot H_2O \cdot EtOH$: C, 56.53; H, 5.71; N, 8.99. Found: C, 56.57; H, 5.32; N, 8.79.

Tris((4-(ethoxycarbonyl)-1-phenyl-1*H*-pyrazol-5-yl)oxy)scandium (24)

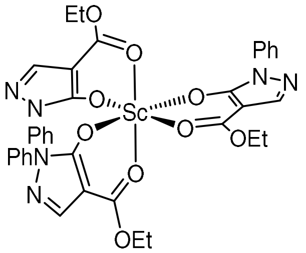

$Sc(OTf)_3$ (124 mg, 0.253 mmol, 1.00 equiv) was added with stirring to pyrazolone **6** (176 mg, 0.758 mmol, 3.00 equiv) in EtOH (3.0 mL) at 79 °C. The mixture was heated at reflux for 16 h, cooled to room temperature and H_2O (10 mL) was added. The precipitate was collected by filtration, washed with H_2O (3 x 10 mL) and dried *in vacuo*. Sc-pyrazolone complex **24** (99.0 mg, 0.134 mmol, 53%) was obtained as a white solid, essentially pure by its ^1H NMR spectrum. Crystals suitable for X-ray crystallography were obtained by slow evaporation from an EtOH solution of complex **24**.

^{1}H-NMR (400 MHz, $CDCl_3$): δ (ppm) = 7.88 (d, *J* = 8.0 Hz, 2H), 7.75 (s, 1H), 7.34 (t, *J* = 7.8 Hz, 2H), 7.19 (t, *J* = 7.4 Hz, 1H), 4.33 (q, *J* = 7.1 Hz, 2H), 1.35 (t, *J* = 7.1 Hz, 3H).

^{13}C{^{1}H}-NMR (101 MHz, $CDCl_3$): δ (ppm) = 170.5, 163.5, 139.1, 138.8, 128.9, 125.9, 120.7, 95.1, 62.8, 14.3.

IR (Diamond-ATR, neat) ν_{max} (cm^{-1}) = 2921, 1662, 1621, 1588, 1534, 1498, 1490, 1457, 1434, 1381, 1327, 1250, 1118, 1083, 955, 757.

HRMS (ES-ToF) *m/z*: $[M + H]^+$ calc. for $(C_{36}H_{34}N_6O_9Sc)^+$: 739.1941, found: 739.1961.

m.p.: 170 – 174 °C (EtOH).

Anal. Calcd for $C_{36}H_{33}N_6O_9Sc \cdot Et_2O$: C, 59.11; H, 5.33; N, 10.34. Found: C, 59.83; H, 5.21; N, 10.05.

Tris((4-benzoyl-1-(4-methoxybenzyl)-1*H*-pyrazol-5-yl)oxy)scandium ethanol dimer (25)

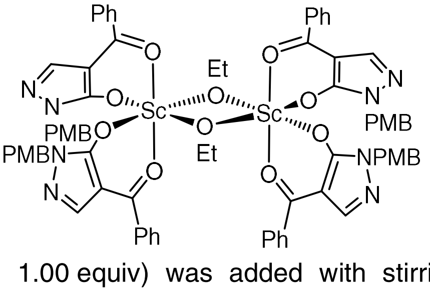

$Sc(OTf)_3$ (84.0 mg, 0.171 mmol, 1.00 equiv) was added with stirring to pyrazolone **10** (158 mg, 0.512 mmol, 3.00 equiv) in EtOH (3.5 mL). Et_3N (52.0 mg, 0.07 mL, 0.512 mmol, 3.00 equiv) was added and the mixture was heated at reflux for 3 h, cooled to room temperature and the precipitate was collected by filtration and dried *in vacuo*. Sc-pyrazolone complex **25** (132 mg, 0.09 mmol, 55%) was obtained as a white solid, essentially pure by its ^1H NMR spectrum. Crystals suitable for X-ray crystallography were obtained by slow evaporation from an EtOH solution of complex **25**.

^{1}H-NMR (400 MHz, $CDCl_3$): δ (ppm) = 7.76 (d, *J* = 7.7 Hz, 3H), 7.71 (s, 1H), 7.57 – 7.51 (m, 1H), 7.43 (t, *J* = 7.6 Hz, 2H), 7.17 (d, *J* = 8.2 Hz, 2H), 6.63 – 6.56 (m, 2H), 4.98 (s, 2H), 3.72 (d, *J* = 7.2 Hz, 2H), 3.65 (s, 3H), 1.23 (t, *J* = 7.0 Hz, 3H).

^{13}C{^{1}H}-NMR (101 MHz, $CDCl_3$): δ (ppm) = 187.6, 164.1, 159.1, 141.0, 137.0, 132.6, 129.7, 129.5, 129.0, 128.7, 114.0, 106.8, 58.6, 55.3, 48.7, 18.6.

IR (Diamond-ATR, neat) ν_{max} (cm^{-1}) = 1611, 1592, 11570, 1510, 1472, 1446, 1394, 1312, 1302, 1243, 1176, 1047, 1023, 900, 807, 795, 764, 752, 705.

HRMS (ES-ToF) *m/z*: $[M + H]^+$ calc. for $(C_{54}H_{46}N_6O_9Sc)^+$: 967.2880, found: 967. 2892.

m.p.: 174 – 176 °C (EtOH).

Anal. Calcd for $C_{76}H_{70}N_8O_{14}Sc_2$: C, 64.77; H, 5.01; N, 7.95. Found: C, 63.96; H, 4.91; N, 7.90.

Tris((4-(ethoxycarbonyl)-1*H*-pyrazol-5-yl)oxy)scandium (26)

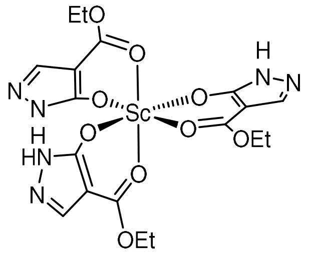

$Sc(OTf)_3$ (143 mg, 0.290 mmol, 1.00 equiv) was added with stirring to pyrazolone **5** (136 mg, 0.871 mmol, 3.00 equiv) in EtOH (3.5 mL) at 79 °C. NaOMe (0.5 M in MeOH; 1.75 mL, 0.871 mmol, 3.00 equiv) was added and the mixture was heated at reflux for 15 h, after which the mixture was cooled to room temperature, H_2O was added (20 mL), the precipitate was collected by filtration and dried *in vacuo*. Sc-pyrazolone complex **26** (125 mg, 0.240 mmol, 84%) was obtained as a white solid, essentially pure by its ^{1}H NMR spectrum.

^{1}H-NMR (400 MHz, $(CD_3)_2SO$): δ (ppm) = 11.49 (s, 1H), 7.41 (s, 1H), 4.37 (t, *J* = 5.0 Hz, 1H), 4.12 (q, *J* = 7.1 Hz, 2H), 3.49 – 3.38 (m, 1H), 1.18 (t, *J* = 7.0 Hz, 4H), 1.05 (t, *J* = 7.0 Hz, 2H).

^{13}C{^{1}H}-NMR (101 MHz, $(CD_3)_2SO$): δ (ppm) = 166.3, 163.6, 149.6, 138.9, 92.2, 59.6, 56.1, 18.6, 14.4.

IR (Diamond-ATR, neat) ν_{max} (cm^{-1}) = 3335, 1690, 1623, 1588, 1558, 1522, 1457, 1320, 1135, 1111, 932, 786, 775.

HRMS (ES-ToF) *m/z*: $[M + H]^+$ calc. for $(C_{18}H_{22}N_6O_9Sc)^+$: 511.1002, found: 511.1014.

m.p.: >275 °C (EtOH) (decomp.).

Anal. Calcd for $C_{18}H_{21}N_6O_9Sc \cdot Et_2O$: C, 45.21; H, 5.35; N, 14.38. Found: C, 45.51; H, 5.36; N, 13.34.

Tris((4-(ethoxycarbonyl)-1-(4-methoxybenzyl)-1*H*-pyrazol-5-yl)oxy)yttrium (27)

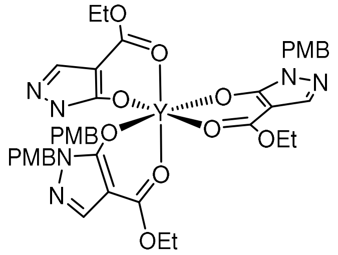

YCl_3•$6H_2O$ (85.0 mg, 0.280 mmol, 1.00 equiv) was added with stirring to pyrazolone **8** (232 mg, 0.840 mmol, 3.00 equiv) in EtOH (4.0 mL) at 79 °C. Aqueous NaOH (2 M; 0.42 mL, 0.840 mmol, 3.00 equiv) was added with stirring and the mixture was heated at reflux for 17 h, cooled to room temperature, when H_2O (15 mL) was added, the precipitate was collected by filtration, washed with H_2O (3 x 10 mL) and dried *in vacuo*. Y-pyrazolone complex **27** (96 mg, 0.105 mmol, 37%) was obtained as a white solid, essentially pure by its ^{1}H NMR spectrum.

^{1}H-NMR (400 MHz, CD_3OD): δ (ppm) = 7.45 (s, 1H), 7.10 (d, *J* = 8.1 Hz, 2H), 6.63 (d, *J* = 8.1 Hz, 2H), 4.83 (s, 2H), 4.00 (q, *J* = 7.2 Hz, 2H), 3.65 (s, 3H), 1.12 (t, *J* = 7.2 Hz, 3H).

^{13}C{^{1}H}-NMR (101 MHz, CD_3OD): δ (ppm) = 169.5, 165.4, 160.2, 138.8, 131.4, 130.1, 114.6, 94.3, 61.4, 55.6, 14.8.

IR (Diamond-ATR, neat) ν_{max} (cm^{-1}) = 2926, 1617, 1509, 1431, 1318, 1243, 1216, 1191, 1174, 1090, 1027, 782.

HRMS (ESI) *m*/*z*: [M + H]$^+$ calc. for $(C_{42}H_{46}N_6O_{12}Y)^+$: 915.2227, found: 915.2232.

m.p.: 120 – 124 °C (EtOH); >220 °C (decomp.).

Anal. Calcd for $C_{46}H_{57}N_6O_{14}Y$: C, 54.87; H, 5.71; N, 8.35. Found: C, 54.17; H, 5.24; N, 8.44.

Tris((4-(ethoxycarbonyl)-1-phenyl-1*H*-pyrazol-5-yl)oxy)yttrium (28)

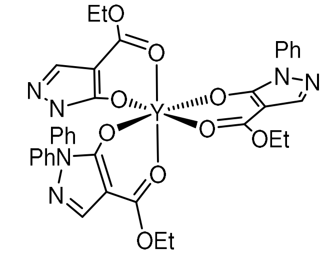

YCl_3•$6H_2O$ (88.0 mg, 0.291 mmol, 1.00 equiv) was added with stirring to pyrazolone **6** (203 mg, 0.874 mmol, 3.00 equiv) in EtOH (4.0 mL) at 79 °C. Aqueous NaOH (2 M; 0.44 mL, 0.874 mmol, 3.00 equiv) was added with stirring and the mixture was heated at reflux for 18 h, cooled to room temperature and the precipitate was collected by filtration and dried *in vacuo*. Y-pyrazolone complex **28** (131 mg, 0.167 mmol, 58%) was obtained as a white solid, essentially pure by its ^{1}H NMR spectrum. Crystals suitable for X-ray crystallography were obtained by slow evaporation from an EtOH solution of complex **28**.

^{1}H-NMR (400 MHz, $(CD_3)_2CO$): δ (ppm) = 8.12 – 8.05 (m, 2H), 7.59 (s, 1H), 7.25 (t, *J* = 7.9 Hz, 2H), 7.12 – 7.04 (m, 1H), 4.24 (q, *J* = 7.1 Hz, 2H), 3.83 (s, 1H), 1.24 (t, *J* = 7.1 Hz, 3H).

^{13}C{^{1}H}-NMR (101 MHz, $(CD_3)_2CO$): δ (ppm) = 169.2, 165.7, 141.0, 139.4, 129.2, 125.0, 120.2, 94.4, 61.1, 14.9.

IR (Diamond-ATR, neat) ν_{max} (cm^{-1}) = 1641, 1597, 1554, 1528, 1498, 1457, 1429, 1359, 1334, 1237, 1211, 1118, 1083, 951, 784, 752.

HRMS (ESI) *m/z*: $[M + H]^+$ calc. for $(C_{36}H_{34}N_6O_9Y)^+$: 783.1440, found: 783.1442.

m.p.: 148 – 150 °C (EtOH); 175 – 177 °C (EtOH); >200 °C (decomp.).

Anal. Calcd for $C_{40}H_{45}N_6O_{11}Y$: C, 54.92; H, 5.19; N, 9.61. Found: C, 53.23; H, 4.93; N, 9.30.

Bis(methoxy) bis(4-(ethoxycarbonyl)-1-(4-methoxybenzyl)-1*H*-pyrazol-5-olate)titanium (29)

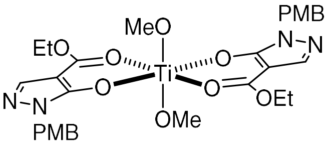

$Ti(OMe)_4$ (57.0 mg, 0.329 mmol, 1.00 equiv) was added with stirring to pyrazolone **8** (182 mg, 0.659 mmol, 2.00 equiv) in PhH (3.5 mL) at 80 °C. The mixture was heated at reflux for 16 h and subsequently cooled to room temperature. An Et_2O and hexanes mixture (1 : 2; 30 mL) was added, the precipitate was collected by filtration and dried *in vacuo*. Ti-pyrazolone complex **29** (61 mg, 0.0924 mmol, 28%) was obtained as a red solid.

^{1}H-NMR (400 MHz, CD_3OD): δ (ppm) = 7.42 (d, *J* = 8.3 Hz, 2H), 7.26 (s, 1H), 6.62 (d, *J* = 8.2 Hz, 2H), 4.75 – 4.50 (m, 2H), 3.70 – 3.62 (m, 2H), 3.60 (s, 4H), 3.48 – 3.37 (m, 2H), 0.82 (t, *J* = 7.1 Hz, 3H).

^{13}C{^{1}H}-NMR (101 MHz, CD_3OD): δ (ppm) = 168.9, 162.4, 159.1, 137.2, 130.6, 129.3, 114.0, 113.6, 95.5, 62.0, 55.1, 49.1, 13.9.

IR (Diamond-ATR, neat) ν_{max} (cm^{-1}) = 1606, 1532, 1512, 1438, 1321, 1244, 1226, 1030, 822, 783.

HRMS (ES-ToF) *m/z*: $[M + H]^+$ calc. for $(C_{30}H_{37}N_4O_{10}Ti)^+$: 661.1984, found: 661.1987.

m.p.: 88 – 90 °C (Et_2O/hexanes).

Anal. Calcd for $C_{30}H_{36}N_4O_{10}Ti \cdot 0.7\ TiO_2$: C, 50.30; H, 5.07; N, 7.82. Found: C, 50.28; H, 4.56; N, 8.13.

Bis(methoxy) bis(4-(ethoxycarbonyl)-1-phenyl-1*H*-pyrazol-5-olate)titanium (30)

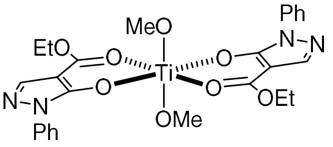

$Ti(OMe)_4$ (63.0 mg, 0.368 mmol, 1.00 equiv) was added with stirring to pyrazolone **6** (171 mg, 0.736 mmol, 2.00 equiv) in PhH (3.0 mL) at 80 °C. The mixture was heated at reflux for 16 h and subsequently cooled to room temperature. A mixture of Et_2O hexanes (1 : 2; 30 mL) was added, the precipitate was collected by filtration and dried *in vacuo*. Ti-pyrazolone complex **30** (27.0 mg, 0.0472 mmol, 13%) was obtained as a red-orange/brown solid.

^{1}H-NMR (400 MHz, CD_3OD): δ (ppm) = 7.93 (s, 2H), 7.80 (s, 1H), 7.52 (s, 2H), 7.36 (s, 1H), 4.19 (s, 2H), 1.22 (s, 3H).
^{13}C{^{1}H}-NMR (126 MHz, CD_3OD): δ (ppm) = 170.5, 164.6, 139.8, 139.4, 129.8, 127.1, 122.3, 95.6, 63.5, 49.9, 14.7.

IR (Diamond-ATR, neat) ν_{max} (cm^{-1}) = 1610, 1595, 1586, 1530, 1508, 1499, 1457, 1431, 1384, 1323, 1255, 1118, 1084, 956, 823, 772, 756.

HRMS (ES-ToF) *m/z*: $[M + H]^+$ calc. for $(C_{26}H_{29}N_4O_8Ti)^+$: 573.1459, found: 573.1443.

m.p.: 140 – 142 °C (Et_2O/hexanes).

Anal. Calcd for $C_{26}H_{28}N_4O_8Ti \cdot 2\ TiO_2$: C, 42.65; H, 3.86; N, 7.65. Found: C, 42.70; H, 3.78; N, 7.77.

Bis(methoxy) bis(4-benzoyl-1-(4-methoxybenzyl)-1*H*-pyrazol-5-olate)titanium (31)

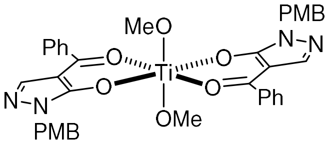

$Ti(OMe)_4$ (41.0 mg, 0.238 mmol, 1.00 equiv) was added with stirring to pyrazolone **10** (147 mg, 0.477 mmol, 2.00 equiv) in PhH (3.5 mL) at 80 °C. The mixture was heated at reflux for 16 h and subsequently cooled to room temperature. A mixture of Et_2O and hexanes (1 : 2:30 mL) was added and the precipitate was collected by filtration and dried *in vacuo*. Ti-pyrazolone complex **31** (93 mg, 0.128 mmol, 54%) was obtained as a red solid.

^{1}H-NMR (400 MHz, $CDCl_3$): δ (ppm) = 7.59 (d, *J* = 8.3 Hz, 2H), 7.40 (s, 1H), 7.27 – 7.21 (m, 2H), 6.95 (s, 1H), 6.88 (d, *J* = 7.9 Hz, 2H), 6.40 (d, *J* = 8.3 Hz, 2H), 4.95 (d, *J* = 14.6 Hz, 1H), 4.77 (d, *J* = 14.6 Hz, 1H), 3.25 (s, 3H).

^{13}C{^{1}H}-NMR (101 MHz, $CDCl_3$): δ (ppm) = 186.5, 162.9, 159.0, 140.0, 135.9, 132.3, 130.8, 129.4, 129.1, 128.3, 113.9, 107.2, 54.8, 49.1.

IR (Diamond-ATR, neat) ν_{max} (cm^{-1}) = 1580, 1564, 1511, 1477, 1438, 1395, 1312, 1303, 1243, 1174, 1033, 904, 783, 764, 744.

HRMS (ES-ToF) *m*/*z*: $[M - MeOH]^+$ calc. for $(C_{37}H_{33}N_4O_7Ti)^+$: 693.1823, found: 693.0573.

m.p.: 153 – 155 °C (Et_2O/hexanes).

Anal. Calcd for $C_{38}H_{36}N_4O_8Ti \cdot 0.85\ TiO_2$: C, 57.59; H, 4.58; N, 7.07. Found: C, 57.84; H, 4.21; N, 7.40.

Bis(methoxy) bis(4-(ethoxycarbonyl)-1*H*-pyrazol-5-olate)titanium (32)

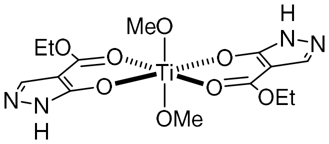

$Ti(OMe)_4$ (80.0 mg, 0.464 mmol, 1.00 equiv) was added with stirring to pyrazolone **5** (145 mg, 0.929 mmol, 2.00 equiv) in PhH (3.5 mL) at 80 °C. The mixture was heated at reflux for 16 h and subsequently cooled to room temperature. A mixture of Et_2O and hexanes (1 : 1; 30 mL) was added, the precipitate was collected by filtration and dried *in vacuo*. Ti-pyrazolone complex **32** (133 mg, 0.317 mmol, 68%) was obtained as a red-brown solid.

^{1}H-NMR (400 MHz, CD_3OD): δ (ppm) = 7.59 (s, 1H), 4.14 (s, 2H), 1.19 (t, *J* = 8.0 Hz, 3H).

^{13}C{^{1}H}-NMR (101 MHz, CD_3OD): δ (ppm) = 170.8, 166.7, 139.2, 95.4, 63.5, 14.5.

IR (Diamond-ATR, neat) ν_{max} (cm^{-1}) = 3178, 1686, 1540, 1500, 1374, 1333, 1174, 1100, 924, 847, 777.

m.p.: >200 °C (decomp.).

Anal. Calcd for $C_{14}H_{20}N_4O_8Ti \cdot 1.28\ TiO_2$: C, 32.19; H, 3.86; N, 10.72. Found: C, 32.19; H, 3.39; N, 11.93.

Tetrakis((4-(ethoxycarbonyl)-1-(4-methoxybenzyl)-1*H*-pyrazol-5-yl)oxy)zirconium (33)

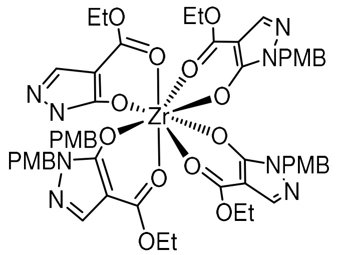

$ZrCl_4$•2THF (52.0 mg, 0.138 mmol, 1.00 equiv) was added with stirring pyrazolone **8** (153 mg, 0.554 mmol, 4.00 equiv) in EtOH (3.0 mL) at 79 °C. The mixture was heated at reflux for 14 h, cooled to room temperature, and the precipitate was collected by filtration and dried *in vacuo*. Zr-pyrazolone complex **33** (76.0 mg, 0.0637 mmol, 46%) was obtained as a white solid, essentially pure by its ^{1}H NMR spectrum. Crystals suitable for X-ray crystallography were obtained by slow evaporation from an EtOH solution of complex **33**.

^{1}H-NMR (400 MHz, $OC(CD_3)_2$): δ (ppm) = 7.48 (s, 1H), 7.30 – 7.20 (m, 2H), 6.75 – 6.67 (m, 2H), 4.92 (s, 2H), 3.74 (t, *J* = 7.1 Hz, 2H), 3.69 (s, 2H), 0.98 (t, *J* = 7.1 Hz, 3H).

^{1}H-NMR (400 MHz, $CDCl_3$): δ (ppm) = 7.53 (s, 1H), 7.23 – 7.20 (m, 2H), 6.66 – 6.63 (m, 2H), 4.90 (s, 2H), 3.68 (s, 3H), 3.63 (q, *J* = 7.1 Hz, 2H), 0.98 (t, *J* = 7.1 Hz, 3H).

^{13}C{^{1}H}-NMR (101 MHz, $OC(CD_3)_2$): δ (ppm) = 168.6, 163.1, 160.1, 137.2, 130.5, 130.4, 114.4, 94.6, 62.3, 55.5, 49.5, 14.5.

^{13}C{^{1}H}-NMR (101 MHz, $CDCl_3$): δ (ppm) = 167.9, 162.2, 159.2, 137.4, 129.8, 129.3, 113.9, 94.1, 61.7, 55.3, 49.1, 14.2.

IR (Diamond-ATR, neat) ν_{max} (cm^{-1}) = 1607, 1535, 1511, 1438, 1424, 1385, 1354, 1318, 1245, 1222, 1194, 1174, 1088, 1030, 805, 783, 760 742.

HRMS (ES-ToF) *m/z*: $[M + H]^+$ calc. for $(C_{56}H_{61}N_8O_{16}Zr)^+$: 1191.3247, found: 1191.3220.

m.p.: 150 – 152 °C (EtOH).

Anal. Calcd for $C_{56}H_{60}N_8O_{16}Zr \cdot C_6H_{14}$: C, 58.24; H, 5.83; N, 8.76. Found: C, 58.64; H, 5.70; N, 8.43.

Tetrakis((4-(ethoxycarbonyl)-1-phenyl-1*H*-pyrazol-5-yl)oxy)zirconium (34)

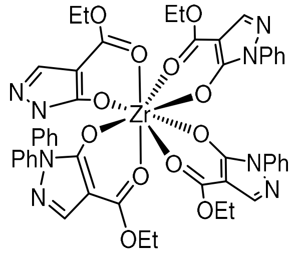

$ZrCl_4$•2THF (95.0 mg, 0.253 mmol, 1.00 equiv) was added with stirring to pyrazolone **6** (235 mg, 1.01 mmol, 4.00 equiv) in EtOH (3.0 mL) at 79 °C. The mixture was heated at reflux for 14 h, cooled to room temperature, and the precipitate was collected by filtration and dried *in vacuo*. Zr-pyrazolone complex **34** (237 mg, 0.233 mmol, 93%) was obtained as a white solid, essentially pure by its ^{1}H NMR spectrum. Crystals suitable for X-ray crystallography were obtained by slow evaporation from an EtOH solution of complex **34**.

^{1}H-NMR (400 MHz, $OC(CD_3)_2$): δ (ppm) = 8.00 (dd, *J* = 7.7, 1.9 Hz, 2H), 7.74 (s, 1H), 7.24 (dd, *J* = 8.5, 6.8 Hz, 2H), 7.18 (dd, *J* = 8.4, 6.0 Hz, 1H), 4.11 (q, *J* = 7.1 Hz, 2H), 1.13 (t, *J* = 7.1 Hz, 3H).

^{1}H-NMR (400 MHz, $CDCl_3$): δ (ppm) = 7.97 – 7.93 (m, 2H), 7.71 (s, 1H), 7.20 – 7.12 (m, 3H), 3.95 (q, *J* = 7.1 Hz, 2H), 1.09 (t, *J* = 7.1 Hz, 3H).

^{13}C{^{1}H}-NMR (101 MHz, $OC(CD_3)_2$): δ (ppm) = 169.2, 163.4, 139.6, 138.8, 129.6, 126.6, 121.0, 95.9, 62.9, 14.5.

^{13}C{^{1}H}-NMR (101 MHz, $CDCl_3$): δ (ppm) = 168.2, 162.5, 138.6, 138.4, 128.7, 125.9, 120.6, 95.4, 62.1, 14.3.

IR (Diamond-ATR, neat) ν_{max} (cm^{-1}) = 1617, 1595, 1587, 1521, 1498, 1429, 1321, 1255, 1118, 1084, 956, 778, 752.

HRMS (ES-ToF) *m/z*: $[M + H]^+$ calc. for $(C_{48}H_{45}N_8O_{12}Zr)^+$:, found: 1015.2214.

m.p.: 227 – 231 °C (EtOH).

Anal. Calcd for $C_{48}H_{44}N_8O_{12}Zr$: C, 56.74; H, 4.36; N, 11.03. Found: C, 56.83; H, 4.31; N, 10.97.

Tetrakis((4-benzoyl-1-(4-methoxybenzyl)-1*H*-pyrazol-5-yl)oxy)zirconium (35)

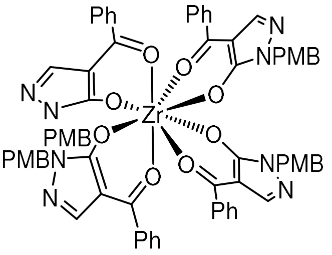

$ZrCl_4$•2THF (38.0 mg, 0.0997 mmol, 1.00 equiv) was added with stirring to pyrazolone **10** (123 mg, 0.399 mmol, 4.00 equiv) in EtOH (3.5 mL) at 79 °C. The mixture was heated at reflux for 48 h, cooled to room temperature, and the mixture was co-evaporated to dryness with Et_2O (4 x 15 mL). The precipitate was suspended in Et_2O, collected by filtration and dried *in vacuo*. Zr-pyrazolone complex **35** (64.0 mg, 0.0485 mmol, 48%) was obtained as a white solid, essentially pure by its ^{1}H NMR spectrum.

^{1}H-NMR (400 MHz, CD_3OD): δ (ppm) = 7.86 (s, 1H), 7.60 (d, *J* = 7.2 Hz, 3H), 7.38 (t, *J* = 7.6 Hz, 2H), 7.08 (d, *J* = 8.1 Hz, 2H), 6.55 (d, *J* = 8.0 Hz, 2H), 4.88 (s, 2H), 3.63 (s, 3H).

^{13}C{^{1}H}-NMR (126 MHz, CD_3OD): δ (ppm) = 187.1, 164.0, 160.7, 141.6, 136.7, 134.7, 130.6, 130.3, 130.1, 129.4, 114.9, 107.7, 55.6, 49.7.

IR (Diamond-ATR, neat) ν_{max} (cm^{-1}) = 1592, 1569, 1512, 1482, 1448, 1314, 1304, 1245, 1176, 904, 807, 748.

HRMS (APCI) *m/z*: $[M + H]^+$ calc. for $(C_{72}H_{61}N_8O_{12}Zr)^+$: 1319.3450, found: 1319.3403.

m.p.: 100 – 102 °C (EtOH).

Anal. Calcd for $C_{72}H_{60}N_8O_{12}Zr \cdot 4\ NaCl \cdot EtOH$: C, 55.54; H, 4.16; N, 7.00. Found: C, 55.61; H, 4.13; N, 7.06.

Tetrakis((4-(ethoxycarbonyl)-1*H*-pyrazol-5-yl)oxy)zirconium (36)

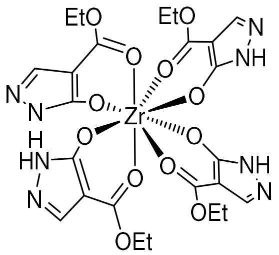

$ZrCl_4$•2THF (77.0 mg, 0.205 mmol, 1.00 equiv) was added with stirring to pyrazolone **5** (128 mg, 0.820 mmol, 4.00 equiv) in EtOH (3.0 mL) at 79 °C. The mixture was heated at reflux for 48 h, cooled to room temperature, and the mixture was co-evaporated to dryness with Et_2O (4 x 15 mL). The precipitate was suspended in Et_2O, collected by filtration and dried *in vacuo*. Zr-pyrazolone complex **36** (102 mg, 0.143 mmol, 70%) was obtained as a white solid, essentially pure by its ^{1}H NMR spectrum

^{1}H-NMR (400 MHz, CD_3OD): δ (ppm) = 8.06 (s, 1H), 4.25 (q, *J* = 7.1 Hz, 2H), 1.30 (t, *J* = 7.1 Hz, 3H).

^{13}C{^{1}H}-NMR (126 MHz, CD_3OD): δ (ppm) = 166.9, 159.2, 137.7, 98.1, 58.3, 18.4.

IR (Diamond-ATR, neat) ν_{max} (cm^{-1}) = 2974, 1617, 1545, 1507, 1437, 1419, 1334, 1103, 779, 762.

m.p.: >206 °C (EtOH) (decomp.).

Anal. Calcd for $C_{24}H_{28}N_8O_{12}Zr \cdot 3\ NaCl \cdot 3\ H_2O$: C, 30.63; H, 3.64; N, 11.91. Found: C, 30.68; H, 3.68; N, 12.09.

Cyclopentadienyl-bis((4-(ethoxycarbonyl)-1-(4-methoxybenzyl)-1*H*-pyrazol-5-yl)oxy)zirconium chloride (37)

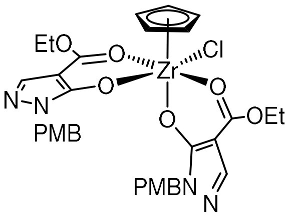

Cp_2ZrCl_2 (147 mg, 0.501 mmol, 1.00 equiv) was added with stirring to pyrazolone **8** (277 mg, 1.00 mmol, 2.00 equiv) and pyridine (79.0 mg, 0.08 mL, 1.00 mmol, 2.00 equiv) in THF (10 mL) at 65 °C. The mixture was heated at reflux for 16 h, was cooled to room temperature, diluted with CH_2Cl_2 (10 mL) and a mixture of Et_2O and hexanes (1 : 1; 30 mL) was added. The precipitated pyridine hydrochloride was removed by filtration and the filtrate was evaporated. Recrystallization of the residue from PhMe and hexanes gave Zr-pyrazolone complex **37** (214 mg, 0.288 mmol, 57%) as a white solid.

^{1}H-NMR (400 MHz, $CDCl_3$): δ (ppm) = 7.56 (s, 1H), 7.48 (s, 1H), 7.45 (d, *J* = 3.2 Hz, 1H), 7.42 – 7.37 (m, 2H), 7.31 – 7.26 (m, 5H), 6.93 – 6.89 (m, 1H), 6.87 (dd, *J* = 8.7, 3.3 Hz, 2H), 6.84 – 6.80 (m, 2H), 6.60 – 6.53 (m, 1H), 6.50 (s, 5H), 6.49 (s, 4H), 5.17 – 5.02 (m, 3H), 5.02 – 4.95 (m, 1H), 4.87 – 4.78 (m, 2H), 4.69 (d, *J* = 14.7 Hz, 1H), 4.47 (dq, *J* = 10.8, 7.1 Hz, 1H), 4.35 (dq, *J* = 10.9, 7.1 Hz, 1H), 3.87 (dqd, *J* = 10.8, 7.1, 2.0 Hz, 2H), 3.80 (s, 3H), 3.77 – 3.69 (m, 8H), 3.38 (dq, *J* = 10.9, 7.1 Hz, 1H), 3.17 (dq, *J* = 10.9, 7.1 Hz, 1H), 1.40 (t, *J* = 7.1 Hz, 2H), 1.04 (t, *J* = 7.1 Hz, 3H), 0.96 (t, *J* = 7.1 Hz, 2H), 0.78 (t, *J* = 7.2 Hz, 3H).
^{13}C{^{1}H}-NMR (101 MHz, $CDCl_3$): δ (ppm) = 169.5, 169.1, 168.1, 168.0, 162.1, 161.7, 161.4, 161.2, 159.4, 159.3, 159.1, 137.8, 137.6, 137.5, 137.4, 130.6, 129.2, 129.1, 129.1, 128.9, 128.7, 128.6, 118.2, 118.0, 114.2, 114.0, 114.0, 113.9, 94.5, 93.9, 93.1, 93.0, 63.6, 62.6, 62.2, 62.2, 55.4, 55.3, 55.2, 49.6, 49.6, 49.4, 49.1, 14.6, 14.1, 13.9.

IR (Diamond-ATR, neat) ν_{max} (cm^{-1}) = 1593, 1532, 1511, 1443, 1384, 1353, 1327, 1228, 1176, 1088, 1028, 798, 775.

HRMS (APCI) *m/z*: $[M + H]^+$ calc. for $(C_{33}H_{36}ClN_4O_8Zr)^+$: 741.1263, found: 741.1297.

m.p.: 173 – 175 °C (PhMe/hexanes).

Anal. Calcd for $C_{33}H_{35}ClN_4O_8Zr \cdot 0.2\ C_7H_8$: C, 54.31; H, 4.85; N, 7.36. Found: C, 54.30; H, 4.86; N, 7.36.

(μ-O)-Di-[cyclopentadienyl-bis((4-(ethoxycarbonyl)-1-phenyl-1*H*-pyrazol-5-yl)oxy)zirconium] (38)

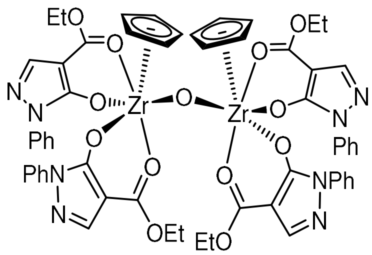

Cp_2ZrCl_2 (148 mg, 0.506 mmol, 1.00 equiv) was added with stirring to pyrazolone **6** (235 mg, 1.01 mmol, 2.00 equiv) and pyridine (80.0 mg, 0.08 mL, 1.01 mmol, 2.00 equiv) in THF (10 mL) at 65 °C. The mixture was heated at reflux for 16 h, cooled to room temperature, diluted with CH_2Cl_2 (10 mL) and a mixture of Et_2O and hexanes (1 : 1; 30 mL) was added. The precipitated pyridine hydrochloride was removed by filtration and the filtrate was evaporated. Recrystallization of the residue from PhMe and hexanes gave Zr-pyrazolone complex **38** (201 mg, 0.160 mmol, 32%) as a yellow-white solid.

^{1}H-NMR (400 MHz, $CDCl_3$): δ (ppm) = 7.96 – 7.86 (m, 5H), 7.80 (s, 1H), 7.78 (s, 1H), 7.71 (s, 1H), 7.65 (s, 1H), 7.57 (s, 2H), 7.57 – 7.52 (m, 1H), 7.48 (t, *J* = 7.8 Hz, 2H), 7.37 (q, *J* = 7.5 Hz, 2H), 7.28 (dd, *J* = 8.8, 6.7 Hz, 3H), 7.18 (t, *J* = 7.5 Hz, 1H), 6.60 (d, *J* = 1.8 Hz, 8H), 4.51 (ddq, *J* = 57.2, 10.8, 7.1 Hz, 1H), 4.22 (dq, *J* = 10.9, 7.2 Hz, 1H), 4.13 (dq, *J* = 10.8, 7.2 Hz, 1H), 3.95 (dq, *J* = 10.6, 7.1 Hz, 2H), 3.78 (dq, *J* = 10.6, 7.1 Hz, 1H), 1.49 (t, *J* = 7.1 Hz, 2H), 1.26 (t, *J* = 7.2 Hz, 3H), 1.20 (t, *J* = 7.1 Hz, 2H), 1.11 (t, *J* = 7.0 Hz, 3H).

^{13}C{^{1}H}-NMR (101 MHz, $CDCl_3$): δ (ppm) = 169.7, 169.4, 168.6, 168.3, 162.2, 162.0, 161.5, 161.1, 138.9, 138.8, 138.7, 138.6, 138.4, 138.3, 138.2, 129.2, 129.2, 129.1, 129.0, 128.7, 126.8, 126.6, 126.4, 126.2, 121.8, 121.4, 120.9, 120.5, 120.2, 118.3, 118.1, 95.5, 95.1, 94.3, 93.9, 63.9, 63.1, 62.9, 62.6, 14.7, 14.2, 14.2.

IR (Diamond-ATR, neat) ν_{max} (cm^{-1}) = 1651, 1634, 1612, 1593, 1586, 1541, 1530, 1500, 1489, 1467, 1431, 1387, 1357, 1321, 1254, 1117, 1084, 1055, 1009, 954, 775, 738, 690.

m.p.: >190 °C (decomp.).

Bis(ethyl 5-hydroxy-1-(4-methoxybenzyl)-1*H*-pyrazole-4-carboxylate)tetrakis(acetato)dirhodium (39)

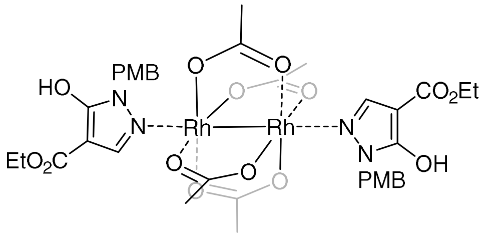

$[Rh_2(OAc)_4]$ (92.0 mg, 0.208 mmol, 1.00 equiv) was added with stirring to pyrazolone **8** (230 mg, 0.833 mmol, 4.00 equiv) in MeCN (4.0 mL) and the mixture was heated at reflux at 85 °C for 18 h, cooled to room temperature and evaporated to dryness. The residue was redissolved in CH_2Cl_2 (5.0 mL) and the complex was precipitated with the addition of hexanes (15 mL). The mixture was cooled in an ice-bath, the precipitate was collected by filtration and dried *in vacuo*. Rh-pyrazolone complex **39** (202 mg, 0.203 mmol, 98%) was obtained as a purple-red solid, essentially pure by its ^{1}H NMR spectrum. Crystals suitable for X-ray crystallography were obtained from the CH_2Cl_2/hexanes filtrate.

^{1}H-NMR (400 MHz, CD_2Cl_2): δ (ppm) = 9.34 (s, 2H), 8.12 (s, 2H), 7.45 (d, *J* = 8.4 Hz, 4H), 6.89 – 6.79 (m, 4H), 5.61 (s, 4H), 4.38 (q, *J* = 7.1 Hz, 4H), 3.76 (s, 6H), 1.84 (s, 11H), 1.38 (t, *J* = 7.1 Hz, 6H).

^{13}C{^{1}H}-NMR (101 MHz, CD_2Cl_2): δ (ppm) = 191.6, 159.7, 157.5, 139.9, 130.3, 128.7, 114.0, 95.8, 61.2, 55.6, 50.5, 23.8, 14.6.

IR (Diamond-ATR, neat) ν_{max} (cm^{-1}) = 1700, 1627, 1611, 1591, 1512, 1436, 1247, 1176, 1031, 775, 695.

HRMS (ES-ToF) *m/z*: $[M + H]^+$ calc. for $(C_{36}H_{45}N_4O_{16}Rh_2)^+$: 995.0935, found: 995.0932.

m.p.: 195 – 199 °C (EtOH) (decomp.).

Anal. Calcd for $C_{36}H_{44}N_4O_{16}Rh_2$: C, 43.48; H, 4.46; N, 5.63. Found: C, 43.37; H, 4.42; N, 5.57.

Bis(ethyl 5-hydroxy-1-phenyl-1*H*-pyrazole-4-carboxylate)tetrakis(acetato)dirhodium (40)

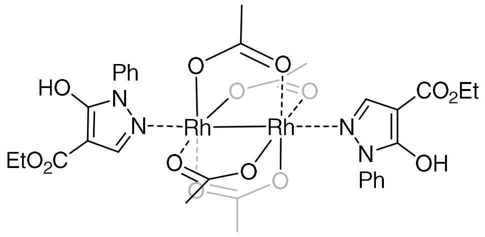

$[Rh_2(OAc)_4]$ (81.0 mg, 0.183 mmol, 1.00 equiv) was added with stirring to pyrazolone **6** (170 mg, 0.732 mmol, 4.00 equiv) in MeCN (4.0 mL) and the mixture was heated at reflux to 85 °C for 19 h, cooled to room temperature and evaporated to dryness. The residue was redissolved in CH_2Cl_2 (5.0 mL) and the complex was precipitated with the addition of hexanes (15 mL). The mixture was cooled in an ice-bath, the precipitate was collected by filtration and dried *in vacuo*. Rh-pyrazolone complex **39** (155 mg, 0.171 mmol, 93%) was obtained as a purple-red solid, essentially pure by its ^{1}H NMR spectrum. Crystals suitable for X-ray crystallography were obtained from a CH_2Cl_2/hexanes solution.

^{1}H-NMR (400 MHz, CD_2Cl_2): δ (ppm) = 8.08 (s, 2H), 7.80 (dd, *J* = 7.6, 1.7 Hz, 4H), 7.47 – 7.40 (m, 4H), 7.39 – 7.33 (m, 2H), 4.41 (q, *J* = 7.1 Hz, 4H), 1.72 (s, 12H), 1.40 (t, *J* = 7.1 Hz, 6H).

^{13}C{^{1}H}-NMR (101 MHz, CD_2Cl_2): δ (ppm) = 191.1, 166.5, 157.5, 148.0, 140.3, 136.8, 129.2, 129.0, 128.4, 125.7, 124.7, 119.5, 95.7, 61.4, 23.6, 14.6.

IR (Diamond-ATR, neat) ν_{max} (cm^{-1}) = 3297, 1670, 1589, 1569, 1457, 1420, 1410, 1321, 1174, 1127, 965, 777, 751, 691.

m.p.: 210 – 212 °C (EtOH) (decomp.).

Anal. Calcd for $C_{32}H_{36}N_4O_{14}Rh_2 \cdot 0.5\ CH_2Cl_2 \cdot 0.5\ C_6H_{14}$: C, 42.98; H, 4.47; N, 5.65. Found: C, 43.37; H, 4.41; N, 5.57.

Bis(ethanol) bis((4-(ethoxycarbonyl)-1-(4-methoxybenzyl)-1*H*-pyrazol-5-yl)oxy)manganese (41)

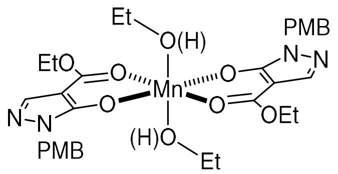

$Mn(OAc)_2$ (58.0 mg, 0.335 mmol, 1.00 equiv) was added with stirring to pyrazolone **8** (185 mg, 0.670 mmol, 2.00 equiv) in EtOH (6.5 mL) at 79 °C. The mixture was heated at reflux for 4 h, cooled to

room temperature, Et_2O was added (15 mL), and the precipitate was collected by filtration and dried *in vacuo*. Mn-pyrazolone complex **41** (157 mg, 0.225 mmol, 67%) was obtained as a pale-pink solid.

IR (Diamond-ATR, neat) ν_{max} (cm^{-1}) = 1638, 1560, 1523, 1511, 1433, 1417, 1331, 1239, 1219, 1186, 1172, 1111, 1030, 10001, 800, 779.

HRMS (ESI) *m*/*z*: [M – 2 EtOH]$^+$ calc. for $(C_{28}H_{31}MnN_4O_8)^+$: 606.1517, found: 606.1515.

m.p.: >250 °C (EtOH) (decomp.).

Bis(ethanol) bis((4-(ethoxycarbonyl)-1-phenyl-1*H*-pyrazol-5-yl)oxy)manganese (42)

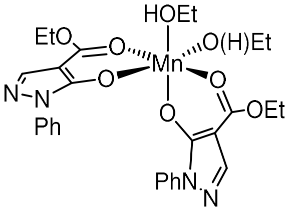

$Mn(OAc)_2$ (67.0 mg, 0.388 mmol, 1.00 equiv) was added with stirring to pyrazolone **6** (180 mg, 0.775 mmol, 2.00 equiv) in EtOH (6.5 mL) at 79 °C. The mixture was heated at reflux for 4 h, cooled to room temperature, Et_2O was added (15 mL), and the precipitate was collected by filtration and dried *in vacuo*. Mn-pyrazolone complex **42** (140 mg, 0.230 mmol, 59%) was obtained as a white solid. Crystals suitable for X-ray crystallography were obtained by slow evaporation from an EtOH solution of complex **42**.

IR (Diamond-ATR, neat) ν_{max} (cm^{-1}) = 1626, 1597, 1559, 1525, 1498, 1457, 1428, 1381, 1374, 1324, 1312, 1247, 1232, 1120, 1083, 952, 852, 780, 751, 687.

m.p.: >230 °C (EtOH), (decomp.).

Anal. Calcd for $C_{24}H_{22}MnN_4O_6$: C, 55.71; H, 4.29; N, 10.83. Found: C, 55.38; H, 4.29; N, 10.88.

Bis(ethanol) bis((4-benzoyl-1-(4-methoxybenzyl)-1*H*-pyrazol-5-yl)oxy)manganese (43)

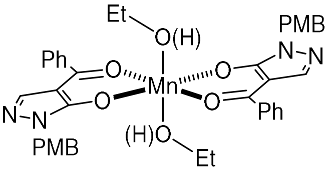

$Mn(OAc)_2$ (49.0 mg, 0.281 mmol, 1.00 equiv) was added with stirring to pyrazolone **10** (173 mg, 0.561 mmol, 2.00 equiv) in EtOH (6.5 mL) at 79 °C. The mixture was heated at reflux for 4 h, cooled to room temperature, Et_2O was added (15 mL), and the precipitate was collected by filtration and dried *in vacuo*. Mn-pyrazolone complex **43** (141 mg, 0.185 mmol, 65%) was obtained as a white solid.

IR (Diamond-ATR, neat) ν_{max} (cm^{-1}) = 1601, 1577, 1521, 1508, 1497, 1486, 1438, 1415, 1375, 1240, 1172, 1027, 898, 800, 758, 710, 699.

m.p.: >288 °C (EtOH) (decomp.).

Bis(ethanol) bis((4-(ethoxycarbonyl)-1*H*-pyrazol-5-yl)oxy)manganese (44)

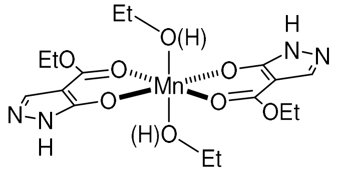

$Mn(OAc)_2$ (64.0 mg, 0.368 mmol, 1.00 equiv) was added with stirring to pyrazolone **5** (115 mg, 0.737 mmol, 2.00 equiv) in EtOH (6.5 mL) at 79 °C. The mixture was heated at reflux for 18 h, cooled to room temperature, Et_2O was added (15 mL), and the precipitate was collected by filtration and dried *in vacuo*. Mn-pyrazolone complex **44** (99.0 mg, 0.216 mmol, 59%) was obtained as a pale-pink solid.

IR (Diamond-ATR, neat) ν_{max} (cm^{-1}) = 3336, 1663, 1638, 1545, 1525, 1435, 1319, 1269, 1172, 1118, 1061, 928, 787, 753.

m.p.: >230 °C (EtOH) (decomp.).

Tris((4-(ethoxycarbonyl)-1-(4-methoxybenzyl)-1*H*-pyrazol-5-yl)oxy)iron (45)

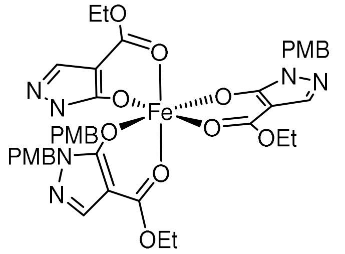

$FeCl_3$•$6H_2O$ (83.0 mg, 0.309 mmol, 1.00 equiv) was added with stirring to pyrazolone **8** (256 mg, 0.927 mmol, 3.00 equiv) and NaOAc (76.0 mg, 0.927 mmol, 3.00 equiv) in EtOH (6.5 mL) at 79 °C. The mixture was heated at reflux for 19 h, after which H_2O was added (20 mL), the mixture was cooled to room temperature, and the precipitate was collected by filtration, washed with H_2O (3 x 10 mL) and dried *in vacuo*. Fe-pyrazolone complex **45** (225 mg, 0.255 mmol, 83%) was obtained as a purple-black solid.

IR (Diamond-ATR, neat) ν_{max} (cm^{-1}) = 1586, 1525, 1509, 1465, 1437, 1382, 1351, 1319, 1243, 1221, 1174, 1088, 1025, 800, 780.

m.p.: 166 – 168 °C (EtOH).

Anal. Calcd for $C_{42}H_{45}N_6O_{12}Fe \cdot H_2O \cdot$ EtOH: C, C, 55.88; H, 5.65; N, 8.89. Found: C, 55.63; H, 5.14; N, 8.94.

Tris((4-(ethoxycarbonyl)-1-phenyl-1*H*-pyrazol-5-yl)oxy)iron (46)

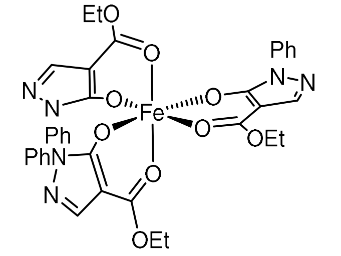

$FeCl_3$•$6H_2O$ (79.0 mg, 0.293 mmol, 1.00 equiv) was added with stirring to pyrazolone **6** (204 mg, 0.878 mmol, 3.00 equiv) and NaOAc (72.0 mg, 0.878 mmol, 3.00 equiv) in EtOH (6.0 mL) at 79 °C. The mixture was heated at reflux for 17 h, after which H_2O was added (20 mL), the mixture was cooled to room temperature, and the precipitate was collected by filtration, washed with H_2O (3 x 10 mL) and dried *in vacuo*. Fe-pyrazolone complex **46** (182 mg, 0.243 mmol, 83%) was obtained as a purple-red

solid. Crystals suitable for X-ray crystallography were obtained by slow evaporation from an EtOH solution of complex **46**.

IR (Diamond-ATR, neat) ν_{max} (cm^{-1}) = 1606, 1593, 1580, 1527, 1498, 1471, 1457, 1429, 1387, 1357, 1323, 1256, 1116, 1083, 1002, 951, 779, 753, 687, 632.

m.p.: 158 – 160 °C (EtOH).

Anal. Calcd for $C_{36}H_{33}FeN_6O_9$: C, 57.69; H, 4.44; N, 11.21. Found: C, 57.74; H, 4.45; N, 10.44.

Tris((4-benzoyl-1-(4-methoxybenzyl)-1*H*-pyrazol-5-yl)oxy)iron (47)

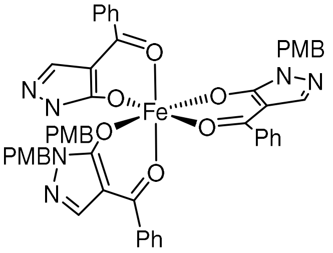

$FeCl_3$•$6H_2O$ (57.0 mg, 0.211 mmol, 1.00 equiv) was added with stirring to pyrazolone **10** (195 mg, 0.632 mmol, 3.00 equiv) and NaOAc (52.0 mg, 0.632 mmol, 3.00 equiv) in EtOH (5.0 mL) at 79 °C. The mixture was heated at reflux for 18 h, after which H_2O was added (20 mL), the mixture was cooled to room temperature, and the precipitate was collected by filtration, washed with H_2O (3 x 10 mL) and dried *in vacuo*. Fe-pyrazolone complex **47** (93 mg, 0.095 mmol, 45%) was obtained as a purple-red solid.

IR (Diamond-ATR, neat) ν_{max} (cm^{-1}) = 1608, 1588, 1566, 1511, 1473, 1445, 1391, 1312, 1303, 1243, 1176, 1025, 902, 807, 797, 751, 706.

m.p.: 163 – 165 °C (EtOH).

Tris((4-(ethoxycarbonyl)-1*H*-pyrazol-5-yl)oxy)iron (48)

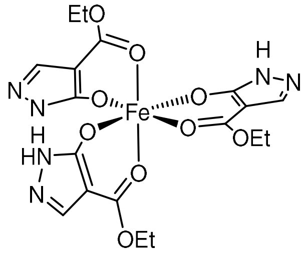

$FeCl_3$•$6H_2O$ (58.0 mg, 0.213 mmol, 1.00 equiv) was added with stirring to pyrazolone **5** (100 mg, 0.640 mmol, 3.00 equiv) and NaOAc (53.0 mg, 0.640 mmol, 3.00 equiv) in EtOH (5.0 mL) at 79 °C. The mixture was heated at reflux for 18 h, after which H_2O was added (20 mL), the mixture was cooled to room temperature, and the precipitate was collected by filtration, washed with H_2O (3 x 10 mL) and dried *in vacuo*. Fe-pyrazolone complex **48** (110 mg, 0.211 mmol, 98%) was obtained as a purple-black solid.

IR (Diamond-ATR, neat) ν_{max} (cm^{-1}) = 3310, 1689, 1616, 1580, 1558, 1517, 1445, 1269, 1108, 1077, 944, 783, 775.

m.p.: >250 °C (EtOH) (decomp.).

((4-(Ethoxycarbonyl)-1-(4-methoxybenzyl)-1*H*-pyrazol-5-yl)oxy)zinc polymer (49)

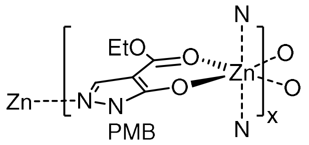

$Zn(OAc)_2$•(75.0 mg, 0.344 mmol, 1.00 equiv) was added with stirring to pyrazolone **8** (190 mg, 0.688 mmol, 2.00 equiv) in (10 mL) at 79 °C. After 14 h at reflux, the mixture was cooled to room temperature and placed in an ice-bath. The resultant precipitate was collected by filtration, washed with Et_2O (5.0 mL) and dried *in vacuo*. Zn-pyrazolone complex **49** (191 mg, 0.310 mmol, 90%) was obtained as a white solid, essentially pure by its ^{1}H NMR spectrum. Crystals suitable for X-ray crystallography were obtained by slow evaporation from an EtOH solution of complex **49**.

^{1}H-NMR (400 MHz, $OS(CD_3)_2$): δ (ppm) = 7.33 (s, 1H), 7.10 (d, *J* = 8.1 Hz, 2H), 6.76 (d, *J* = 8.1 Hz, 2H), 4.84 (s, 2H), 4.02 (q, *J* = 7.1 Hz, 2H), 3.68 (s, 3H), 1.11 (t, *J* = 7.1 Hz, 3H).

^{13}C{^{1}H}-NMR (101 MHz, $OS(CD_3)_2$): δ (ppm) = 167.5, 164.6, 158.2, 137.2, 130.8, 128.6, 113.5, 90.5, 59.0, 55.0, 46.9, 14.4.

IR (Diamond-ATR, neat) v_{max} (cm^{-1}) = 1640, 1561, 1526, 1511, 1435, 1359, 1332, 1284, 1238, 1219, 1187, 1172, 1111, 1029, 1003, 869, 801, 779.

HRMS (ES-ToF) *m/z*: $[M + H]^+$ calc. for $(C_{28}H_{31}N_4O_8Zn)^+$: 615.1428, found: 615.1422.

m.p.: 226 – 228 °C (EtOH).

Anal. Calcd for $C_{28}H_{30}ZnN_4O_8$: C, 54.60; H, 4.91; N, 9.10. Found: C, 54.69; H, 4.91; N, 8.85.

Bis(ethanol) bis((4-(ethoxycarbonyl)-1-phenyl-1*H*-pyrazol-5-yl)oxy)zinc (50)

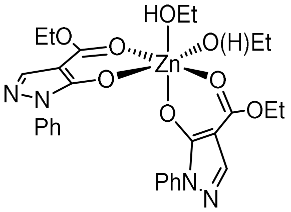

$Zn(OAc)_2$•$2H_2O$ (101 mg, 0.461 mmol, 1.00 equiv) was added with stirring to pyrazolone **6** (214 mg, 0.921 mmol, 2.00 equiv) in EtOH (10 mL). at 79 °C After 13 h at reflux, the mixture was cooled to room temperature and placed in an ice-bath. The resultant precipitate was collected by filtration, washed with Et_2O (5.0 mL) and dried *in vacuo*. Zn-pyrazolone complex **50** (233 mg, 0.376 mmol, 82%) was obtained as a white solid, essentially pure by its ^{1}H NMR spectrum. Crystals suitable for X-ray crystallography were obtained by slow evaporation from an EtOH solution of complex **50**.

^{1}H-NMR (400 MHz, $CDCl_3$): δ (ppm) = 7.69 (d, *J* = 7.9 Hz, 2H), 7.61 (s, 1H), 7.17 (t, *J* = 7.7 Hz, 2H), 7.06 (t, *J* = 7.4 Hz, 1H), 4.11 (q, *J* = 7.1 Hz, 2H), 3.77 – 3.66 (m, 2H), 1.27 (dd, *J* = 16.8, 4.9 Hz, 3H), 1.23 (d, *J* = 4.9 Hz, 3H).

^{13}C{^{1}H}-NMR (101 MHz, $CDCl_3$): δ (ppm) = 168.8, 164.5, 140.8, 138.0, 128.6, 126.1, 122.1, 93.1, 61.0, 58.6, 18.6, 14.5.

IR (Diamond-ATR, neat) v_{max} (cm^{-1}) = 1673, 1636, 1598, 1550, 1526, 1499, 1457, 1417, 1359, 1329, 1226, 1081, 1051, 947, 780, 753.

HRMS (ES-ToF) *m/z*: $[M – 2\ EtOH + H]^+$ calc. for $(C_{24}H_{23}N_4O_6Zn)^+$: 527.0904, found: 527.0884.

m.p.: 150 – 154 °C (EtOH), >235 °C (decomp.).

Anal. Calcd for $C_{28}H_{34}N_4O_8Zn$: C, 54.24; H, 5.53; N, 9.04. Found: C, 54.11; H, 4.96; N, 9.06.

((4-Benzoyl-1-(4-methoxybenzyl)-1*H*-pyrazol-5-yl)oxy)zinc polymer (51)

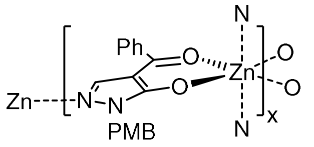

$Zn(OAc)_2•2H_2O$ (49.0 mg, 0.224 mmol, 1.00 equiv) was added with stirring to pyrazolone **10** (138 mg, 0.448 mmol, 2.00 equiv) in EtOH (4.0 mL) at 79 °C. After heating at reflux for 2 h, the mixture was cooled to room temperature, and the resultant precipitate was collected by filtration and dried *in vacuo*. Zn-pyrazolone complex **51** (129 mg, 0.190 mmol, 85%) was obtained as a white solid, essentially pure by its ^{1}H NMR spectrum.

^{1}H-NMR (400 MHz, $OS(CD_3)_2$): δ (ppm) = 7.59 (d, *J* = 7.1 Hz, 2H), 7.51 (d, *J* = 6.9 Hz, 0H), 7.48 – 7.40 (m, 3H), 7.15 (d, *J* = 8.1 Hz, 2H), 6.70 (s, 2H), 4.87 (s, 2H), 3.65 (s, 3H).

$^{13}C\{^1H\}$-NMR (101 MHz, $OS(CD_3)_2$): δ (ppm) = 187.0, 165.4, 158.2, 139.9, 139.5, 130.9, 130.3, 128.8, 128.4, 128.0, 113.5, 102.4, 55.0, 46.7.

IR (Diamond-ATR, neat) ν_{max} (cm^{-1}) = 1600, 1577, 1506, 1487, 1439, 1414, 1378, 1320, 1239, 1172, 1025, 900, 801, 792, 756, 698.

HRMS (ES-ToF) *m/z*: $[M + H]^+$ calc. for $(C_{36}H_{31}N_4O_6Zn)^+$: 679.1530, found: 679.1788.

m.p.: 222 – 224 °C (EtOH).

Anal. Calcd for $C_{36}H_{30}ZnN_4O_6 \cdot 0.5\ H_2O$: C, 62.75; H, 4.53; N, 8.13. Found: C, 62.76; H, 4.16; N, 7.86.

Bis(ethanol) bis((4-(ethoxycarbonyl)-1*H*-pyrazol-5-yl)oxy)zinc (52)

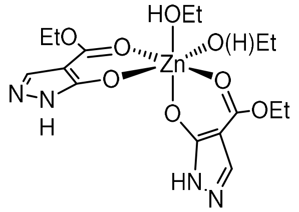

$Zn(OAc)_2•2H_2O$ (76.0 mg, 0.346 mmol, 1.00 equiv) was added with stirring to pyrazolone **5** (108 mg, 0.692 mmol, 2.00 equiv) in EtOH (4.0 mL) at 79 °C. After 48 h at reflux, heating was stopped and Et_2O (10 mL) was slowly added to the warm solution. On cooling to room temperature, the resultant precipitate was collected by filtration and dried *in vacuo*. Zn-pyrazolone complex **52** (114 mg, 0.244 mmol, 70%) was obtained as a white solid, essentially pure by its ^{1}H NMR spectrum.

^{1}H-NMR (400 MHz, $OS(CD_3)_2$): δ (ppm) = 11.37 (s, 1H), 7.37 (s, 1H), 4.16 – 4.10 (m, 3H), 1.21 (d, *J* = 8.5 Hz, 4H).

$^{13}C\{^1H\}$-NMR (126 MHz, $OS(CD_3)_2$): δ (ppm) = 172.5, 167.6, 138.5, 89.3, 59.2, 14.5.

IR (Diamond-ATR, neat) ν_{max} (cm^{-1}) = 3322, 1666, 1635, 1527, 1437, 1318, 1275, 1172, 1120, 1064, 1009, 932, 858, 787, 751, 708.

m.p.: >260 °C (EtOH) (decomp.).

Anal. Calcd for $C_6H_7N_2O_3Zn$: C, 32.68; H, 3.20; N, 12.70. Found: C, 35.72; H, 3.72; N, 12.71.

Bis(ethanol) bis((4-(ethoxycarbonyl)-1-(4-methoxybenzyl)-1*H*-pyrazol-5-yl)oxy)copper (53)

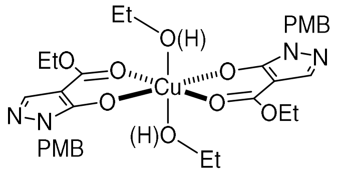

$Cu(OAc)_2$•H_2O (68.0 mg, 0.340 mmol, 1.00 equiv) was added with stirring to pyrazolone **8** (188 mg, 0.681 mmol, 2.00 equiv) in EtOH (3.5 mL) at 79 °C. After 3.5 h at reflux, the mixture was cooled to room temperature, and the resultant precipitate was collected by filtration and dried *in vacuo*. Cu-pyrazolone complex **53** (103 mg, 0.146 mmol, 43%) was obtained as a green-brown solid. Crystals suitable for X-ray crystallography were obtained by slow evaporation from an ethanolic pyridine solution of complex **53**.

IR (Diamond-ATR, neat) ν_{max} (cm^{-1}) = 1662, 1560, 1550, 1525, 1511, 1465, 1437, 1414, 1329, 1236, 1217, 1183, 1173, 1115, 1040, 1010, 794, 778.

HRMS (ES-ToF) *m/z*: $[M – Solvent + H]^+$ calc. for $(C_{28}H_{31}CuN_4O_8)^+$: 614.1432, found: 614.1454.

m.p.: 211 – 213 °C (EtOH).

Bis((4-(ethoxycarbonyl)-1-phenyl-1*H*-pyrazol-5-yl)oxy)copper polymer (54)

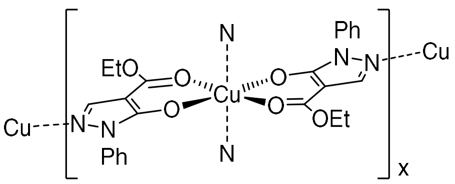

$Cu(OAc)_2$•H_2O (61.0 mg, 0.308 mmol, 1.00 equiv) was added with stirring to pyrazolone **6** (143 mg, 0.616 mmol, 2.00 equiv) in EtOH (3.5 mL) at 79 °C. After 2 h at reflux, the mixture was cooled to room temperature, and the resultant precipitate was collected by filtration and dried *in vacuo*. Cu-pyrazolone complex **54** (141 mg, 0.268 mmol, 88%) was obtained as a green-brown solid. Crystals suitable for X-ray crystallography were obtained by slow evaporation from an EtOH solution of complex **54**.

IR (Diamond-ATR, neat) ν_{max} (cm^{-1}) = 1615, 1593, 1382, 1554, 1498, 1457, 1438, 1372, 1338, 1262, 1126, 1086, 1014, 950, 905, 775, 747, 686.

HRMS (ES-ToF) *m/z*: $[M – Solvent + H]^+$ calc. for $(C_{24}H_{23}CuN_4O_6)^+$: 526.0908, found: 526.0907.

m.p.: 239 – 241 °C (EtOH).

Anal. Calcd for $C_{24}H_{22}CuN_4O_6$: C, 54.80; H, 4.22; N, 10.65. Found: C, 54.37; H, 4.17; N, 10.58.

Bis(ethanol) bis((4-benzoyl-1-(4-methoxybenzyl)-1*H*-pyrazol-5-yl)oxy)copper (55)

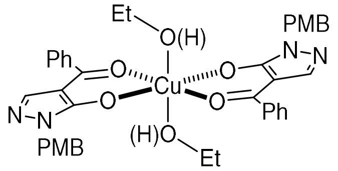

$Cu(OAc)_2$•H_2O (43.0mg, 0.216 mmol, 1.00 equiv) was added with stirring to pyrazolone **10** (133 mg, 0.431 mmol, 2.00 equiv) in EtOH (3.5 mL) at 79 °C. After 2 h at reflux, the mixture was cooled to room

temperature, and the resultant precipitate was collected by filtration and dried *in vacuo*. Cu-pyrazolone complex **55** (124 mg, 0.161 mmol, 75%) was obtained as a brown solid.

IR (Diamond-ATR, neat) ν_{max} (cm^{-1}) = 1594, 1573, 1530, 1511, 1503, 1487, 1438, 1392, 1321, 1286, 1240, 1172, 1026, 902, 804, 796, 754, 699.

HRMS (ES-ToF) *m/z*: [M – Solvent + H]$^+$ calc. for $(C_{36}H_{31}CuN_4O_6)^+$: 678.1534, found: 678.1542.

m.p.: 211 – 214 °C (EtOH).

Anal. Calcd for $C_{36}H_{30}CuN_4O_6$: C, 63.76; H, 4.46; N, 8.26. Found: C, 63.56; H, 4.46; N, 8.18.

Bis(ethanol) bis((4-(ethoxycarbonyl)-1*H*-pyrazol-5-yl)oxy)copper (56)

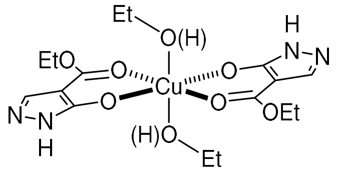

$Cu(OAc)_2•H_2O$ (85.0 mg, 0.426 mmol, 1.00 equiv) was added with stirring to pyrazolone **5** (133 mg, 0.852 mmol, 2.00 equiv) in EtOH (3.5 mL) at 79 °C. After 18 h at reflux, the mixture was cooled to room temperature, and the resultant precipitate was collected by filtration and dried *in vacuo*. Cu-pyrazolone complex **56** (151 mg, 0.324 mmol, 76%) was obtained as a dark green solid. Crystals suitable for X-ray crystallography were obtained by slow evaporation from an ethanolic pyridine solution of complex **56**.

IR (Diamond-ATR, neat) ν_{max} (cm^{-1}) = 3287, 1676, 1636, 1546, 1523, 1438, 1317, 1171, 1105, 1066, 938, 784, 763, 747.

HRMS (ES-ToF) *m/z*: [M – Solvent + H]$^+$ calc. for $(C_{12}H_{15}CuN_4O_6)^+$: 374.0282, found: 374.0051.

m.p.: 235 – 238 °C (EtOH).

Bis(ethanol) bis((4-(ethoxycarbonyl)-1-(4-methoxybenzyl)-1*H*-pyrazol-5-yl)oxy)nickel (57)

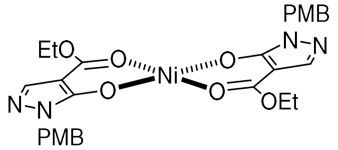

$NiCl_2•6H_2O$ (66.0 mg, 0.264 mmol, 1.00 equiv) was added with stirring to pyrazolone **5** (146 mg, 0.528 mmol, 2.00 equiv) in EtOH (3.5 mL) at 79 °C. After 24 h at reflux, the mixture was cooled to room temperature, and the resultant precipitate was collected by filtration and dried *in vacuo*. Ni-pyrazolone complex **57** (137 mg, 0.195 mmol, 74%) was obtained as a green-turquoise solid. Crystals suitable for X-ray crystallography were obtained by slow evaporation from an ethanolic pyridine solution of complex **57**.

IR (Diamond-ATR, neat) ν_{max} (cm^{-1}) = 1636, 1566, 1528, 1511, 1444, 1336, 1221, 1191, 1031, 803, 778.

m.p.: >300 °C (EtOH) (decomp.).

Anal. Calcd for $C_{28}H_{30}NiN_4O_8$: C, 55.20; H, 4.96; N, 9.20. Found: C, 55.12; H, 4.78; N, 8.71.

Bis(ethanol) bis((4-(ethoxycarbonyl)-1-phenyl-1*H*-pyrazol-5-yl)oxy)nickel (58)

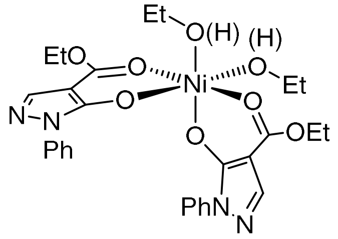

$NiCl_2$•$6H_2O$ (69.0 mg, 0.276 mmol, 1.00 equiv) was added with stirring to pyrazolone **6** (128 mg, 0.551 mmol, 2.00 equiv) in EtOH (3.5 mL). After 24 h at reflux, the mixture was cooled to room temperature, and the resultant precipitate was collected by filtration and dried *in vacuo*. Ni-pyrazolone complex **58** (102 mg, 0.166 mmol, 60%) was obtained as a blue-turquoise solid. Crystals suitable for X-ray crystallography were obtained by slow evaporation from an ethanolic pyridine solution of complex **58**.

IR (Diamond-ATR, neat) ν_{max} (cm^{-1}) = 1642, 1597, 1588, 1570, 1528, 1498, 1457, 1431, 1380, 1358, 1339, 1327, 1237, 1124, 1084, 1060, 953, 896, 782, 755.

m.p.: >300 °C (EtOH) (decomp.).

Anal. Calcd for $C_{24}H_{22}N_4O_6Ni$: C, 55.31; H, 4.26; N, 10.75. Found: C, 55.06; H, 4.25; N, 10.50.

Bis(ethanol) bis((4-benzoyl-1-(4-methoxybenzyl)-1*H*-pyrazol-5-yl)oxy)nickel (59)

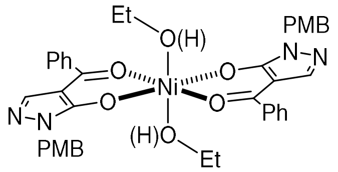

$NiCl_2$•$6H_2O$ (58.0 mg, 0.234 mmol, 1.00 equiv) was added with stirring to pyrazolone **10** (144 mg, 0.467 mmol, 2.00 equiv) in EtOH (3.5 mL) at 79 °C. After 24 h at reflux, the mixture was cooled to room temperature, and the resultant precipitate was collected by filtration and dried *in vacuo*. Ni-pyrazolone complex **59** (107 mg, 0.140 mmol, 60%) was obtained as a green-turquoise solid.

IR (Diamond-ATR, neat) ν_{max} (cm^{-1}) = 1603, 1577, 1508, 1485, 1437, 1321, 1240, 1180, 1058, 804, 794, 755, 697.

m.p.: 294 – 296 °C (EtOH).

Anal. Calcd for $C_{36}H_{30}N_4O_6Ni \cdot H_2O$: C, 62.54; H, 4.67; N, 8.10. Found: C, 62.82; H, 4.50; N, 7.98.

Bis(ethanol) bis((4-(ethoxycarbonyl)-1*H*-pyrazol-5-yl)oxy)nickel (60)

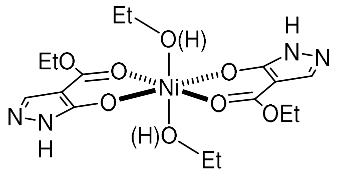

$NiCl_2$•$6H_2O$ (96.0 mg, 0.384 mmol, 1.00 equiv) was added with stirring to pyrazolone **5** (120 mg, 0.769 mmol, 2.00 equiv) in EtOH (3.5 mL) at 79 °C. After 24 h at reflux, the mixture was cooled to room temperature, and the resultant precipitate was collected by filtration and dried *in vacuo*. Ni-pyrazolone complex **60** (110 mg, 0.239 mmol, 62%) was obtained as a green solid.

IR (Diamond-ATR, neat) ν_{max} (cm^{-1}) = 3325, 1636, 1560, 1548, 1526, 1470, 1443, 1323, 1278, 1172, 1126, 1067, 933, 785.

m.p.: 255 – 257 °C (EtOH).

Sodium(I) tetrakis((4-(ethoxycarbonyl)-1-(4-methoxybenzyl)-1H-pyrazol-5-yl)oxy)lanthanum (61)

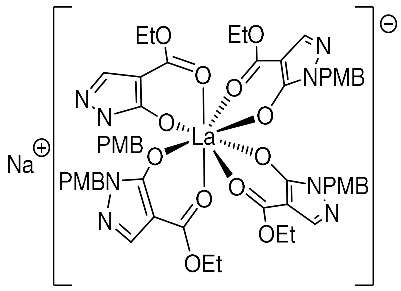

$La(OTf)_3$ (156 mg, 0.267 mmol, 1.00 equiv) was added with stirring to pyrazolone **8** (221 mg, 0.800 mmol, 3.00 equiv) in EtOH (4.0 mL) at 79 °C. Aqueous NaOH (2 M; 0.40 mL, 0.800 mmol, 3.00 equiv) was added with stirring and the mixture was heated at reflux for 18 h, after which the mixture was co-evaporated to dryness with Et_2O (4 x 15 mL), and the residue was suspended in Et_2O, collected by filtration and dried *in vacuo*. La-pyrazolone complex **61** (326 mg, 0.258 mmol, 97%) was obtained as a yellow-white solid, essentially pure by its ^{1}H NMR spectrum. Crystals suitable for X-ray crystallography were obtained by slow evaporation from an EtOH solution of complex **61**.

^{1}H-NMR (400 MHz, CD_3OD): δ (ppm) = 7.40 (s, 1H), 7.07 (s, 2H), 6.58 (s, 2H), 4.85 – 4.66 (m, 2H), 3.87 (s, 2H), 3.63 (s, 3H), 1.03 (s, 3H).

^{13}C{^{1}H}-NMR (101 MHz, CD_3OD): δ (ppm) = 169.0, 165.3, 160.1, 139.3, 131.7, 130.2, 123.3, 120.2, 114.6, 96.2, 60.9, 55.6, 18.4, 14.8.

IR (Diamond-ATR, neat) ν_{max} (cm^{-1}) = 1623, 1511, 1273, 1255, 1242, 1231, 1172, 1034, 784, 634.

HRMS (ES-ToF) *m/z*: [M - Na + 2 H]$^+$ calc. for $(C_{56}H_{62}LaN_8O_{16})^+$: 1241.3342, found: 1241.3403.

m.p.: 176 – 178 °C (EtOH).

Anal. Calcd for $NaC_{56}H_{60}LaN_8O_{16}$: C, 53.25; H, 4.79; N, 8.87. Found: C, 52.86; H, 4.93; N, 8.39.

Bis(aqua) tris((4-(ethoxycarbonyl)-1-phenyl-1*H*-pyrazol-5-yl)oxy)lanthanum (62)

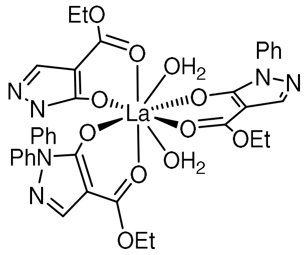

$La(OTf)_3$ (129 mg, 0.220 mmol, 1.00 equiv) was added with stirring to pyrazolone **6** (153 mg, 0.659 mmol, 3.00 equiv) in EtOH (3.5 mL) at 79 °C. Aqueous NaOH (2 M; 0.33 mL, 0.659 mmol, 3.00 equiv) was added with stirring and the mixture was heated at reflux for 20 h, after which the mixture was cooled to room temperature, the precipitate was collected by filtration and dried *in vacuo*. La-pyrazolone complex **62** (75 mg, 0.0901 mmol, 41%) was obtained as a yellow-white solid, essentially pure by its ^{1}H NMR spectrum. Crystals suitable for X-ray crystallography were obtained by slow evaporation from an EtOH solution of complex **62**.

^{1}H-NMR (400 MHz, CD_3OD): δ (ppm) = 7.96 (s, 1H), 7.70 – 7.63 (m, 2H), 7.40 (t, *J* = 7.8 Hz, 2H), 7.31 (t, *J* = 7.4 Hz, 1H), 4.29 (q, *J* = 7.1 Hz, 2H), 1.31 (t, *J* = 7.1 Hz, 3H).

^{13}C{^{1}H}-NMR (126 MHz, CD_3OD): δ (ppm) = 168.5, 163.2, 140.0, 138.2, 130.0, 128.0, 123.3, 123.0, 120.5, 96.7, 62.2, 14.7.

IR (Diamond-ATR, neat) ν_{max} (cm^{-1}) = 1669, 1635, 1600, 1551, 1498, 1420, 1383, 1256, 1230, 1170, 1035, 764, 631.

m.p.: 214 – 216 °C (EtOH).

Tris((4-benzoyl-1-(4-methoxybenzyl)-1*H*-pyrazol-5-yl)oxy)lanthanum (63)

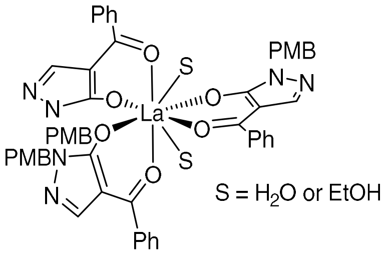

La(OTf)$_3$ (77.0 mg, 0.132 mmol, 1.00 equiv) was added with stirring to pyrazolone **10** (122 mg, 0.400 mmol, 3.00 equiv) in EtOH (3.5 mL) at 79 °C. Aqueous NaOH (2 M; 0.20 mL, 0.400 mmol, 3.00 equiv) was added with stirring and the mixture was heated at reflux for 20 h, after which the mixture was co-evaporated to dryness with Et_2O (4 x 15 mL). The residue was suspended in Et_2O, and the solid collected by filtration and dried *in vacuo*. La-pyrazolone complex **63** (105 mg, 0.100 mmol, 75%) was obtained as a white solid, essentially pure by its ^{1}H NMR spectrum.

^{1}H-NMR (400 MHz, CD_3OD): δ (ppm) = 7.60 (s, 1H), 7.34 (s, 1H), 7.19 (s, 2H), 7.11 (s, 1H), 6.90 (s, 1H), 6.53 (s, 1H), 6.36 – 6.31 (m, 2H), 4.81 (s, 1H), 3.48 (s, 3H).

^{13}C{^{1}H}-NMR (101 MHz, CD_3OD): δ (ppm) = 189.1, 165.4, 160.0, 141.9, 140.5, 132.2, 130.2, 129.9, 129.2, 129.1, 114.6, 106.4, 55.6, 55.5.

IR (Diamond-ATR, neat) ν_{max} (cm^{-1}) = 1596, 1497, 1480, 1437, 1288, 1244, 1232, 1172, 1036, 896, 803, 750, 703, 634.

m.p.: 223 – 225 °C (EtOH).

Anal. Calcd for $C_{54}H_{45}LaN_6O_9 \cdot 3\ H_2O \cdot$ EtOH: C, 57.93; H, 4.95; N, 7.24. Found: C, 58.02; H, 4.94; N, 5.83.

Tris((4-(ethoxycarbonyl)-1*H*-pyrazol-5-yl)oxy)lanthanum (64)

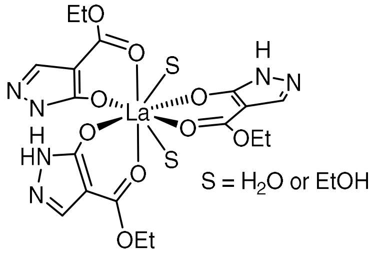

La(OTf)$_3$ (140 mg, 0.239 mmol, 1.00 equiv) was added with stirring to pyrazolone **5** (112 mg, 0.717 mmol, 3.00 equiv) in EtOH (3.5 mL). Aqueous NaOH (2 M; 0.36 mL, 0.717 mmol, 3.00 equiv) was added with stirring and the mixture was heated at reflux for 20 h, after which the mixture was co-evaporated to dryness with Et_2O (4 x 15 mL). The residue was suspended in Et_2O, and the solid collected by filtration and dried *in vacuo*. La-pyrazolone complex **64** (102 mg, 0.169 mmol, 71%) was obtained as a white solid, essentially pure by its ^{1}H NMR spectrum.

^{1}H-NMR (400 MHz, CD_3O): δ (ppm) = 7.42 (s, 1H), 3.99 (s, 2H), 1.22 – 1.12 (m, 3H).

^{13}C{^{1}H}-NMR (126 MHz, CD_3OD): δ (ppm) = 168.8, 167.1, 140.7, 94.2, 61.1, 14.9.

IR (Diamond-ATR, neat) ν_{max} (cm^{-1}) = 3299, 1644, 1570, 1523, 1441, 1290, 1258, 1171, 1126, 1034, 922, 783, 752, 641.

m.p.: 252 – 255 °C (EtOH).

Sodium(I) tetrakis((4-(ethoxycarbonyl)-1-(4-methoxybenzyl)-1H-pyrazol-5-yl)oxy)dysprosium (65)

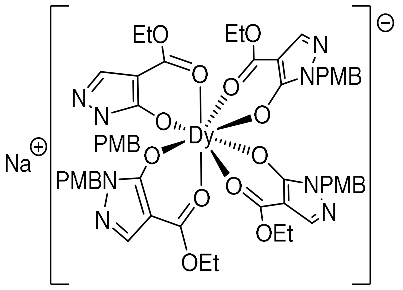

$DyCl_3$•$6H_2O$ (95.0 mg, 0.252 mmol, 1.00 equiv) was added with stirring to pyrazolone **8** (209 mg, 0.757 mmol, 3.00 equiv) in EtOH (4.5 mL) at 79 °C. Aqueous NaOH (2 M; 0.38 mL, 0.757 mmol, 3.00 equiv) was added with stirring and the mixture was heated at reflux for 20 h. H_2O was added (20 mL), the mixture was cooled to room temperature, and the resultant precipitate was collected by filtration, washed with H_2O (3 x 10 mL) and dried *in vacuo*. Dy-pyrazolone complex **65** (165 mg, 0.128 mmol, 51%) was obtained as a yellow-white solid. Crystals suitable for X-ray crystallography were obtained by slow evaporation from an EtOH solution of complex **65**.

Repeating the reaction with $Dy(OTf)_3$ (200 mg, 0.328 mmol, 1.00 equiv), pyrazolone **8** (272 mg, 0.984 mmol, 3.00 equiv) and aqueous NaOH (2M, 0.49 mL, 0.984 mmol, 3.00 equiv) according to the above procedure resulted in an improved yield of Dy-pyrazolone complex **65** (285 mg, 0.222 mmol, 68%).

IR (Diamond-ATR, neat) ν_{max} (cm^{-1}) = 1616, 1510, 1430, 1318, 1243, 1215, 1175, 1090, 1027, 782.

HRMS (ESI) *m/z*: $[M – L + H]^+$ calc. for $(C_{42}H_{46}DyN_6O_{12})^+$: 990.2460, found: 990.2491.

m.p.: 170 – 173°C (EtOH).

Anal. Calcd for $C_{56}H_{60}DyN_8NaO_{16} \cdot H_2O \cdot 2$ EtOH: C, 51.59; H, 5.34; N, 8.02. Found: C, 51.84; H, 4.70; N, 7.81.

Tris((4-(ethoxycarbonyl)-1-phenyl-1*H*-pyrazol-5-yl)oxy)dysprosium (66)

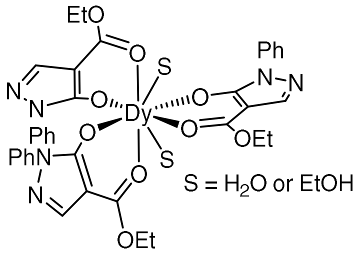

$DyCl_3$•$6H_2O$ (101 mg, 0.267 mmol, 1.00 equiv) was added with stirring to pyrazolone **6** (186 mg, 0.801 mmol, 3.00 equiv) in EtOH (4.5 mL) at 79 °C. Aqueous NaOH (2 M; 0.16 mL, 0.801 mmol, 3.00 equiv) was added with stirring and the mixture was heated at reflux for 20 h. H_2O was added (20 mL), the mixture was cooled to room temperature, the precipitate was collected by filtration, washed with H_2O (3 x 10 mL) and dried *in vacuo*. Dy-pyrazolone complex **66** (115 mg, 0.134 mmol, 50%) was obtained as an off-white solid. Crystals suitable for X-ray crystallography were obtained by slow evaporation from an EtOH solution of complex 66.

Repeating the reaction with $Dy(OTf)_3$ (195 mg, 0.320 mmol, 1.00 equiv), pyrazolone **6** (223 mg, 0.960 mmol, 3.00 equiv) and aqueous NaOH (2M, 0.48 mL, 0.960 mmol, 3.00 equiv) according to the above procedure resulted in an improved yield of Dy-pyrazolone complex **66** (237 mg, 0.277 mmol, 87%).

IR (Diamond-ATR, neat) ν_{max} (cm^{-1}) = 1636, 1596, 1552, 1528, 1497, 1429, 1359, 1333, 1237, 1211, 1118, 1082, 1060, 951, 782, 747.

m.p.: 148 – 150 °C (EtOH); >250 °C (decomp.).

Anal. Calcd for $C_{36}H_{39}N_6O_{12}Dy$: C, 47.50; H, 4.32; N, 9.23. Found: C, 47.23; H, 4.28; N, 7.80.

Tris((4-benzoyl-1-(4-methoxybenzyl)-1*H*-pyrazol-5-yl)oxy)dysprosium (67)

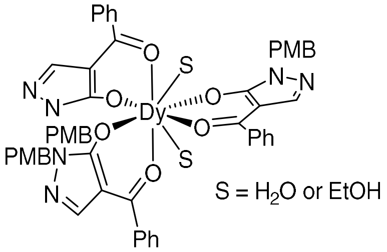

Dy(OTf)$_3$ (147 mg, 0.241 mmol, 1.00 equiv) was added with stirring to pyrazolone **10** (223 mg, 0.723 mmol, 3.00 equiv) in EtOH (5.5 mL) at 79 °C. Aqueous NaOH (2 M; 0.36 mL, 0.723 mmol, 3.00 equiv) was added with stirring and the mixture was heated at reflux for 16 h. H_2O was added (30 mL), the mixture was cooled to room temperature, and the resultant precipitate was collected by filtration, washed with H_2O (3 x 10 mL) and dried *in vacuo*. Dy-pyrazolone complex **67** (121 mg, 0.112 mmol, 46%) was obtained as an off-white-yellow solid.

IR (Diamond-ATR, neat) ν_{max} (cm^{-1}) = 1597, 1573, 1510, 1499, 1482, 1437, 1394, 1314, 1243, 1217, 1174, 1027, 898, 751, 703.

HRMS (ESI) *m/z*: $[M + H]^+$ calc. for $(C_{54}H_{46}DyN_6O_9)^+$: 1086.2612, found: 1086.2613.

m.p.: 182 – 184 °C (EtOH); >250 °C (decomp.).

Anal. Calcd for $C_{58}H_{59}N_6O_{12}Dy$: C, 58.31; H, 4.98; N, 7.03. Found: C, 58.76; H, 4.90; N, 6.79.

Tris((4-(ethoxycarbonyl)-1*H*-pyrazol-5-yl)oxy)dysprosium (68)

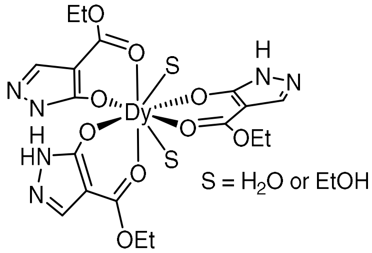

Dy(OTf)$_3$ (163 mg, 0.267 mmol, 1.00 equiv) was added with stirring to pyrazolone **5** (125 mg, 0.801 mmol, 3.00 equiv) in EtOH (6.0 mL) at 79 °C. Aqueous NaOH (2 M; 0.40 mL, 0.801 mmol, 3.00 equiv) was added with stirring and the mixture was heated at reflux for 17 h. H_2O (30 mL) was added, the mixture was cooled to room temperature, and the resultant precipitate was collected by filtration, washed with H_2O (3 x 10 mL) and dried *in vacuo*. Dy-pyrazolone complex **68** (88.0 mg, 0.140 mmol, 52%) was obtained as a white solid.

IR (Diamond-ATR, neat) ν_{max} (cm^{-1}) = 3329, 1636, 1560, 1522, 1512, 1457, 1437, 1314, 1245, 1172, 1124, 1037, 924, 900, 786, 753.

m.p.: >250 °C (EtOH) (decomp.).

Anal. Calcd for $C_{18}H_{21}N_6O_9Dy \cdot 1.5\ H_2O \cdot EtOH$: C, 34.27; H, 4.31; N, 11.99. Found: C, 34.36; H, 3.51; N, 11.90.

Tris((4-(ethoxycarbonyl)-1-(4-methoxybenzyl)-1*H*-pyrazol-5-yl)oxy)ytterbium (69)

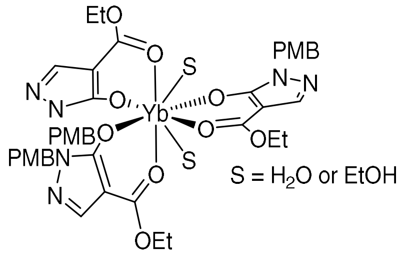

$Yb(OTf)_3$ (138 mg, 0.223 mmol, 1.00 equiv) was added with stirring to pyrazolone **8** (185 mg, 0.670 mmol, 3.00 equiv) in EtOH (4.0 mL). Aqueous NaOH (2 M; 0.33 mL, 0.670 mmol, 3.00 equiv) was added with stirring and the mixture was heated at reflux for 48 h, after which the mixture was cooled to room temperature, H_2O (20 mL) was added, and the resultant precipitate was collected by filtration, washed with H_2O (3 x 10 mL) and dried *in vacuo*. Yb-pyrazolone complex **69** (174 mg, 0.174 mmol, 78%) was obtained as a white solid.

IR (Diamond-ATR, neat) ν_{max} (cm^{-1}) = 1646, 1560, 1523, 1511, 1419, 1247, 1231, 1216, 1172, 1034, 785, 633.

HRMS (ES-ToF) *m/z*: $[M - Sol + H]^+$ calc. for $(C_{42}H_{46}N_6O_{12}Yb)^+$: 1000.2557, found: 1000.2607.

m.p.: 232 - 234 °C (EtOH) (decomp.).

Tris((4-(ethoxycarbonyl)-1-phenyl-1*H*-pyrazol-5-yl)oxy)ytterbium (70)

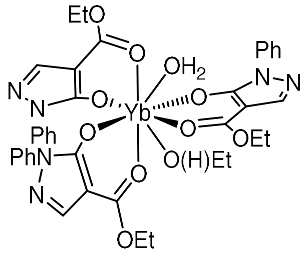

$Yb(OTf)_3$ (166 mg, 0.267 mmol, 1.00 equiv) was added with stirring to pyrazolone **6** (186 mg, 0.801 mmol, 3.00 equiv) in EtOH (4.0 mL) at 79 °C. Aqueous NaOH (2 M; 0.40 mL, 0.801 mmol, 3.00 equiv) was added with stirring and the mixture was heated at reflux for 4 h, after which the mixture was cooled to room temperature, H_2O (20 mL) was added, and the resultant precipitate was collected by filtration, washed with H_2O (3 x 10 mL) and dried *in vacuo*. Yb-pyrazolone complex **70** (211 mg, 0.227 mmol, 85%) was obtained as a white solid. Crystals suitable for X-ray crystallography were obtained by slow evaporation from an EtOH solution of complex **70**.

IR (Diamond-ATR, neat) ν_{max} (cm^{-1}) = 1647, 1598, 1555, 1531, 1500, 1433, 1256, 1232, 1171, 1120, 1085, 1036, 952, 785, 754, 632.

m.p.: >140 °C (EtOH) (decomp.); 208 – 210 °C.

Anal. Calcd for $C_{36}H_{33}N_6O_9Yb \cdot H_2O \cdot EtOH \cdot 1.3\ Yb_2O_3$: C, 31.84; H, 2.88; N, 5.86. Found: C, 31.69; H, 2.78; N, 5.46.

Tris((4-benzoyl-1-(4-methoxybenzyl)-1*H*-pyrazol-5-yl)oxy)ytterbium (71)

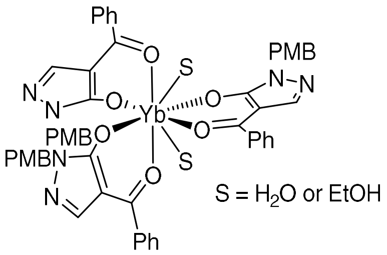

$Yb(OTf)_3$ (111 mg, 0.178 mmol, 1.00 equiv) was added with stirring to pyrazolone **10** (165 mg, 0.535 mmol, 3.00 equiv) in EtOH (4.0 mL). Aqueous NaOH (2 M; 0.27 mL, 0.535 mmol, 3.00 equiv) was added with stirring and the mixture was heated at reflux for 24 h, after which the mixture was cooled to room temperature, H_2O (20 mL) was added, and the resultant precipitate was collected by filtration, washed with H_2O (3 x 10 mL) and dried *in vacuo*. Yb-pyrazolone complex **71** (133 mg, 0.121 mmol, 68%) was obtained as a white solid.

IR (Diamond-ATR, neat) ν_{max} (cm^{-1}) = 1597, 1571, 1508, 1499, 1482, 1437, 1396, 1243, 1172, 1036, 898, 804, 751, 702, 634.

HRMS (ES-ToF) *m/z*: $[M - Sol + H]^+$ calc. for $(C_{54}H_{46}N_6O_9Yb)^+$: 1096.2709, found: 1096.2882.

m.p.: >140 °C (EtOH) (decomp.); 215 – 217 °C.

Tris((4-(ethoxycarbonyl)-1*H*-pyrazol-5-yl)oxy)ytterbium (72)

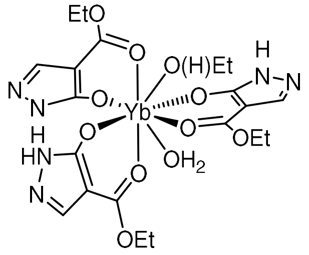

$Yb(OTf)_3$ (197 mg, 0.318 mmol, 1.00 equiv) was added with stirring to pyrazolone **5** (149 mg, 0.954 mmol, 3.00 equiv) in EtOH (5.0 mL). Aqueous NaOH (2 M; 0.48 mL, 0.954 mmol, 3.00 equiv) was added with stirring and the mixture was heated at reflux for 24 h, after which the mixture was cooled to room temperature, H_2O (20 mL) was added, and the resultant precipitate was collected by filtration, washed with H_2O (3 x 10 mL) and dried *in vacuo*. Yb-pyrazolone complex **72** (210 mg, 0.299 mmol, 94%) was obtained as a white solid.

IR (Diamond-ATR, neat) ν_{max} (cm^{-1}) = 3670, 1653, 1647, 1570, 1522, 1442, 1256, 1231, 1170, 1136, 1034, 921, 783, 764, 640, 633.

HRMS (ES-ToF) *m/z*: $[M - Sol + H]^+$ calc. for $(C_{18}H_{22}N_6O_9Yb)^+$: 640.0831, found: 640.0593.

m.p.: 234 – 236 °C (EtOH).

Bis(4-(ethoxycarbonyl)-1-(4-methoxybenzyl)-1*H*-pyrazol-5-olate)dioxouranium (73)

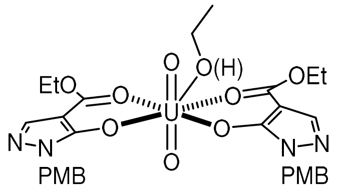

$UO_2(NO_3)_2$•$6H_2O$ (101 mg, 0.201 mmol, 1.00 equiv) was added with stirring to pyrazolone **8** (110 mg, 0.398 mmol, 2.00 equiv) and pyridine (0.50 mL) in EtOH (5.00 mL) at 79 °C. The mixture was heated at reflux for 16 h, cooled to room temperature, when Et_2O (30 mL) and hexanes (20 mL) were added and the precipitate of pyridinium nitrate was removed by filtration. Uranyl-pyrazolone complex **73** (119 mg, 0.137 mmol, 68%) was obtained as orange solid by slow evaporation from the ethanolic filtrate.

Bis(4-(ethoxycarbonyl)-1-phenyl-1*H*-pyrazol-5-olate)dioxouranium (74)

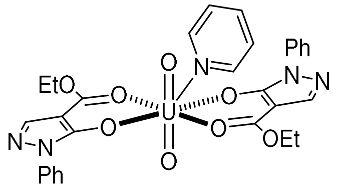

$UO_2(NO_3)_2$•$6H_2O$ (200 mg, 0.398 mmol, 1.00 equiv) was added with stirring to pyrazolone **6** (185 mg, 0.797 mmol, 2.00 equiv) and pyridine (3.00 mL) in EtOH (7 mL) at 79 °C. The mixture was heated at reflux for 16 h, cooled to room temperature. Et_2O (30 mL) and hexanes (20 mL) were added and the precipitate of pyridinium nitrate was removed by filtration. Uranyl-pyrazolone complex **74** (118 mg, 0.145 mmol, 37%) was obtained as a fluorescent-yellow solid by slow evaporation from the ethanolic pyridine filtrate.

IR (Diamond-ATR, neat) ν_{max} (cm^{-1}) = 1621, 1596, 1532, 1496, 1443, 1437, 1324, 1249, 1157, 1086, 952, 926, 698.

Bis(4-benzoyl-1-(4-methoxybenzyl)-1*H*-pyrazol-5-olate)dioxouranium (75)

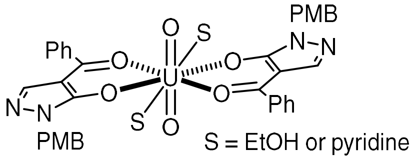

$UO_2(NO_3)_2$•$6H_2O$ (149 mg, 0.297 mmol, 1.00 equiv) was added with stirring to pyrazolone **10** (183 mg, 0.593 mmol, 2.00 equiv) and pyridine (3.00 mL) in EtOH (7 mL) at 79 °C. The mixture was heated at reflux for 16 h, cooled to room temperature, when Et_2O (30 mL) and hexanes (20 mL) were added and the precipitate of pyridinium nitrate was removed by filtration. Uranyl-pyrazolone complex **75** (180 mg, 0.187 mmol, 63%) was obtained as orange-red solid by slow evaporation from the ethanolic pyridine filtrate.

Bis((4-(ethoxycarbonyl)-1*H*-pyrazol-5-yl)oxy)dioxouranium (76)

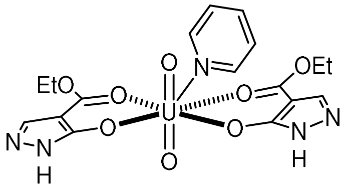

$UO_2(NO_3)_2$•$6H_2O$ (177 mg, 0.352 mmol, 1.00 equiv) was added with stirring to pyrazolone **5** (110 mg, 0.704 mmol, 2.00 equiv) and pyridine (0.50 mL) in EtOH (5.0 mL) at 79 °C. The mixture was heated at reflux for 16 h, cooled to room temperature, when Et_2O (30 mL) and hexanes (20 mL) were added and

the precipitate of pyridinium nitrate was removed by filtration. Uranyl-pyrazolone complex **76** (146 mg, 0.221 mmol, 63%) was obtained as fluorescent yellow solid by slow evaporation from the ethanolic filtrate.

III. References

[1] Mies, T.; Schürmann, C.; Ito, S.; White, A. J. P.; Crimmin, M. R.; Barrett, A. G. M. Synthesis and Characterization of a Calcium-Pyrazolonato Complex. Observation of *In-Situ* Desolvation During Micro-Electron Diffraction. *Z. Anorg. Allg. Chem.* **2023**, *649*, e202200294. ChemRxiv preprint DOI: https://doi.org/10.26434/chemrxiv-2022-q51s2.

[2] Ohle, H.; Melkonian, G. A. Flavazol, II. Mitteil.: Der Bau des Ringsystems. *Ber. Dtsch. Chem. Ges. A/B* **1941**, *74*, 398–408.

[3] (*a*) Taylor, A. W.; Cook, R. T. A direct preparation of 2-aryl-4-ethoxycarbonyl-3- pyrazolin-5-ones from aryl hydrazines. *Tetrahedron* **1987**, *43*, 607–616. (*b*) Holzer, W.; Schmid, E. NMR spectroscopic investigations with ethyl 1-(hetero)aryl-5-hydroxy-1H-pyrazole-4-carboxylates. *J. Het. Chem.* **1995**, *32*, 1341–1349.

[4] Eller, G. A.; Holzer, W. The 4-Methoxybenzyl (PMB) Function as a Versatile Protecting Group in the Synthesis of N-Unsubstituted Pyrazolones. *Heterocycles* **2004**, *63*, 2537–2555.
